# A general framework for modeling growth and division of mammalian cells

**DOI:** 10.1186/1752-0509-5-3

**Published:** 2011-01-06

**Authors:** John H Gauthier, Phillip I Pohl

**Affiliations:** 1 Sandia National Laboratories, P.O. Box 5800, Albuquerque, New Mexico, USA 87185-1188

## Abstract

**Background:**

Modeling the cell-division cycle has been practiced for many years. As time has progressed, this work has gone from understanding the basic principles to addressing distinct biological problems, e.g., the nature of the restriction point, how checkpoints operate, the nonlinear dynamics of the cell cycle, the effect of localization, etc. Most models consist of coupled ordinary differential equations developed by the researchers, restricted to deal with the interactions of a limited number of molecules. In the future, cell-cycle modeling--and indeed all modeling of complex biologic processes--will increase in scope and detail.

**Results:**

A framework for modeling complex cell-biologic processes is proposed here. The framework is based on two constructs: one describing the entire lifecycle of a molecule and the second describing the basic cellular machinery. Use of these constructs allows complex models to be built in a straightforward manner that fosters rigor and completeness. To demonstrate the framework, an example model of the mammalian cell cycle is presented that consists of several hundred differential equations of simple mass action kinetics. The model calculates energy usage, amino acid and nucleotide usage, membrane transport, RNA synthesis and destruction, and protein synthesis and destruction for 33 proteins to give an in-depth look at the cell cycle.

**Conclusions:**

The framework presented here addresses how to develop increasingly descriptive models of complex cell-biologic processes. The example model of cellular growth and division constructed with the framework demonstrates that large structured models can be created with the framework, and these models can generate non-trivial descriptions of cellular processes. Predictions from the example model include those at both the molecular level--e.g., Wee1 spontaneously reactivates--and at the system level--e.g., pathways for timing-critical processes must shut down redundant pathways. A future effort is to automatically estimate parameter values that are insensitive to changes.

## Background

Most researchers keep a serviceable mental model of how molecules of interest react with each other in various cellular pathways. Such qualitative models suffice for many purposes. They can, however, break down when trying to explain more complex interactions between molecules, e.g., when a molecule participates in multiple pathways; when the activity or the appearance of the molecule is dependent on multiple reactions; when the timing of reactions is important; when multiple processes interact, etc. For such reasons, quantitative modeling of complex biologic processes, such as the eukaryote cell cycle, has been practiced for many decades. (For a general review of cell-cycle modeling, see [1-3] and references therein.) These efforts have produced descriptions of cell-cycle subprocesses (e.g., [4]) and predictions (e.g., [5]; [6] testing of [7]) beyond the realm of mental models.

As the work reported here involves cell-cycle modeling as an example of modeling complex biologic processes, a brief summary and discussion of recent eukaryote cell-cycle work follows. Chen et al. [5,7] examined the cell-cycle regulation of budding yeast. Novak and Tyson [8] extended this work to mammalian cells, proceeding on a generalization by Nurse [9,10] that molecular cell-cycle controls are similar in all eukaryotes. Novak and Tyson also used the model to examine restriction point control. Other efforts have continued this trend of quantitative models being used to examine a number of specific cell-cycle subprocesses and extensions: localization [5,11], checkpoints [4,12], apoptosis [13], multisite phosphorylation [14], cell growth and size control [15,16], etc. Several efforts also have looked at the nonlinear dynamics of cell-cycle regulation [17-20].

In detail most of the models used in these efforts are different; however, in general they are constructed in a similar fashion. All consist of coupled ordinary differential equations (ODEs), typically based on wiring diagrams of the molecular pathways of importance to the cell cycle (e.g., the interaction of cyclins and cyclin-dependent kinases; for the ultimate molecular interaction map of the mammalian cell cycle, see [21]). The equations of state are based on specific molecules, and the solutions are for the concentration of each important molecule. Although most state equations are based on simple mass action kinetics, the rate constants are often enhanced with combinations of constants or with other functions (e.g., the Goldbeter-Koshland function used by [22] and [5] and discussed by [23]), presumably necessary to achieve appropriate behavior. An exception is the use of diffusion equations (and approximations thereof) for membrane transport in the work of Yang et al. [16]. Rate constants and initial concentrations are based, where possible, on experimental data; although as noted by [24], few if any of these values have been measured directly and they must be estimated, typically by trial and error. Cell division, when included in a model, is achieved by reducing the cell size by one half (e.g., [18]). Growth of the cell as well as transcription and translation of the regulatory proteins (not to mention the proteins that comprise the bulk of the cell) are infrequently accounted in these models (for an exception, see [11]). When growth is accounted, the purpose is to investigate size control--the idea that the cell must reach a certain size for some of the cell-cycle regulatory processes to occur. Yang et al. [16] used cell volume and nuclear-membrane surface area to model size control. Qu et al. [15] based size control on growth-signal transduction being proportional to membrane surface area. Csikász-Nagy et al. [18] discussed the synthesis rates of cyclins as a form of size control. Other aspects of cell growth e.g., energy usage or membrane synthesis, are not considered in these models. In these efforts, steady-state (G0) behavior of the models is rarely discussed.

The work presented here follows in the footsteps of the efforts discussed above. One missing element in these efforts, and other modeling efforts dealing with complex biologic processes, is a framework with which to capture the necessarily increasing scope and detail that such quantitative models will achieve as they progress. A framework for modeling the mammalian cell cycle is proposed here. The proposed framework is composed of two constructs, which describe (1) a molecule lifecycle and (2) basic cellular machinery--which is in turn built of molecule-lifecycle constructs. The constructs are based on techniques used in System Dynamics [25]. Although at first glance cumbersome, it offers a structure that readily allows modification and extension. A benefit of the framework is that it allows accounting of all processes that are important to a cell-biologic system in a straightforward and rigorous manner to the extent currently possible. Another benefit is that using the framework, a researcher can first concentrate on modeling each individual molecule, then on the interactions of the molecules, then on the interactions between the molecules and the functioning of the cell.

As in most previous work, the framework is based on coupled ODEs. A number of different computational techniques have been proposed and investigated for modeling biological pathways, including Stochastic Petri Nets (e.g., [26]), P Systems (e.g., [27]), Brane Calculus (e.g. [28]), Rule-Based Modeling (e.g., [29]), combination approaches (e.g., [30]), etc. For a general discussion see [31,32]. Each of these techniques has merit, although none solve the problem of complexity that will be encountered as models necessarily increase in scope and detail. Coupled ODEs are used here because they have been traditionally used, they can deal with large numbers of molecules and, with the framework described here, they can be derived and solved with relative facility. The implications of using coupled ODEs are discussed in Additional file 1.

To demonstrate the use of the framework constructs, a model of the growth and division of a mammalian cell is presented. The model consists of several hundred ODEs and parameters, but it tracks molecules from birth to death, accounts for nuclear compartmentalization, and operates respecting the underlying use of amino acids, nucleotides, ATP, mRNA, rRNA, etc. The model executes relatively quickly, but requires a stiff equation solver (Matlab http://www.mathworks.com function *ode15s*; similar to that used by [5]) because of the disparity in the timescales of the reactions. The model returns interesting, albeit unconfirmed, predictions. The model also demonstrates that the framework is useful, not the least because the major effort was not in the creation of the model, but in the calibration.

What likens the work presented here to other mammalian cell-cycle modeling efforts are (1) the use of ODEs to describe the mass action kinetics of chemical reactions and thereby provide a quantitative basis for the modeling (see Additional file 1), (2) the use of researcher-developed state and rate equations, based on literature sources where possible, and (3) the use of researcher-estimated parameters (see Additional file 2). A conscious attempt was made here to use the simplest form of mass action kinetic equation for each interaction, although some processes were simplified to the extent that more complicated expressions had to be used, e.g., for proton transport across the mitochondrial membrane. Some researchers have attempted to use a standard set of kinetic parameters and parameter estimation techniques (e.g., [4]); in this work, the need to meet multiple global and local constraints for calibration at both steady state and cell cycling militated against this approach. An attempt was made to match concentration values, which partially constrain kinetic rates, to those reported in the literature.

What differentiates this work from other efforts are the aspects that involve completeness and comprehensiveness that are allowed by the framework. The example model describes both steady-state (G0) behavior and dynamic growth and division of a cell, using the same set of kinetic parameters. The model describes in a comprehensive fashion many processes that are individually examined in other efforts: nuclear localization (see also [5,11,16]), membrane transport (see also [16]), and growth factors (although still "highly stylized," in the words of Novak and Tyson [8]). The model describes in a comprehensive fashion many processes that are omitted from other efforts: usage of sugars, ATP, nucleotides, and amino acids, synthesis of RNAs--mRNA, rRNA, tRNA, and snRNA--and the action of multiple transcription factors (E2F, B-Myb, NF-Y, and a generic TF-grow meant to represent c-myc, c-Jun, Notch, etc.). Further, the production and degradation of all molecules in the model are tracked (see also [11]). Details concerning features and operation of the model are presented in Additional file 3. As a final differentiation, this work is not directed at a specific biological issue--although some biological predictions are presented and an investigation of a redundant pathway is included in Additional file 4--rather, it addresses what will be necessary to progress the modeling of complex biologic processes.

## Results

### Molecule-lifecycle construct

**Figure 1:**
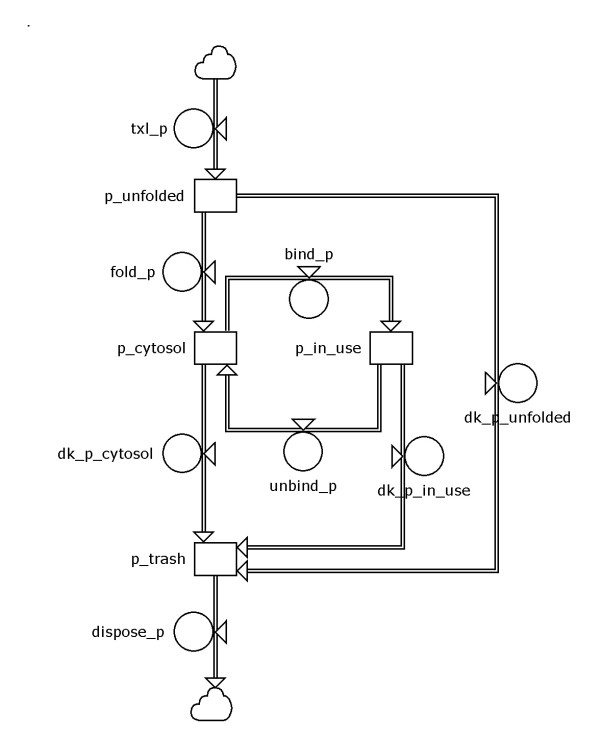
**Construct for the lifecycle of a protein**. Circles represent rate equations; rectangles represent state equations (see text). The protein is described from translation (txl_p), through folding (fold_p), through cyclical interaction with other molecules (bind_p and unbind_p), through decay (dk_p...) and disposal (dispose_p).

Figure 1 illustrates a construct that describes the lifecycle of a protein. Constructs such as these can also apply to other molecules in the cell, e.g., RNA, amino acids, ATP, etc. In the construct, the rectangles correspond to states, and the circles correspond to the rates that allow a molecule to go from one state to another. The cloud at the top of the construct symbolizes the start of the lifecycle, and proteins enter the lifecycle at the translation rate (txl_p). Proteins first enter the unfolded state (p_unfold), then they either decay (dk_p_unfold) to the trash state (p_trash) or are folded (fold_p) into proteins in the cytosol (p_cytosol). Once in the cytosol, this protein either decays (dk_p_cytosol) or binds another molecule (bind_p) and proceeds to the bound or active state (p_in_use). From the in-use state, the protein either decays (dk_p_in_use) or unbinds (unbind_p). Other details can be readily added to this construct, e.g., transfer between cellular compartments or interaction with other molecules. The lifecycle construct is so named because it can account for molecules continually being created and degraded and used and reused.

State and rate equations are assigned to the construct in a straightforward manner as follows. The change in the number of molecules in a given state is dependent on the number of molecules entering that state, minus the number of molecules leaving that state, as described by the ODE dC/dt = R_in _- R_out _, where C is the concentration of molecules of a given molecule type in a given state, t is time, dC/dt is the change over time in the concentration of molecules, R_in _is the rate of molecules entering the state, and R_out _is the rate of molecules leaving the state. Rates can apply to chemical reactions or transition from one compartment in a cell to another. Rates are typically calculated as the product of a rate constant and the concentration of one or more molecules: R = kC_1_C_2_..., where k is the rate constant, C_1 _is the concentration of the first reactant, C_2 _is the concentration of the second reactant, etc. Parameter values that are unknown and must be fitted to the model are the rate constants and the initial concentrations of the states. The construct shown in Figure 1 is a simplification; seven states typically represent the lifecycle for a protein in the example cell-cycle model, and five states represent the lifecycle of the mRNA for the protein. The most complex molecule lifecycle construct in the example model is for cycA; it includes 10 states and 23 rates describing interactions of cycA with Cdk2, p27, and Cdk1, as well as cycA activity in cytosol and in the nucleus (Additional file 5).

### Framework overview--cell growth

Underlying the example mammalian cell-cycle model is a construct that ties the cell cycle to the rest of the cell and ensures overall self-consistency: (1) it is used for calibration purposes (Additional file 2); (2) it calculates available quantities of cellular nucleotides (NT), amino acids (AA), RNA, ATP, proteins, and lipids over time; (3) it calculates the transcription rate and the translation rate for the entire cell; (4) it is used to determine the normalized size of the cell (assumed to be related to the number of proteins), which is used to estimate the concentrations of all molecules.

**Figure 2:**
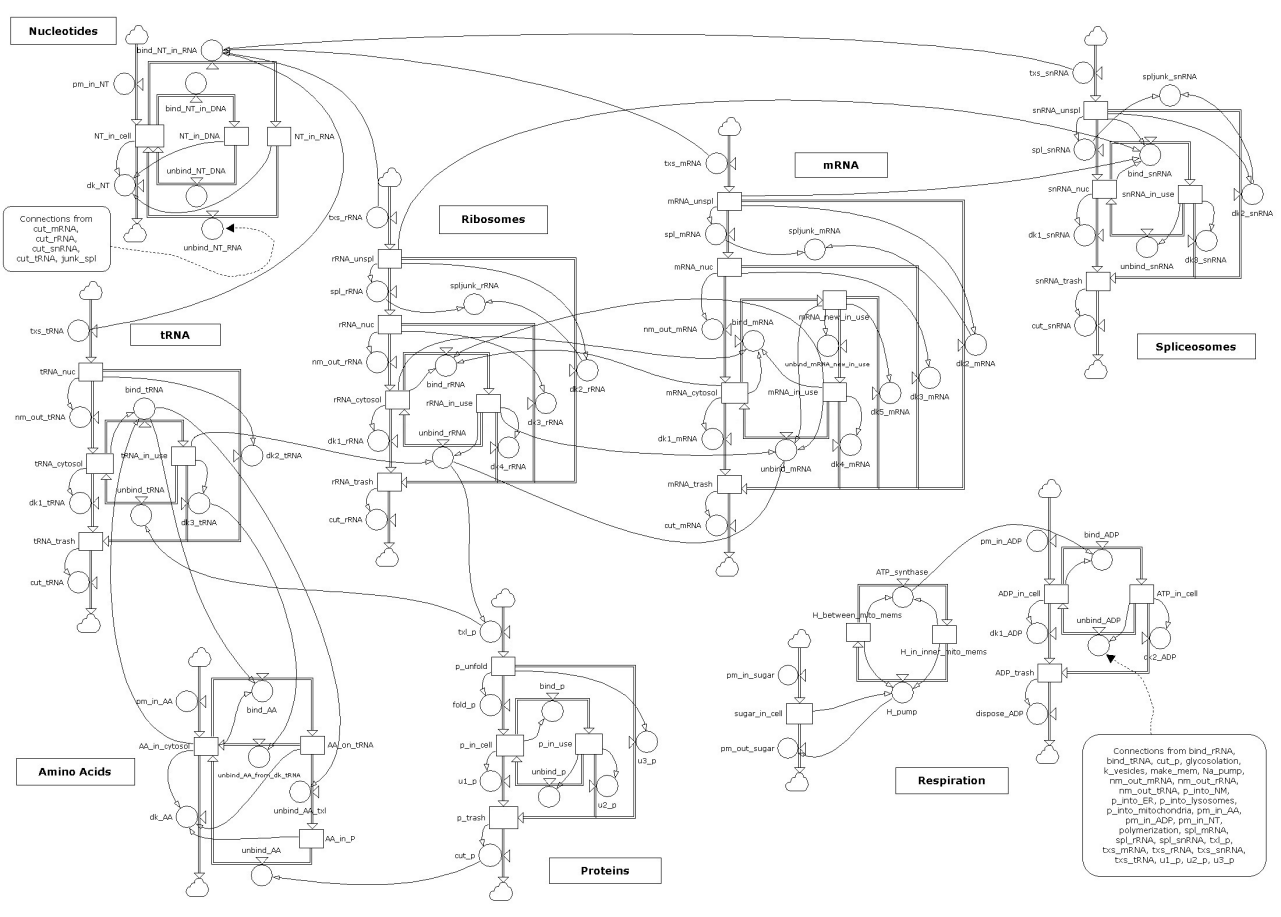
**Base model for keeping track of underlying cell processes**. Constructs represent generic molecule types, e.g., proteins, rRNA, spliceosomes, etc. Thick double lines indicate flows from one state (rectangle) to another controlled by the rate equations (circles); thin connecting lines indicate inputs to state and rate equations.

The basic-cellular-machinery model consists of 41 ODEs and 69 rate equations (Additional file 6). Figure 2 shows the framework of the model construct (lipids not shown). The molecular lifecycles interact through their rate equations; the thinner lines in Figure 2 indicate the interactions. For example, the number of tRNA being bound to amino acids (bind_tRNA) is a function of the number of amino acids available (AA_in_cytosol), which ultimately influences the number of proteins being translated (txl_p). In the diagram, the thin lines from the use of ATP are omitted, but they would extend to most of the rates.

### Framework overview--cell-cycle regulation

The cell-cycle model calculates the quantities of 33 proteins, and their concomitant 33 mRNAs, over time. These proteins constitute an approximate minimum set necessary to describe the mammalian cell cycle. Table 1 summarizes these proteins and their interactions. Illustrations of the lifecycle diagrams of these proteins are presented in Additional file 5. Many of these "proteins" are not strictly single molecules, but rather abstractions of proteins that include representative members of protein complexes and pathways (Additional file 1). The example model exclusive of the basic-cellular-machinery model consists of 387 ODEs and approximately 1100 rate equations (Additional file 6).

**Table 1:** Proteins modeled in the cell-cycle model.

	PROTEIN	LOC	G0	UBIQ	INDUCER	ACTIVATOR	INACTIVATOR	FUNCTION
1	p27	N	high	KPC, SCF(Skp2), APC(Cdc20)	constitutive			inhibits cyclins--prevent S

2	Rb	N	high		constitutive, E2F inhibits	Cdc14*	cycD/Cdk4or6, cycE/Cdk2, cycA/Cdk2, cycA/Cdk1, cycB/Cdk1	inhibits E2F

3	cycD	N	0	SCF(Skp2), APC(Cdc20)*	mitogen, B-Myb*	Cdk4or6		activates Cdk4or6 kinase; inactivates APC(Cdh1)

4	Cdk2	N	high		constitutive, E2F	cycE, cycA, Cdc25A		kinase--phosphorylates RC--allows DNA replication

5	cycE	N	0	SCF(Fbw7)--on Cdk2, SCF(Skp2)--free	E2F	Cdk2	p27	activates Cdk2 kinase

6	B-Myb	N	0	SCF(Skp2)--free	E2F, B-Myb	cycA/Cdk2, cycE/Cdk2*		TF for cycD, Cdk1, Plk1, DNA pol, B-Myb, TF-grow, others

7	NF-Y	N	0	?	constitutive, E2F	cycA/Cdk2, cycE/Cdk2*	Cdc14*	TF for cycA, cycB, Cdk1, Cdc25C, TF-grow

8	E2F	N	0	SCF(Skp2), APC(Cdc20)*	constitutive, E2F, B-Myb inhibits**		Rb, cycB/Cdk1, cycA/Cdk2**, cycA/Cdk1**, cycD/Cdk4or6**	TF for cycE, cycA, Rb, Cdk1, Cdk2, E2F, Cdk25A, DNA pol, others

9	cycA	N	0	APC(Cdh1), SCF(Skp2)**, APC(Cdc20)**	E2F, NF-Y, mitogen, adhesion**	Cdk2, Cdc25A, Cdk1, Cdc25A, Cdc25B, Cdc25C	p27 (for cycA/Cdk2), Wee1 (for cycA/Cdk1)	activates Cdk2 and Cdk1 kinases

10	SCF	CN	0	APC(Cdh1)	constitutive	Skp2, Btrc, Fbw7		ubiquitinase (requires subunit)

11	Skp2	N	0	auto (when no Emi1 or Wee1), APC(Cdh1)	constitutive	SCF		ubiquitinase subunit for p27, E2F, RC, TF-grow, B-Myb, free cycE, cycA, cycD, others

12	Btrc	C	0	auto (when no p27, cycE, E2F, or RC), APC(Cdh1), APC(Cdc20)	E2F	SCF		ubiquitinase subunit for Emi1, Cdc25A (sometimes), Wee1, others

13	Fbw7	N	0	auto (when no cycE, TF-grow**, or RC**)	E2F	SCF		ubiquitinase subunit for cycE, TF-grow, RC**

14	TF-grow (e.g., c-myc, c-Jun, Notch)	N	0	SCF(Skp2), SCF(Fbw7) (not ubiquitinated while TF-grow is on DNA)*	mitogen, Skp2, B-Myb			TF for cell growth

15	RC (e.g., hORC1, hCdc6)	N	0	SCF(Fbw7)**, SCF(Skp2), APC(Cdh1)	E2F	cycE/Cdk2, cycA/Cdk2, cycD/Cdk4or6	cycA/Cdk1, p27	DNA replication complex

16	DNA poly	N	0		E2F, B-Myb, NF-Y*, inhibits itself*	RC		DNA polymerase

17	Wee1	C	high	SCF(Btrc)	constituitive	Cdc14	cycA/Cdk2**, cycA/Cdk1**, cycB/Cdk1**, Plk1	kinase--prevents Cdk1 activation

18	cycB	CN	0	APC(Cdc20), APC(Cdh1)	E2F, B-Myb, NF-Y	Cdk1		activate Cdk1 kinase

19	Cdk1	CN	high*		E2F, B-Myb, NF-Y, constitutive	cycA, cycB, B-Myb, NF-Y, Plk1, Cdc25A, Cdc25B, Cdc25C	Wee1, Cdc14	kinase--activates APC(Cdc20)

20	Cdc25C	CN	high		constitutive, NF-Y**	cycB/Cdk1, Plk1, cycA/Cdk1*	Cdc14	phosphatase--activates cycB/Cdk1

21	Plk1	C	0	APC(Cdh1)	E2F, TF-grow	cycB/Cdk1, cycA/Cdk1*		kinase--activates Cdc25C; deactivates Emi1, Wee1; translocates cycA/Cdk1, cycB/Cdk1, Cdc25C, and Plk1 to nucleus

22	Emi1	CN	0	SCF(Btrc), SCF(Skp2)*	E2F		cycB/Cdk1, cycA/Cdk1*	inhibits Cdh1, Cdc20

23	APC	N	high		constitutive	Cdh1, Cdc20		ubiquitinase (requires subunit)

24	Cdh1	N	high	auto (when no Skp2, cycA, cycB, Cdc25A, Plk1, RC, Cdc20, SCF*, p27**)	constitutive	APC, Cdc14	Emi1, cycA/Cdk2, cycB/Cdk1, cycD/Cdk4or6, cycE/Cdk2	ubiquitinase subunit--maintains G0, G1--ubiq Cdc20, cycA (free), cycB (free), Cdc25A, RC, Plk1, Skp2, others

25	Cdc20	N	0	APC(Cdh1)	constitutive	APC, cycB/Cdk1, cycA/Cdk1*	Emi1, Cdc14	ubiquitinase subunit for Securin, cycB, cycA

26	Cdc14 (represents MEN pathway)	N	high		constitutive	Plk1*	Securin	phosphatase--ends M--activates p27, Wee1, Cdh1; deactivates Cdc25A, Cdc25B, Cdc25C

27	Cdc25A	N	0	APC(Cdh1), SCF(Btrc)*	constitutive, E2F, TF-grow	cycE/Cdk2, cycA/Cdk2, cycA/Cdk1*, cycB/Cdk1*	Cdc14	phosphatase--activates cycE/Cdk2, cycA/Cdk2, cycA/Cdk1*, cycB/Cdk1*

28	Cdc25B	C	0	APC(Cdh1)*, SCF(Btrc)*	E2F*, TF-grow*,	TF-grow**, cycA/Cdk1*, cycB/Cdk1*	Cdc14	phosphatase--activates cycA/Cdk1, cycB/Cdk1*

39	Securin	N	0	APC(Cdc20)	E2F			keeps separase from destroying chromotin cohesion proteins

30	cycC	N	high		constitutive	Cdk8, Cdc14*	mitogen*	inhibits RNA pol

31	KPC	N			constitutive	mitogen		ubiquitinase for p27

32	RNA poly	N	high		constitutive, TF_grow*	mitogen*	cycC/Cdk8, APC(Cdc20)*	txs

33	eIF-4	C	high		constitutive	mRNA		initiates txl

The proteins represent different layers of abstraction (see Additional file 1). LOC = location, N = nucleus, C = cytoplasm, G0 = steady-state concentration, UBIQ = ubiquitinating molecules, auto = autoubiquitination, INDUCER = molecule effecting transcription, ACTIVATOR = molecule promoting activity, TF = transcription factor, txs = transcription, txl = translation, ? = unknown, * = assumed, ** = ignored.

### Testing

A brief overview of the example cell-cycle model results are presented here. See Additional file 3 for a more complete description.

### Cell growth

The results start with a cell in steady state, G0 phase. The cell cycle then consists of doubling the cell's contents, followed by the division of the cell in two. G1 is the initial growth phase; S phase is when DNA is replicated; G2 is the later growth phase; M is the division phase. In the results presented here, G1 begins at 2×10^4 ^seconds (~0.25 days) with the imposition of mitogen and adhesion factors. Although no steady-state results are presented, the G0 values are indicated in the early time values in the plots that follow.

**Figure 3:**
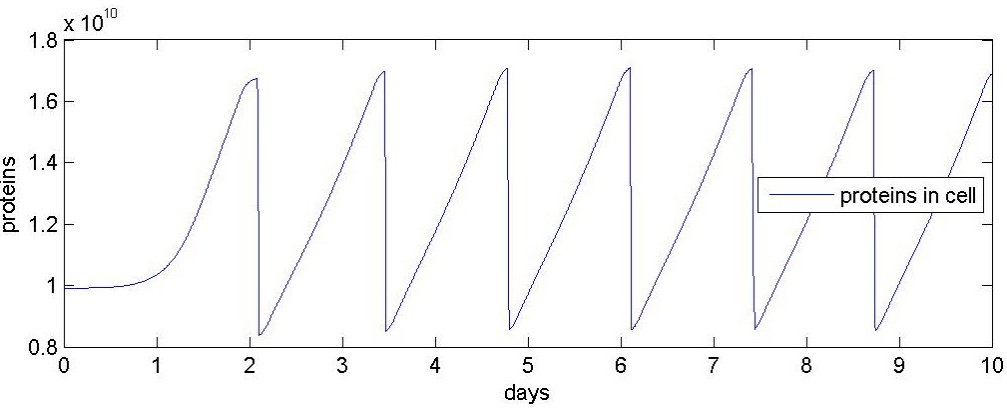
**Number of proteins in a single cell over 10 days**. The cell starts in G0, then cycles through seven divisions.

The example model tracks a single cell. Figure 3 shows how the number of proteins in this cell varies over 10 days. Protein number increases starting at 0.25 days and then suffers an abrupt decline at 2 days when the cell divides; the cycle is then repeated approximately every 1.3 days. The rate of growth slows near the peak of the curve, when transcription and translation slow during M phase. The peaks and troughs of each cycle are not equal (because the solution step occurs at a different point in the cell-division process for every cycle); however, the example model cycles within reasonable bounds for dozens of cell divisions.

**Figure 4:**
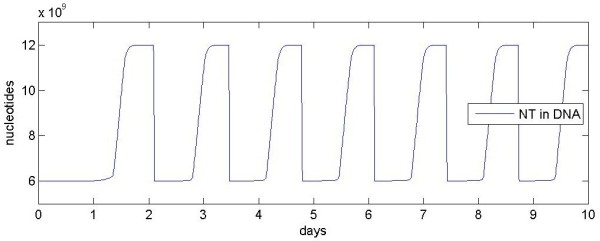
**Number of nucleotides in DNA in a single cell over 10 days**. Increasing slopes indicate S phase, maxima indicate G2 and M phases, decreasing slopes indicate cell division.

Figure 4 shows how the number of nucleotides in DNA varies over 10 days. A positive slope indicates S phase; the maxima indicate G2 and M; the abrupt negative slope indicates cell division. The first DNA replication is from approximately 1.3 days (the G1/S boundary) to approximately 1.6 days (the S/G2 boundary).

**Figure 5:**
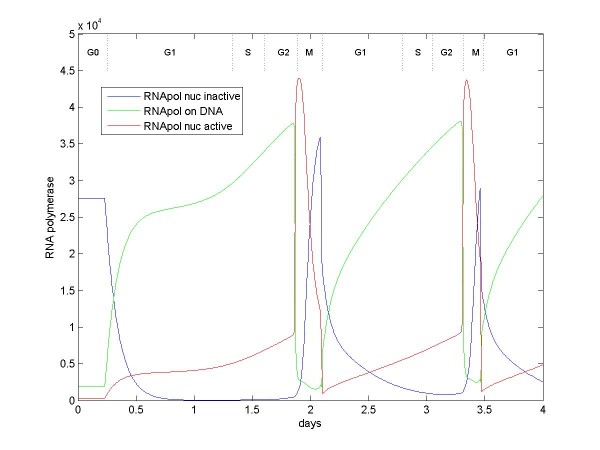
**Number of RNA polymerase over two cell divisions starting from G0**. RNA polymerase proteins are constitutive with enhancement by growth factors (TF-grow). The blue line indicates RNA polymerase that is held inactive by cycC/Cdk8; red line indicates RNA polymerase that is detached from DNA; green line indicates RNA polymerase that is actively transcribing DNA. In order to accomplish the initial cell division in a reasonable time, a large number of RNA polymerase is held inactive during G0.

In the model, cell growth rate is directly related to the number of active RNA polymerases. Figure 5 shows how numbers of RNA polymerase (the sum of polymerase type I, II, and III) vary from state to state as the cell cycle progresses. During G0 phase, ~3×10^4 ^RNA polymerases are present [33], but most are held inactive by cycC/Cdk8 [34,35]. The substantial excess of RNA polymerase is necessary for the cell cycle to initiate and progress at a reasonable rate; without this excess during G0, approximately a week is needed to build up enough RNA polymerase before the cell can divide. One prediction of the model (see Predictions below) is that maintaining this pool of RNA polymerase is a major determinant as to whether the cell is actually capable of dividing.

Mitogen causes inactivation of cycC/Cdk8 beginning at 2×10^4 ^seconds or ~0.25 days in the example model (mitogen activates KPC which ubiquitinates cycC/Cdk8), allowing most RNA polymerase to activate. In the model, active RNA polymerase continually attach and detach from DNA, polymerizing mRNA when attached. RNA polymerase numbers increase during the cell cycle because some of the new mRNA being generated is for new RNA polymerase; RNA polymerase transcription is enhanced by c-myc (represented in the model as part of a collection of molecules called TF-grow). RNA polymerase continues to transcribe mRNA throughout S phase (see Predictions). During M phase, initially at 1.8 days, APC(Cdc20) acts to release RNA polymerase from DNA. When initially unbound, these RNA polymerases are still active, but they are inactivated as Cdc14 reactivates cycC/Cdk8. Some RNA polymerase remains bound to DNA through the cell division (see Predictions).

The initial cell cycle after G0 is prolonged by a few hours while the active RNA polymerase builds the appropriate amounts of rRNA, tRNA, and DNA polymerase. In subsequent cell cycles, large amounts of these molecules plus activated RNA polymerase are available sooner, thus allowing for quicker growth and a shorter cycle time.

### Cell-cycle regulation

G0 steady state is maintained primarily by p27. Application of mitogen and adhesion factor initiates G1. The duration of the initial G1 is approximately 24 hours; subsequent G1 phases last approximately 15 hours; the initial G1 after G0 is timed by p27, subsequent G1 phases are timed primarily by APC(Cdh1). Mitogen cause the accumulation of cyclins and KPC. The presence of both mitogen and adhesion factor are required for cycA accumulation. The cyclins (cyc) bind with their appropriate kinases (Cdk); Cdks are for the most part constitutive. The cyc/Cdk are bound and inhibited by p27. cycD/Cdk4or6, whether bound to p27 or free, releases inhibitor Rb from the E2F transcription factor (see Predictions), E2F then induces many cell-cycle and cell-growth proteins.

Three other transcription factors are included in the example model: B-Myb, NF-Y, and a generic TF-grow, which is meant to represent c-myc, c-Jun, Notch, etc. These transcription factors induce a number of other molecules (Table 1). The amount of induction is calculated as the product of factors quantifying the visibility of the gene locus and the amount of the transcription factor (see Predictions). The amount of the transcription factor is often added to that of other transcription factors or a constitutive factor to determine the overall multiplicative effect. Transcription in total is limited by the number of RNA polymerase and nucleotides; thus, the transcription factors determine what fraction of transcription is devoted to a particular gene. Similarly, translation in total is limited by the number of ribosomes and amino acids; thus, the amount of each mRNA determines what fraction of translation is devoted to a particular protein.

The accumulating cyc/Cdk eventually bind all p27, allowing the accumulation of active (unbound) cyc/Cdk (especially cycE/Cdk2), which cause an abrupt release of the p27-bound cyc/Cdk. Free p27 are ubiquitinated by KPC.

A timing problem occurs because cycE, its activator Cdc25A, and its ubiquitinator Fbw7 are all induced at the same time in G1 by the E2F transcription factors. The model explains how the activities of these molecules occur at different times as follows. Constitutive APC(Cdh1) ubiquitinates both Cdc25A (which can activate cyc/Cdk) and SCF (which can inactivate cycE/Cdk2 with subunit Fbw7), but SCF is ubiquitinated more effectively than Cdc25A. Thus, as the concentration of active APC(Cdh1) decreases during G1 with the accumulation of cycD/Cdk4or6, first Cdc25A appears and abruptly activates the newly p27-unbound cycE/Cdk2 (causing the cycE/Cdk2-Cdc25A cascade), then SCF appears and binds Fbw7 and ubiquitinates cycE. (The cascade starts because of a low level of spontaneous dephosphorylation of cycE/Cdk2.) Ubiquitination of cycE subsequently frees Cdk2 for binding to cycA during S phase.

Iwamoto et al. [4] postulate an intermediate factor "X" for the delay between the accumulation of cycE and cycA during G1. The example model does include NF-Y as an additional transcription factor for cycA. The delay, however, is caused primarily by APC(Cdh1) ubiquitination of cycA (APC(Cdh1) does not interact with cycE). APC(Cdh1) is not included in the Iwamoto et al. model.

**Figure 6:**
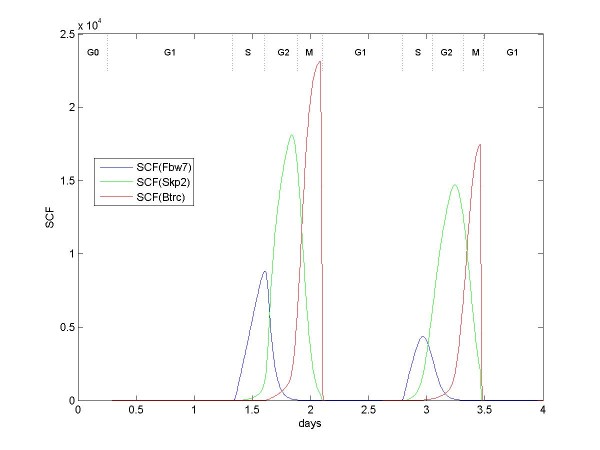
**Numbers of SCF bound to Fbw7, Skp2, and Btrc subunits over two cell divisions**. All molecules are present at the same time. First, SCF preferentially binds Fbw7 and ubiquitinates Fbw7 substrates, at which point Fbw7 autoubiquitinates. Then SCF preferentially binds Skp2 and ubiquitinates Skp2 substrates (in particular, replication complexes RC) at which point Skp2 autoubiquitinates. Finally, SCF binds Btrc, concluding the sequence.

Another timing problem occurs because SCF must bind, in turn, subunit Fbw7, then subunit Skp2, then subunit Btrc (Figure 6). The three subunits are present at the same time beginning in G1. This timing problem is solved in the model by SCF having greater affinity for Fbw7 than Skp2, and having greater affinity for Skp2 than Btrc. All of these SCF complexes autoubiquitinate when no substrate is present [36]. So, SCF preferentially binds Fbw7, which autoubiquitinates when cycE is gone; then SCF preferentially binds Skp2, which autoubiquitinates when p27, cycE, E2F, and RC (replication complex) are gone; then SCF binds Btrc. In the model, only the subunits autoubiquitinate, leaving SCF to immediately bind another subunit [37].

**Figure 7:**
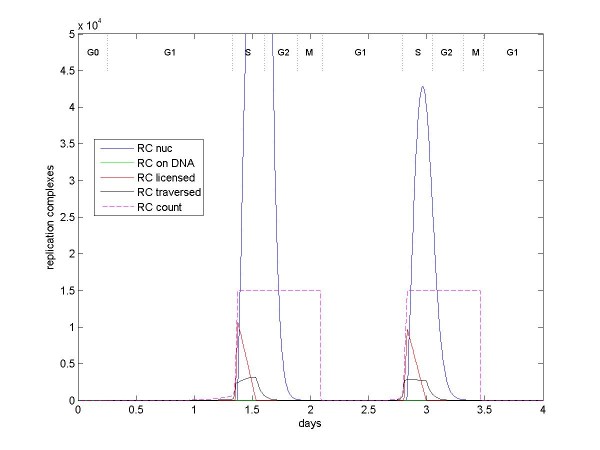
**Numbers of replication complex (RC) over two cell divisions**. RC (consisting of ORC1, Cdc6, etc.) is induced by E2F, accumulates in the nucleus, and binds DNA at 15,000 replication origins. RC is licensed by cyc/Cdk, primarily cycD/Cdk4or6 and cycE/Cdk2, after which RC is bound and traversed by DNA polymerase during DNA replication. Once traversed, the RC is released from the DNA and ubiquitinated, primarily by SCF(Skp2). The figure also shows a modeling construct, RC-count, which is used to set and maintain the 15,000-replication-orgin limit.

The cycE/Cdk2-Cdc25A cascade marks entry into S phase, which lasts approximately 8 hours. E2F induces proteins such as ORC1 and Cdc6 that contribute to DNA replication complexes (RC). RC mark 15,000 binding locations on DNA and, when activated by cycE/Cdk2 or cycA/Cdk2, allow binding of DNA polymerase to these locations and subsequent DNA replication [33]. Figure 7 indicates how RC are first bound to DNA, then how RC are licensed (activated by Cdk2), then how RC are traversed (bound) by DNA polymerase. The model includes continual RC binding and unbinding from DNA, as well as continual decay of the RC. DNA polymerase only attach and traverse licensed RC. Approximately 6000 DNA polymerase are present during S phase. After DNA polymerase bind the RC, the RC become delicensed. Delicensed RC are released from the DNA and ubiquitinated by SCF(Skp2) [38,39].

Growth continues throughout S phase. Therefore, the initiation of G2 is inexact. The initial G2 phase lasts approximately 12 hours; subsequent G2 phases last approximately 7 hours. Although the amount of DNA is double during G2, the transcription rate does not increase--transcription rate is dependent on the number of RNA polymerase.

**Figure 8:**
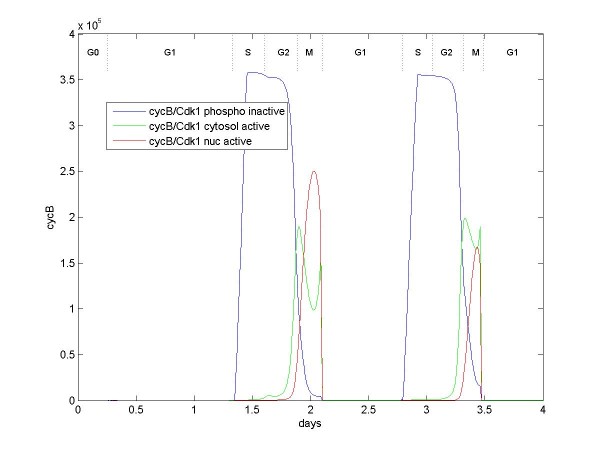
**Numbers of cycB/Cdk1 over two cell divisions**. cycB and Cdk1 are induced and enhanced by E2F, B-Myb, and NF-Y. Cdk1 also has constitutive expression. cycB/Cdk1 accumulates during S phase but is phosphorylated and inactivated by Wee1. Loss of Wee1 and the presence of active Cdc25B (activated by cycA/Cdk1) begin the dephosphorylation and activation of cycB/Cdk1 and thus trigger the cascade between cycB/Cdk1 and constitutive Cdc25C. Active cycB/Cdk1 further activates Plk1, which promotes the translocation of cycB/Cdk1 to the nucleus, completing the G2/M transition.

The termination of G2 occurs abruptly, with the cycB/Cdk1-Cdc25C cascade. Figure 8 shows that inactive cycB/Cdk1 begins accumulating at the beginning during S phase, but at a much faster rate than does cycA/Cdk1. The numbers of cycB/Cdk1 are much greater because of greater transcription and because most cycA binds nuclear Cdk2. In the model, Wee1 suppresses cycB/Cdk1 more strongly than it suppresses cycA/Cdk1. Hence, the activation cascade between cycB/Cdk1 and Cdc25C is delayed when compared with the cycA/Cdk1-Cdc25B cascade. As with cycA/Cdk1, cycB/Cdk1 is translocated to the nucleus after activation by Plk1.

In the model, there is included an intermediary step of a cycA/Cdk1-Cdc25B cascade, and this process could add more complication than necessary. Some evidence supporting this complication is suggested by [40-44]. This intermediary step does allow cycB/Cdk1 and Cdc25C to remain completely inactive during most of G2. Also, it offers an explanation why cycA binds Cdk1 and apparently participates in G2/M. And it offers an explanation why Cdc25B is apparently required for viability while Cdc25C apparently is not. (There is some disagreement in the literature about the necessity of Cdc25B and Cdc25C: Lincoln et al. [45] report that mice lacking Cdc25B are viable; Donzelli and Draetta [46] and Lindqvist et al. [44] report that Cdc25C knockout mice are viable, but Cdc25B knockouts are not, while Ferguson et al. [47] report that mice lacking both Cdc25B and Cdc25C are viable. However, Lammer et al. [41] report that Cdc25B is required for the human cell cycle.) In the model, Cdc25B can substitute for Cdc25C in the cycB/Cdk1-Cdc25C cascade, albeit less efficiently than Cdc25C, but Cdc25C cannot substitute in the cycA/Cdk1-Cdc25B cascade because it is very inactive. Therefore, Cdc25B is essential in the model, but Cdc25C is dispensable. As mentioned in Calibration of the Model (Additional file 2), however, calibration might be improved in these areas.

M phase lasts approximately 4 hours and follows the cycB/Cdk1-Cdc25C cascade. M phase is stylized in the example model. There is no explicit modeling of the subphases (prophase, metaphase, etc.) and the mechanics of division are implied by the lifecycles of a few molecules.

One consequence of the activation of cycB/Cdk1at G2/M is the phosphorylation of Emi1. Phosphorylation of Emi1 causes it to release Cdc20 and Cdh1. In the model, APC binding with Cdc20 is favored over its binding with Cdh1. APC(Cdc20) is activated (phosphorylated) in the nucleus by cycA/Cdk2, cycA/Cdk1, and cycB/Cdk1. APC(Cdc20) then ubiquitinates Securin.

**Figure 9:**
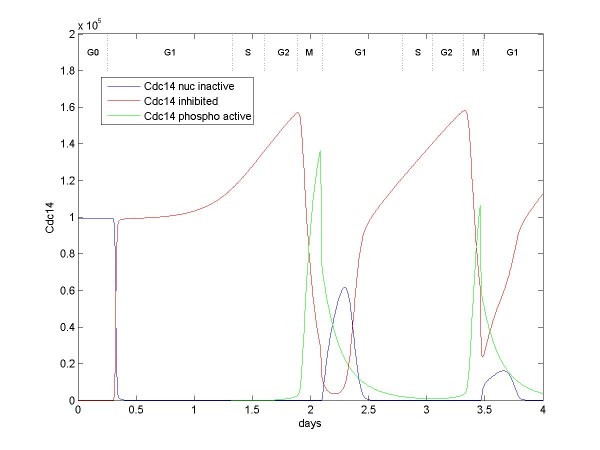
**Numbers of Cdc14 (representing the MEN pathway) over two cell divisions**. Cdc14 is a phosphatase that functions to activate p27, Wee1, and Cdh1 and deactivate the Cdc25 phosphatases, thus resetting the cell cycle after cell division. Cdc14 is constitutively expressed and rapidly translocated to the nucleus. Most of cell cycle, Cdc14 is inhibited by Securin. Cdc14 is activated when phosphorylated by nuclear Plk1after Securin is degraded.

Securin accumulation begins early in G1, culminating in its ubiquitination during and after G2/M. One role of Securin is to inhibit Cdc14 (Figure 9); once Securin is ubiquitinated, Cdc14 can be activated by Plk1. Another role of Securin is keep chromotids connected. With the ubiquitination of Securin, the chromosome pairs separate and the cell divides.

Figure 9 shows that quantities of the phosphatase Cdc14 exist in the nucleus throughout the cell cycle, although usually in an inactive or inhibited state. When active Plk1 moves into the nucleus, and when Securin has been ubiquitinated by APC(Cdc20), Plk1 can phosphorylate and activate Cdc14 (after [48]). In the model, Cdc14 represents the entire mitotic-exit-network (MEN) pathway. Cdc14 causes the cell to exit M phase by deactivating Cdc20, Cdc25A, Cdc25B, Cdc25C, and reactivating Wee1 (when it is again translated), Cdh1, and, in the model, cycC/Cdk8 (although no evidence could be found for this mechanism for reactivating cycC/Cdk8). Of immediate importance at the end of M phase is the unbinding of APC and Cdc20 by Cdc14. Unbinding of APC(Cdc20) allows the binding of APC and Cdh1, which causes the cell to enter either G0 or G1 (depending on the presence of mitogen), thus completing a cell cycle.

## Discussion

### Predictions

Predictions are here defined as necessary model features or model results that are not well known in the field of Cell Biology, that are not readily accepted, or that contradict current interpretations. More explanation of the evidence for predictions can be found in Additional files 3 and 4.

### System-level predictions

• When redundant pathways can activate a time-critical process, one preferred pathway must inactivate the other redundant pathways. This prediction was discovered studying the inactivation of Wee1 (by SCF(Btrc) or by Cdc25A). The explanation is that with redundant pathways, only one pathway must operate to assure correct timing; if both operate, the end reaction will proceed too quickly (unless both reactions are happening at a slower rate, say because of reduced concentration; however, these reactions would not be redundant). This finding has possible implications for understanding cycE-knockout and Cdc25C-knockout experiments (e.g., as discussed in [49,50]), because these knockouts could involve redundant pathways.

• Explicit growth monitoring is unnecessary during the mammalian cell cycle. Abundances of major reactants that advance the cell cycle are explicitly tied to overall cell growth, and the progression of the cell-cycle phases is, for the most part, dependent on cell growth. The ability to alter the timing of the cell cycle by artificially introduced regulatory proteins (as discussed by [49]) supports this finding. Novak and Tyson [8] propose that a "timer" process--similar to the prediction stated here--takes over in the cell cycle after a certain size is reached. Csikász-Nagy et al. [18] propose an "oscillator" process--similar to the prediction here--for quickly growing cells. Qu et al. [15] and Yang et al. [16] offer alternative viewpoints based on concentrations constrained by membrane transport.

• Pre-translation mRNA regulation (other than splicing or the number of RNA polymerase) is unnecessary during the cell cycle. Here, pre-translation mRNA regulation is defined as the process of limiting abundances of specific proteins by storing, destroying, or specifically selecting for translation their mRNA, e.g., by RNA interference or by storage of mRNA.

• Transcription and translation (i.e., growth) continues during S phase at unimpeded rates. If transcription and translation ceased during S phase, the duration of the modeled cell cycle would be extended by several hours, which would not match observations.

• Because a significant number of DNA polymerase is needed in short order during the initial cell cycle from G0, either a supply of DNA polymerase must be kept inactive during G0 or intense transcription and translation activity must occur and be controlled at a saturation level (as modeled).

• Cdc25A and Cdc25B cooperate to instigate the cycB/Cdk1-Cdc25C cascade and the G2/M transition. Evidence from the model is as follows. Negating the dephosphorylation of cycB/Cdk1 by Cdc25A lengthens G2 significantly. Similarly, reducing the ubiquitination of Cdc25A by SCF(Btrc) shortens G2 significantly. (If SCF(Btrc) is completely removed, however, the timing of G2/M is unaffected in the model and as noted experimentally by [51].) And negating the dephosphorylation of cycA/Cdk1 by Cdc25B also appreciably lengthens G2.

• During G0, a readily available supply of inactive RNA polymerase must be present in cells that are capable of dividing. In the model the supply is in the form of RNA polymerases held inactive by cycC/Cdk8. Without this supply, it would take approximately a week for the cell to produce enough RNA polymerases to initiate the first cell division after G0.

• SCF progressively complexes with Fbw7, Skp2, and Btrc through the cell cycle because of differences in affinity of these subunits with SCF and because of autoubiquitination in the absence of substrates.

• Part of the timing of G1/S is determined by a preference for APC(Cdh1) to ubiquitinate SCF over Cdc25A, allowing the cycE/Cdk2-Cdc25A cascade to proceed before SCF/Fbw7 ubiquitinates cycE.

• Transcription factors act multiplicatively.

• All cell-cycle reactions are interconnected with each other and with the basic cellular machinery.

### Molecular-level predictions

• p27 is important for maintaining G0 and for timing the duration of G1 in the first cell cycle after exiting G0. p27 does not have a significant role in timing G1 in continuous cell cycling. The duration of G1 in subsequent cell cycles is timed by APC(Cdh1).

• p27 does not inhibit cycD/Cdk4 or cycD/Cdk6 activity, although p27 does bind these complexes (as experimentally indicated by [52] and also suggested by [8]; Iwamoto et al. [4] assert that p27 binding activates cycD/Cdk4or6). The reason for this ineffectiveness is that growth induced by cycD/Cdk4or6 must proceed while p27 is still available, especially in consecutive cell cycles. p27 does block cycE/Cdk2 and is important in timing S phase in the first cycle after G0.

• cycD/Cdk4or6 does not activate DNA replication complexes. These cycD complexes are active throughout G1, S, and G2; if they activate RC, DNA replication would occur throughout much of the cell cycle.

• Wee1 spontaneously reactivates, either because of spontaneous dephosphorylation or because of continuous dephosphorylation by action of a phosphatase, perhaps PP2A [53]. There would otherwise be no need for SCF(Btrc) to ubiquitinate Wee1.

• Plk1 is the primary inactivator of Wee1 (via phosphorylation). Plk1 has more consistent active concentrations than other candidates (cycA/Cdk1 and cycB/Cdk1) over the first cycle after G0 and subsequent cycles.

• The reason why cycA binds Cdk1 is so that cycA/Cdk1 can react with either Cdc25A or Cdc25B and "prime the pump" for the cycB/Cdk1-Cdc25C cascade.

• Not all RNA polymerase are removed from condensed DNA during mitosis; the number attached is approximately the number active during G0.

### Parameter estimation

The parameters for the example model were determined by generate and test, albeit highly constrained by calibration (Additional file 2). Changes to some input parameters in the example cell-cycle model can currently cause large effects on the solution, including numerical instabilities. Evolution likely determined the set of initial values and rate constants that minimize sensitivity to change, and the model should be likewise calibrated. The author knows of no work in automatically determining the most robust set of parameters for a coupled-ODE model with multiple time-based constraints. Such a method would benefit not just cell-cycle modeling, but modeling complex biologic systems in general. This issue was also raised by [18].

### Possible improvements to the example model

Major areas of possible improvement to the example model include addressing many of the assumptions and simplifications (Additional file 1), improving calibration (Additional file 2), adding cell-cycle checkpoints, incorporating membrane receptors and growth-factor and adhesion-factor pathways, and adding chromatin manipulations during DNA replication [54] and M phase. Some preliminary work has indicated that these improvements are not inconsistent with the model or the framework.

## Conclusions

A framework for modeling complex cell-biologic pathways is described here. The framework includes a description of the entire lifecycles of the modeled molecules, combined with a description of the basic cellular machinery. These constructs foster completeness and rigor--qualities that will be necessary as biological models grow in scope and detail. The framework is applied to a model of the growth and division of mammalian cells. The example model has been exercised and found to generate non-trivial descriptions of the cell cycle, some of which could be worthy of experimental confirmation.

## Competing interests

The authors declare that they have no competing interests.

## Authors' contributions

JG developed the framework and drafted the manuscript. PP conceived of the study and participated in the design of the framework. All authors read and approved the final manuscript.

## Supplementary Material

Additional file 1 **Assumptions, Abstractions, and Simplifications**. Click here for file

Additional file 2 **Calibration of the Model**. Click here for file

Additional file 3 **Cell-Cycle Model--Additional Results**. Description of additional model results. Click here for file

Additional file 4 **Alternative G2/M Trigger**. Investigation of an alternative trigger for G2/M. Click here for file

Additional file 5 **Powersim http://www.powersim.com Diagrams for the Proteins Included in the Cell-Cycle Model**. Lifecycle diagrams of the models used in the cell-cycle model. Click here for file

Additional file 6 **Executing the Cell-Cycle Model and Listing of Matlab Files Used in the Model**. Instructions for executing the example model and a listing of the Matlab source. Click here for file
