# Supplementary material for: A general framework for modeling growth and division of mammalian cells
Source: BMC Syst Biol. 2011 Jan 6;5:3. doi: 10.1186/1752-0509-5-3 (PMC3025838; doi:10.1186/1752-0509-5-3)
Supplement: Additional file 1 — Assumptions, Abstractions, and Simplifications. [file 1752-0509-5-3-S1.DOC]

**Additional File 1**

**Assumptions, Abstractions, and Simplifications**

As described here, assumptions, abstractions, and simplifications have been made in the example cell-cycle model. Some of these assumptions, abstractions, and simplifications have been made for brevity; some have been made out of necessity. Except for the use of ordinary differential equations (ODEs), if they are found to be limiting, most can be circumvented with additional modeling.

A number of assumptions are made in this work concerning use of ODEs to model a cell, including the following: (1) each molecule type is present in sufficient numbers such that a continuum approach allows a reasonable approximation; (2) a solution describing mean behavior is acceptable; (3) simple kinetic equations can describe reactions and movement of molecules between compartments; (4) the only compartmentalization important to the cell cycle is the nucleus and the cytoplasm; (5) all reactions are described. A well-mixed reaction chamber(s) need not be assumed, as long as the modeled reactions occur uniformly; e.g., the rate constant can also take into account transport within the reaction chamber.

The cell-cycle model tracks the number of molecules in the cell over time. Most numbers of molecules and reaction rates are unknown. (Most experimental results in cell biology deal qualitatively with the presence or absence of an effect, e.g., a Western blot, or relative amounts, e.g., microarray analysis, and the actual numbers of molecules and reaction rates are not measured.) In the model, the unknown numbers of molecules are estimated initially and the reaction rates are adjusted to give the appropriate behavior. When the actual numbers are finally known, they can be inserted, the rate constants can be modified, and substantially the same solution can be achieved.

In the model, the cell-cycle proteins represent different layers of abstraction, most are single proteins (e.g., cycE), but also included are protein complexes (RNA polymerase, RC, APC), classes of proteins (e.g., TF-grow represents c-myc, c-Jun, Notch, etc.), and pathways (e.g., Cdc14 represents the mitotic-exit-network pathway, with the assumption that sequential pathways—i.e., pathways that involve a linear progression of reactions, one after another—can be represented by a single member molecule).

Concentration of a given molecule in a chemical reaction is calculated as the product of the number of molecules in the cell and the size of the cell. The size of the cell is the ratio of the number of proteins at a given time and the number of proteins in the G0 cell (nominally 10^10^ proteins). Cell size is assumed to be approximately to that of a generic eukaryote with a radius of 10 μm (Alberts et al., 1994, p.139) and a volume of 4×10^-15^ m^3^.

Cell energy is simplified to be only by ATP hydrolysis. ATP is created from ADP only by respiration involving glucose.

In the base model, the building-block molecules are all treated generically. Nucleotides (NT) are not separated into A, T, C, G, or U, but rather are tracked en mass as a single molecular type. Similarly, amino acids (AA) are tracked en mass. Generic mRNA consist of 7000 NT before splicing and 1500 NT after splicing 5 exons. Generic tRNA consists of 80 NT. Generic snRNA consist of 2500 NT before splicing and 1250 NT after splicing 2 exons. snRNA constitute spliceosomes; the associated proteins are ignored. Generic rRNA are divided into 39% type I, 58% type II (and 0.1% type II that can transcribe snRNA), and 3% type III (Alberts et al., 1994, Table 8-3, p.370). All rRNA consist of 13,000 NT before splicing and 7000 NT after splicing 5 exons. Generic proteins consist of 400 AA. In the cell-cycle model, mRNA and proteins are described explicitly, but all consist of 1600 NT and 400 AA, respectively.

In the base model, transcription rate is dependent on the concentration of tRNA with NT attached, the concentration of active RNA polymerase of the appropriate type, and an elongation rate that is initially 30 NT/s (Alberts et al., 1994, p.368). The elongation rate is dependent on the concentration of NT in the cell, and it increases significantly during cell growth. The base-model transcription rate is used by the cell-cycle model, but modified by a loci-availability parameter and, where applicable, the amount of transcription factor. The affect of transcription factors is multiplicative.

DNA replication rate in the cell-cycle model is dependent on the concentrations of NT, DNA polymerase, active pre-replication complexes (RC), and an elongation rate of initially 50 NT/s (Alberts et al., 1994, p.251). The number of RC origins is 15,000, assuming DNA consists of 3×10^9^ base pairs (bp) with an average spacing of 2×10^5^ bp between origins (RC spacings are between 3×10^4^ bp and 3×10^5^ bp; Alberts et al., 1994, p.360). The model uses an artificial accumulator, RC_count, to control the number of RC that can be activated (licensed) and subsequently traversed by DNA polymerase. RC_count is reset to zero during cell division.

In the base model, the translation rate is dependent on the concentration of type II rRNA, the concentration of mRNA (and the number of those mRNA that do not have an rRNA attached within the first 100 NT), and an elongation rate, which is initially set to 20 AA/s (60 NT/s). The elongation rate is dependent on the concentration of AA bound to tRNA in the cell. There is no availability factor based on mRNA type; the assumption is that ribosomes and tRNA choose an mRNA at random.

In order to sustain transcription and replication rates in the model, both AA and NT enter the cell through the plasma membrane, via a concentration gradient, at the rate they are being used (polymerized). Internal synthesis of AA and NT is ignored.

The switch between G0 and G1 is activated by the presence of mitogen and adhesion factors. Mitogen represents various growth factors. Mitogen and adhesion factors are modeled as a 0 (absence) or 1 (presence). The presence of mitogen allows transcription of cycD and TF-grow. Mitogen and adhesion factors together allow transcription of cycA. Mitogen deactivates cycC/Cdk8, which allows activation of RNA polymerase. Mitogen also activates KPC, which ubiquitinates p27.

Ubiquitination is the ligation of ubiquitin proteins to target proteins, thereby marking them for degradation. In the model, ubiquitination is dependent on the availability of ATP, the concentration of the ubiquitinating protein (KPC, SCF with subunit, or APC with subunit), and the concentration of the target protein. It is assumed that upon ubiquitination, the target protein is immediately degraded, and any proteins complexed with the target protein are immediately available for other processes and any amino acids are available for translation.

Cell division is modeled as removal of (approximately) half of the cell. At the end of each time step, a proportion of the number of each molecule in each state in the cell is subtracted; when the cell is not dividing, this proportion (k_div) is 0; when the cell is dividing, k_div is set to 3×10^–3^. Cell division should occur when APC/Cdc20 ubiquitinates Securin. In the cell-cycle model, it is signaled when the rate that APC binds Cdh1 exceeds either (1) an arbitrary value of 100 or (2) 10 times the sum of the following rates: the rate that active APC/Cdh1 is ubiquinitated, and the rate that APC unbinds Cdh1 (the rate at which active APC/Cdh1 is lost is related to the amount of active APC/Cdh1 that is present). The cell division stops (k_div is set to 0) when the number of NT in DNA in the dividing cell falls to approximately 6×10^9^.

The most abstracted formulation in the example model is for the production of ATP. The rate of protons traversing the inner mitochondrial membrane is calculated as follows. The proton-pump rate (H_pump in Figure 2 in the main text) has a maximum value and an equilibrium value. The maximum value depends on the amount of sugar in the cell. The proton-pump rate attempts to approach the equilibrium value based on a specified rate constant. The equilibrium value is proportional to the product of two ratios: ratio of the concentration of protons within the inner mitochondrial membrane and the concentration between the inner and outer mitochondrial membranes, and the ratio of the ADP and ATP in the cell. Thus, H_pump = k_H_pump * (H_in_inner_mito_mems_conc/H_between_mito_mems) * (ADP_in_cell/ATP_in_cell). The assumption is that these ratios are preferred, and the model tries to keep them in balance under different energy-usage conditions. (The importance of ADP/ATP ratio is discussed by Reed and Cox, 1970, p.222.) The ATP-synthesis rate (ATP_synthase in Figure 2 in the main text) is similar; an equilibrium value is approached at a specified rate constant. In this case, the equilibrium value differs only with respect to the proton ratio—it is calculated as reciprocal of the proton ratio used for the proton-pump rate. Thus, ATP_synthase = k_ATP_synthase * (H_between_mito_mems/H_in_inner_mito_mems_conc) * (ADP_in_cell/ATP_in_cell).

The number of protons available to traverse the mitochondrial membranes was calculated as follows. Mitochondria are assumed to constitute 22% of the cell volume, or 8×10^-16^ m^3^. The volume within the inner mitochondrial membrane is assumed to be similar to that between the inner and outer mitochondrial membranes. The pH within the inner mitochondrial membrane is 8, or 10^-8^ M, and between the membranes the pH is 7, or 10^-7^ M (Alberts et al., 1994, p.695). Based on this information, the number of protons within the inner membrane is calculated to be 1.2×10^3^, and the number between membranes is 1.2×10^4^. Although these values are surprisingly small, what is most important in the formulation given above is the order-of-magnitude ratio between the numbers.

**References**

Alberts, B., D. Bray, J. Lewis, M. Raff, K. Roberts, and J.D. Watson, *Molecular Biology of the Cell*, 3^rd^ Edition, Garland Publishing, Inc., New York, 1994.

Brandhorst, B.P., and E.H. McConkey, Stability of nuclear RNA in mammalian cells, *J Mol Biol*, **85**, 451–463, 1974.

Reed, L.J., and D.J. Cox, Multienzyme complexes, in *The Enzymes, Structure and Control*, Vol. 1, 3^rd^ Edition, P.D. Boyer, Ed., Academic Press, New York, 1970.
