# Supplementary material for: A general framework for modeling growth and division of mammalian cells
Source: BMC Syst Biol. 2011 Jan 6;5:3. doi: 10.1186/1752-0509-5-3 (PMC3025838; doi:10.1186/1752-0509-5-3)
Supplement: Additional file 2 — Calibration of the Model. [file 1752-0509-5-3-S2.DOC]

**Additional file 2**

**Calibration of the Model**

Calibrating the cell-cycle model entails matching model behavior with observations, by adjusting model parameters. Model parameters consist of the initial numbers of each molecule in each state and the rate constants that govern the transitions from one state to another.

Major calibration parameters are shown in Table 2-2. At the time the model was developed, a limited amount of quantitative abundance data existed that can be directly compared with model values. Concentration data had to be taken from a variety of sources, including mammals other than humans. The lack of concentration data is not considered a significant defect in the model, because when better data are available, they can be easily inserted in the model and the applicable reaction rates can be scaled to give substantially the same results. Recently, large comparative cell-cycle databases have become available (e.g., Gauthier, 2008) that could be useful in further calibration.

Some of the quantitative abundance data used in the base model was based on mouse fibroblast data taken during interphase (Brandhost and McConkey, 1974). These data indicate that some molecules—e.g., rRNA and RNA polymerase—are in much greater abundance than is necessary during G0. The amount of RNA polymerase is the deciding factor for realizing a rapid transition from G0 to G1 (see Cell-Cycle Model Results). In the model, a pool of mostly inactive RNA polymerase is maintained in the cell during G0.

The number of DNA polymerases active during S phase is estimated from the literature to be 5000 (based on replicating 6×10^9^ NT at 50 NT/s in 8 hr); in the model this number is ~6000.

Most model calibration is based on derived or generalized-behavior data. For G0, it is calibrated to ~1E9 ATP and ~2E10 NT, and an energy usage of ~1E7 ATP/s. For the cell cycle, it is calibrated to double a cell’s ~1E10 proteins and ~6E9 NT in DNA in a time interval of ~1.3 days, which is relatively consistent with HeLa cells (modeled duration of the cell cycle and the phases of the cell cycle are somewhat longer than reported for HeLa cells by Bravo and Celis, 1980). The nominal elongation rate during both G0 and the cell cycle is reported to be 30 NT/s for transcription, 50 NT/s for DNA replication, and 20 AA/s for translation (Alberts et al., 1994, p.368, p.251, and p.108). For G0, the model is calibrated to these values. Elongation rates during the cell cycle in the model then become approximately 120 AA/s for protein, 50 NT/s for mRNA, and 90 NT/s for DNA. The cell-cycle rates could be reduced in the model by several means, including by increasing the numbers of ribosomes, RNA polymerase, and DNA polymerase, or by reducing the size of the cell.

Although not shown here, the example model is also calibrated to observations concerning lipid production and maintenance of osmotic balance (sodium-potassium pumps).

The most difficult aspects of calibration are as follows: (1) to match observed G0 and cell-cycle behavior using the same set of rate constants, (2) to proceed from G0 to cell division in a reasonable time interval only with the presence of mitogen and adhesion factors, and (3) to proceed from G2 to M, at approximately the same time in the cell cycle, when triggered by SCF/Btrc and also in the absence of SCF/Btrc. The cell-cycle model contains no growth monitoring, so it must be able to cycle sustainably at approximately the same timescale with approximately the same numbers of molecules over many divisions; that is, the cell cannot become increasingly smaller or increasingly larger over a number of divisions.

A number of studies have indicated that suppression or knockout of individual proteins involved in the cell cycle have minimal effect, including Cdk2, Cdc25C, and Btrc. For a discussion, see O’Farrell (2001), Aleem et al. (2004), and Watanabe et al. (2004). Possible explanations for these findings include that cycD/Cdk4-Cdk6 might substitute for cycE/Cdk2; cell division in embryonic cells might be regulated differently than in adult cells; the Cdc25 molecules might substitute for each other. Calibration of the example model for knockouts has been attempted in some cases (see Alternative G2/M Trigger, below). Further calibration of the model for knockouts is warranted, especially to help understand these effects.

Further calibration of the model is also warranted for robustness (see Achieving a Standard Model).

**References**

Alberts, B., D. Bray, J. Lewis, M. Raff, K. Roberts, and J.D. Watson, *Molecular Biology of the Cell*, 3^rd^ Edition, Garland Publishing, Inc., New York, 1994.

Aleem, E., C. Berthet, and P. Kaldis, Cdk2 as a master of S phase entry, *Cell Cycle*, **3**(1), 35–37, January 2004.

Brandhorst, B.P., and E.H. McConkey, Stability of nuclear RNA in mammalian cells, *J Mol Biol*, **85**, 451–463, 1974.

Bravo, R., and J.E. Celis, A search for differential polypeptide synthesis throughout the cell cycle of HeLa cells, *J Cell Biol*, **84**, 795–802, March 1980.

Gauthier, N.P., M.E. Larsen, R. Wernersson, U. de Lichtenberg, L.J. Jensen, S. Brunak, and T.S. Jensen, Cyclebase.org—a comprehensive multi-organism online database of cell-cycle experiments, *Nucleic Acids Res.*, **36**, D854–D859, January 2008. Epub 16 Oct 2007. doi:10.1093/nar/gkm729

O’Farrell, P.H., Triggering the all-or-nothing switch into mitosis, *TRENDS in Cell Biology*, **11**(12), 512–519, Dec 2001.

Watanabe, N., H. Arai, Y. Nishihara, M. Taniguchi, T. Hunter, and H. Osada, M-phase kinases induce phospho-dependent ubiquitination of somatic Wee1 by SCF{beta}-TrCP, *Proc Natl Acad Sci USA*, **101**(13), 4419–4424, 30 Mar 2004. Epub 22 Mar 2004.

Table 2-2. Calibration parameters.

|  | Quantity | Lit. Value | Reference^a^ | Model Value |
| --- | --- | --- | --- | --- |
| G0 | # proteins | 10^10^ | p.551 | 10^10^ |
|  | # amino acids (AA) unbound | 6×10^11^ | p.43 | 5.7×10^10^ |
|  | # nucleotides (NT) unbound | 0.4% by wt.  => 2×10^10^ | p.43 | 2×10^10^ |
|  | # ATP | 10^9^ | p.67 | 10^9^ |
|  | # ADP | 10^8^ | p.667 | 10^8^ |
|  | ATP usage | 10^7^ ATP/s | p.134 | 10^7^ ATP/s |
|  | Transcription elongation rate | 30 NT/s | p.368 | 30 NT/s |
|  | Translation elongation rate | 20 AA/s | p.108 | 20 AA/s |
|  | DNA replication elongation rate | 50 NT/s | p.359 | 50 NT/s |
|  | Na^+^-K^+^ ATPase energy (fraction of total) | ~33% | p.514 | 32% |
|  | Translation energy fraction | Most | p.234 | 51% |
| Cell  Cycle^b^ | Cell-cycle duration (HeLa) | 25 hr | (c) | 33 hr |
|  | G1 duration | 12 hr | (c) | 15 hr |
|  | S duration | 8 hr | (c) | 7 hr |
|  | G2 | 4 hr | (c) | 7 hr |
|  | M | 4 hr | (c) | 4 hr |
|  | # nucleotides (NT) in RNA | 5×10^10^ | (d) | 1.6×10^10^ (ave) |
|  | # rRNA  (not including pre-rRNA) | 3.6×10^6^; 10^7^ | p.369, 378 | 1.5×10^6^ |
|  | # tRNA | 9×10^7^ | p.370 | 2.8×10^7^ |
|  | # mRNA  (not including hnRNA) | 3.6×10^5^ | p.369 | 2.7×10^5^ |
|  | # snRNA | 1 or 2 per transcript | p.373 | 9.7×10^3^ (~0.25 per transcript) |
|  | # RNA polymerases | 2×10^4^-4×10^4^ | p.367 | 3×10^4^ (ave) |
|  | hnRNA fraction | 7% | (d) | 8% (ave) |
|  | mRNA (cytosol) fraction | 3% | (d) | 3% (ave) |
|  | rRNA precursors fraction | 4% | (d) | 8% (ave) |
|  | rRNA fraction | 71% | (d) | 67% (ave) |
|  | tRNA fraction | 15% | (d) | 14% (ave) |
|  | RNA fraction in nucleus | 14% | (d) | 16% (ave) |
| Misc. | Same reaction rates in G0 and during the cell cycle | See text | | |
|  | Viability of knockouts | See text | | |
|  | G0/cell-cycle energy ratio | 20% | p.462 | ~7% |
|  | G0 translation energy fraction | Most | p.234 | 51% |

^a^ Alberts et al. (1994)

^b^ continuous cycling

^c^ Bravo and Celis, 1980—note that the reported cycle duration does not equal the sum of the durations of the phases

^d^ values from murine fibroblast culture from Brandhost and McConkey (1974)

^e^ dependent on the amount of energy used to transport metabolites and nutrients across the plasma membrane
