# Supplementary material for: A general framework for modeling growth and division of mammalian cells
Source: BMC Syst Biol. 2011 Jan 6;5:3. doi: 10.1186/1752-0509-5-3 (PMC3025838; doi:10.1186/1752-0509-5-3)
Supplement: Additional file 3 — Cell-Cycle Model--Additional Results. Description of additional model results. [file 1752-0509-5-3-S3.DOC]

**Additional file 3**

**Cell-Cycle Model—Additional Results**

In this section, the cell-cycle model is described through its results. Of most interest are the interaction between molecules and the timing of the various reactions—the model indicates what can occur for the entire cell cycle to be self-consistent.

The results presented here start with a cell in G0. An example would be a quiescent fibroblast stem cell. The cell cycle consists of doubling the cell’s contents, followed by the division of the cell in two. G1 is the initial growth phase; S phase is when DNA is replicated; G2 is the later growth phase; M is the division phase. In the results presented here, G1 begins at 2×10^4^ seconds (~0.25 days) with the imposition of mitogen and adhesion factors. Although no steady-state results are displayed here, the G0 values can be determined from the early time values in the plots shown below.

*Cell cycle—G1 phase*

G1 is the first growth phase of the cell cycle; the initial G1 lasts 24 hours, subsequent G1 phases last 15 hours. G1 initially begins with the appearance of mitogen or subsequently with the culmination of M phase.

The appearance of mitogen at 0.25 days in the example model has several effects: (1) mitogen deactivates cycC/Cdk8, thus releasing inhibition of RNA polymerase; (2) mitogen activates KPC (not shown), which ubiquitinates p27 (Figure 3-1); and (3) mitogen allows transcription of several proteins, including cycD (Figure 3-2). In Figures 3-1 and 3-2, and most of the following figures, the numbers of molecules in the cell given in the Y-axis are those estimated by the cell model; cellular concentration data cannot be found in the literature for most proteins.

Protein p27 is an inhibitor of cyclin-dependent kinases (Cdk). One function of p27 is to keep the cell in G0. Another function of p27 is to time the duration of the initial G1 phase when the cell exits G0. As shown in Figure 3-1, p27 is abundant during G0. When the cell cycle begins, mitogen promotes the accumulation of cyclins, which complex with their appropriate kinases—cycD with Cdk4 or Cdk6 and cycE with Cdk2. These cyc/Cdk complexes are bound and inhibited by p27 (Figures 3-2 and 3-4). Mitogen also activates KPC, however, which ubiquitinates p27. In the model, KPC only serves to reduce the number of free p27 in the nucleus. Thus, through G1 the number of p27 decreases and the number of cyc/Cdk complexes increases until p27 can no longer inhibit all of the cyc/Cdk. At this point, active cyc/Cdk (primarily cycD/Cdk4or6 and cycE/Cdk2) cause p27 to be released from inactive cyc/Cdk, as shown by the green spike in the figure at 1.4 days. The released p27 are rapidly ubiquitinated by KPC and SCF(Skp2) (see Figure 3-7). The timing of complete ubiquitination of p27 and the simultaneous inactivation of APC(Cdh1) (see Figure 3-6) determines the duration of the G1. The model suggests that p27 ubiquitination is more important for the timing of the initial G1/S, while APC(Cdh1) inactivation is more important for timing of subsequent iterations of G1/S.


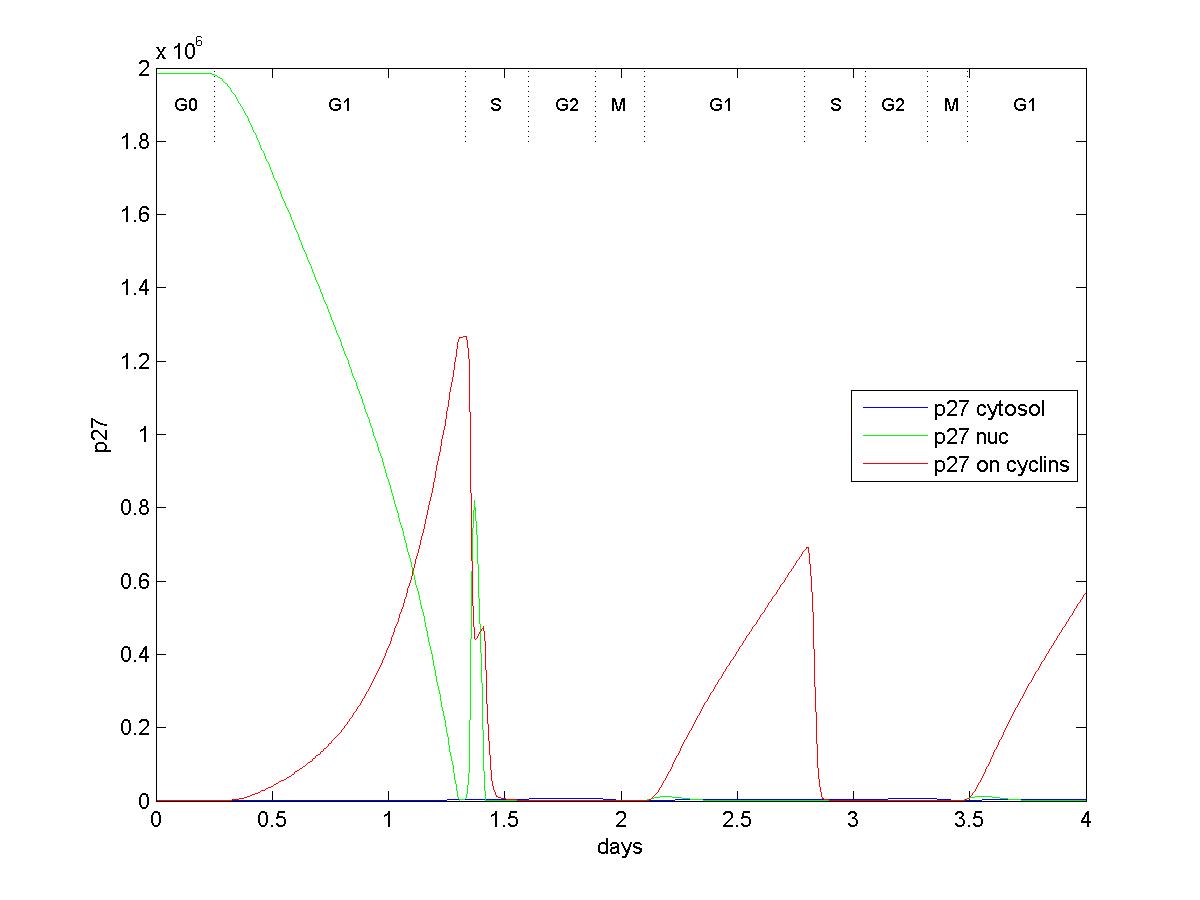
 Figure 3-1. Numbers of p27 over two cell divisions. p27 is constitutively expressed. With the introduction of mitogen, p27 is initially reduced by KPC (not shown); some p27 escapes KPC by binding and, for the most part, inhibiting cycD and cycE as they are translated. Upon entry into S, active cycE/Cdk2 and cycD/Cdk4or6 release p27, which is ubiquitinated by KPC and SCF(Skp2).

*Modeling of p27 (aka CDKN1B, Kip1, Men4, CDKN4, Men1B, or p27(kip1)) is based on Ang and Harper (2004), Vodemaier (2004), Malumbres and Barbacid (2001), Nakayama and Nakayama (2005), Novak, Sible, and Tyson (2002), Bollen and Beullens (2002), and Sherr (1996). Modeling KPC (Kip1 ubiquitination-promoting complex) is based on Kamura et al. (2004).*


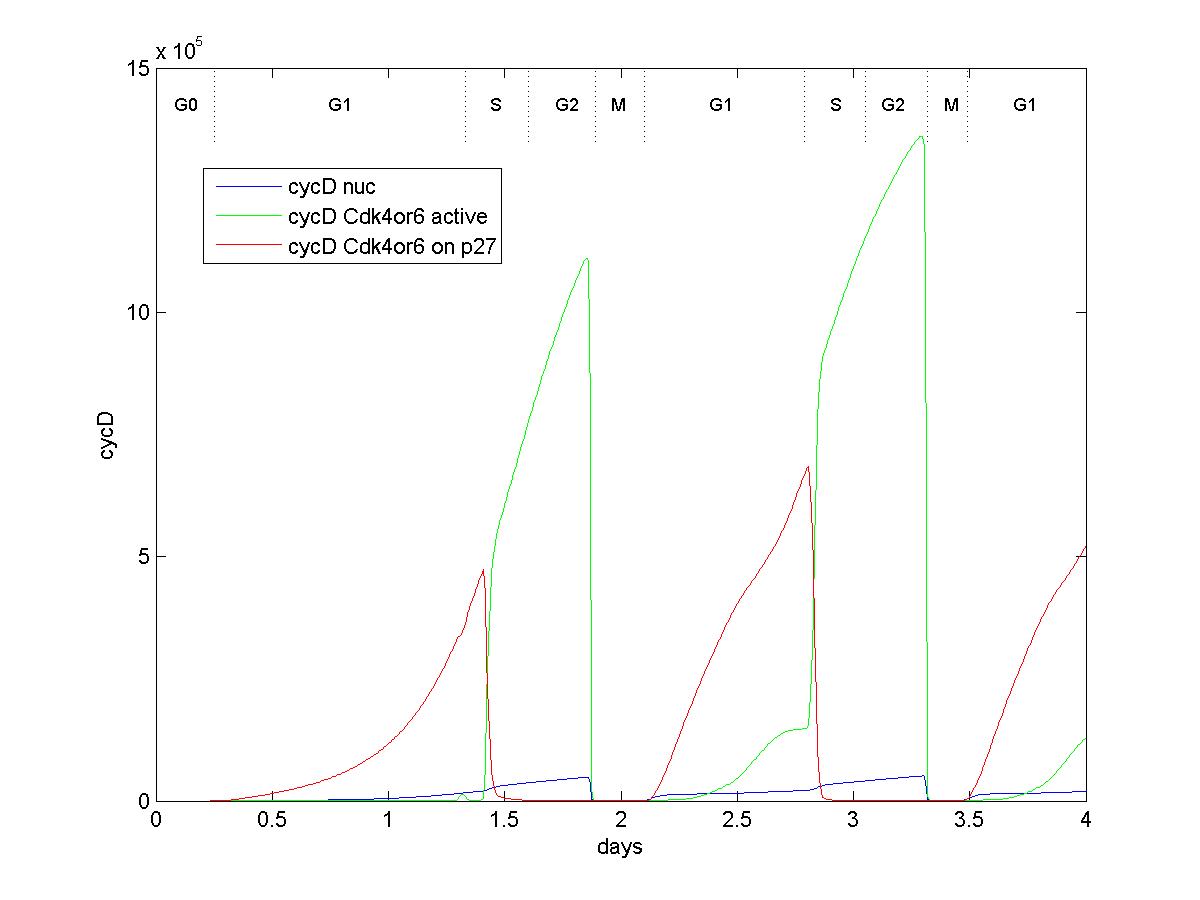
Figure 3-2. Numbers of cycD over two cell divisions. cycD is induced by mitogen and enhanced by B-Myb, then binds Cdk4or6 (a term used here for Cdk4 or Cdk6). cycD/Cdk4or6 is mostly held inactive by p27, although it can still phosphorylate Rb. When p27 is removed, cycD/Cdk4or6 becomes fully active and suppresses APC(Cdh1). Not enough p27 is available to completely inactivate cycD/Cdk4or6 during the second and subsequent cell cycles, resulting in shorter G1 phases.

Transcription of cycD (Figure 3-2) is induced by mitogen and B-myb (not shown). Newly translated cycD binds constitutive Cdk4 or Cdk6, and cycD/Cdk4or6 is immediately bound by p27. Near the end of G1, p27 is ubiquitinated, and cycD/Cdk4or6 becomes fully active. During S and G2, SCF/Skp2 ubiquitinates cycD, although poorly; cycD/Cdk4or6 mostly remains active through S and G2. During M phase, cycD is completely ubiquitinated by APC(Cdc20).

One function of cycD in the cell cycle is to phosphorylate Rb (not shown) and thereby activate the E2F family of transcription factors (Figure 3-3). In the model, both p27-bound cycD/Cdk4or6 and fully active cycD/Cdk4or6 can cause the release of Rb from E2F. Another function of cycD is to phosphorylate and deactivate Cdh1 (Figure 3-6) throughout S and G2.

*Modeling cycD (aka cyclin D, growth-factor sensor, CCND1, BCL1, D11S287E, PRAD1, or U21B31) is based on Malumbres and Barbacid (2001), Novak, Sible, and Tyson (2002), and Sherr (1996).*


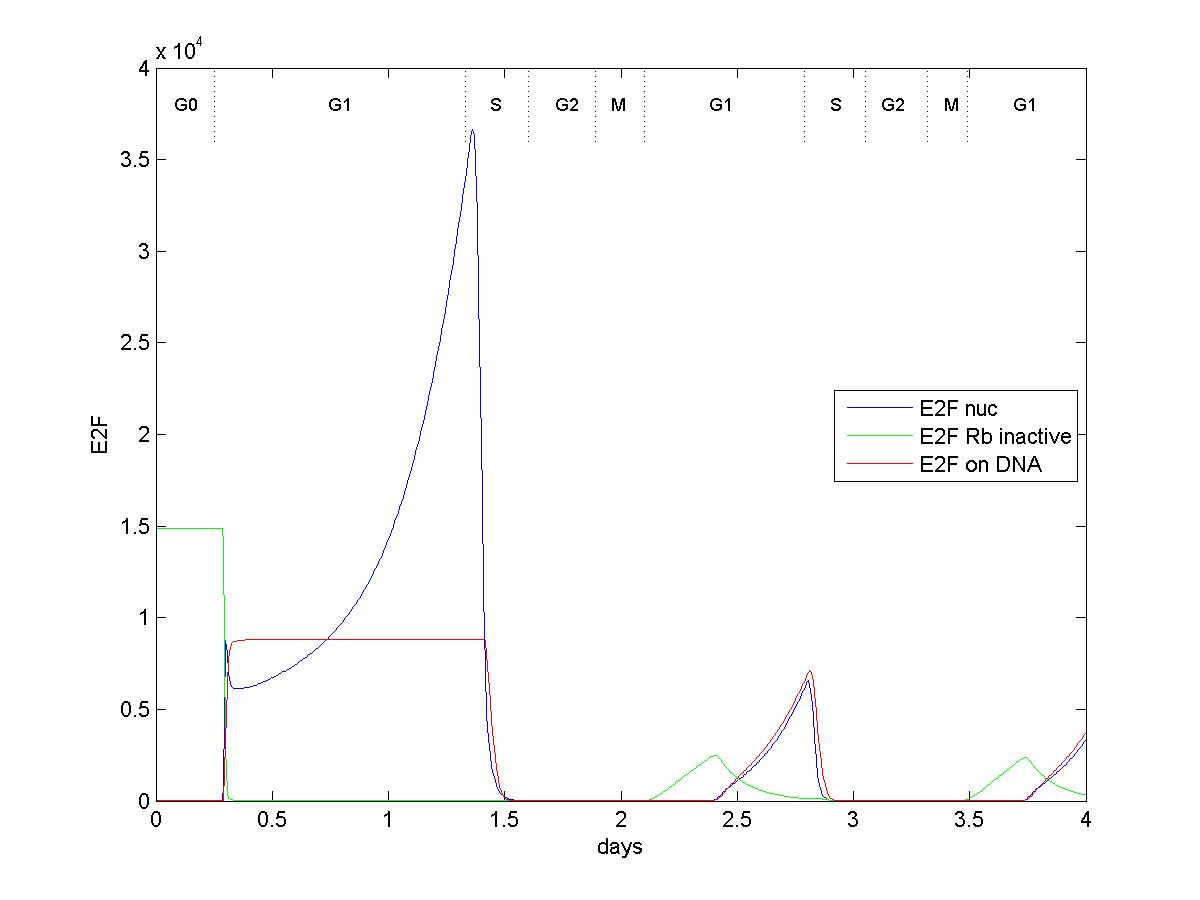
 Figure 3-3. Numbers of the E2F family of transcription factors over two cell divisions. E2F is released from Rb by cycD/Cdk4or6 early in G1, allowing many E2F to bind DNA. During S phase, E2F is released from DNA because of weak binding and phosphorylation by active cyc/Cdk; then E2F is ubiquitinated by SCF(Skp2).

E2F (representing the E2F family of proteins) is a transcription factor responsible for inducing many cell-cycle and cell-growth proteins. As shown in Figure 3-3, E2F is present during G0, but is bound and inhibited by Rb (not shown). cycD/Cdk4or6 releases Rb. E2F then binds DNA for much of G1, where it induces cycE, cycA , Fbw7, Emi1, etc., and itself. E2F is released from DNA at the beginning of S phase, when it is phosphorylated by cycD/Cdk4or6 and cycA/Cdk2. (cycE/Cdk2 cannot be responsible for releasing E2F from DNA because cycE/Cdk2 appears too soon.) E2F also tends to spontaneously detach because it is weakly bound to DNA. When E2F is free in the nucleus, it is ubiquitinated by SCF/Skp2 (Figure 3-7). Three other major transcription factors are contained in the model: B-Myb, NF-Y, and a generic TF-grow that is meant to represent several transcription factors (molecules not shown).

*Modeling E2F (representing the E2F family) is based on Sherr (1996), Nakayama and Nakayama (2005), and Malumbres and Barbacid (2001); modeling Rb (aka retinoblastoma 1, Rb1, RB, or OSRC) is based on Sherr (1996) and Novak, Sible, and Tyson (2002). And although not shown, modeling B-Myb (aka Mybl2) is based on Nakayama and Nakayama (2005), Fung and Poon (2005), Sala et al. (1996), Sala et al. (1997), Robinson (1996), Zhu (2004), and Cicchillitti et al. (2004); modeling NF-Y (aka Cp1, or CBF) composed of NF-YA, NF-YB, and NF-YC, all required for DNA binding) is based on Yun et al. (2003), Chae et al. (2004), Fung and Poon (2005), Eggen et al. (2001), Gurtner et al. (2003), and Zhao et al. (2005); modeling TF-grow (meant to represent c-myc, c-Jun, Jun, Ap1, Notch1, Notch4, etc.) is based on Nakayama and Nakayama (2005), and Cicchillitti et al. (2004).*


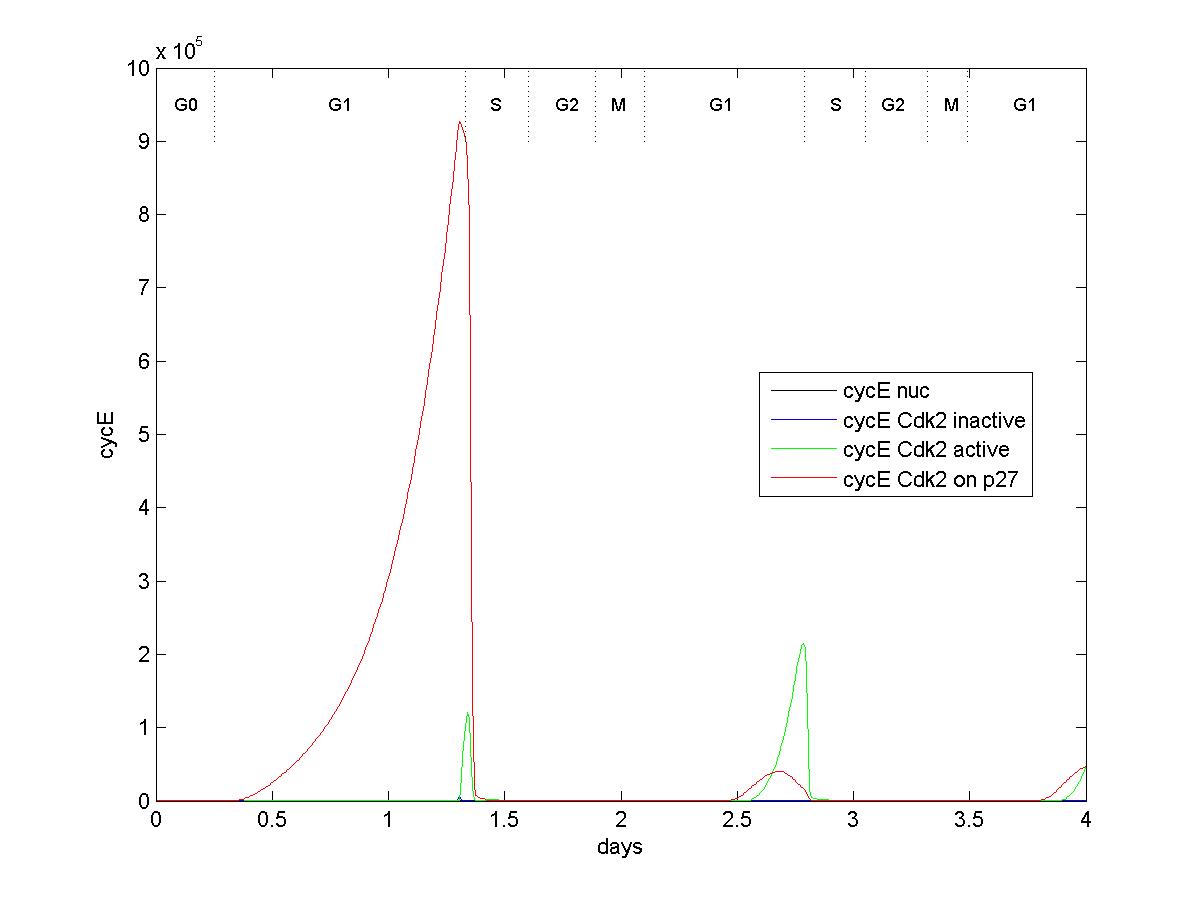
 Figure 3-4. Numbers of cycE over two cell divisions. E2F induces cycE. Cdk2 is constitutive and is also enhanced by E2F. Nuclear cycE binds Cdk2. p27 binds and inactivates cycE/Cdk2. Large numbers of inactive cycE/Cdk2 accumulate during the long first G1 phase. cycE/Cdk2 are activated in an irreversible cascade with Cdc25A; almost immediately cycE/Cdk2 is ubiquitinated by SCF(Fbw7).

The function of cycE is to bind and activate Cdk2 and allow this kinase to activate the DNA replication complexes (RC; see Figure 3-8) and thus initiate S phase. cycE (Figure 3-4) is induced by E2F and binds Cdk2 (not shown), which is (mostly) constitutively present. cycE/Cdk2 is immediately bound by p27 and held inactive until p27 is released. Because of the prolonged duration of the initial G1, large numbers of cycE/Cdk2 accumulate; but, in the model, fewer cycE/Cdk2 activate during the initial G1 because of the large number of p27. As p27 is released, cycE/Cdk2 phosphorylates and activates Cdc25A, and in turn is dephosphorylated and activated by Cdc25A (Figure 3-5) in an irreversible cascade. The cascade starts because of a low level of spontaneous dephosphorylation of cycE/Cdk2. After the cycE/Cdk2-Cdc25A cascade, cycE is abruptly ubiquitinated by SCF(Fbw7) (Figure 3-7). Degradation of cycE frees Cdk2 to bind cycA. Cdc25A then activates cycA/Cdk2 (not shown). In the model, cycE has a greater affinity for Cdk2 than does cycA, although in all other aspects, cycA can functionally substitute for cycE.


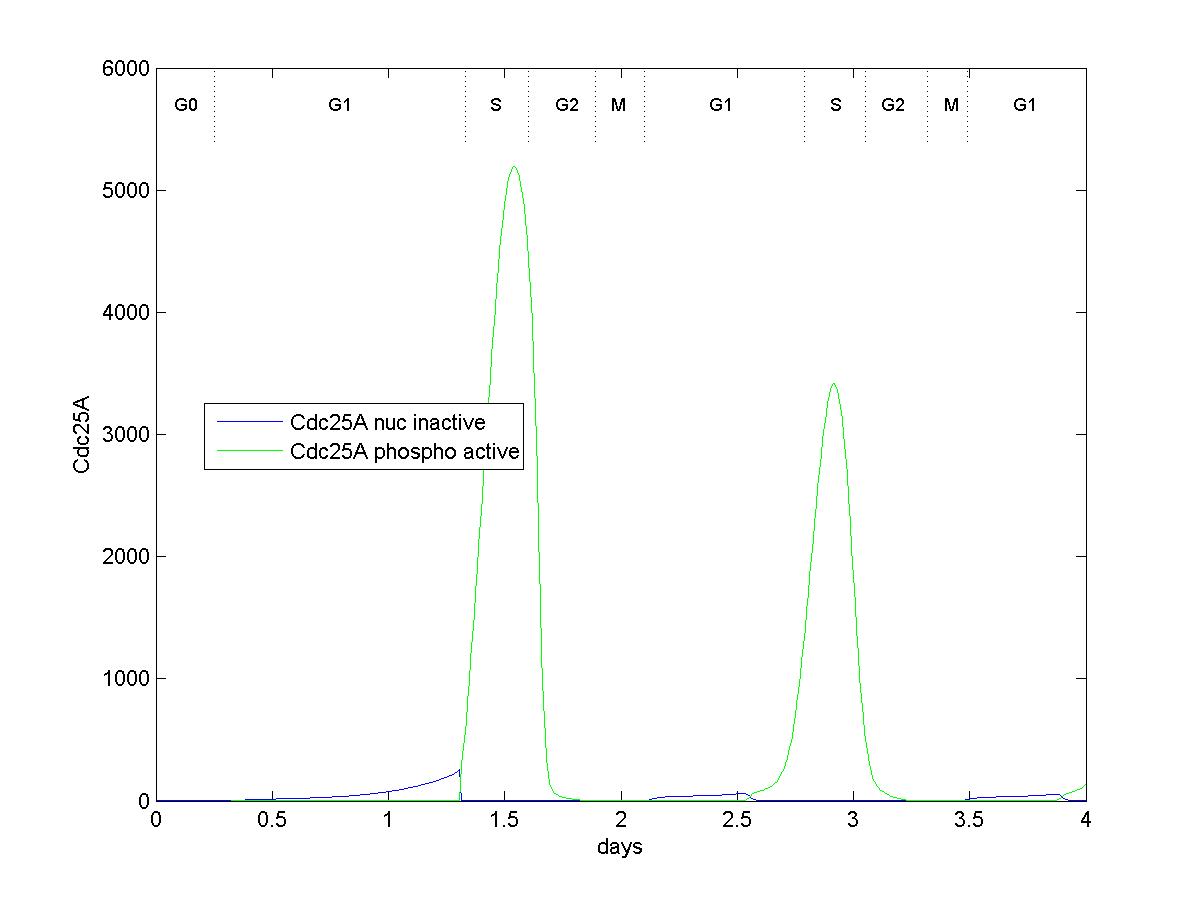
 Figure 3-5. Numbers of Cdc25A over two cell divisions. Cdc25A accumulates rapidly in S phase after APC(Cdh1) is inhibited. Cdc25A dephosphorylates cycE/Cdk2 and cycA/Cdk2, then is ubiquitinated by SCF(Btrc).

*Modeling cycE (cyclin E, CCNE1) is based on Bartek and Lukas (2001) and Malumbres and Barbacid (2001); modeling Cdc25A is based on Novak, Sible, and Tyson (2002), Donzelli and Draetta (2003), Bollen and Beullens (2002), Pagano (2006), Nakayama and Nakayama (2005), Kristjansdottir and Rudolf (2004), and Vazquez-Novelle et al. (2005). Although not shown, modeling Cdk2 (cyclin-dependent kinase 2, p33(Cdk2), S. cerevisiae cdc28, S. pombe cdc2) is based on Sherr (1996), Novak, Sible, and Tyson (2002), Donzelli and Draetta (2003), and Malumbres and Barbacid (2001).*


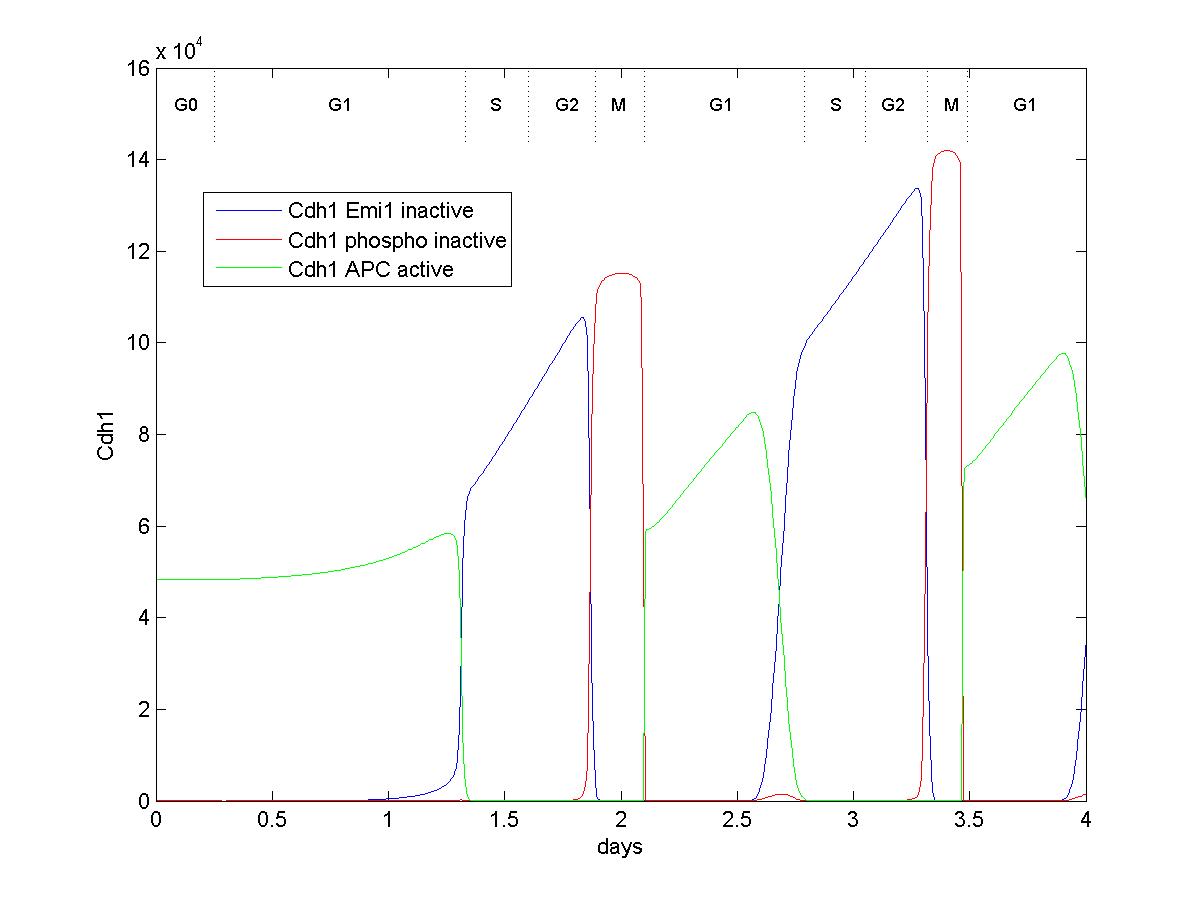
 Figure 3-6. Numbers of Cdh1 over two cell divisions. Cdh1 is active when bound to APC during G0 and G1, when it ubiquitinates a number of proteins involved in cell-cycle progression. Cdh1 is phosphorylated and released from APC by cyc/Cdk (primarily cycD/Cdk4or6), at which time it binds Emi1. When Emi1 is ubiquitinated by SCF(Btrc), Cdh1 becomes unattached, because during M phase APC preferentially binds Cdc20. At the end of M phase, with the absence of active cyc/Cdk and Cdc20, Cdc14 dephosphorylates Cdh1, which allows Cdh1 to bind APC again, allowing G1 to progress again.

The anaphase-promoting complex (APC) is an ubiquitin ligase that is activated by subunit Cdh1 or subunit Cdc20. The function of APC(Cdh1) (Figure 3-6) is to maintain G0 and G1 by ubiquitinating, and thus marking for degradation, many of the proteins necessary for cell-cycle progression, including Cdc25A, cycA, cycB, and SCF.

A timing problem for the initiation of S phase occurs because cycE, its activator Cdc25A, and its ubiquitinator Fbw7 are all induced at the same time in G1 by the E2F transcription factors. The model explains why the activities of these molecules occur at different times, as follows. APC(Cdh1) ubiquitinates both Cdc25A and SCF (which is required by Fbw7) before the cycE/Cdk2-Cdc25A cascade; further, SCF is ubiquitinated more effectively than Cdc25A. Thus, as the concentration of active APC(Cdh1) decreases during G1 with the accumulation of cyc/Cdk, first Cdc25A appears and activates cycE/Cdk2, then SCF appears and binds Fbw7 and ubiquitinates cycE. Ubiquitination of cycE subsequently frees Cdk2 for binding to cycA during S phase.

APC(Cdh1) concentration decreases during G1 because phosphorylation by cycD/Cdk4or6, cycE/Cdk2, and cycACdk2 causes Cdh1 to unbind from APC. Cdh1 is then bound and inhibited by Emi1 (not shown). Emi1 is another protein induced by E2F. As Cdh1 is inhibited, cycA and cycB concentrations increase. cycA/Cdk2 (complex not shown) can take the place of cycE/Cdk2 and can initiate S phase, but the major role for cycA/Cdk2, at least in the model, is to keep Cdh1 deactivated until the end of the cell cycle.

*Modeling APC (anaphase-promoting complex) is based on Novak, Sible, and Tyson (2002), Ang and Harper (2004), and Pagano (2006); modeling Cdh1 (aka Hct1 or Fizzy-related) is based on Ang and Harper (2004), Pagano (2006), Novak, Sible, and Tyson (2002), Bollen and Beullens (2002), and Bennbenek and Yu (2001).*


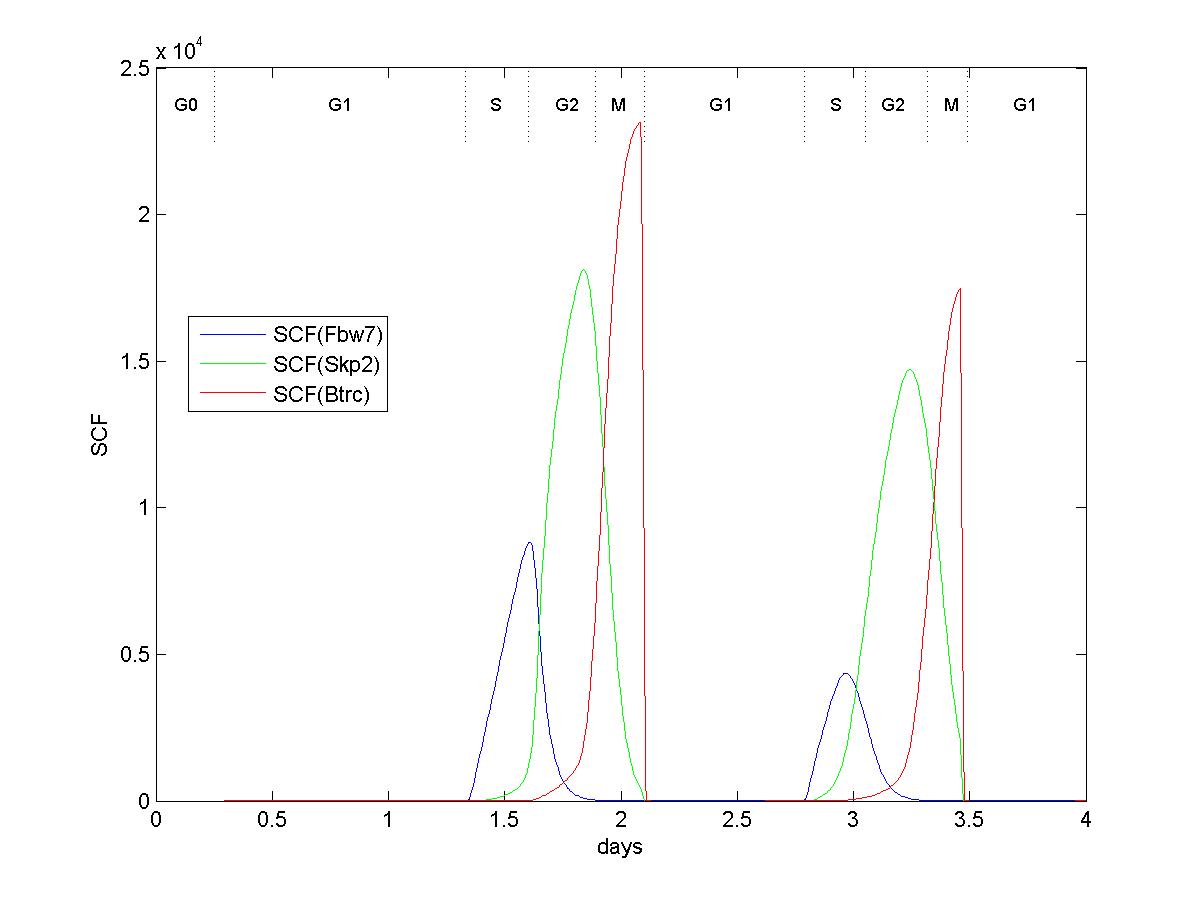
 Figure 3-7. Numbers of SCF bound to Fbw7, Skp2, and Btrc subunits over two cell divisions. SCF and Skp2 are constitutively transcribed, but ubiquitinated by APC(Cdh1), thus limiting their expression to S, G2, and M. Fbw7 and Btrc are induced by E2F. Thus, all are present at the same time. First, SCF preferentially binds Fbw7 and ubiquitinates Fbw7 substrates, at which point Fbw7 is autoubiquitinated. Then SCF preferentially binds Skp2 and ubiquitinates Skp2 substrates (in particular, RC) at which point Skp2 is autoubiquitinated. Finally, SCF binds Btrc, concluding the sequence.

The function of the ubiquitin ligase SCF (an acronym for Skp1-Cullen-F-box) is to ubiquitinate, with the aid of the appropriate subunit, several molecules that progress the cell cycle, which, if left intact, could possibly cause processes to continue or repeat with detrimental consequences. Interestingly, SCF must bind, in turn, subunit Fbw7, then subunit Skp2, then subunit Btrc (Figure 3-7). The three subunits are present at the same time beginning in G1. This timing problem is solved in the model by SCF having greater affinity for Fbw7 than Skp2, and having greater affinity for Skp2 than Btrc. All of these SCF complexes autoubiquitinate when no substrate is present (Vodermaier, 2004). So, SCF preferentially binds Fbw7, which autoubiquitinates when cycE is gone; then SCF preferentially binds Skp2, which autoubiquitinates when p27, cycE, E2F, and RC are gone; then SCF binds Btrc. (SCF(Skp2) substrate cycD does not suppress autoubiquitination in the model, given evidence for cycD abundance through most of the cell cycle.) In the model, only the subunits autoubiquitinate, leaving SCF to immediately bind another subunit (Zhou and Howley, 1998). Interestingly, this process provides a timing method for the duration of S phase and, to a certain extent, G2 phase (see Cell cycle—G2 phase, below).

*Modeling SCF (Skp- Cullen-F-box) is based on Ang and Harper (2004); modeling Fbw7 (aka F-box and WD repeat domain containing 7, Fbxw7, Fbx30, Fbxw6, Cdc4, Ago, Sel10, or DKFZp686F23254) is based on Bartek and Lukas (2001), Sim et al. (2004), Nakayama and Nakayama (2005), Vodermaier (2004), and Pagano (2006); modeling Skp2 (aka S-phase kinase-associated protein 2, p45, Fbl1, Fbxl1, Flb1, or Mgc1366) is based on Ang and Harper (2004), Vodermaier (2004), Nakayama and Nakayama (2005), Amati (2004), and Pagano (2006); modeling Btrc (aka beta-transducin repeat containing, beta-TrCP, bTrCP, bTrCP1, Rp11-529I10.2, Fbw1A, Fbxw1A, Fwd1, Mgc4643) is based on Vodermaier (2004), Watanabe et al. (2004), and Nakayama and Nakayama (2005).*

*Cell cycle—S phase*

S phase is when DNA replication occurs. S phase begins with the cycE/Cdk2-Cdc25A cascade and lasts approximately 8 hours. E2F induces proteins such as hORC1 and hCdc6 that contribute to DNA replication complexes (RC). The function of the RC is to mark 15,000 binding locations on DNA and, when activated by cycE/Cdk2 or cycA/Cdk2, allow binding of DNA polymerases to these locations and subsequent DNA replication (Alberts et al., 1994, p.356ff). Figure 3-8 indicates how RC are first bound to DNA, then licensed (activated by Cdk2), then traversed by DNA polymerase. Also shown is an artificial modeling construct, RC-count, which ensures that only 15,000 RC locations on DNA are bound and traversed. The model includes continual RC binding and unbinding from DNA, as well as continual decay of the RC. DNA polymerases only attach and traverse licensed RC. After DNA polymerases bind the RC, the RC become delicensed. Delicensed RC are released from the DNA and ubiquitinated by SCF(Skp2) (Mendez et al., 2002; Li et al., 2003).


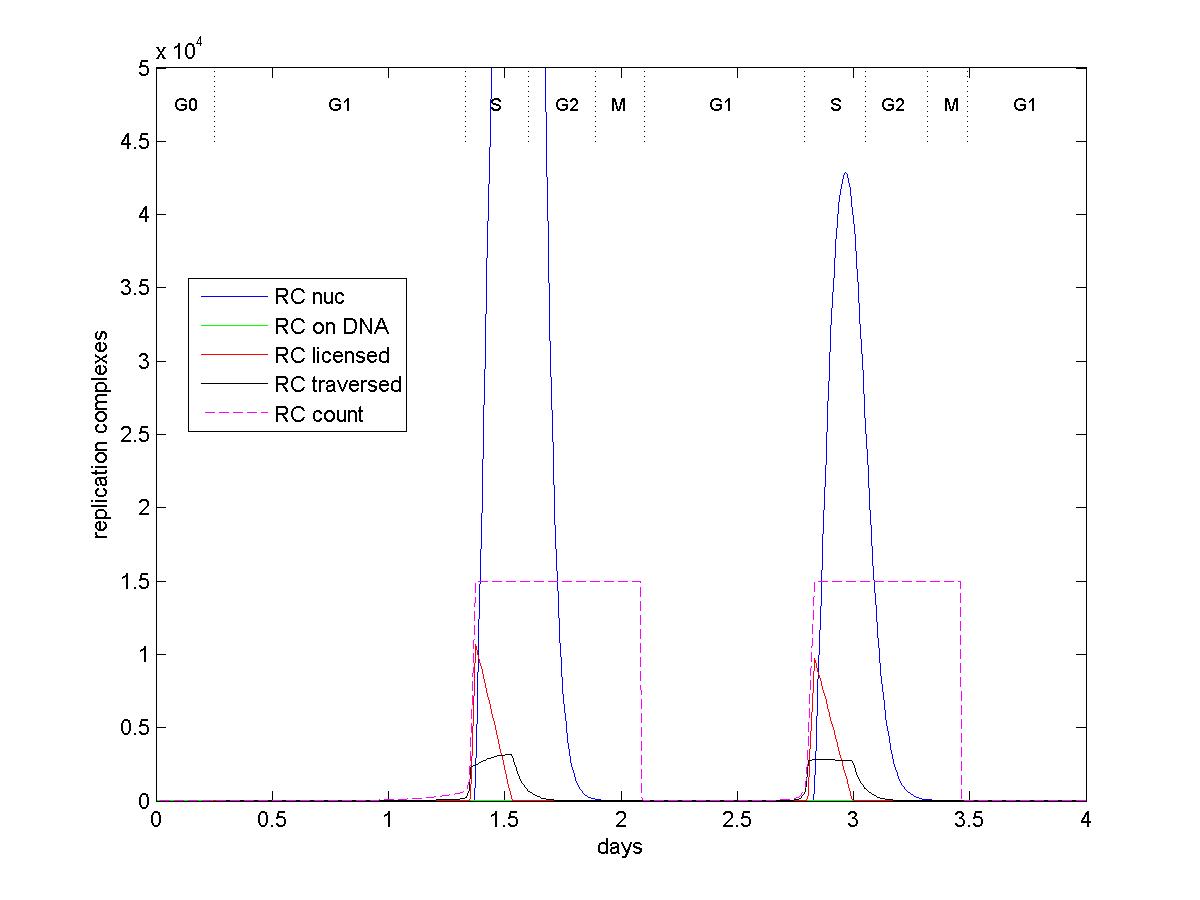
 Figure 3-8. Numbers of replication complex (RC) over two cell divisions. RC is induced by E2F, accumulates in the nucleus, and binds DNA at 15,000 replication origins. RC is licensed by cyc/Cdk, primarily cycD/Cdk4or6 and cycE/Cdk2, after which RC is bound and traversed by DNA polymerase during DNA replication. Once traversed, the RC is released from the DNA and ubiquitinated, primarily by SCF(Skp2). The figure also shows a modeling construct, RC-count, which is used to set and maintain the 15,000-replication-orgin limit.


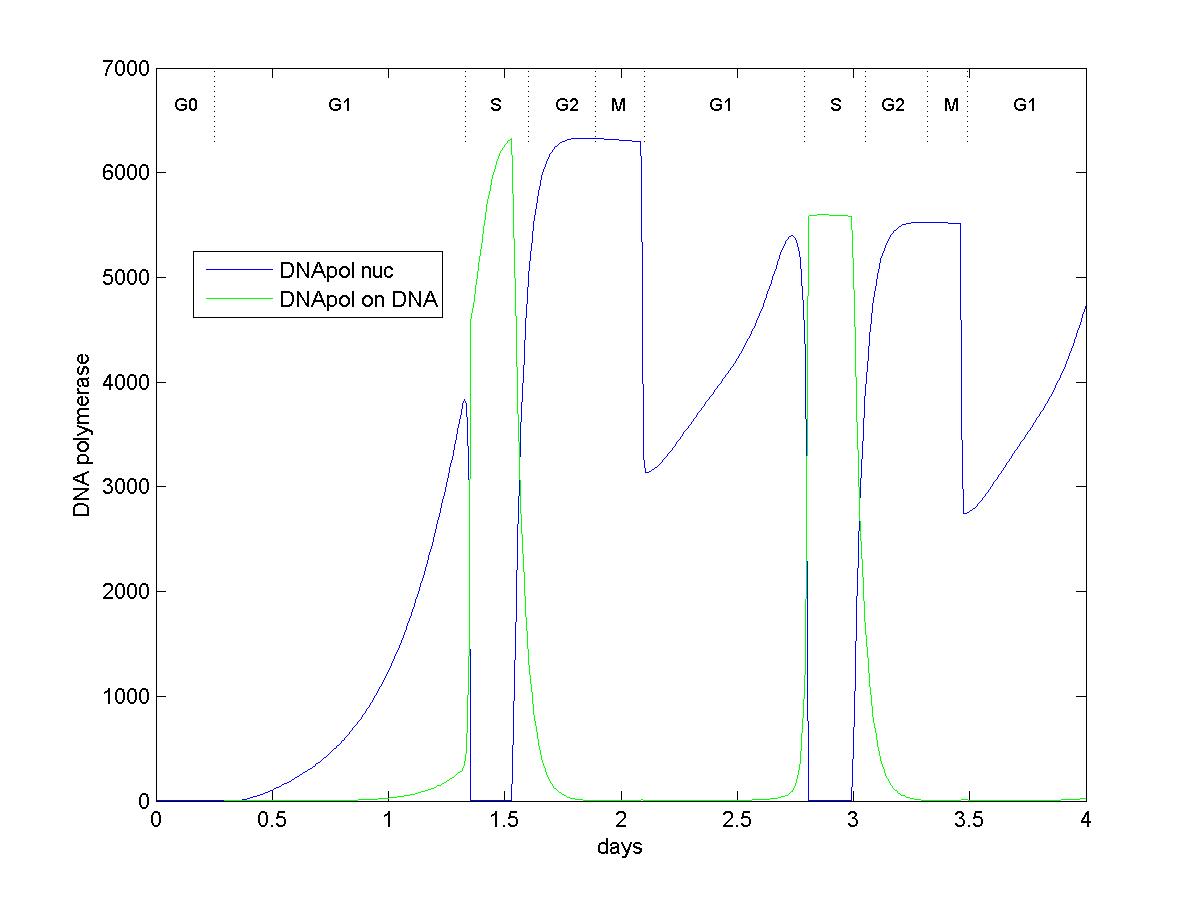
 Figure 3-9. Numbers of DNA polymerase over two cell divisions. When cyc/Cdk licenses an RC, a DNA polymerase binds and traverses a section of DNA that is 200,000 base pairs in length, creating an additional 200,000 base pairs of DNA, and delicenses the RC, thereby releasing the RC from the DNA. The DNA polymerase then detaches and reattaches at another licensed RC. This process is repeated until all RC are delicensed. The DNA polymerase then remains free in the nucleus until licensed RC are again available during the next S phase. The total number of DNA polymerase is halved at cell division. An artificial saturation limit for DNA polymerases is included in the model (see text)

As shown in Figure 3-9, DNA replication involves ~6000 DNA polymerases. DNA-polymerase transcription is induced and enhanced by E2F, B-Myb, and NF-Y. In order to have a sufficient accumulation rate for the initial cycle, the duration of the initial G1 phase is prolonged. The necessary transcription and translation rates lead to too many DNA polymerase over time. An assumption is made that there is a saturation value that limits the accumulation of DNA polymerase. As the actual mechanism to maintain a saturation limit is unknown, an artificial limit is imposed. In the model, all basic cell processes, including transcription and translation, continue uninterrupted during S phase.

*Modeling RC (aka preRC or the replication complex, including ORC1-5, Cdc6=Cdc18, Mcm2-7, etc.) is based on Alberts et al. (1994) p.360, Yam et al. (2002), Vodermaier (2004), Nasmyth (1996), Mendez et al. (2002), and Quintana and Dutta (1999). Modeling DNA polymerase is based on Alberts et al. (1994) pp.250, 337, and 361, Chae et al. (2004), and Sherr (1996).*

*Cell cycle—G2 phase*

G2 is a continuation of cell growth after S phase. G2 nominally begins after DNA replication subsides; however, modeling suggests that DNA replication does not end abruptly, but rather tails off. Also, growth continues throughout S phase. Therefore, the initiation of G2 is inexact. The initial G2 phase lasts 12 hours; subsequent G2 phases last 10 hours. The termination of G2 occurs abruptly, with the cycB/Cdk1-Cdc25C cascade. Although the amount of DNA is double during G2, the transcription rate does not increase—transcription rate is dependent on the number of RNA polymerase.

A function of cycA (Figure 3-10) and cycB (Figure 3-11) is to bind and activate kinase Cdk1; the main function of Cdk1 is to ultimately activate APC(Cdc20) and initiate cell division. (cycA also binds and activates Cdk2 kinase.) During G2, both cycA and cycB accumulate and bind Cdk1 in the cytoplasm. Initially after binding, both cycA/Cdk1 and cycB/Cdk1 are inactive, suppressed by Wee1 (Figure 3-13).


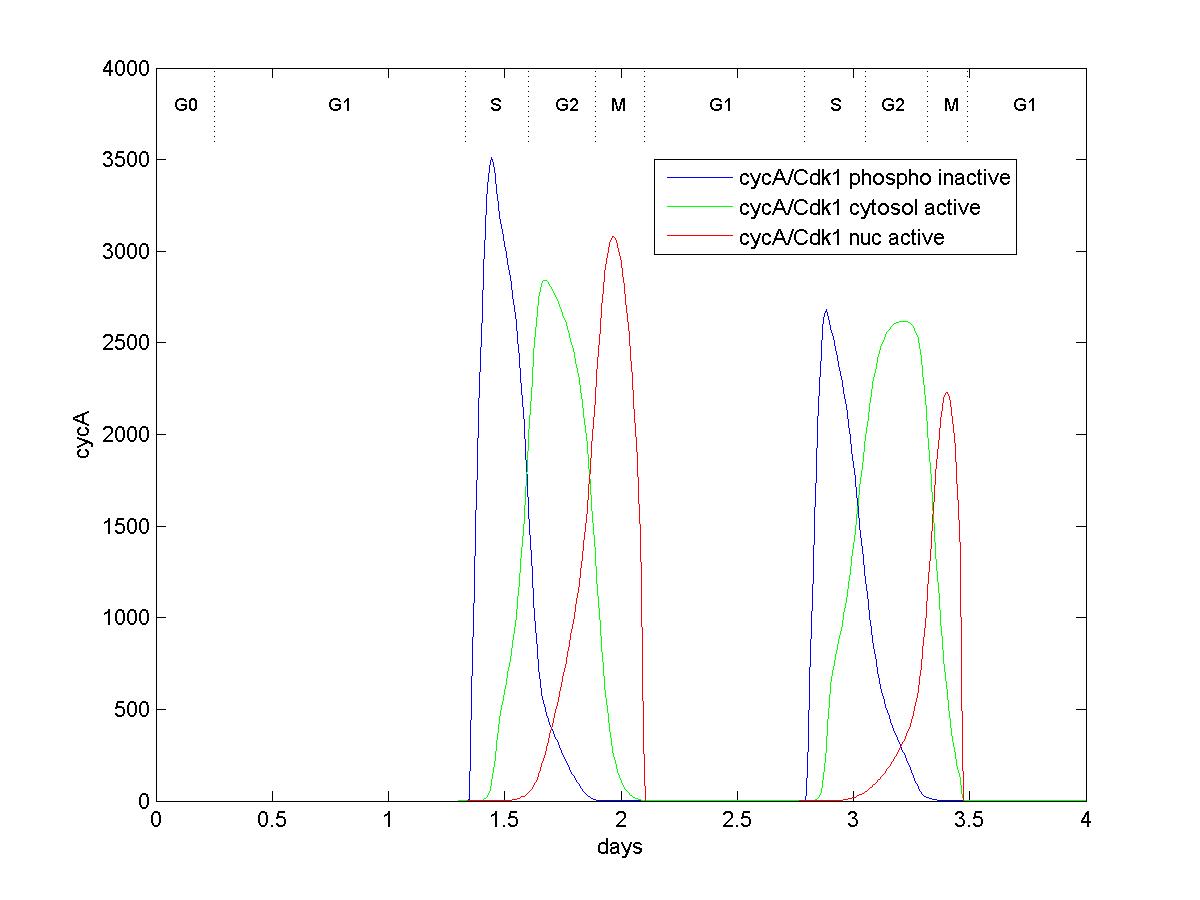
 Figure 3-10. Numbers of cycA/Cdk1 over two cell divisions. cycA is induced and enhanced by mitogen, adhesion factors, E2F, and NF-Y. Cdk1 is induced and enhanced by E2F, B-Myb, NF-Y, and is also expressed constitutively. cycA preferentially binds nuclear Cdk2; a small number bind Cdk1 in the cytosol. A relatively slow, controlled cascade is initiated between cycA/Cdk1 and Cdc25B during G2. Cdc25B is induced and enhanced by E2F and TF-grow. Active cycA/Cdk1 then begins phosphorylation and deactivation of Plk1, and thus progression toward the G2/M transition. Active Plk1 in turn promotes the translocation of cycA/Cdk1 to the nucleus.

Figure 3-10 shows that cycA starts accumulating at the beginning of S phase. Most cycA is shuttled to the nucleus where it binds with Cdk2 (not shown), performing a number of chores, including activating RC and suppressing APC/Cdh1. The cycA/Cdk2 binding is favored over the cycA/Cdk1 binding. Some cycA, however, does bind with Cdk1 in the cytoplasm shortly after it is translated. Although suppressed by Wee1, cycA/Cdk1 does engage in a relatively slow, controlled activation-cascade with Cdc25B (not shown). Thus, a quantity of active cycA/Cdk1 is available throughout G2.

Also shown in Figure 3-10 is the transfer of cycA/Cdk1 to the nucleus during M phase, which is promoted by Plk1 (Figure 3-12). During M phase, cycA and cycB undergo ubiquitination by APC/Cdc20, and at the time of cell division, their levels are so low that APC and Cdh1 bind (Figure 3-6) and abruptly ubiquitinate any remaining cycA and cycB.

Figure 3-11 shows that inactive cycB/Cdk1 begins accumulating at the beginning during S phase, but at a much faster rate than does cycA/Cdk1. The numbers of cycB/Cdk1 are much greater because of greater transcription and because most cycA binds nuclear Cdk2. In the model, Wee1 suppresses cycB/Cdk1 more strongly than it suppresses cycA/Cdk1. Hence, the activation cascade between cycB/Cdk1 and Cdc25C is delayed when compared with the cycA/Cdk1-Cdc25B cascade. As with cycA/Cdk1, cycB/Cdk1 is translocated to the nucleus after activation by Plk1.


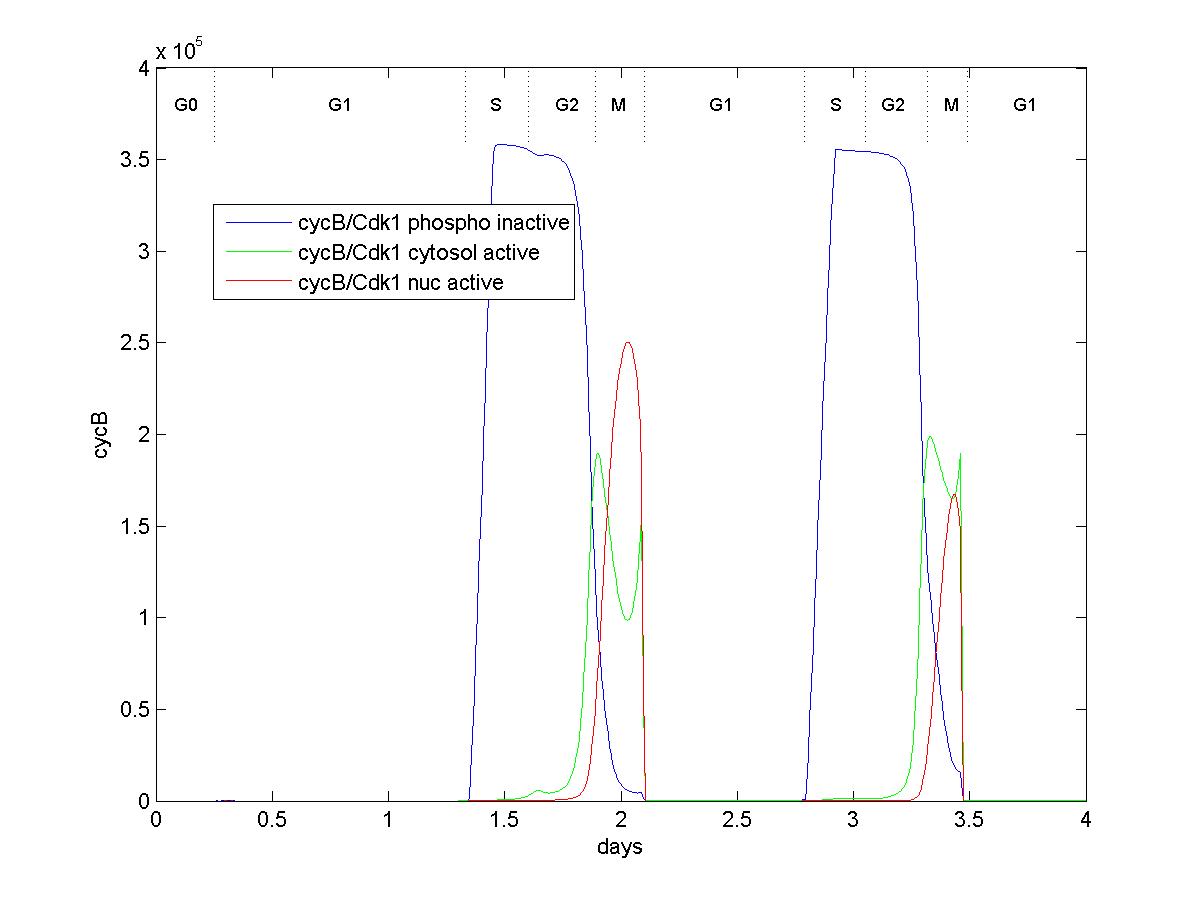
 Figure 3-11. Numbers of cycB/Cdk1 over two cell divisions. cycB and Cdk1 are induced and enhanced by E2F, B-Myb, and NF-Y. Cdk1 also has constitutive expression. cycB/Cdk1 accumulates during S phase but is phosphorylated and inactivated by Wee1. Loss of Wee1 and the presence of active Cdc25B begin the dephosphorylation and activation of cycB/Cdk1 and thus trigger the cascade between cycB/Cdk1 and constitutive Cdc25C. Active cycB/Cdk1 further activates Plk1, which promotes the translocation of cycB/Cdk1 to the nucleus, completing the G2/M transition.

*Modeling cycA (cyclin A) is based on Sherr (1996), Nakayama and Nakayama (2005), Malumbres and Barbacid (2001), Pagano (2006), Chae et al. (2004), and Vodermaier (2004); modeling cycB (cyclin B, including CCNB1 and CCNB2) is based on Novak et al. (2002), Ang and Harper (2004), Van Vugt and Medema (2005), Pagano (2006), Fung and Poon (2005), Chae et al. (2004), and Vodermaier (2004); modeling Cdk1 (cyclin-dependent kinase 1) is based on Donzelli and Draetta (2003), Yun et al. (2003), Pagano (2006), Zhu et al. (2004), Chae et al. (2004), Vodermaier (2004), and Sala et al. (1997). Modeling of Cdc25B and Cdc25C is based on Donzelli and Draetta (2003), Kristjansdottir and Rudolf (2004), Bollen and Beullens (2002), Van Vugt and Medema (2005), Chae et al. (2004), Novak, Sible, and Tyson (2002), and Vazquez-Novelle et al. (2005).*


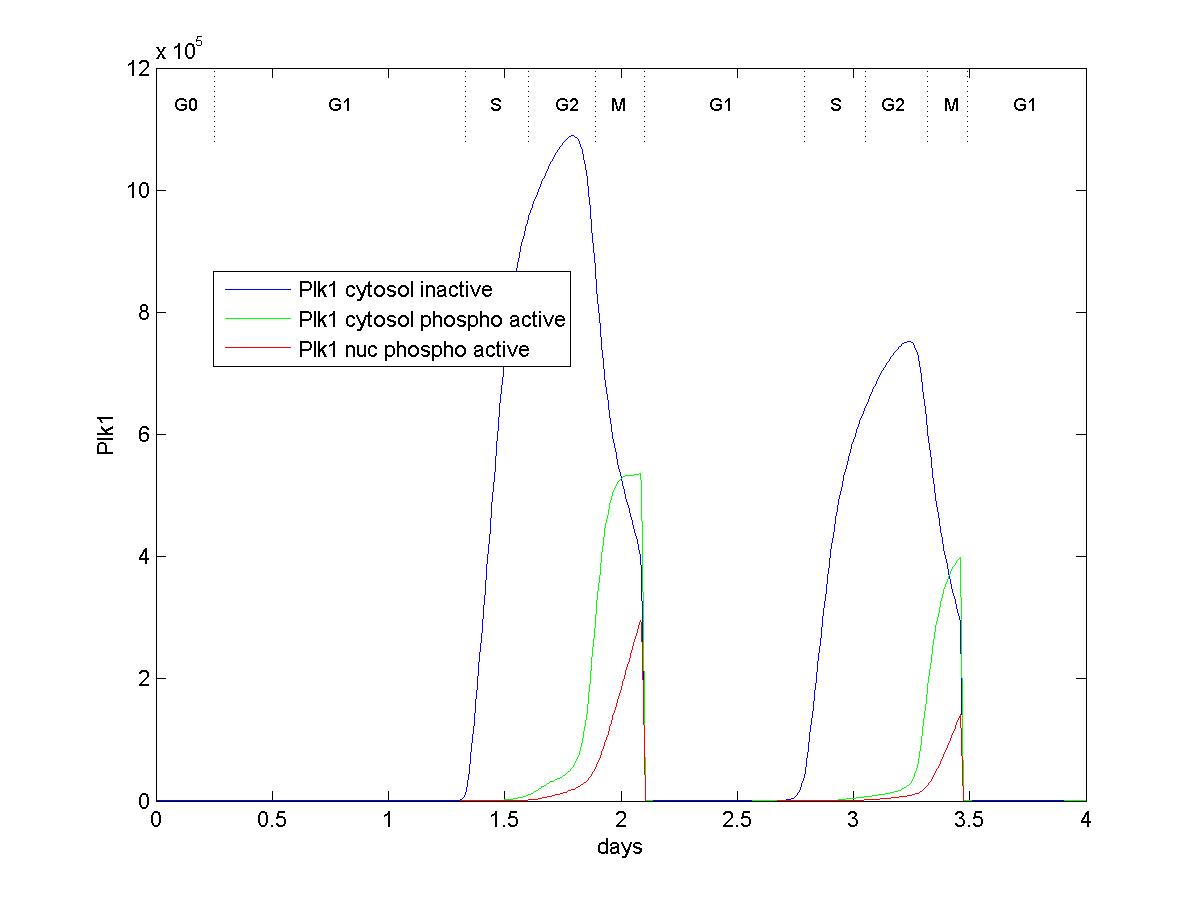
 Figure 3-12. Numbers of Plk1 over two cell divisions. Plk1 is induced and enhanced by E2F and TF-grow, with accumulation starting in S phase. Plk1 is initially inactive; active cycA/Cdk1 begins to phosphorylate and activate Plk1 at the end of S phase. Active Plk1 phosphorylates and suppresses Wee1. With the cycB/Cdk1-Cdc25C cascade, most Plk1 are activated and subsequently translocate to the nucleus.

Plk1 has several functions, including (1) phosphorylation and deactivation of Wee1; (2) translocation of cycA/Cdk1, cycB/Cdk1, and itself into the nucleus; and (3) when it is in the nucleus, activation of Cdc14 (Figure 3-16) and the mitotic exit network. Figure 3-12 shows accumulation of Plk1 begins in early S phase. Phosphorylation and activation of Plk1 by cycA/Cdk1 begins early in G2; however, most activation of Plk1 occurs by cycB/Cdk1 immediately before the G2/M transition. Plk1 translocates to the nucleus after activation, where it activates Cdc14.

*Modeling Plk1 (aka polo-like 1) is primarily based on Ang and Harper (2004) and Van Vugt and Medema (2005).*

During much of the cell cycle, cycB/Cdk1, cycA/Cdk1, and Plk1 are phosphorylated and suppressed by Wee1 and Myt1 (Figure 3-13). In the model, Wee1 represents both Wee1 and Myt1. Active cycA/Cdk1 (and active cycB/Cdk1) are modeled to phosphorylate and activate Plk1, but only Plk1 phosphorylates and inactivates Wee1. (cycB/Cdk1 phosphorylation of Plk1 is suggested by Okano-Uchida, 2003, vice versa is suggested by Fisher and Ferris, 2002, and Van Vugt and Medema, 2005.) Initially, not enough active Plk1 exists to completely inactivate Wee1, because Wee1 spontaneously dephosphorylates and so spontaneously reactivates, suppressing Plk1. Although this dephosphorylation is modeled to occur spontaneously, it could be caused by PP2A phosphatase (OMIM 176915). Thus an equilibrium state is reached, where active and inactive cycA/Cdk1, Cdc25B, Plk1, and Wee1 exist. Wee1 strongly suppresses cycB/Cdk1, so only a small amount of it suppresses G2/M. This equilibrium is apparently robust—a wide range of concentrations and reaction rates can produce it. The equilibrium is maintained until SCF(Btrc) begins to accumulate.


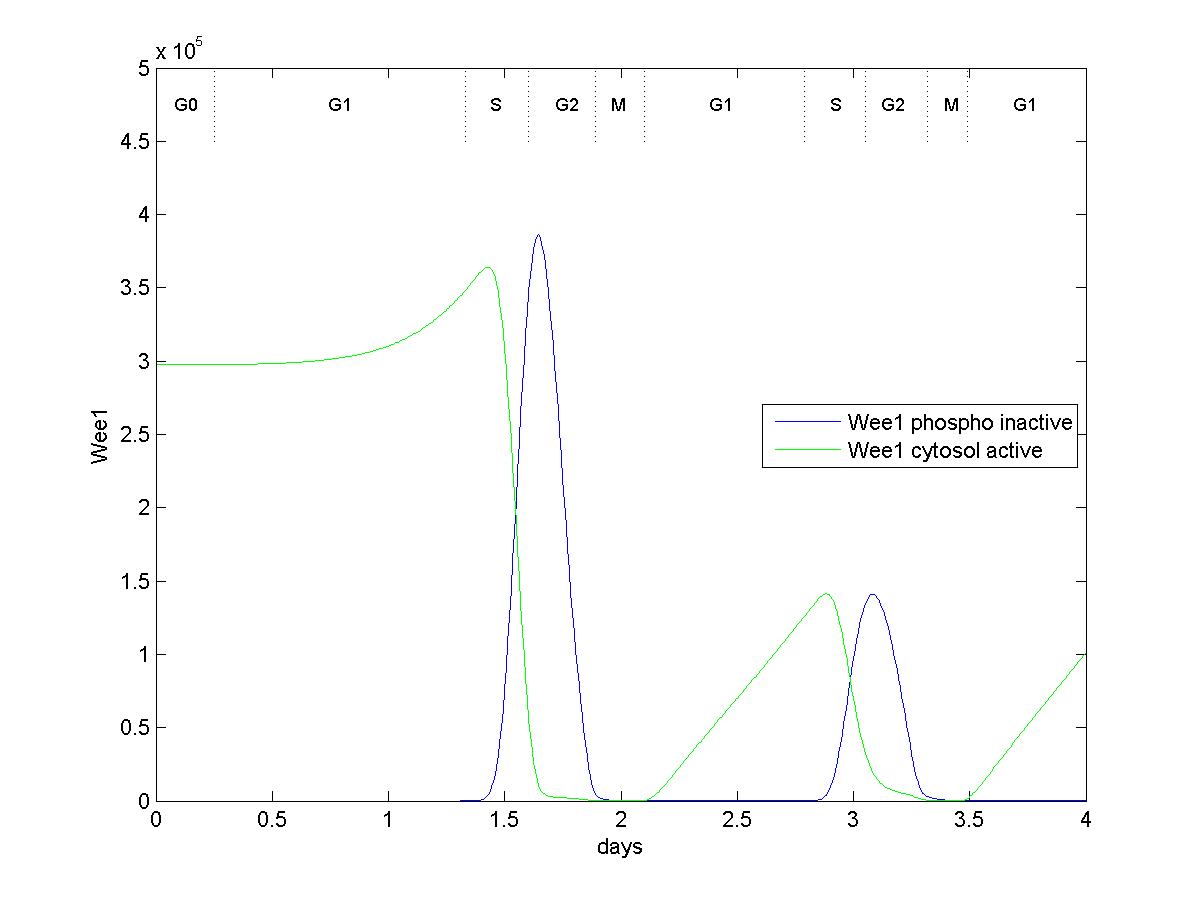
 Figure 3-13. Numbers of Wee1 over two cell divisions. Wee1 is constitutively expressed and function to phosphorylate and deactivate Cdk1. Small numbers of active Plk1 act to phosphorylate and inactivate Wee1 beginning in S phase. Wee 1 tends to dephosphorylate; however, inactive Wee1 is ubiquitinated by SCF(Btrc), limiting this reverse reaction. As SCF(Btrc) only appears after completion of S phase, loss of Wee1 activity and thus the cycB/Cdk1-Cdc25C cascade are guaranteed to occur only after completion of DNA replication.

Figure 3-13 shows that the appearance of active Plk1 phosphorylates and inactivates Wee1 during G2 phase. Inactive Wee1 is subsequently ubiquitinated by SCF(Btrc), limiting the amount that spontaneously dephosphorylates. The complete elimination of Wee1 at the end of G2 allows the cycB/Cdk1 cascade. The absence of cycA/Cdk1 and cycB/Cdk1 during G1 allows Wee1 to reaccumulate. Reaccumulation of active Wee1 insures that cycA/Cdk1 and cycB/Cdk1 are suppressed until well into the next G2 phase.

*Modeling Wee1 (which in the model is also meant to represent Myt1) is based on Ang and Harper (2004), Van Vugt and Medema (2005), Nakayama and Nakayama (2005), Novak, Sible, and Tyson (2002), Bollen and Beullens (2002), and Elledge (1996).*

The function of SCF(Btrc) (Figure 3-7) is to ubiquitinate Wee1, Emi1, Cdc25A, and Cdc25B (although the ubiquitination rate for Cdc25B is very low in the model). SCF(Btrc) accumulates after SCF(Skp2) autoubiquitinates. SCF(Skp2) autoubiquitinates when it has no substrate. Substrates of SCF(Skp2) include Orc1 and Cdt1—proteins in the DNA replication complex (Mendez et al., 2002; Li et al., 2003). These RC proteins are ubiquitinated after they are released from DNA during the replication process. In the model, RC is considered to be a substrate for SCF(Skp2) when it is bound to DNA—even though it cannot be ubiquitinated—and therefore it blocks SCF(Skp2) autoubiquitination. Thus, SCF(Skp2) autoubiquitination only occurs after S phase has successfully completed, and SCF(Btrc) only accumulates after S phase has successfully completed, to some extent insuring that G2 progresses only after DNA replication completes.

Watanabe et al. (2004) report that SCF(Btrc) ubiquitinates phosphorylated Wee1 and is necessary for normal progression through G2/M. (SCF/Tome1 is reported to perform this function in *X. laevis*; Ayad et al., 2003. Guardavaccaro et al. (2003) report that mice lacking SCF(Btrc1) are viable, although Btrc2 might be replacing Btrc1 in this case.) They affirmed the necessity of SCF(Btrc) to cause progression through G2/M by transfecting cells with a Wee1 plasmid to artificially increase Wee1 to levels similar to those in SCF(Btrc) depleted cells and noting a significant increase in the G2/M population. In the example model, SCF(Btrc) removes phosphorylated Wee1 and thus assists in breaking the equilibrium between Wee1 and Plk1 and cycA/Cdk1. As Wee1 is eliminated, cycB/Cdk1 is no longer suppressed, and concurrently active Plk1, active cycA/Cdk1, and active Cdc25B precipitate the cycB/Cdk1-Cdc25C cascade.

Note however that, in the model, SCF(Btrc) acts only secondarily to the initial phosphorylation and deactivation of Wee1. This initial phosphorylation is the result of Cdc25A and Cdc25B activating cycA/Cdk1, which in turn activates Plk1.

In the model, there is included an intermediary step of a cycA/Cdk1-Cdc25B cascade, and this process could add more complication than necessary. Some evidence supporting this complication is suggested by Cans et al., 1999, Lammer et al., 1998, Nishijima et al., 1997, Baldin et al., 1997, and Lindqvist et al., 2005. This intermediary step does allow cycB/Cdk1 and Cdc25C to remain completely inactive during most of G2. Also, it offers an explanation why cycA binds Cdk1 and apparently participates in G2/M. And it offers an explanation why Cdc25B is apparently required for viability while Cdc25C apparently is not. (There is some disagreement in the literature about the necessity of Cdc25B and Cdc25C: Lincoln et al., 2002, report that mice lacking Cdc25B are viable; Donzelli and Draetta, 2003, and Lindqvist et al., 2005, report that Cdc25C knockout mice are viable, but Cdc25B knockouts are not, while Ferguson et al., 2005, report that mice lacking both Cdc25B and Cdc25C are viable. However, Lammer et al., 1998 report that Cdc25B is required for the human cell cycle.) In the model, Cdc25B can substitute for Cdc25C in the cycB/Cdk1-Cdc25C cascade, albeit less efficiently than Cdc25C, but Cdc25C cannot substitute in the cycA/Cdk1-Cdc25B cascade because it is very inactive. Therefore, Cdc25B is essential in the model, but Cdc25C is dispensable. As mentioned in Calibration of the Model (Additional file 2), however, calibration might be improved in these areas.

The process laid out here explains why Watanabe et al. find SCF(Btrc) to be important to the G2/M transition. Because of the excess of Wee1 in their experimental conditions, and because Wee1 spontaneously dephosphorylates (or is dephosphorylated by a phosphatase, as mentioned above), there is not sufficient cycA/Cdk1 and Plk1 activity to completely suppress Wee1. However, when SCF(Btrc) is present, it removes the Wee1 that is suppressed and thus acts to advance the process. So inclusion of SCF(Btrc) in the cell cycle has an interesting benefit—it mediates the duration of G2 in a manner independent of the concentrations of the other participants, and thus it enforces an acceptable amount of cell growth in G2.

Watanabe et al. also report, however, that depletion of SCF(Btrc) via siRNA has no obvious affect on the G2/M. This apparent contradiction is discussed in Alternative G2/M Trigger (Additional file 4).

*Cell cycle—M phase*

M phase lasts 4 hours and follows the cycB/Cdk1-Cdc25C cascade. Cell division occurs during M phase.

One consequence of the activation of cycB/Cdk1 is the phosphorylation of Emi1. Phosphorylation of Emi1 causes it to release Cdc20 (Figure 3-14) and Cdh1 (Figure 3-6). In the model, APC binding with Cdc20 is favored over its binding with Cdh1. APC(Cdc20) is activated (phosphorylated) in the nucleus by cycA/Cdk2, cycA/Cdk1, and cycB/Cdk1. APC(Cdc20) then ubiquitinates Securin (Figure 3-15).


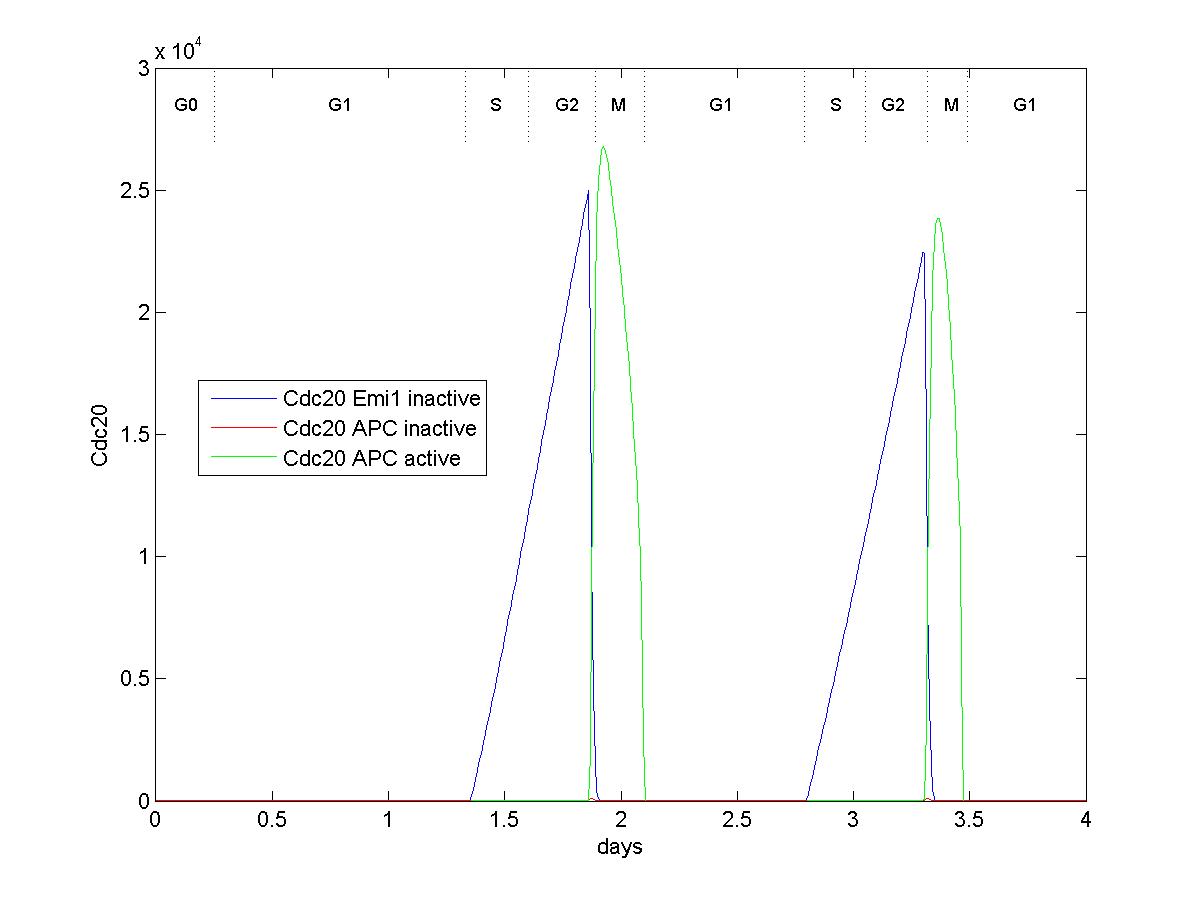


Figure 3-14. Numbers of Cdc20 over two cell divisions. Cdc20 is constitutively expressed but is ubiquitinated by APC(Cdh1), hence delaying accumulation until S phase. Initially, Cdc20 is held inactive by E2F-induced Emi1. Phosphorylation by active cycA/Cdk1 and active cycB/Cdk1 release Cdc20 at the G2/M transition. Cdc20 binds constitutive APC, and APC(Cdc20) then ubiquitinates Securin, which allows chromotid separation and cell division. After cell division, Cdc20 is released from APC by Cdc14-mediated dephosphorylation and again it is ubiquitinated by APC(Cdh1).

Figure 3-15 shows Securin accumulation beginning early in G1, culminating in its ubiquitination during and after G2/M. One role of Securin is to inhibit Cdc14; once Securin is ubiquitinated, Cdc14 (Figure 3-16) can be activated by Plk1. Another role of Securin is keep the chromotids connected. With the ubiquitination of Securin, the chromosome pairs separate and the cell divides.


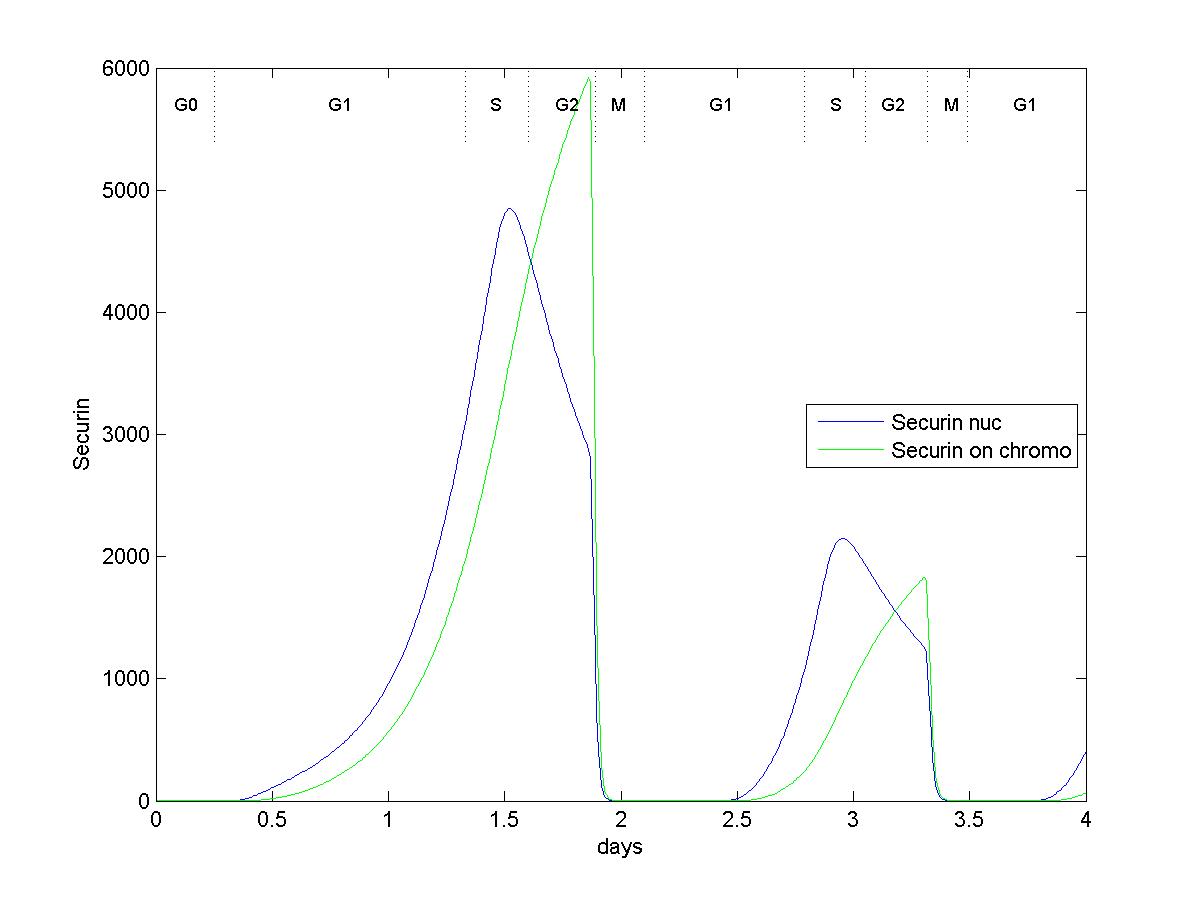


Figure 3-15. Numbers of Securin over two cell divisions. Securin functions to inhibit Cdc14 and to prevent the Separase protein from causing chromotids to separate. Securin is induced by E2F and accumulates throughout the cell cycle. At the G2/M transition, Securin is ubiquitinated by APC(Cdc20).

Figure 3-16 shows that quantities of the phosphatase Cdc14 exist throughout the cell cycle, although usually in an inactive state. When active Plk1 moves into the nucleus, and when Securin has been ubiquitinated by APC(Cdc20), Plk1 can phosphorylate and active Cdc14 (after Shou et al., 2002—although little is known about this pathway in humans and this model is a simplification). In the model, Cdc14 represents the entire mitotic-exit-network (MEN) pathway. Cdc14 causes the cell to exit M phase by deactivating Cdc20, Cdc25A, Cdc25B, Cdc25C, and reactivating Wee1 (when it is again translated), Cdh1, and, in the model, cycC/Cdk8 (although no evidence could be found for this mechanism for reactivating cycC/Cdk8). Of immediate importance at the end of M phase is the unbinding of APC and Cdc20 by Cdc14. Unbinding of APC(Cdc20) allows the binding of APC and Cdh1, which causes the cell to enter either G0 or G1 (depending on the presence of mitogen), thus completing this particular cell cycle.


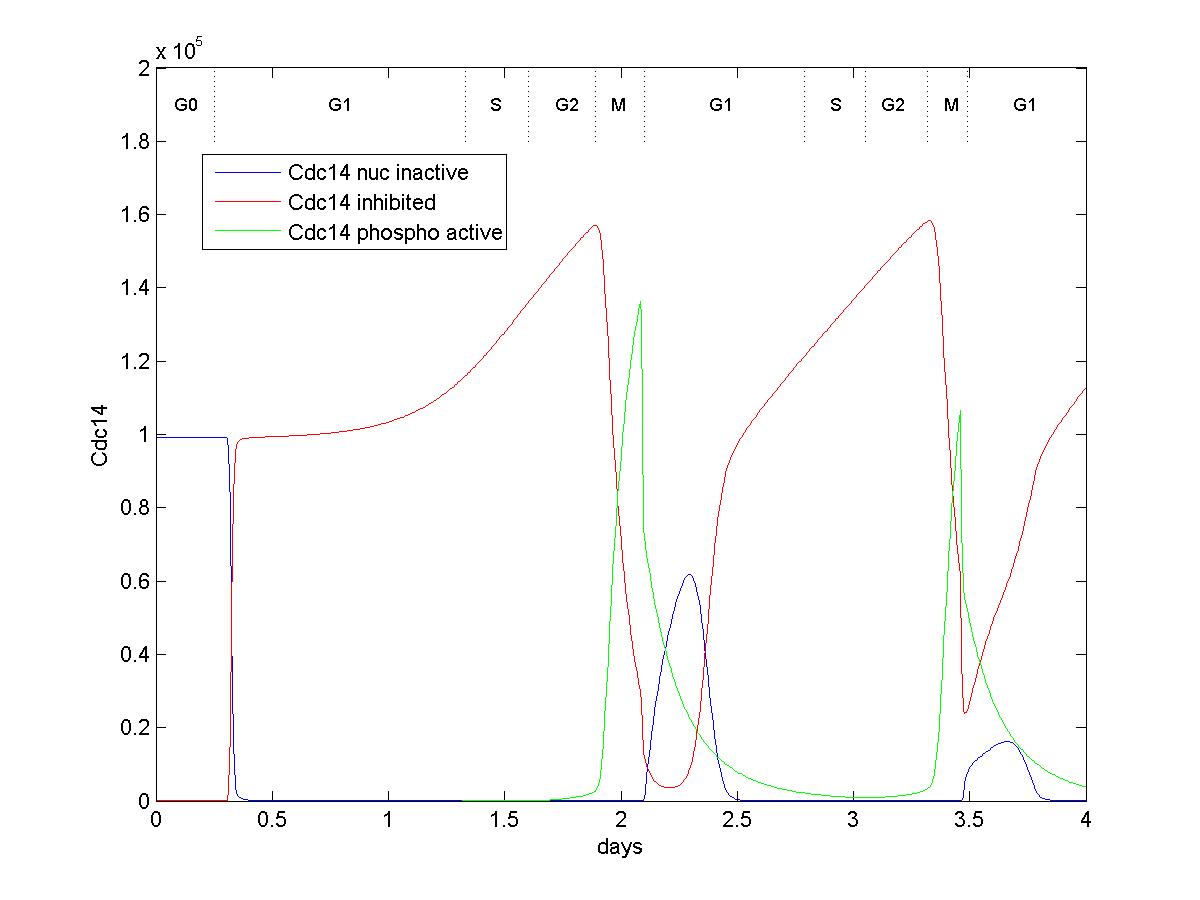
 Figure 3-16. Numbers of Cdc14 (representing the MEN pathway) over two cell divisions. Cdc14 is a phosphatase that functions to activate p27, Wee1, and Cdh1 and deactivate the Cdc25 phosphatases, thus resetting the cell cycle after cell division. Cdc14 is constitutively expressed and translocated to the nucleus. Most of cell cycle, Cdc14 is inhibited by Securin. Cdc14 is activated when phosphorylated by nuclear Plk1after Securin is degraded.

*Modeling Cdc20 (aka Fizzy) is based on Novak, Sible, and Tyson (2002), Ang and Harper (2004), and Pagano (2006); modeling Securin (Pds1) is based on Novak, Sible, and Tyson (2002), and Pagano (2006); modeling Cdc14 (hCdc14a, here representing MEN (Cdc5-?-?-?-Cdc14) and FEAR (Cdc5-Slk9-Spo12-Esp1) pathways) is based on Ang and Harper (2004), Novak, Sible, and Tyson (2002), Bollen and Beullens (2002), Stegmeier and Amon (2004), Bennbenek and Yu (2001), and Vazquez-Novelle et al. (2005). Modeling of Emi1 is based on Nakayama and Nakayama (2005), Ang and Harper (2004), and Pagano (2006).*

**References**

Alberts, B., D. Bray, J. Lewis, M. Raff, K. Roberts, and J.D. Watson, *Molecular Biology of the Cell*, 3^rd^ Edition, Garland Publishing, Inc., New York, 1994.

Amati, B, Myc degradation: dancing with ubiquitin ligases, *Proc Natl Acad Sci USA*, **101**(24), 8843–8844, 15 June 2004. Epub 8 June 2004.

Ang, X.L., and J.W. Harper, Interwoven ubiquitination oscillators and control of cell cycle transitions, *Sci STKE*, **2004**(242), pe31, 13 July 2004.

Ayad, N.G., S. Rankin, M. Murakami, J. Jebanathirajah, S. Gygi, and M.W. Kirschner, Tome-1, a trigger of mitotic entry, is degraded during G1 via the APC, *Cell*, **113**(1), 101–113, 4 Apr 2003.

Baldin, V., C. Cans, M. Knibiehler, and B. Ducommun, Phosphorylation of human CDC25B phosphatase by CDK1-cyclin A triggers its proteasome-dependent degradation, *J Biol Chem*, **272**(52), 32731–4, 26 Dec 1997.

Bartek, J., and J. Lukas, Cell cycle. Order from destruction, *Science*, **294**(5540), 66–67, 5 Oct 2001.

Bembenek, J., and H. Yu, Regulation of the anaphase-promoting complex by the dual specificity phosphatase human Cdc14a, *J Biol Chem*, **276**(51), 48237–42, 21 Dec 2001. Epub 11 Oct 2001.

Bollen, M., and M. Beullens, Signaling by protein phosphatases in the nucleus, *Trends Cell Biol*, **12**(3), 138–145, Mar 2002.

Cans, C., B. Ducommun, and V. Baldin, Proteasome-dependent degradation of human CDC25B phosphatase, *Molecular Biology Reports*, **26**, 53–57, 1999.

Chae, H.D., J. Yun, Y.J. Bang, and D.Y. Shin, Cdk2-dependent phosphorylation of the NF-Y transcription factor is essential for the expression of the cell cycle-regulatory genes and cell cycle G1/S and G2/M transitions, *Oncogene*, **23**(23), 4084–4088, 20 May 2004.

Cicchillitti, L., S.A. Jimenez, A. Sala, and B. Saitta, B-Myb acts as a repressor of human COL1A1 collagen gene expression by interacting with Sp1 and CBF factors in scleroderma fibroblasts, *Biochem J*, **378**(Pt 2), 609–616, 1 Mar 2004.

Donzelli, M., and G.F. Draetta, Regulating mammalian checkpoints through Cdc25 inactivation, *EMBO Rep*, **4**(7), 671–677, July 2003.

Eggen, B.J.L., G.F.J.D. Benus, S. Folkertsma, L.J. Jonk, and W. Kruijer, TAK1 activation of the mouse JunB promoter is mediated through a CCAAT box and NF-Y, *FEBS Letters 506*, 267–271, 2001.

Elledge, S.J., Cell cycle checkpoints: preventing an identity crisis, *Science*, **274**, 1664–1672, 6 Dec 1996.

Ferguson, A.M., L.S. White, P.J. Donovan, and H. Piwnica-Worms, Normal cell cycle and checkpoint responses in mice and cells lacking Cdc25B and Cdc25C protein phosphatases, *Mol Cell Biol*, **25**(7), 2853–2860, Apr 2005.

Fisher, R.A.H., and D.K. Ferris, The functions of polo-like kinases and the relevance to human disease, *Curr Med Chem—Imun Endoc & Metab Agents*, **2**, 125–134, 2002.

Fung, T.K., and R.Y. Poon, A roller coaster ride with the mitotic cyclins, *Semin Cell Dev Biol*, **16**(3), 335–342, Jun 2005.

Guardavaccaro, D., Y. Kudo, J. Boulaire, M. Barchi, L. Busino, M. Donzelli, F. Margottin-Goguet, P.K. Jackson, L. Yamasaki, and M. Pagano, Control of meiotic and mitotic progression by the F box protein beta-Trcp1 in vivo, *Dev Cell*, **4**(6), 799–812, June 2003.

Gurtner, A., I. Manni, P. Fushi, R. Mantovani, F. Guadagni, A. Sacchi, and G. Piaggio, Requirement for down-regulation of the CCAAT-binding activity of the NF-Y transcription factor during skeletal muscle differentiation, *Mol Biol Cell*, **14**(7), 2706–2715, July 2003.

Kamura, T., T. Hara, M. Matsumoto, N. Ishida, F. Okumura, S. Hatakeyama, M. Yoshida, K. Nakayama, K.I. Nakayama, Cytoplasmic ubiquitin ligase KPC regulates proteolysis of p27(Kip1) at G1 phase, *Nat Cell Biol*, **6**(12), 1229–1235, Dec 2004. Epub 7 Nov 2004.

Kristjansdottir, K., and J. Rudolph, Cdc25 phosphatases and cancer, *Chem Biol*, **11**(8), 1043–1051, Aug 2004.

Lammer, C., S. Wagerer, R. Saffrich, D. Mertens, W. Ansorge, and I. Hoffmann, The cdc25B phosphatase is essential for the G2/M phase transition in human cells, *J Cell Sci*, **111** ( Pt 16), 2445–2453, Aug 1998.

Li X., Q. Zhao, R. Liao, P. Sun, and X. Wu, The SCF(Skp2) ubiquitin ligase complex interacts with the human replication licensing factor Cdt1 and regulates Cdt1 degradation, *J Biol Chem*, **278**(33), 30854–30858, 15 Aug 2003. Epub 2 July 2003.

Lincoln, A.J., D. Wickramasinghe, P. Stein, R.M. Schultz, M.E. Palko, M.P. De Miguel, L. Tessarollo, and P.J. Donovan, Cdc25b phosphatase is required for resumption of meiosis during oocyte maturation, *Nat Genet*, **30**(4), 446–449, Apr 2002. Epub 25 Mar 2002.

Lindqvist, A., H. Kallstrom, A. Lundgren, E. Barsoum, and C.K. Rosenthal, Cdc25B cooperates with Cdc25A to induce mitosis but has a unique role in activating cyclin B1-Cdk1 at the centrosome, *J Cell Biol*, **171**(1), 35–45, 10 Oct 2005.

Malumbres, M., and M. Barbacid, To cycle or not to cycle: a critical decision in cancer, *Nat Rev Cancer*, **1**(3), 222–231, Dec 2001.

Mendez, J., X.H. Zou-Yang, S.Y. Kim, M. Hidaka, W.P. Tansey, and B. Stillman, Human origin recognition complex large subunit is degraded by ubiquitin-mediated proteolysis after initiation of DNA replication, *Mol Cell*, **9**(3), 481–491, Mar 2002.

Nakayama, K.I., and K. Nakayama, Regulation of the cell cycle by SCF-type ubiquitin ligases, *Semin Cell Dev Biol*, **16**(3):323–333, Jun 2005.

Nasmyth, K., Viewpoint: putting the cell cycle in order, *Science*, **274**, 1643–1645, 6 Dec 1996.

Nishijima, H., H. Nishitani, T. Seki, and T. Nishimoto, A dual-specificity phosphatase Cdc25B is an unstable protein and triggers p34cdc2/cyclin B activation in hamster BHK21 cells arrested with hydroxyurea, *J Cell Biol*, **138**(5), 1105–1116, 8 Sep 1997.

Novák, B., J.C. Sible, and J.J. Tyson, Checkpoints in the Cell Cycle, in Encyclopedia of Life Sciences, London: Nature Publishing Group, 2002. http://www.els.net/

Okano-Uchida, T., E. Okumura, M. Iswahita, H. Yoshida, K. Tachibana, and T. Kishimoto, Distinct regulators for Plk1 activation in starfish meiotic and early embryonic cycles, *EMBO J*, **22**(20), 5633–5642, 2003.

OMIM 176915, Protein phosphatase 2, Online Mendelian Inheritance in Man, no date, retrieved 20 April 2007.

Pagano, M., Control of the cell cycle by the ubiquitin system, *ACBF001-Ed-Book*, **1**(32), 162–165, [http://pathology.med.nyu.edu/Pagano/PDFs/162%20MP.pdf](http://pathology.med.nyu.edu/Pagano/PDFs/162 MP.pdf), 4 Mar 2006.

Quintana, D.G., and A. Dutta, The metazoan origin recognition complex, *Front Biosci*, **4**, D805–815, 1 Dec 1999.

Robinson, C., Y. Light, R. Groves, D. Mann, R. Marias, and R. Watson, Cell-cycle regulation of B-Myb protein expression: specific phosphorylation during the S phase of the cell cycle, *Oncogene*, **12**(9), 1855–1864, 2 May 1996.

Sala, A., I. Casella, T. Bellon, B. Calabretta, R.J. Watson, and C. Peschle, B-myb promotes S phase and is a downstream target of the negative regulator p107 in human cells, *J Biol Chem*, **271**(16), 9363–9367, 19 Apr 1996.

Sala, A., M. Kundu, I. Casella, A. Engelhard, B. Calabretta, L. Grasso, M.G. Paggi, A. Giordano, R.J. Watson, K. Khalili, and C. Peschle, Activation of human B-MYB by cyclins, *Proc Natl Acad Sci USA*, **94**(2), 532–536, 21 Jan 1997.

Sherr, C.J., Cancer cell cycles, Science, **274**, 1672–1677, 6 December 1996. DOI: 10.1126/science.274.5293.1672

Shou, W., R. Azzam, S.L. Chen, M.L. Huddleston, C. Baskerville, H. Charbonneau, R.S. Annan, S.A. Carr, and R.J. Deshaies, Cdc5 influences phosphorylation of Net1 and disassembly of the RENT complex, *BMC Molecular Biology*, **3**(3), 17 April 2002. doi:10.1186/1471-2199-3-3.

Sim, K.G., Z. Zang, C.M. Yang, J.V. Bonventre, and S.I. Hsu, TRIP-Br links E2F to novel functions in the regulation of cyclin E expression during cell cycle progression and in the maintenance of genomic stability, *Cell Cycle*, **3**(10), 1296–1304, Oct 2004. Epub 6 Oct 2004.

Stegmeier, F., and A. Amon, Closing mitosis: the functions of the Cdc14 phosphatase and its regulation, *Annu Rev Genet*, **38**, 203–232, 2004.

van Vugt, M.A., and R.H. Medema, Getting in and out of mitosis with Polo-like kinase-1, *Oncogene*, **24**(17), 2844–2859, 18 Apr 2005.

Vazquez-Novelle, M.D., V. Esteban, A. Bueno, and M.P. Sacristan, Functional homology among human and fission yeast Cdc14 phosphatases, *J Biol Chem*, **280**(32), 29144–29150, 12 Aug 2005. Epub 23 May 2005.

Vodermaier, H.C., APC/C and SCF: controlling each other and the cell cycle, *Curr Biol*, **14**(18), R787–796, 21 Sep 2004.

Watanabe, N., H. Arai, Y. Nishihara, M. Taniguchi, T. Hunter, and H. Osada, M-phase kinases induce phospho-dependent ubiquitination of somatic Wee1 by SCF{beta}-TrCP, *Proc Natl Acad Sci USA*, **101**(13), 4419–4424, 30 Mar 2004. Epub 22 Mar 2004.

Yam, C.H., T.K. Fung, and R.Y. Poon, Cyclin A in cell cycle control and cancer, *Cell Mol Life Sci*, **59**(8), 1317–1326, Aug 2002.

Yun, J., H-D Chae, T-S Choi, E-H Kimm, Y-J Bang, J. Chung, K-S Choi, R. Mantovani, and D.Y. Shin, Cdk2-dependent phosphorylation of the NF-Y transcription factor and its involvement in the p53-p21 signaling pathway, j Biol Chem, 278(38), 36966-972, 19 Sep 2003.

Zhao, J., A. Bilsland, K. Jackson, and W.N. Keith, MDM2 negatively regulates the human telomerase RNA gene promoter, *BMC Cancer*, **5**, 6, 18 Jan 2005.

Zhou, P., and P.M. Howley, Ubiquitination and degradation of the substrate recognition subunits of SCF ubiquitin-protein ligases, *Mol Cell*, **2**(5), 571–580, Nov 1998.

Zhu, W., P.H. Giangrande, and J.R. Nevins, E2Fs link the control of G1/S and G2/M transcription, *EMBO J*, **23**(23), 4615–4626, 24 Nov 2004. Epub 28 Oct 2004.
