# Supplementary material for: A general framework for modeling growth and division of mammalian cells
Source: BMC Syst Biol. 2011 Jan 6;5:3. doi: 10.1186/1752-0509-5-3 (PMC3025838; doi:10.1186/1752-0509-5-3)
Supplement: Additional file 4 — Alternative G2/M Trigger. Investigation of an alternative trigger for G2/M. [file 1752-0509-5-3-S4.DOC]

**Additional file 4**

**Alternative G2/M Trigger**

A major question in our understanding of the cell cycle involves the triggering of the cycB/Cdk1-Cdc25C cascade and thus the triggering of the G2/M transition (O’Farrell 2001). Watanabe et al. (2004) find that SCF/Btrc is important to the G2/M transition; they also note, however, that depletion of SCF(Btrc) via siRNA interference has no discernable effect on the cell cycle. They conjecture that Cdc25A, an SCF(Btrc) substrate that would normally be ubiquinated and degraded during G2, accumulates and counteracts the Wee1 inhibition of the cycB/Cdk1-Cdc25C cascade.

It has already been shown (Additional file 3) that Cdc25A, SCF(Btrc), and other molecules team to instigate the cycB/Cdk1-Cdc25C cascade. Here, it is shown that Cdc25A accomplishes this action by itself in the absence of SCF(Btrc). This situation is an example of perhaps the simplest form of an alternate pathway—the function is accomplished in the presence or absence of one of the participating molecules.

The only difference between the results presented here and those presented in the rest of this paper is that Btrc transcription is effectively zeroed out. All proteins in the cell-cycle model have a transcription rate that determines how often RNA polymerases visit the gene, in the absence of transcription factors. This rate is reduced by four orders of magnitude for Btrc. Execution of the model shows that the cell cycle still progresses. The abundance plots for all of the proteins are similar for the Btrc-presence case and the Btrc-omission case, except for those plots involving Cdc25A, Wee1, and cycB/Cdk1 (as well as SCF(Btrc), of course).

Figure 4-1 shows that SCF(Btrc) is now omitted from the cell cycle. Also noticeable in the figure is that SCF abundances increase sooner in the second cycle of the Btrc-omitted case. Indeed, the entire cell cycle is speeded up. The major affect of this quickening is that the cell is somewhat smaller when it divides—about 1.6 times the steady-state size as opposed to the 1.7 times the steady-state size in the case where SCF(Btrc) is present. It is assumed that the cell rebounds to its steady-state size when divisions cease, although this assumption is not investigated. The speedup occurs because SCF(Btrc) acts to ubiquitinate Wee1, Emi1, Cdc25A, and Cdc25B, and the increased levels of Cdc25 promote more rapid cell division.

Figures 4-2 shows that, in the absence of ubiquitination by SCF(Btrc), Cdc25A is more abundant and, importantly, this abundance is now maintained throughout G2 and M. Early in the following G1 phase, Cdc25A is finally dephosphorylated by Cdc14 and ubiquitinated by APC(Cdh1).

Figure 4-3 indicates that Wee1 is now present during late G2 and M, albeit phosphorylated and deactivated. Inactive Wee1 allows the cycB/Cdk1-Cdc25C cascade to occur, although Figure 4-4 indicates that, for this parameterization of the cell-cycle model, the cascade is feeble. Only about 20 percent of the amount of active cycB/Cdk1 is generated without the SCF(Btrc) timing mechanism, probably because the suppression of Wee1 is less effective. Remember, in the cell-cycle model, Wee1 spontaneously dephosphorylates (Additional file 3); residual levels are enough to mitigate, although not completely suppress, cycB/Cdk1 activity. However, when Cdc25A initiates the cascade, active cycB/Cdk1 does begin to accumulate sooner. This reduced amount of cycB/Cdk1 is sufficient to release Cdc20 from Emi1, allowing the formation of APC(Cdc20) and the initiation of M phase.

These results support the conjecture of Watanabe et al. The differences in abundances of Wee1, Cdc25A, and cycB/Cdk1 shown here present an opportunity to test this hypothesis and to test the operation of the cell model. An experiment might be devised as follows. Consider two groups of cells: one group is unmodified (wild type), and the other has Btrc depleted using siRNA. Synchronize and stop the cells near the G2/M transition. Determine differences in abundances or the activities of Cdc25A and Wee1. Abundant active Cdc25A and abundant inactive Wee1 in the Btrc-depleted cells compared to the unmodified cells would provide evidence for this hypothesis.

O’Farrell (2001) proposes three possible timing mechanisms for the cycB/Cdk1-Cdc25C cascade: (1) “bursting the dam,” where the gradual build up of the participant molecules eventually triggers the cascade; (2) “lowering the bar,” where the activity of inhibitory molecules is reduced until the cascade is triggered; and (3) “changing the rules,” where an unknown or unexpected molecule or reaction intervenes and causes the cascade to trigger. The SCF ubiquitinases provide a lowering-the-bar mechanism; Cdc25A provides a bursting-the-dam mechanism. It is interesting to note that the cycE/Cdk2-Cdc25A cascade that initiates S phase is triggered by another lowering-the-bar mechanism: the suppression of APC(Cdh1) by cycD/Cdk4or6. The basic strategies differ, however, as S/G1 triggering involves a ubiquitinase suppressed by a kinase, while the primary mechanism for G2/M triggering involves a kinase suppressed by a ubiquitinase.

The alternative trigger for the cycB/Cdk1-Cdc25C cascade and G2/M motivates several questions. Are there other ways to trigger G2/M other than the two presented here? Why are so many molecules, some perhaps not absolutely necessary, involved in G2/M triggering? Do more pathways cause a less robust system? Are the Cdc25B and Cdc25A pathways actually redundant or are both needed for G2/M triggering (as currently is the case in the example cell-cycle model)? With different values for the rate constants, could Cdc25B be removed from the G2/M triggering process? With different rate constants, could Cdc25A be removed from the G2/M triggering process? If the Cdc25A and Cdc25B pathways (and perhaps other pathways) are redundant and necessary, why would the cell invest so much complexity in saving a cell division when the cell is defective in one of the pathways?

**References**

O’Farrell, P.H., Triggering the all-or-nothing switch into mitosis, *TRENDS in Cell Biology*, **11**(12), 512–519, Dec 2001.

Watanabe, N., H. Arai, Y. Nishihara, M. Taniguchi, T. Hunter, and H. Osada, M-phase kinases induce phospho-dependent ubiquitination of somatic Wee1 by SCF{beta}-TrCP, *Proc Natl Acad Sci USA*, **101**(13), 4419–4424, 30 Mar 2004. Epub 22 Mar 2004.


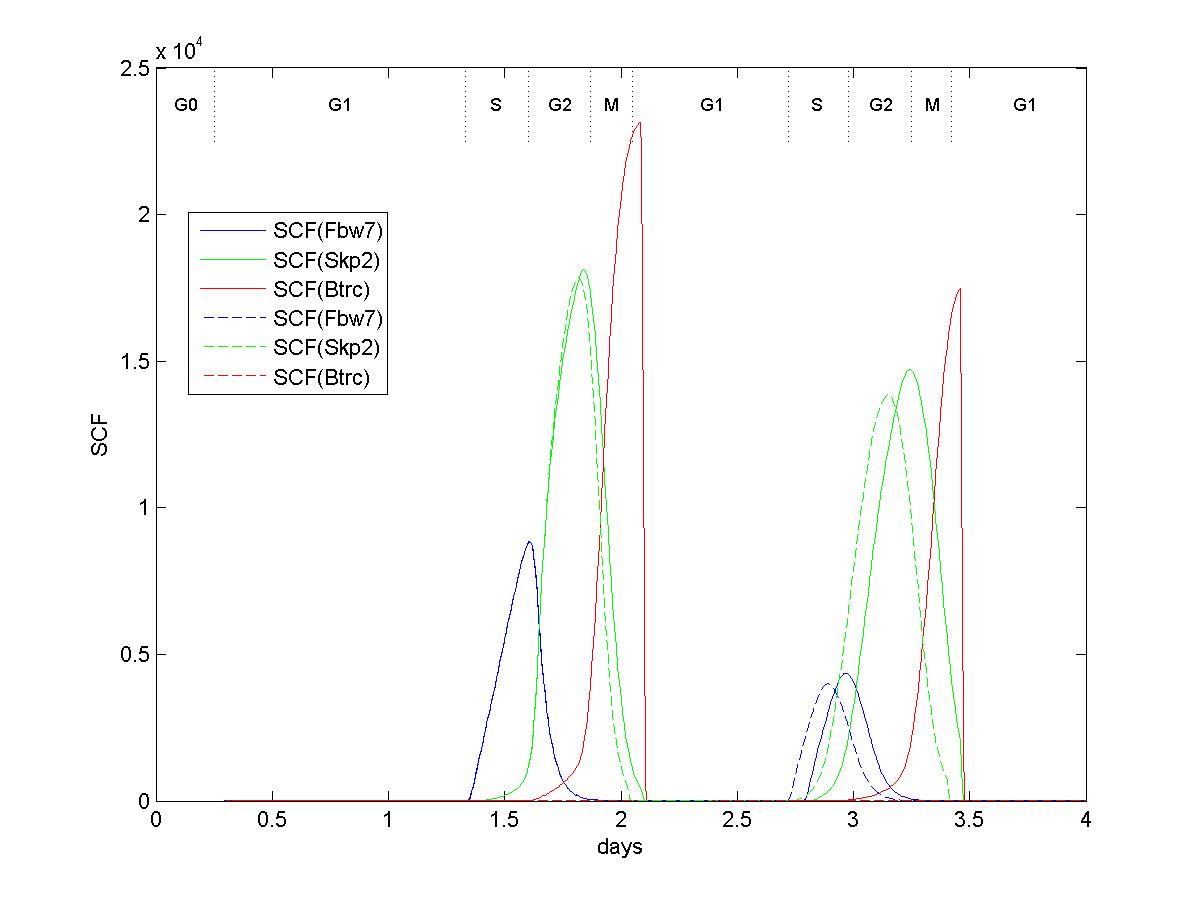
 Figure 4-1. Numbers of SCF bound to subunits Fbw7, Skp2, and Btrc over two cell divisions. Solid lines represent calculation with SCF(Btrc) (as in Additional file 3, Figure 3-7); dashed lines represent calculation without SCF(Btrc). Shown is that reduction of Btrc transcription by 4 orders of magnitude has effectively removed Btrc from the problem.


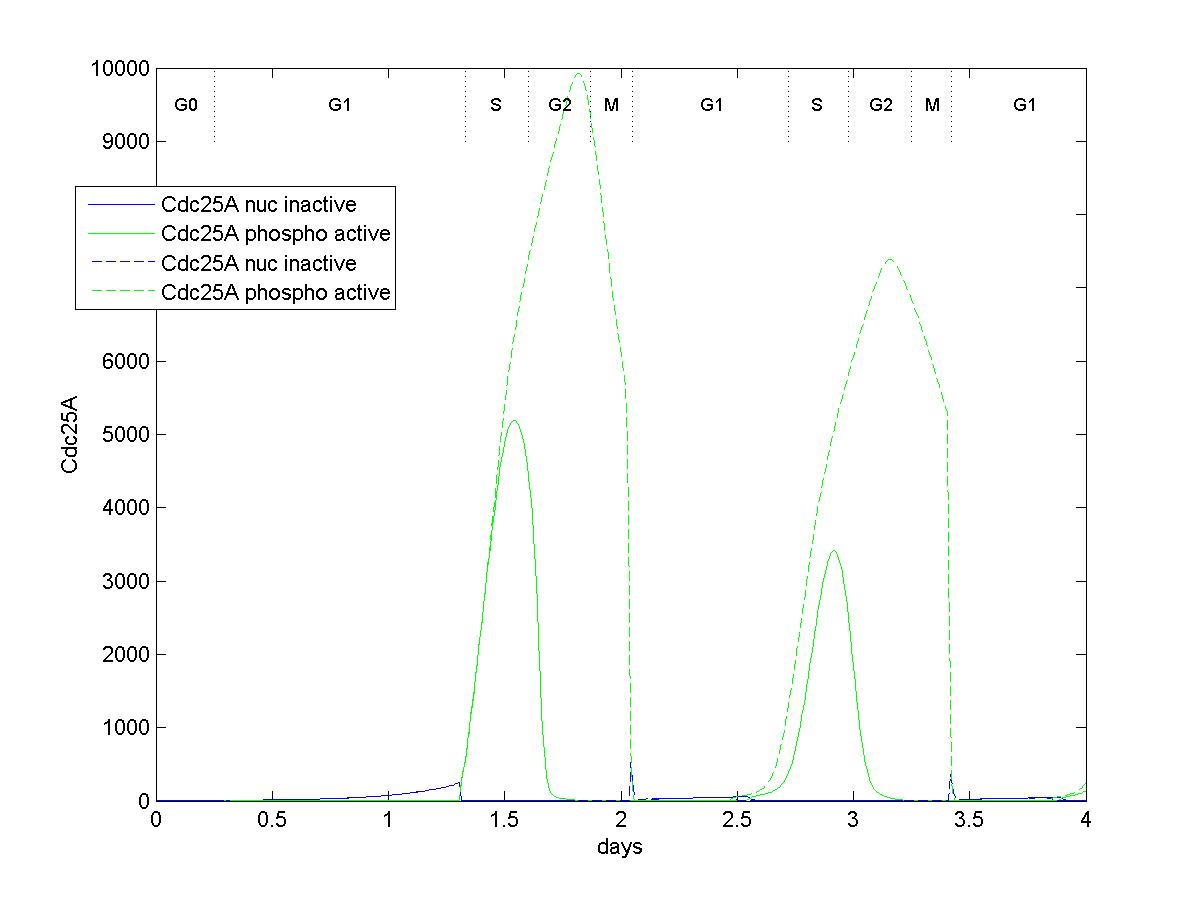
 Figure 4-2. Numbers of Cdc25A over two cell divisions. Solid lines represent calculation with SCF(Btrc) (as in Additional file 3, Figure 3-5); dashed lines represent calculation without SCF(Btrc). Greater numbers of SCF(Btrc) substrate Cdc25A is present and, more significantly, Cdc25A is present throughout G2 and M phases in measurable quantities.


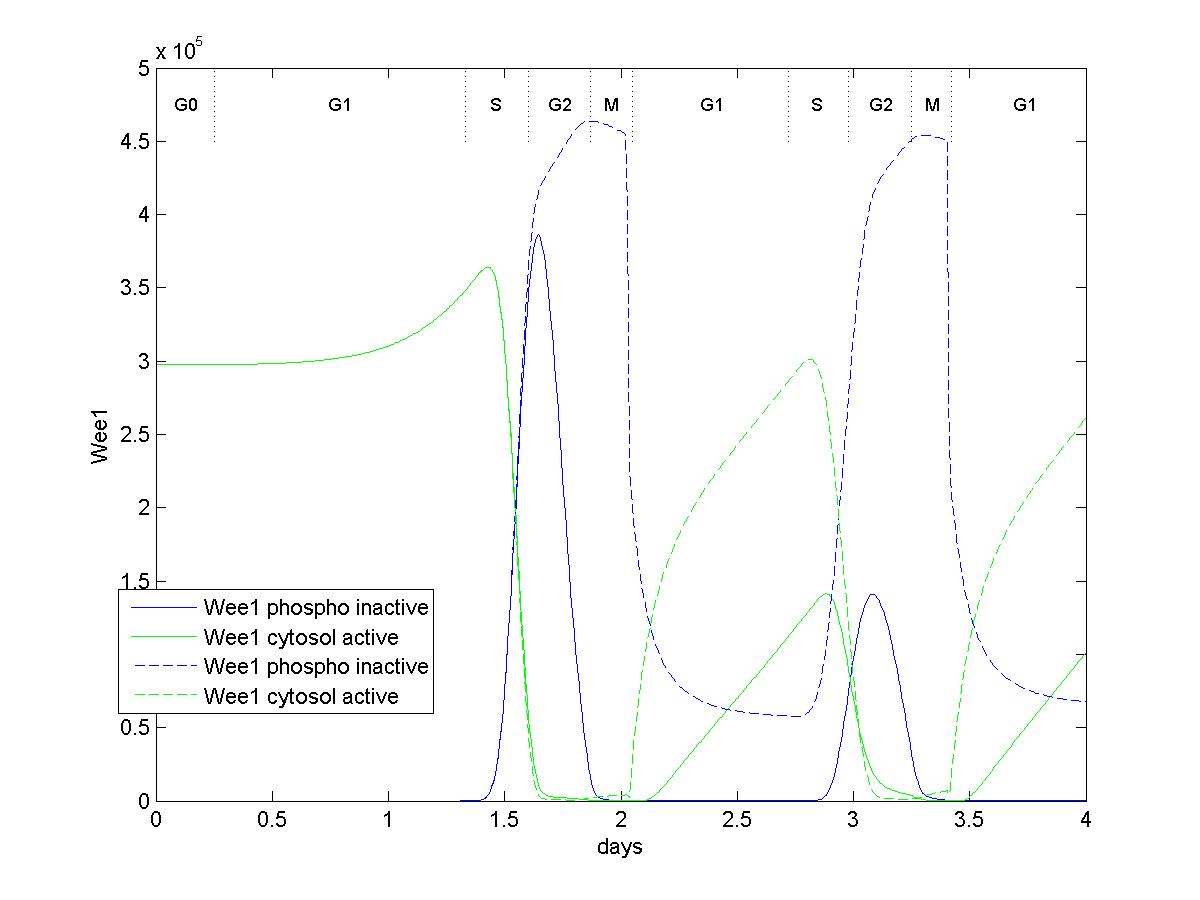
 Figure 4-3. Numbers of Wee1 over two cell divisions. Solid lines represent calculation with SCF(Btrc) (as in Additional file 3, Figure 3-13); dashed lines represent calculation without SCF(Btrc). Inactive Wee1, an SCF(Btrc) substrate, is present in greater numbers and, more significantly, is present in late G2 phase and throughout M phase in measurable quantities.


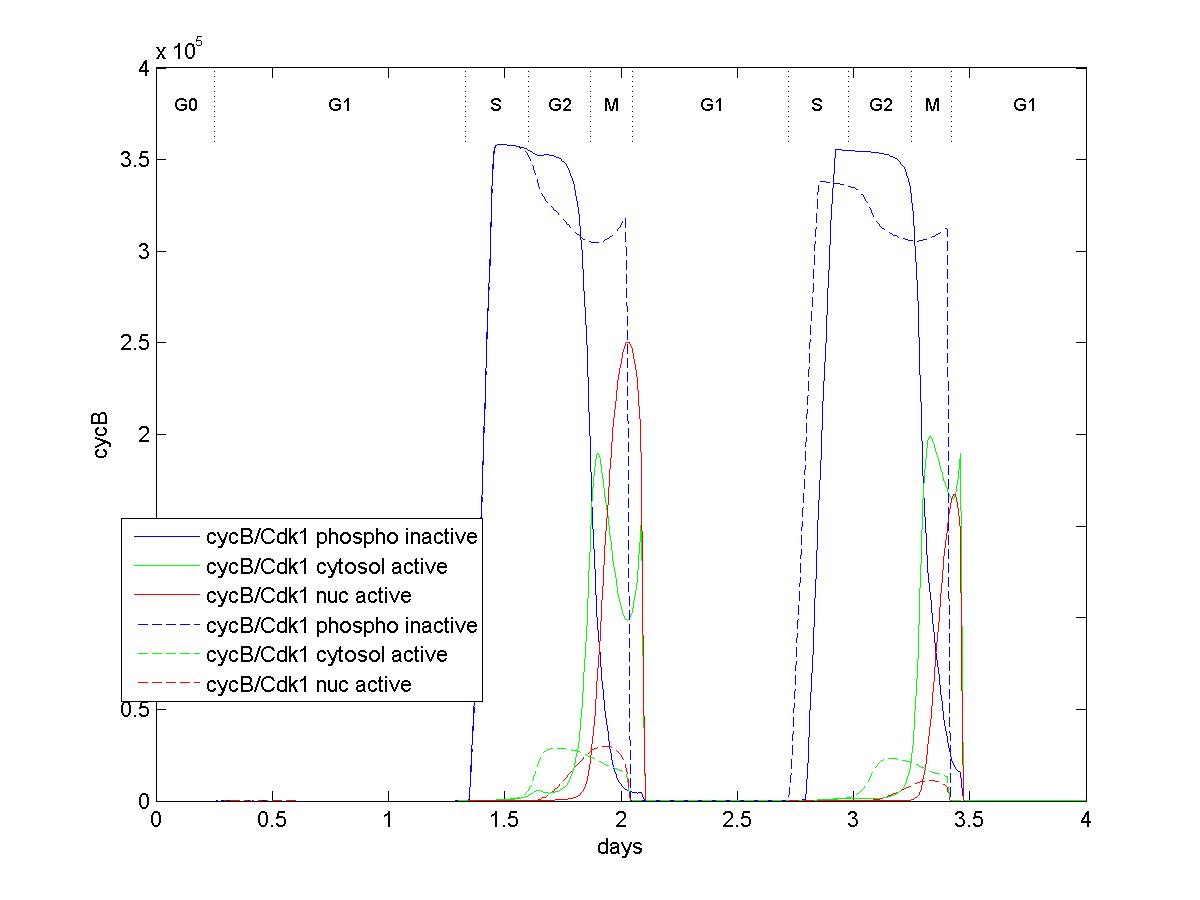
 Figure 4-4. Numbers of cycB/Cdk1 over two cell divisions. Solid lines represent calculation with SCF(Btrc) (as in Additional file 3, Figure 3-11); dashed lines represent calculation without SCF(Btrc). Of significance is the reduced cycB/Cdk1-Cdc25C cascade associated incomplete removal of Wee1.
