# Supplementary material for: A general framework for modeling growth and division of mammalian cells
Source: BMC Syst Biol. 2011 Jan 6;5:3. doi: 10.1186/1752-0509-5-3 (PMC3025838; doi:10.1186/1752-0509-5-3)

**Additional file 5**

**Powersim (http://www.powersim.com)** **Diagrams for the Proteins Included in the Cell-Cycle Model**
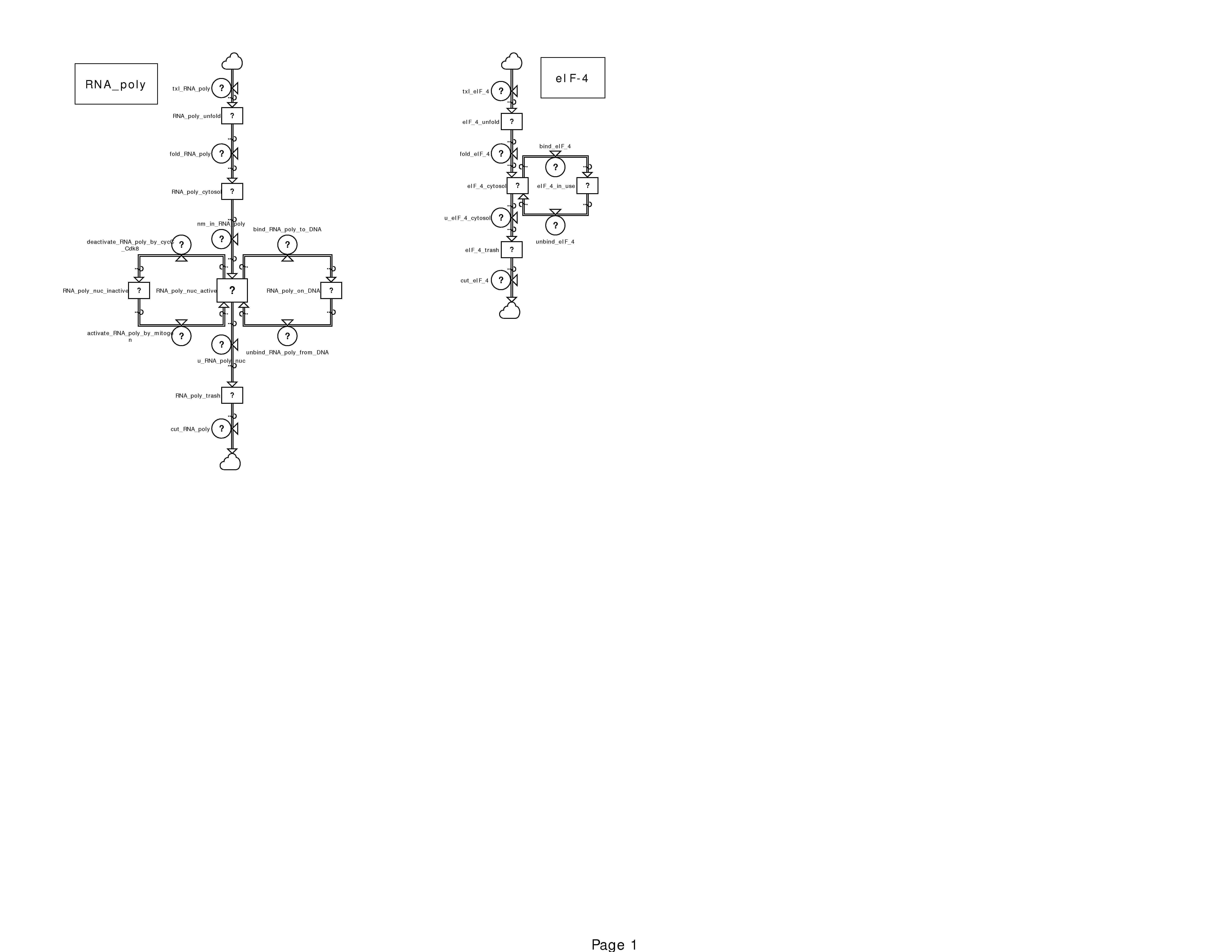


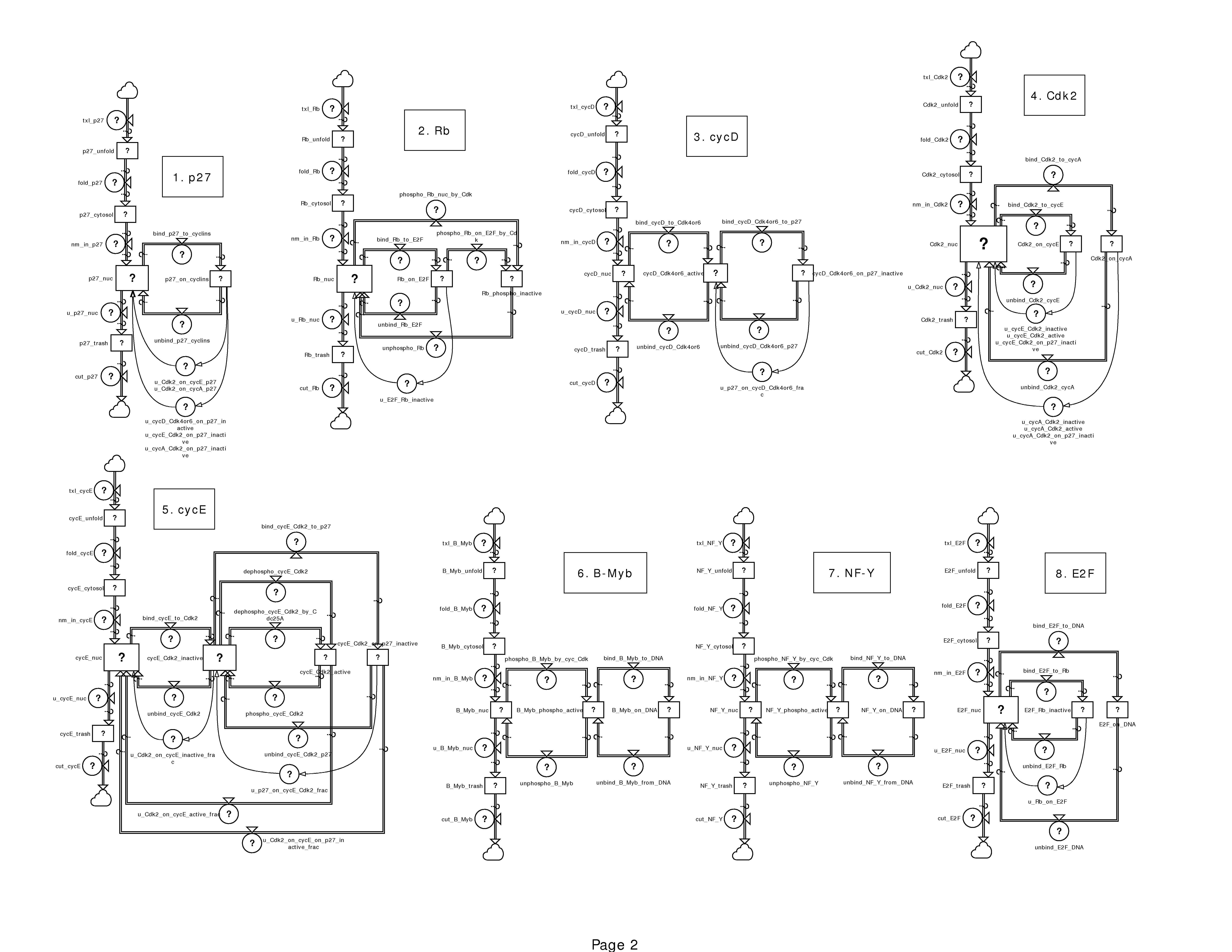

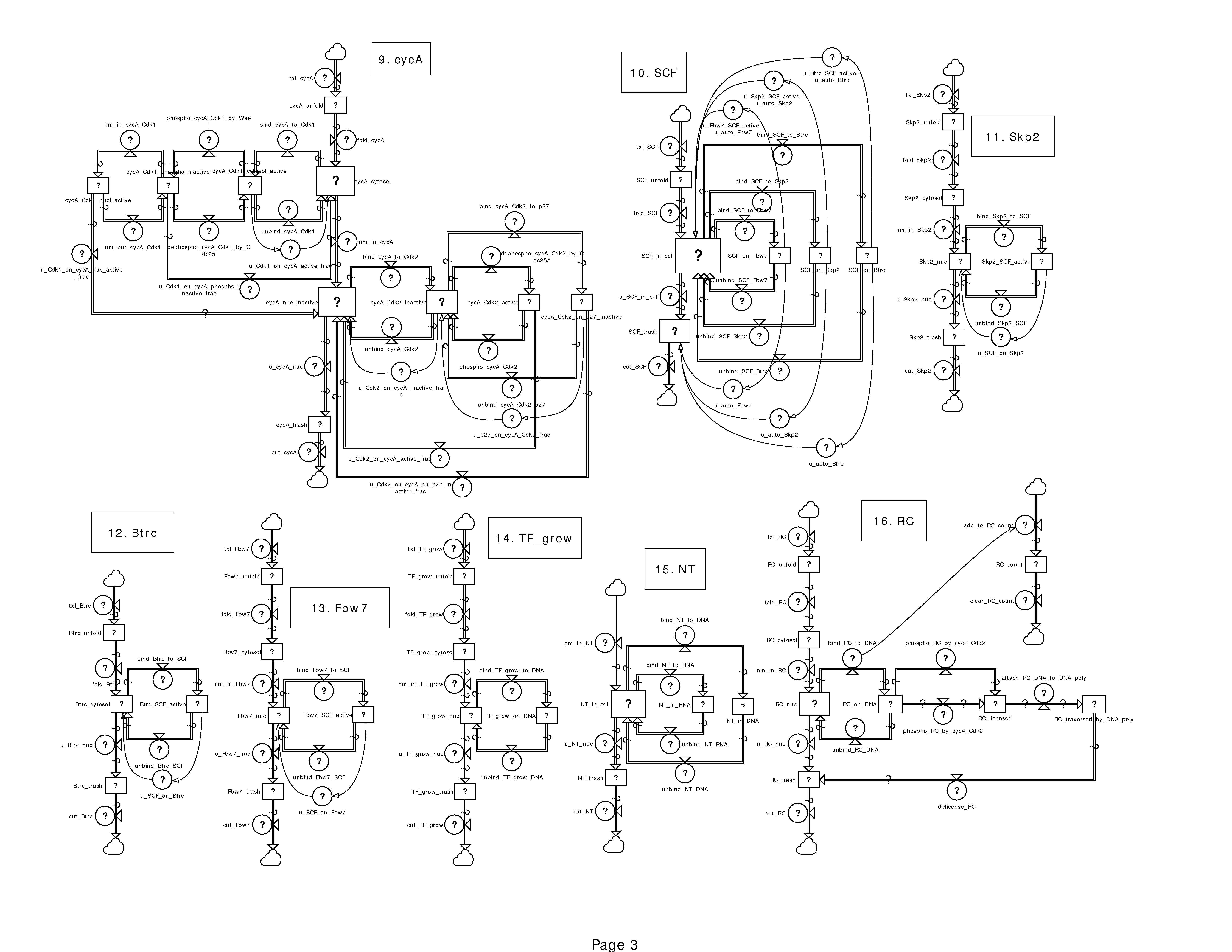

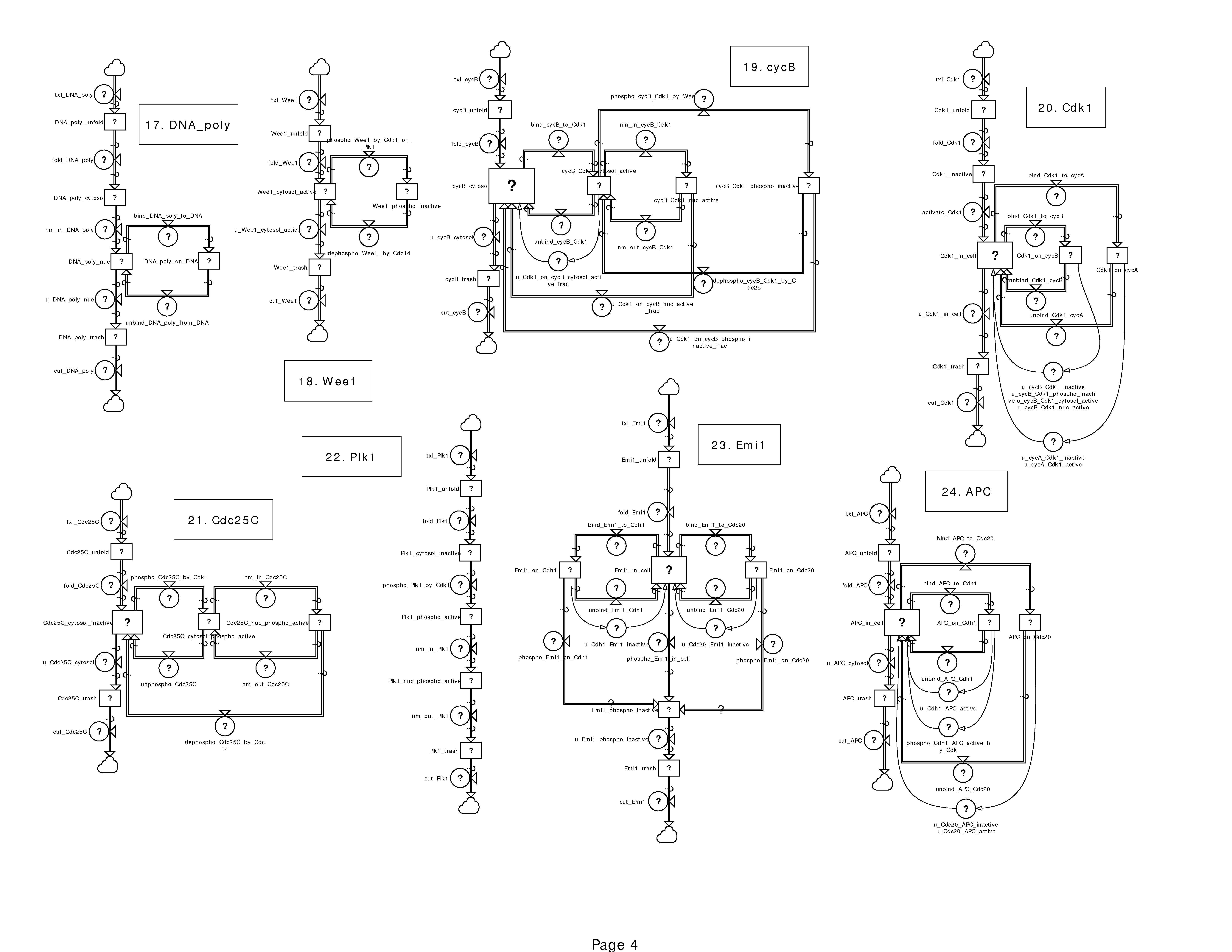

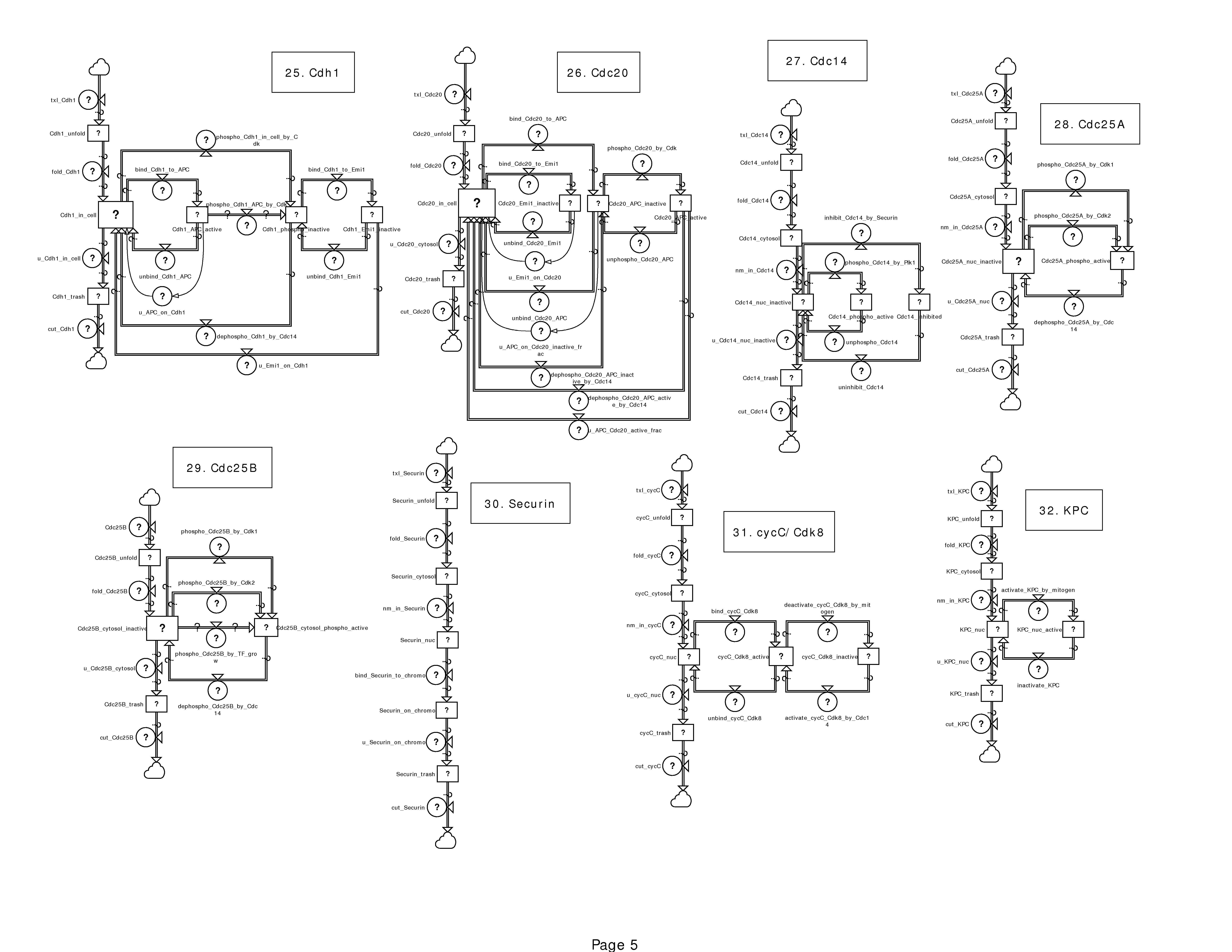

Supplement: Additional file 5 — Powersim http://www.powersim.com Diagrams for the Proteins Included in the Cell-Cycle Model. Lifecycle diagrams of the models used in the cell-cycle model. [file 1752-0509-5-3-S5.DOC]
