# Supplementary material for: A general framework for modeling growth and division of mammalian cells
Source: BMC Syst Biol. 2011 Jan 6;5:3. doi: 10.1186/1752-0509-5-3 (PMC3025838; doi:10.1186/1752-0509-5-3)
Supplement: Additional file 6 — Executing the Cell-Cycle Model and Listing of Matlab Files Used in the Model. Instructions for executing the example model and a listing of the Matlab source. [file 1752-0509-5-3-S6.DOC]

**Additional file 6**

**Executing the Cell-Cycle Model**

The illustrations shown in Figures 1 and 2 in the main text and in Additional file 5 were created with Powersim ([http://www.powersim.com](http://www.powersim.com/)), and Systems Dynamics methods were used in designing the model.  The model, however, is implemented in Matlab ([http://www.mathworks.com](http://www.mathworks.com/)). Matlab is used to take advantage of an ODE solver, ode15s, which is used for systems of stiff equations (see Background in the main text).

The base model and the cell-cycle model are combined in the same system of equations. All model runs are initially solved for steady state, corresponding to cell-cycle phase G0. Steady-state behavior requires that the sum of the input rates into each state should equal the sum of the output rates, and the overall rates of creation (input) of each type of molecule should equal the overall rates of decay (degradation). After steady state is achieved, dynamic behavior (e.g., the cell cycle) is instigated with the introduction of mitogen and adhesion factors at a set time.

The model is executed as follows. Input files are placed in the same directory. Matlab is started and the workspace is pointed to that directory. On the Matlab command line is entered: *cellrun* . The file cellrun.m contains the Matlab commands for executing the model. The files cellsseqns.m and celldyneqns.m (which are almost identical) contain the ODEs. The files getssconst.m and getconst.m contain the constant parameter values. The file getdividing.m contains the logic for determining when the cell is dividing. The other files are used to write the output files and plots (cellssplot.m, cellssrates.m, cellssstates.m, celldynplot.m, celldynrates.m, and celldynstates.m). The result plots in this paper are defined in the file figpaper.m, which can be executed from the Matlab command line after cellrun has terminated. Execution time for a 6-day simulation is approximately 40 minutes on a personal computer.

**Listing of Matlab Files Used in the Model**

The following are the source listings for the files mentioned above. When copied (and modified where specified), a Matlab run can produce the same results as presented in this paper. These files can also be obtained upon request from the corresponding author ([jhgauth@sandia.gov](mailto:jhgauth@sandia.gov)). Most of these files are created and maintained automatically from simpler data files. For more information, contact the corresponding author.

# cellrun.m

%

% cellssrun.m

% run the cell model to steady state

%

starttime=clock;

disp(['starttime=' num2str(starttime(4)) ':' num2str(starttime(5)) ':' num2str(starttime(6)) ])

% !!!!!!!!!!!!!!!!!!!!!!!!!!!!!!!!!!!!!!!!!!!!!!! TIME SPAN

% 1 yr

% tspan=[0:86400:31536000];

% 10 yr

tspan=[0:86400:315360000];

% !!!!!!!!!!!!!!!!!!!!!!!!!!!!!!!!!!!!!!!!!!!!!!!! INITIAL CONDITIONS FOR LEVELS

[z,y0]=getssconst;

% !!!!!!!!!!!!!!!!!!!!!!!!!!!!!!!!!!!!!!!!!!!!!!! RUN ODE SOLVER

[t,y]=ode15s('cellsseqns',tspan,y0);

% !!!!!!!!!!!!!!!!!!!!!!!!!!!!!!!!!!!!!!!!!!!!!!! WRITE OUTPUT FILES

[ylength,ycol]=size(y);

cellssstates(ylength,y);

cellssrates(ylength,y);

cellssplot(t,y);

disp(['ss ylength=' num2str(ylength)])

fid=fopen('steadystate.bin','w');

fwrite(fid,y(ylength,:),'double');

fclose(fid);

% !!!!!!!!!!!!!!!!!!!!!!!!!!!!!!!!!!!!!!!!!!!!!!! WRITE OUT RUNTIME

stoptime=clock;

days=stoptime(3)-starttime(3);

runtime=86400*days+3600*stoptime(4)+60*stoptime(5)+stoptime(6)-(3600*starttime(4)+60*starttime(5)+starttime(6));

hours=floor((runtime-86400*days)/3600);

minutes=floor((runtime-3600*hours-86400*days)/60);

seconds=runtime-60*minutes-3600*hours-86400*days;

disp(['ss runtime sec=' num2str(runtime)])

disp(['ss runtime min=' num2str(minutes) ' sec=' num2str(seconds)])

%

% celldynrun.m

% run the cell model dynamically

%

starttime=clock;

% !!!!!!!!!!!!!!!!!!!!!!!!!!!!!!!!!!!!!!!!!!!!!!!!!!!! TIME SPAN

% 1 day

% tspan=[0:60:86400];

% 10 day

tspan=[0:600:864000];

% !!!!!!!!!!!!!!!!!!!!!!!!!!!!!!!!!!! DYNAMIC INITIAL CONDITIONS

% [z,y0]=getdynconst;

fid=fopen('steadystate.bin','r');

y0=fread(fid,inf,'double');

fclose(fid);

% !!!!!!!!!!!!!!!!!!!!!!!!!!!!!!! INITIALIZE DIVIDING TO NO

idividing = 0;

tprev = 0;

[fid2,message] = fopen('celldiv.dat','w');

if(fid2 == -1)

disp(message)

end

fprintf(fid2,'%f %f',idividing,tprev);

fclose(fid2);

% !!!!!!!!!!!!!!!!!!!!!!!!!!!!!!!!!!!!!!!!!!!!!!! RUN ODE SOLVER

[t,y]=ode15s('celldyneqns',tspan,y0);

% !!!!!!!!!!!!!!!!!!!!!!!!!!!!!!!!!!!!!!!!!!! WRITE OUTPUT FILES

[ylength,ycol]=size(y);

celldynstates(ylength,y);

celldynrates(t(length(t)),ylength,y);

celldynplot(t,y);

disp(['ss ylength=' num2str(ylength)])

% !!!!!!!!!!!!!!!!!!!!!!!!!!!!!!!!!!!!!!!!!!!!!!! WRITE OUT RUNTIME

stoptime=clock;

days=stoptime(3)-starttime(3);

runtime=86400*days+3600*stoptime(4)+60*stoptime(5)+stoptime(6)-(3600*starttime(4)+60*starttime(5)+starttime(6));

% hours=floor(runtime/3600)-86400*days;

% minutes=floor(runtime/60)-3600*hours-86400*days;

% seconds=runtime-60*minutes-3600*hours-86400*days;

hours=floor((runtime-86400*days)/3600);

minutes=floor((runtime-3600*hours-86400*days)/60);

seconds=runtime-60*minutes-3600*hours-86400*days;

disp(['runtime sec=' num2str(runtime)])

disp(['runtime min=' num2str(minutes) ' sec=' num2str(seconds)])

# cellsseqns.m

function ydot=cellsseqns(t,y)

% !!!!!!!!!!!!!!!!!!!!!!!!!!!!!!!!!!!!!!!!!!!! GET CONSTANTS

[z,y0]=getssconst;

% !!!!!!!!!!!!!!!!!!!!!!!!!!!!!!!!!!!!!!!!! UNPACK CONSTANTS

ATP_AA = z(1);

ATP_eIF_4 = z(2);

ATP_fat = z(3);

ATP_fold = z(4);

ATP_glycolysis = z(5);

ATP_glycosolation = z(6);

ATP_H = z(7);

ATP_mem_transfer = z(8);

ATP_pm_transfer = z(9);

ATP_Na = z(10);

ATP_NT = z(11);

ATP_NT_out_nm = z(12);

ATP_polymerization = z(13);

ATP_proteasome = z(14);

ATP_respiration = z(15);

ATP_spl = z(16);

ATP_tRNA = z(17);

ATP_u = z(18);

ATP_vesicle = z(19);

exon_mRNA = z(20);

exon_rRNA = z(21);

exon_snRNA = z(22);

frac_p_cytosol = z(23);

frac_p_ER = z(24);

frac_p_lysosome = z(25);

frac_p_mitochondria = z(26);

frac_p_nuc = z(27);

frac_polyI = z(28);

frac_polyII = z(29);

frac_polyII_snRNA = z(30);

frac_polyIII = z(31);

i_eIF_4 = z(32);

i_ER_p = z(33);

i_ER_RNA = z(34);

i_poly_all = z(35);

x_mRNA = z(36);

x_mRNA_unspl = z(37);

x_p = z(38);

x_rRNA = z(39);

x_rRNA_unspl = z(40);

x_snRNA = z(41);

x_snRNA_unspl = z(42);

x_tRNA = z(43);

x_vesicle = z(44);

k_ATP_synthase = z(45);

k_bind_p = z(46);

k_bind_snRNA = z(47);

k_cut_p = z(48);

k_cut_RNA = z(49);

k_cut_mRNA = z(50);

k_cut_rRNA = z(51);

k_cut_snRNA = z(52);

k_cut_tRNA = z(53);

k_cut_spljunk_mRNA = z(54);

k_cut_spljunk_rRNA = z(55);

k_cut_spljunk_snRNA = z(56);

k_dk_AA = z(57);

k_dk_ADP = z(58);

k_dk_fat = z(59);

k_dk_NT = z(60);

k_dk_p = z(61);

k_dk_mRNA = z(62);

k_dk_rRNA = z(63);

k_dk_snRNA = z(64);

k_dk_tRNA = z(65);

k_bind_rRNA_mRNA = z(66);

k_ER_p_constant = z(67);

k_ER_RNA_constant = z(68);

k_fold_p = z(69);

k_grow_H = z(70);

k_grow_Na = z(71);

k_make_vesicle = z(72);

k_H_pump = z(73);

k_Na_pump_out = z(74);

k_Na_return = z(75);

k_nm_mRNA = z(76);

k_nm_rRNA = z(77);

k_nm_tRNA = z(78);

k_pm_in_AA = z(79);

k_pm_in_ADP = z(80);

k_pm_in_NT = z(81);

k_pm_in_fats = z(82);

k_pm_in_sugar = z(83);

k_restructure = z(84);

k_spl = z(85);

k_tRNA_AA_binding = z(86);

k_unbind_fats = z(87);

k_bind_fats = z(88);

rRNA_spacing_on_mRNA = z(89);

k_bind_p_poly_to_DNA = z(90);

k_unbind_p_poly_from_DNA_by_APC_Cdc20 = z(91);

k_nm_in_p = z(92);

poly_loci_frac = z(93);

RNA_poly_loci_constitutive = z(94);

RNA_poly_active_constitutive = z(95);

k_activate_p_poly_by_mitogen = z(96);

k_deactivate_p_poly_by_cycC_Cdk8 = z(97);

k_activate_mRNA = z(98);

eIF_4_loci_frac = z(99);

p27_loci_frac = z(100);

k_u_p27_by_SCF_Skp2_fast = z(101);

k_u_p27_by_SCF_Skp2_slow = z(102);

k_u_p27_by_APC_Cdc20 = z(103);

k_u_p27_by_KPC = z(104);

Rb_loci_frac = z(105);

k_bind_Rb_to_E2F = z(106);

k_unbind_Rb_E2F = z(107);

k_phospho_Rb_by_Cdk = z(108);

k_unphospho_Rb = z(109);

k_dephospho_Rb_by_Cdc14 = z(110);

mitogen_stimulation = z(111);

cycD_loci_frac = z(112);

cycD_constitutive = z(113);

k_bind_cycD_to_Cdk4or6 = z(114);

k_unbind_cycD_Cdk4or6 = z(115);

k_bind_cycD_Cdk4or6_to_p27 = z(116);

k_unbind_cycD_Cdk4or6_p27 = z(117);

k_u_cycD_by_SCF_Skp2 = z(118);

k_u_cycD_by_APC_Cdc20 = z(119);

Cdk2_loci_frac = z(120);

Cdk2_constituitive = z(121);

cycE_loci_frac = z(122);

k_bind_cycE_to_Cdk2 = z(123);

k_unbind_cycE_Cdk2 = z(124);

k_bind_cycE_Cdk2_to_p27 = z(125);

k_unbind_cycE_Cdk2_p27 = z(126);

k_phospho_cycE_Cdk2 = z(127);

k_dephospho_cycE_Cdk2 = z(128);

k_dephospho_cycE_Cdk2_by_Cdc25A = z(129);

k_u_cycE_by_SCF_Skp2 = z(130);

k_u_cycE_by_SCF_Fbw7 = z(131);

B_Myb_loci_frac = z(132);

k_phospho_B_Myb_by_cyc_Cdk = z(133);

k_unphospho_B_Myb = z(134);

sat_B_Myb_on_DNA = z(135);

k_bind_B_Myb_to_DNA = z(136);

k_unbind_B_Myb_from_DNA = z(137);

k_u_B_Myb_by_SCF_Skp2 = z(138);

NF_Y_loci_frac = z(139);

NF_Y_constitutive = z(140);

k_phospho_NF_Y_by_cyc_Cdk = z(141);

k_unphospho_NF_Y = z(142);

k_dephospho_NF_Y_by_Cdc14 = z(143);

sat_NF_Y_on_DNA = z(144);

k_bind_NF_Y_to_DNA = z(145);

k_unbind_NF_Y_from_DNA = z(146);

E2F_loci_frac = z(147);

E2F_constituitive = z(148);

sat_E2F_on_DNA = z(149);

k_bind_E2F_to_DNA = z(150);

k_unbind_E2F_DNA = z(151);

k_E2F_phospho_by_cycA_Cdk2 = z(152);

k_E2F_phospho_by_cycA_Cdk1 = z(153);

k_E2F_phospho_by_cycB_Cdk1 = z(154);

k_E2F_phospho_by_cycD_Cdk4or6 = z(155);

k_u_E2F_by_SCF_Skp2 = z(156);

k_u_E2F_by_APC_Cdc20 = z(157);

adhesion_factor = z(158);

cycA_loci_frac = z(159);

k_bind_cycA_to_Cdk2 = z(160);

k_unbind_cycA_Cdk2 = z(161);

k_dephospho_cycA_Cdk2_by_Cdc25A = z(162);

k_phospho_cycA_Cdk2 = z(163);

k_bind_cycA_Cdk2_to_p27 = z(164);

k_unbind_cycA_Cdk2_p27 = z(165);

k_bind_cycA_to_Cdk1 = z(166);

k_unbind_cycA_Cdk1 = z(167);

k_phospho_cycA_Cdk1_by_Wee1 = z(168);

k_phospho_cycA_Cdk1_by_phospho_Wee1 = z(169);

k_unphospho_cycA_Cdk1_from_Wee1 = z(170);

k_dephospho_cycA_Cdk1_by_Cdc25A = z(171);

k_dephospho_cycA_Cdk1_by_Cdc25B = z(172);

k_dephospho_cycA_Cdk1_by_Cdc25C = z(173);

k_nm_in_cycA_Cdk1_by_Plk1 = z(174);

k_nm_out_cycA_Cdk1 = z(175);

k_u_cycA_by_SCF_Skp2 = z(176);

k_u_cycA_by_APC_Cdc20 = z(177);

k_u_cycA_by_APC_Cdh1 = z(178);

SCF_loci_frac = z(179);

k_u_SCF_in_cell_by_APC_Cdh1 = z(180);

k_u_SCF_active_by_APC_Cdh1 = z(181);

Skp2_loci_frac = z(182);

k_bind_Skp2_to_SCF = z(183);

k_unbind_Skp2_SCF = z(184);

k_u_Skp2_by_APC_Cdh1 = z(185);

k_u_auto_Skp2 = z(186);

Btrc_loci_frac = z(187);

k_bind_Btrc_to_SCF = z(188);

k_unbind_Btrc_SCF = z(189);

k_u_Btrc_by_APC_Cdh1 = z(190);

k_u_auto_Btrc = z(191);

Fbw7_loci_frac = z(192);

k_bind_Fbw7_to_SCF = z(193);

k_unbind_Fbw7_SCF = z(194);

k_u_auto_Fbw7 = z(195);

TF_grow_loci_frac = z(196);

TF_grow_txs_by_mitogen = z(197);

k_TF_grow_txs_by_Skp2 = z(198);

sat_TF_grow_on_DNA = z(199);

k_bind_TF_grow_to_DNA = z(200);

k_unbind_TF_grow_DNA = z(201);

k_u_TF_grow_by_SCF_Fbw7 = z(202);

k_u_TF_grow_by_SCF_Skp2 = z(203);

ATP_DNA_repl = z(204);

ATP_NT_txs = z(205);

x_DNA = z(206);

i_ER_DNA = z(207);

k_ER_DNA_constant = z(208);

k_div_DNA_factor = z(209);

x_RC = z(210);

x_RC_frag = z(211);

x_NT_RC_frag = z(212);

x_DNA_poly_per_RC = z(213);

RC_loci_frac = z(214);

k_bind_RC_to_DNA = z(215);

k_unbind_RC_DNA = z(216);

k_phospho_RC_by_cycD_Cdk4or6 = z(217);

k_phospho_RC_by_cycD_Cdk4or6_on_p27 = z(218);

k_phospho_RC_by_cycE_Cdk2 = z(219);

k_phospho_RC_by_cycA_Cdk2 = z(220);

k_u_RC_by_SCF_Fbw7 = z(221);

k_u_RC_by_SCF_Skp2 = z(222);

k_u_RC_by_APC_Cdh1 = z(223);

k_clear_RC_count = z(224);

DNA_poly_loci_frac = z(225);

k_bind_DNA_poly_to_DNA = z(226);

sat_DNA_poly = z(227);

Wee1_loci_frac = z(228);

k_phospho_Wee1_by_cycA_Cdk2 = z(229);

k_phospho_Wee1_by_cycA_Cdk1 = z(230);

k_phospho_Wee1_by_cycB_Cdk1 = z(231);

k_phospho_Wee1_by_Plk1 = z(232);

k_unphospho_Wee1 = z(233);

k_dephospho_Wee1_by_Cdc14 = z(234);

k_u_Wee1_by_SCF_Btrc = z(235);

k_u_Wee1_by_SCF_Skp2_test = z(236);

cycB_loci_frac = z(237);

k_bind_cycB_to_Cdk1 = z(238);

k_unbind_cycB_Cdk1 = z(239);

k_phospho_cycB_Cdk1_by_Wee1 = z(240);

k_phospho_cycB_Cdk1_by_phospho_Wee1 = z(241);

k_unphospho_cycB_Cdk1_from_Wee1 = z(242);

k_dephospho_cycB_Cdk1_by_Cdc25A = z(243);

k_dephospho_cycB_Cdk1_by_Cdc25B = z(244);

k_dephospho_cycB_Cdk1_by_Cdc25C = z(245);

k_nm_in_cycB_Cdk1_by_Plk1 = z(246);

k_nm_out_cycB_Cdk1 = z(247);

k_u_cycB_by_APC_Cdc20 = z(248);

k_u_cycB_by_APC_Cdh1 = z(249);

Cdk1_loci_frac = z(250);

Cdk1_constitutive = z(251);

k_activate_Cdk1_by_B_Myb_and_NF_Y = z(252);

k_deactivate_Cdk1 = z(253);

k_dephospho_Cdk1_by_Cdc14 = z(254);

Cdc25C_loci_frac = z(255);

k_phospho_Cdc25C_by_cycA_Cdk1 = z(256);

k_phospho_Cdc25C_by_cycB_Cdk1 = z(257);

k_phospho_Cdc25C_by_Plk1 = z(258);

k_dephospho_Cdc25C = z(259);

k_unphospho_Cdc25C = z(260);

k_nm_in_Cdc25C = z(261);

k_nm_out_Cdc25C = z(262);

k_dephospho_Cdc25C_by_Cdc14 = z(263);

Plk1_loci_frac = z(264);

k_phospho_Plk1_by_cycA_Cdk1 = z(265);

k_phospho_Plk1_by_cycB_Cdk1 = z(266);

k_unphospho_Plk1 = z(267);

k_nm_in_Plk1 = z(268);

k_u_Plk1_by_APC_Cdh1 = z(269);

Emi1_loci_frac = z(270);

k_bind_Emi1_to_Cdh1 = z(271);

k_unbind_Emi1_Cdh1 = z(272);

k_bind_Emi1_to_Cdc20 = z(273);

k_unbind_Emi1_Cdc20 = z(274);

k_phospho_Emi1_by_cycA_Cdk1 = z(275);

k_phospho_Emi1_by_cycB_Cdk1 = z(276);

k_u_Emi1_by_SCF_Btrc = z(277);

k_u_Emi1_by_SCF_Skp2_test = z(278);

APC_loci_frac = z(279);

Cdh1_loci_frac = z(280);

k_phospho_Cdh1_by_cycD_Cdk4or6 = z(281);

k_phospho_Cdh1_by_cycD_Cdk4or6_on_p27 = z(282);

k_phospho_Cdh1_by_cycE_Cdk2 = z(283);

k_phospho_Cdh1_by_cycA_Cdk2 = z(284);

k_phospho_Cdh1_by_Cdk1 = z(285);

k_dephospho_Cdh1_by_Cdc14 = z(286);

k_bind_Cdh1_to_APC = z(287);

k_unbind_Cdh1_APC = z(288);

k_u_auto_Cdh1_APC_active = z(289);

Cdc20_loci_frac = z(290);

k_bind_Cdc20_to_APC = z(291);

k_unbind_Cdc20_APC = z(292);

k_phospho_Cdc20_by_Cdk1 = z(293);

k_unphospho_Cdc20_APC_active = z(294);

k_dephospho_Cdc20_APC_by_Cdc14 = z(295);

k_u_Cdc20_by_APC_Cdh1 = z(296);

Cdc14_loci_frac = z(297);

k_phospho_Cdc14_by_Plk1 = z(298);

k_unphospho_Cdc14 = z(299);

k_inhibit_Cdc14_by_Securin = z(300);

k_uninhibit_Cdc14 = z(301);

Cdc25A_loci_frac = z(302);

Cdc25A_constitutive = z(303);

k_phospho_Cdc25A_by_Cdk2 = z(304);

k_phospho_Cdc25A_by_cycA_Cdk1 = z(305);

k_phospho_Cdc25A_by_cycB_Cdk1 = z(306);

k_dephospho_Cdc25A = z(307);

k_dephospho_Cdc25A_by_Cdc14 = z(308);

k_u_Cdc25A_by_APC_Cdh1 = z(309);

k_u_Cdc25A_by_SCF_Btrc = z(310);

Cdc25B_loci_frac = z(311);

k_phospho_Cdc25B_by_Cdk2 = z(312);

k_phospho_Cdc25B_by_cycA_Cdk1 = z(313);

k_phospho_Cdc25B_by_cycB_Cdk1 = z(314);

k_phospho_Cdc25B_by_TF_grow = z(315);

k_phospho_Cdc25B_by_Plk1 = z(316);

k_dephospho_Cdc25B = z(317);

k_dephospho_Cdc25B_by_Cdc14 = z(318);

k_u_Cdc25B_by_SCF_Btrc = z(319);

k_u_Cdc25B_by_APC_Cdh1 = z(320);

k_u_Cdc25B_by_APC_Cdh1 = z(321);

Securin_loci_frac = z(322);

k_bind_Securin_to_chromo = z(323);

k_u_Securin_by_APC_Cdc20 = z(324);

cycC_loci_frac = z(325);

k_bind_cycC_to_Cdk8 = z(326);

k_unbind_cycC_from_Cdk8 = z(327);

k_deactivate_cycC_Cdk8_by_mitogen = z(328);

k_activate_cycC_Cdk8_by_Cdc14 = z(329);

KPC_loci_frac = z(330);

k_activate_KPC_by_mitogen = z(331);

k_inactivate_KPC = z(332);

% !!!!!!!!!!!!!!!!!!!!!!!!!!!!!!! UNPACK INITIAL CONDITIONS

i_AA_in_cytosol = y0(1);

i_AA_in_p = y0(2);

i_AA_on_tRNA = y0(3);

i_ADP_in_cell = y0(4);

i_ADP_trash = y0(5);

i_ATP_in_cell = y0(6);

i_fats_cytosol = y0(7);

i_fats_in_mem = y0(8);

i_fats_in_vesicles = y0(9);

i_H_between_mito_mems = y0(10);

i_H_in_inner_mito_mems = y0(11);

i_junk_spl = y0(12);

i_junk_spl_mRNA = y0(13);

i_junk_spl_rRNA = y0(14);

i_junk_spl_snRNA = y0(15);

i_mRNA_cytosol = y0(16);

i_mRNA_in_use = y0(17);

i_mRNA_nuc = y0(18);

i_mRNA_inactive = y0(19);

i_mRNA_trash = y0(20);

i_mRNA_unspl = y0(21);

i_Na_in_cell = y0(22);

i_Na_out_cell = y0(23);

i_NT_in_cell = y0(24);

i_NT_in_RNA = y0(25);

i_p_in_cell = y0(26);

i_p_in_use = y0(27);

i_p_trash = y0(28);

i_p_unfold = y0(29);

i_rRNA_cytosol = y0(30);

i_rRNA_in_use = y0(31);

i_rRNA_nuc = y0(32);

i_rRNA_trash = y0(33);

i_rRNA_unspl = y0(34);

i_snRNA_in_use = y0(35);

i_snRNA_nuc = y0(36);

i_snRNA_trash = y0(37);

i_snRNA_unspl = y0(38);

i_sugar_in_cell = y0(39);

i_tRNA_cytosol = y0(40);

i_tRNA_in_use = y0(41);

i_tRNA_nuc = y0(42);

i_tRNA_trash = y0(43);

i_mRNA_poly_unspl = y0(44);

i_mRNA_poly_nuc = y0(45);

i_mRNA_poly_inactive = y0(46);

i_mRNA_poly_cytosol = y0(47);

i_mRNA_poly_in_use = y0(48);

i_mRNA_poly_trash = y0(49);

i_p_poly_unfold = y0(50);

i_p_poly_cytosol = y0(51);

i_p_poly_nuc_inactive = y0(52);

i_p_poly_nuc_active = y0(53);

i_p_poly_in_use = y0(54);

i_p_poly_trash = y0(55);

i_mRNA_eIF_4_unspl = y0(56);

i_mRNA_eIF_4_nuc = y0(57);

i_mRNA_eIF_4_inactive = y0(58);

i_mRNA_eIF_4_cytosol = y0(59);

i_mRNA_eIF_4_in_use = y0(60);

i_mRNA_eIF_4_trash = y0(61);

i_p_eIF_4_unfold = y0(62);

i_p_eIF_4_cytosol = y0(63);

i_p_eIF_4_in_use = y0(64);

i_p_eIF_4_trash = y0(65);

i_mRNA_p27_unspl = y0(66);

i_mRNA_p27_nuc = y0(67);

i_mRNA_p27_inactive = y0(68);

i_mRNA_p27_cytosol = y0(69);

i_mRNA_p27_in_use = y0(70);

i_mRNA_p27_trash = y0(71);

i_p27_unfold = y0(72);

i_p27_cytosol = y0(73);

i_p27_nuc = y0(74);

i_p27_on_cyclins = y0(75);

i_p27_trash = y0(76);

i_mRNA_Rb_unspl = y0(77);

i_mRNA_Rb_nuc = y0(78);

i_mRNA_Rb_inactive = y0(79);

i_mRNA_Rb_cytosol = y0(80);

i_mRNA_Rb_in_use = y0(81);

i_mRNA_Rb_trash = y0(82);

i_Rb_unfold = y0(83);

i_Rb_cytosol = y0(84);

i_Rb_nuc = y0(85);

i_Rb_on_E2F = y0(86);

i_Rb_phospho_inactive = y0(87);

i_Rb_trash = y0(88);

i_mRNA_cycD_unspl = y0(89);

i_mRNA_cycD_nuc = y0(90);

i_mRNA_cycD_inactive = y0(91);

i_mRNA_cycD_cytosol = y0(92);

i_mRNA_cycD_in_use = y0(93);

i_mRNA_cycD_trash = y0(94);

i_cycD_unfold = y0(95);

i_cycD_cytosol = y0(96);

i_cycD_nuc = y0(97);

i_cycD_Cdk4or6_active = y0(98);

i_cycD_Cdk4or6_on_p27_inactive = y0(99);

i_cycD_trash = y0(100);

i_mRNA_Cdk2_unspl = y0(101);

i_mRNA_Cdk2_nuc = y0(102);

i_mRNA_Cdk2_inactive = y0(103);

i_mRNA_Cdk2_cytosol = y0(104);

i_mRNA_Cdk2_in_use = y0(105);

i_mRNA_Cdk2_trash = y0(106);

i_Cdk2_unfold = y0(107);

i_Cdk2_cytosol = y0(108);

i_Cdk2_nuc = y0(109);

i_Cdk2_on_cycE = y0(110);

i_Cdk2_on_cycA = y0(111);

i_Cdk2_trash = y0(112);

i_mRNA_cycE_unspl = y0(113);

i_mRNA_cycE_nuc = y0(114);

i_mRNA_cycE_inactive = y0(115);

i_mRNA_cycE_cytosol = y0(116);

i_mRNA_cycE_in_use = y0(117);

i_mRNA_cycE_trash = y0(118);

i_cycE_unfold = y0(119);

i_cycE_cytosol = y0(120);

i_cycE_nuc = y0(121);

i_cycE_Cdk2_inactive = y0(122);

i_cycE_Cdk2_active = y0(123);

i_cycE_Cdk2_on_p27_inactive = y0(124);

i_cycE_trash = y0(125);

i_mRNA_B_Myb_unspl = y0(126);

i_mRNA_B_Myb_nuc = y0(127);

i_mRNA_B_Myb_inactive = y0(128);

i_mRNA_B_Myb_cytosol = y0(129);

i_mRNA_B_Myb_in_use = y0(130);

i_mRNA_B_Myb_trash = y0(131);

i_B_Myb_unfold = y0(132);

i_B_Myb_cytosol = y0(133);

i_B_Myb_nuc = y0(134);

i_B_Myb_phospho_active = y0(135);

i_B_Myb_on_DNA = y0(136);

i_B_Myb_trash = y0(137);

i_mRNA_NF_Y_unspl = y0(138);

i_mRNA_NF_Y_nuc = y0(139);

i_mRNA_NF_Y_inactive = y0(140);

i_mRNA_NF_Y_cytosol = y0(141);

i_mRNA_NF_Y_in_use = y0(142);

i_mRNA_NF_Y_trash = y0(143);

i_NF_Y_unfold = y0(144);

i_NF_Y_cytosol = y0(145);

i_NF_Y_nuc = y0(146);

i_NF_Y_phospho_active = y0(147);

i_NF_Y_on_DNA = y0(148);

i_NF_Y_trash = y0(149);

i_mRNA_E2F_unspl = y0(150);

i_mRNA_E2F_nuc = y0(151);

i_mRNA_E2F_inactive = y0(152);

i_mRNA_E2F_cytosol = y0(153);

i_mRNA_E2F_in_use = y0(154);

i_mRNA_E2F_trash = y0(155);

i_E2F_unfold = y0(156);

i_E2F_cytosol = y0(157);

i_E2F_nuc = y0(158);

i_E2F_Rb_inactive = y0(159);

i_E2F_on_DNA = y0(160);

i_E2F_trash = y0(161);

i_mRNA_cycA_unspl = y0(162);

i_mRNA_cycA_nuc = y0(163);

i_mRNA_cycA_inactive = y0(164);

i_mRNA_cycA_cytosol = y0(165);

i_mRNA_cycA_in_use = y0(166);

i_mRNA_cycA_trash = y0(167);

i_cycA_unfold = y0(168);

i_cycA_cytosol = y0(169);

i_cycA_nuc = y0(170);

i_cycA_Cdk2_inactive = y0(171);

i_cycA_Cdk2_on_p27_inactive = y0(172);

i_cycA_Cdk2_active = y0(173);

i_cycA_Cdk1_cytosol_active = y0(174);

i_cycA_Cdk1_phospho_inactive = y0(175);

i_cycA_Cdk1_nuc_active = y0(176);

i_cycA_trash = y0(177);

i_mRNA_SCF_unspl = y0(178);

i_mRNA_SCF_nuc = y0(179);

i_mRNA_SCF_inactive = y0(180);

i_mRNA_SCF_cytosol = y0(181);

i_mRNA_SCF_in_use = y0(182);

i_mRNA_SCF_trash = y0(183);

i_SCF_unfold = y0(184);

i_SCF_in_cell = y0(185);

i_SCF_on_Btrc = y0(186);

i_SCF_on_Fbw7 = y0(187);

i_SCF_on_Skp2 = y0(188);

i_SCF_trash = y0(189);

i_mRNA_Skp2_unspl = y0(190);

i_mRNA_Skp2_nuc = y0(191);

i_mRNA_Skp2_inactive = y0(192);

i_mRNA_Skp2_cytosol = y0(193);

i_mRNA_Skp2_in_use = y0(194);

i_mRNA_Skp2_trash = y0(195);

i_Skp2_unfold = y0(196);

i_Skp2_cytosol = y0(197);

i_Skp2_nuc = y0(198);

i_Skp2_SCF_active = y0(199);

i_Skp2_trash = y0(200);

i_mRNA_Btrc_unspl = y0(201);

i_mRNA_Btrc_nuc = y0(202);

i_mRNA_Btrc_inactive = y0(203);

i_mRNA_Btrc_cytosol = y0(204);

i_mRNA_Btrc_in_use = y0(205);

i_mRNA_Btrc_trash = y0(206);

i_Btrc_unfold = y0(207);

i_Btrc_cytosol = y0(208);

i_Btrc_SCF_active = y0(209);

i_Btrc_trash = y0(210);

i_mRNA_Fbw7_unspl = y0(211);

i_mRNA_Fbw7_nuc = y0(212);

i_mRNA_Fbw7_inactive = y0(213);

i_mRNA_Fbw7_cytosol = y0(214);

i_mRNA_Fbw7_in_use = y0(215);

i_mRNA_Fbw7_trash = y0(216);

i_Fbw7_unfold = y0(217);

i_Fbw7_cytosol = y0(218);

i_Fbw7_nuc = y0(219);

i_Fbw7_SCF_active = y0(220);

i_Fbw7_trash = y0(221);

i_mRNA_TF_grow_unspl = y0(222);

i_mRNA_TF_grow_inactive = y0(223);

i_mRNA_TF_grow_cytosol = y0(224);

i_mRNA_TF_grow_nuc = y0(225);

i_mRNA_TF_grow_in_use = y0(226);

i_mRNA_TF_grow_trash = y0(227);

i_TF_grow_unfold = y0(228);

i_TF_grow_cytosol = y0(229);

i_TF_grow_nuc = y0(230);

i_TF_grow_on_DNA = y0(231);

i_TF_grow_trash = y0(232);

i_NT_in_DNA = y0(233);

i_mRNA_RC_unspl = y0(234);

i_mRNA_RC_nuc = y0(235);

i_mRNA_RC_inactive = y0(236);

i_mRNA_RC_cytosol = y0(237);

i_mRNA_RC_in_use = y0(238);

i_mRNA_RC_trash = y0(239);

i_RC_unfold = y0(240);

i_RC_cytosol = y0(241);

i_RC_nuc = y0(242);

i_RC_on_DNA = y0(243);

i_RC_licensed = y0(244);

i_RC_traversed_by_DNA_poly = y0(245);

i_RC_trash = y0(246);

i_RC_count = y0(247);

i_mRNA_DNA_poly_unspl = y0(248);

i_mRNA_DNA_poly_nuc = y0(249);

i_mRNA_DNA_poly_inactive = y0(250);

i_mRNA_DNA_poly_cytosol = y0(251);

i_mRNA_DNA_poly_in_use = y0(252);

i_mRNA_DNA_poly_trash = y0(253);

i_DNA_poly_unfold = y0(254);

i_DNA_poly_cytosol = y0(255);

i_DNA_poly_nuc = y0(256);

i_DNA_poly_on_DNA = y0(257);

i_DNA_poly_trash = y0(258);

i_mRNA_Wee1_unspl = y0(259);

i_mRNA_Wee1_nuc = y0(260);

i_mRNA_Wee1_inactive = y0(261);

i_mRNA_Wee1_cytosol = y0(262);

i_mRNA_Wee1_in_use = y0(263);

i_mRNA_Wee1_trash = y0(264);

i_Wee1_unfold = y0(265);

i_Wee1_cytosol_active = y0(266);

i_Wee1_phospho_inactive = y0(267);

i_Wee1_trash = y0(268);

i_mRNA_cycB_unspl = y0(269);

i_mRNA_cycB_nuc = y0(270);

i_mRNA_cycB_inactive = y0(271);

i_mRNA_cycB_cytosol = y0(272);

i_mRNA_cycB_in_use = y0(273);

i_mRNA_cycB_trash = y0(274);

i_cycB_unfold = y0(275);

i_cycB_cytosol = y0(276);

i_cycB_Cdk1_cytosol_active = y0(277);

i_cycB_Cdk1_phospho_inactive = y0(278);

i_cycB_Cdk1_nuc_active = y0(279);

i_cycB_trash = y0(280);

i_mRNA_Cdk1_unspl = y0(281);

i_mRNA_Cdk1_nuc = y0(282);

i_mRNA_Cdk1_inactive = y0(283);

i_mRNA_Cdk1_cytosol = y0(284);

i_mRNA_Cdk1_in_use = y0(285);

i_mRNA_Cdk1_trash = y0(286);

i_Cdk1_unfold = y0(287);

i_Cdk1_inactive = y0(288);

i_Cdk1_in_cell = y0(289);

i_Cdk1_on_cycB = y0(290);

i_Cdk1_on_cycA = y0(291);

i_Cdk1_trash = y0(292);

i_mRNA_Cdc25C_unspl = y0(293);

i_mRNA_Cdc25C_nuc = y0(294);

i_mRNA_Cdc25C_inactive = y0(295);

i_mRNA_Cdc25C_cytosol = y0(296);

i_mRNA_Cdc25C_in_use = y0(297);

i_mRNA_Cdc25C_trash = y0(298);

i_Cdc25C_unfold = y0(299);

i_Cdc25C_cytosol_inactive = y0(300);

i_Cdc25C_cytosol_phospho_active = y0(301);

i_Cdc25C_nuc_phospho_active = y0(302);

i_Cdc25C_trash = y0(303);

i_mRNA_Plk1_unspl = y0(304);

i_mRNA_Plk1_nuc = y0(305);

i_mRNA_Plk1_inactive = y0(306);

i_mRNA_Plk1_cytosol = y0(307);

i_mRNA_Plk1_in_use = y0(308);

i_mRNA_Plk1_trash = y0(309);

i_Plk1_unfold = y0(310);

i_Plk1_cytosol_inactive = y0(311);

i_Plk1_cytosol_phospho_active = y0(312);

i_Plk1_nuc_phospho_active = y0(313);

i_Plk1_trash = y0(314);

i_mRNA_Emi1_unspl = y0(315);

i_mRNA_Emi1_nuc = y0(316);

i_mRNA_Emi1_inactive = y0(317);

i_mRNA_Emi1_cytosol = y0(318);

i_mRNA_Emi1_in_use = y0(319);

i_mRNA_Emi1_trash = y0(320);

i_Emi1_unfold = y0(321);

i_Emi1_in_cell = y0(322);

i_Emi1_on_Cdh1 = y0(323);

i_Emi1_on_Cdc20 = y0(324);

i_Emi1_phospho_inactive = y0(325);

i_Emi1_trash = y0(326);

i_mRNA_APC_unspl = y0(327);

i_mRNA_APC_nuc = y0(328);

i_mRNA_APC_inactive = y0(329);

i_mRNA_APC_cytosol = y0(330);

i_mRNA_APC_in_use = y0(331);

i_mRNA_APC_trash = y0(332);

i_APC_unfold = y0(333);

i_APC_in_cell = y0(334);

i_APC_on_Cdh1 = y0(335);

i_APC_on_Cdc20 = y0(336);

i_APC_trash = y0(337);

i_mRNA_Cdh1_unspl = y0(338);

i_mRNA_Cdh1_nuc = y0(339);

i_mRNA_Cdh1_inactive = y0(340);

i_mRNA_Cdh1_cytosol = y0(341);

i_mRNA_Cdh1_in_use = y0(342);

i_mRNA_Cdh1_trash = y0(343);

i_Cdh1_unfold = y0(344);

i_Cdh1_in_cell = y0(345);

i_Cdh1_Emi1_inactive = y0(346);

i_Cdh1_phospho_inactive = y0(347);

i_Cdh1_APC_active = y0(348);

i_Cdh1_trash = y0(349);

i_mRNA_Cdc20_unspl = y0(350);

i_mRNA_Cdc20_nuc = y0(351);

i_mRNA_Cdc20_inactive = y0(352);

i_mRNA_Cdc20_cytosol = y0(353);

i_mRNA_Cdc20_in_use = y0(354);

i_mRNA_Cdc20_trash = y0(355);

i_Cdc20_unfold = y0(356);

i_Cdc20_cytosol = y0(357);

i_Cdc20_nuc = y0(358);

i_Cdc20_Emi1_inactive = y0(359);

i_Cdc20_APC_inactive = y0(360);

i_Cdc20_APC_active = y0(361);

i_Cdc20_trash = y0(362);

i_mRNA_Cdc14_unspl = y0(363);

i_mRNA_Cdc14_nuc = y0(364);

i_mRNA_Cdc14_inactive = y0(365);

i_mRNA_Cdc14_cytosol = y0(366);

i_mRNA_Cdc14_in_use = y0(367);

i_mRNA_Cdc14_trash = y0(368);

i_Cdc14_unfold = y0(369);

i_Cdc14_cytosol = y0(370);

i_Cdc14_nuc_inactive = y0(371);

i_Cdc14_inhibited = y0(372);

i_Cdc14_phospho_active = y0(373);

i_Cdc14_trash = y0(374);

i_mRNA_Cdc25A_unspl = y0(375);

i_mRNA_Cdc25A_nuc = y0(376);

i_mRNA_Cdc25A_inactive = y0(377);

i_mRNA_Cdc25A_cytosol = y0(378);

i_mRNA_Cdc25A_in_use = y0(379);

i_mRNA_Cdc25A_trash = y0(380);

i_Cdc25A_unfold = y0(381);

i_Cdc25A_cytosol = y0(382);

i_Cdc25A_nuc_inactive = y0(383);

i_Cdc25A_phospho_active = y0(384);

i_Cdc25A_trash = y0(385);

i_mRNA_Cdc25B_unspl = y0(386);

i_mRNA_Cdc25B_nuc = y0(387);

i_mRNA_Cdc25B_inactive = y0(388);

i_mRNA_Cdc25B_cytosol = y0(389);

i_mRNA_Cdc25B_in_use = y0(390);

i_mRNA_Cdc25B_trash = y0(391);

i_Cdc25B_unfold = y0(392);

i_Cdc25B_cytosol_inactive = y0(393);

i_Cdc25B_cytosol_phospho_active = y0(394);

i_Cdc25B_trash = y0(395);

i_mRNA_Securin_unspl = y0(396);

i_mRNA_Securin_nuc = y0(397);

i_mRNA_Securin_inactive = y0(398);

i_mRNA_Securin_cytosol = y0(399);

i_mRNA_Securin_in_use = y0(400);

i_mRNA_Securin_trash = y0(401);

i_Securin_unfold = y0(402);

i_Securin_cytosol = y0(403);

i_Securin_nuc = y0(404);

i_Securin_on_chromo = y0(405);

i_Securin_trash = y0(406);

i_mRNA_cycC_unspl = y0(407);

i_mRNA_cycC_nuc = y0(408);

i_mRNA_cycC_inactive = y0(409);

i_mRNA_cycC_cytosol = y0(410);

i_mRNA_cycC_in_use = y0(411);

i_mRNA_cycC_trash = y0(412);

i_cycC_unfold = y0(413);

i_cycC_cytosol = y0(414);

i_cycC_nuc = y0(415);

i_cycC_Cdk8_active = y0(416);

i_cycC_Cdk8_inactive = y0(417);

i_cycC_trash = y0(418);

i_mRNA_KPC_unspl = y0(419);

i_mRNA_KPC_nuc = y0(420);

i_mRNA_KPC_inactive = y0(421);

i_mRNA_KPC_cytosol = y0(422);

i_mRNA_KPC_in_use = y0(423);

i_mRNA_KPC_trash = y0(424);

i_KPC_unfold = y0(425);

i_KPC_in_cell = y0(426);

i_KPC_active = y0(427);

i_KPC_trash = y0(428);

% !!!!!!!!!!!!!!!!!!!!!!!!!!!!!!!!!!!!!!!!!! INITIALIZATION

AA_in_cytosol = y(1);

AA_in_p = y(2);

AA_on_tRNA = y(3);

ADP_in_cell = y(4);

ADP_trash = y(5);

ATP_in_cell = y(6);

fats_cytosol = y(7);

fats_in_mem = y(8);

fats_in_vesicles = y(9);

H_between_mito_mems = y(10);

H_in_inner_mito_mems = y(11);

junk_spl = y(12);

junk_spl_mRNA = y(13);

junk_spl_rRNA = y(14);

junk_spl_snRNA = y(15);

mRNA_cytosol = y(16);

mRNA_in_use = y(17);

mRNA_nuc = y(18);

mRNA_inactive = y(19);

mRNA_trash = y(20);

mRNA_unspl = y(21);

Na_in_cell = y(22);

Na_out_cell = y(23);

NT_in_cell = y(24);

NT_in_RNA = y(25);

p_in_cell = y(26);

p_in_use = y(27);

p_trash = y(28);

p_unfold = y(29);

rRNA_cytosol = y(30);

rRNA_in_use = y(31);

rRNA_nuc = y(32);

rRNA_trash = y(33);

rRNA_unspl = y(34);

snRNA_in_use = y(35);

snRNA_nuc = y(36);

snRNA_trash = y(37);

snRNA_unspl = y(38);

sugar_in_cell = y(39);

tRNA_cytosol = y(40);

tRNA_in_use = y(41);

tRNA_nuc = y(42);

tRNA_trash = y(43);

mRNA_poly_unspl = y(44);

mRNA_poly_nuc = y(45);

mRNA_poly_inactive = y(46);

mRNA_poly_cytosol = y(47);

mRNA_poly_in_use = y(48);

mRNA_poly_trash = y(49);

p_poly_unfold = y(50);

p_poly_cytosol = y(51);

p_poly_nuc_inactive = y(52);

p_poly_nuc_active = y(53);

p_poly_in_use = y(54);

p_poly_trash = y(55);

mRNA_eIF_4_unspl = y(56);

mRNA_eIF_4_nuc = y(57);

mRNA_eIF_4_inactive = y(58);

mRNA_eIF_4_cytosol = y(59);

mRNA_eIF_4_in_use = y(60);

mRNA_eIF_4_trash = y(61);

p_eIF_4_unfold = y(62);

p_eIF_4_cytosol = y(63);

p_eIF_4_in_use = y(64);

p_eIF_4_trash = y(65);

mRNA_p27_unspl = y(66);

mRNA_p27_nuc = y(67);

mRNA_p27_inactive = y(68);

mRNA_p27_cytosol = y(69);

mRNA_p27_in_use = y(70);

mRNA_p27_trash = y(71);

p27_unfold = y(72);

p27_cytosol = y(73);

p27_nuc = y(74);

p27_on_cyclins = y(75);

p27_trash = y(76);

mRNA_Rb_unspl = y(77);

mRNA_Rb_nuc = y(78);

mRNA_Rb_inactive = y(79);

mRNA_Rb_cytosol = y(80);

mRNA_Rb_in_use = y(81);

mRNA_Rb_trash = y(82);

Rb_unfold = y(83);

Rb_cytosol = y(84);

Rb_nuc = y(85);

Rb_on_E2F = y(86);

Rb_phospho_inactive = y(87);

Rb_trash = y(88);

mRNA_cycD_unspl = y(89);

mRNA_cycD_nuc = y(90);

mRNA_cycD_inactive = y(91);

mRNA_cycD_cytosol = y(92);

mRNA_cycD_in_use = y(93);

mRNA_cycD_trash = y(94);

cycD_unfold = y(95);

cycD_cytosol = y(96);

cycD_nuc = y(97);

cycD_Cdk4or6_active = y(98);

cycD_Cdk4or6_on_p27_inactive = y(99);

cycD_trash = y(100);

mRNA_Cdk2_unspl = y(101);

mRNA_Cdk2_nuc = y(102);

mRNA_Cdk2_inactive = y(103);

mRNA_Cdk2_cytosol = y(104);

mRNA_Cdk2_in_use = y(105);

mRNA_Cdk2_trash = y(106);

Cdk2_unfold = y(107);

Cdk2_cytosol = y(108);

Cdk2_nuc = y(109);

Cdk2_on_cycE = y(110);

Cdk2_on_cycA = y(111);

Cdk2_trash = y(112);

mRNA_cycE_unspl = y(113);

mRNA_cycE_nuc = y(114);

mRNA_cycE_inactive = y(115);

mRNA_cycE_cytosol = y(116);

mRNA_cycE_in_use = y(117);

mRNA_cycE_trash = y(118);

cycE_unfold = y(119);

cycE_cytosol = y(120);

cycE_nuc = y(121);

cycE_Cdk2_inactive = y(122);

cycE_Cdk2_active = y(123);

cycE_Cdk2_on_p27_inactive = y(124);

cycE_trash = y(125);

mRNA_B_Myb_unspl = y(126);

mRNA_B_Myb_nuc = y(127);

mRNA_B_Myb_inactive = y(128);

mRNA_B_Myb_cytosol = y(129);

mRNA_B_Myb_in_use = y(130);

mRNA_B_Myb_trash = y(131);

B_Myb_unfold = y(132);

B_Myb_cytosol = y(133);

B_Myb_nuc = y(134);

B_Myb_phospho_active = y(135);

B_Myb_on_DNA = y(136);

B_Myb_trash = y(137);

mRNA_NF_Y_unspl = y(138);

mRNA_NF_Y_nuc = y(139);

mRNA_NF_Y_inactive = y(140);

mRNA_NF_Y_cytosol = y(141);

mRNA_NF_Y_in_use = y(142);

mRNA_NF_Y_trash = y(143);

NF_Y_unfold = y(144);

NF_Y_cytosol = y(145);

NF_Y_nuc = y(146);

NF_Y_phospho_active = y(147);

NF_Y_on_DNA = y(148);

NF_Y_trash = y(149);

mRNA_E2F_unspl = y(150);

mRNA_E2F_nuc = y(151);

mRNA_E2F_inactive = y(152);

mRNA_E2F_cytosol = y(153);

mRNA_E2F_in_use = y(154);

mRNA_E2F_trash = y(155);

E2F_unfold = y(156);

E2F_cytosol = y(157);

E2F_nuc = y(158);

E2F_Rb_inactive = y(159);

E2F_on_DNA = y(160);

E2F_trash = y(161);

mRNA_cycA_unspl = y(162);

mRNA_cycA_nuc = y(163);

mRNA_cycA_inactive = y(164);

mRNA_cycA_cytosol = y(165);

mRNA_cycA_in_use = y(166);

mRNA_cycA_trash = y(167);

cycA_unfold = y(168);

cycA_cytosol = y(169);

cycA_nuc = y(170);

cycA_Cdk2_inactive = y(171);

cycA_Cdk2_on_p27_inactive = y(172);

cycA_Cdk2_active = y(173);

cycA_Cdk1_cytosol_active = y(174);

cycA_Cdk1_phospho_inactive = y(175);

cycA_Cdk1_nuc_active = y(176);

cycA_trash = y(177);

mRNA_SCF_unspl = y(178);

mRNA_SCF_nuc = y(179);

mRNA_SCF_inactive = y(180);

mRNA_SCF_cytosol = y(181);

mRNA_SCF_in_use = y(182);

mRNA_SCF_trash = y(183);

SCF_unfold = y(184);

SCF_in_cell = y(185);

SCF_on_Btrc = y(186);

SCF_on_Fbw7 = y(187);

SCF_on_Skp2 = y(188);

SCF_trash = y(189);

mRNA_Skp2_unspl = y(190);

mRNA_Skp2_nuc = y(191);

mRNA_Skp2_inactive = y(192);

mRNA_Skp2_cytosol = y(193);

mRNA_Skp2_in_use = y(194);

mRNA_Skp2_trash = y(195);

Skp2_unfold = y(196);

Skp2_cytosol = y(197);

Skp2_nuc = y(198);

Skp2_SCF_active = y(199);

Skp2_trash = y(200);

mRNA_Btrc_unspl = y(201);

mRNA_Btrc_nuc = y(202);

mRNA_Btrc_inactive = y(203);

mRNA_Btrc_cytosol = y(204);

mRNA_Btrc_in_use = y(205);

mRNA_Btrc_trash = y(206);

Btrc_unfold = y(207);

Btrc_cytosol = y(208);

Btrc_SCF_active = y(209);

Btrc_trash = y(210);

mRNA_Fbw7_unspl = y(211);

mRNA_Fbw7_nuc = y(212);

mRNA_Fbw7_inactive = y(213);

mRNA_Fbw7_cytosol = y(214);

mRNA_Fbw7_in_use = y(215);

mRNA_Fbw7_trash = y(216);

Fbw7_unfold = y(217);

Fbw7_cytosol = y(218);

Fbw7_nuc = y(219);

Fbw7_SCF_active = y(220);

Fbw7_trash = y(221);

mRNA_TF_grow_unspl = y(222);

mRNA_TF_grow_inactive = y(223);

mRNA_TF_grow_cytosol = y(224);

mRNA_TF_grow_nuc = y(225);

mRNA_TF_grow_in_use = y(226);

mRNA_TF_grow_trash = y(227);

TF_grow_unfold = y(228);

TF_grow_cytosol = y(229);

TF_grow_nuc = y(230);

TF_grow_on_DNA = y(231);

TF_grow_trash = y(232);

NT_in_DNA = y(233);

mRNA_RC_unspl = y(234);

mRNA_RC_nuc = y(235);

mRNA_RC_inactive = y(236);

mRNA_RC_cytosol = y(237);

mRNA_RC_in_use = y(238);

mRNA_RC_trash = y(239);

RC_unfold = y(240);

RC_cytosol = y(241);

RC_nuc = y(242);

RC_on_DNA = y(243);

RC_licensed = y(244);

RC_traversed_by_DNA_poly = y(245);

RC_trash = y(246);

RC_count = y(247);

mRNA_DNA_poly_unspl = y(248);

mRNA_DNA_poly_nuc = y(249);

mRNA_DNA_poly_inactive = y(250);

mRNA_DNA_poly_cytosol = y(251);

mRNA_DNA_poly_in_use = y(252);

mRNA_DNA_poly_trash = y(253);

DNA_poly_unfold = y(254);

DNA_poly_cytosol = y(255);

DNA_poly_nuc = y(256);

DNA_poly_on_DNA = y(257);

DNA_poly_trash = y(258);

mRNA_Wee1_unspl = y(259);

mRNA_Wee1_nuc = y(260);

mRNA_Wee1_inactive = y(261);

mRNA_Wee1_cytosol = y(262);

mRNA_Wee1_in_use = y(263);

mRNA_Wee1_trash = y(264);

Wee1_unfold = y(265);

Wee1_cytosol_active = y(266);

Wee1_phospho_inactive = y(267);

Wee1_trash = y(268);

mRNA_cycB_unspl = y(269);

mRNA_cycB_nuc = y(270);

mRNA_cycB_inactive = y(271);

mRNA_cycB_cytosol = y(272);

mRNA_cycB_in_use = y(273);

mRNA_cycB_trash = y(274);

cycB_unfold = y(275);

cycB_cytosol = y(276);

cycB_Cdk1_cytosol_active = y(277);

cycB_Cdk1_phospho_inactive = y(278);

cycB_Cdk1_nuc_active = y(279);

cycB_trash = y(280);

mRNA_Cdk1_unspl = y(281);

mRNA_Cdk1_nuc = y(282);

mRNA_Cdk1_inactive = y(283);

mRNA_Cdk1_cytosol = y(284);

mRNA_Cdk1_in_use = y(285);

mRNA_Cdk1_trash = y(286);

Cdk1_unfold = y(287);

Cdk1_inactive = y(288);

Cdk1_in_cell = y(289);

Cdk1_on_cycB = y(290);

Cdk1_on_cycA = y(291);

Cdk1_trash = y(292);

mRNA_Cdc25C_unspl = y(293);

mRNA_Cdc25C_nuc = y(294);

mRNA_Cdc25C_inactive = y(295);

mRNA_Cdc25C_cytosol = y(296);

mRNA_Cdc25C_in_use = y(297);

mRNA_Cdc25C_trash = y(298);

Cdc25C_unfold = y(299);

Cdc25C_cytosol_inactive = y(300);

Cdc25C_cytosol_phospho_active = y(301);

Cdc25C_nuc_phospho_active = y(302);

Cdc25C_trash = y(303);

mRNA_Plk1_unspl = y(304);

mRNA_Plk1_nuc = y(305);

mRNA_Plk1_inactive = y(306);

mRNA_Plk1_cytosol = y(307);

mRNA_Plk1_in_use = y(308);

mRNA_Plk1_trash = y(309);

Plk1_unfold = y(310);

Plk1_cytosol_inactive = y(311);

Plk1_cytosol_phospho_active = y(312);

Plk1_nuc_phospho_active = y(313);

Plk1_trash = y(314);

mRNA_Emi1_unspl = y(315);

mRNA_Emi1_nuc = y(316);

mRNA_Emi1_inactive = y(317);

mRNA_Emi1_cytosol = y(318);

mRNA_Emi1_in_use = y(319);

mRNA_Emi1_trash = y(320);

Emi1_unfold = y(321);

Emi1_in_cell = y(322);

Emi1_on_Cdh1 = y(323);

Emi1_on_Cdc20 = y(324);

Emi1_phospho_inactive = y(325);

Emi1_trash = y(326);

mRNA_APC_unspl = y(327);

mRNA_APC_nuc = y(328);

mRNA_APC_inactive = y(329);

mRNA_APC_cytosol = y(330);

mRNA_APC_in_use = y(331);

mRNA_APC_trash = y(332);

APC_unfold = y(333);

APC_in_cell = y(334);

APC_on_Cdh1 = y(335);

APC_on_Cdc20 = y(336);

APC_trash = y(337);

mRNA_Cdh1_unspl = y(338);

mRNA_Cdh1_nuc = y(339);

mRNA_Cdh1_inactive = y(340);

mRNA_Cdh1_cytosol = y(341);

mRNA_Cdh1_in_use = y(342);

mRNA_Cdh1_trash = y(343);

Cdh1_unfold = y(344);

Cdh1_in_cell = y(345);

Cdh1_Emi1_inactive = y(346);

Cdh1_phospho_inactive = y(347);

Cdh1_APC_active = y(348);

Cdh1_trash = y(349);

mRNA_Cdc20_unspl = y(350);

mRNA_Cdc20_nuc = y(351);

mRNA_Cdc20_inactive = y(352);

mRNA_Cdc20_cytosol = y(353);

mRNA_Cdc20_in_use = y(354);

mRNA_Cdc20_trash = y(355);

Cdc20_unfold = y(356);

Cdc20_cytosol = y(357);

Cdc20_nuc = y(358);

Cdc20_Emi1_inactive = y(359);

Cdc20_APC_inactive = y(360);

Cdc20_APC_active = y(361);

Cdc20_trash = y(362);

mRNA_Cdc14_unspl = y(363);

mRNA_Cdc14_nuc = y(364);

mRNA_Cdc14_inactive = y(365);

mRNA_Cdc14_cytosol = y(366);

mRNA_Cdc14_in_use = y(367);

mRNA_Cdc14_trash = y(368);

Cdc14_unfold = y(369);

Cdc14_cytosol = y(370);

Cdc14_nuc_inactive = y(371);

Cdc14_inhibited = y(372);

Cdc14_phospho_active = y(373);

Cdc14_trash = y(374);

mRNA_Cdc25A_unspl = y(375);

mRNA_Cdc25A_nuc = y(376);

mRNA_Cdc25A_inactive = y(377);

mRNA_Cdc25A_cytosol = y(378);

mRNA_Cdc25A_in_use = y(379);

mRNA_Cdc25A_trash = y(380);

Cdc25A_unfold = y(381);

Cdc25A_cytosol = y(382);

Cdc25A_nuc_inactive = y(383);

Cdc25A_phospho_active = y(384);

Cdc25A_trash = y(385);

mRNA_Cdc25B_unspl = y(386);

mRNA_Cdc25B_nuc = y(387);

mRNA_Cdc25B_inactive = y(388);

mRNA_Cdc25B_cytosol = y(389);

mRNA_Cdc25B_in_use = y(390);

mRNA_Cdc25B_trash = y(391);

Cdc25B_unfold = y(392);

Cdc25B_cytosol_inactive = y(393);

Cdc25B_cytosol_phospho_active = y(394);

Cdc25B_trash = y(395);

mRNA_Securin_unspl = y(396);

mRNA_Securin_nuc = y(397);

mRNA_Securin_inactive = y(398);

mRNA_Securin_cytosol = y(399);

mRNA_Securin_in_use = y(400);

mRNA_Securin_trash = y(401);

Securin_unfold = y(402);

Securin_cytosol = y(403);

Securin_nuc = y(404);

Securin_on_chromo = y(405);

Securin_trash = y(406);

mRNA_cycC_unspl = y(407);

mRNA_cycC_nuc = y(408);

mRNA_cycC_inactive = y(409);

mRNA_cycC_cytosol = y(410);

mRNA_cycC_in_use = y(411);

mRNA_cycC_trash = y(412);

cycC_unfold = y(413);

cycC_cytosol = y(414);

cycC_nuc = y(415);

cycC_Cdk8_active = y(416);

cycC_Cdk8_inactive = y(417);

cycC_trash = y(418);

mRNA_KPC_unspl = y(419);

mRNA_KPC_nuc = y(420);

mRNA_KPC_inactive = y(421);

mRNA_KPC_cytosol = y(422);

mRNA_KPC_in_use = y(423);

mRNA_KPC_trash = y(424);

KPC_unfold = y(425);

KPC_in_cell = y(426);

KPC_active = y(427);

KPC_trash = y(428);

% !!!!!!!!!!!!!!!!!!!!!!!!!!!!!!!!! STEADY STATE VS DYNAMIC

% steady state

size_ratio = 1;

dividing = 0;

k_div = 0;

% dynamics

% size_ratio = p_in_use/i_p_in_use;

% [tprev,dividing,k_div] = getdividing...

% (t,bind_Cdh1_to_APC,unbind_Cdh1_APC,...

% u_Cdh1_APC_active,i_NT_in_DNA,NT_in_DNA);

% if(dividing == 1)

% pm_in_NT = 0;

% end

% if(t>2.0E4)

% mitogen_stimulation = 1;

% adhesion_factor = 1;

% end

% !!!!!!!!!!!!!!!!!!!!!!!!!!!!!!!!!!!!! CONCENTRATION FUDGE

AA_in_cytosol_conc = AA_in_cytosol/size_ratio;

AA_in_p_conc = AA_in_p/size_ratio;

AA_on_tRNA_conc = AA_on_tRNA/size_ratio;

ADP_in_cell_conc = ADP_in_cell/size_ratio;

ADP_trash_conc = ADP_trash/size_ratio;

ATP_in_cell_conc = ATP_in_cell/size_ratio;

fats_cytosol_conc = fats_cytosol/size_ratio;

fats_in_mem_conc = fats_in_mem/size_ratio;

fats_in_vesicles_conc = fats_in_vesicles/size_ratio;

H_between_mito_mems_conc = H_between_mito_mems/size_ratio;

H_in_inner_mito_mems_conc = H_in_inner_mito_mems/size_ratio;

junk_spl_conc = junk_spl/size_ratio;

junk_spl_mRNA_conc = junk_spl_mRNA/size_ratio;

junk_spl_rRNA_conc = junk_spl_rRNA/size_ratio;

junk_spl_snRNA_conc = junk_spl_snRNA/size_ratio;

mRNA_cytosol_conc = mRNA_cytosol/size_ratio;

mRNA_in_use_conc = mRNA_in_use/size_ratio;

mRNA_nuc_conc = mRNA_nuc/size_ratio;

mRNA_inactive_conc = mRNA_inactive/size_ratio;

mRNA_trash_conc = mRNA_trash/size_ratio;

mRNA_unspl_conc = mRNA_unspl/size_ratio;

Na_in_cell_conc = Na_in_cell/size_ratio;

Na_out_cell_conc = Na_out_cell/size_ratio;

NT_in_cell_conc = NT_in_cell/size_ratio;

NT_in_RNA_conc = NT_in_RNA/size_ratio;

p_in_cell_conc = p_in_cell/size_ratio;

p_in_use_conc = p_in_use/size_ratio;

p_trash_conc = p_trash/size_ratio;

p_unfold_conc = p_unfold/size_ratio;

rRNA_cytosol_conc = rRNA_cytosol/size_ratio;

rRNA_in_use_conc = rRNA_in_use/size_ratio;

rRNA_nuc_conc = rRNA_nuc/size_ratio;

rRNA_trash_conc = rRNA_trash/size_ratio;

rRNA_unspl_conc = rRNA_unspl/size_ratio;

snRNA_in_use_conc = snRNA_in_use/size_ratio;

snRNA_nuc_conc = snRNA_nuc/size_ratio;

snRNA_trash_conc = snRNA_trash/size_ratio;

snRNA_unspl_conc = snRNA_unspl/size_ratio;

sugar_in_cell_conc = sugar_in_cell/size_ratio;

tRNA_cytosol_conc = tRNA_cytosol/size_ratio;

tRNA_in_use_conc = tRNA_in_use/size_ratio;

tRNA_nuc_conc = tRNA_nuc/size_ratio;

tRNA_trash_conc = tRNA_trash/size_ratio;

mRNA_poly_unspl_conc = mRNA_poly_unspl/size_ratio;

mRNA_poly_nuc_conc = mRNA_poly_nuc/size_ratio;

mRNA_poly_inactive_conc = mRNA_poly_inactive/size_ratio;

mRNA_poly_cytosol_conc = mRNA_poly_cytosol/size_ratio;

mRNA_poly_in_use_conc = mRNA_poly_in_use/size_ratio;

mRNA_poly_trash_conc = mRNA_poly_trash/size_ratio;

p_poly_unfold_conc = p_poly_unfold/size_ratio;

p_poly_cytosol_conc = p_poly_cytosol/size_ratio;

p_poly_nuc_inactive_conc = p_poly_nuc_inactive/size_ratio;

p_poly_nuc_active_conc = p_poly_nuc_active/size_ratio;

p_poly_in_use_conc = p_poly_in_use/size_ratio;

p_poly_trash_conc = p_poly_trash/size_ratio;

mRNA_eIF_4_unspl_conc = mRNA_eIF_4_unspl/size_ratio;

mRNA_eIF_4_nuc_conc = mRNA_eIF_4_nuc/size_ratio;

mRNA_eIF_4_inactive_conc = mRNA_eIF_4_inactive/size_ratio;

mRNA_eIF_4_cytosol_conc = mRNA_eIF_4_cytosol/size_ratio;

mRNA_eIF_4_in_use_conc = mRNA_eIF_4_in_use/size_ratio;

mRNA_eIF_4_trash_conc = mRNA_eIF_4_trash/size_ratio;

p_eIF_4_unfold_conc = p_eIF_4_unfold/size_ratio;

p_eIF_4_cytosol_conc = p_eIF_4_cytosol/size_ratio;

p_eIF_4_in_use_conc = p_eIF_4_in_use/size_ratio;

p_eIF_4_trash_conc = p_eIF_4_trash/size_ratio;

mRNA_p27_unspl_conc = mRNA_p27_unspl/size_ratio;

mRNA_p27_nuc_conc = mRNA_p27_nuc/size_ratio;

mRNA_p27_inactive_conc = mRNA_p27_inactive/size_ratio;

mRNA_p27_cytosol_conc = mRNA_p27_cytosol/size_ratio;

mRNA_p27_in_use_conc = mRNA_p27_in_use/size_ratio;

mRNA_p27_trash_conc = mRNA_p27_trash/size_ratio;

p27_unfold_conc = p27_unfold/size_ratio;

p27_cytosol_conc = p27_cytosol/size_ratio;

p27_nuc_conc = p27_nuc/size_ratio;

p27_on_cyclins_conc = p27_on_cyclins/size_ratio;

p27_trash_conc = p27_trash/size_ratio;

mRNA_Rb_unspl_conc = mRNA_Rb_unspl/size_ratio;

mRNA_Rb_nuc_conc = mRNA_Rb_nuc/size_ratio;

mRNA_Rb_inactive_conc = mRNA_Rb_inactive/size_ratio;

mRNA_Rb_cytosol_conc = mRNA_Rb_cytosol/size_ratio;

mRNA_Rb_in_use_conc = mRNA_Rb_in_use/size_ratio;

mRNA_Rb_trash_conc = mRNA_Rb_trash/size_ratio;

Rb_unfold_conc = Rb_unfold/size_ratio;

Rb_cytosol_conc = Rb_cytosol/size_ratio;

Rb_nuc_conc = Rb_nuc/size_ratio;

Rb_on_E2F_conc = Rb_on_E2F/size_ratio;

Rb_phospho_inactive_conc = Rb_phospho_inactive/size_ratio;

Rb_trash_conc = Rb_trash/size_ratio;

mRNA_cycD_unspl_conc = mRNA_cycD_unspl/size_ratio;

mRNA_cycD_nuc_conc = mRNA_cycD_nuc/size_ratio;

mRNA_cycD_inactive_conc = mRNA_cycD_inactive/size_ratio;

mRNA_cycD_cytosol_conc = mRNA_cycD_cytosol/size_ratio;

mRNA_cycD_in_use_conc = mRNA_cycD_in_use/size_ratio;

mRNA_cycD_trash_conc = mRNA_cycD_trash/size_ratio;

cycD_unfold_conc = cycD_unfold/size_ratio;

cycD_cytosol_conc = cycD_cytosol/size_ratio;

cycD_nuc_conc = cycD_nuc/size_ratio;

cycD_Cdk4or6_active_conc = cycD_Cdk4or6_active/size_ratio;

cycD_Cdk4or6_on_p27_inactive_conc = cycD_Cdk4or6_on_p27_inactive/size_ratio;

cycD_trash_conc = cycD_trash/size_ratio;

mRNA_Cdk2_unspl_conc = mRNA_Cdk2_unspl/size_ratio;

mRNA_Cdk2_nuc_conc = mRNA_Cdk2_nuc/size_ratio;

mRNA_Cdk2_inactive_conc = mRNA_Cdk2_inactive/size_ratio;

mRNA_Cdk2_cytosol_conc = mRNA_Cdk2_cytosol/size_ratio;

mRNA_Cdk2_in_use_conc = mRNA_Cdk2_in_use/size_ratio;

mRNA_Cdk2_trash_conc = mRNA_Cdk2_trash/size_ratio;

Cdk2_unfold_conc = Cdk2_unfold/size_ratio;

Cdk2_cytosol_conc = Cdk2_cytosol/size_ratio;

Cdk2_nuc_conc = Cdk2_nuc/size_ratio;

Cdk2_on_cycE_conc = Cdk2_on_cycE/size_ratio;

Cdk2_on_cycA_conc = Cdk2_on_cycA/size_ratio;

Cdk2_trash_conc = Cdk2_trash/size_ratio;

mRNA_cycE_unspl_conc = mRNA_cycE_unspl/size_ratio;

mRNA_cycE_nuc_conc = mRNA_cycE_nuc/size_ratio;

mRNA_cycE_inactive_conc = mRNA_cycE_inactive/size_ratio;

mRNA_cycE_cytosol_conc = mRNA_cycE_cytosol/size_ratio;

mRNA_cycE_in_use_conc = mRNA_cycE_in_use/size_ratio;

mRNA_cycE_trash_conc = mRNA_cycE_trash/size_ratio;

cycE_unfold_conc = cycE_unfold/size_ratio;

cycE_cytosol_conc = cycE_cytosol/size_ratio;

cycE_nuc_conc = cycE_nuc/size_ratio;

cycE_Cdk2_inactive_conc = cycE_Cdk2_inactive/size_ratio;

cycE_Cdk2_active_conc = cycE_Cdk2_active/size_ratio;

cycE_Cdk2_on_p27_inactive_conc = cycE_Cdk2_on_p27_inactive/size_ratio;

cycE_trash_conc = cycE_trash/size_ratio;

mRNA_B_Myb_unspl_conc = mRNA_B_Myb_unspl/size_ratio;

mRNA_B_Myb_nuc_conc = mRNA_B_Myb_nuc/size_ratio;

mRNA_B_Myb_inactive_conc = mRNA_B_Myb_inactive/size_ratio;

mRNA_B_Myb_cytosol_conc = mRNA_B_Myb_cytosol/size_ratio;

mRNA_B_Myb_in_use_conc = mRNA_B_Myb_in_use/size_ratio;

mRNA_B_Myb_trash_conc = mRNA_B_Myb_trash/size_ratio;

B_Myb_unfold_conc = B_Myb_unfold/size_ratio;

B_Myb_cytosol_conc = B_Myb_cytosol/size_ratio;

B_Myb_nuc_conc = B_Myb_nuc/size_ratio;

B_Myb_phospho_active_conc = B_Myb_phospho_active/size_ratio;

B_Myb_on_DNA_conc = B_Myb_on_DNA/size_ratio;

B_Myb_trash_conc = B_Myb_trash/size_ratio;

mRNA_NF_Y_unspl_conc = mRNA_NF_Y_unspl/size_ratio;

mRNA_NF_Y_nuc_conc = mRNA_NF_Y_nuc/size_ratio;

mRNA_NF_Y_inactive_conc = mRNA_NF_Y_inactive/size_ratio;

mRNA_NF_Y_cytosol_conc = mRNA_NF_Y_cytosol/size_ratio;

mRNA_NF_Y_in_use_conc = mRNA_NF_Y_in_use/size_ratio;

mRNA_NF_Y_trash_conc = mRNA_NF_Y_trash/size_ratio;

NF_Y_unfold_conc = NF_Y_unfold/size_ratio;

NF_Y_cytosol_conc = NF_Y_cytosol/size_ratio;

NF_Y_nuc_conc = NF_Y_nuc/size_ratio;

NF_Y_phospho_active_conc = NF_Y_phospho_active/size_ratio;

NF_Y_on_DNA_conc = NF_Y_on_DNA/size_ratio;

NF_Y_trash_conc = NF_Y_trash/size_ratio;

mRNA_E2F_unspl_conc = mRNA_E2F_unspl/size_ratio;

mRNA_E2F_nuc_conc = mRNA_E2F_nuc/size_ratio;

mRNA_E2F_inactive_conc = mRNA_E2F_inactive/size_ratio;

mRNA_E2F_cytosol_conc = mRNA_E2F_cytosol/size_ratio;

mRNA_E2F_in_use_conc = mRNA_E2F_in_use/size_ratio;

mRNA_E2F_trash_conc = mRNA_E2F_trash/size_ratio;

E2F_unfold_conc = E2F_unfold/size_ratio;

E2F_cytosol_conc = E2F_cytosol/size_ratio;

E2F_nuc_conc = E2F_nuc/size_ratio;

E2F_Rb_inactive_conc = E2F_Rb_inactive/size_ratio;

E2F_on_DNA_conc = E2F_on_DNA/size_ratio;

E2F_trash_conc = E2F_trash/size_ratio;

mRNA_cycA_unspl_conc = mRNA_cycA_unspl/size_ratio;

mRNA_cycA_nuc_conc = mRNA_cycA_nuc/size_ratio;

mRNA_cycA_inactive_conc = mRNA_cycA_inactive/size_ratio;

mRNA_cycA_cytosol_conc = mRNA_cycA_cytosol/size_ratio;

mRNA_cycA_in_use_conc = mRNA_cycA_in_use/size_ratio;

mRNA_cycA_trash_conc = mRNA_cycA_trash/size_ratio;

cycA_unfold_conc = cycA_unfold/size_ratio;

cycA_cytosol_conc = cycA_cytosol/size_ratio;

cycA_nuc_conc = cycA_nuc/size_ratio;

cycA_Cdk2_inactive_conc = cycA_Cdk2_inactive/size_ratio;

cycA_Cdk2_on_p27_inactive_conc = cycA_Cdk2_on_p27_inactive/size_ratio;

cycA_Cdk2_active_conc = cycA_Cdk2_active/size_ratio;

cycA_Cdk1_cytosol_active_conc = cycA_Cdk1_cytosol_active/size_ratio;

cycA_Cdk1_phospho_inactive_conc = cycA_Cdk1_phospho_inactive/size_ratio;

cycA_Cdk1_nuc_active_conc = cycA_Cdk1_nuc_active/size_ratio;

cycA_trash_conc = cycA_trash/size_ratio;

mRNA_SCF_unspl_conc = mRNA_SCF_unspl/size_ratio;

mRNA_SCF_nuc_conc = mRNA_SCF_nuc/size_ratio;

mRNA_SCF_inactive_conc = mRNA_SCF_inactive/size_ratio;

mRNA_SCF_cytosol_conc = mRNA_SCF_cytosol/size_ratio;

mRNA_SCF_in_use_conc = mRNA_SCF_in_use/size_ratio;

mRNA_SCF_trash_conc = mRNA_SCF_trash/size_ratio;

SCF_unfold_conc = SCF_unfold/size_ratio;

SCF_in_cell_conc = SCF_in_cell/size_ratio;

SCF_on_Btrc_conc = SCF_on_Btrc/size_ratio;

SCF_on_Fbw7_conc = SCF_on_Fbw7/size_ratio;

SCF_on_Skp2_conc = SCF_on_Skp2/size_ratio;

SCF_trash_conc = SCF_trash/size_ratio;

mRNA_Skp2_unspl_conc = mRNA_Skp2_unspl/size_ratio;

mRNA_Skp2_nuc_conc = mRNA_Skp2_nuc/size_ratio;

mRNA_Skp2_inactive_conc = mRNA_Skp2_inactive/size_ratio;

mRNA_Skp2_cytosol_conc = mRNA_Skp2_cytosol/size_ratio;

mRNA_Skp2_in_use_conc = mRNA_Skp2_in_use/size_ratio;

mRNA_Skp2_trash_conc = mRNA_Skp2_trash/size_ratio;

Skp2_unfold_conc = Skp2_unfold/size_ratio;

Skp2_cytosol_conc = Skp2_cytosol/size_ratio;

Skp2_nuc_conc = Skp2_nuc/size_ratio;

Skp2_SCF_active_conc = Skp2_SCF_active/size_ratio;

Skp2_trash_conc = Skp2_trash/size_ratio;

mRNA_Btrc_unspl_conc = mRNA_Btrc_unspl/size_ratio;

mRNA_Btrc_nuc_conc = mRNA_Btrc_nuc/size_ratio;

mRNA_Btrc_inactive_conc = mRNA_Btrc_inactive/size_ratio;

mRNA_Btrc_cytosol_conc = mRNA_Btrc_cytosol/size_ratio;

mRNA_Btrc_in_use_conc = mRNA_Btrc_in_use/size_ratio;

mRNA_Btrc_trash_conc = mRNA_Btrc_trash/size_ratio;

Btrc_unfold_conc = Btrc_unfold/size_ratio;

Btrc_cytosol_conc = Btrc_cytosol/size_ratio;

Btrc_SCF_active_conc = Btrc_SCF_active/size_ratio;

Btrc_trash_conc = Btrc_trash/size_ratio;

mRNA_Fbw7_unspl_conc = mRNA_Fbw7_unspl/size_ratio;

mRNA_Fbw7_nuc_conc = mRNA_Fbw7_nuc/size_ratio;

mRNA_Fbw7_inactive_conc = mRNA_Fbw7_inactive/size_ratio;

mRNA_Fbw7_cytosol_conc = mRNA_Fbw7_cytosol/size_ratio;

mRNA_Fbw7_in_use_conc = mRNA_Fbw7_in_use/size_ratio;

mRNA_Fbw7_trash_conc = mRNA_Fbw7_trash/size_ratio;

Fbw7_unfold_conc = Fbw7_unfold/size_ratio;

Fbw7_cytosol_conc = Fbw7_cytosol/size_ratio;

Fbw7_nuc_conc = Fbw7_nuc/size_ratio;

Fbw7_SCF_active_conc = Fbw7_SCF_active/size_ratio;

Fbw7_trash_conc = Fbw7_trash/size_ratio;

mRNA_TF_grow_unspl_conc = mRNA_TF_grow_unspl/size_ratio;

mRNA_TF_grow_inactive_conc = mRNA_TF_grow_inactive/size_ratio;

mRNA_TF_grow_cytosol_conc = mRNA_TF_grow_cytosol/size_ratio;

mRNA_TF_grow_nuc_conc = mRNA_TF_grow_nuc/size_ratio;

mRNA_TF_grow_in_use_conc = mRNA_TF_grow_in_use/size_ratio;

mRNA_TF_grow_trash_conc = mRNA_TF_grow_trash/size_ratio;

TF_grow_unfold_conc = TF_grow_unfold/size_ratio;

TF_grow_cytosol_conc = TF_grow_cytosol/size_ratio;

TF_grow_nuc_conc = TF_grow_nuc/size_ratio;

TF_grow_on_DNA_conc = TF_grow_on_DNA/size_ratio;

TF_grow_trash_conc = TF_grow_trash/size_ratio;

NT_in_DNA_conc = NT_in_DNA/size_ratio;

mRNA_RC_unspl_conc = mRNA_RC_unspl/size_ratio;

mRNA_RC_nuc_conc = mRNA_RC_nuc/size_ratio;

mRNA_RC_inactive_conc = mRNA_RC_inactive/size_ratio;

mRNA_RC_cytosol_conc = mRNA_RC_cytosol/size_ratio;

mRNA_RC_in_use_conc = mRNA_RC_in_use/size_ratio;

mRNA_RC_trash_conc = mRNA_RC_trash/size_ratio;

RC_unfold_conc = RC_unfold/size_ratio;

RC_cytosol_conc = RC_cytosol/size_ratio;

RC_nuc_conc = RC_nuc/size_ratio;

RC_on_DNA_conc = RC_on_DNA/size_ratio;

RC_licensed_conc = RC_licensed/size_ratio;

RC_traversed_by_DNA_poly_conc = RC_traversed_by_DNA_poly/size_ratio;

RC_trash_conc = RC_trash/size_ratio;

RC_count_conc = RC_count/size_ratio;

mRNA_DNA_poly_unspl_conc = mRNA_DNA_poly_unspl/size_ratio;

mRNA_DNA_poly_nuc_conc = mRNA_DNA_poly_nuc/size_ratio;

mRNA_DNA_poly_inactive_conc = mRNA_DNA_poly_inactive/size_ratio;

mRNA_DNA_poly_cytosol_conc = mRNA_DNA_poly_cytosol/size_ratio;

mRNA_DNA_poly_in_use_conc = mRNA_DNA_poly_in_use/size_ratio;

mRNA_DNA_poly_trash_conc = mRNA_DNA_poly_trash/size_ratio;

DNA_poly_unfold_conc = DNA_poly_unfold/size_ratio;

DNA_poly_cytosol_conc = DNA_poly_cytosol/size_ratio;

DNA_poly_nuc_conc = DNA_poly_nuc/size_ratio;

DNA_poly_on_DNA_conc = DNA_poly_on_DNA/size_ratio;

DNA_poly_trash_conc = DNA_poly_trash/size_ratio;

mRNA_Wee1_unspl_conc = mRNA_Wee1_unspl/size_ratio;

mRNA_Wee1_nuc_conc = mRNA_Wee1_nuc/size_ratio;

mRNA_Wee1_inactive_conc = mRNA_Wee1_inactive/size_ratio;

mRNA_Wee1_cytosol_conc = mRNA_Wee1_cytosol/size_ratio;

mRNA_Wee1_in_use_conc = mRNA_Wee1_in_use/size_ratio;

mRNA_Wee1_trash_conc = mRNA_Wee1_trash/size_ratio;

Wee1_unfold_conc = Wee1_unfold/size_ratio;

Wee1_cytosol_active_conc = Wee1_cytosol_active/size_ratio;

Wee1_phospho_inactive_conc = Wee1_phospho_inactive/size_ratio;

Wee1_trash_conc = Wee1_trash/size_ratio;

mRNA_cycB_unspl_conc = mRNA_cycB_unspl/size_ratio;

mRNA_cycB_nuc_conc = mRNA_cycB_nuc/size_ratio;

mRNA_cycB_inactive_conc = mRNA_cycB_inactive/size_ratio;

mRNA_cycB_cytosol_conc = mRNA_cycB_cytosol/size_ratio;

mRNA_cycB_in_use_conc = mRNA_cycB_in_use/size_ratio;

mRNA_cycB_trash_conc = mRNA_cycB_trash/size_ratio;

cycB_unfold_conc = cycB_unfold/size_ratio;

cycB_cytosol_conc = cycB_cytosol/size_ratio;

cycB_Cdk1_cytosol_active_conc = cycB_Cdk1_cytosol_active/size_ratio;

cycB_Cdk1_phospho_inactive_conc = cycB_Cdk1_phospho_inactive/size_ratio;

cycB_Cdk1_nuc_active_conc = cycB_Cdk1_nuc_active/size_ratio;

cycB_trash_conc = cycB_trash/size_ratio;

mRNA_Cdk1_unspl_conc = mRNA_Cdk1_unspl/size_ratio;

mRNA_Cdk1_nuc_conc = mRNA_Cdk1_nuc/size_ratio;

mRNA_Cdk1_inactive_conc = mRNA_Cdk1_inactive/size_ratio;

mRNA_Cdk1_cytosol_conc = mRNA_Cdk1_cytosol/size_ratio;

mRNA_Cdk1_in_use_conc = mRNA_Cdk1_in_use/size_ratio;

mRNA_Cdk1_trash_conc = mRNA_Cdk1_trash/size_ratio;

Cdk1_unfold_conc = Cdk1_unfold/size_ratio;

Cdk1_inactive_conc = Cdk1_inactive/size_ratio;

Cdk1_in_cell_conc = Cdk1_in_cell/size_ratio;

Cdk1_on_cycB_conc = Cdk1_on_cycB/size_ratio;

Cdk1_on_cycA_conc = Cdk1_on_cycA/size_ratio;

Cdk1_trash_conc = Cdk1_trash/size_ratio;

mRNA_Cdc25C_unspl_conc = mRNA_Cdc25C_unspl/size_ratio;

mRNA_Cdc25C_nuc_conc = mRNA_Cdc25C_nuc/size_ratio;

mRNA_Cdc25C_inactive_conc = mRNA_Cdc25C_inactive/size_ratio;

mRNA_Cdc25C_cytosol_conc = mRNA_Cdc25C_cytosol/size_ratio;

mRNA_Cdc25C_in_use_conc = mRNA_Cdc25C_in_use/size_ratio;

mRNA_Cdc25C_trash_conc = mRNA_Cdc25C_trash/size_ratio;

Cdc25C_unfold_conc = Cdc25C_unfold/size_ratio;

Cdc25C_cytosol_inactive_conc = Cdc25C_cytosol_inactive/size_ratio;

Cdc25C_cytosol_phospho_active_conc = Cdc25C_cytosol_phospho_active/size_ratio;

Cdc25C_nuc_phospho_active_conc = Cdc25C_nuc_phospho_active/size_ratio;

Cdc25C_trash_conc = Cdc25C_trash/size_ratio;

mRNA_Plk1_unspl_conc = mRNA_Plk1_unspl/size_ratio;

mRNA_Plk1_nuc_conc = mRNA_Plk1_nuc/size_ratio;

mRNA_Plk1_inactive_conc = mRNA_Plk1_inactive/size_ratio;

mRNA_Plk1_cytosol_conc = mRNA_Plk1_cytosol/size_ratio;

mRNA_Plk1_in_use_conc = mRNA_Plk1_in_use/size_ratio;

mRNA_Plk1_trash_conc = mRNA_Plk1_trash/size_ratio;

Plk1_unfold_conc = Plk1_unfold/size_ratio;

Plk1_cytosol_inactive_conc = Plk1_cytosol_inactive/size_ratio;

Plk1_cytosol_phospho_active_conc = Plk1_cytosol_phospho_active/size_ratio;

Plk1_nuc_phospho_active_conc = Plk1_nuc_phospho_active/size_ratio;

Plk1_trash_conc = Plk1_trash/size_ratio;

mRNA_Emi1_unspl_conc = mRNA_Emi1_unspl/size_ratio;

mRNA_Emi1_nuc_conc = mRNA_Emi1_nuc/size_ratio;

mRNA_Emi1_inactive_conc = mRNA_Emi1_inactive/size_ratio;

mRNA_Emi1_cytosol_conc = mRNA_Emi1_cytosol/size_ratio;

mRNA_Emi1_in_use_conc = mRNA_Emi1_in_use/size_ratio;

mRNA_Emi1_trash_conc = mRNA_Emi1_trash/size_ratio;

Emi1_unfold_conc = Emi1_unfold/size_ratio;

Emi1_in_cell_conc = Emi1_in_cell/size_ratio;

Emi1_on_Cdh1_conc = Emi1_on_Cdh1/size_ratio;

Emi1_on_Cdc20_conc = Emi1_on_Cdc20/size_ratio;

Emi1_phospho_inactive_conc = Emi1_phospho_inactive/size_ratio;

Emi1_trash_conc = Emi1_trash/size_ratio;

mRNA_APC_unspl_conc = mRNA_APC_unspl/size_ratio;

mRNA_APC_nuc_conc = mRNA_APC_nuc/size_ratio;

mRNA_APC_inactive_conc = mRNA_APC_inactive/size_ratio;

mRNA_APC_cytosol_conc = mRNA_APC_cytosol/size_ratio;

mRNA_APC_in_use_conc = mRNA_APC_in_use/size_ratio;

mRNA_APC_trash_conc = mRNA_APC_trash/size_ratio;

APC_unfold_conc = APC_unfold/size_ratio;

APC_in_cell_conc = APC_in_cell/size_ratio;

APC_on_Cdh1_conc = APC_on_Cdh1/size_ratio;

APC_on_Cdc20_conc = APC_on_Cdc20/size_ratio;

APC_trash_conc = APC_trash/size_ratio;

mRNA_Cdh1_unspl_conc = mRNA_Cdh1_unspl/size_ratio;

mRNA_Cdh1_nuc_conc = mRNA_Cdh1_nuc/size_ratio;

mRNA_Cdh1_inactive_conc = mRNA_Cdh1_inactive/size_ratio;

mRNA_Cdh1_cytosol_conc = mRNA_Cdh1_cytosol/size_ratio;

mRNA_Cdh1_in_use_conc = mRNA_Cdh1_in_use/size_ratio;

mRNA_Cdh1_trash_conc = mRNA_Cdh1_trash/size_ratio;

Cdh1_unfold_conc = Cdh1_unfold/size_ratio;

Cdh1_in_cell_conc = Cdh1_in_cell/size_ratio;

Cdh1_Emi1_inactive_conc = Cdh1_Emi1_inactive/size_ratio;

Cdh1_phospho_inactive_conc = Cdh1_phospho_inactive/size_ratio;

Cdh1_APC_active_conc = Cdh1_APC_active/size_ratio;

Cdh1_trash_conc = Cdh1_trash/size_ratio;

mRNA_Cdc20_unspl_conc = mRNA_Cdc20_unspl/size_ratio;

mRNA_Cdc20_nuc_conc = mRNA_Cdc20_nuc/size_ratio;

mRNA_Cdc20_inactive_conc = mRNA_Cdc20_inactive/size_ratio;

mRNA_Cdc20_cytosol_conc = mRNA_Cdc20_cytosol/size_ratio;

mRNA_Cdc20_in_use_conc = mRNA_Cdc20_in_use/size_ratio;

mRNA_Cdc20_trash_conc = mRNA_Cdc20_trash/size_ratio;

Cdc20_unfold_conc = Cdc20_unfold/size_ratio;

Cdc20_cytosol_conc = Cdc20_cytosol/size_ratio;

Cdc20_nuc_conc = Cdc20_nuc/size_ratio;

Cdc20_Emi1_inactive_conc = Cdc20_Emi1_inactive/size_ratio;

Cdc20_APC_inactive_conc = Cdc20_APC_inactive/size_ratio;

Cdc20_APC_active_conc = Cdc20_APC_active/size_ratio;

Cdc20_trash_conc = Cdc20_trash/size_ratio;

mRNA_Cdc14_unspl_conc = mRNA_Cdc14_unspl/size_ratio;

mRNA_Cdc14_nuc_conc = mRNA_Cdc14_nuc/size_ratio;

mRNA_Cdc14_inactive_conc = mRNA_Cdc14_inactive/size_ratio;

mRNA_Cdc14_cytosol_conc = mRNA_Cdc14_cytosol/size_ratio;

mRNA_Cdc14_in_use_conc = mRNA_Cdc14_in_use/size_ratio;

mRNA_Cdc14_trash_conc = mRNA_Cdc14_trash/size_ratio;

Cdc14_unfold_conc = Cdc14_unfold/size_ratio;

Cdc14_cytosol_conc = Cdc14_cytosol/size_ratio;

Cdc14_nuc_inactive_conc = Cdc14_nuc_inactive/size_ratio;

Cdc14_inhibited_conc = Cdc14_inhibited/size_ratio;

Cdc14_phospho_active_conc = Cdc14_phospho_active/size_ratio;

Cdc14_trash_conc = Cdc14_trash/size_ratio;

mRNA_Cdc25A_unspl_conc = mRNA_Cdc25A_unspl/size_ratio;

mRNA_Cdc25A_nuc_conc = mRNA_Cdc25A_nuc/size_ratio;

mRNA_Cdc25A_inactive_conc = mRNA_Cdc25A_inactive/size_ratio;

mRNA_Cdc25A_cytosol_conc = mRNA_Cdc25A_cytosol/size_ratio;

mRNA_Cdc25A_in_use_conc = mRNA_Cdc25A_in_use/size_ratio;

mRNA_Cdc25A_trash_conc = mRNA_Cdc25A_trash/size_ratio;

Cdc25A_unfold_conc = Cdc25A_unfold/size_ratio;

Cdc25A_cytosol_conc = Cdc25A_cytosol/size_ratio;

Cdc25A_nuc_inactive_conc = Cdc25A_nuc_inactive/size_ratio;

Cdc25A_phospho_active_conc = Cdc25A_phospho_active/size_ratio;

Cdc25A_trash_conc = Cdc25A_trash/size_ratio;

mRNA_Cdc25B_unspl_conc = mRNA_Cdc25B_unspl/size_ratio;

mRNA_Cdc25B_nuc_conc = mRNA_Cdc25B_nuc/size_ratio;

mRNA_Cdc25B_inactive_conc = mRNA_Cdc25B_inactive/size_ratio;

mRNA_Cdc25B_cytosol_conc = mRNA_Cdc25B_cytosol/size_ratio;

mRNA_Cdc25B_in_use_conc = mRNA_Cdc25B_in_use/size_ratio;

mRNA_Cdc25B_trash_conc = mRNA_Cdc25B_trash/size_ratio;

Cdc25B_unfold_conc = Cdc25B_unfold/size_ratio;

Cdc25B_cytosol_inactive_conc = Cdc25B_cytosol_inactive/size_ratio;

Cdc25B_cytosol_phospho_active_conc = Cdc25B_cytosol_phospho_active/size_ratio;

Cdc25B_trash_conc = Cdc25B_trash/size_ratio;

mRNA_Securin_unspl_conc = mRNA_Securin_unspl/size_ratio;

mRNA_Securin_nuc_conc = mRNA_Securin_nuc/size_ratio;

mRNA_Securin_inactive_conc = mRNA_Securin_inactive/size_ratio;

mRNA_Securin_cytosol_conc = mRNA_Securin_cytosol/size_ratio;

mRNA_Securin_in_use_conc = mRNA_Securin_in_use/size_ratio;

mRNA_Securin_trash_conc = mRNA_Securin_trash/size_ratio;

Securin_unfold_conc = Securin_unfold/size_ratio;

Securin_cytosol_conc = Securin_cytosol/size_ratio;

Securin_nuc_conc = Securin_nuc/size_ratio;

Securin_on_chromo_conc = Securin_on_chromo/size_ratio;

Securin_trash_conc = Securin_trash/size_ratio;

mRNA_cycC_unspl_conc = mRNA_cycC_unspl/size_ratio;

mRNA_cycC_nuc_conc = mRNA_cycC_nuc/size_ratio;

mRNA_cycC_inactive_conc = mRNA_cycC_inactive/size_ratio;

mRNA_cycC_cytosol_conc = mRNA_cycC_cytosol/size_ratio;

mRNA_cycC_in_use_conc = mRNA_cycC_in_use/size_ratio;

mRNA_cycC_trash_conc = mRNA_cycC_trash/size_ratio;

cycC_unfold_conc = cycC_unfold/size_ratio;

cycC_cytosol_conc = cycC_cytosol/size_ratio;

cycC_nuc_conc = cycC_nuc/size_ratio;

cycC_Cdk8_active_conc = cycC_Cdk8_active/size_ratio;

cycC_Cdk8_inactive_conc = cycC_Cdk8_inactive/size_ratio;

cycC_trash_conc = cycC_trash/size_ratio;

mRNA_KPC_unspl_conc = mRNA_KPC_unspl/size_ratio;

mRNA_KPC_nuc_conc = mRNA_KPC_nuc/size_ratio;

mRNA_KPC_inactive_conc = mRNA_KPC_inactive/size_ratio;

mRNA_KPC_cytosol_conc = mRNA_KPC_cytosol/size_ratio;

mRNA_KPC_in_use_conc = mRNA_KPC_in_use/size_ratio;

mRNA_KPC_trash_conc = mRNA_KPC_trash/size_ratio;

KPC_unfold_conc = KPC_unfold/size_ratio;

KPC_in_cell_conc = KPC_in_cell/size_ratio;

KPC_active_conc = KPC_active/size_ratio;

KPC_trash_conc = KPC_trash/size_ratio;

% !!!!!!!!!!!!!!!!!!!!!!!!!!!!!!!!!!!!!!!!!!!!!!! RATE EQNS

% equations "ATP_" give actual rate of energy used in a process

% equations "energy_" give rate of energy anticipated in a process

% equations "ER_" give elongation rate

% equations "NT_frac_" give percent of various RNA types

% equations "poly" give number of RNA polymerases

% equations "R_diff_" give normalized ratio (times 10^6)

% of difference of input and output rates

%

% 000000000011111111112222222222333333333344444444445555555555666666666677777777778888888888

%

energy_dispose = ATP_u*k_dk_p...

*(p_in_cell_conc + p_in_use_conc + p_unfold_conc)...

+ ATP_proteasome*k_cut_p*p_trash_conc*x_p;

energy_Na_pump = ATP_Na*k_Na_pump_out*Na_in_cell_conc;

energy_out_nm = ATP_NT_out_nm*(x_mRNA*k_nm_mRNA*mRNA_nuc_conc...

+ x_rRNA*k_nm_rRNA*rRNA_nuc_conc...

+ x_tRNA*k_nm_tRNA*tRNA_nuc_conc);

energy_p_transport = ATP_mem_transfer*k_bind_p*p_in_cell_conc...

*(frac_p_nuc + frac_p_ER + frac_p_mitochondria...

+ frac_p_lysosome);

spl_pool = exon_mRNA*mRNA_unspl + exon_rRNA*rRNA_unspl...

+ exon_snRNA*snRNA_unspl;

spl_pool_conc = spl_pool/size_ratio;

spl_frac = min(1, k_spl*snRNA_in_use_conc/spl_pool_conc);

energy_spl = ATP_spl*(exon_mRNA*min(mRNA_unspl,...

spl_frac*exon_mRNA*mRNA_unspl)...

+ exon_rRNA*min(rRNA_unspl, spl_frac*exon_rRNA*rRNA_unspl)...

+ exon_snRNA*min(snRNA_unspl,...

spl_frac*exon_snRNA*snRNA_unspl));

energy_structure = ATP_polymerization*frac_p_cytosol*k_bind_p*p_in_cell_conc...

+ ATP_glycosolation*frac_p_ER*k_bind_p*p_in_cell_conc...

+ ATP_fat*k_make_vesicle*fats_cytosol_conc...

+ ATP_vesicle/x_vesicle*k_bind_fats*fats_in_vesicles_conc;

ER_p = k_ER_p_constant*i_ER_p*AA_on_tRNA_conc;

k_p_txl = ER_p/x_p;

e_txl_tRNA = ATP_tRNA*min(k_tRNA_AA_binding*tRNA_cytosol_conc...

*AA_in_cytosol_conc, AA_in_cytosol_conc);

e_txl_AA = ATP_AA*x_p*min(k_p_txl*rRNA_in_use_conc, tRNA_in_use/x_p);

e_txl_fold = ATP_fold*k_fold_p*p_unfold;

e_txl_eIF_4 = ATP_eIF_4*k_bind_rRNA_mRNA*mRNA_cytosol_conc*rRNA_cytosol_conc;

energy_txl = e_txl_tRNA + e_txl_AA + e_txl_fold + e_txl_eIF_4;

ER_RNA = k_ER_RNA_constant*i_ER_RNA*NT_in_cell_conc;

ER_DNA = k_ER_DNA_constant*i_ER_DNA*NT_in_cell_conc;

polyI = frac_polyI*p_poly_in_use;

polyII = frac_polyII*p_poly_in_use;

polyIII = frac_polyIII*p_poly_in_use;

NT_request = ER_RNA*(polyI + polyII + frac_polyII_snRNA*polyII + polyIII)...

+ DNA_poly_on_DNA*ER_DNA;

NT_frac_avail = min(1, NT_in_cell/NT_request);

k_mRNA_txs = NT_frac_avail*ER_RNA/x_mRNA_unspl;

k_rRNA_txs = NT_frac_avail*ER_RNA/x_rRNA_unspl;

k_snRNA_txs = NT_frac_avail*ER_RNA/x_snRNA_unspl;

k_tRNA_txs = NT_frac_avail*ER_RNA/x_tRNA;

energy_txs = ATP_NT_txs*NT_frac_avail...

*(x_mRNA_unspl*k_mRNA_txs*polyII...

+ x_rRNA_unspl*k_rRNA_txs*polyI...

+ x_snRNA_unspl*k_snRNA_txs*frac_polyII_snRNA*polyII...

+ x_tRNA*k_tRNA_txs*polyIII);

energy_DNA_repl = ATP_DNA_repl*NT_frac_avail*ER_DNA*DNA_poly_on_DNA;

energy_in_pm = ATP_pm_transfer*(k_pm_in_AA...

*max(0, i_AA_in_cytosol - AA_in_cytosol_conc)...

+ k_pm_in_ADP*max(0, i_ATP_in_cell + i_ADP_in_cell...

- (ATP_in_cell_conc + ADP_in_cell_conc))...

+ k_pm_in_NT*max(0, i_NT_in_cell - NT_in_cell_conc));

energy_request = energy_dispose + energy_out_nm + energy_Na_pump...

+ energy_p_transport + energy_txs + energy_txl...

+ energy_structure + energy_spl + energy_in_pm;

ATP_frac_avail = min(1, ATP_in_cell/energy_request);

txs_mRNA = ATP_frac_avail*k_mRNA_txs*polyII;

txs_rRNA = ATP_frac_avail*k_rRNA_txs*polyI;

txs_snRNA = ATP_frac_avail*k_snRNA_txs*frac_polyII_snRNA*polyII;

txs_tRNA = ATP_frac_avail*k_tRNA_txs*polyIII;

ATP_txs = ATP_NT*(x_mRNA_unspl*txs_mRNA + x_rRNA_unspl*txs_rRNA...

+ x_snRNA_unspl*txs_snRNA + x_tRNA*txs_tRNA);

bind_p = ATP_frac_avail*k_bind_p*p_in_cell_conc;

u1_p = ATP_frac_avail*k_dk_p*p_in_cell_conc;

u2_p = ATP_frac_avail*k_dk_p*p_in_use_conc;

u3_p = ATP_frac_avail*k_dk_p*p_unfold_conc;

cut_p = ATP_frac_avail*k_cut_p*p_trash_conc;

ATP_dispose = ATP_u*(u1_p + u2_p + u3_p) + ATP_proteasome*x_p*cut_p;

Na_pump = k_Na_pump_out*ATP_frac_avail*Na_in_cell_conc;

ATP_Na_pump = ATP_Na*Na_pump;

nm_out_mRNA = ATP_frac_avail*k_nm_mRNA*mRNA_nuc_conc;

nm_out_rRNA = ATP_frac_avail*k_nm_rRNA*rRNA_nuc_conc;

nm_out_tRNA = ATP_frac_avail*k_nm_tRNA*tRNA_nuc_conc;

ATP_out_nm = ATP_NT_out_nm*(x_mRNA*nm_out_mRNA + x_rRNA*nm_out_rRNA...

+ x_tRNA*nm_out_tRNA);

glycosolation = ATP_frac_avail*frac_p_ER*bind_p;

polymerization = ATP_frac_avail*frac_p_cytosol*bind_p;

p_into_ER = frac_p_ER*bind_p;

p_into_lysosomes = frac_p_lysosome*bind_p;

p_into_mitochondria = frac_p_mitochondria*bind_p;

p_into_nm = frac_p_nuc*bind_p;

ATP_p_transport = ATP_mem_transfer*(p_into_nm + p_into_ER...

+ p_into_lysosomes + p_into_mitochondria);

spl_mRNA = ATP_frac_avail*spl_frac*mRNA_unspl;

spl_rRNA = ATP_frac_avail*spl_frac*rRNA_unspl;

spl_snRNA = ATP_frac_avail*spl_frac*snRNA_unspl;

ATP_for_spl = ATP_spl*(spl_mRNA*exon_mRNA + spl_rRNA*exon_rRNA...

+ spl_snRNA*exon_snRNA);

make_vesicle = ATP_frac_avail*k_make_vesicle*fats_cytosol_conc;

bind_fats = ATP_frac_avail*k_bind_fats*fats_in_vesicles_conc;

ATP_structure = ATP_polymerization*polymerization...

+ ATP_glycosolation*glycosolation...

+ ATP_fat*make_vesicle + ATP_vesicle/x_vesicle*bind_fats;

mRNA_in_use_blocked = rRNA_in_use*rRNA_spacing_on_mRNA/x_mRNA;

tot_mRNA_avail = mRNA_cytosol + mRNA_in_use - mRNA_in_use_blocked;

tot_mRNA_avail_conc = tot_mRNA_avail/size_ratio;

bind_rRNA = ATP_frac_avail*k_bind_rRNA_mRNA*tot_mRNA_avail_conc...

*rRNA_cytosol_conc;

bind_mRNA = bind_rRNA*mRNA_cytosol_conc/tot_mRNA_avail_conc;

% bind_rRNA = ATP_frac_avail*k_bind_rRNA_mRNA*mRNA_cytosol_conc...

% *rRNA_cytosol_conc;

% bind_mRNA = bind_rRNA;

bind_snRNA = k_bind_snRNA*snRNA_nuc_conc;

bind_tRNA = ATP_frac_avail*k_tRNA_AA_binding*tRNA_cytosol_conc...

*AA_in_cytosol_conc;

bind_NT_in_RNA = x_mRNA_unspl*txs_mRNA + x_rRNA_unspl*txs_rRNA...

+ x_snRNA_unspl*txs_snRNA + x_tRNA*txs_tRNA;

bind_NT_in_DNA = ATP_frac_avail*NT_frac_avail*ER_DNA*DNA_poly_on_DNA;

unbind_rRNA = ATP_frac_avail*k_p_txl*rRNA_in_use_conc;

txl_p = unbind_rRNA;

ATP_txl = ATP_tRNA*bind_tRNA + ATP_AA*x_p*txl_p + ATP_fold*txl_p...

+ ATP_eIF_4*bind_rRNA;

pm_in_AA = ATP_frac_avail*k_pm_in_AA...

*max(0, i_AA_in_cytosol - AA_in_cytosol_conc);

pm_in_ADP = ATP_frac_avail*k_pm_in_ADP*max(0, i_ATP_in_cell...

+ i_ADP_in_cell - (ATP_in_cell_conc + ADP_in_cell_conc));

% if(dividing == 1)

% pm_in_NT = 0;

% else

pm_in_NT = ATP_frac_avail*k_pm_in_NT*max(0, i_NT_in_cell - NT_in_cell_conc);

% end

ATP_in_pm = ATP_pm_transfer*(pm_in_AA + pm_in_ADP + pm_in_NT);

ATP_demand = ATP_dispose + ATP_out_nm + ATP_p_transport + ATP_txl + ATP_txs...

+ ATP_structure + ATP_for_spl + ATP_Na_pump + ATP_in_pm;

Eq_ATP = ADP_in_cell/ATP_in_cell;

% ATP_synthase = k_ATP_synthase*ADP_in_cell_conc*H_between_mito_mems_conc...

% /H_in_inner_mito_mems_conc;

% ATP_synthase = k_ATP_synthase*ADP_in_cell_conc*(H_between_mito_mems_conc...

% - H_in_inner_mito_mems_conc);

ATP_synthase = k_ATP_synthase*Eq_ATP*H_between_mito_mems_conc...

/H_in_inner_mito_mems_conc;

% ATP_synthase = k_ATP_synthase*Eq_ATP*(H_between_mito_mems_conc...

% - H_in_inner_mito_mems_conc);

bind_AA = bind_tRNA;

bind_ADP = ATP_H*ATP_synthase;

cut_spljunk_mRNA = k_cut_spljunk_mRNA*junk_spl_mRNA;

cut_spljunk_rRNA = k_cut_spljunk_rRNA*junk_spl_rRNA;

cut_spljunk_snRNA = k_cut_spljunk_snRNA*junk_spl_snRNA;

cut_mRNA = k_cut_mRNA*mRNA_trash;

cut_rRNA = k_cut_rRNA*rRNA_trash;

cut_snRNA = k_cut_snRNA*snRNA_trash;

cut_tRNA = k_cut_tRNA*tRNA_trash;

dispose_ADP = ADP_trash;

dk1_ADP = k_dk_ADP*ADP_in_cell;

dk1_mRNA = k_dk_mRNA*mRNA_cytosol;

dk1_rRNA = k_dk_rRNA*rRNA_cytosol;

dk1_snRNA = k_dk_snRNA*snRNA_nuc;

dk1_tRNA = k_dk_tRNA*tRNA_cytosol;

dk2_ADP = k_dk_ADP*ATP_in_cell;

dk2_mRNA = k_dk_mRNA*mRNA_unspl;

dk2_rRNA = k_dk_rRNA*rRNA_unspl;

dk2_snRNA = k_dk_snRNA*snRNA_unspl;

dk2_tRNA = k_dk_tRNA*tRNA_nuc;

dk3_mRNA = k_dk_mRNA*mRNA_nuc;

dk3_rRNA = k_dk_rRNA*rRNA_nuc;

dk3_snRNA = k_dk_snRNA*snRNA_in_use;

dk3_tRNA = k_dk_tRNA*tRNA_in_use;

dk4_mRNA = k_dk_mRNA*mRNA_in_use;

dk4_rRNA = k_dk_rRNA*rRNA_in_use;

dk5_mRNA = k_dk_mRNA*mRNA_inactive;

% dk5_mRNA = 0;

dk_AA = ATP_frac_avail*k_dk_AA*(AA_in_cytosol + AA_on_tRNA + AA_in_p);

dk1_fats = k_dk_fat*fats_cytosol;

dk2_fats = k_dk_fat*fats_in_vesicles;

dk3_fats = k_dk_fat*fats_in_mem;

% NOTE: I am assuming when an NT decays in RNA, it causes the RNA to decay,

% and it is accounted for in RNA decay,

% and I ignore NT decay in DNA

dk_NT = k_dk_NT*NT_in_cell;

fold_p = ATP_frac_avail*k_fold_p*p_unfold;

H_grow = k_grow_H*max(0, i_H_between_mito_mems - H_between_mito_mems_conc);

H_shrink = 0;

% zwork25

% H_pump = min(ATP_respiration/ATP_H*sugar_in_cell,...

% k_H_pump*H_in_inner_mito_mems_conc/H_between_mito_mems_conc...

% /ATP_in_cell_conc);

% H_pump = min(ATP_respiration/ATP_H*sugar_in_cell,...

% k_H_pump*H_in_inner_mito_mems_conc*H_in_inner_mito_mems_conc...

% /H_between_mito_mems_conc/ATP_in_cell_conc);

H_pump = min(ATP_respiration/ATP_H*sugar_in_cell,...

k_H_pump*Eq_ATP*H_in_inner_mito_mems_conc/H_between_mito_mems_conc);

mRNA_synthesis_frac = x_mRNA_unspl*txs_mRNA/bind_NT_in_RNA;

% Na_grow = k_grow_Na*max(0, size_ratio*(i_Na_out_cell - Na_out_cell));

Na_grow = k_grow_Na*max(0, i_Na_out_cell - Na_out_cell_conc);

Na_shrink = 0;

Na_return = max(0, k_Na_return*(Na_out_cell_conc - Na_in_cell_conc));

NT_junk_spl_pool = junk_spl_mRNA + junk_spl_rRNA + junk_spl_snRNA + junk_spl;

NT_mRNA_pool = x_mRNA_unspl*mRNA_unspl...

+ x_mRNA*(mRNA_nuc + mRNA_cytosol + mRNA_in_use);

NT_mRNA_txs = 0.5*polyII*x_mRNA_unspl;

NT_rRNA_pool = x_rRNA_unspl*rRNA_unspl...

+ x_rRNA*(rRNA_nuc + rRNA_cytosol + rRNA_in_use);

NT_rRNA_txs = 0.5*polyI*x_rRNA_unspl;

NT_snRNA_pool = x_snRNA_unspl*snRNA_unspl...

+ x_snRNA*(snRNA_nuc + snRNA_in_use);

NT_snRNA_txs = 0.5*frac_polyII_snRNA*polyII*x_snRNA_unspl;

NT_trash_pool = x_mRNA*mRNA_trash + x_rRNA*rRNA_trash...

+ x_snRNA*snRNA_trash + x_tRNA*tRNA_trash;

NT_tRNA_pool = x_tRNA*(tRNA_nuc + tRNA_cytosol + tRNA_in_use);

NT_tRNA_txs = 0.5*polyIII*x_tRNA;

NT_txs = NT_mRNA_txs + NT_rRNA_txs + NT_snRNA_txs + NT_tRNA_txs;

temp_NT = NT_txs + NT_mRNA_pool + NT_rRNA_pool + NT_snRNA_pool...

+ NT_tRNA_pool + NT_junk_spl_pool + NT_trash_pool;

total_NT = temp_NT + NT_in_cell;

NT_frac_hnRNA = (0.5*polyII*x_mRNA_unspl + x_mRNA_unspl*mRNA_unspl...

+ x_mRNA*mRNA_nuc + (dk2_mRNA + dk3_mRNA)...

/cut_mRNA*mRNA_trash*x_mRNA + junk_spl_mRNA)/temp_NT;

NT_frac_mRNA = x_mRNA*(mRNA_cytosol + mRNA_in_use...

+ (dk1_mRNA + dk4_mRNA + dk5_mRNA)/cut_mRNA*mRNA_trash)/temp_NT;

% NT_frac_mRNA = x_mRNA*(mRNA_cytosol + mRNA_in_use...

% + (dk1_mRNA + dk4_mRNA)/cut_mRNA*mRNA_trash)/temp_NT;

NT_frac_rRNA = x_rRNA*(rRNA_cytosol + rRNA_in_use...

+ (dk1_rRNA + dk4_rRNA)/cut_rRNA*rRNA_trash)/temp_NT;

NT_frac_rRNA_precursors = (0.5*polyI*x_rRNA_unspl + x_rRNA_unspl*rRNA_unspl...

+ x_rRNA*rRNA_nuc + (dk2_rRNA + dk3_rRNA)...

/cut_rRNA*rRNA_trash*x_rRNA...

+ junk_spl_rRNA)/temp_NT;

NT_frac_snRNA = (0.5*frac_polyII_snRNA*polyII*x_snRNA...

+ x_snRNA_unspl*snRNA_unspl + x_snRNA*(snRNA_nuc...

+ snRNA_trash + snRNA_in_use) + junk_spl_snRNA)/temp_NT;

NT_frac_tRNA = (0.5*polyIII*x_tRNA + x_tRNA*(tRNA_nuc...

+ tRNA_cytosol + tRNA_trash + tRNA_in_use))/temp_NT;

NT_frac_RNA_nuc = NT_frac_rRNA_precursors + NT_frac_hnRNA + NT_frac_snRNA...

+ (x_tRNA*tRNA_nuc + 0.5*polyIII*x_tRNA)/temp_NT;

pm_in_fats = k_pm_in_fats*max(0, i_fats_cytosol - fats_cytosol_conc);

pm_out_sugar = H_pump*ATP_H/(ATP_respiration + ATP_glycolysis);

pm_in_sugar = k_pm_in_sugar*max(0, i_sugar_in_cell - sugar_in_cell_conc);

R_diff_mRNA_cyto = 1E6*(nm_out_mRNA...

- (dk1_mRNA + dk4_mRNA + dk5_mRNA))/nm_out_mRNA;

R_diff_mRNA_dk = 1E6*(txs_mRNA...

- (dk1_mRNA + dk2_mRNA + dk3_mRNA + dk4_mRNA...

+ dk5_mRNA))/txs_mRNA;

% R_diff_mRNA_cyto = 1E6*(nm_out_mRNA - (dk1_mRNA + dk4_mRNA))/nm_out_mRNA;

% R_diff_mRNA_dk = 1E6*(txs_mRNA...

% - (dk1_mRNA + dk2_mRNA + dk3_mRNA + dk4_mRNA))/txs_mRNA;

R_diff_mRNA_nm = 1E6*(spl_mRNA - (nm_out_mRNA + dk3_mRNA))/spl_mRNA;

R_diff_mRNA_spl = 1E6*(txs_mRNA - (spl_mRNA + dk2_mRNA))/txs_mRNA;

R_diff_p_dk = 1E6*(txl_p - (u1_p + u2_p + u3_p))/txl_p;

R_diff_p_fold = 1E6*(fold_p - (u1_p + u2_p))/fold_p;

R_diff_rRNA_cyto = 1E6*(nm_out_rRNA - (dk1_rRNA + dk4_rRNA))/nm_out_rRNA;

R_diff_rRNA_dk = 1E6*(txs_rRNA...

- (dk1_rRNA + dk2_rRNA + dk3_rRNA + dk4_rRNA))/txs_rRNA;

R_diff_rRNA_nm = 1E6*(spl_rRNA - (nm_out_rRNA + dk3_rRNA))/spl_rRNA;

R_diff_rRNA_spl = 1E6*(txs_rRNA - (spl_rRNA + dk2_rRNA))/txs_rRNA;

R_diff_snRNA_dk = 1E6*(txs_snRNA...

- (dk1_snRNA + dk2_snRNA + dk3_snRNA))/txs_snRNA;

R_diff_snRNA_spl = 1E6*(txs_snRNA - (spl_snRNA + dk2_snRNA))/txs_snRNA;

R_diff_tRNA_cyto = 1E6*(nm_out_tRNA - (dk1_tRNA + dk3_tRNA))/nm_out_tRNA;

R_diff_tRNA_dk = 1E6*(txs_tRNA - (dk1_tRNA + dk2_tRNA + dk3_tRNA))/txs_tRNA;

R_diff_tRNA_nm = 1E6*(txs_tRNA - (dk1_tRNA + dk2_tRNA + dk3_tRNA))/txs_tRNA;

rRNA_synthesis_frac = x_rRNA_unspl*txs_rRNA/bind_NT_in_RNA;

snRNA_synthesis_frac = x_snRNA_unspl*txs_snRNA/bind_NT_in_RNA;

spljunk = junk_spl;

spljunk_mRNA = (x_mRNA_unspl - x_mRNA)*(spl_mRNA + dk2_mRNA);

spljunk_rRNA = (x_rRNA_unspl - x_rRNA)*(spl_rRNA + dk2_rRNA);

spljunk_snRNA = (x_snRNA_unspl - x_snRNA)*(spl_snRNA + dk2_snRNA);

tRNA_synthesis_frac = x_tRNA*txs_tRNA/bind_NT_in_RNA;

unbind_AA = x_p*cut_p;

unbind_tRNA = x_p*txl_p;

unbind_AA_txl = unbind_tRNA;

unbind_AA_from_dk_tRNA = dk3_tRNA;

unbind_ADP = ATP_demand;

unbind_fats = ATP_frac_avail*k_unbind_fats*fats_in_mem_conc;

unbind_NT_RNA = x_mRNA*cut_mRNA + x_rRNA*cut_rRNA + x_snRNA*cut_snRNA...

+ x_tRNA*cut_tRNA + spljunk_mRNA + spljunk_rRNA...

+ spljunk_snRNA;

unbind_NT_DNA = 0;

% unbind_mRNA = unbind_rRNA*mRNA_in_use/(rRNA_in_use - mRNA_new_in_use);

% unbind_mRNA_new_in_use = 3*ER_p*mRNA_new_in_use/rRNA_spacing_on_mRNA;

% unbind_mRNA = 3*ER_p*mRNA_in_use/rRNA_spacing_on_mRNA;

% unbind_mRNA = 3*ER_p*mRNA_in_use/rRNA_spacing_on_mRNA + dk4_rRNA;

% unbind_mRNA_new_in_use = 0;

unbind_mRNA = unbind_rRNA*mRNA_in_use_conc/rRNA_in_use_conc;

unbind_p = ATP_frac_avail*k_restructure*p_in_use_conc;

unbind_snRNA = k_spl*snRNA_in_use_conc;

activate_mRNA = k_activate_mRNA*mRNA_inactive_conc*p_eIF_4_cytosol_conc;

% RNA polymerase

txs_mRNA_poly = ATP_frac_avail*k_mRNA_txs*polyII*poly_loci_frac...

*(RNA_poly_loci_constitutive + TF_grow_on_DNA);

% txs_mRNA_poly = ATP_frac_avail*k_mRNA_txs*polyII*poly_loci_frac...

% *RNA_poly_loci_constitutive;

spl_mRNA_poly = ATP_frac_avail*spl_frac*mRNA_poly_unspl;

nm_out_mRNA_poly = ATP_frac_avail*k_nm_mRNA*mRNA_poly_nuc_conc;

activate_mRNA_poly = activate_mRNA*mRNA_poly_inactive/mRNA_inactive;

bind_mRNA_poly = bind_mRNA*mRNA_poly_cytosol/mRNA_cytosol;

unbind_mRNA_poly = unbind_mRNA*mRNA_poly_in_use/mRNA_in_use;

% bind_mRNA_poly = eIF_4*k_bind_rRNA_mRNA*mRNA_poly_cytosol_conc*rRNA_cytosol_conc;

% unbind_mRNA_poly = 3*ER_p*mRNA_poly_in_use/rRNA_spacing_on_mRNA;

dk1_mRNA_poly = k_dk_mRNA*mRNA_poly_cytosol;

dk2_mRNA_poly = k_dk_mRNA*mRNA_poly_unspl;

dk3_mRNA_poly = k_dk_mRNA*mRNA_poly_nuc;

dk4_mRNA_poly = k_dk_mRNA*mRNA_poly_inactive;

dk5_mRNA_poly = k_dk_mRNA*mRNA_poly_in_use;

cut_mRNA_poly = k_cut_mRNA*mRNA_poly_trash;

txl_p_poly = unbind_rRNA*(mRNA_poly_cytosol + mRNA_poly_in_use)...

/(mRNA_cytosol + mRNA_in_use);

fold_p_poly = ATP_frac_avail*k_fold_p*p_poly_unfold_conc;

nm_in_p_poly = ATP_frac_avail*k_nm_in_p*p_poly_cytosol_conc;

activate_p_poly_by_mitogen = k_activate_p_poly_by_mitogen...

*p_poly_nuc_inactive_conc...

*(RNA_poly_active_constitutive...

+ mitogen_stimulation);

% activate_p_poly_by_mitogen = k_activate_p_poly_by_mitogen...

% *p_poly_nuc_inactive_conc...

% *mitogen_stimulation;

deactivate_p_poly_by_cycC_Cdk8 = k_deactivate_p_poly_by_cycC_Cdk8...

*cycC_Cdk8_active_conc...

*p_poly_nuc_active_conc;

% bind_p_poly = max(0, k_bind_p_poly...

% *min(i_poly_avail/size_ratio, p_poly_nuc_conc));

bind_p_poly_to_DNA = k_bind_p_poly_to_DNA*p_poly_nuc_active_conc;

% unbind_p_poly_from_DNA = txs_mRNA + txs_rRNA + txs_snRNA + txs_tRNA;

unbind_p_poly_from_DNA = txs_mRNA + txs_rRNA + txs_snRNA + txs_tRNA...

+ k_unbind_p_poly_from_DNA_by_APC_Cdc20...

*Cdc20_APC_active_conc*p_poly_in_use_conc;

u_p_poly_unfold = ATP_frac_avail*k_dk_p*p_poly_unfold;

u_p_poly_cytosol = ATP_frac_avail*k_dk_p*p_poly_cytosol;

u_p_poly_nuc_active = ATP_frac_avail*k_dk_p*p_poly_nuc_active;

u_p_poly_nuc_inactive = ATP_frac_avail*k_dk_p*p_poly_nuc_inactive;

u_p_poly_in_use = ATP_frac_avail*k_dk_p*p_poly_in_use;

cut_p_poly = ATP_frac_avail*k_cut_p*p_poly_trash_conc;

% eIF_4

txs_mRNA_eIF_4 = ATP_frac_avail*k_mRNA_txs*polyII*eIF_4_loci_frac;

spl_mRNA_eIF_4 = ATP_frac_avail*spl_frac*mRNA_eIF_4_unspl;

nm_out_mRNA_eIF_4 = ATP_frac_avail*k_nm_mRNA*mRNA_eIF_4_nuc_conc;

activate_mRNA_eIF_4 = activate_mRNA*mRNA_eIF_4_inactive/mRNA_inactive;

bind_mRNA_eIF_4 = bind_mRNA*mRNA_eIF_4_cytosol/mRNA_cytosol;

unbind_mRNA_eIF_4 = unbind_mRNA*mRNA_eIF_4_in_use/mRNA_in_use;

dk1_mRNA_eIF_4 = k_dk_mRNA*mRNA_eIF_4_cytosol;

dk2_mRNA_eIF_4 = k_dk_mRNA*mRNA_eIF_4_unspl;

dk3_mRNA_eIF_4 = k_dk_mRNA*mRNA_eIF_4_nuc;

dk4_mRNA_eIF_4 = k_dk_mRNA*mRNA_eIF_4_inactive;

dk5_mRNA_eIF_4 = k_dk_mRNA*mRNA_eIF_4_in_use;

cut_mRNA_eIF_4 = k_cut_mRNA*mRNA_eIF_4_trash;

txl_p_eIF_4 = unbind_rRNA*(mRNA_eIF_4_cytosol + mRNA_eIF_4_in_use)...

/(mRNA_cytosol + mRNA_in_use);

fold_p_eIF_4 = ATP_frac_avail*k_fold_p*p_eIF_4_unfold_conc;

% bind_p_eIF_4 = max(0, k_activate_mRNA*mRNA_cytosol_conc...

% *min(i_eIF_4_avail/size_ratio, p_eIF_4_cytosol_conc));

bind_p_eIF_4 = activate_mRNA;

unbind_p_eIF_4 = dk1_mRNA + dk4_mRNA;

u1_p_eIF_4 = ATP_frac_avail*k_dk_p*p_eIF_4_cytosol_conc;

u2_p_eIF_4 = ATP_frac_avail*k_dk_p*p_eIF_4_unfold_conc;

% u3_p_eIF_4 = ATP_frac_avail*k_dk_p*p_eIF_4_in_use_conc;

u3_p_eIF_4 = 0;

cut_p_eIF_4 = ATP_frac_avail*k_cut_p*p_eIF_4_trash_conc;

% ****************** calculate ratios to later correct for ubiq and decay of partner molecules

% for correction of ubiq and decay of p27 on cycD, cycE, and cycA

% (NOTE the idea here is to divide the released cyclins amongst the cyclins according to the

% fraction that is in the cyc_Cdk2_on_p27_inactive state for each cyclin)

u_p27_by_SCF_Skp2_fast = ATP_frac_avail*k_u_p27_by_SCF_Skp2_fast...

*Skp2_SCF_active_conc;

u_p27_by_APC_Cdc20 = ATP_frac_avail*k_u_p27_by_APC_Cdc20*Cdc20_APC_active_conc;

u_p27_by_KPC = ATP_frac_avail*k_u_p27_by_KPC*KPC_active_conc;

u_p27_fast = u_p27_by_SCF_Skp2_fast + u_p27_by_APC_Cdc20 + u_p27_by_KPC;

u_p27_on_cyclins = u_p27_fast*p27_on_cyclins_conc...

+ ATP_frac_avail*k_dk_p*p27_on_cyclins;

cyc_on_p27_tot = cycD_Cdk4or6_on_p27_inactive...

+ cycE_Cdk2_on_p27_inactive...

+ cycA_Cdk2_on_p27_inactive;

if(cyc_on_p27_tot <= 0E-9)

u_p27_on_cycD_Cdk4or6_frac = 0E-9;

u_p27_on_cycE_Cdk2_frac = 0E-9;

u_p27_on_cycA_Cdk2_frac = 0E-9;

else

u_p27_on_cycD_Cdk4or6_frac = u_p27_on_cyclins...

*cycD_Cdk4or6_on_p27_inactive/cyc_on_p27_tot;

u_p27_on_cycE_Cdk2_frac = u_p27_on_cyclins...

*cycE_Cdk2_on_p27_inactive/cyc_on_p27_tot;

u_p27_on_cycA_Cdk2_frac = u_p27_on_cyclins...

*cycA_Cdk2_on_p27_inactive/cyc_on_p27_tot;

end

% for correction of decay of Cdk2 on p27 (assume Cdk4or6 does not decay)

u_Cdk2_on_cycE = ATP_frac_avail*k_dk_p*Cdk2_on_cycE;

u_Cdk2_on_cycA = ATP_frac_avail*k_dk_p*Cdk2_on_cycA;

Cdk2_on_cycE_tot = cycE_Cdk2_inactive + cycE_Cdk2_active...

+ cycE_Cdk2_on_p27_inactive;

if(Cdk2_on_cycE_tot <= 0E-9)

u_Cdk2_on_cycE_p27 = 0E-9;

else

u_Cdk2_on_cycE_p27 = u_Cdk2_on_cycE*cycE_Cdk2_on_p27_inactive/Cdk2_on_cycE_tot;

end

Cdk2_on_cycA_tot = cycA_Cdk2_inactive + cycA_Cdk2_active...

+ cycA_Cdk2_on_p27_inactive;

if(Cdk2_on_cycA_tot <= 0E-9)

u_Cdk2_on_cycA_p27 = 0E-9;

else

u_Cdk2_on_cycA_p27 = u_Cdk2_on_cycA*cycA_Cdk2_on_p27_inactive/Cdk2_on_cycA_tot;

end

% for correction of decay of Cdk2 on cycE

if(Cdk2_on_cycE_tot <= 0E-9)

u_Cdk2_on_cycE_inactive_frac = 0E-9;

u_Cdk2_on_cycE_active_frac = 0E-9;

u_Cdk2_on_cycE_on_p27_inactive_frac = 0E-9;

else

u_Cdk2_on_cycE_inactive_frac = u_Cdk2_on_cycE...

*cycE_Cdk2_inactive/Cdk2_on_cycE_tot;

u_Cdk2_on_cycE_active_frac = u_Cdk2_on_cycE...

*cycE_Cdk2_active/Cdk2_on_cycE_tot;

u_Cdk2_on_cycE_on_p27_inactive_frac = u_Cdk2_on_cycE...

*cycE_Cdk2_on_p27_inactive/Cdk2_on_cycE_tot;

end

% for correction of decay of Cdk2 on cycA

if(Cdk2_on_cycA_tot <= 0E-9)

u_Cdk2_on_cycA_inactive_frac = 0E-9;

u_Cdk2_on_cycA_active_frac = 0E-9;

u_Cdk2_on_cycA_on_p27_inactive_frac = 0E-9;

else

u_Cdk2_on_cycA_inactive_frac = u_Cdk2_on_cycA...

*cycA_Cdk2_inactive/Cdk2_on_cycA_tot;

u_Cdk2_on_cycA_active_frac = u_Cdk2_on_cycA...

*cycA_Cdk2_active/Cdk2_on_cycA_tot;

u_Cdk2_on_cycA_on_p27_inactive_frac = u_Cdk2_on_cycA...

*cycA_Cdk2_on_p27_inactive/Cdk2_on_cycA_tot;

end

% for correction of decay of Cdk1 on cycA

u_Cdk1_on_cycB = ATP_frac_avail*k_dk_p*Cdk1_on_cycB;

u_Cdk1_on_cycA = ATP_frac_avail*k_dk_p*Cdk1_on_cycA;

Cdk1_on_cycA_tot = cycA_Cdk1_cytosol_active + cycA_Cdk1_phospho_inactive...

+ cycA_Cdk1_nuc_active;

if(Cdk1_on_cycA_tot <= 0E-9)

u_Cdk1_on_cycA_cytosol_active_frac = 0E-9;

u_Cdk1_on_cycA_phospho_inactive_frac = 0E-9;

u_Cdk1_on_cycA_nuc_active_frac = 0E-9;

else

u_Cdk1_on_cycA_cytosol_active_frac = u_Cdk1_on_cycA...

*cycA_Cdk1_cytosol_active...

/Cdk1_on_cycA_tot;

u_Cdk1_on_cycA_phospho_inactive_frac = u_Cdk1_on_cycA...

*cycA_Cdk1_phospho_inactive...

/Cdk1_on_cycA_tot;

u_Cdk1_on_cycA_nuc_active_frac = u_Cdk1_on_cycA...

*cycA_Cdk1_nuc_active/Cdk1_on_cycA_tot;

end

% for correction of decay of Cdk1 on cycB

Cdk1_on_cycB_tot = cycB_Cdk1_cytosol_active + cycB_Cdk1_phospho_inactive...

+ cycB_Cdk1_nuc_active;

if(Cdk1_on_cycB_tot <= 0E-9)

u_Cdk1_on_cycB_cytosol_active_frac = 0E-9;

u_Cdk1_on_cycB_phospho_inactive_frac = 0E-9;

u_Cdk1_on_cycB_nuc_active_frac = 0E-9;

else

u_Cdk1_on_cycB_cytosol_active_frac = u_Cdk1_on_cycB...

*cycB_Cdk1_cytosol_active...

/Cdk1_on_cycB_tot;

u_Cdk1_on_cycB_phospho_inactive_frac = u_Cdk1_on_cycB...

*cycB_Cdk1_phospho_inactive...

/Cdk1_on_cycB_tot;

u_Cdk1_on_cycB_nuc_active_frac = u_Cdk1_on_cycB...

*cycB_Cdk1_nuc_active...

/Cdk1_on_cycB_tot;

end

% for correction of decay of APC on Cdc20

u_APC_on_Cdc20 = ATP_frac_avail*k_dk_p*APC_on_Cdc20;

APC_on_Cdc20_tot = Cdc20_APC_inactive + Cdc20_APC_active;

if(APC_on_Cdc20_tot <= 0E-9)

u_APC_on_Cdc20_inactive_frac = 0E-9;

u_APC_on_Cdc20_active_frac = 0E-9;

else

u_APC_on_Cdc20_inactive_frac = u_APC_on_Cdc20...

*Cdc20_APC_inactive/APC_on_Cdc20_tot;

u_APC_on_Cdc20_active_frac = u_APC_on_Cdc20...

*Cdc20_APC_active/APC_on_Cdc20_tot;

end

% 2 Rb RATES

txs_mRNA_Rb = ATP_frac_avail*k_mRNA_txs*polyII*Rb_loci_frac...

/max(1, E2F_on_DNA);

spl_mRNA_Rb = ATP_frac_avail*spl_frac*mRNA_Rb_unspl;

nm_out_mRNA_Rb = ATP_frac_avail*k_nm_mRNA*mRNA_Rb_nuc_conc;

activate_mRNA_Rb = activate_mRNA*mRNA_Rb_inactive/mRNA_inactive;

bind_mRNA_Rb = bind_mRNA*mRNA_Rb_cytosol/mRNA_cytosol;

unbind_mRNA_Rb = unbind_mRNA*mRNA_Rb_in_use/mRNA_in_use;

dk_mRNA_Rb_cytosol = k_dk_mRNA*mRNA_Rb_cytosol;

dk_mRNA_Rb_unspl = k_dk_mRNA*mRNA_Rb_unspl;

dk_mRNA_Rb_nuc = k_dk_mRNA*mRNA_Rb_nuc;

dk_mRNA_Rb_inactive = k_dk_mRNA*mRNA_Rb_inactive;

dk_mRNA_Rb_in_use = k_dk_mRNA*mRNA_Rb_in_use;

cut_mRNA_Rb = k_cut_mRNA*mRNA_Rb_trash;

txl_Rb = unbind_rRNA*(mRNA_Rb_cytosol + mRNA_Rb_in_use)...

/(mRNA_cytosol + mRNA_in_use);

fold_Rb = ATP_frac_avail*k_fold_p*Rb_unfold_conc;

nm_in_Rb = ATP_frac_avail*k_nm_in_p*Rb_cytosol_conc;

bind_Rb_to_E2F = k_bind_Rb_to_E2F*Rb_nuc_conc*E2F_nuc_conc;

unbind_Rb_E2F = k_unbind_Rb_E2F*Rb_on_E2F_conc;

% cyclins phosphorylate Rb

Cdk_active_conc = cycD_Cdk4or6_active_conc...

+ cycD_Cdk4or6_on_p27_inactive...

+ cycE_Cdk2_active_conc + cycA_Cdk2_active_conc...

+ cycA_Cdk1_nuc_active_conc + cycB_Cdk1_nuc_active_conc;

phospho_Rb_nuc_by_Cdk = k_phospho_Rb_by_Cdk*Cdk_active_conc*Rb_nuc_conc;

phospho_Rb_on_E2F_by_Cdk = k_phospho_Rb_by_Cdk*Cdk_active_conc*Rb_on_E2F_conc;

% cyclins keep Rb phosphorylated

unphospho_Rb = k_unphospho_Rb*Rb_phospho_inactive_conc...

+ k_dephospho_Rb_by_Cdc14*Rb_phospho_inactive_conc...

*Cdc14_phospho_active_conc;

u_Rb_unfold = ATP_frac_avail*k_dk_p*Rb_unfold;

u_Rb_cytosol = ATP_frac_avail*k_dk_p*Rb_cytosol;

u_Rb_nuc = ATP_frac_avail*k_dk_p*Rb_nuc;

u_Rb_on_E2F = ATP_frac_avail*k_dk_p*Rb_on_E2F;

u_Rb_phospho_inactive = ATP_frac_avail*k_dk_p*Rb_phospho_inactive;

cut_Rb = ATP_frac_avail*k_cut_p*Rb_trash_conc;

% 3 cycD RATES

% mitogen_stimulation = 0 or 1; --> see define_const.in and makedynfiles.f

% txs_mRNA_cycD = ATP_frac_avail*k_mRNA_txs*polyII*cycD_loci_frac...

% *mitogen_stimulation;

% txs_mRNA_cycD = ATP_frac_avail*k_mRNA_txs*polyII*cycD_loci_frac...

% *mitogen_stimulation*B_Myb_on_DNA;

txs_mRNA_cycD = ATP_frac_avail*k_mRNA_txs*polyII*cycD_loci_frac...

*mitogen_stimulation...

*(cycD_constitutive + B_Myb_on_DNA);

spl_mRNA_cycD = ATP_frac_avail*spl_frac*mRNA_cycD_unspl;

nm_out_mRNA_cycD = ATP_frac_avail*k_nm_mRNA*mRNA_cycD_nuc_conc;

activate_mRNA_cycD = activate_mRNA*mRNA_cycD_inactive/mRNA_inactive;

bind_mRNA_cycD = bind_mRNA*mRNA_cycD_cytosol/mRNA_cytosol;

unbind_mRNA_cycD = unbind_mRNA*mRNA_cycD_in_use/mRNA_in_use;

dk_mRNA_cycD_cytosol = k_dk_mRNA*mRNA_cycD_cytosol;

dk_mRNA_cycD_unspl = k_dk_mRNA*mRNA_cycD_unspl;

dk_mRNA_cycD_nuc = k_dk_mRNA*mRNA_cycD_nuc;

dk_mRNA_cycD_inactive = k_dk_mRNA*mRNA_cycD_inactive;

dk_mRNA_cycD_in_use = k_dk_mRNA*mRNA_cycD_in_use;

cut_mRNA_cycD = k_cut_mRNA*mRNA_cycD_trash;

txl_cycD = unbind_rRNA*(mRNA_cycD_cytosol + mRNA_cycD_in_use)...

/(mRNA_cytosol + mRNA_in_use);

fold_cycD = ATP_frac_avail*k_fold_p*cycD_unfold_conc;

nm_in_cycD = ATP_frac_avail*k_nm_in_p*cycD_cytosol_conc;

bind_cycD_to_Cdk4or6 = k_bind_cycD_to_Cdk4or6*cycD_nuc_conc;

% note -- I originally had unbinding due to Cdk4or6 decay

% unbind_cycD_Cdk4or6 = ATP_frac_avail*k_dk_p*cycD_Cdk4or6_active;

unbind_cycD_Cdk4or6 = k_unbind_cycD_Cdk4or6*cycD_Cdk4or6_active;

% ASSUME p27 does not inhibit cycD/Cdk4or6, so I set these rate constants to 0

bind_cycD_Cdk4or6_to_p27 = k_bind_cycD_Cdk4or6_to_p27*cycD_Cdk4or6_active_conc...

*p27_nuc_conc;

unbind_cycD_Cdk4or6_p27 = k_unbind_cycD_Cdk4or6_p27...

*cycD_Cdk4or6_on_p27_inactive_conc;

u_cycD_by_SCF_Skp2 = ATP_frac_avail*k_u_cycD_by_SCF_Skp2*Skp2_SCF_active_conc;

% some "other" ubiquinates cycD -- ASSUME it is Cdc20

u_cycD_by_APC_Cdc20 = ATP_frac_avail*k_u_cycD_by_APC_Cdc20...

*Cdc20_APC_active_conc;

u_cycD = u_cycD_by_SCF_Skp2 + u_cycD_by_APC_Cdc20;

u_cycD_unfold = ATP_frac_avail*k_dk_p*cycD_unfold;

u_cycD_cytosol = ATP_frac_avail*k_dk_p*cycD_cytosol;

u_cycD_nuc = u_cycD*cycD_nuc_conc...

+ ATP_frac_avail*k_dk_p*cycD_nuc;

u_cycD_Cdk4or6_active = u_cycD*cycD_Cdk4or6_active_conc...

+ ATP_frac_avail*k_dk_p*cycD_Cdk4or6_active;

u_cycD_Cdk4or6_on_p27_inactive = u_cycD...

*cycD_Cdk4or6_on_p27_inactive_conc...

+ ATP_frac_avail...

*k_dk_p*cycD_Cdk4or6_on_p27_inactive;

cut_cycD = ATP_frac_avail*k_cut_p*cycD_trash_conc;

cycD_conc_all = cycD_nuc_conc + cycD_Cdk4or6_active_conc...

+ cycD_Cdk4or6_on_p27_inactive_conc;

energy_u_cycD_by_SCF_Skp2 = ATP_u*u_cycD_by_SCF_Skp2*cycD_conc_all;

% energy_u_cycD_by_APC_Cdh1 = ATP_u*u_cycD_by_APC_Cdh1*cycD_conc_all;

energy_u_cycD_by_APC_Cdc20 = ATP_u*u_cycD_by_APC_Cdc20*cycD_conc_all;

% 5 cycE RATES

txs_mRNA_cycE = ATP_frac_avail*k_mRNA_txs*polyII*cycE_loci_frac*E2F_on_DNA;

spl_mRNA_cycE = ATP_frac_avail*spl_frac*mRNA_cycE_unspl;

nm_out_mRNA_cycE = ATP_frac_avail*k_nm_mRNA*mRNA_cycE_nuc_conc;

activate_mRNA_cycE = activate_mRNA*mRNA_cycE_inactive/mRNA_inactive;

bind_mRNA_cycE = bind_mRNA*mRNA_cycE_cytosol/mRNA_cytosol;

unbind_mRNA_cycE = unbind_mRNA*mRNA_cycE_in_use/mRNA_in_use;

dk_mRNA_cycE_cytosol = k_dk_mRNA*mRNA_cycE_cytosol;

dk_mRNA_cycE_unspl = k_dk_mRNA*mRNA_cycE_unspl;

dk_mRNA_cycE_nuc = k_dk_mRNA*mRNA_cycE_nuc;

dk_mRNA_cycE_inactive = k_dk_mRNA*mRNA_cycE_inactive;

dk_mRNA_cycE_in_use = k_dk_mRNA*mRNA_cycE_in_use;

cut_mRNA_cycE = k_cut_mRNA*mRNA_cycE_trash;

txl_cycE = unbind_rRNA*(mRNA_cycE_cytosol + mRNA_cycE_in_use)...

/(mRNA_cytosol + mRNA_in_use);

fold_cycE = ATP_frac_avail*k_fold_p*cycE_unfold_conc;

nm_in_cycE = ATP_frac_avail*k_nm_in_p*cycE_cytosol_conc;

bind_cycE_to_Cdk2 = k_bind_cycE_to_Cdk2*cycE_nuc_conc*Cdk2_nuc_conc;

unbind_cycE_Cdk2 = k_unbind_cycE_Cdk2*cycE_Cdk2_inactive_conc;

% cycE/Cdk2 is activated (dephospohorylated) by Cdc25A

% (any Cdc25, but only Cdc25A is nuclear at this time)

dephospho_cycE_Cdk2 = k_dephospho_cycE_Cdk2*cycE_Cdk2_inactive_conc;

dephospho_cycE_Cdk2_by_Cdc25A = k_dephospho_cycE_Cdk2_by_Cdc25A*cycE_Cdk2_inactive_conc...

*Cdc25A_phospho_active_conc;

phospho_cycE_Cdk2 = k_phospho_cycE_Cdk2*cycE_Cdk2_active_conc;

% cycE/Cdk2 is inhibited by p27

% note that unbinding p27 entails phospho of p27, which is assumed to be automatic by Cdk2

bind_cycE_Cdk2_to_p27 = k_bind_cycE_Cdk2_to_p27*p27_nuc_conc...

*cycE_Cdk2_inactive_conc;

unbind_cycE_Cdk2_p27 = k_unbind_cycE_Cdk2_p27*cycE_Cdk2_on_p27_inactive_conc;

% cycE/Cdk2 is ubiq by SCF(Fbw7); cycE free (in nucleus) is ubiq by SCF(Skp2)

u_cycE_by_SCF_Skp2 = ATP_frac_avail*k_u_cycE_by_SCF_Skp2*Skp2_SCF_active_conc;

u_cycE_by_SCF_Fbw7 = ATP_frac_avail*k_u_cycE_by_SCF_Fbw7*Fbw7_SCF_active_conc;

u_cycE_unfold = ATP_frac_avail*k_dk_p*cycE_unfold;

u_cycE_cytosol = ATP_frac_avail*k_dk_p*cycE_cytosol;

u_cycE_nuc = u_cycE_by_SCF_Skp2*cycE_nuc_conc...

+ ATP_frac_avail*k_dk_p*cycE_nuc;

u_cycE_Cdk2_inactive = u_cycE_by_SCF_Fbw7*cycE_Cdk2_inactive_conc...

+ ATP_frac_avail*k_dk_p*cycE_Cdk2_inactive;

u_cycE_Cdk2_active = u_cycE_by_SCF_Fbw7*cycE_Cdk2_active_conc...

+ ATP_frac_avail*k_dk_p*cycE_Cdk2_active;

u_cycE_Cdk2_on_p27_inactive = u_cycE_by_SCF_Fbw7*cycE_Cdk2_on_p27_inactive_conc...

+ ATP_frac_avail*k_dk_p*cycE_Cdk2_on_p27_inactive;

cut_cycE = ATP_frac_avail*k_cut_p*cycE_trash_conc;

u_cycE_by_SCF_Skp2_all = u_cycE_by_SCF_Skp2*cycE_nuc_conc;

u_cycE_by_SCF_Fbw7_all = u_cycE_by_SCF_Fbw7...

*(cycE_Cdk2_inactive_conc + cycE_Cdk2_active_conc...

+ cycE_Cdk2_on_p27_inactive_conc);

energy_u_cycE_by_SCF_Skp2 = ATP_u*u_cycE_by_SCF_Skp2_all;

energy_u_cycE_by_SCF_Fbw7 = ATP_u*u_cycE_by_SCF_Fbw7_all;

% 6 B-Myb RATES

% log10_B_Myb_on_DNA = log10(max(10, B_Myb_on_DNA));

% txs_mRNA_B_Myb = ATP_frac_avail*k_mRNA_txs*polyII*B_Myb_loci_frac...

% *E2F_on_DNA*log10_B_Myb_on_DNA;

txs_mRNA_B_Myb = ATP_frac_avail*k_mRNA_txs*polyII*B_Myb_loci_frac...

*(E2F_on_DNA + B_Myb_on_DNA);

spl_mRNA_B_Myb = ATP_frac_avail*spl_frac*mRNA_B_Myb_unspl;

nm_out_mRNA_B_Myb = ATP_frac_avail*k_nm_mRNA*mRNA_B_Myb_nuc_conc;

activate_mRNA_B_Myb = activate_mRNA*mRNA_B_Myb_inactive/mRNA_inactive;

bind_mRNA_B_Myb = bind_mRNA*mRNA_B_Myb_cytosol/mRNA_cytosol;

unbind_mRNA_B_Myb = unbind_mRNA*mRNA_B_Myb_in_use/mRNA_in_use;

dk_mRNA_B_Myb_cytosol = k_dk_mRNA*mRNA_B_Myb_cytosol;

dk_mRNA_B_Myb_unspl = k_dk_mRNA*mRNA_B_Myb_unspl;

dk_mRNA_B_Myb_nuc = k_dk_mRNA*mRNA_B_Myb_nuc;

dk_mRNA_B_Myb_inactive = k_dk_mRNA*mRNA_B_Myb_inactive;

dk_mRNA_B_Myb_in_use = k_dk_mRNA*mRNA_B_Myb_in_use;

cut_mRNA_B_Myb = k_cut_mRNA*mRNA_B_Myb_trash;

txl_B_Myb = unbind_rRNA*(mRNA_B_Myb_cytosol + mRNA_B_Myb_in_use)...

/(mRNA_cytosol + mRNA_in_use);

fold_B_Myb = ATP_frac_avail*k_fold_p*B_Myb_unfold_conc;

nm_in_B_Myb = ATP_frac_avail*k_nm_in_p*B_Myb_cytosol_conc;

Cdk2_active_conc = cycE_Cdk2_active_conc + cycA_Cdk2_active_conc;

phospho_B_Myb_by_cyc_Cdk = k_phospho_B_Myb_by_cyc_Cdk*B_Myb_nuc_conc...

*Cdk2_active_conc;

unphospho_B_Myb = 0;

% bind_B_Myb_to_DNA = k_bind_B_Myb_to_DNA*B_Myb_phospho_active_conc;

bind_B_Myb_to_DNA = min(k_bind_B_Myb_to_DNA*B_Myb_phospho_active_conc, ...

k_bind_B_Myb_to_DNA*max(0, sat_B_Myb_on_DNA - B_Myb_on_DNA));

% unbind_B_Myb_from_DNA = k_unbind_B_Myb_from_DNA*B_Myb_on_DNA_conc;

unbind_B_Myb_from_DNA = k_unbind_B_Myb_from_DNA*B_Myb_on_DNA;

u_B_Myb_by_SCF_Skp2 = ATP_frac_avail*k_u_B_Myb_by_SCF_Skp2*Skp2_SCF_active_conc;

u_B_Myb_unfold = ATP_frac_avail*k_dk_p*B_Myb_unfold;

u_B_Myb_cytosol = ATP_frac_avail*k_dk_p*B_Myb_cytosol;

u_B_Myb_nuc = u_B_Myb_by_SCF_Skp2*B_Myb_nuc_conc...

+ ATP_frac_avail*k_dk_p*B_Myb_nuc;

u_B_Myb_phospho_active = u_B_Myb_by_SCF_Skp2*B_Myb_phospho_active_conc...

+ ATP_frac_avail*k_dk_p*B_Myb_phospho_active;

% ASSUME no ubiq of B-Myb while it is on DNA

u_B_Myb_on_DNA = ATP_frac_avail*k_dk_p*B_Myb_on_DNA;

cut_B_Myb = ATP_frac_avail*k_cut_p*B_Myb_trash_conc;

energy_u_B_Myb_by_SCF_Skp2 = ATP_u*u_B_Myb_by_SCF_Skp2...

*(B_Myb_nuc_conc + B_Myb_phospho_active_conc);

% 7 NF-Y RATES

txs_mRNA_NF_Y = ATP_frac_avail*k_mRNA_txs*polyII*NF_Y_loci_frac...

*(NF_Y_constitutive + E2F_on_DNA);

spl_mRNA_NF_Y = ATP_frac_avail*spl_frac*mRNA_NF_Y_unspl;

nm_out_mRNA_NF_Y = ATP_frac_avail*k_nm_mRNA*mRNA_NF_Y_nuc_conc;

activate_mRNA_NF_Y = activate_mRNA*mRNA_NF_Y_inactive/mRNA_inactive;

bind_mRNA_NF_Y = bind_mRNA*mRNA_NF_Y_cytosol/mRNA_cytosol;

unbind_mRNA_NF_Y = unbind_mRNA*mRNA_NF_Y_in_use/mRNA_in_use;

dk_mRNA_NF_Y_cytosol = k_dk_mRNA*mRNA_NF_Y_cytosol;

dk_mRNA_NF_Y_unspl = k_dk_mRNA*mRNA_NF_Y_unspl;

dk_mRNA_NF_Y_nuc = k_dk_mRNA*mRNA_NF_Y_nuc;

dk_mRNA_NF_Y_inactive = k_dk_mRNA*mRNA_NF_Y_inactive;

dk_mRNA_NF_Y_in_use = k_dk_mRNA*mRNA_NF_Y_in_use;

cut_mRNA_NF_Y = k_cut_mRNA*mRNA_NF_Y_trash;

txl_NF_Y = unbind_rRNA*(mRNA_NF_Y_cytosol + mRNA_NF_Y_in_use)...

/(mRNA_cytosol + mRNA_in_use);

fold_NF_Y = ATP_frac_avail*k_fold_p*NF_Y_unfold_conc;

nm_in_NF_Y = ATP_frac_avail*k_nm_in_p*NF_Y_cytosol_conc;

Cdk2_active_conc = cycE_Cdk2_active_conc + cycA_Cdk2_active_conc;

phospho_NF_Y_by_cyc_Cdk = k_phospho_NF_Y_by_cyc_Cdk*NF_Y_nuc_conc...

*Cdk2_active_conc;

unphospho_NF_Y = k_unphospho_NF_Y*NF_Y_phospho_active_conc;

dephospho_NF_Y_by_Cdc14 = k_dephospho_NF_Y_by_Cdc14*Cdc14_phospho_active_conc...

*NF_Y_phospho_active_conc;

% bind_NF_Y_to_DNA = k_bind_NF_Y_to_DNA*NF_Y_phospho_active_conc;

bind_NF_Y_to_DNA = min(k_bind_NF_Y_to_DNA*NF_Y_phospho_active_conc, ...

k_bind_NF_Y_to_DNA*max(0, sat_NF_Y_on_DNA - NF_Y_on_DNA));

unbind_NF_Y_from_DNA = k_unbind_NF_Y_from_DNA*NF_Y_on_DNA_conc;

% NOTE -- NF-Y probably should be ubiquinated by SCF(Btrc) or APC(Cdc20)

u_NF_Y_unfold = ATP_frac_avail*k_dk_p*NF_Y_unfold;

u_NF_Y_cytosol = ATP_frac_avail*k_dk_p*NF_Y_cytosol;

u_NF_Y_nuc = ATP_frac_avail*k_dk_p*NF_Y_nuc;

u_NF_Y_phospho_active = ATP_frac_avail*k_dk_p*NF_Y_phospho_active;

u_NF_Y_on_DNA = ATP_frac_avail*k_dk_p*NF_Y_on_DNA;

cut_NF_Y = ATP_frac_avail*k_cut_p*NF_Y_trash_conc;

% 8 E2F RATES

txs_mRNA_E2F = ATP_frac_avail*k_mRNA_txs*polyII*E2F_loci_frac...

*(E2F_constituitive + E2F_on_DNA);

% /max(1, B_Myb_on_DNA);

spl_mRNA_E2F = ATP_frac_avail*spl_frac*mRNA_E2F_unspl;

nm_out_mRNA_E2F = ATP_frac_avail*k_nm_mRNA*mRNA_E2F_nuc_conc;

activate_mRNA_E2F = activate_mRNA*mRNA_E2F_inactive/mRNA_inactive;

bind_mRNA_E2F = bind_mRNA*mRNA_E2F_cytosol/mRNA_cytosol;

unbind_mRNA_E2F = unbind_mRNA*mRNA_E2F_in_use/mRNA_in_use;

dk_mRNA_E2F_cytosol = k_dk_mRNA*mRNA_E2F_cytosol;

dk_mRNA_E2F_unspl = k_dk_mRNA*mRNA_E2F_unspl;

dk_mRNA_E2F_nuc = k_dk_mRNA*mRNA_E2F_nuc;

dk_mRNA_E2F_inactive = k_dk_mRNA*mRNA_E2F_inactive;

dk_mRNA_E2F_in_use = k_dk_mRNA*mRNA_E2F_in_use;

cut_mRNA_E2F = k_cut_mRNA*mRNA_E2F_trash;

txl_E2F = unbind_rRNA*(mRNA_E2F_cytosol + mRNA_E2F_in_use)...

/(mRNA_cytosol + mRNA_in_use);

fold_E2F = ATP_frac_avail*k_fold_p*E2F_unfold_conc;

nm_in_E2F = ATP_frac_avail*k_nm_in_p*E2F_cytosol_conc;

bind_E2F_to_DNA = min(k_bind_E2F_to_DNA*E2F_nuc_conc, ...

k_bind_E2F_to_DNA*max(0, sat_E2F_on_DNA - E2F_on_DNA));

% Cdk_all_conc = cycA_Cdk2_active_conc...

% + cycA_Cdk1_nuc_active_conc + cycB_Cdk1_nuc_active_conc;

% unbind_E2F_DNA = k_E2F_phospho_by_cyc_Cdk*Cdk_all_conc*E2F_on_DNA_conc;

% unbind_E2F_DNA = k_E2F_phospho_by_cyc_Cdk*Cdk_all_conc*E2F_on_DNA;

% unbind_E2F_DNA = (k_E2F_phospho_by_cyc_Cdk*Cdk_all_conc...

% + k_E2F_phospho_by_cycD_Cdk4or6*cycD_Cdk4or6_active...

% + k_unbind_E2F_DNA)...

% *E2F_on_DNA;

unbind_E2F_DNA = (k_E2F_phospho_by_cycA_Cdk2*cycA_Cdk2_active_conc...

+ k_E2F_phospho_by_cycA_Cdk1*cycA_Cdk1_nuc_active_conc...

+ k_E2F_phospho_by_cycB_Cdk1*cycB_Cdk1_nuc_active_conc...

+ k_E2F_phospho_by_cycD_Cdk4or6*cycD_Cdk4or6_active...

+ k_unbind_E2F_DNA)...

*E2F_on_DNA;

bind_E2F_to_Rb = bind_Rb_to_E2F;

unbind_E2F_Rb = unbind_Rb_E2F + phospho_Rb_on_E2F_by_Cdk;

% E2F is ubiq by SCF(Skp2) (in nucleus) and "other"

% ASSUME (although it is probably not needed) SCF(Skp2) does not ubiq E2F_on_DNA

% ASSUME "other" is APC(Cdc20)

u_E2F_by_SCF_Skp2 = ATP_frac_avail*k_u_E2F_by_SCF_Skp2*Skp2_SCF_active_conc;

u_E2F_by_APC_Cdc20 = ATP_frac_avail*k_u_E2F_by_APC_Cdc20*Cdc20_APC_active_conc;

u_E2F = u_E2F_by_SCF_Skp2 + u_E2F_by_APC_Cdc20;

u_E2F_unfold = ATP_frac_avail*k_dk_p*E2F_unfold;

u_E2F_cytosol = ATP_frac_avail*k_dk_p*E2F_cytosol;

u_E2F_nuc = u_E2F*E2F_nuc_conc...

+ ATP_frac_avail*k_dk_p*E2F_nuc;

u_E2F_on_DNA = u_E2F_by_APC_Cdc20*E2F_on_DNA_conc...

+ ATP_frac_avail*k_dk_p*E2F_on_DNA;

u_E2F_Rb_inactive = u_E2F*E2F_Rb_inactive_conc...

+ ATP_frac_avail*k_dk_p*E2F_Rb_inactive;

cut_E2F = ATP_frac_avail*k_cut_p*E2F_trash_conc;

E2F_conc_some = E2F_nuc_conc + E2F_Rb_inactive_conc;

E2F_conc_all = E2F_nuc_conc + E2F_on_DNA_conc...

+ E2F_Rb_inactive_conc;

energy_u_E2F_by_SCF_Skp2 = ATP_u*u_E2F_by_SCF_Skp2*E2F_conc_some;

energy_u_E2F_by_ACP_Cdc20 = ATP_u*u_E2F_by_APC_Cdc20*E2F_conc_all;

% 9 cycA RATES

% adhesion_factor = 0 or 1; --> see define_const.in and makedynfiles.f

txs_mRNA_cycA = ATP_frac_avail*k_mRNA_txs*polyII*cycA_loci_frac...

*(mitogen_stimulation + adhesion_factor)...

*(E2F_on_DNA + NF_Y_on_DNA);

spl_mRNA_cycA = ATP_frac_avail*spl_frac*mRNA_cycA_unspl;

nm_out_mRNA_cycA = ATP_frac_avail*k_nm_mRNA*mRNA_cycA_nuc_conc;

activate_mRNA_cycA = activate_mRNA*mRNA_cycA_inactive/mRNA_inactive;

bind_mRNA_cycA = bind_mRNA*mRNA_cycA_cytosol/mRNA_cytosol;

unbind_mRNA_cycA = unbind_mRNA*mRNA_cycA_in_use/mRNA_in_use;

dk_mRNA_cycA_cytosol = k_dk_mRNA*mRNA_cycA_cytosol;

dk_mRNA_cycA_unspl = k_dk_mRNA*mRNA_cycA_unspl;

dk_mRNA_cycA_nuc = k_dk_mRNA*mRNA_cycA_nuc;

dk_mRNA_cycA_inactive = k_dk_mRNA*mRNA_cycA_inactive;

dk_mRNA_cycA_in_use = k_dk_mRNA*mRNA_cycA_in_use;

cut_mRNA_cycA = k_cut_mRNA*mRNA_cycA_trash;

txl_cycA = unbind_rRNA*(mRNA_cycA_cytosol + mRNA_cycA_in_use)...

/(mRNA_cytosol + mRNA_in_use);

fold_cycA = ATP_frac_avail*k_fold_p*cycA_unfold_conc;

nm_in_cycA = ATP_frac_avail*k_nm_in_p*cycA_cytosol_conc;

bind_cycA_to_Cdk2 = k_bind_cycA_to_Cdk2*cycA_nuc_conc*Cdk2_nuc_conc;

unbind_cycA_Cdk2 = k_unbind_cycA_Cdk2*cycA_Cdk2_inactive_conc;

dephospho_cycA_Cdk2_by_Cdc25A = k_dephospho_cycA_Cdk2_by_Cdc25A...

*cycA_Cdk2_inactive_conc...

*Cdc25A_phospho_active_conc;

phospho_cycA_Cdk2 = k_phospho_cycA_Cdk2*cycA_Cdk2_active_conc;

bind_cycA_Cdk2_to_p27 = k_bind_cycA_Cdk2_to_p27...

*(p27_nuc_conc/max(1, Skp2_nuc_conc))...

*cycA_Cdk2_inactive_conc;

unbind_cycA_Cdk2_p27 = k_unbind_cycA_Cdk2_p27*cycA_Cdk2_on_p27_inactive_conc;

bind_cycA_to_Cdk1 = k_bind_cycA_to_Cdk1*cycA_nuc_conc...

*Cdk1_in_cell_conc;

unbind_cycA_Cdk1 = k_unbind_cycA_Cdk1*cycA_Cdk1_cytosol_active_conc;

phospho_cycA_Cdk1_by_Wee1 = k_phospho_cycA_Cdk1_by_Wee1...

*Wee1_cytosol_active_conc*cycA_Cdk1_cytosol_active_conc...

+ k_phospho_cycA_Cdk1_by_phospho_Wee1...

*Wee1_phospho_inactive_conc*cycA_Cdk1_cytosol_active_conc;

unphospho_cycA_Cdk1_from_Wee1 = k_unphospho_cycA_Cdk1_from_Wee1...

*cycA_Cdk1_phospho_inactive_conc;

% cycA/Cdk1 is activated when dephosphorylated by Cdc25B or Cdc25C in cytosol

dephospho_cycA_Cdk1_by_Cdc25 = (k_dephospho_cycA_Cdk1_by_Cdc25A...

*Cdc25A_phospho_active_conc...

+ k_dephospho_cycA_Cdk1_by_Cdc25B...

*Cdc25B_cytosol_phospho_active_conc...

+ k_dephospho_cycA_Cdk1_by_Cdc25C...

*Cdc25C_cytosol_phospho_active_conc)...

*cycA_Cdk1_phospho_inactive_conc;

nm_in_cycA_Cdk1 = ATP_frac_avail*k_nm_in_cycA_Cdk1_by_Plk1...

*Plk1_cytosol_phospho_active_conc...

*cycA_Cdk1_cytosol_active_conc;

nm_out_cycA_Cdk1 = k_nm_out_cycA_Cdk1*cycA_Cdk1_nuc_active_conc;

% ASSUME SCF(Skp2) and APC(Cdc20) are nuclear

% ASSUME APC(Cdh1) is cellular

% NOTE -- Skp2 binds with cycA to protect it from p27 (Ji et al., 2006)

u_cycA_by_SCF_Skp2 = ATP_frac_avail*k_u_cycA_by_SCF_Skp2*Skp2_SCF_active_conc;

u_cycA_by_APC_Cdh1 = ATP_frac_avail*k_u_cycA_by_APC_Cdh1*Cdh1_APC_active_conc;

u_cycA_by_APC_Cdc20 = ATP_frac_avail*k_u_cycA_by_APC_Cdc20*Cdc20_APC_active_conc;

u_cycA = u_cycA_by_SCF_Skp2 + u_cycA_by_APC_Cdh1...

+ u_cycA_by_APC_Cdc20;

u_cycA_unfold = ATP_frac_avail*k_dk_p*cycA_unfold;

u_cycA_cytosol = u_cycA_by_APC_Cdh1*cycA_cytosol...

+ ATP_frac_avail*k_dk_p*cycA_cytosol;

u_cycA_Cdk1_cytosol_active = u_cycA_by_APC_Cdh1*cycA_Cdk1_cytosol_active_conc...

+ ATP_frac_avail*k_dk_p*cycA_Cdk1_cytosol_active;

u_cycA_Cdk1_phospho_inactive = u_cycA_by_APC_Cdh1*cycA_Cdk1_phospho_inactive_conc...

+ ATP_frac_avail*k_dk_p*cycA_Cdk1_phospho_inactive;

u_cycA_Cdk1_nuc_active = u_cycA*cycA_Cdk1_nuc_active_conc...

+ ATP_frac_avail*k_dk_p*cycA_Cdk1_nuc_active;

u_cycA_nuc = u_cycA*cycA_nuc_conc...

+ ATP_frac_avail*k_dk_p*cycA_nuc;

u_cycA_Cdk2_inactive = u_cycA*cycA_Cdk2_inactive_conc...

+ ATP_frac_avail*k_dk_p*cycA_Cdk2_inactive;

u_cycA_Cdk2_active = u_cycA*cycA_Cdk2_active_conc...

+ ATP_frac_avail*k_dk_p*cycA_Cdk2_active;

u_cycA_Cdk2_on_p27_inactive = u_cycA*cycA_Cdk2_on_p27_inactive_conc...

+ ATP_frac_avail*k_dk_p*cycA_Cdk2_on_p27_inactive;

cut_cycA = ATP_frac_avail*k_cut_p*cycA_trash_conc;

cycA_conc_all = cycA_nuc_conc...

+ cycA_Cdk2_inactive_conc + cycA_Cdk2_active_conc...

+ cycA_Cdk2_on_p27_inactive_conc...

+ cycA_Cdk1_nuc_active_conc;

energy_u_cycA_by_SCF_Skp2 = ATP_u*u_cycA_by_SCF_Skp2*cycA_conc_all;

energy_u_cycA_by_APC_Cdh1 = ATP_u*u_cycA_by_APC_Cdh1...

*(cycA_conc_all + cycA_cytosol_conc...

+ cycA_Cdk1_cytosol_active_conc...

+ cycA_Cdk1_phospho_inactive_conc);

energy_u_cycA_by_APC_Cdc20 = ATP_u*u_cycA_by_APC_Cdc20*cycA_conc_all;

% 1 p27 RATES

txs_mRNA_p27 = ATP_frac_avail*k_mRNA_txs*polyII*p27_loci_frac;

spl_mRNA_p27 = ATP_frac_avail*spl_frac*mRNA_p27_unspl;

nm_out_mRNA_p27 = ATP_frac_avail*k_nm_mRNA*mRNA_p27_nuc_conc;

activate_mRNA_p27 = activate_mRNA*mRNA_p27_inactive/mRNA_inactive;

bind_mRNA_p27 = bind_mRNA*mRNA_p27_cytosol/mRNA_cytosol;

unbind_mRNA_p27 = unbind_mRNA*mRNA_p27_in_use/mRNA_in_use;

dk_mRNA_p27_cytosol = k_dk_mRNA*mRNA_p27_cytosol;

dk_mRNA_p27_unspl = k_dk_mRNA*mRNA_p27_unspl;

dk_mRNA_p27_nuc = k_dk_mRNA*mRNA_p27_nuc;

dk_mRNA_p27_inactive = k_dk_mRNA*mRNA_p27_inactive;

dk_mRNA_p27_in_use = k_dk_mRNA*mRNA_p27_in_use;

cut_mRNA_p27 = k_cut_mRNA*mRNA_p27_trash;

txl_p27 = unbind_rRNA*(mRNA_p27_cytosol + mRNA_p27_in_use)...

/(mRNA_cytosol + mRNA_in_use);

fold_p27 = ATP_frac_avail*k_fold_p*p27_unfold_conc;

nm_in_p27 = ATP_frac_avail*k_nm_in_p*p27_cytosol_conc;

bind_p27_to_cyclins = bind_cycD_Cdk4or6_to_p27 + bind_cycE_Cdk2_to_p27...

+ bind_cycA_Cdk2_to_p27;

unbind_p27_cyclins = unbind_cycD_Cdk4or6_p27 + unbind_cycE_Cdk2_p27...

+ unbind_cycA_Cdk2_p27;

% p27 is ubiq by SCF(Skp2) and APC(Cdc20) and primarily by KPC

% p27 ubiq by SCF(Skp2) is fast when p27_on_cyclins

% ASSUME APC(Cdc20) and SCF(Skp2) are nuclear, thus can only ubiq p27_nuc and p27_on_cyclins

% ASSUME KPC is cellular

u_p27_by_SCF_Skp2_fast = ATP_frac_avail*k_u_p27_by_SCF_Skp2_fast...

*Skp2_SCF_active_conc;

u_p27_by_SCF_Skp2_slow = ATP_frac_avail*k_u_p27_by_SCF_Skp2_slow...

*Skp2_SCF_active_conc;

u_p27_by_APC_Cdc20 = ATP_frac_avail*k_u_p27_by_APC_Cdc20*Cdc20_APC_active_conc;

u_p27_by_KPC = ATP_frac_avail*k_u_p27_by_KPC*KPC_active_conc;

u_p27_fast = u_p27_by_SCF_Skp2_fast + u_p27_by_APC_Cdc20 + u_p27_by_KPC;

u_p27_slow = u_p27_by_SCF_Skp2_slow + u_p27_by_APC_Cdc20 + u_p27_by_KPC;

u_p27_unfold = ATP_frac_avail*k_dk_p*p27_unfold;

u_p27_cytosol = u_p27_by_KPC*p27_cytosol_conc + ATP_frac_avail*k_dk_p*p27_cytosol;

u_p27_nuc = u_p27_slow*p27_nuc_conc + ATP_frac_avail*k_dk_p*p27_nuc;

u_p27_on_cyclins = u_p27_fast*p27_on_cyclins_conc...

+ ATP_frac_avail*k_dk_p*p27_on_cyclins;

% u_p27_nuc = (u_p27_by_KPC + u_p27_slow)*p27_nuc_conc...

% + ATP_frac_avail*k_dk_p*p27_nuc;

% u_p27_on_cyclins = (u_p27_by_KPC + u_p27_fast)*p27_on_cyclins_conc...

% + ATP_frac_avail*k_dk_p*p27_on_cyclins;

cut_p27 = ATP_frac_avail*k_cut_p*p27_trash_conc;

p27_all_conc = p27_nuc_conc + p27_on_cyclins_conc;

energy_u_p27_by_SCF_Skp2 = ATP_u*u_p27_by_SCF_Skp2_slow*p27_nuc_conc;

+ ATP_u*u_p27_by_SCF_Skp2_fast*p27_on_cyclins_conc;

energy_u_p27_by_APC_Cdc20 = ATP_u*u_p27_by_APC_Cdc20*p27_all_conc;

energy_u_p27_by_KPC = ATP_u*u_p27_by_KPC*p27_cytosol_conc;

% energy_u_p27_by_KPC = ATP_u*u_p27_by_KPC*(p27_cytosol_conc + p27_all_conc);

% 4 Cdk2 RATES

txs_mRNA_Cdk2 = ATP_frac_avail*k_mRNA_txs*polyII*Cdk2_loci_frac...

*(Cdk2_constituitive + E2F_on_DNA);

% txs_mRNA_Cdk2 = ATP_frac_avail*k_mRNA_txs*polyII*Cdk2_loci_frac;

spl_mRNA_Cdk2 = ATP_frac_avail*spl_frac*mRNA_Cdk2_unspl;

nm_out_mRNA_Cdk2 = ATP_frac_avail*k_nm_mRNA*mRNA_Cdk2_nuc_conc;

activate_mRNA_Cdk2 = activate_mRNA*mRNA_Cdk2_inactive/mRNA_inactive;

bind_mRNA_Cdk2 = bind_mRNA*mRNA_Cdk2_cytosol/mRNA_cytosol;

unbind_mRNA_Cdk2 = unbind_mRNA*mRNA_Cdk2_in_use/mRNA_in_use;

dk_mRNA_Cdk2_cytosol = k_dk_mRNA*mRNA_Cdk2_cytosol;

dk_mRNA_Cdk2_unspl = k_dk_mRNA*mRNA_Cdk2_unspl;

dk_mRNA_Cdk2_nuc = k_dk_mRNA*mRNA_Cdk2_nuc;

dk_mRNA_Cdk2_inactive = k_dk_mRNA*mRNA_Cdk2_inactive;

dk_mRNA_Cdk2_in_use = k_dk_mRNA*mRNA_Cdk2_in_use;

cut_mRNA_Cdk2 = k_cut_mRNA*mRNA_Cdk2_trash;

txl_Cdk2 = unbind_rRNA*(mRNA_Cdk2_cytosol + mRNA_Cdk2_in_use)...

/(mRNA_cytosol + mRNA_in_use);

fold_Cdk2 = ATP_frac_avail*k_fold_p*Cdk2_unfold_conc;

nm_in_Cdk2 = ATP_frac_avail*k_nm_in_p*Cdk2_cytosol_conc;

bind_Cdk2_to_cycE = bind_cycE_to_Cdk2;

unbind_Cdk2_cycE = unbind_cycE_Cdk2;

bind_Cdk2_to_cycA = bind_cycA_to_Cdk2;

unbind_Cdk2_cycA = unbind_cycA_Cdk2;

u_Cdk2_unfold = ATP_frac_avail*k_dk_p*Cdk2_unfold;

u_Cdk2_cytosol = ATP_frac_avail*k_dk_p*Cdk2_cytosol;

u_Cdk2_nuc = ATP_frac_avail*k_dk_p*Cdk2_nuc;

u_Cdk2_on_cycE = ATP_frac_avail*k_dk_p*Cdk2_on_cycE;

u_Cdk2_on_cycA = ATP_frac_avail*k_dk_p*Cdk2_on_cycA;

cut_Cdk2 = ATP_frac_avail*k_cut_p*Cdk2_trash_conc;

% 11 Skp2 RATES

txs_mRNA_Skp2 = ATP_frac_avail*k_mRNA_txs*polyII*Skp2_loci_frac;

spl_mRNA_Skp2 = ATP_frac_avail*spl_frac*mRNA_Skp2_unspl;

nm_out_mRNA_Skp2 = ATP_frac_avail*k_nm_mRNA*mRNA_Skp2_nuc_conc;

activate_mRNA_Skp2 = activate_mRNA*mRNA_Skp2_inactive/mRNA_inactive;

bind_mRNA_Skp2 = bind_mRNA*mRNA_Skp2_cytosol/mRNA_cytosol;

unbind_mRNA_Skp2 = unbind_mRNA*mRNA_Skp2_in_use/mRNA_in_use;

dk_mRNA_Skp2_cytosol = k_dk_mRNA*mRNA_Skp2_cytosol;

dk_mRNA_Skp2_unspl = k_dk_mRNA*mRNA_Skp2_unspl;

dk_mRNA_Skp2_nuc = k_dk_mRNA*mRNA_Skp2_nuc;

dk_mRNA_Skp2_inactive = k_dk_mRNA*mRNA_Skp2_inactive;

dk_mRNA_Skp2_in_use = k_dk_mRNA*mRNA_Skp2_in_use;

cut_mRNA_Skp2 = k_cut_mRNA*mRNA_Skp2_trash;

txl_Skp2 = unbind_rRNA*(mRNA_Skp2_cytosol + mRNA_Skp2_in_use)...

/(mRNA_cytosol + mRNA_in_use);

fold_Skp2 = ATP_frac_avail*k_fold_p*Skp2_unfold_conc;

nm_in_Skp2 = ATP_frac_avail*k_nm_in_p*Skp2_cytosol_conc;

% bind_Skp2_to_SCF = k_bind_Skp2_to_SCF*SCF_nuc_conc*Skp2_nuc_conc;

bind_Skp2_to_SCF = k_bind_Skp2_to_SCF*SCF_in_cell_conc*Skp2_nuc_conc;

unbind_Skp2_SCF = k_unbind_Skp2_SCF*Skp2_SCF_active_conc;

u_Skp2_by_APC_Cdh1 = ATP_frac_avail*k_u_Skp2_by_APC_Cdh1*Cdh1_APC_active_conc;

u_Skp2_unfold = ATP_frac_avail*k_dk_p*Skp2_unfold;

u_Skp2_cytosol = u_Skp2_by_APC_Cdh1*Skp2_cytosol_conc...

+ ATP_frac_avail*k_dk_p*Skp2_cytosol;

u_Skp2_nuc = u_Skp2_by_APC_Cdh1*Skp2_nuc_conc + ATP_frac_avail*k_dk_p*Skp2_nuc;

% Skp2 autoubiquinates when bound to SCF and there are no substrates

% substrates are p27, cycD, cycE (free), and RC

% (autoubiquination is dependent on number of molecules, not concentration)

% ASSUME Skp2 is nuclear and APC(Cdh1) is cellular

% IGNORE cycD

% NOTE -- Skp2 binds with cycA to protect it from p27 (Ji et al., 2006)

% substrates_Skp2_all = p27_cytosol + p27_nuc + p27_on_cyclins...

% + cycD_cytosol + cycD_nuc...

% + cycD_Cdk4or6_active + cycD_Cdk4or6_on_p27_inactive...

% + cycE_cytosol + cycE_nuc...

% + E2F_cytosol + E2F_nuc + E2F_Rb_inactive + E2F_on_DNA...

% + RC_cytosol + RC_nuc;

% I want Skp2 to hang around longer (to delay SCF(Btrc)) so I think any RC delays autoubiq

substrates_Skp2_all = p27_nuc + p27_on_cyclins...

+ cycE_nuc...

+ E2F_nuc...

+ RC_nuc + RC_on_DNA + RC_licensed + RC_traversed_by_DNA_poly;

% I want Skp2 to be able to ubiq Wee1 when Btrc is blocked 5-11-07

% substrates_Skp2_all = p27_nuc + p27_on_cyclins...

% + cycE_nuc...

% + E2F_nuc...

% + RC_nuc + RC_on_DNA + RC_licensed + RC_traversed_by_DNA_poly...

% + Wee1_phospho_inactive;

u_auto_Skp2 = ATP_frac_avail*k_u_auto_Skp2*Skp2_SCF_active...

/max(1, substrates_Skp2_all);

u_Skp2_SCF_active = u_Skp2_by_APC_Cdh1*Skp2_SCF_active_conc + u_auto_Skp2...

+ ATP_frac_avail*k_dk_p*Skp2_SCF_active;

cut_Skp2 = ATP_frac_avail*k_cut_p*Skp2_trash_conc;

energy_u_Skp2_by_APC_Cdh1 = ATP_u*u_Skp2_by_APC_Cdh1...

*(Skp2_cytosol_conc + Skp2_nuc_conc...

+ Skp2_SCF_active_conc);

energy_u_auto_Skp2 = ATP_u*u_auto_Skp2;

% 12 Btrc RATES

txs_mRNA_Btrc = ATP_frac_avail*k_mRNA_txs*polyII*Btrc_loci_frac*E2F_on_DNA;

spl_mRNA_Btrc = ATP_frac_avail*spl_frac*mRNA_Btrc_unspl;

nm_out_mRNA_Btrc = ATP_frac_avail*k_nm_mRNA*mRNA_Btrc_nuc_conc;

activate_mRNA_Btrc = activate_mRNA*mRNA_Btrc_inactive/mRNA_inactive;

bind_mRNA_Btrc = bind_mRNA*mRNA_Btrc_cytosol/mRNA_cytosol;

unbind_mRNA_Btrc = unbind_mRNA*mRNA_Btrc_in_use/mRNA_in_use;

dk_mRNA_Btrc_cytosol = k_dk_mRNA*mRNA_Btrc_cytosol;

dk_mRNA_Btrc_unspl = k_dk_mRNA*mRNA_Btrc_unspl;

dk_mRNA_Btrc_nuc = k_dk_mRNA*mRNA_Btrc_nuc;

dk_mRNA_Btrc_inactive = k_dk_mRNA*mRNA_Btrc_inactive;

dk_mRNA_Btrc_in_use = k_dk_mRNA*mRNA_Btrc_in_use;

cut_mRNA_Btrc = k_cut_mRNA*mRNA_Btrc_trash;

txl_Btrc = unbind_rRNA*(mRNA_Btrc_cytosol + mRNA_Btrc_in_use)...

/(mRNA_cytosol + mRNA_in_use);

fold_Btrc = ATP_frac_avail*k_fold_p*Btrc_unfold_conc;

bind_Btrc_to_SCF = k_bind_Btrc_to_SCF*SCF_in_cell_conc*Btrc_cytosol_conc;

unbind_Btrc_SCF = k_unbind_Btrc_SCF*Btrc_SCF_active_conc;

% ASSUME Btrc ubiq by cellular APC(Cdh1)

u_Btrc_by_APC_Cdh1 = ATP_frac_avail*k_u_Btrc_by_APC_Cdh1*Cdh1_APC_active_conc;

u_Btrc_unfold = ATP_frac_avail*k_dk_p*Btrc_unfold;

u_Btrc_cytosol = u_Btrc_by_APC_Cdh1*Btrc_cytosol_conc...

+ ATP_frac_avail*k_dk_p*Btrc_cytosol;

% Btrc autoubiqs (because it is cytoplasmic)

% Btrc autoubiquinates when bound to SCF and there are no substrates

% substrates are Emi1 and Wee1_phospho_inactive (i.e., marked by cycB/Cdk1 and Plk1)

% (autoubiquination is dependent on number of molecules, not concentration)

% substrates_Btrc_all = Emi1_in_cell + Emi1_on_Cdh1 + Emi1_on_Cdc20...

% + Emi1_phospho_inactive...

% + Wee1_cytosol_active + Wee1_phospho_inactive...

% + Cdc25A_cytosol + Cdc25A_nuc_inactive + Cdc25A_phospho_active;

% ASSUME Cdc25A is a substrate only as a checkpoint

substrates_Btrc_all = Emi1_in_cell + Emi1_on_Cdh1 + Emi1_on_Cdc20...

+ Emi1_phospho_inactive...

+ Wee1_phospho_inactive;

u_auto_Btrc = ATP_frac_avail*k_u_auto_Btrc*Btrc_SCF_active...

/max(1, substrates_Btrc_all);

u_Btrc_SCF_active = u_Btrc_by_APC_Cdh1*Btrc_SCF_active_conc...

+ ATP_frac_avail*k_dk_p*Btrc_SCF_active;

cut_Btrc = ATP_frac_avail*k_cut_p*Btrc_trash_conc;

energy_u_auto_Btrc = ATP_u*u_Btrc_by_APC_Cdh1...

*(Btrc_cytosol_conc + Btrc_SCF_active_conc)...

+ ATP_u*u_auto_Btrc;

% 13 Fbw7 RATES

txs_mRNA_Fbw7 = ATP_frac_avail*k_mRNA_txs*polyII*Fbw7_loci_frac*E2F_on_DNA;

spl_mRNA_Fbw7 = ATP_frac_avail*spl_frac*mRNA_Fbw7_unspl;

nm_out_mRNA_Fbw7 = ATP_frac_avail*k_nm_mRNA*mRNA_Fbw7_nuc_conc;

activate_mRNA_Fbw7 = activate_mRNA*mRNA_Fbw7_inactive/mRNA_inactive;

bind_mRNA_Fbw7 = bind_mRNA*mRNA_Fbw7_cytosol/mRNA_cytosol;

unbind_mRNA_Fbw7 = unbind_mRNA*mRNA_Fbw7_in_use/mRNA_in_use;

dk_mRNA_Fbw7_cytosol = k_dk_mRNA*mRNA_Fbw7_cytosol;

dk_mRNA_Fbw7_unspl = k_dk_mRNA*mRNA_Fbw7_unspl;

dk_mRNA_Fbw7_nuc = k_dk_mRNA*mRNA_Fbw7_nuc;

dk_mRNA_Fbw7_inactive = k_dk_mRNA*mRNA_Fbw7_inactive;

dk_mRNA_Fbw7_in_use = k_dk_mRNA*mRNA_Fbw7_in_use;

cut_mRNA_Fbw7 = k_cut_mRNA*mRNA_Fbw7_trash;

txl_Fbw7 = unbind_rRNA*(mRNA_Fbw7_cytosol + mRNA_Fbw7_in_use)...

/(mRNA_cytosol + mRNA_in_use);

fold_Fbw7 = ATP_frac_avail*k_fold_p*Fbw7_unfold_conc;

nm_in_Fbw7 = ATP_frac_avail*k_nm_in_p*Fbw7_cytosol_conc;

bind_Fbw7_to_SCF = k_bind_Fbw7_to_SCF*SCF_in_cell_conc*Fbw7_nuc_conc;

unbind_Fbw7_SCF = k_unbind_Fbw7_SCF*Fbw7_SCF_active_conc;

u_Fbw7_unfold = ATP_frac_avail*k_dk_p*Fbw7_unfold;

u_Fbw7_cytosol = ATP_frac_avail*k_dk_p*Fbw7_cytosol;

u_Fbw7_nuc = ATP_frac_avail*k_dk_p*Fbw7_nuc;

% Fbw7 autoubiquinates when bound to SCF and there are no substrates

% substrates are TF_grow (not on DNA), cycE (bound to Cdk2), and RC (all in nucleus)

% (autoubiquination is dependent on number of molecules, not concentration)

% Assume Fbw7 nuclear

% substrates_Fbw7_all = TF_grow_nuc_conc...

% + cycE_Cdk2_active_conc + cycE_Cdk2_inactive_conc...

% + cycE_Cdk2_on_p27_inactive_conc...

% + RC_nuc + RC_on_DNA + RC_licensed + RC_traversed_by_DNA_poly;

substrates_Fbw7_all = cycE_Cdk2_active_conc + cycE_Cdk2_inactive_conc...

+ cycE_Cdk2_on_p27_inactive_conc;

u_auto_Fbw7 = ATP_frac_avail*k_u_auto_Fbw7*Fbw7_SCF_active_conc...

/max(1, substrates_Fbw7_all);

% u_auto_Fbw7 = ATP_frac_avail*k_u_auto_Fbw7*Fbw7_SCF_active_conc...

% /log10(max(10, substrates_Fbw7_all));

u_Fbw7_SCF_active = u_auto_Fbw7 + ATP_frac_avail*k_dk_p*Fbw7_SCF_active;

cut_Fbw7 = ATP_frac_avail*k_cut_p*Fbw7_trash_conc;

energy_u_auto_Fbw7 = ATP_u*u_auto_Fbw7;

% 10 SCF RATES

txs_mRNA_SCF = ATP_frac_avail*k_mRNA_txs*polyII*SCF_loci_frac;

spl_mRNA_SCF = ATP_frac_avail*spl_frac*mRNA_SCF_unspl;

nm_out_mRNA_SCF = ATP_frac_avail*k_nm_mRNA*mRNA_SCF_nuc_conc;

activate_mRNA_SCF = activate_mRNA*mRNA_SCF_inactive/mRNA_inactive;

bind_mRNA_SCF = bind_mRNA*mRNA_SCF_cytosol/mRNA_cytosol;

unbind_mRNA_SCF = unbind_mRNA*mRNA_SCF_in_use/mRNA_in_use;

dk_mRNA_SCF_cytosol = k_dk_mRNA*mRNA_SCF_cytosol;

dk_mRNA_SCF_unspl = k_dk_mRNA*mRNA_SCF_unspl;

dk_mRNA_SCF_nuc = k_dk_mRNA*mRNA_SCF_nuc;

dk_mRNA_SCF_inactive = k_dk_mRNA*mRNA_SCF_inactive;

dk_mRNA_SCF_in_use = k_dk_mRNA*mRNA_SCF_in_use;

cut_mRNA_SCF = k_cut_mRNA*mRNA_SCF_trash;

txl_SCF = unbind_rRNA*(mRNA_SCF_cytosol + mRNA_SCF_in_use)...

/(mRNA_cytosol + mRNA_in_use);

fold_SCF = ATP_frac_avail*k_fold_p*SCF_unfold_conc;

bind_SCF_to_Btrc = bind_Btrc_to_SCF;

unbind_SCF_Btrc = unbind_Btrc_SCF;

bind_SCF_to_Fbw7 = bind_Fbw7_to_SCF;

unbind_SCF_Fbw7 = unbind_Fbw7_SCF;

bind_SCF_to_Skp2 = bind_Skp2_to_SCF;

unbind_SCF_Skp2 = unbind_Skp2_SCF;

% ASSUME SCF easily passes between nucleus and cytosol so that it can bind

% nuclear Fbw7 and Skp2 and cytoplasmic Btrc,

% and so it can be ubiq by APC(Cdh1) (ASSUME even when it is on Btrc)

u_SCF_in_cell_by_APC_Cdh1 = ATP_frac_avail*k_u_SCF_in_cell_by_APC_Cdh1...

*Cdh1_APC_active_conc;

u_SCF_active_by_APC_Cdh1 = ATP_frac_avail*k_u_SCF_active_by_APC_Cdh1...

*Cdh1_APC_active_conc;

u_SCF_unfold = ATP_frac_avail*k_dk_p*SCF_unfold;

u_SCF_in_cell = u_SCF_in_cell_by_APC_Cdh1*SCF_in_cell_conc...

+ ATP_frac_avail*k_dk_p*SCF_in_cell;

u_SCF_on_Btrc = ATP_frac_avail*k_dk_p*SCF_on_Btrc;

u_SCF_on_Fbw7 = u_SCF_active_by_APC_Cdh1*SCF_on_Fbw7_conc...

+ ATP_frac_avail*k_dk_p*SCF_on_Fbw7;

u_SCF_on_Skp2 = u_SCF_active_by_APC_Cdh1*SCF_on_Skp2_conc...

+ ATP_frac_avail*k_dk_p*SCF_on_Skp2;

cut_SCF = ATP_frac_avail*k_cut_p*SCF_trash_conc;

energy_u_SCF_by_APC_Cdh1 = ATP_u*u_SCF_in_cell_by_APC_Cdh1*SCF_in_cell_conc...

+ ATP_u*u_SCF_active_by_APC_Cdh1...

*(SCF_on_Skp2_conc + SCF_on_Fbw7_conc + SCF_on_Btrc_conc);

% 14 TF_grow RATES

txs_mRNA_TF_grow = ATP_frac_avail*k_mRNA_txs*polyII*TF_grow_loci_frac...

*mitogen_stimulation*(TF_grow_txs_by_mitogen...

+ k_TF_grow_txs_by_Skp2*Skp2_nuc_conc...

+ B_Myb_on_DNA);

spl_mRNA_TF_grow = ATP_frac_avail*spl_frac*mRNA_TF_grow_unspl;

nm_out_mRNA_TF_grow = ATP_frac_avail*k_nm_mRNA*mRNA_TF_grow_nuc_conc;

activate_mRNA_TF_grow = activate_mRNA*mRNA_TF_grow_inactive/mRNA_inactive;

bind_mRNA_TF_grow = bind_mRNA*mRNA_TF_grow_cytosol/mRNA_cytosol;

unbind_mRNA_TF_grow = unbind_mRNA*mRNA_TF_grow_in_use/mRNA_in_use;

dk_mRNA_TF_grow_cytosol = k_dk_mRNA*mRNA_TF_grow_cytosol;

dk_mRNA_TF_grow_unspl = k_dk_mRNA*mRNA_TF_grow_unspl;

dk_mRNA_TF_grow_nuc = k_dk_mRNA*mRNA_TF_grow_nuc;

dk_mRNA_TF_grow_inactive = k_dk_mRNA*mRNA_TF_grow_inactive;

dk_mRNA_TF_grow_in_use = k_dk_mRNA*mRNA_TF_grow_in_use;

cut_mRNA_TF_grow = k_cut_mRNA*mRNA_TF_grow_trash;

txl_TF_grow = unbind_rRNA*(mRNA_TF_grow_cytosol + mRNA_TF_grow_in_use)...

/(mRNA_cytosol + mRNA_in_use);

fold_TF_grow = ATP_frac_avail*k_fold_p*TF_grow_unfold_conc;

nm_in_TF_grow = ATP_frac_avail*k_nm_in_p*TF_grow_cytosol_conc;

% bind_TF_grow_to_DNA = k_bind_TF_grow_to_DNA*TF_grow_nuc_conc;

bind_TF_grow_to_DNA = min(k_bind_TF_grow_to_DNA*TF_grow_nuc_conc, ...

k_bind_TF_grow_to_DNA*max(0, sat_TF_grow_on_DNA - TF_grow_on_DNA));

unbind_TF_grow_DNA = k_unbind_TF_grow_DNA*TF_grow_on_DNA_conc;

% ASSUME (for now) that TF_grow is not ubiquinated while on DNA

u_TF_grow_by_SCF_Fbw7 = ATP_frac_avail*k_u_TF_grow_by_SCF_Fbw7*Fbw7_SCF_active_conc;

u_TF_grow_by_SCF_Skp2 = ATP_frac_avail*k_u_TF_grow_by_SCF_Skp2*Skp2_SCF_active_conc;

u_TF_grow = u_TF_grow_by_SCF_Fbw7 + u_TF_grow_by_SCF_Skp2;

u_TF_grow_unfold = ATP_frac_avail*k_dk_p*TF_grow_unfold;

u_TF_grow_cytosol = ATP_frac_avail*k_dk_p*TF_grow_cytosol;

u_TF_grow_nuc = u_TF_grow*TF_grow_nuc_conc...

+ ATP_frac_avail*k_dk_p*TF_grow_nuc;

u_TF_grow_on_DNA = ATP_frac_avail*k_dk_p*TF_grow_on_DNA;

cut_TF_grow = ATP_frac_avail*k_cut_p*TF_grow_trash_conc;

energy_u_TF_grow_by_SCF_Skp2 = ATP_u*u_TF_grow_by_SCF_Skp2*TF_grow_nuc_conc;

energy_u_TF_grow_by_SCF_Fbw7 = ATP_u*u_TF_grow_by_SCF_Fbw7*TF_grow_nuc_conc;

% 17 DNA_poly RATES

% txs_mRNA_DNA_poly = ATP_frac_avail*k_mRNA_txs*polyII*DNA_poly_loci_frac...

% *(E2F_on_DNA + B_Myb_on_DNA + NF_Y_on_DNA);

% txs_mRNA_DNA_poly = ATP_frac_avail*k_mRNA_txs*polyII*DNA_poly_loci_frac...

% *E2F_on_DNA;

txs_mRNA_DNA_poly = min(ATP_frac_avail*k_mRNA_txs*polyII*DNA_poly_loci_frac...

*(E2F_on_DNA + B_Myb_on_DNA + NF_Y_on_DNA), ...

max(0, sat_DNA_poly - (DNA_poly_nuc + DNA_poly_on_DNA)));

spl_mRNA_DNA_poly = ATP_frac_avail*spl_frac*mRNA_DNA_poly_unspl;

nm_out_mRNA_DNA_poly = ATP_frac_avail*k_nm_mRNA*mRNA_DNA_poly_nuc_conc;

activate_mRNA_DNA_poly = activate_mRNA*mRNA_DNA_poly_inactive/mRNA_inactive;

bind_mRNA_DNA_poly = bind_mRNA*mRNA_DNA_poly_cytosol/mRNA_cytosol;

unbind_mRNA_DNA_poly = unbind_mRNA*mRNA_DNA_poly_in_use/mRNA_in_use;

dk_mRNA_DNA_poly_cytosol = k_dk_mRNA*mRNA_DNA_poly_cytosol;

dk_mRNA_DNA_poly_unspl = k_dk_mRNA*mRNA_DNA_poly_unspl;

dk_mRNA_DNA_poly_nuc = k_dk_mRNA*mRNA_DNA_poly_nuc;

dk_mRNA_DNA_poly_inactive = k_dk_mRNA*mRNA_DNA_poly_inactive;

dk_mRNA_DNA_poly_in_use = k_dk_mRNA*mRNA_DNA_poly_in_use;

cut_mRNA_DNA_poly = k_cut_mRNA*mRNA_DNA_poly_trash;

txl_DNA_poly = unbind_rRNA*(mRNA_DNA_poly_cytosol + mRNA_DNA_poly_in_use)...

/(mRNA_cytosol + mRNA_in_use);

fold_DNA_poly = ATP_frac_avail*k_fold_p*DNA_poly_unfold_conc;

nm_in_DNA_poly = ATP_frac_avail*k_nm_in_p*DNA_poly_cytosol_conc;

% tie bind_DNA_poly to number of available RC

bind_DNA_poly_to_DNA = min(1, k_bind_DNA_poly_to_DNA*DNA_poly_nuc_conc)...

*x_DNA_poly_per_RC*RC_licensed;

% unbind_DNA_poly when a DNA frag is completed

% unbind_DNA_poly_from_DNA = ATP_frac_avail*NT_frac_avail*ER_DNA...

% *DNA_poly_on_DNA/x_RC_frag;

unbind_DNA_poly_from_DNA = ATP_frac_avail*NT_frac_avail*ER_DNA...

*DNA_poly_on_DNA*x_DNA_poly_per_RC/x_NT_RC_frag;

u_DNA_poly_unfold = ATP_frac_avail*k_dk_p*DNA_poly_unfold;

u_DNA_poly_cytosol = ATP_frac_avail*k_dk_p*DNA_poly_cytosol;

u_DNA_poly_nuc = ATP_frac_avail*k_dk_p*DNA_poly_nuc;

u_DNA_poly_on_DNA = ATP_frac_avail*k_dk_p*DNA_poly_on_DNA;

cut_DNA_poly = ATP_frac_avail*k_cut_p*DNA_poly_trash_conc;

% 16 RC RATES

txs_mRNA_RC = ATP_frac_avail*k_mRNA_txs*polyII*RC_loci_frac*E2F_on_DNA;

spl_mRNA_RC = ATP_frac_avail*spl_frac*mRNA_RC_unspl;

nm_out_mRNA_RC = ATP_frac_avail*k_nm_mRNA*mRNA_RC_nuc_conc;

activate_mRNA_RC = activate_mRNA*mRNA_RC_inactive/mRNA_inactive;

bind_mRNA_RC = bind_mRNA*mRNA_RC_cytosol/mRNA_cytosol;

unbind_mRNA_RC = unbind_mRNA*mRNA_RC_in_use/mRNA_in_use;

dk_mRNA_RC_cytosol = k_dk_mRNA*mRNA_RC_cytosol;

dk_mRNA_RC_unspl = k_dk_mRNA*mRNA_RC_unspl;

dk_mRNA_RC_nuc = k_dk_mRNA*mRNA_RC_nuc;

dk_mRNA_RC_inactive = k_dk_mRNA*mRNA_RC_inactive;

dk_mRNA_RC_in_use = k_dk_mRNA*mRNA_RC_in_use;

cut_mRNA_RC = k_cut_mRNA*mRNA_RC_trash;

txl_RC = unbind_rRNA*(mRNA_RC_cytosol + mRNA_RC_in_use)...

/(mRNA_cytosol + mRNA_in_use);

fold_RC = ATP_frac_avail*k_fold_p*RC_unfold_conc;

nm_in_RC = ATP_frac_avail*k_nm_in_p*RC_cytosol_conc;

% RC attached to DNA must not exceed x_RC = 3E9 bp / 2E5 bp = 1.5E4 RC

bind_RC_to_DNA = k_bind_RC_to_DNA*RC_nuc_conc*(x_RC - RC_count);

unbind_RC_DNA = k_unbind_RC_DNA*RC_on_DNA_conc;

% phospho_RC_by_cycD_Cdk4or6 = k_phospho_RC_by_cycD_Cdk4or6...

% *cycD_Cdk4or6_active_conc...

% *RC_on_DNA_conc;

phospho_RC_by_cycD_Cdk4or6 = (k_phospho_RC_by_cycD_Cdk4or6...

*cycD_Cdk4or6_active_conc...

+ k_phospho_RC_by_cycD_Cdk4or6_on_p27...

*cycD_Cdk4or6_on_p27_inactive_conc)...

*RC_on_DNA_conc;

phospho_RC_by_cycE_Cdk2 = k_phospho_RC_by_cycE_Cdk2*cycE_Cdk2_active_conc...

*RC_on_DNA_conc;

phospho_RC_by_cycA_Cdk2 = k_phospho_RC_by_cycA_Cdk2*cycA_Cdk2_active_conc...

*RC_on_DNA_conc;

attach_RC_DNA_to_DNA_poly = bind_DNA_poly_to_DNA/x_DNA_poly_per_RC;

% no need to worry about concentration for DNA_poly

delicense_RC = unbind_DNA_poly_from_DNA/x_DNA_poly_per_RC;

% ASSUME RC ubiq only when not on DNA

% AND since SCF(Fbw7) and SCF(Skp2) are nuclear, they ubiq only RC_nuc

% AND since APC(Cdh1) is cellular, it ubiqs RC in nuc and cytosol

% AND since SCF(Fbw7) apparently activates too soon, I will probably omit ubiq by it

u_RC_by_SCF_Fbw7 = k_u_RC_by_SCF_Fbw7*Fbw7_SCF_active_conc;

u_RC_by_SCF_Skp2 = k_u_RC_by_SCF_Skp2*Skp2_SCF_active_conc;

u_RC_by_APC_Cdh1 = k_u_RC_by_APC_Cdh1*Cdh1_APC_active_conc;

u_RC = u_RC_by_SCF_Fbw7 + u_RC_by_SCF_Skp2 + u_RC_by_APC_Cdh1;

u_RC_unfold = ATP_frac_avail*k_dk_p*RC_unfold;

u_RC_cytosol = u_RC_by_APC_Cdh1*RC_cytosol_conc...

+ ATP_frac_avail*k_dk_p*RC_cytosol;

u_RC_nuc = u_RC*RC_nuc_conc + ATP_frac_avail*k_dk_p*RC_nuc;

u_RC_on_DNA = ATP_frac_avail*k_dk_p*RC_on_DNA;

u_RC_licensed = ATP_frac_avail*k_dk_p*RC_licensed;

u_RC_traversed_by_DNA_poly = ATP_frac_avail*k_dk_p*RC_traversed_by_DNA_poly;

cut_RC = ATP_frac_avail*k_cut_p*RC_trash_conc;

energy_u_RC_by_SCF_Fbw7 = ATP_u*u_RC_by_SCF_Fbw7*RC_nuc_conc;

energy_u_RC_by_SCF_Skp2 = ATP_u*u_RC_by_SCF_Skp2*RC_nuc_conc;

energy_u_RC_by_APC_Cdh1 = ATP_u*u_RC_by_APC_Cdh1*(RC_cytosol_conc + RC_nuc_conc);

% 18 Wee1 RATES

txs_mRNA_Wee1 = ATP_frac_avail*k_mRNA_txs*polyII*Wee1_loci_frac;

spl_mRNA_Wee1 = ATP_frac_avail*spl_frac*mRNA_Wee1_unspl;

nm_out_mRNA_Wee1 = ATP_frac_avail*k_nm_mRNA*mRNA_Wee1_nuc_conc;

activate_mRNA_Wee1 = activate_mRNA*mRNA_Wee1_inactive/mRNA_inactive;

bind_mRNA_Wee1 = bind_mRNA*mRNA_Wee1_cytosol/mRNA_cytosol;

unbind_mRNA_Wee1 = unbind_mRNA*mRNA_Wee1_in_use/mRNA_in_use;

dk_mRNA_Wee1_cytosol = k_dk_mRNA*mRNA_Wee1_cytosol;

dk_mRNA_Wee1_unspl = k_dk_mRNA*mRNA_Wee1_unspl;

dk_mRNA_Wee1_nuc = k_dk_mRNA*mRNA_Wee1_nuc;

dk_mRNA_Wee1_inactive = k_dk_mRNA*mRNA_Wee1_inactive;

dk_mRNA_Wee1_in_use = k_dk_mRNA*mRNA_Wee1_in_use;

cut_mRNA_Wee1 = k_cut_mRNA*mRNA_Wee1_trash;

txl_Wee1 = unbind_rRNA*(mRNA_Wee1_cytosol + mRNA_Wee1_in_use)...

/(mRNA_cytosol + mRNA_in_use);

fold_Wee1 = ATP_frac_avail*k_fold_p*Wee1_unfold_conc;

% marked for ubiquination by cycB/Cdk1 and Plk1

% NOTE that according to Watanabe et al. 2005, ubiq is by Cdk2, Ck2, and most importantly, Plk1

% THEREFORE, I will only consider Plk1 for now

% i.e., k_phospho_Wee1_by_cycA_Cdk1 = 0 & k_phospho_Wee1_by_cycB_Cdk1 = 0

phospho_Wee1_by_Cdk1_and_Plk1 = (k_phospho_Wee1_by_cycA_Cdk1...

*cycA_Cdk1_cytosol_active_conc...

+ k_phospho_Wee1_by_cycB_Cdk1...

*cycB_Cdk1_cytosol_active_conc...

+ k_phospho_Wee1_by_Plk1...

*Plk1_cytosol_phospho_active_conc)...

*Wee1_cytosol_active_conc;

unphospho_Wee1 = k_unphospho_Wee1*Wee1_phospho_inactive_conc;

% unmarked by Cdc14

dephospho_Wee1_by_Cdc14 = k_dephospho_Wee1_by_Cdc14*Cdc14_phospho_active_conc...

*Wee1_phospho_inactive_conc;

u_Wee1_by_SCF_Btrc = ATP_frac_avail*k_u_Wee1_by_SCF_Btrc*Btrc_SCF_active_conc;

u_Wee1_by_SCF_Skp2 = ATP_frac_avail*k_u_Wee1_by_SCF_Skp2_test...

*Skp2_SCF_active_conc;

u_Wee1_unfold = ATP_frac_avail*k_dk_p*Wee1_unfold;

u_Wee1_cytosol_active = ATP_frac_avail*k_dk_p*Wee1_cytosol_active;

u_Wee1_phospho_inactive = u_Wee1_by_SCF_Btrc*Wee1_phospho_inactive_conc...

+ u_Wee1_by_SCF_Skp2*Wee1_phospho_inactive_conc...

+ ATP_frac_avail*k_dk_p*Wee1_phospho_inactive;

cut_Wee1 = ATP_frac_avail*k_cut_p*Wee1_trash_conc;

energy_u_Wee1_by_SCF_Btrc = ATP_u*u_Wee1_by_SCF_Btrc*Wee1_phospho_inactive_conc;

energy_u_Wee1_by_SCF_Skp2 = ATP_u*u_Wee1_by_SCF_Skp2*Wee1_phospho_inactive_conc;

% 19 cycB RATES

txs_mRNA_cycB = ATP_frac_avail*k_mRNA_txs*polyII*cycB_loci_frac...

*(E2F_on_DNA + B_Myb_on_DNA + NF_Y_on_DNA);

% txs_mRNA_cycB = ATP_frac_avail*k_mRNA_txs*polyII*cycB_loci_frac...

% *E2F_on_DNA*log10_B_Myb_on_DNA*log10_NF_Y_on_DNA;

spl_mRNA_cycB = ATP_frac_avail*spl_frac*mRNA_cycB_unspl;

nm_out_mRNA_cycB = ATP_frac_avail*k_nm_mRNA*mRNA_cycB_nuc_conc;

activate_mRNA_cycB = activate_mRNA*mRNA_cycB_inactive/mRNA_inactive;

bind_mRNA_cycB = bind_mRNA*mRNA_cycB_cytosol/mRNA_cytosol;

unbind_mRNA_cycB = unbind_mRNA*mRNA_cycB_in_use/mRNA_in_use;

dk_mRNA_cycB_cytosol = k_dk_mRNA*mRNA_cycB_cytosol;

dk_mRNA_cycB_unspl = k_dk_mRNA*mRNA_cycB_unspl;

dk_mRNA_cycB_nuc = k_dk_mRNA*mRNA_cycB_nuc;

dk_mRNA_cycB_inactive = k_dk_mRNA*mRNA_cycB_inactive;

dk_mRNA_cycB_in_use = k_dk_mRNA*mRNA_cycB_in_use;

cut_mRNA_cycB = k_cut_mRNA*mRNA_cycB_trash;

txl_cycB = unbind_rRNA*(mRNA_cycB_cytosol + mRNA_cycB_in_use)...

/(mRNA_cytosol + mRNA_in_use);

fold_cycB = ATP_frac_avail*k_fold_p*cycB_unfold_conc;

bind_cycB_to_Cdk1 = k_bind_cycB_to_Cdk1*cycB_cytosol_conc*Cdk1_in_cell_conc;

unbind_cycB_Cdk1 = k_unbind_cycB_Cdk1*cycB_Cdk1_cytosol_active_conc;

phospho_cycB_Cdk1_by_Wee1 = k_phospho_cycB_Cdk1_by_Wee1...

*Wee1_cytosol_active_conc*cycB_Cdk1_cytosol_active_conc...

+ k_phospho_cycB_Cdk1_by_phospho_Wee1...

*Wee1_phospho_inactive_conc*cycB_Cdk1_cytosol_active_conc;

unphospho_cycB_Cdk1_from_Wee1 = k_unphospho_cycB_Cdk1_from_Wee1...

*cycB_Cdk1_phospho_inactive_conc;

% ASSUME only Cdc25B and Cdc25C are active in cytosol

Cdc25_cytosol_all = k_dephospho_cycB_Cdk1_by_Cdc25A...

*Cdc25A_phospho_active_conc...

+ k_dephospho_cycB_Cdk1_by_Cdc25B...

*Cdc25B_cytosol_phospho_active_conc...

+ k_dephospho_cycB_Cdk1_by_Cdc25C...

*Cdc25C_cytosol_phospho_active_conc;

dephospho_cycB_Cdk1_by_Cdc25 = Cdc25_cytosol_all*cycB_Cdk1_phospho_inactive_conc...

+ unphospho_cycB_Cdk1_from_Wee1;

nm_in_cycB_Cdk1 = ATP_frac_avail*k_nm_in_cycB_Cdk1_by_Plk1...

*Plk1_cytosol_phospho_active_conc...

*cycB_Cdk1_cytosol_active_conc;

nm_out_cycB_Cdk1 = k_nm_out_cycB_Cdk1*cycB_Cdk1_nuc_active_conc;

u_cycB_by_APC_Cdc20 = k_u_cycB_by_APC_Cdc20*Cdc20_APC_active_conc;

u_cycB_by_APC_Cdh1 = k_u_cycB_by_APC_Cdh1*Cdh1_APC_active_conc;

u_cycB = u_cycB_by_APC_Cdc20 + u_cycB_by_APC_Cdh1;

u_cycB_unfold = ATP_frac_avail*k_dk_p*cycB_unfold;

u_cycB_cytosol = u_cycB_by_APC_Cdh1*cycB_cytosol_conc...

+ ATP_frac_avail*k_dk_p*cycB_cytosol;

u_cycB_Cdk1_phospho_inactive = u_cycB_by_APC_Cdh1*cycB_Cdk1_phospho_inactive_conc...

+ ATP_frac_avail*k_dk_p*cycB_Cdk1_phospho_inactive;

u_cycB_Cdk1_cytosol_active = u_cycB_by_APC_Cdh1*cycB_Cdk1_cytosol_active_conc...

+ ATP_frac_avail*k_dk_p*cycB_Cdk1_cytosol_active;

u_cycB_Cdk1_nuc_active = u_cycB*cycB_Cdk1_nuc_active_conc...

+ ATP_frac_avail*k_dk_p*cycB_Cdk1_nuc_active;

cut_cycB = ATP_frac_avail*k_cut_p*cycB_trash_conc;

energy_u_cycB_by_APC_Cdc20 = ATP_u*u_cycB_by_APC_Cdc20*cycB_Cdk1_nuc_active_conc;

energy_u_cycB_by_APC_Cdh1 = ATP_u*u_cycB_by_APC_Cdh1...

*(cycB_cytosol_conc...

+ cycB_Cdk1_phospho_inactive_conc...

+ cycB_Cdk1_cytosol_active_conc...

+ cycB_Cdk1_nuc_active_conc);

% 20 Cdk1 RATES

txs_mRNA_Cdk1 = ATP_frac_avail*k_mRNA_txs*polyII*Cdk1_loci_frac...

*(Cdk1_constitutive + E2F_on_DNA...

+ B_Myb_on_DNA + NF_Y_on_DNA);

spl_mRNA_Cdk1 = ATP_frac_avail*spl_frac*mRNA_Cdk1_unspl;

nm_out_mRNA_Cdk1 = ATP_frac_avail*k_nm_mRNA*mRNA_Cdk1_nuc_conc;

activate_mRNA_Cdk1 = activate_mRNA*mRNA_Cdk1_inactive/mRNA_inactive;

bind_mRNA_Cdk1 = bind_mRNA*mRNA_Cdk1_cytosol/mRNA_cytosol;

unbind_mRNA_Cdk1 = unbind_mRNA*mRNA_Cdk1_in_use/mRNA_in_use;

dk_mRNA_Cdk1_cytosol = k_dk_mRNA*mRNA_Cdk1_cytosol;

dk_mRNA_Cdk1_unspl = k_dk_mRNA*mRNA_Cdk1_unspl;

dk_mRNA_Cdk1_nuc = k_dk_mRNA*mRNA_Cdk1_nuc;

dk_mRNA_Cdk1_inactive = k_dk_mRNA*mRNA_Cdk1_inactive;

dk_mRNA_Cdk1_in_use = k_dk_mRNA*mRNA_Cdk1_in_use;

cut_mRNA_Cdk1 = k_cut_mRNA*mRNA_Cdk1_trash;

txl_Cdk1 = unbind_rRNA*(mRNA_Cdk1_cytosol + mRNA_Cdk1_in_use)...

/(mRNA_cytosol + mRNA_in_use);

fold_Cdk1 = ATP_frac_avail*k_fold_p*Cdk1_unfold_conc;

activate_Cdk1 = ATP_frac_avail*k_activate_Cdk1_by_B_Myb_and_NF_Y...

*Cdk1_inactive_conc*B_Myb_phospho_active_conc...

*NF_Y_phospho_active_conc;

deactivate_Cdk1 = k_deactivate_Cdk1*Cdk1_in_cell_conc;

dephospho_Cdk1_by_Cdc14 = k_dephospho_Cdk1_by_Cdc14*Cdc14_phospho_active_conc...

*Cdk1_in_cell_conc;

bind_Cdk1_to_cycB = bind_cycB_to_Cdk1;

unbind_Cdk1_cycB = unbind_cycB_Cdk1;

bind_Cdk1_to_cycA = bind_cycA_to_Cdk1;

unbind_Cdk1_cycA = unbind_cycA_Cdk1;

u_Cdk1_unfold = ATP_frac_avail*k_dk_p*Cdk1_unfold;

u_Cdk1_inactive = ATP_frac_avail*k_dk_p*Cdk1_inactive;

u_Cdk1_in_cell = ATP_frac_avail*k_dk_p*Cdk1_in_cell;

u_Cdk1_on_cycB = ATP_frac_avail*k_dk_p*Cdk1_on_cycB;

u_Cdk1_on_cycA = ATP_frac_avail*k_dk_p*Cdk1_on_cycA;

cut_Cdk1 = ATP_frac_avail*k_cut_p*Cdk1_trash_conc;

% 21 Cdc25C RATES

txs_mRNA_Cdc25C = ATP_frac_avail*k_mRNA_txs*polyII*Cdc25C_loci_frac;

spl_mRNA_Cdc25C = ATP_frac_avail*spl_frac*mRNA_Cdc25C_unspl;

nm_out_mRNA_Cdc25C = ATP_frac_avail*k_nm_mRNA*mRNA_Cdc25C_nuc_conc;

activate_mRNA_Cdc25C = activate_mRNA*mRNA_Cdc25C_inactive/mRNA_inactive;

bind_mRNA_Cdc25C = bind_mRNA*mRNA_Cdc25C_cytosol/mRNA_cytosol;

unbind_mRNA_Cdc25C = unbind_mRNA*mRNA_Cdc25C_in_use/mRNA_in_use;

dk_mRNA_Cdc25C_cytosol = k_dk_mRNA*mRNA_Cdc25C_cytosol;

dk_mRNA_Cdc25C_unspl = k_dk_mRNA*mRNA_Cdc25C_unspl;

dk_mRNA_Cdc25C_nuc = k_dk_mRNA*mRNA_Cdc25C_nuc;

dk_mRNA_Cdc25C_inactive = k_dk_mRNA*mRNA_Cdc25C_inactive;

dk_mRNA_Cdc25C_in_use = k_dk_mRNA*mRNA_Cdc25C_in_use;

cut_mRNA_Cdc25C = k_cut_mRNA*mRNA_Cdc25C_trash;

txl_Cdc25C = unbind_rRNA*(mRNA_Cdc25C_cytosol + mRNA_Cdc25C_in_use)...

/(mRNA_cytosol + mRNA_in_use);

fold_Cdc25C = ATP_frac_avail*k_fold_p*Cdc25C_unfold_conc;

phospho_Cdc25C_by_Cdk1 = k_phospho_Cdc25C_by_cycA_Cdk1...

*cycA_Cdk1_cytosol_active_conc...

*Cdc25C_cytosol_inactive_conc...

+ k_phospho_Cdc25C_by_cycB_Cdk1...

*cycB_Cdk1_cytosol_active_conc...

*Cdc25C_cytosol_inactive_conc;

phospho_Cdc25C_by_Plk1 = k_phospho_Cdc25C_by_Plk1...

*Plk1_cytosol_phospho_active_conc...

*Cdc25C_cytosol_inactive_conc;

unphospho_Cdc25C = k_unphospho_Cdc25C*Cdc25C_cytosol_phospho_active_conc;

% Cdc25C must enter nucleus to be inactivated by Cdc14

% ASSUME no ATP is necessary

% ASSUME that inactivated Cdc25C ends up back in the cytosol

nm_in_Cdc25C = k_nm_in_Cdc25C...

*Plk1_cytosol_phospho_active_conc...

*Cdc25C_cytosol_phospho_active_conc;

nm_out_Cdc25C = k_nm_out_Cdc25C*Cdc25C_nuc_phospho_active_conc;

dephospho_Cdc25C_by_Cdc14 = k_dephospho_Cdc25C_by_Cdc14*Cdc14_phospho_active_conc...

*Cdc25C_nuc_phospho_active_conc...

+ k_dephospho_Cdc25C*Cdc25C_nuc_phospho_active_conc;

u_Cdc25C_unfold = ATP_frac_avail*k_dk_p*Cdc25C_unfold;

u_Cdc25C_cytosol_inactive = ATP_frac_avail*k_dk_p*Cdc25C_cytosol_inactive;

u_Cdc25C_cytosol_phospho_active = ATP_frac_avail*k_dk_p...

*Cdc25C_cytosol_phospho_active;

u_Cdc25C_nuc_phospho_active = ATP_frac_avail*k_dk_p*Cdc25C_nuc_phospho_active;

cut_Cdc25C = ATP_frac_avail*k_cut_p*Cdc25C_trash_conc;

% 22 Plk1 RATES

% txs_mRNA_Plk1 = ATP_frac_avail*k_mRNA_txs*polyII*Plk1_loci_frac...

% *(E2F_on_DNA + B_Myb_on_DNA + NF_Y_on_DNA + TF_grow_on_DNA);

txs_mRNA_Plk1 = ATP_frac_avail*k_mRNA_txs*polyII*Plk1_loci_frac...

*(E2F_on_DNA + TF_grow_on_DNA);

spl_mRNA_Plk1 = ATP_frac_avail*spl_frac*mRNA_Plk1_unspl;

nm_out_mRNA_Plk1 = ATP_frac_avail*k_nm_mRNA*mRNA_Plk1_nuc_conc;

activate_mRNA_Plk1 = activate_mRNA*mRNA_Plk1_inactive/mRNA_inactive;

bind_mRNA_Plk1 = bind_mRNA*mRNA_Plk1_cytosol/mRNA_cytosol;

unbind_mRNA_Plk1 = unbind_mRNA*mRNA_Plk1_in_use/mRNA_in_use;

dk_mRNA_Plk1_cytosol = k_dk_mRNA*mRNA_Plk1_cytosol;

dk_mRNA_Plk1_unspl = k_dk_mRNA*mRNA_Plk1_unspl;

dk_mRNA_Plk1_nuc = k_dk_mRNA*mRNA_Plk1_nuc;

dk_mRNA_Plk1_inactive = k_dk_mRNA*mRNA_Plk1_inactive;

dk_mRNA_Plk1_in_use = k_dk_mRNA*mRNA_Plk1_in_use;

cut_mRNA_Plk1 = k_cut_mRNA*mRNA_Plk1_trash;

txl_Plk1 = unbind_rRNA*(mRNA_Plk1_cytosol + mRNA_Plk1_in_use)...

/(mRNA_cytosol + mRNA_in_use);

fold_Plk1 = ATP_frac_avail*k_fold_p*Plk1_unfold_conc;

% Plk1 is cytoplasmic

phospho_Plk1_by_Cdk1 = (k_phospho_Plk1_by_cycA_Cdk1*cycA_Cdk1_cytosol_active_conc...

+ k_phospho_Plk1_by_cycB_Cdk1*cycB_Cdk1_cytosol_active_conc)...

*Plk1_cytosol_inactive_conc;

unphospho_Plk1 = k_unphospho_Plk1*Plk1_cytosol_phospho_active_conc;

% Plk1 must enter nucleus to activate Cdc14 and be ubiq by APC(Cdh1)

% ASSUME no ATP is necessary

nm_in_Plk1 = k_nm_in_Plk1*Plk1_cytosol_phospho_active_conc;

u_Plk1_by_APC_Cdh1 = k_u_Plk1_by_APC_Cdh1*Cdh1_APC_active_conc;

u_Plk1_unfold = ATP_frac_avail*k_dk_p*Plk1_unfold;

u_Plk1_cytosol_inactive = u_Plk1_by_APC_Cdh1*Plk1_cytosol_inactive_conc...

+ ATP_frac_avail*k_dk_p*Plk1_cytosol_inactive;

u_Plk1_cytosol_phospho_active = u_Plk1_by_APC_Cdh1*Plk1_cytosol_phospho_active_conc...

+ ATP_frac_avail*k_dk_p*Plk1_cytosol_phospho_active;

u_Plk1_nuc_phospho_active = u_Plk1_by_APC_Cdh1*Plk1_nuc_phospho_active_conc...

+ ATP_frac_avail*k_dk_p*Plk1_nuc_phospho_active;

cut_Plk1 = ATP_frac_avail*k_cut_p*Plk1_trash_conc;

energy_u_Plk1_by_APC_Cdh1 = ATP_u*u_Plk1_by_APC_Cdh1...

*(Plk1_cytosol_inactive_conc...

+ Plk1_cytosol_phospho_active_conc...

+ Plk1_nuc_phospho_active_conc);

% 23 Emi1 RATES

txs_mRNA_Emi1 = ATP_frac_avail*k_mRNA_txs*polyII*Emi1_loci_frac*E2F_on_DNA;

spl_mRNA_Emi1 = ATP_frac_avail*spl_frac*mRNA_Emi1_unspl;

nm_out_mRNA_Emi1 = ATP_frac_avail*k_nm_mRNA*mRNA_Emi1_nuc_conc;

activate_mRNA_Emi1 = activate_mRNA*mRNA_Emi1_inactive/mRNA_inactive;

bind_mRNA_Emi1 = bind_mRNA*mRNA_Emi1_cytosol/mRNA_cytosol;

unbind_mRNA_Emi1 = unbind_mRNA*mRNA_Emi1_in_use/mRNA_in_use;

dk_mRNA_Emi1_cytosol = k_dk_mRNA*mRNA_Emi1_cytosol;

dk_mRNA_Emi1_unspl = k_dk_mRNA*mRNA_Emi1_unspl;

dk_mRNA_Emi1_nuc = k_dk_mRNA*mRNA_Emi1_nuc;

dk_mRNA_Emi1_inactive = k_dk_mRNA*mRNA_Emi1_inactive;

dk_mRNA_Emi1_in_use = k_dk_mRNA*mRNA_Emi1_in_use;

cut_mRNA_Emi1 = k_cut_mRNA*mRNA_Emi1_trash;

txl_Emi1 = unbind_rRNA*(mRNA_Emi1_cytosol + mRNA_Emi1_in_use)...

/(mRNA_cytosol + mRNA_in_use);

fold_Emi1 = ATP_frac_avail*k_fold_p*Emi1_unfold_conc;

bind_Emi1_to_Cdh1 = k_bind_Emi1_to_Cdh1*Cdh1_phospho_inactive_conc...

*Emi1_in_cell_conc;

unbind_Emi1_Cdh1 = k_unbind_Emi1_Cdh1*Emi1_on_Cdh1_conc;

bind_Emi1_to_Cdc20 = k_bind_Emi1_to_Cdc20*Emi1_in_cell_conc*Cdc20_nuc_conc;

unbind_Emi1_Cdc20 = k_unbind_Emi1_Cdc20*Emi1_on_Cdc20_conc;

% WARNING! I am assuming that cycB/Cdk1 is the major phosphorylator

% phospho_Emi1 = k_phospho_Emi1_by_Cdk1...

% *(Plk1_nuc_phospho_active_conc...

% + cycA_Cdk1_nuc_active_conc + cycB_Cdk1_nuc_active_conc);

phospho_Emi1 = k_phospho_Emi1_by_cycA_Cdk1*cycA_Cdk1_nuc_active_conc...

+ k_phospho_Emi1_by_cycA_Cdk1*cycB_Cdk1_nuc_active_conc;

phospho_Emi1_in_cell = phospho_Emi1*Emi1_in_cell_conc;

phospho_Emi1_on_Cdh1 = phospho_Emi1*Emi1_on_Cdh1_conc;

phospho_Emi1_on_Cdc20 = phospho_Emi1*Emi1_on_Cdc20_conc;

% ASSUME Emi1 is nuclear when bound to Cdh1 and Cdc20,

% but passes easily from nuc to cytosol when phophorylated to be ubiq by SCF(Btrc)

u_Emi1_unfold = ATP_frac_avail*k_dk_p*Emi1_unfold;

u_Emi1_in_cell = ATP_frac_avail*k_dk_p*Emi1_in_cell;

u_Emi1_on_Cdh1 = ATP_frac_avail*k_dk_p*Emi1_on_Cdh1;

u_Emi1_on_Cdc20 = ATP_frac_avail*k_dk_p*Emi1_on_Cdc20;

% WARNING! I am assuming the SCF(Skp2) also ubiqs Emi1

% u_Emi1_by_SCF_Btrc = k_u_Emi1_by_SCF_Btrc*Btrc_SCF_active_conc...

% *Emi1_phospho_inactive_conc;

u_Emi1_by_SCF_Btrc = (k_u_Emi1_by_SCF_Skp2_test*Skp2_SCF_active_conc...

+ k_u_Emi1_by_SCF_Btrc*Btrc_SCF_active_conc)...

*Emi1_phospho_inactive_conc;

u_Emi1_phospho_inactive = u_Emi1_by_SCF_Btrc*Emi1_phospho_inactive_conc...

+ ATP_frac_avail*k_dk_p*Emi1_phospho_inactive;

cut_Emi1 = ATP_frac_avail*k_cut_p*Emi1_trash_conc;

energy_u_Emi1_by_SCF_Btrc = ATP_u*u_Emi1_by_SCF_Btrc*Emi1_phospho_inactive_conc;

% 25 Cdh1 RATES

txs_mRNA_Cdh1 = ATP_frac_avail*k_mRNA_txs*polyII*Cdh1_loci_frac;

spl_mRNA_Cdh1 = ATP_frac_avail*spl_frac*mRNA_Cdh1_unspl;

nm_out_mRNA_Cdh1 = ATP_frac_avail*k_nm_mRNA*mRNA_Cdh1_nuc_conc;

activate_mRNA_Cdh1 = activate_mRNA*mRNA_Cdh1_inactive/mRNA_inactive;

bind_mRNA_Cdh1 = bind_mRNA*mRNA_Cdh1_cytosol/mRNA_cytosol;

unbind_mRNA_Cdh1 = unbind_mRNA*mRNA_Cdh1_in_use/mRNA_in_use;

dk_mRNA_Cdh1_cytosol = k_dk_mRNA*mRNA_Cdh1_cytosol;

dk_mRNA_Cdh1_unspl = k_dk_mRNA*mRNA_Cdh1_unspl;

dk_mRNA_Cdh1_nuc = k_dk_mRNA*mRNA_Cdh1_nuc;

dk_mRNA_Cdh1_inactive = k_dk_mRNA*mRNA_Cdh1_inactive;

dk_mRNA_Cdh1_in_use = k_dk_mRNA*mRNA_Cdh1_in_use;

cut_mRNA_Cdh1 = k_cut_mRNA*mRNA_Cdh1_trash;

txl_Cdh1 = unbind_rRNA*(mRNA_Cdh1_cytosol + mRNA_Cdh1_in_use)...

/(mRNA_cytosol + mRNA_in_use);

fold_Cdh1 = ATP_frac_avail*k_fold_p*Cdh1_unfold_conc;

% nm_in_Cdh1 = ATP_frac_avail*k_nm_in_p*Cdh1_cytosol_conc;

% Emi1 binds and inactivates Cdh1

bind_Cdh1_to_Emi1 = bind_Emi1_to_Cdh1;

unbind_Cdh1_Emi1 = unbind_Emi1_Cdh1;

% Cdk phosphorylates and inactivates Cdh1

% ASSUME: cycD_Cdk4or6 must release Rb from E2F before it can inhibit Cdh1

k_Cdk_active = (k_phospho_Cdh1_by_cycD_Cdk4or6...

*cycD_Cdk4or6_active_conc...

+ k_phospho_Cdh1_by_cycD_Cdk4or6_on_p27...

*cycD_Cdk4or6_on_p27_inactive_conc)...

/max(1, Rb_nuc_conc + Rb_on_E2F_conc)...

+ k_phospho_Cdh1_by_cycE_Cdk2*cycE_Cdk2_active_conc...

+ k_phospho_Cdh1_by_cycA_Cdk2*cycA_Cdk2_active_conc...

+ k_phospho_Cdh1_by_Cdk1...

*(cycA_Cdk1_nuc_active_conc + cycB_Cdk1_nuc_active_conc);

phospho_Cdh1_in_cell_by_Cdk1 = k_Cdk_active*Cdh1_in_cell_conc;

% phospho_Cdh1_nuc_by_Cdk1 = k_Cdk_active*Cdh1_nuc_conc;

% Cdc14 dephosphorylates and reactivates Cdh1

dephospho_Cdh1_by_Cdc14 = k_dephospho_Cdh1_by_Cdc14*Cdc14_phospho_active_conc...

*Cdh1_phospho_inactive_conc;

bind_Cdh1_to_APC = k_bind_Cdh1_to_APC*APC_in_cell_conc*Cdh1_in_cell_conc;

% bind_Cdh1_to_APC = k_bind_Cdh1_to_APC*APC_nuc_conc*Cdh1_nuc_conc;

unbind_Cdh1_APC = k_unbind_Cdh1_APC*Cdh1_APC_active_conc;

phospho_Cdh1_APC_active_by_Cdk = k_Cdk_active*Cdh1_APC_active_conc;

u_Cdh1_unfold = ATP_frac_avail*k_dk_p*Cdh1_unfold;

u_Cdh1_in_cell = ATP_frac_avail*k_dk_p*Cdh1_in_cell;

% u_Cdh1_cytosol = ATP_frac_avail*k_dk_p*Cdh1_cytosol;

% u_Cdh1_nuc = ATP_frac_avail*k_dk_p*Cdh1_nuc;

u_Cdh1_Emi1_inactive = ATP_frac_avail*k_dk_p*Cdh1_Emi1_inactive;

u_Cdh1_phospho_inactive = ATP_frac_avail*k_dk_p*Cdh1_phospho_inactive;

% Cdh1 autoubiquinates when bound to APC and there are no substrates

% substrates are Skp2, cycA, cycB, Cdc25A, Plk1, RC, and Cdc20 (ignore p27)

% (autoubiquination is dependent on number of molecules, not concentration)

substrates_Cdh1_all = Skp2_cytosol + Skp2_nuc + Skp2_SCF_active...

+ SCF_in_cell + SCF_on_Btrc...

+ SCF_on_Fbw7 + SCF_on_Skp2...

+ cycA_cytosol + cycA_nuc...

+ cycA_Cdk2_inactive + cycA_Cdk2_active...

+ cycA_Cdk2_on_p27_inactive...

+ cycA_Cdk1_cytosol_active + cycA_Cdk1_phospho_inactive...

+ cycA_Cdk1_nuc_active...

+ cycB_Cdk1_cytosol_active + cycB_Cdk1_phospho_inactive...

+ cycB_Cdk1_nuc_active...

+ Cdc25A_cytosol + Cdc25A_nuc_inactive...

+ Cdc25A_phospho_active...

+ Plk1_cytosol_inactive...

+ Plk1_cytosol_phospho_active + Plk1_nuc_phospho_active...

+ RC_cytosol + RC_nuc + RC_on_DNA...

+ RC_licensed + RC_traversed_by_DNA_poly...

+ Cdc20_cytosol + Cdc20_nuc + Cdc20_Emi1_inactive...

+ Cdc20_APC_inactive + Cdc20_APC_active;

% substrates_Cdh1_all = Skp2_nuc + Skp2_SCF_active...

% + SCF_in_cell + SCF_on_Fbw7 + SCF_on_Skp2...

% + cycA_nuc + cycA_Cdk2_inactive + cycA_Cdk2_active...

% + cycA_Cdk2_on_p27_inactive + cycA_Cdk1_nuc_active...

% + cycB_Cdk1_nuc_active...

% + Cdc25A_nuc_inactive + Cdc25A_phospho_active...

% + Plk1_nuc_phospho_active...

% + RC_nuc + RC_on_DNA...

% + RC_licensed + RC_traversed_by_DNA_poly...

% + Cdc20_nuc + Cdc20_Emi1_inactive...

% + Cdc20_APC_inactive + Cdc20_APC_active;

u_Cdh1_APC_active = ATP_frac_avail*k_u_auto_Cdh1_APC_active*Cdh1_APC_active...

/max(1, substrates_Cdh1_all)...

+ ATP_frac_avail*k_dk_p*Cdh1_APC_active;

cut_Cdh1 = ATP_frac_avail*k_cut_p*Cdh1_trash_conc;

energy_u_auto_Cdh1_APC = ATP_u*ATP_frac_avail*k_u_auto_Cdh1_APC_active...

*Cdh1_APC_active/max(1, substrates_Cdh1_all);

% 26 Cdc20 RATES

txs_mRNA_Cdc20 = ATP_frac_avail*k_mRNA_txs*polyII*Cdc20_loci_frac;

spl_mRNA_Cdc20 = ATP_frac_avail*spl_frac*mRNA_Cdc20_unspl;

nm_out_mRNA_Cdc20 = ATP_frac_avail*k_nm_mRNA*mRNA_Cdc20_nuc_conc;

activate_mRNA_Cdc20 = activate_mRNA*mRNA_Cdc20_inactive/mRNA_inactive;

bind_mRNA_Cdc20 = bind_mRNA*mRNA_Cdc20_cytosol/mRNA_cytosol;

unbind_mRNA_Cdc20 = unbind_mRNA*mRNA_Cdc20_in_use/mRNA_in_use;

dk_mRNA_Cdc20_cytosol = k_dk_mRNA*mRNA_Cdc20_cytosol;

dk_mRNA_Cdc20_unspl = k_dk_mRNA*mRNA_Cdc20_unspl;

dk_mRNA_Cdc20_nuc = k_dk_mRNA*mRNA_Cdc20_nuc;

dk_mRNA_Cdc20_inactive = k_dk_mRNA*mRNA_Cdc20_inactive;

dk_mRNA_Cdc20_in_use = k_dk_mRNA*mRNA_Cdc20_in_use;

cut_mRNA_Cdc20 = k_cut_mRNA*mRNA_Cdc20_trash;

txl_Cdc20 = unbind_rRNA*(mRNA_Cdc20_cytosol + mRNA_Cdc20_in_use)...

/(mRNA_cytosol + mRNA_in_use);

fold_Cdc20 = ATP_frac_avail*k_fold_p*Cdc20_unfold_conc;

nm_in_Cdc20 = ATP_frac_avail*k_nm_in_p*Cdc20_cytosol_conc;

bind_Cdc20_to_Emi1 = bind_Emi1_to_Cdc20;

unbind_Cdc20_Emi1 = unbind_Emi1_Cdc20;

bind_Cdc20_to_APC = k_bind_Cdc20_to_APC*APC_in_cell_conc*Cdc20_nuc_conc;

% bind_Cdc20_to_APC = k_bind_Cdc20_to_APC*APC_nuc_conc*Cdc20_nuc_conc;

unbind_Cdc20_APC = k_unbind_Cdc20_APC*Cdc20_APC_inactive_conc;

dephospho_Cdc20_APC_inactive_by_Cdc14 = k_dephospho_Cdc20_APC_by_Cdc14...

*Cdc14_phospho_active_conc*Cdc20_APC_inactive_conc;

dephospho_Cdc20_APC_active_by_Cdc14 = k_dephospho_Cdc20_APC_by_Cdc14...

*Cdc14_phospho_active_conc*Cdc20_APC_active_conc;

Cdk_all_conc = cycA_Cdk1_nuc_active_conc + cycB_Cdk1_nuc_active_conc;

phospho_Cdc20_by_Cdk1 = k_phospho_Cdc20_by_Cdk1...

*Cdk_all_conc*Cdc20_APC_inactive_conc;

unphospho_Cdc20_APC = k_unphospho_Cdc20_APC_active*Cdc20_APC_active_conc;

u_Cdc20_by_APC_Cdh1 = k_u_Cdc20_by_APC_Cdh1*Cdh1_APC_active_conc;

u_Cdc20_unfold = ATP_frac_avail*k_dk_p*Cdc20_unfold;

u_Cdc20_cytosol = u_Cdc20_by_APC_Cdh1*Cdc20_cytosol_conc...

+ ATP_frac_avail*k_dk_p*Cdc20_cytosol;

u_Cdc20_nuc = u_Cdc20_by_APC_Cdh1*Cdc20_nuc_conc...

+ ATP_frac_avail*k_dk_p*Cdc20_nuc;

u_Cdc20_Emi1_inactive = u_Cdc20_by_APC_Cdh1*Cdc20_Emi1_inactive_conc...

+ ATP_frac_avail*k_dk_p*Cdc20_Emi1_inactive;

u_Cdc20_APC_inactive = u_Cdc20_by_APC_Cdh1*Cdc20_APC_inactive_conc...

+ ATP_frac_avail*k_dk_p*Cdc20_APC_inactive;

u_Cdc20_APC_active = u_Cdc20_by_APC_Cdh1*Cdc20_APC_active_conc...

+ ATP_frac_avail*k_dk_p*Cdc20_APC_active;

cut_Cdc20 = ATP_frac_avail*k_cut_p*Cdc20_trash_conc;

energy_u_Cdc20_by_APC_Cdh1 = ATP_u*u_Cdc20_by_APC_Cdh1...

*(Cdc20_cytosol_conc + Cdc20_nuc_conc...

+ Cdc20_Emi1_inactive_conc + Cdc20_APC_inactive_conc...

+ Cdc20_APC_active_conc);

% 24 APC RATES

txs_mRNA_APC = ATP_frac_avail*k_mRNA_txs*polyII*APC_loci_frac;

spl_mRNA_APC = ATP_frac_avail*spl_frac*mRNA_APC_unspl;

nm_out_mRNA_APC = ATP_frac_avail*k_nm_mRNA*mRNA_APC_nuc_conc;

activate_mRNA_APC = activate_mRNA*mRNA_APC_inactive/mRNA_inactive;

bind_mRNA_APC = bind_mRNA*mRNA_APC_cytosol/mRNA_cytosol;

unbind_mRNA_APC = unbind_mRNA*mRNA_APC_in_use/mRNA_in_use;

dk_mRNA_APC_cytosol = k_dk_mRNA*mRNA_APC_cytosol;

dk_mRNA_APC_unspl = k_dk_mRNA*mRNA_APC_unspl;

dk_mRNA_APC_nuc = k_dk_mRNA*mRNA_APC_nuc;

dk_mRNA_APC_inactive = k_dk_mRNA*mRNA_APC_inactive;

dk_mRNA_APC_in_use = k_dk_mRNA*mRNA_APC_in_use;

cut_mRNA_APC = k_cut_mRNA*mRNA_APC_trash;

txl_APC = unbind_rRNA*(mRNA_APC_cytosol + mRNA_APC_in_use)...

/(mRNA_cytosol + mRNA_in_use);

fold_APC = ATP_frac_avail*k_fold_p*APC_unfold_conc;

% nm_in_APC = ATP_frac_avail*k_nm_in_p*APC_cytosol_conc;

bind_APC_to_Cdh1 = bind_Cdh1_to_APC;

unbind_APC_Cdh1 = unbind_Cdh1_APC + phospho_Cdh1_APC_active_by_Cdk;

bind_APC_to_Cdc20 = bind_Cdc20_to_APC;

unbind_APC_Cdc20 = unbind_Cdc20_APC + dephospho_Cdc20_APC_inactive_by_Cdc14...

+ dephospho_Cdc20_APC_active_by_Cdc14;

u_APC_unfold = ATP_frac_avail*k_dk_p*APC_unfold;

u_APC_in_cell = ATP_frac_avail*k_dk_p*APC_in_cell;

% u_APC_cytosol = ATP_frac_avail*k_dk_p*APC_cytosol;

% u_APC_nuc = ATP_frac_avail*k_dk_p*APC_nuc;

u_APC_on_Cdh1 = ATP_frac_avail*k_dk_p*APC_on_Cdh1;

u_APC_on_Cdc20 = ATP_frac_avail*k_dk_p*APC_on_Cdc20;

cut_APC = ATP_frac_avail*k_cut_p*APC_trash_conc;

% 27 Cdc14 RATES

txs_mRNA_Cdc14 = ATP_frac_avail*k_mRNA_txs*polyII*Cdc14_loci_frac;

spl_mRNA_Cdc14 = ATP_frac_avail*spl_frac*mRNA_Cdc14_unspl;

nm_out_mRNA_Cdc14 = ATP_frac_avail*k_nm_mRNA*mRNA_Cdc14_nuc_conc;

activate_mRNA_Cdc14 = activate_mRNA*mRNA_Cdc14_inactive/mRNA_inactive;

bind_mRNA_Cdc14 = bind_mRNA*mRNA_Cdc14_cytosol/mRNA_cytosol;

unbind_mRNA_Cdc14 = unbind_mRNA*mRNA_Cdc14_in_use/mRNA_in_use;

dk_mRNA_Cdc14_cytosol = k_dk_mRNA*mRNA_Cdc14_cytosol;

dk_mRNA_Cdc14_unspl = k_dk_mRNA*mRNA_Cdc14_unspl;

dk_mRNA_Cdc14_nuc = k_dk_mRNA*mRNA_Cdc14_nuc;

dk_mRNA_Cdc14_inactive = k_dk_mRNA*mRNA_Cdc14_inactive;

dk_mRNA_Cdc14_in_use = k_dk_mRNA*mRNA_Cdc14_in_use;

cut_mRNA_Cdc14 = k_cut_mRNA*mRNA_Cdc14_trash;

txl_Cdc14 = unbind_rRNA*(mRNA_Cdc14_cytosol + mRNA_Cdc14_in_use)...

/(mRNA_cytosol + mRNA_in_use);

fold_Cdc14 = ATP_frac_avail*k_fold_p*Cdc14_unfold_conc;

nm_in_Cdc14 = ATP_frac_avail*k_nm_in_p*Cdc14_cytosol_conc;

% Securin inhibits Cdc14

inhibit_Cdc14_by_Securin = k_inhibit_Cdc14_by_Securin...

*(Securin_nuc_conc + Securin_on_chromo_conc)...

*Cdc14_nuc_inactive_conc;

uninhibit_Cdc14 = k_uninhibit_Cdc14*Cdc14_inhibited_conc;

phospho_Cdc14_by_Plk1 = k_phospho_Cdc14_by_Plk1*Plk1_nuc_phospho_active_conc...

*Cdc14_nuc_inactive_conc;

unphospho_Cdc14 = k_unphospho_Cdc14*Cdc14_phospho_active_conc;

u_Cdc14_unfold = ATP_frac_avail*k_dk_p*Cdc14_unfold;

u_Cdc14_cytosol = ATP_frac_avail*k_dk_p*Cdc14_cytosol;

u_Cdc14_nuc_inactive = ATP_frac_avail*k_dk_p*Cdc14_nuc_inactive;

u_Cdc14_inhibited = ATP_frac_avail*k_dk_p*Cdc14_inhibited;

u_Cdc14_phospho_active = ATP_frac_avail*k_dk_p*Cdc14_phospho_active;

cut_Cdc14 = ATP_frac_avail*k_cut_p*Cdc14_trash_conc;

% 28 Cdc25A RATES

% txs_mRNA_Cdc25A = ATP_frac_avail*k_mRNA_txs*polyII*Cdc25A_loci_frac...

% *(E2F_on_DNA + TF_grow_on_DNA);

% NOTE: txs also promoted by c-myc (here TF_grow).

% NOTE: ASSUME constitutive txs component.

txs_mRNA_Cdc25A = ATP_frac_avail*k_mRNA_txs*polyII*Cdc25A_loci_frac...

*(Cdc25A_constitutive + E2F_on_DNA...

+ TF_grow_on_DNA);

spl_mRNA_Cdc25A = ATP_frac_avail*spl_frac*mRNA_Cdc25A_unspl;

nm_out_mRNA_Cdc25A = ATP_frac_avail*k_nm_mRNA*mRNA_Cdc25A_nuc_conc;

activate_mRNA_Cdc25A = activate_mRNA*mRNA_Cdc25A_inactive/mRNA_inactive;

bind_mRNA_Cdc25A = bind_mRNA*mRNA_Cdc25A_cytosol/mRNA_cytosol;

unbind_mRNA_Cdc25A = unbind_mRNA*mRNA_Cdc25A_in_use/mRNA_in_use;

dk_mRNA_Cdc25A_cytosol = k_dk_mRNA*mRNA_Cdc25A_cytosol;

dk_mRNA_Cdc25A_unspl = k_dk_mRNA*mRNA_Cdc25A_unspl;

dk_mRNA_Cdc25A_nuc = k_dk_mRNA*mRNA_Cdc25A_nuc;

dk_mRNA_Cdc25A_inactive = k_dk_mRNA*mRNA_Cdc25A_inactive;

dk_mRNA_Cdc25A_in_use = k_dk_mRNA*mRNA_Cdc25A_in_use;

cut_mRNA_Cdc25A = k_cut_mRNA*mRNA_Cdc25A_trash;

txl_Cdc25A = unbind_rRNA*(mRNA_Cdc25A_cytosol + mRNA_Cdc25A_in_use)...

/(mRNA_cytosol + mRNA_in_use);

fold_Cdc25A = ATP_frac_avail*k_fold_p*Cdc25A_unfold_conc;

nm_in_Cdc25A = ATP_frac_avail*k_nm_in_p*Cdc25A_cytosol_conc;

% Cdc25A is activated when phosphorylated

phospho_Cdc25A_by_Cdk2 = k_phospho_Cdc25A_by_Cdk2*Cdc25A_nuc_inactive_conc...

*(cycE_Cdk2_active_conc + cycA_Cdk2_active_conc);

% WARNING!!! Cdc25A (supposedly nuclear) is being phosphoed by cyto cycB/Cdk1 and cycA/Cdk1!!!

% phospho_Cdc25A_by_Cdk1 = k_phospho_Cdc25A_by_Cdk1*Cdc25A_nuc_inactive_conc...

% *(cycB_Cdk1_nuc_active_conc + cycA_Cdk1_nuc_active_conc);

% phospho_Cdc25A_by_Cdk1 = k_phospho_Cdc25A_by_Cdk1*Cdc25A_nuc_inactive_conc...

% *(cycB_Cdk1_nuc_active_conc + cycA_Cdk1_nuc_active_conc...

% + cycB_Cdk1_cytosol_active_conc...

% + cycA_Cdk1_cytosol_active_conc);

phospho_Cdc25A_by_Cdk1 = Cdc25A_nuc_inactive_conc...

*(k_phospho_Cdc25A_by_cycB_Cdk1...

*(cycB_Cdk1_nuc_active_conc...

+ cycB_Cdk1_cytosol_active_conc)...

+ k_phospho_Cdc25A_by_cycA_Cdk1...

*(cycA_Cdk1_nuc_active_conc...

+ cycA_Cdk1_cytosol_active_conc));

unphospho_Cdc25A = k_dephospho_Cdc25A*Cdc25A_phospho_active_conc;

dephospho_Cdc25A_by_Cdc14 = k_dephospho_Cdc25A_by_Cdc14*Cdc14_phospho_active_conc...

*Cdc25A_phospho_active_conc...

+ unphospho_Cdc25A;

% Cdc25A is ubiq by APC(Cdh1) and by SCF(Btrc)

% NOTE SCF(Btrc) is cytoplasmic: ASSUME Cdc25A ubiq by SCF(Btrc) for checkpoint control only

u_Cdc25A_by_SCF_Btrc = ATP_frac_avail*k_u_Cdc25A_by_SCF_Btrc...

*Btrc_SCF_active_conc;

u_Cdc25A_by_APC_Cdh1 = ATP_frac_avail*k_u_Cdc25A_by_APC_Cdh1...

*Cdh1_APC_active_conc;

u_Cdc25A_unfold = ATP_frac_avail*k_dk_p*Cdc25A_unfold;

u_Cdc25A_cytosol = u_Cdc25A_by_SCF_Btrc*Cdc25A_cytosol_conc...

+ u_Cdc25A_by_APC_Cdh1*Cdc25A_cytosol_conc...

+ ATP_frac_avail*k_dk_p*Cdc25A_cytosol;

% WARNING! I am ubiquintinating nuclear Cdc25A with cytoplasmic SCF(Btrc)!!!!!!!!!!!!!!

% u_Cdc25A_nuc_inactive = u_Cdc25A_by_APC_Cdh1*Cdc25A_nuc_inactive_conc...

% + ATP_frac_avail*k_dk_p*Cdc25A_nuc_inactive;

% u_Cdc25A_phospho_active = u_Cdc25A_by_APC_Cdh1*Cdc25A_phospho_active_conc...

% + ATP_frac_avail*k_dk_p*Cdc25A_phospho_active;

u_Cdc25A_nuc_inactive = u_Cdc25A_by_SCF_Btrc*Cdc25A_nuc_inactive_conc...

+ u_Cdc25A_by_APC_Cdh1*Cdc25A_nuc_inactive_conc...

+ ATP_frac_avail*k_dk_p*Cdc25A_nuc_inactive;

u_Cdc25A_phospho_active = u_Cdc25A_by_SCF_Btrc*Cdc25A_phospho_active_conc...

+ u_Cdc25A_by_APC_Cdh1*Cdc25A_phospho_active_conc...

+ ATP_frac_avail*k_dk_p*Cdc25A_phospho_active;

cut_Cdc25A = ATP_frac_avail*k_cut_p*Cdc25A_trash_conc;

energy_u_Cdc25A_by_APC_Cdh1 = ATP_u*u_Cdc25A_by_APC_Cdh1...

*(Cdc25A_cytosol_conc...

+ Cdc25A_nuc_inactive_conc...

+ Cdc25A_phospho_active_conc);

energy_u_Cdc25A_by_SCF_Btrc = ATP_u*u_Cdc25A_by_SCF_Btrc...

*(Cdc25A_cytosol_conc...

+ Cdc25A_nuc_inactive_conc...

+ Cdc25A_phospho_active_conc);

% 29 Cdc25B RATES

txs_mRNA_Cdc25B = ATP_frac_avail*k_mRNA_txs*polyII*Cdc25B_loci_frac...

*(E2F_on_DNA + TF_grow_on_DNA);

spl_mRNA_Cdc25B = ATP_frac_avail*spl_frac*mRNA_Cdc25B_unspl;

nm_out_mRNA_Cdc25B = ATP_frac_avail*k_nm_mRNA*mRNA_Cdc25B_nuc_conc;

activate_mRNA_Cdc25B = activate_mRNA*mRNA_Cdc25B_inactive/mRNA_inactive;

bind_mRNA_Cdc25B = bind_mRNA*mRNA_Cdc25B_cytosol/mRNA_cytosol;

unbind_mRNA_Cdc25B = unbind_mRNA*mRNA_Cdc25B_in_use/mRNA_in_use;

dk_mRNA_Cdc25B_cytosol = k_dk_mRNA*mRNA_Cdc25B_cytosol;

dk_mRNA_Cdc25B_unspl = k_dk_mRNA*mRNA_Cdc25B_unspl;

dk_mRNA_Cdc25B_nuc = k_dk_mRNA*mRNA_Cdc25B_nuc;

dk_mRNA_Cdc25B_inactive = k_dk_mRNA*mRNA_Cdc25B_inactive;

dk_mRNA_Cdc25B_in_use = k_dk_mRNA*mRNA_Cdc25B_in_use;

cut_mRNA_Cdc25B = k_cut_mRNA*mRNA_Cdc25B_trash;

txl_Cdc25B = unbind_rRNA*(mRNA_Cdc25B_cytosol + mRNA_Cdc25B_in_use)...

/(mRNA_cytosol + mRNA_in_use);

fold_Cdc25B = ATP_frac_avail*k_fold_p*Cdc25B_unfold_conc;

% Cdc25B is cytoplasmic: ASSUME active Cdk2 is nuclear

phospho_Cdc25B_by_Cdk2 = 0;

phospho_Cdc25B_by_Cdk1 = k_phospho_Cdc25B_by_cycA_Cdk1...

*cycA_Cdk1_cytosol_active_conc...

*Cdc25B_cytosol_inactive_conc...

+ k_phospho_Cdc25B_by_cycB_Cdk1...

*cycB_Cdk1_cytosol_active_conc...

*Cdc25B_cytosol_inactive_conc;

% ASSUME c-myc does not activate Cdc25B (maybe because c-myc is nuclear)

phospho_Cdc25B_by_TF_grow = 0;

phospho_Cdc25B_by_Plk1 = k_phospho_Cdc25B_by_Plk1...

*Plk1_cytosol_phospho_active_conc...

*Cdc25B_cytosol_inactive_conc;

dephospho_Cdc25B_by_Cdc14 = k_dephospho_Cdc25B_by_Cdc14*Cdc14_phospho_active_conc...

*Cdc25B_cytosol_phospho_active_conc...

+ k_dephospho_Cdc25B*Cdc25B_cytosol_phospho_active_conc;

% ASSUME Cdc25B ubiq by SCF(Btrc)

% or ASSUME Cdc25B ubiq by SCF(Btrc) for checkpoint control only (k_u_Cdc25B_by_SCF_Btrc = 0)

% ASSUME Cdc25B ubiq by APC(Cdh1)

u_Cdc25B_by_SCF_Btrc = ATP_frac_avail*k_u_Cdc25B_by_SCF_Btrc*Btrc_SCF_active_conc;

u_Cdc25B_by_APC_Cdh1 = ATP_frac_avail*k_u_Cdc25B_by_APC_Cdh1*Cdh1_APC_active_conc;

u_Cdc25B = u_Cdc25B_by_SCF_Btrc + u_Cdc25B_by_APC_Cdh1;

u_Cdc25B_unfold = ATP_frac_avail*k_dk_p*Cdc25B_unfold;

u_Cdc25B_cytosol_inactive = u_Cdc25B*Cdc25B_cytosol_inactive_conc...

+ ATP_frac_avail*k_dk_p*Cdc25B_cytosol_inactive;

u_Cdc25B_cytosol_phospho_active = u_Cdc25B*Cdc25B_cytosol_phospho_active_conc...

+ ATP_frac_avail*k_dk_p...

*Cdc25B_cytosol_phospho_active;

cut_Cdc25B = ATP_frac_avail*k_cut_p*Cdc25B_trash_conc;

Cdc25B_all_conc = Cdc25B_cytosol_inactive_conc...

+ Cdc25B_cytosol_phospho_active_conc;

energy_u_Cdc25B_by_SCF_Btrc = ATP_u*u_Cdc25B_by_SCF_Btrc*Cdc25B_all_conc;

energy_u_Cdc25B_by_APC_Cdh1 = ATP_u*u_Cdc25B_by_APC_Cdh1*Cdc25B_all_conc;

% 30 Securin RATES

txs_mRNA_Securin = ATP_frac_avail*k_mRNA_txs*polyII*Securin_loci_frac...

*E2F_on_DNA;

spl_mRNA_Securin = ATP_frac_avail*spl_frac*mRNA_Securin_unspl;

nm_out_mRNA_Securin = ATP_frac_avail*k_nm_mRNA*mRNA_Securin_nuc_conc;

activate_mRNA_Securin = activate_mRNA*mRNA_Securin_inactive/mRNA_inactive;

bind_mRNA_Securin = bind_mRNA*mRNA_Securin_cytosol/mRNA_cytosol;

unbind_mRNA_Securin = unbind_mRNA*mRNA_Securin_in_use/mRNA_in_use;

dk_mRNA_Securin_cytosol = k_dk_mRNA*mRNA_Securin_cytosol;

dk_mRNA_Securin_unspl = k_dk_mRNA*mRNA_Securin_unspl;

dk_mRNA_Securin_nuc = k_dk_mRNA*mRNA_Securin_nuc;

dk_mRNA_Securin_inactive = k_dk_mRNA*mRNA_Securin_inactive;

dk_mRNA_Securin_in_use = k_dk_mRNA*mRNA_Securin_in_use;

cut_mRNA_Securin = k_cut_mRNA*mRNA_Securin_trash;

txl_Securin = unbind_rRNA...

*(mRNA_Securin_cytosol + mRNA_Securin_in_use)...

/(mRNA_cytosol + mRNA_in_use);

fold_Securin = ATP_frac_avail*k_fold_p*Securin_unfold_conc;

nm_in_Securin = ATP_frac_avail*k_nm_in_p*Securin_cytosol_conc;

bind_Securin_to_chromo = k_bind_Securin_to_chromo*Securin_nuc_conc;

u_Securin_by_APC_Cdc20 = k_u_Securin_by_APC_Cdc20*Cdc20_APC_active_conc;

% u_Securin_by_APC_Cdc20 = k_u_Securin_by_APC_Cdc20*round(Cdc20_APC_active_conc);

u_Securin_unfold = ATP_frac_avail*k_dk_p*Securin_unfold;

u_Securin_cytosol = ATP_frac_avail*k_dk_p*Securin_cytosol;

u_Securin_nuc = u_Securin_by_APC_Cdc20*Securin_nuc_conc...

+ ATP_frac_avail*k_dk_p*Securin_nuc;

u_Securin_on_chromo = u_Securin_by_APC_Cdc20*Securin_on_chromo_conc...

+ ATP_frac_avail*k_dk_p*Securin_on_chromo;

cut_Securin = ATP_frac_avail*k_cut_p*Securin_trash_conc;

energy_u_Securin_by_APC_Cdc20 = ATP_u*u_Securin_by_APC_Cdc20...

*(Securin_nuc_conc + Securin_on_chromo_conc);

% 31 cycC/Cdk8

txs_mRNA_cycC = ATP_frac_avail*k_mRNA_txs*polyII*cycC_loci_frac;

spl_mRNA_cycC = ATP_frac_avail*spl_frac*mRNA_cycC_unspl;

nm_out_mRNA_cycC = ATP_frac_avail*k_nm_mRNA*mRNA_cycC_nuc_conc;

activate_mRNA_cycC = activate_mRNA*mRNA_cycC_inactive/mRNA_inactive;

bind_mRNA_cycC = bind_mRNA*mRNA_cycC_cytosol/mRNA_cytosol;

unbind_mRNA_cycC = unbind_mRNA*mRNA_cycC_in_use/mRNA_in_use;

dk_mRNA_cycC_unspl = k_dk_mRNA*mRNA_cycC_unspl;

dk_mRNA_cycC_nuc = k_dk_mRNA*mRNA_cycC_nuc;

dk_mRNA_cycC_inactive = k_dk_mRNA*mRNA_cycC_inactive;

dk_mRNA_cycC_cytosol = k_dk_mRNA*mRNA_cycC_cytosol;

dk_mRNA_cycC_in_use = k_dk_mRNA*mRNA_cycC_in_use;

cut_mRNA_cycC = k_cut_mRNA*mRNA_cycC_trash;

txl_cycC = unbind_rRNA*(mRNA_cycC_cytosol + mRNA_cycC_in_use)...

/(mRNA_cytosol + mRNA_in_use);

fold_cycC = ATP_frac_avail*k_fold_p*cycC_unfold_conc;

nm_in_cycC = ATP_frac_avail*k_nm_in_p*cycC_cytosol_conc;

bind_cycC_to_Cdk8 = k_bind_cycC_to_Cdk8*cycC_nuc_conc;

unbind_cycC_from_Cdk8 = k_unbind_cycC_from_Cdk8*cycC_Cdk8_active_conc;

deactivate_cycC_Cdk8_by_mitogen = k_deactivate_cycC_Cdk8_by_mitogen...

*mitogen_stimulation*cycC_Cdk8_active_conc;

activate_cycC_Cdk8_by_Cdc14 = k_activate_cycC_Cdk8_by_Cdc14...

*Cdc14_phospho_active_conc*cycC_Cdk8_inactive_conc;

u_cycC_unfold = ATP_frac_avail*k_dk_p*cycC_unfold;

u_cycC_cytosol = ATP_frac_avail*k_dk_p*cycC_cytosol;

u_cycC_nuc = ATP_frac_avail*k_dk_p*cycC_nuc;

u_cycC_Cdk8_active = ATP_frac_avail*k_dk_p*cycC_Cdk8_active;

u_cycC_Cdk8_inactive = ATP_frac_avail*k_dk_p*cycC_Cdk8_inactive;

cut_cycC = ATP_frac_avail*k_cut_p*cycC_trash_conc;

% 32 KPC

txs_mRNA_KPC = ATP_frac_avail*k_mRNA_txs*polyII*KPC_loci_frac;

spl_mRNA_KPC = ATP_frac_avail*spl_frac*mRNA_KPC_unspl;

nm_out_mRNA_KPC = ATP_frac_avail*k_nm_mRNA*mRNA_KPC_nuc_conc;

activate_mRNA_KPC = activate_mRNA*mRNA_KPC_inactive/mRNA_inactive;

bind_mRNA_KPC = bind_mRNA*mRNA_KPC_cytosol/mRNA_cytosol;

unbind_mRNA_KPC = unbind_mRNA*mRNA_KPC_in_use/mRNA_in_use;

dk_mRNA_KPC_unspl = k_dk_mRNA*mRNA_KPC_unspl;

dk_mRNA_KPC_nuc = k_dk_mRNA*mRNA_KPC_nuc;

dk_mRNA_KPC_inactive = k_dk_mRNA*mRNA_KPC_inactive;

dk_mRNA_KPC_cytosol = k_dk_mRNA*mRNA_KPC_cytosol;

dk_mRNA_KPC_in_use = k_dk_mRNA*mRNA_KPC_in_use;

cut_mRNA_KPC = k_cut_mRNA*mRNA_KPC_trash;

txl_KPC = unbind_rRNA*(mRNA_KPC_cytosol + mRNA_KPC_in_use)...

/(mRNA_cytosol + mRNA_in_use);

fold_KPC = ATP_frac_avail*k_fold_p*KPC_unfold_conc;

activate_KPC_by_mitogen = k_activate_KPC_by_mitogen...

*mitogen_stimulation*KPC_in_cell_conc;

inactivate_KPC = k_inactivate_KPC*KPC_active_conc;

u_KPC_unfold = ATP_frac_avail*k_dk_p*KPC_unfold;

u_KPC_in_cell = ATP_frac_avail*k_dk_p*KPC_in_cell;

u_KPC_active = ATP_frac_avail*k_dk_p*KPC_active;

cut_KPC = ATP_frac_avail*k_cut_p*KPC_trash_conc;

% Corrections ****************************************

% Corrections for decay of rRNA on mRNA and vice versa

unbind_rRNA = unbind_rRNA + dk4_mRNA*rRNA_in_use_conc/mRNA_in_use_conc;

unbind_mRNA = unbind_mRNA + dk4_rRNA*mRNA_in_use_conc/rRNA_in_use_conc;

% and correct for loss of E2F

unbind_Rb_E2F = unbind_Rb_E2F + u_E2F_Rb_inactive;

% and correct for loss of Rb

unbind_E2F_Rb = unbind_E2F_Rb + u_Rb_on_E2F;

% and correct for loss of p27

unbind_cycD_Cdk4or6_p27 = unbind_cycD_Cdk4or6_p27 + u_p27_on_cycD_Cdk4or6_frac;

% and correct for loss of p27

unbind_cycE_Cdk2_p27 = unbind_cycE_Cdk2_p27 + u_p27_on_cycE_Cdk2_frac;

% and correct for loss of Cdk2 (from cycE)

unbind_cycE_Cdk2 = unbind_cycE_Cdk2 + u_Cdk2_on_cycE_inactive_frac;

% and correct for loss of Cdk2 (from cycA)

unbind_cycA_Cdk2 = unbind_cycA_Cdk2 + u_Cdk2_on_cycA_inactive_frac;

% and correct for loss of p27

unbind_cycA_Cdk2_p27 = unbind_cycA_Cdk2_p27 + u_p27_on_cycA_Cdk2_frac;

% and correct for loss of cycD, cycE, cycA, and Cdk2

unbind_p27_cyclins = unbind_p27_cyclins + u_cycD_Cdk4or6_on_p27_inactive...

+ u_cycE_Cdk2_on_p27_inactive + u_cycA_Cdk2_on_p27_inactive...

+ u_Cdk2_on_cycE_p27 + u_Cdk2_on_cycA_p27;

% and correct for loss of cycE (from Cdk2)

unbind_Cdk2_cycE = unbind_Cdk2_cycE...

+ u_cycE_Cdk2_inactive + u_cycE_Cdk2_active...

+ u_cycE_Cdk2_on_p27_inactive;

% and correct for loss of cycA (from Cdk2)

unbind_Cdk2_cycA = unbind_Cdk2_cycA...

+ u_cycA_Cdk2_inactive + u_cycA_Cdk2_active...

+ u_cycA_Cdk2_on_p27_inactive;

% and correct for loss of SCF

unbind_Skp2_SCF = unbind_Skp2_SCF + u_SCF_on_Skp2;

% and correct for loss of SCF

unbind_Btrc_SCF = unbind_Btrc_SCF + u_SCF_on_Btrc;

% and correct for loss of SCF

unbind_Fbw7_SCF = unbind_Fbw7_SCF + u_SCF_on_Fbw7;

% NOTE: corrections for u_auto_Btrc, u_auto_Fbw7, u_auto_Skp2

% are now in define_levels.in because SCF is now also ubiq

% (they appear here to cancel their presence in u_Btrc_SCF_active, etc.)

% and correct for loss of Btrc

unbind_SCF_Btrc = unbind_SCF_Btrc + u_Btrc_SCF_active - u_auto_Btrc;

% and correct for loss of Fbw7

unbind_SCF_Fbw7 = unbind_SCF_Fbw7 + u_Fbw7_SCF_active - u_auto_Fbw7;

% and correct for loss of Skp2

unbind_SCF_Skp2 = unbind_SCF_Skp2 + u_Skp2_SCF_active - u_auto_Skp2;

% and correct for loss of Cdk1 (from cycA)

unbind_cycA_Cdk1 = unbind_cycA_Cdk1 + u_Cdk1_on_cycA_cytosol_active_frac;

% and correct for loss of Cdk1 (from cycB)

unbind_cycB_Cdk1 = unbind_cycB_Cdk1 + u_Cdk1_on_cycB_cytosol_active_frac;

% and correct for loss of cycA (from Cdk1)

unbind_Cdk1_cycA = unbind_Cdk1_cycA + u_cycA_Cdk1_cytosol_active...

+ u_cycA_Cdk1_phospho_inactive + u_cycA_Cdk1_nuc_active;

% and correct for loss of cycB (from Cdk1)

unbind_Cdk1_cycB = unbind_Cdk1_cycB + u_cycB_Cdk1_cytosol_active...

+ u_cycB_Cdk1_phospho_inactive + u_cycB_Cdk1_nuc_active;

% and correct for loss of Cdh1 (from Emi1)

unbind_Emi1_Cdh1 = unbind_Emi1_Cdh1 + u_Cdh1_Emi1_inactive;

% and correct for loss of Cdc20 (from Emi1)

unbind_Emi1_Cdc20 = unbind_Emi1_Cdc20 + u_Cdc20_Emi1_inactive;

% and correct for loss of Cdh1 (from APC -- see unbind_APC_Cdh1 above for phospho)

unbind_APC_Cdh1 = unbind_APC_Cdh1 + u_Cdh1_APC_active;

% and correct for loss of Cdc20 (from APC)

unbind_APC_Cdc20 = unbind_APC_Cdc20 + u_Cdc20_APC_inactive + u_Cdc20_APC_active;

% and correct for loss of Emi1 (from Cdh1 -- see define_levels.in)

% unbind_Cdh1_Emi1 = unbind_Cdh1_Emi1 + u_Emi1_on_Cdh1 + phospho_Emi1_on_Cdh1;

% and correct for loss of Emi1 (from Cdc20)

unbind_Cdc20_Emi1 = unbind_Cdc20_Emi1 + u_Emi1_on_Cdc20 + phospho_Emi1_on_Cdc20;

% and correct for loss of APC (from Cdh1)

unbind_Cdh1_APC = unbind_Cdh1_APC + u_APC_on_Cdh1;

% and correct for loss of APC (from Cdc20)

unbind_Cdc20_APC = unbind_Cdc20_APC + u_APC_on_Cdc20_inactive_frac;

% !!!!!!!!!!!!!!!!!!!!!!!!!!!!!!!!!!!!!!!!! IS IT DIVIDING?

% [tprev,dividing,k_div] = getdividing...

% (t,bind_Cdh1_to_APC,unbind_Cdh1_APC,...

% u_Cdh1_APC_active,i_NT_in_DNA,NT_in_DNA);

% if(dividing == 1)

% pm_in_NT = 0;

% end

% !!!!!!!!!!!!!!!!!!!!!!!!!!!!!!!!!!!!!!!!!!!! LEVELS EQNS

% base cell

AA_in_cytosol = pm_in_AA + unbind_AA_from_dk_tRNA + unbind_AA - bind_AA - dk_AA...

- k_div*AA_in_cytosol;

AA_in_p = unbind_AA_txl - unbind_AA - k_div*AA_in_p;

AA_on_tRNA = bind_AA - unbind_AA_from_dk_tRNA - unbind_AA_txl - k_div*AA_on_tRNA;

ADP_in_cell = pm_in_ADP + unbind_ADP - bind_ADP - dk1_ADP - k_div*ADP_in_cell;

ADP_trash = dk1_ADP + dk2_ADP - dispose_ADP - k_div*ADP_trash;

ATP_in_cell = bind_ADP - unbind_ADP - dk2_ADP - k_div*ATP_in_cell;

fats_cytosol = pm_in_fats + unbind_fats - make_vesicle - dk1_fats...

- k_div*fats_cytosol;

fats_in_mem = bind_fats - unbind_fats - dk3_fats - k_div*fats_in_mem;

fats_in_vesicles = make_vesicle - bind_fats - dk2_fats - k_div*fats_in_vesicles;

H_between_mito_mems = H_pump - ATP_synthase + H_grow - H_shrink...

- k_div*H_between_mito_mems;

H_in_inner_mito_mems = ATP_synthase - H_pump - k_div*H_in_inner_mito_mems;

junk_spl = cut_spljunk_mRNA + cut_spljunk_rRNA + cut_spljunk_snRNA...

- spljunk - k_div*junk_spl;

junk_spl_mRNA = spljunk_mRNA - cut_spljunk_mRNA - k_div*junk_spl_mRNA;

junk_spl_rRNA = spljunk_rRNA - cut_spljunk_rRNA - k_div*junk_spl_rRNA;

junk_spl_snRNA = spljunk_snRNA - cut_spljunk_snRNA - k_div*junk_spl_snRNA;

mRNA_cytosol = activate_mRNA + unbind_mRNA - bind_mRNA - dk1_mRNA - k_div*mRNA_cytosol;

mRNA_in_use = bind_mRNA - unbind_mRNA - dk4_mRNA - k_div*mRNA_in_use;

mRNA_nuc = spl_mRNA - nm_out_mRNA - dk3_mRNA - k_div*mRNA_nuc;

mRNA_inactive = nm_out_mRNA - activate_mRNA - dk5_mRNA - k_div*mRNA_inactive;

mRNA_trash = dk1_mRNA + dk2_mRNA + dk3_mRNA + dk4_mRNA + dk5_mRNA...

- cut_mRNA - k_div*mRNA_trash;

mRNA_unspl = txs_mRNA - spl_mRNA - dk2_mRNA - k_div*mRNA_unspl;

Na_in_cell = Na_return - Na_pump - k_div*Na_in_cell;

Na_out_cell = Na_pump - Na_return + Na_grow - Na_shrink - k_div*Na_out_cell;

p_in_cell = fold_p + unbind_p - bind_p - u1_p - k_div*p_in_cell;

p_in_use = bind_p - unbind_p - u2_p - k_div*p_in_use;

p_trash = u1_p + u2_p + u3_p - cut_p - k_div*p_trash;

p_unfold = txl_p - fold_p - u3_p - k_div*p_unfold;

rRNA_cytosol = nm_out_rRNA + unbind_rRNA - bind_rRNA - dk1_rRNA - k_div*rRNA_cytosol;

rRNA_in_use = bind_rRNA - unbind_rRNA - dk4_rRNA - k_div*rRNA_in_use;

rRNA_nuc = spl_rRNA - nm_out_rRNA - dk3_rRNA - k_div*rRNA_nuc;

rRNA_trash = dk1_rRNA + dk2_rRNA + dk3_rRNA + dk4_rRNA - cut_rRNA - k_div*rRNA_trash;

rRNA_unspl = txs_rRNA - spl_rRNA - dk2_rRNA - k_div*rRNA_unspl;

snRNA_in_use = bind_snRNA - unbind_snRNA - dk3_snRNA - k_div*snRNA_in_use;

snRNA_nuc = spl_snRNA + unbind_snRNA - bind_snRNA - dk1_snRNA - k_div*snRNA_nuc;

snRNA_trash = dk1_snRNA + dk2_snRNA + dk3_snRNA - cut_snRNA - k_div*snRNA_trash;

snRNA_unspl = txs_snRNA - spl_snRNA - dk2_snRNA - k_div*snRNA_unspl;

sugar_in_cell = pm_in_sugar - pm_out_sugar - k_div*sugar_in_cell;

tRNA_cytosol = nm_out_tRNA + unbind_tRNA - bind_tRNA - dk1_tRNA - k_div*tRNA_cytosol;

tRNA_in_use = bind_tRNA - unbind_tRNA - dk3_tRNA - k_div*tRNA_in_use;

tRNA_nuc = txs_tRNA - nm_out_tRNA - dk2_tRNA - k_div*tRNA_nuc;

tRNA_trash = dk1_tRNA + dk2_tRNA + dk3_tRNA - cut_tRNA - k_div*tRNA_trash;

% RNA polymerase

mRNA_poly_unspl = txs_mRNA_poly - spl_mRNA_poly - dk2_mRNA_poly...

- k_div*mRNA_poly_unspl;

mRNA_poly_nuc = spl_mRNA_poly - nm_out_mRNA_poly - dk3_mRNA_poly...

- k_div*mRNA_poly_nuc;

mRNA_poly_inactive = nm_out_mRNA_poly - activate_mRNA_poly - dk4_mRNA_poly...

- k_div*mRNA_poly_inactive;

mRNA_poly_cytosol = activate_mRNA_poly + unbind_mRNA_poly - bind_mRNA_poly...

- dk1_mRNA_poly - k_div*mRNA_poly_cytosol;

mRNA_poly_in_use = bind_mRNA_poly - unbind_mRNA_poly - dk5_mRNA_poly...

- k_div*mRNA_poly_in_use;

mRNA_poly_trash = dk1_mRNA_poly + dk2_mRNA_poly + dk3_mRNA_poly + dk4_mRNA_poly...

+ dk5_mRNA_poly - cut_mRNA_poly - k_div*mRNA_poly_trash;

p_poly_unfold = txl_p_poly - fold_p_poly - u_p_poly_unfold...

- k_div*p_poly_unfold;

p_poly_cytosol = fold_p_poly - nm_in_p_poly - u_p_poly_cytosol...

- k_div*p_poly_cytosol;

p_poly_nuc_inactive = deactivate_p_poly_by_cycC_Cdk8 - activate_p_poly_by_mitogen...

- u_p_poly_nuc_inactive - k_div*p_poly_nuc_inactive;

p_poly_nuc_active = nm_in_p_poly - bind_p_poly_to_DNA + unbind_p_poly_from_DNA...

- deactivate_p_poly_by_cycC_Cdk8 + activate_p_poly_by_mitogen...

- u_p_poly_nuc_active - k_div*p_poly_nuc_active;

p_poly_in_use = bind_p_poly_to_DNA - unbind_p_poly_from_DNA...

- u_p_poly_in_use - k_div*p_poly_in_use;

p_poly_trash = u_p_poly_unfold + u_p_poly_cytosol + u_p_poly_nuc_active...

+ u_p_poly_nuc_inactive + u_p_poly_in_use - cut_p_poly...

- k_div*p_poly_trash;

% eIF_4

mRNA_eIF_4_unspl = txs_mRNA_eIF_4 - spl_mRNA_eIF_4 - dk2_mRNA_eIF_4...

- k_div*mRNA_eIF_4_unspl;

mRNA_eIF_4_nuc = spl_mRNA_eIF_4 - nm_out_mRNA_eIF_4 - dk3_mRNA_eIF_4...

- k_div*mRNA_eIF_4_nuc;

mRNA_eIF_4_inactive = nm_out_mRNA_eIF_4 - activate_mRNA_eIF_4 - dk5_mRNA_eIF_4...

- k_div*mRNA_eIF_4_inactive;

mRNA_eIF_4_cytosol = activate_mRNA_eIF_4 + unbind_mRNA_eIF_4 - bind_mRNA_eIF_4...

- dk1_mRNA_eIF_4 - k_div*mRNA_eIF_4_cytosol;

mRNA_eIF_4_in_use = bind_mRNA_eIF_4 - unbind_mRNA_eIF_4 - dk5_mRNA_eIF_4...

- k_div*mRNA_eIF_4_in_use;

mRNA_eIF_4_trash = dk1_mRNA_eIF_4 + dk2_mRNA_eIF_4 + dk3_mRNA_eIF_4...

+ dk4_mRNA_eIF_4 + dk5_mRNA_eIF_4 - cut_mRNA_eIF_4...

- k_div*mRNA_eIF_4_trash;

p_eIF_4_unfold = txl_p_eIF_4 - fold_p_eIF_4 - u2_p_eIF_4 - k_div*p_eIF_4_unfold;

p_eIF_4_cytosol = fold_p_eIF_4 - bind_p_eIF_4 + unbind_p_eIF_4 - u1_p_eIF_4...

- k_div*p_eIF_4_cytosol;

p_eIF_4_in_use = bind_p_eIF_4 - unbind_p_eIF_4 - u3_p_eIF_4...

- k_div*p_eIF_4_in_use;

p_eIF_4_trash = u1_p_eIF_4 + u2_p_eIF_4 + u3_p_eIF_4 - cut_p_eIF_4...

- k_div*p_eIF_4_trash;

% 1 p27 STATES

mRNA_p27_unspl = txs_mRNA_p27 - spl_mRNA_p27 - dk_mRNA_p27_unspl...

- k_div*mRNA_p27_unspl;

mRNA_p27_nuc = spl_mRNA_p27 - nm_out_mRNA_p27 - dk_mRNA_p27_nuc...

- k_div*mRNA_p27_nuc;

mRNA_p27_inactive = nm_out_mRNA_p27 - activate_mRNA_p27...

- dk_mRNA_p27_inactive - k_div*mRNA_p27_inactive;

mRNA_p27_cytosol = activate_mRNA_p27 + unbind_mRNA_p27 - bind_mRNA_p27...

- dk_mRNA_p27_cytosol - k_div*mRNA_p27_cytosol;

mRNA_p27_in_use = bind_mRNA_p27 - unbind_mRNA_p27 - dk_mRNA_p27_in_use...

- k_div*mRNA_p27_in_use;

mRNA_p27_trash = dk_mRNA_p27_cytosol + dk_mRNA_p27_unspl + dk_mRNA_p27_nuc...

+ dk_mRNA_p27_inactive + dk_mRNA_p27_in_use...

- cut_mRNA_p27 - k_div*mRNA_p27_trash;

p27_unfold = txl_p27 - fold_p27 - u_p27_unfold - k_div*p27_unfold;

p27_cytosol = fold_p27 - nm_in_p27 - u_p27_cytosol - k_div*p27_cytosol;

p27_nuc = nm_in_p27 + unbind_p27_cyclins - bind_p27_to_cyclins...

- u_p27_nuc - k_div*p27_nuc;

p27_on_cyclins = bind_p27_to_cyclins - unbind_p27_cyclins...

- u_p27_on_cyclins - k_div*p27_on_cyclins;

p27_trash = u_p27_unfold + u_p27_cytosol + u_p27_nuc...

+ u_p27_on_cyclins - cut_p27 - k_div*p27_trash;

% 2 Rb STATES

mRNA_Rb_unspl = txs_mRNA_Rb - spl_mRNA_Rb - dk_mRNA_Rb_unspl - k_div*mRNA_Rb_unspl;

mRNA_Rb_nuc = spl_mRNA_Rb - nm_out_mRNA_Rb - dk_mRNA_Rb_nuc - k_div*mRNA_Rb_nuc;

mRNA_Rb_inactive = nm_out_mRNA_Rb - activate_mRNA_Rb...

- dk_mRNA_Rb_inactive - k_div*mRNA_Rb_inactive;

mRNA_Rb_cytosol = activate_mRNA_Rb + unbind_mRNA_Rb - bind_mRNA_Rb...

- dk_mRNA_Rb_cytosol - k_div*mRNA_Rb_cytosol;

mRNA_Rb_in_use = bind_mRNA_Rb - unbind_mRNA_Rb - dk_mRNA_Rb_in_use...

- k_div*mRNA_Rb_in_use;

mRNA_Rb_trash = dk_mRNA_Rb_cytosol + dk_mRNA_Rb_unspl + dk_mRNA_Rb_nuc...

+ dk_mRNA_Rb_inactive + dk_mRNA_Rb_in_use...

- cut_mRNA_Rb - k_div*mRNA_Rb_trash;

Rb_unfold = txl_Rb - fold_Rb - u_Rb_unfold - k_div*Rb_unfold;

Rb_cytosol = fold_Rb - nm_in_Rb - u_Rb_cytosol - k_div*Rb_cytosol;

Rb_nuc = nm_in_Rb + unbind_Rb_E2F - bind_Rb_to_E2F + unphospho_Rb...

- phospho_Rb_nuc_by_Cdk - u_Rb_nuc - k_div*Rb_nuc;

Rb_on_E2F = bind_Rb_to_E2F - unbind_Rb_E2F - phospho_Rb_on_E2F_by_Cdk...

- u_Rb_on_E2F - k_div*Rb_on_E2F;

Rb_phospho_inactive = phospho_Rb_nuc_by_Cdk + phospho_Rb_on_E2F_by_Cdk - unphospho_Rb...

- u_Rb_phospho_inactive - k_div*Rb_phospho_inactive;

Rb_trash = u_Rb_nuc + u_Rb_unfold + u_Rb_cytosol + u_Rb_on_E2F...

+ u_Rb_phospho_inactive - cut_Rb - k_div*Rb_trash;

% 3 cycD STATES

mRNA_cycD_unspl = txs_mRNA_cycD - spl_mRNA_cycD - dk_mRNA_cycD_unspl...

- k_div*mRNA_cycD_unspl;

mRNA_cycD_nuc = spl_mRNA_cycD - nm_out_mRNA_cycD - dk_mRNA_cycD_nuc...

- k_div*mRNA_cycD_nuc;

mRNA_cycD_inactive = nm_out_mRNA_cycD - activate_mRNA_cycD...

- dk_mRNA_cycD_inactive - k_div*mRNA_cycD_inactive;

mRNA_cycD_cytosol = activate_mRNA_cycD + unbind_mRNA_cycD - bind_mRNA_cycD...

- dk_mRNA_cycD_cytosol - k_div*mRNA_cycD_cytosol;

mRNA_cycD_in_use = bind_mRNA_cycD - unbind_mRNA_cycD...

- dk_mRNA_cycD_in_use - k_div*mRNA_cycD_in_use;

mRNA_cycD_trash = dk_mRNA_cycD_cytosol + dk_mRNA_cycD_unspl + dk_mRNA_cycD_nuc...

+ dk_mRNA_cycD_inactive + dk_mRNA_cycD_in_use...

- cut_mRNA_cycD - k_div*mRNA_cycD_trash;

cycD_unfold = txl_cycD - fold_cycD - u_cycD_unfold - k_div*cycD_unfold;

cycD_cytosol = fold_cycD - nm_in_cycD - u_cycD_cytosol - k_div*cycD_cytosol;

cycD_nuc = nm_in_cycD + unbind_cycD_Cdk4or6 - bind_cycD_to_Cdk4or6...

- u_cycD_nuc - k_div*cycD_nuc;

cycD_Cdk4or6_active = bind_cycD_to_Cdk4or6 - unbind_cycD_Cdk4or6...

- bind_cycD_Cdk4or6_to_p27 + unbind_cycD_Cdk4or6_p27...

- u_cycD_Cdk4or6_active - k_div*cycD_Cdk4or6_active;

cycD_Cdk4or6_on_p27_inactive = bind_cycD_Cdk4or6_to_p27 - unbind_cycD_Cdk4or6_p27...

- u_cycD_Cdk4or6_on_p27_inactive...

- k_div*cycD_Cdk4or6_on_p27_inactive;

cycD_trash = u_cycD_unfold + u_cycD_cytosol + u_cycD_nuc...

+ u_cycD_Cdk4or6_active + u_cycD_Cdk4or6_on_p27_inactive...

- cut_cycD - k_div*cycD_trash;

% 4 Cdk2 STATES

mRNA_Cdk2_unspl = txs_mRNA_Cdk2 - spl_mRNA_Cdk2 - dk_mRNA_Cdk2_unspl...

- k_div*mRNA_Cdk2_unspl;

mRNA_Cdk2_nuc = spl_mRNA_Cdk2 - nm_out_mRNA_Cdk2 - dk_mRNA_Cdk2_nuc...

- k_div*mRNA_Cdk2_nuc;

mRNA_Cdk2_inactive = nm_out_mRNA_Cdk2 - activate_mRNA_Cdk2...

- dk_mRNA_Cdk2_inactive - k_div*mRNA_Cdk2_inactive;

mRNA_Cdk2_cytosol = activate_mRNA_Cdk2 + unbind_mRNA_Cdk2 - bind_mRNA_Cdk2...

- dk_mRNA_Cdk2_cytosol - k_div*mRNA_Cdk2_cytosol;

mRNA_Cdk2_in_use = bind_mRNA_Cdk2 - unbind_mRNA_Cdk2 - dk_mRNA_Cdk2_in_use...

- k_div*mRNA_Cdk2_in_use;

mRNA_Cdk2_trash = dk_mRNA_Cdk2_cytosol + dk_mRNA_Cdk2_unspl + dk_mRNA_Cdk2_nuc...

+ dk_mRNA_Cdk2_inactive + dk_mRNA_Cdk2_in_use...

- cut_mRNA_Cdk2 - k_div*mRNA_Cdk2_trash;

Cdk2_unfold = txl_Cdk2 - fold_Cdk2 - u_Cdk2_unfold - k_div*Cdk2_unfold;

Cdk2_cytosol = fold_Cdk2 - nm_in_Cdk2 - u_Cdk2_cytosol - k_div*Cdk2_cytosol;

Cdk2_nuc = nm_in_Cdk2 + unbind_Cdk2_cycE - bind_Cdk2_to_cycE...

+ unbind_Cdk2_cycA - bind_Cdk2_to_cycA...

- u_Cdk2_nuc - k_div*Cdk2_nuc;

Cdk2_on_cycE = bind_Cdk2_to_cycE - unbind_Cdk2_cycE...

- u_Cdk2_on_cycE - k_div*Cdk2_on_cycE;

Cdk2_on_cycA = bind_Cdk2_to_cycA - unbind_Cdk2_cycA...

- u_Cdk2_on_cycA - k_div*Cdk2_on_cycA;

Cdk2_trash = u_Cdk2_unfold + u_Cdk2_cytosol + u_Cdk2_on_cycE + u_Cdk2_nuc...

+ u_Cdk2_on_cycA - cut_Cdk2 - k_div*Cdk2_trash;

% 5 cycE STATES

mRNA_cycE_unspl = txs_mRNA_cycE - spl_mRNA_cycE - dk_mRNA_cycE_unspl...

- k_div*mRNA_cycE_unspl;

mRNA_cycE_nuc = spl_mRNA_cycE - nm_out_mRNA_cycE - dk_mRNA_cycE_nuc...

- k_div*mRNA_cycE_nuc;

mRNA_cycE_inactive = nm_out_mRNA_cycE - activate_mRNA_cycE...

- dk_mRNA_cycE_inactive - k_div*mRNA_cycE_inactive;

mRNA_cycE_cytosol = activate_mRNA_cycE + unbind_mRNA_cycE - bind_mRNA_cycE...

- dk_mRNA_cycE_cytosol - k_div*mRNA_cycE_cytosol;

mRNA_cycE_in_use = bind_mRNA_cycE - unbind_mRNA_cycE - dk_mRNA_cycE_in_use...

- k_div*mRNA_cycE_in_use;

mRNA_cycE_trash = dk_mRNA_cycE_cytosol + dk_mRNA_cycE_unspl + dk_mRNA_cycE_nuc...

+ dk_mRNA_cycE_inactive + dk_mRNA_cycE_in_use...

- cut_mRNA_cycE - k_div*mRNA_cycE_trash;

cycE_unfold = txl_cycE - fold_cycE - u_cycE_unfold - k_div*cycE_unfold;

cycE_cytosol = fold_cycE - nm_in_cycE - u_cycE_cytosol - k_div*cycE_cytosol;

% and correct for loss of Cdk2

cycE_nuc = nm_in_cycE + unbind_cycE_Cdk2 - bind_cycE_to_Cdk2...

- u_cycE_nuc - k_div*cycE_nuc...

+ u_Cdk2_on_cycE_active_frac...

+ u_Cdk2_on_cycE_on_p27_inactive_frac;

cycE_Cdk2_inactive = bind_cycE_to_Cdk2 - unbind_cycE_Cdk2...

- bind_cycE_Cdk2_to_p27 + unbind_cycE_Cdk2_p27...

- dephospho_cycE_Cdk2...

- dephospho_cycE_Cdk2_by_Cdc25A + phospho_cycE_Cdk2...

- u_cycE_Cdk2_inactive - k_div*cycE_Cdk2_inactive;

% cycE/Cdk2 is activated when dephosphorylated

% and correct for loss of Cdk2

cycE_Cdk2_active = dephospho_cycE_Cdk2...

+ dephospho_cycE_Cdk2_by_Cdc25A - phospho_cycE_Cdk2...

- u_cycE_Cdk2_active - k_div*cycE_Cdk2_active...

- u_Cdk2_on_cycE_active_frac;

% cycE/Cdk2 is inhibited by p27

% and correct for loss of Cdk2

cycE_Cdk2_on_p27_inactive = bind_cycE_Cdk2_to_p27 - unbind_cycE_Cdk2_p27...

- u_cycE_Cdk2_on_p27_inactive...

- k_div*cycE_Cdk2_on_p27_inactive...

- u_Cdk2_on_cycE_on_p27_inactive_frac;

cycE_trash = u_cycE_unfold + u_cycE_cytosol + u_cycE_nuc...

+ u_cycE_Cdk2_inactive + u_cycE_Cdk2_active...

+ u_cycE_Cdk2_on_p27_inactive - cut_cycE - k_div*cycE_trash;

% 6 B-Myb STATES

mRNA_B_Myb_unspl = txs_mRNA_B_Myb - spl_mRNA_B_Myb...

- dk_mRNA_B_Myb_unspl - k_div*mRNA_B_Myb_unspl;

mRNA_B_Myb_nuc = spl_mRNA_B_Myb - nm_out_mRNA_B_Myb...

- dk_mRNA_B_Myb_nuc - k_div*mRNA_B_Myb_nuc;

mRNA_B_Myb_inactive = nm_out_mRNA_B_Myb - activate_mRNA_B_Myb...

- dk_mRNA_B_Myb_inactive - k_div*mRNA_B_Myb_inactive;

mRNA_B_Myb_cytosol = activate_mRNA_B_Myb + unbind_mRNA_B_Myb - bind_mRNA_B_Myb...

- dk_mRNA_B_Myb_cytosol - k_div*mRNA_B_Myb_cytosol;

mRNA_B_Myb_in_use = bind_mRNA_B_Myb - unbind_mRNA_B_Myb...

- dk_mRNA_B_Myb_in_use - k_div*mRNA_B_Myb_in_use;

mRNA_B_Myb_trash = dk_mRNA_B_Myb_cytosol + dk_mRNA_B_Myb_unspl...

+ dk_mRNA_B_Myb_nuc + dk_mRNA_B_Myb_inactive...

+ dk_mRNA_B_Myb_in_use...

- cut_mRNA_B_Myb - k_div*mRNA_B_Myb_trash;

B_Myb_unfold = txl_B_Myb - fold_B_Myb - u_B_Myb_unfold - k_div*B_Myb_unfold;

B_Myb_cytosol = fold_B_Myb - nm_in_B_Myb - u_B_Myb_cytosol - k_div*B_Myb_cytosol;

B_Myb_nuc = nm_in_B_Myb + unphospho_B_Myb - phospho_B_Myb_by_cyc_Cdk...

- u_B_Myb_nuc - k_div*B_Myb_nuc;

B_Myb_phospho_active = phospho_B_Myb_by_cyc_Cdk - unphospho_B_Myb...

- bind_B_Myb_to_DNA + unbind_B_Myb_from_DNA...

- u_B_Myb_phospho_active - k_div*B_Myb_phospho_active;

B_Myb_on_DNA = bind_B_Myb_to_DNA - unbind_B_Myb_from_DNA - u_B_Myb_on_DNA...

- k_div*B_Myb_on_DNA;

B_Myb_trash = u_B_Myb_unfold + u_B_Myb_cytosol + u_B_Myb_nuc...

+ u_B_Myb_phospho_active...

+ u_B_Myb_on_DNA - cut_B_Myb - k_div*B_Myb_trash;

% 7 NF-Y STATES

mRNA_NF_Y_unspl = txs_mRNA_NF_Y - spl_mRNA_NF_Y - dk_mRNA_NF_Y_unspl...

- k_div*mRNA_NF_Y_unspl;

mRNA_NF_Y_nuc = spl_mRNA_NF_Y - nm_out_mRNA_NF_Y - dk_mRNA_NF_Y_nuc...

- k_div*mRNA_NF_Y_nuc;

mRNA_NF_Y_inactive = nm_out_mRNA_NF_Y - activate_mRNA_NF_Y...

- dk_mRNA_NF_Y_inactive - k_div*mRNA_NF_Y_inactive;

mRNA_NF_Y_cytosol = activate_mRNA_NF_Y + unbind_mRNA_NF_Y - bind_mRNA_NF_Y...

- dk_mRNA_NF_Y_cytosol - k_div*mRNA_NF_Y_cytosol;

mRNA_NF_Y_in_use = bind_mRNA_NF_Y - unbind_mRNA_NF_Y - dk_mRNA_NF_Y_in_use...

- k_div*mRNA_NF_Y_in_use;

mRNA_NF_Y_trash = dk_mRNA_NF_Y_cytosol + dk_mRNA_NF_Y_unspl + dk_mRNA_NF_Y_nuc...

+ dk_mRNA_NF_Y_inactive + dk_mRNA_NF_Y_in_use...

- cut_mRNA_NF_Y - k_div*mRNA_NF_Y_trash;

NF_Y_unfold = txl_NF_Y - fold_NF_Y - u_NF_Y_unfold - k_div*NF_Y_unfold;

NF_Y_cytosol = fold_NF_Y - nm_in_NF_Y - u_NF_Y_cytosol - k_div*NF_Y_cytosol;

% NF_Y_nuc = nm_in_NF_Y + unphospho_NF_Y - phospho_NF_Y_by_cyc_Cdk...

% - u_NF_Y_nuc - k_div*NF_Y_nuc;

% NF_Y_phospho_active = phospho_NF_Y_by_cyc_Cdk - unphospho_NF_Y - bind_NF_Y_to_DNA...

% + unbind_NF_Y_from_DNA - u_NF_Y_phospho_active...

% - k_div*NF_Y_phospho_active;

NF_Y_nuc = nm_in_NF_Y + unphospho_NF_Y + dephospho_NF_Y_by_Cdc14...

- phospho_NF_Y_by_cyc_Cdk...

- u_NF_Y_nuc - k_div*NF_Y_nuc;

NF_Y_phospho_active = phospho_NF_Y_by_cyc_Cdk - unphospho_NF_Y... \

- dephospho_NF_Y_by_Cdc14 - bind_NF_Y_to_DNA...

+ unbind_NF_Y_from_DNA - u_NF_Y_phospho_active...

- k_div*NF_Y_phospho_active;

NF_Y_on_DNA = bind_NF_Y_to_DNA - unbind_NF_Y_from_DNA - u_NF_Y_on_DNA...

- k_div*NF_Y_on_DNA;

NF_Y_trash = u_NF_Y_unfold + u_NF_Y_cytosol + u_NF_Y_nuc...

+ u_NF_Y_phospho_active...

+ u_NF_Y_on_DNA - cut_NF_Y - k_div*NF_Y_trash;

% 8 E2F STATES

mRNA_E2F_unspl = txs_mRNA_E2F - spl_mRNA_E2F - dk_mRNA_E2F_unspl...

- k_div*mRNA_E2F_unspl;

mRNA_E2F_nuc = spl_mRNA_E2F - nm_out_mRNA_E2F - dk_mRNA_E2F_nuc...

- k_div*mRNA_E2F_nuc;

mRNA_E2F_inactive = nm_out_mRNA_E2F - activate_mRNA_E2F...

- dk_mRNA_E2F_inactive - k_div*mRNA_E2F_inactive;

mRNA_E2F_cytosol = activate_mRNA_E2F + unbind_mRNA_E2F - bind_mRNA_E2F...

- dk_mRNA_E2F_cytosol - k_div*mRNA_E2F_cytosol;

mRNA_E2F_in_use = bind_mRNA_E2F - unbind_mRNA_E2F - dk_mRNA_E2F_in_use...

- k_div*mRNA_E2F_in_use;

mRNA_E2F_trash = dk_mRNA_E2F_cytosol + dk_mRNA_E2F_unspl + dk_mRNA_E2F_nuc...

+ dk_mRNA_E2F_inactive + dk_mRNA_E2F_in_use...

- cut_mRNA_E2F - k_div*mRNA_E2F_trash;

E2F_unfold = txl_E2F - fold_E2F - u_E2F_unfold - k_div*E2F_unfold;

E2F_cytosol = fold_E2F - nm_in_E2F - u_E2F_cytosol - k_div*E2F_cytosol;

E2F_nuc = nm_in_E2F + unbind_E2F_Rb + unbind_E2F_DNA - bind_E2F_to_Rb...

- bind_E2F_to_DNA - u_E2F_nuc - k_div*E2F_nuc;

E2F_Rb_inactive = bind_E2F_to_Rb - unbind_E2F_Rb - u_E2F_Rb_inactive...

- k_div*E2F_Rb_inactive;

E2F_on_DNA = bind_E2F_to_DNA - unbind_E2F_DNA - u_E2F_on_DNA - k_div*E2F_on_DNA;

E2F_trash = u_E2F_unfold + u_E2F_cytosol + u_E2F_nuc + u_E2F_Rb_inactive...

+ u_E2F_on_DNA - cut_E2F - k_div*E2F_trash;

% 9 cycA STATES

mRNA_cycA_unspl = txs_mRNA_cycA - spl_mRNA_cycA - dk_mRNA_cycA_unspl...

- k_div*mRNA_cycA_unspl;

mRNA_cycA_nuc = spl_mRNA_cycA - nm_out_mRNA_cycA - dk_mRNA_cycA_nuc...

- k_div*mRNA_cycA_nuc;

mRNA_cycA_inactive = nm_out_mRNA_cycA - activate_mRNA_cycA...

- dk_mRNA_cycA_inactive - k_div*mRNA_cycA_inactive;

mRNA_cycA_cytosol = activate_mRNA_cycA + unbind_mRNA_cycA - bind_mRNA_cycA...

- dk_mRNA_cycA_cytosol - k_div*mRNA_cycA_cytosol;

mRNA_cycA_in_use = bind_mRNA_cycA - unbind_mRNA_cycA - dk_mRNA_cycA_in_use...

- k_div*mRNA_cycA_in_use;

mRNA_cycA_trash = dk_mRNA_cycA_cytosol + dk_mRNA_cycA_unspl + dk_mRNA_cycA_nuc...

+ dk_mRNA_cycA_inactive + dk_mRNA_cycA_in_use...

- cut_mRNA_cycA - k_div*mRNA_cycA_trash;

cycA_unfold = txl_cycA - fold_cycA - u_cycA_unfold - k_div*cycA_unfold;

% and correct for loss of Cdk1

cycA_cytosol = fold_cycA - nm_in_cycA...

- bind_cycA_to_Cdk1 + unbind_cycA_Cdk1...

- u_cycA_cytosol - k_div*cycA_cytosol...

+ u_Cdk1_on_cycA_phospho_inactive_frac;

% cycA/Cdk1

cycA_Cdk1_cytosol_active = bind_cycA_to_Cdk1 - unbind_cycA_Cdk1...

- phospho_cycA_Cdk1_by_Wee1...

+ dephospho_cycA_Cdk1_by_Cdc25...

+ unphospho_cycA_Cdk1_from_Wee1...

- nm_in_cycA_Cdk1 + nm_out_cycA_Cdk1...

- u_cycA_Cdk1_cytosol_active...

- k_div*cycA_Cdk1_cytosol_active;

% and correct for loss of Cdk1

cycA_Cdk1_phospho_inactive = phospho_cycA_Cdk1_by_Wee1...

- dephospho_cycA_Cdk1_by_Cdc25...

- unphospho_cycA_Cdk1_from_Wee1...

- u_cycA_Cdk1_phospho_inactive...

- k_div*cycA_Cdk1_phospho_inactive...

- u_Cdk1_on_cycA_phospho_inactive_frac;

% and correct for loss of Cdk1

cycA_Cdk1_nuc_active = nm_in_cycA_Cdk1 - nm_out_cycA_Cdk1...

- u_cycA_Cdk1_nuc_active - k_div*cycA_Cdk1_nuc_active...

- u_Cdk1_on_cycA_nuc_active_frac;

% and correct for loss of Cdk2 and Cdk1

cycA_nuc = nm_in_cycA + unbind_cycA_Cdk2 - bind_cycA_to_Cdk2...

+ unbind_cycA_Cdk1 - bind_cycA_to_Cdk1...

- u_cycA_nuc - k_div*cycA_nuc...

+ u_Cdk2_on_cycA_active_frac...

+ u_Cdk2_on_cycA_on_p27_inactive_frac...

+ u_Cdk1_on_cycA_nuc_active_frac;

% cycA/Cdk2

cycA_Cdk2_inactive = bind_cycA_to_Cdk2 - unbind_cycA_Cdk2...

- bind_cycA_Cdk2_to_p27 + unbind_cycA_Cdk2_p27...

- dephospho_cycA_Cdk2_by_Cdc25A + phospho_cycA_Cdk2...

- u_cycA_Cdk2_inactive - k_div*cycA_Cdk2_inactive;

% and correct for loss of Cdk2

cycA_Cdk2_active = dephospho_cycA_Cdk2_by_Cdc25A - phospho_cycA_Cdk2...

- u_cycA_Cdk2_active - k_div*cycA_Cdk2_active...

- u_Cdk2_on_cycA_active_frac;

% and correct for loss of Cdk2

cycA_Cdk2_on_p27_inactive = bind_cycA_Cdk2_to_p27 - unbind_cycA_Cdk2_p27...

- u_cycA_Cdk2_on_p27_inactive...

- k_div*cycA_Cdk2_on_p27_inactive...

- u_Cdk2_on_cycA_on_p27_inactive_frac;

cycA_trash = u_cycA_unfold + u_cycA_cytosol + u_cycA_nuc...

+ u_cycA_Cdk2_inactive + u_cycA_Cdk2_active...

+ u_cycA_Cdk2_on_p27_inactive...

+ u_cycA_Cdk1_cytosol_active + u_cycA_Cdk1_phospho_inactive...

+ u_cycA_Cdk1_nuc_active...

- cut_cycA - k_div*cycA_trash;

% 10 SCF STATES

mRNA_SCF_unspl = txs_mRNA_SCF - spl_mRNA_SCF - dk_mRNA_SCF_unspl...

- k_div*mRNA_SCF_unspl;

mRNA_SCF_nuc = spl_mRNA_SCF - nm_out_mRNA_SCF - dk_mRNA_SCF_nuc...

- k_div*mRNA_SCF_nuc;

mRNA_SCF_inactive = nm_out_mRNA_SCF - activate_mRNA_SCF - dk_mRNA_SCF_inactive...

- k_div*mRNA_SCF_inactive;

mRNA_SCF_cytosol = activate_mRNA_SCF + unbind_mRNA_SCF - bind_mRNA_SCF...

- dk_mRNA_SCF_cytosol - k_div*mRNA_SCF_cytosol;

mRNA_SCF_in_use = bind_mRNA_SCF - unbind_mRNA_SCF - dk_mRNA_SCF_in_use...

- k_div*mRNA_SCF_in_use;

mRNA_SCF_trash = dk_mRNA_SCF_cytosol + dk_mRNA_SCF_unspl + dk_mRNA_SCF_nuc...

+ dk_mRNA_SCF_inactive + dk_mRNA_SCF_in_use...

- cut_mRNA_SCF - k_div*mRNA_SCF_trash;

SCF_unfold = txl_SCF - fold_SCF - u_SCF_unfold - k_div*SCF_unfold;

% There are 2 ways to handle the autoubiq of Skp2, Fbw7, and Btrc:

% (1) leave the remaining SCF alone, or

% (2) also ubiq the SCF

% (oops, another possible way is that one or two of the substrates ubiqs SCF and the others don't)

% Next is the leave SCF alone

SCF_in_cell = fold_SCF...

+ unbind_SCF_Btrc - bind_SCF_to_Btrc...

+ unbind_SCF_Fbw7 - bind_SCF_to_Fbw7...

+ unbind_SCF_Skp2 - bind_SCF_to_Skp2...

- u_SCF_in_cell - k_div*SCF_in_cell...

+ u_auto_Fbw7...

+ u_auto_Skp2...

+ u_auto_Btrc;

% Next is the ubiq SCF

% SCF_in_cell = fold_SCF...

% + unbind_SCF_Btrc - bind_SCF_to_Btrc...

% + unbind_SCF_Fbw7 - bind_SCF_to_Fbw7...

% + unbind_SCF_Skp2 - bind_SCF_to_Skp2...

% - u_SCF_in_cell - k_div*SCF_in_cell;

% Correct for co-auto-ubiq of Fbw7

SCF_on_Fbw7 = bind_SCF_to_Fbw7 - unbind_SCF_Fbw7...

- u_SCF_on_Fbw7 - k_div*SCF_on_Fbw7...

- u_auto_Fbw7;

% Correct for co-auto-ubiq Skp2

SCF_on_Skp2 = bind_SCF_to_Skp2 - unbind_SCF_Skp2...

- u_SCF_on_Skp2 - k_div*SCF_on_Skp2...

- u_auto_Skp2;

% Correct for co-auto-ubiq Btrc

SCF_on_Btrc = bind_SCF_to_Btrc - unbind_SCF_Btrc...

- u_SCF_on_Btrc - k_div*SCF_on_Btrc...

- u_auto_Btrc;

% Correct for co-auto-ubiq of Fbw7, Skp2, and Btrc

SCF_trash = u_SCF_unfold + u_SCF_in_cell...

+ u_SCF_on_Skp2 + u_SCF_on_Fbw7 + u_SCF_on_Btrc...

- cut_SCF - k_div*SCF_trash...

+ u_auto_Fbw7 + u_auto_Skp2 + u_auto_Btrc;

% 11 Skp2 STATES

mRNA_Skp2_unspl = txs_mRNA_Skp2 - spl_mRNA_Skp2 - dk_mRNA_Skp2_unspl...

- k_div*mRNA_Skp2_unspl;

mRNA_Skp2_nuc = spl_mRNA_Skp2 - nm_out_mRNA_Skp2 - dk_mRNA_Skp2_nuc...

- k_div*mRNA_Skp2_nuc;

mRNA_Skp2_inactive = nm_out_mRNA_Skp2 - activate_mRNA_Skp2...

- dk_mRNA_Skp2_inactive - k_div*mRNA_Skp2_inactive;

mRNA_Skp2_cytosol = activate_mRNA_Skp2 + unbind_mRNA_Skp2 - bind_mRNA_Skp2...

- dk_mRNA_Skp2_cytosol - k_div*mRNA_Skp2_cytosol;

mRNA_Skp2_in_use = bind_mRNA_Skp2 - unbind_mRNA_Skp2 - dk_mRNA_Skp2_in_use...

- k_div*mRNA_Skp2_in_use;

mRNA_Skp2_trash = dk_mRNA_Skp2_cytosol + dk_mRNA_Skp2_unspl + dk_mRNA_Skp2_nuc...

+ dk_mRNA_Skp2_inactive + dk_mRNA_Skp2_in_use...

- cut_mRNA_Skp2 - k_div*mRNA_Skp2_trash;

Skp2_unfold = txl_Skp2 - fold_Skp2 - u_Skp2_unfold - k_div*Skp2_unfold;

Skp2_cytosol = fold_Skp2 - nm_in_Skp2 - u_Skp2_cytosol - k_div*Skp2_cytosol;

Skp2_nuc = nm_in_Skp2 + unbind_Skp2_SCF - bind_Skp2_to_SCF...

- u_Skp2_nuc - k_div*Skp2_nuc;

Skp2_SCF_active = bind_Skp2_to_SCF - unbind_Skp2_SCF - u_Skp2_SCF_active...

- k_div*Skp2_SCF_active;

Skp2_trash = u_Skp2_nuc + u_Skp2_unfold + u_Skp2_cytosol + u_Skp2_SCF_active...

- cut_Skp2 - k_div*Skp2_trash;

% 12 Btrc STATES

mRNA_Btrc_unspl = txs_mRNA_Btrc - spl_mRNA_Btrc - dk_mRNA_Btrc_unspl...

- k_div*mRNA_Btrc_unspl;

mRNA_Btrc_nuc = spl_mRNA_Btrc - nm_out_mRNA_Btrc - dk_mRNA_Btrc_nuc...

- k_div*mRNA_Btrc_nuc;

mRNA_Btrc_inactive = nm_out_mRNA_Btrc - activate_mRNA_Btrc...

- dk_mRNA_Btrc_inactive - k_div*mRNA_Btrc_inactive;

mRNA_Btrc_cytosol = activate_mRNA_Btrc + unbind_mRNA_Btrc - bind_mRNA_Btrc...

- dk_mRNA_Btrc_cytosol - k_div*mRNA_Btrc_cytosol;

mRNA_Btrc_in_use = bind_mRNA_Btrc - unbind_mRNA_Btrc - dk_mRNA_Btrc_in_use...

- k_div*mRNA_Btrc_in_use;

mRNA_Btrc_trash = dk_mRNA_Btrc_cytosol + dk_mRNA_Btrc_unspl + dk_mRNA_Btrc_nuc...

+ dk_mRNA_Btrc_inactive + dk_mRNA_Btrc_in_use...

- cut_mRNA_Btrc - k_div*mRNA_Btrc_trash;

Btrc_unfold = txl_Btrc - fold_Btrc - u_Btrc_unfold - k_div*Btrc_unfold;

Btrc_cytosol = fold_Btrc + unbind_Btrc_SCF - bind_Btrc_to_SCF...

- u_Btrc_cytosol - k_div*Btrc_cytosol;

Btrc_SCF_active = bind_Btrc_to_SCF - unbind_Btrc_SCF - u_Btrc_SCF_active...

- k_div*Btrc_SCF_active;

Btrc_trash = u_Btrc_unfold + u_Btrc_cytosol + u_Btrc_SCF_active - cut_Btrc...

- k_div*Btrc_trash;

% 13 Fbw7 STATES

mRNA_Fbw7_unspl = txs_mRNA_Fbw7 - spl_mRNA_Fbw7 - dk_mRNA_Fbw7_unspl...

- k_div*mRNA_Fbw7_unspl;

mRNA_Fbw7_nuc = spl_mRNA_Fbw7 - nm_out_mRNA_Fbw7 - dk_mRNA_Fbw7_nuc...

- k_div*mRNA_Fbw7_nuc;

mRNA_Fbw7_inactive = nm_out_mRNA_Fbw7 - activate_mRNA_Fbw7...

- dk_mRNA_Fbw7_inactive - k_div*mRNA_Fbw7_inactive;

mRNA_Fbw7_cytosol = activate_mRNA_Fbw7 + unbind_mRNA_Fbw7 - bind_mRNA_Fbw7...

- dk_mRNA_Fbw7_cytosol - k_div*mRNA_Fbw7_cytosol;

mRNA_Fbw7_in_use = bind_mRNA_Fbw7 - unbind_mRNA_Fbw7 - dk_mRNA_Fbw7_in_use...

- k_div*mRNA_Fbw7_in_use;

mRNA_Fbw7_trash = dk_mRNA_Fbw7_cytosol + dk_mRNA_Fbw7_unspl + dk_mRNA_Fbw7_nuc...

+ dk_mRNA_Fbw7_inactive + dk_mRNA_Fbw7_in_use...

- cut_mRNA_Fbw7 - k_div*mRNA_Fbw7_trash;

Fbw7_unfold = txl_Fbw7 - fold_Fbw7 - u_Fbw7_unfold - k_div*Fbw7_unfold;

Fbw7_cytosol = fold_Fbw7 - nm_in_Fbw7 - u_Fbw7_cytosol - k_div*Fbw7_cytosol;

Fbw7_nuc = nm_in_Fbw7 + unbind_Fbw7_SCF - bind_Fbw7_to_SCF...

- u_Fbw7_nuc - k_div*Fbw7_nuc;

Fbw7_SCF_active = bind_Fbw7_to_SCF - unbind_Fbw7_SCF - u_Fbw7_SCF_active...

- k_div*Fbw7_SCF_active;

Fbw7_trash = u_Fbw7_nuc + u_Fbw7_unfold + u_Fbw7_cytosol + u_Fbw7_SCF_active...

- cut_Fbw7 - k_div*Fbw7_trash;

% 14 TF_grow STATES

mRNA_TF_grow_unspl = txs_mRNA_TF_grow - spl_mRNA_TF_grow - dk_mRNA_TF_grow_unspl...

- k_div*mRNA_TF_grow_unspl;

mRNA_TF_grow_cytosol = activate_mRNA_TF_grow + unbind_mRNA_TF_grow - bind_mRNA_TF_grow...

- dk_mRNA_TF_grow_cytosol - k_div*mRNA_TF_grow_cytosol;

mRNA_TF_grow_inactive = nm_out_mRNA_TF_grow - activate_mRNA_TF_grow...

- dk_mRNA_TF_grow_inactive - k_div*mRNA_TF_grow_inactive;

mRNA_TF_grow_nuc = spl_mRNA_TF_grow - nm_out_mRNA_TF_grow - dk_mRNA_TF_grow_nuc...

- k_div*mRNA_TF_grow_nuc;

mRNA_TF_grow_in_use = bind_mRNA_TF_grow - unbind_mRNA_TF_grow - dk_mRNA_TF_grow_in_use...

- k_div*mRNA_TF_grow_in_use;

mRNA_TF_grow_trash = dk_mRNA_TF_grow_cytosol + dk_mRNA_TF_grow_unspl...

+ dk_mRNA_TF_grow_nuc...

+ dk_mRNA_TF_grow_inactive + dk_mRNA_TF_grow_in_use...

- cut_mRNA_TF_grow - k_div*mRNA_TF_grow_trash;

TF_grow_unfold = txl_TF_grow - fold_TF_grow - u_TF_grow_unfold - k_div*TF_grow_unfold;

TF_grow_cytosol = fold_TF_grow - nm_in_TF_grow - u_TF_grow_cytosol...

- k_div*TF_grow_cytosol;

TF_grow_nuc = nm_in_TF_grow + unbind_TF_grow_DNA - bind_TF_grow_to_DNA...

- u_TF_grow_nuc - k_div*TF_grow_nuc;

TF_grow_on_DNA = bind_TF_grow_to_DNA - unbind_TF_grow_DNA - u_TF_grow_on_DNA...

- k_div*TF_grow_on_DNA;

TF_grow_trash = u_TF_grow_unfold + u_TF_grow_cytosol + u_TF_grow_nuc...

+ u_TF_grow_on_DNA - cut_TF_grow - k_div*TF_grow_trash;

% 15 NT STATES

NT_in_cell = pm_in_NT - bind_NT_in_RNA - bind_NT_in_DNA + unbind_NT_RNA...

+ unbind_NT_DNA - dk_NT - k_div*NT_in_cell;

NT_in_RNA = bind_NT_in_RNA - unbind_NT_RNA - k_div*NT_in_RNA;

NT_in_DNA = bind_NT_in_DNA - unbind_NT_DNA - k_div_DNA_factor*k_div*NT_in_DNA;

% 16 RC STATES

mRNA_RC_unspl = txs_mRNA_RC - spl_mRNA_RC - dk_mRNA_RC_unspl - k_div*mRNA_RC_unspl;

mRNA_RC_nuc = spl_mRNA_RC - nm_out_mRNA_RC - dk_mRNA_RC_nuc - k_div*mRNA_RC_nuc;

mRNA_RC_inactive = nm_out_mRNA_RC - activate_mRNA_RC...

- dk_mRNA_RC_inactive - k_div*mRNA_RC_inactive;

mRNA_RC_cytosol = activate_mRNA_RC + unbind_mRNA_RC - bind_mRNA_RC...

- dk_mRNA_RC_cytosol - k_div*mRNA_RC_cytosol;

mRNA_RC_in_use = bind_mRNA_RC - unbind_mRNA_RC - dk_mRNA_RC_in_use...

- k_div*mRNA_RC_in_use;

mRNA_RC_trash = dk_mRNA_RC_cytosol + dk_mRNA_RC_unspl + dk_mRNA_RC_nuc...

+ dk_mRNA_RC_inactive + dk_mRNA_RC_in_use...

- cut_mRNA_RC - k_div*mRNA_RC_trash;

RC_unfold = txl_RC - fold_RC - u_RC_unfold - k_div*RC_unfold;

RC_cytosol = fold_RC - nm_in_RC - u_RC_cytosol - k_div*RC_cytosol;

RC_nuc = nm_in_RC - bind_RC_to_DNA + unbind_RC_DNA...

+ delicense_RC...

- u_RC_nuc - k_div*RC_nuc;

RC_on_DNA = bind_RC_to_DNA - unbind_RC_DNA...

- phospho_RC_by_cycD_Cdk4or6...

- phospho_RC_by_cycE_Cdk2 - phospho_RC_by_cycA_Cdk2...

- u_RC_on_DNA - k_div*RC_on_DNA;

RC_licensed = phospho_RC_by_cycD_Cdk4or6...

+ phospho_RC_by_cycE_Cdk2 + phospho_RC_by_cycA_Cdk2...

- attach_RC_DNA_to_DNA_poly - u_RC_licensed - k_div*RC_licensed;

RC_traversed_by_DNA_poly = attach_RC_DNA_to_DNA_poly - delicense_RC...

- u_RC_traversed_by_DNA_poly...

- k_div*RC_traversed_by_DNA_poly;

RC_trash = u_RC_unfold + u_RC_cytosol + u_RC_nuc...

+ u_RC_on_DNA + u_RC_licensed + u_RC_traversed_by_DNA_poly...

- cut_RC - k_div*RC_trash;

% keep track of the number of RC attached to DNA (must not exceed x_RC = 3E9 bp / 2E5 bp = 1.5E4 RC)

add_to_RC_count = bind_RC_to_DNA;

% zero the count at cell division.

if(dividing)

clear_RC_count = k_clear_RC_count*RC_count;

else

% correct for RC ubiquination while licensed

clear_RC_count = u_RC_on_DNA + u_RC_licensed + u_RC_traversed_by_DNA_poly;

end

RC_count = add_to_RC_count - clear_RC_count;

% 17 DNA_poly STATES

mRNA_DNA_poly_unspl = txs_mRNA_DNA_poly - spl_mRNA_DNA_poly...

- dk_mRNA_DNA_poly_unspl - k_div*mRNA_DNA_poly_unspl;

mRNA_DNA_poly_nuc = spl_mRNA_DNA_poly - nm_out_mRNA_DNA_poly...

- dk_mRNA_DNA_poly_nuc - k_div*mRNA_DNA_poly_nuc;

mRNA_DNA_poly_inactive = nm_out_mRNA_DNA_poly - activate_mRNA_DNA_poly...

- dk_mRNA_DNA_poly_inactive - k_div*mRNA_DNA_poly_inactive;

mRNA_DNA_poly_cytosol = activate_mRNA_DNA_poly + unbind_mRNA_DNA_poly...

- bind_mRNA_DNA_poly - dk_mRNA_DNA_poly_cytosol...

- k_div*mRNA_DNA_poly_cytosol;

mRNA_DNA_poly_in_use = bind_mRNA_DNA_poly - unbind_mRNA_DNA_poly...

- dk_mRNA_DNA_poly_in_use - k_div*mRNA_DNA_poly_in_use;

mRNA_DNA_poly_trash = dk_mRNA_DNA_poly_cytosol + dk_mRNA_DNA_poly_unspl...

+ dk_mRNA_DNA_poly_nuc + dk_mRNA_DNA_poly_inactive...

+ dk_mRNA_DNA_poly_in_use...

- cut_mRNA_DNA_poly - k_div*mRNA_DNA_poly_trash;

DNA_poly_unfold = txl_DNA_poly - fold_DNA_poly - u_DNA_poly_unfold...

- k_div*DNA_poly_unfold;

DNA_poly_cytosol = fold_DNA_poly - nm_in_DNA_poly - u_DNA_poly_cytosol...

- k_div*DNA_poly_cytosol;

DNA_poly_nuc = nm_in_DNA_poly + unbind_DNA_poly_from_DNA - bind_DNA_poly_to_DNA...

- u_DNA_poly_nuc - k_div*DNA_poly_nuc;

DNA_poly_on_DNA = bind_DNA_poly_to_DNA - unbind_DNA_poly_from_DNA...

- u_DNA_poly_on_DNA - k_div*DNA_poly_on_DNA;

DNA_poly_trash = u_DNA_poly_nuc + u_DNA_poly_unfold + u_DNA_poly_cytosol...

+ u_DNA_poly_on_DNA - cut_DNA_poly - k_div*DNA_poly_trash;

% 18 Wee1 STATES

mRNA_Wee1_unspl = txs_mRNA_Wee1 - spl_mRNA_Wee1 - dk_mRNA_Wee1_unspl...

- k_div*mRNA_Wee1_unspl;

mRNA_Wee1_nuc = spl_mRNA_Wee1 - nm_out_mRNA_Wee1 - dk_mRNA_Wee1_nuc...

- k_div*mRNA_Wee1_nuc;

mRNA_Wee1_inactive = nm_out_mRNA_Wee1 - activate_mRNA_Wee1...

- dk_mRNA_Wee1_inactive - k_div*mRNA_Wee1_inactive;

mRNA_Wee1_cytosol = activate_mRNA_Wee1 + unbind_mRNA_Wee1 - bind_mRNA_Wee1...

- dk_mRNA_Wee1_cytosol - k_div*mRNA_Wee1_cytosol;

mRNA_Wee1_in_use = bind_mRNA_Wee1 - unbind_mRNA_Wee1 - dk_mRNA_Wee1_in_use...

- k_div*mRNA_Wee1_in_use;

mRNA_Wee1_trash = dk_mRNA_Wee1_cytosol + dk_mRNA_Wee1_unspl + dk_mRNA_Wee1_nuc...

+ dk_mRNA_Wee1_inactive + dk_mRNA_Wee1_in_use...

- cut_mRNA_Wee1 - k_div*mRNA_Wee1_trash;

Wee1_unfold = txl_Wee1 - fold_Wee1 - u_Wee1_unfold - k_div*Wee1_unfold;

% ASSUME Wee1 is restricted to the cytoplasm

Wee1_cytosol_active = fold_Wee1 - phospho_Wee1_by_Cdk1_and_Plk1...

+ unphospho_Wee1 + dephospho_Wee1_by_Cdc14...

- u_Wee1_cytosol_active - k_div*Wee1_cytosol_active;

Wee1_phospho_inactive = phospho_Wee1_by_Cdk1_and_Plk1...

- unphospho_Wee1 - dephospho_Wee1_by_Cdc14...

- u_Wee1_phospho_inactive - k_div*Wee1_phospho_inactive;

Wee1_trash = u_Wee1_unfold + u_Wee1_cytosol_active + u_Wee1_phospho_inactive...

- cut_Wee1 - k_div*Wee1_trash;

% 19 cycB STATES

mRNA_cycB_unspl = txs_mRNA_cycB - spl_mRNA_cycB - dk_mRNA_cycB_unspl...

- k_div*mRNA_cycB_unspl;

mRNA_cycB_nuc = spl_mRNA_cycB - nm_out_mRNA_cycB - dk_mRNA_cycB_nuc...

- k_div*mRNA_cycB_nuc;

mRNA_cycB_inactive = nm_out_mRNA_cycB - activate_mRNA_cycB...

- dk_mRNA_cycB_inactive - k_div*mRNA_cycB_inactive;

mRNA_cycB_cytosol = activate_mRNA_cycB + unbind_mRNA_cycB - bind_mRNA_cycB...

- dk_mRNA_cycB_cytosol - k_div*mRNA_cycB_cytosol;

mRNA_cycB_in_use = bind_mRNA_cycB - unbind_mRNA_cycB - dk_mRNA_cycB_in_use...

- k_div*mRNA_cycB_in_use;

mRNA_cycB_trash = dk_mRNA_cycB_cytosol + dk_mRNA_cycB_unspl + dk_mRNA_cycB_nuc...

+ dk_mRNA_cycB_inactive + dk_mRNA_cycB_in_use...

- cut_mRNA_cycB - k_div*mRNA_cycB_trash;

cycB_unfold = txl_cycB - fold_cycB - u_cycB_unfold - k_div*cycB_unfold;

% and correct for loss of Cdk1

cycB_cytosol = fold_cycB - bind_cycB_to_Cdk1 + unbind_cycB_Cdk1...

- u_cycB_cytosol - k_div*cycB_cytosol...

+ u_Cdk1_on_cycB_phospho_inactive_frac...

+ u_Cdk1_on_cycB_nuc_active_frac;

cycB_Cdk1_cytosol_active = bind_cycB_to_Cdk1 - unbind_cycB_Cdk1...

- phospho_cycB_Cdk1_by_Wee1...

+ dephospho_cycB_Cdk1_by_Cdc25...

- nm_in_cycB_Cdk1 + nm_out_cycB_Cdk1...

- u_cycB_Cdk1_cytosol_active...

- k_div*cycB_Cdk1_cytosol_active;

% and correct for loss of Cdk1

cycB_Cdk1_phospho_inactive = phospho_cycB_Cdk1_by_Wee1...

- dephospho_cycB_Cdk1_by_Cdc25...

- u_cycB_Cdk1_phospho_inactive...

- k_div*cycB_Cdk1_phospho_inactive...

- u_Cdk1_on_cycB_phospho_inactive_frac;

% and correct for loss of Cdk1

cycB_Cdk1_nuc_active = nm_in_cycB_Cdk1 - nm_out_cycB_Cdk1...

- u_cycB_Cdk1_nuc_active - k_div*cycB_Cdk1_nuc_active...

- u_Cdk1_on_cycB_nuc_active_frac;

cycB_trash = u_cycB_unfold + u_cycB_cytosol...

+ u_cycB_Cdk1_cytosol_active...

+ u_cycB_Cdk1_phospho_inactive + u_cycB_Cdk1_nuc_active...

- cut_cycB - k_div*cycB_trash;

% 20 Cdk1 STATES

mRNA_Cdk1_unspl = txs_mRNA_Cdk1 - spl_mRNA_Cdk1 - dk_mRNA_Cdk1_unspl...

- k_div*mRNA_Cdk1_unspl;

mRNA_Cdk1_nuc = spl_mRNA_Cdk1 - nm_out_mRNA_Cdk1 - dk_mRNA_Cdk1_nuc...

- k_div*mRNA_Cdk1_nuc;

mRNA_Cdk1_inactive = nm_out_mRNA_Cdk1 - activate_mRNA_Cdk1...

- dk_mRNA_Cdk1_inactive - k_div*mRNA_Cdk1_inactive;

mRNA_Cdk1_cytosol = activate_mRNA_Cdk1 + unbind_mRNA_Cdk1 - bind_mRNA_Cdk1...

- dk_mRNA_Cdk1_cytosol - k_div*mRNA_Cdk1_cytosol;

mRNA_Cdk1_in_use = bind_mRNA_Cdk1 - unbind_mRNA_Cdk1 - dk_mRNA_Cdk1_in_use...

- k_div*mRNA_Cdk1_in_use;

mRNA_Cdk1_trash = dk_mRNA_Cdk1_cytosol + dk_mRNA_Cdk1_unspl + dk_mRNA_Cdk1_nuc...

+ dk_mRNA_Cdk1_inactive + dk_mRNA_Cdk1_in_use...

- cut_mRNA_Cdk1 - k_div*mRNA_Cdk1_trash;

Cdk1_unfold = txl_Cdk1 - fold_Cdk1 - u_Cdk1_unfold - k_div*Cdk1_unfold;

% Cdk1_inactive = fold_Cdk1 - activate_Cdk1 - u_Cdk1_inactive - k_div*Cdk1_inactive;

Cdk1_inactive = fold_Cdk1 - activate_Cdk1...

+ deactivate_Cdk1 + dephospho_Cdk1_by_Cdc14...

- u_Cdk1_inactive - k_div*Cdk1_inactive;

% ASSUME Cdk1 moves freely in and out of nucleus

% Cdk1_in_cell = activate_Cdk1...

% + unbind_Cdk1_cycB - bind_Cdk1_to_cycB...

% + unbind_Cdk1_cycA - bind_Cdk1_to_cycA...

% - u_Cdk1_in_cell - k_div*Cdk1_in_cell;

Cdk1_in_cell = activate_Cdk1 - deactivate_Cdk1 - dephospho_Cdk1_by_Cdc14...

+ unbind_Cdk1_cycB - bind_Cdk1_to_cycB...

+ unbind_Cdk1_cycA - bind_Cdk1_to_cycA...

- u_Cdk1_in_cell - k_div*Cdk1_in_cell;

Cdk1_on_cycB = bind_Cdk1_to_cycB - unbind_Cdk1_cycB...

- k_div*Cdk1_on_cycB - u_Cdk1_on_cycB;

Cdk1_on_cycA = bind_Cdk1_to_cycA - unbind_Cdk1_cycA...

- k_div*Cdk1_on_cycA - u_Cdk1_on_cycA;

Cdk1_trash = u_Cdk1_unfold + u_Cdk1_inactive + u_Cdk1_in_cell...

+ u_Cdk1_on_cycB + u_Cdk1_on_cycA...

- cut_Cdk1 - k_div*Cdk1_trash;

% 21 Cdc25C STATES

mRNA_Cdc25C_unspl = txs_mRNA_Cdc25C - spl_mRNA_Cdc25C - dk_mRNA_Cdc25C_unspl...

- k_div*mRNA_Cdc25C_unspl;

mRNA_Cdc25C_nuc = spl_mRNA_Cdc25C - nm_out_mRNA_Cdc25C - dk_mRNA_Cdc25C_nuc...

- k_div*mRNA_Cdc25C_nuc;

mRNA_Cdc25C_inactive = nm_out_mRNA_Cdc25C - activate_mRNA_Cdc25C...

- dk_mRNA_Cdc25C_inactive - k_div*mRNA_Cdc25C_inactive;

mRNA_Cdc25C_cytosol = activate_mRNA_Cdc25C + unbind_mRNA_Cdc25C - bind_mRNA_Cdc25C...

- dk_mRNA_Cdc25C_cytosol - k_div*mRNA_Cdc25C_cytosol;

mRNA_Cdc25C_in_use = bind_mRNA_Cdc25C - unbind_mRNA_Cdc25C - dk_mRNA_Cdc25C_in_use...

- k_div*mRNA_Cdc25C_in_use;

mRNA_Cdc25C_trash = dk_mRNA_Cdc25C_cytosol + dk_mRNA_Cdc25C_unspl...

+ dk_mRNA_Cdc25C_nuc + dk_mRNA_Cdc25C_inactive...

+ dk_mRNA_Cdc25C_in_use - cut_mRNA_Cdc25C - k_div*mRNA_Cdc25C_trash;

Cdc25C_unfold = txl_Cdc25C - fold_Cdc25C - u_Cdc25C_unfold - k_div*Cdc25C_unfold;

Cdc25C_cytosol_inactive = fold_Cdc25C...

- phospho_Cdc25C_by_Cdk1 - phospho_Cdc25C_by_Plk1...

+ unphospho_Cdc25C + dephospho_Cdc25C_by_Cdc14...

- u_Cdc25C_cytosol_inactive - k_div*Cdc25C_cytosol_inactive;

Cdc25C_cytosol_phospho_active = phospho_Cdc25C_by_Cdk1 + phospho_Cdc25C_by_Plk1...

- unphospho_Cdc25C - nm_in_Cdc25C + nm_out_Cdc25C...

- u_Cdc25C_cytosol_phospho_active...

- k_div*Cdc25C_cytosol_phospho_active;

% Cdc25C must be nuclear to be inactivated by Cdc14

% ASSUME that after inactivation, Cdc25C returns to Cdc25C_cytosol_inactive

Cdc25C_nuc_phospho_active = nm_in_Cdc25C - nm_out_Cdc25C...

- dephospho_Cdc25C_by_Cdc14...

- u_Cdc25C_nuc_phospho_active...

- k_div*Cdc25C_nuc_phospho_active;

Cdc25C_trash = u_Cdc25C_unfold + u_Cdc25C_cytosol_inactive...

+ u_Cdc25C_cytosol_phospho_active + u_Cdc25C_nuc_phospho_active...

- cut_Cdc25C - k_div*Cdc25C_trash;

% 22 Plk1 STATES

mRNA_Plk1_unspl = txs_mRNA_Plk1 - spl_mRNA_Plk1 - dk_mRNA_Plk1_unspl...

- k_div*mRNA_Plk1_unspl;

mRNA_Plk1_nuc = spl_mRNA_Plk1 - nm_out_mRNA_Plk1 - dk_mRNA_Plk1_nuc...

- k_div*mRNA_Plk1_nuc;

mRNA_Plk1_inactive = nm_out_mRNA_Plk1 - activate_mRNA_Plk1...

- dk_mRNA_Plk1_inactive - k_div*mRNA_Plk1_inactive;

mRNA_Plk1_cytosol = activate_mRNA_Plk1 + unbind_mRNA_Plk1 - bind_mRNA_Plk1...

- dk_mRNA_Plk1_cytosol - k_div*mRNA_Plk1_cytosol;

mRNA_Plk1_in_use = bind_mRNA_Plk1 - unbind_mRNA_Plk1 - dk_mRNA_Plk1_in_use...

- k_div*mRNA_Plk1_in_use;

mRNA_Plk1_trash = dk_mRNA_Plk1_cytosol + dk_mRNA_Plk1_unspl + dk_mRNA_Plk1_nuc...

+ dk_mRNA_Plk1_inactive + dk_mRNA_Plk1_in_use...

- cut_mRNA_Plk1 - k_div*mRNA_Plk1_trash;

Plk1_unfold = txl_Plk1 - fold_Plk1 - u_Plk1_unfold - k_div*Plk1_unfold;

Plk1_cytosol_inactive = fold_Plk1...

- phospho_Plk1_by_Cdk1 + unphospho_Plk1...

- u_Plk1_cytosol_inactive - k_div*Plk1_cytosol_inactive;

Plk1_cytosol_phospho_active = phospho_Plk1_by_Cdk1 - unphospho_Plk1...

- nm_in_Plk1...

- u_Plk1_cytosol_phospho_active...

- k_div*Plk1_cytosol_phospho_active;

Plk1_nuc_phospho_active = nm_in_Plk1...

- u_Plk1_nuc_phospho_active...

- k_div*Plk1_nuc_phospho_active;

Plk1_trash = u_Plk1_unfold + u_Plk1_cytosol_inactive...

+ u_Plk1_cytosol_phospho_active + u_Plk1_nuc_phospho_active...

- cut_Plk1 - k_div*Plk1_trash;

% 23 Emi1 STATES

mRNA_Emi1_unspl = txs_mRNA_Emi1 - spl_mRNA_Emi1 - dk_mRNA_Emi1_unspl...

- k_div*mRNA_Emi1_unspl;

mRNA_Emi1_nuc = spl_mRNA_Emi1 - nm_out_mRNA_Emi1 - dk_mRNA_Emi1_nuc...

- k_div*mRNA_Emi1_nuc;

mRNA_Emi1_inactive = nm_out_mRNA_Emi1 - activate_mRNA_Emi1...

- dk_mRNA_Emi1_inactive - k_div*mRNA_Emi1_inactive;

mRNA_Emi1_cytosol = activate_mRNA_Emi1 + unbind_mRNA_Emi1 - bind_mRNA_Emi1...

- dk_mRNA_Emi1_cytosol - k_div*mRNA_Emi1_cytosol;

mRNA_Emi1_in_use = bind_mRNA_Emi1 - unbind_mRNA_Emi1 - dk_mRNA_Emi1_in_use...

- k_div*mRNA_Emi1_in_use;

mRNA_Emi1_trash = dk_mRNA_Emi1_cytosol + dk_mRNA_Emi1_unspl + dk_mRNA_Emi1_nuc...

+ dk_mRNA_Emi1_inactive + dk_mRNA_Emi1_in_use...

- cut_mRNA_Emi1 - k_div*mRNA_Emi1_trash;

Emi1_unfold = txl_Emi1 - fold_Emi1 - u_Emi1_unfold - k_div*Emi1_unfold;

% ASSUME Emi1 moves freely in and out of nucleus

Emi1_in_cell = fold_Emi1 - bind_Emi1_to_Cdh1 + unbind_Emi1_Cdh1...

- bind_Emi1_to_Cdc20 + unbind_Emi1_Cdc20 - phospho_Emi1_in_cell...

- u_Emi1_in_cell - k_div*Emi1_in_cell;

Emi1_on_Cdh1 = bind_Emi1_to_Cdh1 - unbind_Emi1_Cdh1 - phospho_Emi1_on_Cdh1...

- u_Emi1_on_Cdh1 - k_div*Emi1_on_Cdh1;

Emi1_on_Cdc20 = bind_Emi1_to_Cdc20 - unbind_Emi1_Cdc20 - phospho_Emi1_on_Cdc20...

- u_Emi1_on_Cdc20 - k_div*Emi1_on_Cdc20;

Emi1_phospho_inactive = phospho_Emi1_in_cell...

+ phospho_Emi1_on_Cdh1 + phospho_Emi1_on_Cdc20...

- u_Emi1_phospho_inactive - k_div*Emi1_phospho_inactive;

Emi1_trash = u_Emi1_phospho_inactive - cut_Emi1 - k_div*Emi1_trash;

% 24 APC STATES

mRNA_APC_unspl = txs_mRNA_APC - spl_mRNA_APC - dk_mRNA_APC_unspl...

- k_div*mRNA_APC_unspl;

mRNA_APC_nuc = spl_mRNA_APC - nm_out_mRNA_APC - dk_mRNA_APC_nuc...

- k_div*mRNA_APC_nuc;

mRNA_APC_inactive = nm_out_mRNA_APC - activate_mRNA_APC...

- dk_mRNA_APC_inactive - k_div*mRNA_APC_inactive;

mRNA_APC_cytosol = activate_mRNA_APC + unbind_mRNA_APC - bind_mRNA_APC...

- dk_mRNA_APC_cytosol - k_div*mRNA_APC_cytosol;

mRNA_APC_in_use = bind_mRNA_APC - unbind_mRNA_APC - dk_mRNA_APC_in_use...

- k_div*mRNA_APC_in_use;

mRNA_APC_trash = dk_mRNA_APC_cytosol + dk_mRNA_APC_unspl + dk_mRNA_APC_nuc...

+ dk_mRNA_APC_inactive + dk_mRNA_APC_in_use...

- cut_mRNA_APC - k_div*mRNA_APC_trash;

APC_unfold = txl_APC - fold_APC - u_APC_unfold - k_div*APC_unfold;

APC_in_cell = fold_APC...

- bind_APC_to_Cdh1 + unbind_APC_Cdh1...

- bind_APC_to_Cdc20 + unbind_APC_Cdc20...

- u_APC_in_cell - k_div*APC_in_cell;

% APC_cytosol = fold_APC - nm_in_APC - u_APC_cytosol - k_div*APC_cytosol;

% APC_nuc = nm_in_APC - bind_APC_to_Cdh1 + unbind_APC_Cdh1...

% - bind_APC_to_Cdc20 + unbind_APC_Cdc20...

% - u_APC_nuc - k_div*APC_nuc;

APC_on_Cdh1 = bind_APC_to_Cdh1 - unbind_APC_Cdh1 - u_APC_on_Cdh1...

- k_div*APC_on_Cdh1;

APC_on_Cdc20 = bind_APC_to_Cdc20 - unbind_APC_Cdc20 - u_APC_on_Cdc20...

- k_div*APC_on_Cdc20;

% APC_trash = u_APC_unfold + u_APC_cytosol + u_APC_nuc + u_APC_on_Cdh1...

APC_trash = u_APC_unfold + u_APC_in_cell + u_APC_on_Cdh1...

+ u_APC_on_Cdc20 - cut_APC - k_div*APC_trash;

% 25 Cdh1 STATES

mRNA_Cdh1_unspl = txs_mRNA_Cdh1 - spl_mRNA_Cdh1 - dk_mRNA_Cdh1_unspl...

- k_div*mRNA_Cdh1_unspl;

mRNA_Cdh1_nuc = spl_mRNA_Cdh1 - nm_out_mRNA_Cdh1 - dk_mRNA_Cdh1_nuc...

- k_div*mRNA_Cdh1_nuc;

mRNA_Cdh1_inactive = nm_out_mRNA_Cdh1 - activate_mRNA_Cdh1...

- dk_mRNA_Cdh1_inactive - k_div*mRNA_Cdh1_inactive;

mRNA_Cdh1_cytosol = activate_mRNA_Cdh1 + unbind_mRNA_Cdh1 - bind_mRNA_Cdh1...

- dk_mRNA_Cdh1_cytosol - k_div*mRNA_Cdh1_cytosol;

mRNA_Cdh1_in_use = bind_mRNA_Cdh1 - unbind_mRNA_Cdh1 - dk_mRNA_Cdh1_in_use...

- k_div*mRNA_Cdh1_in_use;

mRNA_Cdh1_trash = dk_mRNA_Cdh1_cytosol + dk_mRNA_Cdh1_unspl + dk_mRNA_Cdh1_nuc...

+ dk_mRNA_Cdh1_inactive + dk_mRNA_Cdh1_in_use...

- cut_mRNA_Cdh1 - k_div*mRNA_Cdh1_trash;

Cdh1_unfold = txl_Cdh1 - fold_Cdh1 - u_Cdh1_unfold - k_div*Cdh1_unfold;

% and correct for loss of Emi1

Cdh1_in_cell = fold_Cdh1...

- phospho_Cdh1_in_cell_by_Cdk1 + dephospho_Cdh1_by_Cdc14...

- bind_Cdh1_to_APC + unbind_Cdh1_APC...

- u_Cdh1_in_cell - k_div*Cdh1_in_cell...

+ u_Emi1_on_Cdh1 + phospho_Emi1_on_Cdh1;

% Cdh1_cytosol = fold_Cdh1 - nm_in_Cdh1 - u_Cdh1_cytosol - k_div*Cdh1_cytosol;

% and correct for loss of Emi1

% Cdh1_nuc = nm_in_Cdh1...

% - phospho_Cdh1_nuc_by_Cdk1 + dephospho_Cdh1_by_Cdc14...

% - bind_Cdh1_to_APC + unbind_Cdh1_APC...

% - u_Cdh1_nuc - k_div*Cdh1_nuc...

% + u_Emi1_on_Cdh1 + phospho_Emi1_on_Cdh1;

% and correct for loss of Emi1

Cdh1_Emi1_inactive = bind_Cdh1_to_Emi1 - unbind_Cdh1_Emi1 - u_Cdh1_Emi1_inactive...

- k_div*Cdh1_Emi1_inactive...

- u_Emi1_on_Cdh1 - phospho_Emi1_on_Cdh1;

% Cdh1_phospho_inactive = phospho_Cdh1_nuc_by_Cdk1 + phospho_Cdh1_APC_active_by_Cdk...

Cdh1_phospho_inactive = phospho_Cdh1_in_cell_by_Cdk1 + phospho_Cdh1_APC_active_by_Cdk...

- dephospho_Cdh1_by_Cdc14...

- bind_Cdh1_to_Emi1 + unbind_Cdh1_Emi1...

- u_Cdh1_phospho_inactive - k_div*Cdh1_phospho_inactive;

Cdh1_APC_active = bind_Cdh1_to_APC - unbind_Cdh1_APC...

- phospho_Cdh1_APC_active_by_Cdk...

- u_Cdh1_APC_active - k_div*Cdh1_APC_active;

% Cdh1_trash = u_Cdh1_unfold + u_Cdh1_cytosol + u_Cdh1_nuc...

Cdh1_trash = u_Cdh1_unfold + u_Cdh1_in_cell...

+ u_Cdh1_Emi1_inactive + u_Cdh1_phospho_inactive...

+ u_Cdh1_APC_active - cut_Cdh1 - k_div*Cdh1_trash;

% 26 Cdc20 STATES

mRNA_Cdc20_unspl = txs_mRNA_Cdc20 - spl_mRNA_Cdc20 - dk_mRNA_Cdc20_unspl...

- k_div*mRNA_Cdc20_unspl;

mRNA_Cdc20_nuc = spl_mRNA_Cdc20 - nm_out_mRNA_Cdc20 - dk_mRNA_Cdc20_nuc...

- k_div*mRNA_Cdc20_nuc;

mRNA_Cdc20_inactive = nm_out_mRNA_Cdc20 - activate_mRNA_Cdc20...

- dk_mRNA_Cdc20_inactive - k_div*mRNA_Cdc20_inactive;

mRNA_Cdc20_cytosol = activate_mRNA_Cdc20 + unbind_mRNA_Cdc20 - bind_mRNA_Cdc20...

- dk_mRNA_Cdc20_cytosol - k_div*mRNA_Cdc20_cytosol;

mRNA_Cdc20_in_use = bind_mRNA_Cdc20 - unbind_mRNA_Cdc20 - dk_mRNA_Cdc20_in_use...

- k_div*mRNA_Cdc20_in_use;

mRNA_Cdc20_trash = dk_mRNA_Cdc20_cytosol + dk_mRNA_Cdc20_unspl + dk_mRNA_Cdc20_nuc...

+ dk_mRNA_Cdc20_inactive + dk_mRNA_Cdc20_in_use...

- cut_mRNA_Cdc20 - k_div*mRNA_Cdc20_trash;

Cdc20_unfold = txl_Cdc20 - fold_Cdc20 - u_Cdc20_unfold - k_div*Cdc20_unfold;

Cdc20_cytosol = fold_Cdc20 - nm_in_Cdc20...

- u_Cdc20_cytosol - k_div*Cdc20_cytosol;

% and correct for loss of APC

Cdc20_nuc = nm_in_Cdc20 - bind_Cdc20_to_Emi1 + unbind_Cdc20_Emi1...

- bind_Cdc20_to_APC + unbind_Cdc20_APC...

+ dephospho_Cdc20_APC_inactive_by_Cdc14...

+ dephospho_Cdc20_APC_active_by_Cdc14...

- u_Cdc20_nuc - k_div*Cdc20_nuc...

+ u_APC_on_Cdc20_active_frac;

Cdc20_Emi1_inactive = bind_Cdc20_to_Emi1 - unbind_Cdc20_Emi1 - u_Cdc20_Emi1_inactive...

- k_div*Cdc20_Emi1_inactive;

Cdc20_APC_inactive = bind_Cdc20_to_APC - unbind_Cdc20_APC...

- dephospho_Cdc20_APC_inactive_by_Cdc14...

- phospho_Cdc20_by_Cdk1 + unphospho_Cdc20_APC...

- u_Cdc20_APC_inactive - k_div*Cdc20_APC_inactive;

% and correct for loss of APC

Cdc20_APC_active = phospho_Cdc20_by_Cdk1 - unphospho_Cdc20_APC...

- dephospho_Cdc20_APC_active_by_Cdc14...

- u_Cdc20_APC_active - k_div*Cdc20_APC_active...

- u_APC_on_Cdc20_active_frac;

Cdc20_trash = u_Cdc20_unfold + u_Cdc20_cytosol + u_Cdc20_nuc...

+ u_Cdc20_Emi1_inactive + u_Cdc20_APC_inactive...

+ u_Cdc20_APC_active - cut_Cdc20 - k_div*Cdc20_trash;

% 27 Cdc14 STATES

mRNA_Cdc14_unspl = txs_mRNA_Cdc14 - spl_mRNA_Cdc14 - dk_mRNA_Cdc14_unspl...

- k_div*mRNA_Cdc14_unspl;

mRNA_Cdc14_nuc = spl_mRNA_Cdc14 - nm_out_mRNA_Cdc14 - dk_mRNA_Cdc14_nuc...

- k_div*mRNA_Cdc14_nuc;

mRNA_Cdc14_inactive = nm_out_mRNA_Cdc14 - activate_mRNA_Cdc14...

- dk_mRNA_Cdc14_inactive - k_div*mRNA_Cdc14_inactive;

mRNA_Cdc14_cytosol = activate_mRNA_Cdc14 + unbind_mRNA_Cdc14 - bind_mRNA_Cdc14...

- dk_mRNA_Cdc14_cytosol - k_div*mRNA_Cdc14_cytosol;

mRNA_Cdc14_in_use = bind_mRNA_Cdc14 - unbind_mRNA_Cdc14 - dk_mRNA_Cdc14_in_use...

- k_div*mRNA_Cdc14_in_use;

mRNA_Cdc14_trash = dk_mRNA_Cdc14_cytosol + dk_mRNA_Cdc14_unspl + dk_mRNA_Cdc14_nuc...

+ dk_mRNA_Cdc14_inactive + dk_mRNA_Cdc14_in_use...

- cut_mRNA_Cdc14 - k_div*mRNA_Cdc14_trash;

Cdc14_unfold = txl_Cdc14 - fold_Cdc14 - u_Cdc14_unfold - k_div*Cdc14_unfold;

Cdc14_cytosol = fold_Cdc14 - nm_in_Cdc14 - u_Cdc14_cytosol - k_div*Cdc14_cytosol;

Cdc14_nuc_inactive = nm_in_Cdc14 + unphospho_Cdc14 - phospho_Cdc14_by_Plk1...

- inhibit_Cdc14_by_Securin + uninhibit_Cdc14...

- u_Cdc14_nuc_inactive - k_div*Cdc14_nuc_inactive;

Cdc14_inhibited = inhibit_Cdc14_by_Securin - uninhibit_Cdc14...

- u_Cdc14_inhibited - k_div*Cdc14_inhibited;

Cdc14_phospho_active = phospho_Cdc14_by_Plk1 - unphospho_Cdc14...

- u_Cdc14_phospho_active - k_div*Cdc14_phospho_active;

Cdc14_trash = u_Cdc14_unfold + u_Cdc14_cytosol + u_Cdc14_nuc_inactive...

+ u_Cdc14_inhibited + u_Cdc14_phospho_active...

- cut_Cdc14 - k_div*Cdc14_trash;

% 28 Cdc25A STATES

mRNA_Cdc25A_unspl = txs_mRNA_Cdc25A - spl_mRNA_Cdc25A - dk_mRNA_Cdc25A_unspl...

- k_div*mRNA_Cdc25A_unspl;

mRNA_Cdc25A_nuc = spl_mRNA_Cdc25A - nm_out_mRNA_Cdc25A - dk_mRNA_Cdc25A_nuc...

- k_div*mRNA_Cdc25A_nuc;

mRNA_Cdc25A_inactive = nm_out_mRNA_Cdc25A - activate_mRNA_Cdc25A...

- dk_mRNA_Cdc25A_inactive - k_div*mRNA_Cdc25A_inactive;

mRNA_Cdc25A_cytosol = activate_mRNA_Cdc25A + unbind_mRNA_Cdc25A - bind_mRNA_Cdc25A...

- dk_mRNA_Cdc25A_cytosol - k_div*mRNA_Cdc25A_cytosol;

mRNA_Cdc25A_in_use = bind_mRNA_Cdc25A - unbind_mRNA_Cdc25A - dk_mRNA_Cdc25A_in_use...

- k_div*mRNA_Cdc25A_in_use;

mRNA_Cdc25A_trash = dk_mRNA_Cdc25A_cytosol + dk_mRNA_Cdc25A_unspl + dk_mRNA_Cdc25A_nuc...

+ dk_mRNA_Cdc25A_inactive + dk_mRNA_Cdc25A_in_use...

- cut_mRNA_Cdc25A - k_div*mRNA_Cdc25A_trash;

Cdc25A_unfold = txl_Cdc25A - fold_Cdc25A - u_Cdc25A_unfold - k_div*Cdc25A_unfold;

Cdc25A_cytosol = fold_Cdc25A - nm_in_Cdc25A - u_Cdc25A_cytosol...

- k_div*Cdc25A_cytosol;

Cdc25A_nuc_inactive = nm_in_Cdc25A + dephospho_Cdc25A_by_Cdc14...

- phospho_Cdc25A_by_Cdk2 - phospho_Cdc25A_by_Cdk1...

- u_Cdc25A_nuc_inactive - k_div*Cdc25A_nuc_inactive;

Cdc25A_phospho_active = phospho_Cdc25A_by_Cdk2 + phospho_Cdc25A_by_Cdk1...

- dephospho_Cdc25A_by_Cdc14 - u_Cdc25A_phospho_active...

- k_div*Cdc25A_phospho_active;

Cdc25A_trash = u_Cdc25A_unfold + u_Cdc25A_cytosol + u_Cdc25A_nuc_inactive...

+ u_Cdc25A_phospho_active - cut_Cdc25A - k_div*Cdc25A_trash;

% 29 Cdc25B STATES

mRNA_Cdc25B_unspl = txs_mRNA_Cdc25B - spl_mRNA_Cdc25B - dk_mRNA_Cdc25B_unspl...

- k_div*mRNA_Cdc25B_unspl;

mRNA_Cdc25B_nuc = spl_mRNA_Cdc25B - nm_out_mRNA_Cdc25B - dk_mRNA_Cdc25B_nuc...

- k_div*mRNA_Cdc25B_nuc;

mRNA_Cdc25B_inactive = nm_out_mRNA_Cdc25B - activate_mRNA_Cdc25B...

- dk_mRNA_Cdc25B_inactive - k_div*mRNA_Cdc25B_inactive;

mRNA_Cdc25B_cytosol = activate_mRNA_Cdc25B + unbind_mRNA_Cdc25B - bind_mRNA_Cdc25B...

- dk_mRNA_Cdc25B_cytosol - k_div*mRNA_Cdc25B_cytosol;

mRNA_Cdc25B_in_use = bind_mRNA_Cdc25B - unbind_mRNA_Cdc25B - dk_mRNA_Cdc25B_in_use...

- k_div*mRNA_Cdc25B_in_use;

mRNA_Cdc25B_trash = dk_mRNA_Cdc25B_cytosol + dk_mRNA_Cdc25B_unspl...

+ dk_mRNA_Cdc25B_nuc...

+ dk_mRNA_Cdc25B_inactive + dk_mRNA_Cdc25B_in_use...

- cut_mRNA_Cdc25B - k_div*mRNA_Cdc25B_trash;

Cdc25B_unfold = txl_Cdc25B - fold_Cdc25B - u_Cdc25B_unfold - k_div*Cdc25B_unfold;

Cdc25B_cytosol_inactive = fold_Cdc25B + dephospho_Cdc25B_by_Cdc14...

- phospho_Cdc25B_by_Cdk2 - phospho_Cdc25B_by_Cdk1...

- phospho_Cdc25B_by_TF_grow...

- phospho_Cdc25B_by_Plk1...

- u_Cdc25B_cytosol_inactive...

- k_div*Cdc25B_cytosol_inactive;

Cdc25B_cytosol_phospho_active = phospho_Cdc25B_by_Cdk2 + phospho_Cdc25B_by_Cdk1...

+ phospho_Cdc25B_by_TF_grow...

+ phospho_Cdc25B_by_Plk1...

- dephospho_Cdc25B_by_Cdc14...

- u_Cdc25B_cytosol_phospho_active...

- k_div*Cdc25B_cytosol_phospho_active;

Cdc25B_trash = u_Cdc25B_unfold + u_Cdc25B_cytosol_inactive...

+ u_Cdc25B_cytosol_phospho_active...

- cut_Cdc25B - k_div*Cdc25B_trash;

% 30 Securin STATES

mRNA_Securin_unspl = txs_mRNA_Securin - spl_mRNA_Securin - dk_mRNA_Securin_unspl...

- k_div*mRNA_Securin_unspl;

mRNA_Securin_nuc = spl_mRNA_Securin - nm_out_mRNA_Securin - dk_mRNA_Securin_nuc...

- k_div*mRNA_Securin_nuc;

mRNA_Securin_inactive = nm_out_mRNA_Securin - activate_mRNA_Securin...

- dk_mRNA_Securin_inactive - k_div*mRNA_Securin_inactive;

mRNA_Securin_cytosol = activate_mRNA_Securin + unbind_mRNA_Securin - bind_mRNA_Securin...

- dk_mRNA_Securin_cytosol - k_div*mRNA_Securin_cytosol;

mRNA_Securin_in_use = bind_mRNA_Securin - unbind_mRNA_Securin...

- dk_mRNA_Securin_in_use - k_div*mRNA_Securin_in_use;

mRNA_Securin_trash = dk_mRNA_Securin_cytosol + dk_mRNA_Securin_unspl...

+ dk_mRNA_Securin_nuc + dk_mRNA_Securin_inactive...

+ dk_mRNA_Securin_in_use...

- cut_mRNA_Securin - k_div*mRNA_Securin_trash;

Securin_unfold = txl_Securin - fold_Securin - u_Securin_unfold...

- k_div*Securin_unfold;

Securin_cytosol = fold_Securin - nm_in_Securin - u_Securin_cytosol...

- k_div*Securin_cytosol;

Securin_nuc = nm_in_Securin - bind_Securin_to_chromo - u_Securin_nuc...

- k_div*Securin_nuc;

Securin_on_chromo = bind_Securin_to_chromo - u_Securin_on_chromo...

- k_div*Securin_on_chromo;

Securin_trash = u_Securin_unfold + u_Securin_cytosol + u_Securin_nuc...

+ u_Securin_on_chromo - cut_Securin - k_div*Securin_trash;

% 31 cycC/Cdk8

mRNA_cycC_unspl = txs_mRNA_cycC - spl_mRNA_cycC - dk_mRNA_cycC_unspl...

- k_div*mRNA_cycC_unspl;

mRNA_cycC_nuc = spl_mRNA_cycC - nm_out_mRNA_cycC - dk_mRNA_cycC_nuc...

- k_div*mRNA_cycC_nuc;

mRNA_cycC_inactive = nm_out_mRNA_cycC - activate_mRNA_cycC - dk_mRNA_cycC_inactive...

- k_div*mRNA_cycC_inactive;

mRNA_cycC_cytosol = activate_mRNA_cycC + unbind_mRNA_cycC - bind_mRNA_cycC...

- dk_mRNA_cycC_cytosol - k_div*mRNA_cycC_cytosol;

mRNA_cycC_in_use = bind_mRNA_cycC - unbind_mRNA_cycC - dk_mRNA_cycC_in_use...

- k_div*mRNA_cycC_in_use;

mRNA_cycC_trash = dk_mRNA_cycC_unspl + dk_mRNA_cycC_nuc + dk_mRNA_cycC_inactive...

+ dk_mRNA_cycC_cytosol + dk_mRNA_cycC_in_use...

- cut_mRNA_cycC - k_div*mRNA_cycC_trash;

cycC_unfold = txl_cycC - fold_cycC - u_cycC_unfold - k_div*cycC_unfold;

cycC_cytosol = fold_cycC - nm_in_cycC - u_cycC_cytosol...

- k_div*cycC_cytosol;

cycC_nuc = nm_in_cycC - bind_cycC_to_Cdk8 + unbind_cycC_from_Cdk8...

- u_cycC_nuc - k_div*cycC_nuc;

cycC_Cdk8_active = bind_cycC_to_Cdk8 - unbind_cycC_from_Cdk8...

+ activate_cycC_Cdk8_by_Cdc14...

- deactivate_cycC_Cdk8_by_mitogen...

- u_cycC_Cdk8_active - k_div*cycC_Cdk8_active;

cycC_Cdk8_inactive = deactivate_cycC_Cdk8_by_mitogen...

- activate_cycC_Cdk8_by_Cdc14...

- u_cycC_Cdk8_inactive - k_div*cycC_Cdk8_inactive;

cycC_trash = u_cycC_unfold + u_cycC_cytosol + u_cycC_nuc...

+ u_cycC_Cdk8_active + u_cycC_Cdk8_inactive - cut_cycC...

- k_div*cycC_trash;

% 32 KPC

mRNA_KPC_unspl = txs_mRNA_KPC - spl_mRNA_KPC - dk_mRNA_KPC_unspl...

- k_div*mRNA_KPC_unspl;

mRNA_KPC_nuc = spl_mRNA_KPC - nm_out_mRNA_KPC - dk_mRNA_KPC_nuc...

- k_div*mRNA_KPC_nuc;

mRNA_KPC_inactive = nm_out_mRNA_KPC - activate_mRNA_KPC - dk_mRNA_KPC_inactive...

- k_div*mRNA_KPC_inactive;

mRNA_KPC_cytosol = activate_mRNA_KPC + unbind_mRNA_KPC - bind_mRNA_KPC...

- dk_mRNA_KPC_cytosol - k_div*mRNA_KPC_cytosol;

mRNA_KPC_in_use = bind_mRNA_KPC - unbind_mRNA_KPC - dk_mRNA_KPC_in_use...

- k_div*mRNA_KPC_in_use;

mRNA_KPC_trash = dk_mRNA_KPC_unspl + dk_mRNA_KPC_nuc + dk_mRNA_KPC_inactive...

+ dk_mRNA_KPC_cytosol + dk_mRNA_KPC_in_use...

- cut_mRNA_KPC - k_div*mRNA_KPC_trash;

KPC_unfold = txl_KPC - fold_KPC - u_KPC_unfold - k_div*KPC_unfold;

KPC_in_cell = fold_KPC - activate_KPC_by_mitogen + inactivate_KPC...

- u_KPC_in_cell - k_div*KPC_in_cell;

KPC_active = activate_KPC_by_mitogen - inactivate_KPC...

- u_KPC_active - k_div*KPC_active;

KPC_trash = u_KPC_unfold + u_KPC_in_cell...

+ u_KPC_active - cut_KPC - k_div*KPC_trash;

% !!!!!!!!!!!!!!!!!!!!!!!!!!!!!!!!!!!!!!!!!!! PACK LEVELS

ydot=[

AA_in_cytosol; AA_in_p; AA_on_tRNA; ADP_in_cell; ADP_trash; ATP_in_cell;

fats_cytosol; fats_in_mem; fats_in_vesicles; H_between_mito_mems;

H_in_inner_mito_mems; junk_spl; junk_spl_mRNA; junk_spl_rRNA;

junk_spl_snRNA; mRNA_cytosol; mRNA_in_use; mRNA_nuc; mRNA_inactive;

mRNA_trash; mRNA_unspl; Na_in_cell; Na_out_cell; NT_in_cell; NT_in_RNA;

p_in_cell; p_in_use; p_trash; p_unfold; rRNA_cytosol; rRNA_in_use;

rRNA_nuc; rRNA_trash; rRNA_unspl; snRNA_in_use; snRNA_nuc; snRNA_trash;

snRNA_unspl; sugar_in_cell; tRNA_cytosol; tRNA_in_use; tRNA_nuc;

tRNA_trash;

mRNA_poly_unspl; mRNA_poly_nuc; mRNA_poly_inactive; mRNA_poly_cytosol;

mRNA_poly_in_use; mRNA_poly_trash; p_poly_unfold; p_poly_cytosol;

p_poly_nuc_inactive; p_poly_nuc_active; p_poly_in_use; p_poly_trash;

mRNA_eIF_4_unspl; mRNA_eIF_4_nuc; mRNA_eIF_4_inactive;

mRNA_eIF_4_cytosol; mRNA_eIF_4_in_use; mRNA_eIF_4_trash; p_eIF_4_unfold;

p_eIF_4_cytosol; p_eIF_4_in_use; p_eIF_4_trash;

mRNA_p27_unspl; mRNA_p27_nuc; mRNA_p27_inactive; mRNA_p27_cytosol;

mRNA_p27_in_use; mRNA_p27_trash; p27_unfold; p27_cytosol; p27_nuc;

p27_on_cyclins; p27_trash;

mRNA_Rb_unspl; mRNA_Rb_nuc; mRNA_Rb_inactive; mRNA_Rb_cytosol;

mRNA_Rb_in_use; mRNA_Rb_trash; Rb_unfold; Rb_cytosol; Rb_nuc; Rb_on_E2F;

Rb_phospho_inactive; Rb_trash;

mRNA_cycD_unspl; mRNA_cycD_nuc; mRNA_cycD_inactive; mRNA_cycD_cytosol;

mRNA_cycD_in_use; mRNA_cycD_trash; cycD_unfold; cycD_cytosol; cycD_nuc;

cycD_Cdk4or6_active; cycD_Cdk4or6_on_p27_inactive; cycD_trash;

mRNA_Cdk2_unspl; mRNA_Cdk2_nuc; mRNA_Cdk2_inactive; mRNA_Cdk2_cytosol;

mRNA_Cdk2_in_use; mRNA_Cdk2_trash; Cdk2_unfold; Cdk2_cytosol; Cdk2_nuc;

Cdk2_on_cycE; Cdk2_on_cycA; Cdk2_trash;

mRNA_cycE_unspl; mRNA_cycE_nuc; mRNA_cycE_inactive; mRNA_cycE_cytosol;

mRNA_cycE_in_use; mRNA_cycE_trash; cycE_unfold; cycE_cytosol; cycE_nuc;

cycE_Cdk2_inactive; cycE_Cdk2_active; cycE_Cdk2_on_p27_inactive;

cycE_trash;

mRNA_B_Myb_unspl; mRNA_B_Myb_nuc; mRNA_B_Myb_inactive;

mRNA_B_Myb_cytosol; mRNA_B_Myb_in_use; mRNA_B_Myb_trash; B_Myb_unfold;

B_Myb_cytosol; B_Myb_nuc; B_Myb_phospho_active; B_Myb_on_DNA;

B_Myb_trash;

mRNA_NF_Y_unspl; mRNA_NF_Y_nuc; mRNA_NF_Y_inactive; mRNA_NF_Y_cytosol;

mRNA_NF_Y_in_use; mRNA_NF_Y_trash; NF_Y_unfold; NF_Y_cytosol; NF_Y_nuc;

NF_Y_phospho_active; NF_Y_on_DNA; NF_Y_trash;

mRNA_E2F_unspl; mRNA_E2F_nuc; mRNA_E2F_inactive; mRNA_E2F_cytosol;

mRNA_E2F_in_use; mRNA_E2F_trash; E2F_unfold; E2F_cytosol; E2F_nuc;

E2F_Rb_inactive; E2F_on_DNA; E2F_trash;

mRNA_cycA_unspl; mRNA_cycA_nuc; mRNA_cycA_inactive; mRNA_cycA_cytosol;

mRNA_cycA_in_use; mRNA_cycA_trash; cycA_unfold; cycA_cytosol; cycA_nuc;

cycA_Cdk2_inactive; cycA_Cdk2_on_p27_inactive; cycA_Cdk2_active;

cycA_Cdk1_cytosol_active; cycA_Cdk1_phospho_inactive;

cycA_Cdk1_nuc_active; cycA_trash;

mRNA_SCF_unspl; mRNA_SCF_nuc; mRNA_SCF_inactive; mRNA_SCF_cytosol;

mRNA_SCF_in_use; mRNA_SCF_trash; SCF_unfold; SCF_in_cell; SCF_on_Btrc;

SCF_on_Fbw7; SCF_on_Skp2; SCF_trash;

mRNA_Skp2_unspl; mRNA_Skp2_nuc; mRNA_Skp2_inactive; mRNA_Skp2_cytosol;

mRNA_Skp2_in_use; mRNA_Skp2_trash; Skp2_unfold; Skp2_cytosol; Skp2_nuc;

Skp2_SCF_active; Skp2_trash;

mRNA_Btrc_unspl; mRNA_Btrc_nuc; mRNA_Btrc_inactive; mRNA_Btrc_cytosol;

mRNA_Btrc_in_use; mRNA_Btrc_trash; Btrc_unfold; Btrc_cytosol;

Btrc_SCF_active; Btrc_trash;

mRNA_Fbw7_unspl; mRNA_Fbw7_nuc; mRNA_Fbw7_inactive; mRNA_Fbw7_cytosol;

mRNA_Fbw7_in_use; mRNA_Fbw7_trash; Fbw7_unfold; Fbw7_cytosol; Fbw7_nuc;

Fbw7_SCF_active; Fbw7_trash;

mRNA_TF_grow_unspl; mRNA_TF_grow_inactive; mRNA_TF_grow_cytosol;

mRNA_TF_grow_nuc; mRNA_TF_grow_in_use; mRNA_TF_grow_trash;

TF_grow_unfold; TF_grow_cytosol; TF_grow_nuc; TF_grow_on_DNA;

TF_grow_trash;

NT_in_DNA;

mRNA_RC_unspl; mRNA_RC_nuc; mRNA_RC_inactive; mRNA_RC_cytosol;

mRNA_RC_in_use; mRNA_RC_trash; RC_unfold; RC_cytosol; RC_nuc; RC_on_DNA;

RC_licensed; RC_traversed_by_DNA_poly; RC_trash; RC_count;

mRNA_DNA_poly_unspl; mRNA_DNA_poly_nuc; mRNA_DNA_poly_inactive;

mRNA_DNA_poly_cytosol; mRNA_DNA_poly_in_use; mRNA_DNA_poly_trash;

DNA_poly_unfold; DNA_poly_cytosol; DNA_poly_nuc; DNA_poly_on_DNA;

DNA_poly_trash;

mRNA_Wee1_unspl; mRNA_Wee1_nuc; mRNA_Wee1_inactive; mRNA_Wee1_cytosol;

mRNA_Wee1_in_use; mRNA_Wee1_trash; Wee1_unfold; Wee1_cytosol_active;

Wee1_phospho_inactive; Wee1_trash;

mRNA_cycB_unspl; mRNA_cycB_nuc; mRNA_cycB_inactive; mRNA_cycB_cytosol;

mRNA_cycB_in_use; mRNA_cycB_trash; cycB_unfold; cycB_cytosol;

cycB_Cdk1_cytosol_active; cycB_Cdk1_phospho_inactive;

cycB_Cdk1_nuc_active; cycB_trash;

mRNA_Cdk1_unspl; mRNA_Cdk1_nuc; mRNA_Cdk1_inactive; mRNA_Cdk1_cytosol;

mRNA_Cdk1_in_use; mRNA_Cdk1_trash; Cdk1_unfold; Cdk1_inactive;

Cdk1_in_cell; Cdk1_on_cycB; Cdk1_on_cycA; Cdk1_trash;

mRNA_Cdc25C_unspl; mRNA_Cdc25C_nuc; mRNA_Cdc25C_inactive;

mRNA_Cdc25C_cytosol; mRNA_Cdc25C_in_use; mRNA_Cdc25C_trash;

Cdc25C_unfold; Cdc25C_cytosol_inactive; Cdc25C_cytosol_phospho_active;

Cdc25C_nuc_phospho_active; Cdc25C_trash;

mRNA_Plk1_unspl; mRNA_Plk1_nuc; mRNA_Plk1_inactive; mRNA_Plk1_cytosol;

mRNA_Plk1_in_use; mRNA_Plk1_trash; Plk1_unfold; Plk1_cytosol_inactive;

Plk1_cytosol_phospho_active; Plk1_nuc_phospho_active; Plk1_trash;

mRNA_Emi1_unspl; mRNA_Emi1_nuc; mRNA_Emi1_inactive; mRNA_Emi1_cytosol;

mRNA_Emi1_in_use; mRNA_Emi1_trash; Emi1_unfold; Emi1_in_cell;

Emi1_on_Cdh1; Emi1_on_Cdc20; Emi1_phospho_inactive; Emi1_trash;

mRNA_APC_unspl; mRNA_APC_nuc; mRNA_APC_inactive; mRNA_APC_cytosol;

mRNA_APC_in_use; mRNA_APC_trash; APC_unfold; APC_in_cell; APC_on_Cdh1;

APC_on_Cdc20; APC_trash;

mRNA_Cdh1_unspl; mRNA_Cdh1_nuc; mRNA_Cdh1_inactive; mRNA_Cdh1_cytosol;

mRNA_Cdh1_in_use; mRNA_Cdh1_trash; Cdh1_unfold; Cdh1_in_cell;

Cdh1_Emi1_inactive; Cdh1_phospho_inactive; Cdh1_APC_active; Cdh1_trash;

mRNA_Cdc20_unspl; mRNA_Cdc20_nuc; mRNA_Cdc20_inactive;

mRNA_Cdc20_cytosol; mRNA_Cdc20_in_use; mRNA_Cdc20_trash; Cdc20_unfold;

Cdc20_cytosol; Cdc20_nuc; Cdc20_Emi1_inactive; Cdc20_APC_inactive;

Cdc20_APC_active; Cdc20_trash;

mRNA_Cdc14_unspl; mRNA_Cdc14_nuc; mRNA_Cdc14_inactive;

mRNA_Cdc14_cytosol; mRNA_Cdc14_in_use; mRNA_Cdc14_trash; Cdc14_unfold;

Cdc14_cytosol; Cdc14_nuc_inactive; Cdc14_inhibited;

Cdc14_phospho_active; Cdc14_trash;

mRNA_Cdc25A_unspl; mRNA_Cdc25A_nuc; mRNA_Cdc25A_inactive;

mRNA_Cdc25A_cytosol; mRNA_Cdc25A_in_use; mRNA_Cdc25A_trash;

Cdc25A_unfold; Cdc25A_cytosol; Cdc25A_nuc_inactive;

Cdc25A_phospho_active; Cdc25A_trash;

mRNA_Cdc25B_unspl; mRNA_Cdc25B_nuc; mRNA_Cdc25B_inactive;

mRNA_Cdc25B_cytosol; mRNA_Cdc25B_in_use; mRNA_Cdc25B_trash;

Cdc25B_unfold; Cdc25B_cytosol_inactive; Cdc25B_cytosol_phospho_active;

Cdc25B_trash;

mRNA_Securin_unspl; mRNA_Securin_nuc; mRNA_Securin_inactive;

mRNA_Securin_cytosol; mRNA_Securin_in_use; mRNA_Securin_trash;

Securin_unfold; Securin_cytosol; Securin_nuc; Securin_on_chromo;

Securin_trash;

mRNA_cycC_unspl; mRNA_cycC_nuc; mRNA_cycC_inactive; mRNA_cycC_cytosol;

mRNA_cycC_in_use; mRNA_cycC_trash; cycC_unfold; cycC_cytosol; cycC_nuc;

cycC_Cdk8_active; cycC_Cdk8_inactive; cycC_trash;

mRNA_KPC_unspl; mRNA_KPC_nuc; mRNA_KPC_inactive; mRNA_KPC_cytosol;

mRNA_KPC_in_use; mRNA_KPC_trash; KPC_unfold; KPC_in_cell; KPC_active;

KPC_trash

];

# celldyneqns.m

celldyneqns.m is identical to cellsseqns.m except for the following…

On line 2, change…

< function ydot=cellsseqns(t,y)

---

> function ydot=celldyneqns(t,y)

On line 7, change…

< [z,y0]=getssconst;

---

> z=getconst;

On about line 347, insert…

> fid0=fopen('steadystate.bin','r');

> y0=fread(fid0,inf,'double');

> fclose(fid0);

>

On about line 1213, change…

< size_ratio = 1;

< dividing = 0;

< k_div = 0;

< % size_ratio = p_in_use/i_p_in_use;

< % if(t>2.0E4)

< % mitogen_stimulation = 1;

< % adhesion_factor = 1;

< % end

---

> % size_ratio = 1;

> % dividing = 0;

> % k_div = 0;

> size_ratio = p_in_use/i_p_in_use;

> if(t>2.0E4)

> mitogen_stimulation = 1;

> adhesion_factor = 1;

> end

On about line 3827, change…

< % [tprev,dividing,k_div] = getdividing...

< % (t,bind_Cdh1_to_APC,unbind_Cdh1_APC,...

< % u_Cdh1_APC_active,i_NT_in_DNA,NT_in_DNA);

< % if(dividing == 1)

< % pm_in_NT = 0;

< % end

---

> [tprev,dividing,k_div] = getdividing...

> (t,bind_Cdh1_to_APC,unbind_Cdh1_APC,...

> u_Cdh1_APC_active,i_NT_in_DNA,NT_in_DNA);

> if(dividing == 1)

> pm_in_NT = 0;

> end

# getssconst.m

function [z,y0]=getssconst

% !!!!!!!!!!!!!!!!!!!!!!!!!!!!!!!!!!!!!!!!!!!!!!!! CONSTANTS

z=getconst;

% !!!!!!!!!!!!!!!!!!!!!!!!!!!!!!!!!!!!!!!!! UNPACK CONSTANTS

ATP_AA = z(1);

ATP_eIF_4 = z(2);

ATP_fat = z(3);

ATP_fold = z(4);

ATP_glycolysis = z(5);

ATP_glycosolation = z(6);

ATP_H = z(7);

ATP_mem_transfer = z(8);

ATP_pm_transfer = z(9);

ATP_Na = z(10);

ATP_NT = z(11);

ATP_NT_out_nm = z(12);

ATP_polymerization = z(13);

ATP_proteasome = z(14);

ATP_respiration = z(15);

ATP_spl = z(16);

ATP_tRNA = z(17);

ATP_u = z(18);

ATP_vesicle = z(19);

exon_mRNA = z(20);

exon_rRNA = z(21);

exon_snRNA = z(22);

frac_p_cytosol = z(23);

frac_p_ER = z(24);

frac_p_lysosome = z(25);

frac_p_mitochondria = z(26);

frac_p_nuc = z(27);

frac_polyI = z(28);

frac_polyII = z(29);

frac_polyII_snRNA = z(30);

frac_polyIII = z(31);

i_eIF_4 = z(32);

i_ER_p = z(33);

i_ER_RNA = z(34);

i_poly_all = z(35);

x_mRNA = z(36);

x_mRNA_unspl = z(37);

x_p = z(38);

x_rRNA = z(39);

x_rRNA_unspl = z(40);

x_snRNA = z(41);

x_snRNA_unspl = z(42);

x_tRNA = z(43);

x_vesicle = z(44);

k_ATP_synthase = z(45);

k_bind_p = z(46);

k_bind_snRNA = z(47);

k_cut_p = z(48);

k_cut_RNA = z(49);

k_cut_mRNA = z(50);

k_cut_rRNA = z(51);

k_cut_snRNA = z(52);

k_cut_tRNA = z(53);

k_cut_spljunk_mRNA = z(54);

k_cut_spljunk_rRNA = z(55);

k_cut_spljunk_snRNA = z(56);

k_dk_AA = z(57);

k_dk_ADP = z(58);

k_dk_fat = z(59);

k_dk_NT = z(60);

k_dk_p = z(61);

k_dk_mRNA = z(62);

k_dk_rRNA = z(63);

k_dk_snRNA = z(64);

k_dk_tRNA = z(65);

k_bind_rRNA_mRNA = z(66);

k_ER_p_constant = z(67);

k_ER_RNA_constant = z(68);

k_fold_p = z(69);

k_grow_H = z(70);

k_grow_Na = z(71);

k_make_vesicle = z(72);

k_H_pump = z(73);

k_Na_pump_out = z(74);

k_Na_return = z(75);

k_nm_mRNA = z(76);

k_nm_rRNA = z(77);

k_nm_tRNA = z(78);

k_pm_in_AA = z(79);

k_pm_in_ADP = z(80);

k_pm_in_NT = z(81);

k_pm_in_fats = z(82);

k_pm_in_sugar = z(83);

k_restructure = z(84);

k_spl = z(85);

k_tRNA_AA_binding = z(86);

k_unbind_fats = z(87);

k_bind_fats = z(88);

rRNA_spacing_on_mRNA = z(89);

k_bind_p_poly_to_DNA = z(90);

k_unbind_p_poly_from_DNA_by_APC_Cdc20 = z(91);

k_nm_in_p = z(92);

poly_loci_frac = z(93);

RNA_poly_loci_constitutive = z(94);

RNA_poly_active_constitutive = z(95);

k_activate_p_poly_by_mitogen = z(96);

k_deactivate_p_poly_by_cycC_Cdk8 = z(97);

k_activate_mRNA = z(98);

eIF_4_loci_frac = z(99);

p27_loci_frac = z(100);

k_u_p27_by_SCF_Skp2_fast = z(101);

k_u_p27_by_SCF_Skp2_slow = z(102);

k_u_p27_by_APC_Cdc20 = z(103);

k_u_p27_by_KPC = z(104);

Rb_loci_frac = z(105);

k_bind_Rb_to_E2F = z(106);

k_unbind_Rb_E2F = z(107);

k_phospho_Rb_by_Cdk = z(108);

k_unphospho_Rb = z(109);

k_dephospho_Rb_by_Cdc14 = z(110);

mitogen_stimulation = z(111);

cycD_loci_frac = z(112);

cycD_constitutive = z(113);

k_bind_cycD_to_Cdk4or6 = z(114);

k_unbind_cycD_Cdk4or6 = z(115);

k_bind_cycD_Cdk4or6_to_p27 = z(116);

k_unbind_cycD_Cdk4or6_p27 = z(117);

k_u_cycD_by_SCF_Skp2 = z(118);

k_u_cycD_by_APC_Cdc20 = z(119);

Cdk2_loci_frac = z(120);

Cdk2_constituitive = z(121);

cycE_loci_frac = z(122);

k_bind_cycE_to_Cdk2 = z(123);

k_unbind_cycE_Cdk2 = z(124);

k_bind_cycE_Cdk2_to_p27 = z(125);

k_unbind_cycE_Cdk2_p27 = z(126);

k_phospho_cycE_Cdk2 = z(127);

k_dephospho_cycE_Cdk2 = z(128);

k_dephospho_cycE_Cdk2_by_Cdc25A = z(129);

k_u_cycE_by_SCF_Skp2 = z(130);

k_u_cycE_by_SCF_Fbw7 = z(131);

B_Myb_loci_frac = z(132);

k_phospho_B_Myb_by_cyc_Cdk = z(133);

k_unphospho_B_Myb = z(134);

sat_B_Myb_on_DNA = z(135);

k_bind_B_Myb_to_DNA = z(136);

k_unbind_B_Myb_from_DNA = z(137);

k_u_B_Myb_by_SCF_Skp2 = z(138);

NF_Y_loci_frac = z(139);

NF_Y_constitutive = z(140);

k_phospho_NF_Y_by_cyc_Cdk = z(141);

k_unphospho_NF_Y = z(142);

k_dephospho_NF_Y_by_Cdc14 = z(143);

sat_NF_Y_on_DNA = z(144);

k_bind_NF_Y_to_DNA = z(145);

k_unbind_NF_Y_from_DNA = z(146);

E2F_loci_frac = z(147);

E2F_constituitive = z(148);

sat_E2F_on_DNA = z(149);

k_bind_E2F_to_DNA = z(150);

k_unbind_E2F_DNA = z(151);

k_E2F_phospho_by_cycA_Cdk2 = z(152);

k_E2F_phospho_by_cycA_Cdk1 = z(153);

k_E2F_phospho_by_cycB_Cdk1 = z(154);

k_E2F_phospho_by_cycD_Cdk4or6 = z(155);

k_u_E2F_by_SCF_Skp2 = z(156);

k_u_E2F_by_APC_Cdc20 = z(157);

adhesion_factor = z(158);

cycA_loci_frac = z(159);

k_bind_cycA_to_Cdk2 = z(160);

k_unbind_cycA_Cdk2 = z(161);

k_dephospho_cycA_Cdk2_by_Cdc25A = z(162);

k_phospho_cycA_Cdk2 = z(163);

k_bind_cycA_Cdk2_to_p27 = z(164);

k_unbind_cycA_Cdk2_p27 = z(165);

k_bind_cycA_to_Cdk1 = z(166);

k_unbind_cycA_Cdk1 = z(167);

k_phospho_cycA_Cdk1_by_Wee1 = z(168);

k_phospho_cycA_Cdk1_by_phospho_Wee1 = z(169);

k_unphospho_cycA_Cdk1_from_Wee1 = z(170);

k_dephospho_cycA_Cdk1_by_Cdc25A = z(171);

k_dephospho_cycA_Cdk1_by_Cdc25B = z(172);

k_dephospho_cycA_Cdk1_by_Cdc25C = z(173);

k_nm_in_cycA_Cdk1_by_Plk1 = z(174);

k_nm_out_cycA_Cdk1 = z(175);

k_u_cycA_by_SCF_Skp2 = z(176);

k_u_cycA_by_APC_Cdc20 = z(177);

k_u_cycA_by_APC_Cdh1 = z(178);

SCF_loci_frac = z(179);

k_u_SCF_in_cell_by_APC_Cdh1 = z(180);

k_u_SCF_active_by_APC_Cdh1 = z(181);

Skp2_loci_frac = z(182);

k_bind_Skp2_to_SCF = z(183);

k_unbind_Skp2_SCF = z(184);

k_u_Skp2_by_APC_Cdh1 = z(185);

k_u_auto_Skp2 = z(186);

Btrc_loci_frac = z(187);

k_bind_Btrc_to_SCF = z(188);

k_unbind_Btrc_SCF = z(189);

k_u_Btrc_by_APC_Cdh1 = z(190);

k_u_auto_Btrc = z(191);

Fbw7_loci_frac = z(192);

k_bind_Fbw7_to_SCF = z(193);

k_unbind_Fbw7_SCF = z(194);

k_u_auto_Fbw7 = z(195);

TF_grow_loci_frac = z(196);

TF_grow_txs_by_mitogen = z(197);

k_TF_grow_txs_by_Skp2 = z(198);

sat_TF_grow_on_DNA = z(199);

k_bind_TF_grow_to_DNA = z(200);

k_unbind_TF_grow_DNA = z(201);

k_u_TF_grow_by_SCF_Fbw7 = z(202);

k_u_TF_grow_by_SCF_Skp2 = z(203);

ATP_DNA_repl = z(204);

ATP_NT_txs = z(205);

x_DNA = z(206);

i_ER_DNA = z(207);

k_ER_DNA_constant = z(208);

k_div_DNA_factor = z(209);

x_RC = z(210);

x_RC_frag = z(211);

x_NT_RC_frag = z(212);

x_DNA_poly_per_RC = z(213);

RC_loci_frac = z(214);

k_bind_RC_to_DNA = z(215);

k_unbind_RC_DNA = z(216);

k_phospho_RC_by_cycD_Cdk4or6 = z(217);

k_phospho_RC_by_cycD_Cdk4or6_on_p27 = z(218);

k_phospho_RC_by_cycE_Cdk2 = z(219);

k_phospho_RC_by_cycA_Cdk2 = z(220);

k_u_RC_by_SCF_Fbw7 = z(221);

k_u_RC_by_SCF_Skp2 = z(222);

k_u_RC_by_APC_Cdh1 = z(223);

k_clear_RC_count = z(224);

DNA_poly_loci_frac = z(225);

k_bind_DNA_poly_to_DNA = z(226);

sat_DNA_poly = z(227);

Wee1_loci_frac = z(228);

k_phospho_Wee1_by_cycA_Cdk2 = z(229);

k_phospho_Wee1_by_cycA_Cdk1 = z(230);

k_phospho_Wee1_by_cycB_Cdk1 = z(231);

k_phospho_Wee1_by_Plk1 = z(232);

k_unphospho_Wee1 = z(233);

k_dephospho_Wee1_by_Cdc14 = z(234);

k_u_Wee1_by_SCF_Btrc = z(235);

k_u_Wee1_by_SCF_Skp2_test = z(236);

cycB_loci_frac = z(237);

k_bind_cycB_to_Cdk1 = z(238);

k_unbind_cycB_Cdk1 = z(239);

k_phospho_cycB_Cdk1_by_Wee1 = z(240);

k_phospho_cycB_Cdk1_by_phospho_Wee1 = z(241);

k_unphospho_cycB_Cdk1_from_Wee1 = z(242);

k_dephospho_cycB_Cdk1_by_Cdc25A = z(243);

k_dephospho_cycB_Cdk1_by_Cdc25B = z(244);

k_dephospho_cycB_Cdk1_by_Cdc25C = z(245);

k_nm_in_cycB_Cdk1_by_Plk1 = z(246);

k_nm_out_cycB_Cdk1 = z(247);

k_u_cycB_by_APC_Cdc20 = z(248);

k_u_cycB_by_APC_Cdh1 = z(249);

Cdk1_loci_frac = z(250);

Cdk1_constitutive = z(251);

k_activate_Cdk1_by_B_Myb_and_NF_Y = z(252);

k_deactivate_Cdk1 = z(253);

k_dephospho_Cdk1_by_Cdc14 = z(254);

Cdc25C_loci_frac = z(255);

k_phospho_Cdc25C_by_cycA_Cdk1 = z(256);

k_phospho_Cdc25C_by_cycB_Cdk1 = z(257);

k_phospho_Cdc25C_by_Plk1 = z(258);

k_dephospho_Cdc25C = z(259);

k_unphospho_Cdc25C = z(260);

k_nm_in_Cdc25C = z(261);

k_nm_out_Cdc25C = z(262);

k_dephospho_Cdc25C_by_Cdc14 = z(263);

Plk1_loci_frac = z(264);

k_phospho_Plk1_by_cycA_Cdk1 = z(265);

k_phospho_Plk1_by_cycB_Cdk1 = z(266);

k_unphospho_Plk1 = z(267);

k_nm_in_Plk1 = z(268);

k_u_Plk1_by_APC_Cdh1 = z(269);

Emi1_loci_frac = z(270);

k_bind_Emi1_to_Cdh1 = z(271);

k_unbind_Emi1_Cdh1 = z(272);

k_bind_Emi1_to_Cdc20 = z(273);

k_unbind_Emi1_Cdc20 = z(274);

k_phospho_Emi1_by_cycA_Cdk1 = z(275);

k_phospho_Emi1_by_cycB_Cdk1 = z(276);

k_u_Emi1_by_SCF_Btrc = z(277);

k_u_Emi1_by_SCF_Skp2_test = z(278);

APC_loci_frac = z(279);

Cdh1_loci_frac = z(280);

k_phospho_Cdh1_by_cycD_Cdk4or6 = z(281);

k_phospho_Cdh1_by_cycD_Cdk4or6_on_p27 = z(282);

k_phospho_Cdh1_by_cycE_Cdk2 = z(283);

k_phospho_Cdh1_by_cycA_Cdk2 = z(284);

k_phospho_Cdh1_by_Cdk1 = z(285);

k_dephospho_Cdh1_by_Cdc14 = z(286);

k_bind_Cdh1_to_APC = z(287);

k_unbind_Cdh1_APC = z(288);

k_u_auto_Cdh1_APC_active = z(289);

Cdc20_loci_frac = z(290);

k_bind_Cdc20_to_APC = z(291);

k_unbind_Cdc20_APC = z(292);

k_phospho_Cdc20_by_Cdk1 = z(293);

k_unphospho_Cdc20_APC_active = z(294);

k_dephospho_Cdc20_APC_by_Cdc14 = z(295);

k_u_Cdc20_by_APC_Cdh1 = z(296);

Cdc14_loci_frac = z(297);

k_phospho_Cdc14_by_Plk1 = z(298);

k_unphospho_Cdc14 = z(299);

k_inhibit_Cdc14_by_Securin = z(300);

k_uninhibit_Cdc14 = z(301);

Cdc25A_loci_frac = z(302);

Cdc25A_constitutive = z(303);

k_phospho_Cdc25A_by_Cdk2 = z(304);

k_phospho_Cdc25A_by_cycA_Cdk1 = z(305);

k_phospho_Cdc25A_by_cycB_Cdk1 = z(306);

k_dephospho_Cdc25A = z(307);

k_dephospho_Cdc25A_by_Cdc14 = z(308);

k_u_Cdc25A_by_APC_Cdh1 = z(309);

k_u_Cdc25A_by_SCF_Btrc = z(310);

Cdc25B_loci_frac = z(311);

k_phospho_Cdc25B_by_Cdk2 = z(312);

k_phospho_Cdc25B_by_cycA_Cdk1 = z(313);

k_phospho_Cdc25B_by_cycB_Cdk1 = z(314);

k_phospho_Cdc25B_by_TF_grow = z(315);

k_phospho_Cdc25B_by_Plk1 = z(316);

k_dephospho_Cdc25B = z(317);

k_dephospho_Cdc25B_by_Cdc14 = z(318);

k_u_Cdc25B_by_SCF_Btrc = z(319);

k_u_Cdc25B_by_APC_Cdh1 = z(320);

k_u_Cdc25B_by_APC_Cdh1 = z(321);

Securin_loci_frac = z(322);

k_bind_Securin_to_chromo = z(323);

k_u_Securin_by_APC_Cdc20 = z(324);

cycC_loci_frac = z(325);

k_bind_cycC_to_Cdk8 = z(326);

k_unbind_cycC_from_Cdk8 = z(327);

k_deactivate_cycC_Cdk8_by_mitogen = z(328);

k_activate_cycC_Cdk8_by_Cdc14 = z(329);

KPC_loci_frac = z(330);

k_activate_KPC_by_mitogen = z(331);

k_inactivate_KPC = z(332);

% !!!!!!!!!!!!!!!!!!!!!!!!!! STEADY-STATE INITIAL CONDITIONS

% base quantities

i_AA_in_cytosol = 5.8E10;

i_AA_in_p = 400*(6.4E4 + 1E10 + 1.6E7 + 3.2E4);

i_AA_on_tRNA = 2.5E6;

i_ADP_in_cell = 1E8;

i_ADP_trash = 2;

i_ATP_in_cell = 1.0E9;

i_fats_cytosol = 1E8;

i_fats_in_mem = 2E10;

i_fats_in_vesicles = 2E9;

% i_H_between_mito_mems = 1.2E5;

% i_H_in_inner_mito_mems = 1.2E4;

i_H_between_mito_mems = 1.2E4;

i_H_in_inner_mito_mems = 1.2E3;

i_junk_spl = 3.0E4;

i_junk_spl_mRNA = 3.2E4;

i_junk_spl_rRNA = 1.3E4;

i_junk_spl_snRNA = 20;

% i_mRNA_cytosol = 2.0E4;

% i_mRNA_in_use = 5.2E3;

i_mRNA_cytosol = 1.0E3;

i_mRNA_in_use = 1.1E4;

i_mRNA_nuc = 5.9E4;

i_mRNA_inactive = 10;

i_mRNA_trash = 8.7E3;

i_mRNA_unspl = 4;

i_Na_in_cell = 2.5E10;

i_Na_out_cell = 3.75E11;

i_NT_in_cell = 2.00E10;

i_NT_in_RNA = 1500*(2.0E4 + 5.2E3 + 5.9E4 + 8.7E3) + 7E3*4 ...

+ 7000*(7.1E4 + 6.3E4 + 6.7E3 + 1.5E4) + 1.3E4*1 ...

+ 1250*(1.0E4 + 400 + 20) + 2.5E3*1 ...

+ 80*(2.0E5 + 2.5E6 + 1.2E3 + 2.1E3)...

+ 3.0E4 + 3.2E4 + 1.3E4 + 20;

i_p_in_cell = 6.4E4;

i_p_in_use = 1E10;

i_p_trash = 1.6E7;

i_p_unfold = 3.2E4;

i_rRNA_cytosol = 7.1E4;

i_rRNA_in_use = 6.3E4;

i_rRNA_nuc = 6.7E3;

i_rRNA_trash = 1.5E4;

i_rRNA_unspl = 1;

i_snRNA_in_use = 1.0E4;

i_snRNA_nuc = 400;

i_snRNA_trash = 20;

i_snRNA_unspl = 1;

i_sugar_in_cell = 1.0E9;

i_tRNA_cytosol = 2.0E5;

i_tRNA_in_use = i_AA_on_tRNA;

i_tRNA_nuc = 1.2E3;

i_tRNA_trash = 2.1E3;

% i_AA_in_p = x_p*(i_p_in_cell+i_p_in_use+i_p_trash+i_p_unfold);

% i_NT_in_RNA = x_mRNA*(i_mRNA_cytosol + i_mRNA_in_use + i_mRNA_nuc...

% + i_mRNA_trash) + x_mRNA_unspl*i_mRNA_unspl...

% + x_rRNA*(i_rRNA_cytosol + i_rRNA_in_use + i_rRNA_nuc...

% + i_rRNA_trash) + x_rRNA_unspl*i_rRNA_unspl...

% + x_snRNA*(i_snRNA_in_use + i_snRNA_nuc + i_snRNA_trash)...

% + x_snRNA_unspl*i_snRNA_unspl...

% + x_tRNA*(i_tRNA_cytosol + i_tRNA_in_use + i_tRNA_nuc...

% + i_tRNA_trash)...

% + i_junk_spl + i_junk_spl_mRNA + i_junk_spl_rRNA...

% + i_junk_spl_snRNA;

% RNA polymerase

i_mRNA_poly_unspl = 1;

i_mRNA_poly_nuc = 1;

i_mRNA_poly_inactive = 1;

i_mRNA_poly_cytosol = 1;

i_mRNA_poly_in_use = 1;

i_mRNA_poly_trash = 1;

i_p_poly_unfold = 1;

i_p_poly_cytosol = 2;

i_p_poly_nuc_inactive = 2.9E4;

i_p_poly_nuc_active = 3.0E2;

i_p_poly_in_use = 2.0E3;

i_p_poly_trash = 50;

% eIF_4... y0(55)--y0(64)

i_mRNA_eIF_4_unspl = 1;

i_mRNA_eIF_4_nuc = 6;

i_mRNA_eIF_4_inactive = 1;

i_mRNA_eIF_4_cytosol = 2;

i_mRNA_eIF_4_in_use = 1;

i_mRNA_eIF_4_trash = 1;

i_p_eIF_4_unfold = 3;

i_p_eIF_4_cytosol = 8.2E5;

i_p_eIF_4_in_use = i_mRNA_cytosol + i_mRNA_in_use;

i_p_eIF_4_trash = 1.3E3;

%%%%%%%%%%%%%%%%%%%%%%%%%%%%%%%%%%%%%%%%%%%%%%%%%% ADD CELL-CYCLE PROTEINS

% 1 p27... y0(65)--y0(75)

i_mRNA_p27_unspl = 1;

i_mRNA_p27_nuc = 1;

i_mRNA_p27_inactive = 1;

i_mRNA_p27_cytosol = 1;

i_mRNA_p27_in_use = 1;

i_mRNA_p27_trash = 1;

i_p27_unfold = 1E1;

i_p27_cytosol = 1E2;

i_p27_nuc = 1E6;

% i_p27_on_cyclins = i_cycD_Cdk4or6_on_p27_inactive + i_cycE_Cdk2_on_p27_inactive...

% + i_cycA_Cdk2_on_p27_inactive;

i_p27_on_cyclins = 0E-3;

i_p27_trash = 1E2;

% 2 Rb

i_mRNA_Rb_unspl = 1;

i_mRNA_Rb_nuc = 1;

i_mRNA_Rb_inactive = 1;

i_mRNA_Rb_cytosol = 1;

i_mRNA_Rb_in_use = 1;

i_mRNA_Rb_trash = 1;

i_Rb_unfold = 1E1;

i_Rb_cytosol = 1E2;

i_Rb_nuc = 1E6;

% i_Rb_on_E2F = i_E2F_Rb_inactive;

i_Rb_on_E2F = 1.0E3;

i_Rb_phospho_inactive = 1E2;

i_Rb_trash = 1E2;

% 3 cycD

i_mRNA_cycD_unspl = 0E-6;

i_mRNA_cycD_nuc = 0E-6;

i_mRNA_cycD_inactive = 0E-6;

i_mRNA_cycD_cytosol = 0E-6;

i_mRNA_cycD_in_use = 0E-6;

i_mRNA_cycD_trash = 0E-6;

i_cycD_unfold = 0E-3;

i_cycD_cytosol = 0E-3;

i_cycD_nuc = 0E-3;

i_cycD_Cdk4or6_active = 0E-3;

i_cycD_Cdk4or6_on_p27_inactive = 0E-3;

i_cycD_trash = 0E-3;

% 4 Cdk2

i_mRNA_Cdk2_unspl = 0E-6;

i_mRNA_Cdk2_nuc = 0E-6;

i_mRNA_Cdk2_inactive = 0E-6;

i_mRNA_Cdk2_cytosol = 0E-6;

i_mRNA_Cdk2_in_use = 0E-6;

i_mRNA_Cdk2_trash = 0E-6;

i_Cdk2_unfold = 1E1;

i_Cdk2_cytosol = 1E2;

i_Cdk2_nuc = 1E5;

% i_Cdk2_on_cycE = i_cycE_Cdk2_inactive + i_cycE_Cdk2_active...

% + i_cycE_Cdk2_on_p27_inactive;

i_Cdk2_on_cycE = 0E-3;

% i_Cdk2_on_cycA = i_cycA_Cdk2_inactive + i_cycA_Cdk2_active...

% + i_cycA_Cdk2_on_p27_inactive;

i_Cdk2_on_cycA = 0E-3;

i_Cdk2_trash = 1E2;

% 5 cycE

i_mRNA_cycE_unspl = 0E-6;

i_mRNA_cycE_nuc = 0E-6;

i_mRNA_cycE_inactive = 0E-6;

i_mRNA_cycE_cytosol = 0E-6;

i_mRNA_cycE_in_use = 0E-6;

i_mRNA_cycE_trash = 0E-6;

i_cycE_unfold = 0E-3;

i_cycE_cytosol = 0E-3;

i_cycE_nuc = 0E-3;

i_cycE_Cdk2_inactive = 0E-3;

i_cycE_Cdk2_active = 0E-6;

i_cycE_Cdk2_on_p27_inactive = 0E-6;

i_cycE_trash = 0E-3;

% 6 B-Myb

i_mRNA_B_Myb_unspl = 0E-6;

i_mRNA_B_Myb_nuc = 0E-6;

i_mRNA_B_Myb_inactive = 0E-6;

i_mRNA_B_Myb_cytosol = 0E-6;

i_mRNA_B_Myb_in_use = 0E-6;

i_mRNA_B_Myb_trash = 0E-6;

i_B_Myb_unfold = 0E-3;

i_B_Myb_cytosol = 0E-3;

i_B_Myb_nuc = 0E-3;

i_B_Myb_phospho_active = 0E-3;

i_B_Myb_on_DNA = 0E-3;

i_B_Myb_trash = 0E-3;

% 7 NF-Y

i_mRNA_NF_Y_unspl = 0E-6;

i_mRNA_NF_Y_nuc = 0E-6;

i_mRNA_NF_Y_inactive = 0E-6;

i_mRNA_NF_Y_cytosol = 0E-6;

i_mRNA_NF_Y_in_use = 0E-6;

i_mRNA_NF_Y_trash = 0E-6;

i_NF_Y_unfold = 0E-3;

i_NF_Y_cytosol = 0E-3;

i_NF_Y_nuc = 0E-3;

i_NF_Y_phospho_active = 0E-3;

i_NF_Y_on_DNA = 0E-3;

i_NF_Y_trash = 0E-3;

% 8 E2F

i_mRNA_E2F_unspl = 0E-6;

i_mRNA_E2F_nuc = 0E-6;

i_mRNA_E2F_inactive = 0E-6;

i_mRNA_E2F_cytosol = 0E-6;

i_mRNA_E2F_in_use = 0E-6;

i_mRNA_E2F_trash = 0E-6;

i_E2F_unfold = 0E-3;

i_E2F_cytosol = 0E-3;

i_E2F_nuc = 0E-3;

% i_E2F_Rb_inactive = i_Rb_on_E2F;

i_E2F_Rb_inactive = 1.0E3;

i_E2F_on_DNA = 0E-9;

i_E2F_trash = 0E-3;

% 9 cycA

i_mRNA_cycA_unspl = 0E-6;

i_mRNA_cycA_nuc = 0E-6;

i_mRNA_cycA_inactive = 0E-6;

i_mRNA_cycA_cytosol = 0E-6;

i_mRNA_cycA_in_use = 0E-6;

i_mRNA_cycA_trash = 0E-6;

i_cycA_unfold = 0E-3;

i_cycA_cytosol = 0E-3;

i_cycA_nuc = 0E-3;

i_cycA_Cdk2_inactive = 0E-3;

i_cycA_Cdk2_on_p27_inactive = 0E-3;

i_cycA_Cdk2_active = 0E-3;

i_cycA_Cdk1_cytosol_active = 0E-3;

i_cycA_Cdk1_phospho_inactive = 0E-3;

i_cycA_Cdk1_nuc_active = 0E-3;

i_cycA_trash = 0E-3;

% 10 SCF

i_mRNA_SCF_unspl = 0E-6;

i_mRNA_SCF_nuc = 0E-6;

i_mRNA_SCF_inactive = 0E-6;

i_mRNA_SCF_cytosol = 0E-6;

i_mRNA_SCF_in_use = 0E-6;

i_mRNA_SCF_trash = 0E-6;

i_SCF_unfold = 0E-3;

% i_SCF_cytosol = 0E-3;

i_SCF_in_cell = 0E-3;

i_SCF_on_Btrc = 0E-3;

% i_SCF_nuc = 0E-3;

i_SCF_on_Fbw7 = 0E-3;

i_SCF_on_Skp2 = 0E-3;

i_SCF_trash = 0E-3;

% 11 Skp2

i_mRNA_Skp2_unspl = 0E-6;

i_mRNA_Skp2_nuc = 0E-6;

i_mRNA_Skp2_inactive = 0E-6;

i_mRNA_Skp2_cytosol = 0E-6;

i_mRNA_Skp2_in_use = 0E-6;

i_mRNA_Skp2_trash = 0E-6;

i_Skp2_unfold = 0E-3;

i_Skp2_cytosol = 0E-3;

i_Skp2_nuc = 0E-3;

% i_Skp2_SCF_active = i_SCF_on_Skp2;

i_Skp2_SCF_active = 0E-3;

i_Skp2_trash = 0E-3;

% 12 Btrc

i_mRNA_Btrc_unspl = 0E-6;

i_mRNA_Btrc_nuc = 0E-6;

i_mRNA_Btrc_inactive = 0E-6;

i_mRNA_Btrc_cytosol = 0E-6;

i_mRNA_Btrc_in_use = 0E-6;

i_mRNA_Btrc_trash = 0E-6;

i_Btrc_unfold = 0E-3;

i_Btrc_cytosol = 0E-3;

% i_Btrc_SCF_active = i_SCF_on_Btrc;

i_Btrc_SCF_active = 0E-3;

i_Btrc_trash = 0E-3;

% 13 Fbw7

i_mRNA_Fbw7_unspl = 0E-6;

i_mRNA_Fbw7_nuc = 0E-6;

i_mRNA_Fbw7_inactive = 0E-6;

i_mRNA_Fbw7_cytosol = 0E-6;

i_mRNA_Fbw7_in_use = 0E-6;

i_mRNA_Fbw7_trash = 0E-6;

i_Fbw7_unfold = 0E-3;

i_Fbw7_cytosol = 0E-3;

i_Fbw7_nuc = 0E-3;

% i_Fbw7_SCF_active = i_SCF_on_Fbw7;

i_Fbw7_SCF_active = 0E-3;

i_Fbw7_trash = 0E-3;

% 14 TF_grow

i_mRNA_TF_grow_unspl = 0E-6;

i_mRNA_TF_grow_inactive = 0E-6;

i_mRNA_TF_grow_cytosol = 0E-6;

i_mRNA_TF_grow_nuc = 0E-6;

i_mRNA_TF_grow_in_use = 0E-6;

i_mRNA_TF_grow_trash = 0E-6;

i_TF_grow_unfold = 0E-3;

i_TF_grow_cytosol = 0E-3;

i_TF_grow_nuc = 0E-3;

i_TF_grow_on_DNA = 0E-3;

i_TF_grow_trash = 0E-3;

% 15 NT

% i_NT_in_cell = i_NT_in_cell;

% i_NT_in_RNA = i_NT_in_RNA;

% i_NT_in_DNA = 12E9;

i_NT_in_DNA = 6E9;

% 16 RC

i_mRNA_RC_unspl = 0E-6;

i_mRNA_RC_nuc = 0E-6;

i_mRNA_RC_inactive = 0E-6;

i_mRNA_RC_cytosol = 0E-6;

i_mRNA_RC_in_use = 0E-6;

i_mRNA_RC_trash = 0E-6;

i_RC_unfold = 0E-3;

i_RC_cytosol = 0E-3;

i_RC_nuc = 0E-3;

i_RC_on_DNA = 0E-3;

i_RC_licensed = 0E-3;

i_RC_traversed_by_DNA_poly = 0E-3;

i_RC_trash = 0E-3;

i_RC_count = 0E-3;

% 17 DNA_poly

i_mRNA_DNA_poly_unspl = 0E-6;

i_mRNA_DNA_poly_nuc = 0E-6;

i_mRNA_DNA_poly_inactive = 0E-6;

i_mRNA_DNA_poly_cytosol = 0E-6;

i_mRNA_DNA_poly_in_use = 0E-6;

i_mRNA_DNA_poly_trash = 0E-6;

i_DNA_poly_unfold = 0E-3;

i_DNA_poly_cytosol = 0E-3;

i_DNA_poly_nuc = 0E-3;

i_DNA_poly_on_DNA = 0E-3;

i_DNA_poly_trash = 0E-3;

% 18 Wee1

i_mRNA_Wee1_unspl = 1;

i_mRNA_Wee1_nuc = 1;

i_mRNA_Wee1_inactive = 1;

i_mRNA_Wee1_cytosol = 1;

i_mRNA_Wee1_in_use = 1;

i_mRNA_Wee1_trash = 1;

i_Wee1_unfold = 1E1;

% i_Wee1_cytosol_active = 1E6;

% i_Wee1_phospho_inactive = 1E4;

i_Wee1_cytosol_active = 5E5;

i_Wee1_phospho_inactive = 0E-3;

i_Wee1_trash = 1E2;

% 19 cycB

i_mRNA_cycB_unspl = 0E-6;

i_mRNA_cycB_nuc = 0E-6;

i_mRNA_cycB_inactive = 0E-6;

i_mRNA_cycB_cytosol = 0E-6;

i_mRNA_cycB_in_use = 0E-6;

i_mRNA_cycB_trash = 0E-6;

i_cycB_unfold = 0E-3;

i_cycB_cytosol = 0E-3;

i_cycB_Cdk1_cytosol_active = 0E-3;

i_cycB_Cdk1_phospho_inactive = 0E-3;

i_cycB_Cdk1_nuc_active = 0E-3;

i_cycB_trash = 0E-3;

% 20 Cdk1

i_mRNA_Cdk1_unspl = 1;

i_mRNA_Cdk1_nuc = 1;

i_mRNA_Cdk1_inactive = 1;

i_mRNA_Cdk1_cytosol = 1;

i_mRNA_Cdk1_in_use = 1;

i_mRNA_Cdk1_trash = 1;

i_Cdk1_unfold = 1E1;

% i_Cdk1_inactive = 1E2;

i_Cdk1_inactive = 1E5;

% i_Cdk1_cytosol = 1E4;

% i_Cdk1_in_cell = 2E4;

% i_Cdk1_in_cell = 0E-3;

i_Cdk1_in_cell = 1E5;

% i_Cdk1_on_cycB = i_cycB_Cdk1_cytosol_active + i_cycB_Cdk1_phospho_inactive...

% + i_cycB_Cdk1_nuc_active;

i_Cdk1_on_cycB = 0E-3;

% i_Cdk1_nuc = 1E4;

% i_Cdk1_on_cycA = i_cycA_Cdk1_active + i_cycA_Cdk1_phospho_inactive;

i_Cdk1_on_cycA = 0E-3;

i_Cdk1_trash = 1E2;

% 21 Cdc25C

i_mRNA_Cdc25C_unspl = 1;

i_mRNA_Cdc25C_nuc = 1;

i_mRNA_Cdc25C_inactive = 1;

i_mRNA_Cdc25C_cytosol = 1;

i_mRNA_Cdc25C_in_use = 1;

i_mRNA_Cdc25C_trash = 1;

i_Cdc25C_unfold = 1E1;

i_Cdc25C_cytosol_inactive = 1E5;

i_Cdc25C_cytosol_phospho_active = 0E-3;

i_Cdc25C_nuc_phospho_active = 0E-3;

i_Cdc25C_trash = 1E2;

% 22 Plk1

i_mRNA_Plk1_unspl = 0E-6;

i_mRNA_Plk1_nuc = 0E-6;

i_mRNA_Plk1_inactive = 0E-6;

i_mRNA_Plk1_cytosol = 0E-6;

i_mRNA_Plk1_in_use = 0E-6;

i_mRNA_Plk1_trash = 0E-6;

i_Plk1_unfold = 0E-3;

i_Plk1_cytosol_inactive = 0E-3;

i_Plk1_cytosol_phospho_active = 0E-3;

i_Plk1_nuc_phospho_active = 0E-3;

i_Plk1_trash = 0E-3;

% 23 Emi1

i_mRNA_Emi1_unspl = 0E-6;

i_mRNA_Emi1_nuc = 0E-6;

i_mRNA_Emi1_inactive = 0E-6;

i_mRNA_Emi1_cytosol = 0E-6;

i_mRNA_Emi1_in_use = 0E-6;

i_mRNA_Emi1_trash = 0E-6;

i_Emi1_unfold = 0E-3;

i_Emi1_in_cell = 0E-3;

i_Emi1_on_Cdh1 = 0E-3;

i_Emi1_on_Cdc20 = 0E-3;

i_Emi1_phospho_inactive = 0E-3;

i_Emi1_trash = 0E-3;

% 24 APC

i_mRNA_APC_unspl = 1;

i_mRNA_APC_nuc = 1;

i_mRNA_APC_inactive = 1;

i_mRNA_APC_cytosol = 1;

i_mRNA_APC_in_use = 1;

i_mRNA_APC_trash = 1;

i_APC_unfold = 1E1;

i_APC_in_cell = 8E4;

% i_APC_cytosol = 1E3;

% i_APC_nuc = 8E4;

% i_APC_on_Cdh1 = i_Cdh1_APC_active;

% i_APC_on_Cdh1 = 2.5E4;

i_APC_on_Cdh1 = 3.3E4;

% i_APC_on_Cdc20 = i_Cdc20_APC_inactive + i_Cdc20_APC_active;

i_APC_on_Cdc20 = 0E-3;

i_APC_trash = 1E3;

% 25 Cdh1

i_mRNA_Cdh1_unspl = 1;

i_mRNA_Cdh1_nuc = 1;

i_mRNA_Cdh1_inactive = 1;

i_mRNA_Cdh1_cytosol = 1;

i_mRNA_Cdh1_in_use = 1;

i_mRNA_Cdh1_trash = 1;

i_Cdh1_unfold = 1E0;

i_Cdh1_in_cell = 0E-3;

% i_Cdh1_cytosol = 0E-3;

% i_Cdh1_nuc = 0E-3;

% i_Cdh1_Emi1_inactive = i_Emi1_on_Cdh1;

i_Cdh1_Emi1_inactive = 0E-3;

i_Cdh1_phospho_inactive = 0E-3;

% i_Cdh1_APC_active = i_APC_on_Cdh1;

% i_Cdh1_APC_active = 2.5E4;

i_Cdh1_APC_active = 3.3E4;

i_Cdh1_trash = 1E2;

% 26 Cdc20

i_mRNA_Cdc20_unspl = 0E-6;

i_mRNA_Cdc20_nuc = 0E-6;

i_mRNA_Cdc20_inactive = 0E-6;

i_mRNA_Cdc20_cytosol = 0E-6;

i_mRNA_Cdc20_in_use = 0E-6;

i_mRNA_Cdc20_trash = 0E-6;

i_Cdc20_unfold = 0E-3;

i_Cdc20_cytosol = 0E-3;

i_Cdc20_nuc = 0E-3;

% i_Cdc20_Emi1_inactive = i_Emi1_on_Cdc20;

i_Cdc20_Emi1_inactive = 0E-3;

i_Cdc20_APC_inactive = 0E-3;

i_Cdc20_APC_active = 0E-3;

i_Cdc20_trash = 0E-3;

% 27 Cdc14

i_mRNA_Cdc14_unspl = 1;

i_mRNA_Cdc14_nuc = 1;

i_mRNA_Cdc14_inactive = 1;

i_mRNA_Cdc14_cytosol = 1;

i_mRNA_Cdc14_in_use = 1;

i_mRNA_Cdc14_trash = 1;

i_Cdc14_unfold = 1E0;

i_Cdc14_cytosol = 1E0;

i_Cdc14_nuc_inactive = 1E5;

i_Cdc14_inhibited = 1E0;

i_Cdc14_phospho_active = 1E0;

i_Cdc14_trash = 1E2;

% 28 Cdc25A

i_mRNA_Cdc25A_unspl = 0E-6;

i_mRNA_Cdc25A_nuc = 0E-6;

i_mRNA_Cdc25A_inactive = 0E-6;

i_mRNA_Cdc25A_cytosol = 0E-6;

i_mRNA_Cdc25A_in_use = 0E-6;

i_mRNA_Cdc25A_trash = 0E-6;

i_Cdc25A_unfold = 0E-3;

i_Cdc25A_cytosol = 0E-3;

i_Cdc25A_nuc_inactive = 0E-3;

i_Cdc25A_phospho_active = 0E-3;

i_Cdc25A_trash = 0E-3;

% 29 Cdc25B

i_mRNA_Cdc25B_unspl = 0E-6;

i_mRNA_Cdc25B_nuc = 0E-6;

i_mRNA_Cdc25B_inactive = 0E-6;

i_mRNA_Cdc25B_cytosol = 0E-6;

i_mRNA_Cdc25B_in_use = 0E-6;

i_mRNA_Cdc25B_trash = 0E-6;

i_Cdc25B_unfold = 0E-3;

i_Cdc25B_cytosol_inactive = 0E-3;

i_Cdc25B_cytosol_phospho_active = 0E-3;

i_Cdc25B_trash = 0E-3;

% 30 Securin

i_mRNA_Securin_unspl = 0E-6;

i_mRNA_Securin_nuc = 0E-6;

i_mRNA_Securin_inactive = 0E-6;

i_mRNA_Securin_cytosol = 0E-6;

i_mRNA_Securin_in_use = 0E-6;

i_mRNA_Securin_trash = 0E-6;

i_Securin_unfold = 0E-3;

i_Securin_cytosol = 0E-3;

i_Securin_nuc = 0E-3;

i_Securin_on_chromo = 0E-3;

i_Securin_trash = 0E-3;

% 31 cycC/Cdk8

i_mRNA_cycC_unspl = 1;

i_mRNA_cycC_nuc = 1;

i_mRNA_cycC_inactive = 1;

i_mRNA_cycC_cytosol = 1;

i_mRNA_cycC_in_use = 1;

i_mRNA_cycC_trash = 1;

i_cycC_unfold = 1;

i_cycC_cytosol = 2;

i_cycC_nuc = 1.0E3;

i_cycC_Cdk8_active = 3.0E4;

i_cycC_Cdk8_inactive = 0E-3;

i_cycC_trash = 50;

% 32 KPC

i_mRNA_KPC_unspl = 1;

i_mRNA_KPC_nuc = 1;

i_mRNA_KPC_inactive = 1;

i_mRNA_KPC_cytosol = 1;

i_mRNA_KPC_in_use = 1;

i_mRNA_KPC_trash = 1;

i_KPC_unfold = 1;

i_KPC_in_cell = 3.0E4;

i_KPC_active = 0E-3;

i_KPC_trash = 50;

% !!!!!!!!!!!!!!!!!!!!!!!!!!!!!!!!! PACK INITIAL CONDITIONS

y0=[

i_AA_in_cytosol; i_AA_in_p; i_AA_on_tRNA; i_ADP_in_cell; i_ADP_trash;

i_ATP_in_cell; i_fats_cytosol; i_fats_in_mem; i_fats_in_vesicles;

i_H_between_mito_mems; i_H_in_inner_mito_mems; i_junk_spl;

i_junk_spl_mRNA; i_junk_spl_rRNA; i_junk_spl_snRNA; i_mRNA_cytosol;

i_mRNA_in_use; i_mRNA_nuc; i_mRNA_inactive; i_mRNA_trash; i_mRNA_unspl;

i_Na_in_cell; i_Na_out_cell; i_NT_in_cell; i_NT_in_RNA; i_p_in_cell;

i_p_in_use; i_p_trash; i_p_unfold; i_rRNA_cytosol; i_rRNA_in_use;

i_rRNA_nuc; i_rRNA_trash; i_rRNA_unspl; i_snRNA_in_use; i_snRNA_nuc;

i_snRNA_trash; i_snRNA_unspl; i_sugar_in_cell; i_tRNA_cytosol;

i_tRNA_in_use; i_tRNA_nuc; i_tRNA_trash;

i_mRNA_poly_unspl; i_mRNA_poly_nuc; i_mRNA_poly_inactive;

i_mRNA_poly_cytosol; i_mRNA_poly_in_use; i_mRNA_poly_trash;

i_p_poly_unfold; i_p_poly_cytosol; i_p_poly_nuc_inactive;

i_p_poly_nuc_active; i_p_poly_in_use; i_p_poly_trash;

i_mRNA_eIF_4_unspl; i_mRNA_eIF_4_nuc; i_mRNA_eIF_4_inactive;

i_mRNA_eIF_4_cytosol; i_mRNA_eIF_4_in_use; i_mRNA_eIF_4_trash;

i_p_eIF_4_unfold; i_p_eIF_4_cytosol; i_p_eIF_4_in_use; i_p_eIF_4_trash;

i_mRNA_p27_unspl; i_mRNA_p27_nuc; i_mRNA_p27_inactive;

i_mRNA_p27_cytosol; i_mRNA_p27_in_use; i_mRNA_p27_trash; i_p27_unfold;

i_p27_cytosol; i_p27_nuc; i_p27_on_cyclins; i_p27_trash;

i_mRNA_Rb_unspl; i_mRNA_Rb_nuc; i_mRNA_Rb_inactive; i_mRNA_Rb_cytosol;

i_mRNA_Rb_in_use; i_mRNA_Rb_trash; i_Rb_unfold; i_Rb_cytosol; i_Rb_nuc;

i_Rb_on_E2F; i_Rb_phospho_inactive; i_Rb_trash;

i_mRNA_cycD_unspl; i_mRNA_cycD_nuc; i_mRNA_cycD_inactive;

i_mRNA_cycD_cytosol; i_mRNA_cycD_in_use; i_mRNA_cycD_trash;

i_cycD_unfold; i_cycD_cytosol; i_cycD_nuc; i_cycD_Cdk4or6_active;

i_cycD_Cdk4or6_on_p27_inactive; i_cycD_trash;

i_mRNA_Cdk2_unspl; i_mRNA_Cdk2_nuc; i_mRNA_Cdk2_inactive;

i_mRNA_Cdk2_cytosol; i_mRNA_Cdk2_in_use; i_mRNA_Cdk2_trash;

i_Cdk2_unfold; i_Cdk2_cytosol; i_Cdk2_nuc; i_Cdk2_on_cycE;

i_Cdk2_on_cycA; i_Cdk2_trash;

i_mRNA_cycE_unspl; i_mRNA_cycE_nuc; i_mRNA_cycE_inactive;

i_mRNA_cycE_cytosol; i_mRNA_cycE_in_use; i_mRNA_cycE_trash;

i_cycE_unfold; i_cycE_cytosol; i_cycE_nuc; i_cycE_Cdk2_inactive;

i_cycE_Cdk2_active; i_cycE_Cdk2_on_p27_inactive; i_cycE_trash;

i_mRNA_B_Myb_unspl; i_mRNA_B_Myb_nuc; i_mRNA_B_Myb_inactive;

i_mRNA_B_Myb_cytosol; i_mRNA_B_Myb_in_use; i_mRNA_B_Myb_trash;

i_B_Myb_unfold; i_B_Myb_cytosol; i_B_Myb_nuc; i_B_Myb_phospho_active;

i_B_Myb_on_DNA; i_B_Myb_trash;

i_mRNA_NF_Y_unspl; i_mRNA_NF_Y_nuc; i_mRNA_NF_Y_inactive;

i_mRNA_NF_Y_cytosol; i_mRNA_NF_Y_in_use; i_mRNA_NF_Y_trash;

i_NF_Y_unfold; i_NF_Y_cytosol; i_NF_Y_nuc; i_NF_Y_phospho_active;

i_NF_Y_on_DNA; i_NF_Y_trash;

i_mRNA_E2F_unspl; i_mRNA_E2F_nuc; i_mRNA_E2F_inactive;

i_mRNA_E2F_cytosol; i_mRNA_E2F_in_use; i_mRNA_E2F_trash; i_E2F_unfold;

i_E2F_cytosol; i_E2F_nuc; i_E2F_Rb_inactive; i_E2F_on_DNA; i_E2F_trash;

i_mRNA_cycA_unspl; i_mRNA_cycA_nuc; i_mRNA_cycA_inactive;

i_mRNA_cycA_cytosol; i_mRNA_cycA_in_use; i_mRNA_cycA_trash;

i_cycA_unfold; i_cycA_cytosol; i_cycA_nuc; i_cycA_Cdk2_inactive;

i_cycA_Cdk2_on_p27_inactive; i_cycA_Cdk2_active;

i_cycA_Cdk1_cytosol_active; i_cycA_Cdk1_phospho_inactive;

i_cycA_Cdk1_nuc_active; i_cycA_trash;

i_mRNA_SCF_unspl; i_mRNA_SCF_nuc; i_mRNA_SCF_inactive;

i_mRNA_SCF_cytosol; i_mRNA_SCF_in_use; i_mRNA_SCF_trash; i_SCF_unfold;

i_SCF_in_cell; i_SCF_on_Btrc; i_SCF_on_Fbw7; i_SCF_on_Skp2; i_SCF_trash;

i_mRNA_Skp2_unspl; i_mRNA_Skp2_nuc; i_mRNA_Skp2_inactive;

i_mRNA_Skp2_cytosol; i_mRNA_Skp2_in_use; i_mRNA_Skp2_trash;

i_Skp2_unfold; i_Skp2_cytosol; i_Skp2_nuc; i_Skp2_SCF_active;

i_Skp2_trash;

i_mRNA_Btrc_unspl; i_mRNA_Btrc_nuc; i_mRNA_Btrc_inactive;

i_mRNA_Btrc_cytosol; i_mRNA_Btrc_in_use; i_mRNA_Btrc_trash;

i_Btrc_unfold; i_Btrc_cytosol; i_Btrc_SCF_active; i_Btrc_trash;

i_mRNA_Fbw7_unspl; i_mRNA_Fbw7_nuc; i_mRNA_Fbw7_inactive;

i_mRNA_Fbw7_cytosol; i_mRNA_Fbw7_in_use; i_mRNA_Fbw7_trash;

i_Fbw7_unfold; i_Fbw7_cytosol; i_Fbw7_nuc; i_Fbw7_SCF_active;

i_Fbw7_trash;

i_mRNA_TF_grow_unspl; i_mRNA_TF_grow_inactive; i_mRNA_TF_grow_cytosol;

i_mRNA_TF_grow_nuc; i_mRNA_TF_grow_in_use; i_mRNA_TF_grow_trash;

i_TF_grow_unfold; i_TF_grow_cytosol; i_TF_grow_nuc; i_TF_grow_on_DNA;

i_TF_grow_trash;

i_NT_in_DNA;

i_mRNA_RC_unspl; i_mRNA_RC_nuc; i_mRNA_RC_inactive; i_mRNA_RC_cytosol;

i_mRNA_RC_in_use; i_mRNA_RC_trash; i_RC_unfold; i_RC_cytosol; i_RC_nuc;

i_RC_on_DNA; i_RC_licensed; i_RC_traversed_by_DNA_poly; i_RC_trash;

i_RC_count;

i_mRNA_DNA_poly_unspl; i_mRNA_DNA_poly_nuc; i_mRNA_DNA_poly_inactive;

i_mRNA_DNA_poly_cytosol; i_mRNA_DNA_poly_in_use; i_mRNA_DNA_poly_trash;

i_DNA_poly_unfold; i_DNA_poly_cytosol; i_DNA_poly_nuc;

i_DNA_poly_on_DNA; i_DNA_poly_trash;

i_mRNA_Wee1_unspl; i_mRNA_Wee1_nuc; i_mRNA_Wee1_inactive;

i_mRNA_Wee1_cytosol; i_mRNA_Wee1_in_use; i_mRNA_Wee1_trash;

i_Wee1_unfold; i_Wee1_cytosol_active; i_Wee1_phospho_inactive;

i_Wee1_trash;

i_mRNA_cycB_unspl; i_mRNA_cycB_nuc; i_mRNA_cycB_inactive;

i_mRNA_cycB_cytosol; i_mRNA_cycB_in_use; i_mRNA_cycB_trash;

i_cycB_unfold; i_cycB_cytosol; i_cycB_Cdk1_cytosol_active;

i_cycB_Cdk1_phospho_inactive; i_cycB_Cdk1_nuc_active; i_cycB_trash;

i_mRNA_Cdk1_unspl; i_mRNA_Cdk1_nuc; i_mRNA_Cdk1_inactive;

i_mRNA_Cdk1_cytosol; i_mRNA_Cdk1_in_use; i_mRNA_Cdk1_trash;

i_Cdk1_unfold; i_Cdk1_inactive; i_Cdk1_in_cell; i_Cdk1_on_cycB;

i_Cdk1_on_cycA; i_Cdk1_trash;

i_mRNA_Cdc25C_unspl; i_mRNA_Cdc25C_nuc; i_mRNA_Cdc25C_inactive;

i_mRNA_Cdc25C_cytosol; i_mRNA_Cdc25C_in_use; i_mRNA_Cdc25C_trash;

i_Cdc25C_unfold; i_Cdc25C_cytosol_inactive;

i_Cdc25C_cytosol_phospho_active; i_Cdc25C_nuc_phospho_active;

i_Cdc25C_trash;

i_mRNA_Plk1_unspl; i_mRNA_Plk1_nuc; i_mRNA_Plk1_inactive;

i_mRNA_Plk1_cytosol; i_mRNA_Plk1_in_use; i_mRNA_Plk1_trash;

i_Plk1_unfold; i_Plk1_cytosol_inactive; i_Plk1_cytosol_phospho_active;

i_Plk1_nuc_phospho_active; i_Plk1_trash;

i_mRNA_Emi1_unspl; i_mRNA_Emi1_nuc; i_mRNA_Emi1_inactive;

i_mRNA_Emi1_cytosol; i_mRNA_Emi1_in_use; i_mRNA_Emi1_trash;

i_Emi1_unfold; i_Emi1_in_cell; i_Emi1_on_Cdh1; i_Emi1_on_Cdc20;

i_Emi1_phospho_inactive; i_Emi1_trash;

i_mRNA_APC_unspl; i_mRNA_APC_nuc; i_mRNA_APC_inactive;

i_mRNA_APC_cytosol; i_mRNA_APC_in_use; i_mRNA_APC_trash; i_APC_unfold;

i_APC_in_cell; i_APC_on_Cdh1; i_APC_on_Cdc20; i_APC_trash;

i_mRNA_Cdh1_unspl; i_mRNA_Cdh1_nuc; i_mRNA_Cdh1_inactive;

i_mRNA_Cdh1_cytosol; i_mRNA_Cdh1_in_use; i_mRNA_Cdh1_trash;

i_Cdh1_unfold; i_Cdh1_in_cell; i_Cdh1_Emi1_inactive;

i_Cdh1_phospho_inactive; i_Cdh1_APC_active; i_Cdh1_trash;

i_mRNA_Cdc20_unspl; i_mRNA_Cdc20_nuc; i_mRNA_Cdc20_inactive;

i_mRNA_Cdc20_cytosol; i_mRNA_Cdc20_in_use; i_mRNA_Cdc20_trash;

i_Cdc20_unfold; i_Cdc20_cytosol; i_Cdc20_nuc; i_Cdc20_Emi1_inactive;

i_Cdc20_APC_inactive; i_Cdc20_APC_active; i_Cdc20_trash;

i_mRNA_Cdc14_unspl; i_mRNA_Cdc14_nuc; i_mRNA_Cdc14_inactive;

i_mRNA_Cdc14_cytosol; i_mRNA_Cdc14_in_use; i_mRNA_Cdc14_trash;

i_Cdc14_unfold; i_Cdc14_cytosol; i_Cdc14_nuc_inactive;

i_Cdc14_inhibited; i_Cdc14_phospho_active; i_Cdc14_trash;

i_mRNA_Cdc25A_unspl; i_mRNA_Cdc25A_nuc; i_mRNA_Cdc25A_inactive;

i_mRNA_Cdc25A_cytosol; i_mRNA_Cdc25A_in_use; i_mRNA_Cdc25A_trash;

i_Cdc25A_unfold; i_Cdc25A_cytosol; i_Cdc25A_nuc_inactive;

i_Cdc25A_phospho_active; i_Cdc25A_trash;

i_mRNA_Cdc25B_unspl; i_mRNA_Cdc25B_nuc; i_mRNA_Cdc25B_inactive;

i_mRNA_Cdc25B_cytosol; i_mRNA_Cdc25B_in_use; i_mRNA_Cdc25B_trash;

i_Cdc25B_unfold; i_Cdc25B_cytosol_inactive;

i_Cdc25B_cytosol_phospho_active; i_Cdc25B_trash;

i_mRNA_Securin_unspl; i_mRNA_Securin_nuc; i_mRNA_Securin_inactive;

i_mRNA_Securin_cytosol; i_mRNA_Securin_in_use; i_mRNA_Securin_trash;

i_Securin_unfold; i_Securin_cytosol; i_Securin_nuc; i_Securin_on_chromo;

i_Securin_trash;

i_mRNA_cycC_unspl; i_mRNA_cycC_nuc; i_mRNA_cycC_inactive;

i_mRNA_cycC_cytosol; i_mRNA_cycC_in_use; i_mRNA_cycC_trash;

i_cycC_unfold; i_cycC_cytosol; i_cycC_nuc; i_cycC_Cdk8_active;

i_cycC_Cdk8_inactive; i_cycC_trash;

i_mRNA_KPC_unspl; i_mRNA_KPC_nuc; i_mRNA_KPC_inactive;

i_mRNA_KPC_cytosol; i_mRNA_KPC_in_use; i_mRNA_KPC_trash; i_KPC_unfold;

i_KPC_in_cell; i_KPC_active; i_KPC_trash

];

# getconst.m

function z=getconst

% !!!!!!!!!!!!!!!!!!!!!!!!!!!!!!!!!!!!!!!!!!!!!!!! CONSTANTS

%

% base constants

ATP_AA = 2;

ATP_eIF_4 = 1;

ATP_fat = 0;

ATP_fold = 12;

ATP_glycolysis = 2;

ATP_glycosolation = 5;

ATP_H = 0.3333333;

ATP_mem_transfer = 5;

ATP_pm_transfer = 1;

ATP_Na = 0.3333333;

ATP_NT = 2;

ATP_NT_out_nm = 0.2;

ATP_polymerization = 1;

ATP_proteasome = 1;

ATP_respiration = 30;

ATP_spl = 1;

ATP_tRNA = 2;

ATP_u = 3;

ATP_vesicle = 150;

exon_mRNA = 5;

exon_rRNA = 5;

exon_snRNA = 2;

frac_p_cytosol = 0.5;

frac_p_ER = 0.15;

frac_p_lysosome = 0.03;

frac_p_mitochondria = 0.22;

frac_p_nuc = 0.06;

frac_polyI = 0.39;

frac_polyII = 0.58;

frac_polyII_snRNA = 1e-3;

frac_polyIII = 0.03;

i_eIF_4 = 1.0E6;

i_ER_p = 20;

i_ER_RNA = 30;

i_poly_all = 2.0E3;

x_mRNA = 1500;

x_mRNA_unspl = 7E3;

x_p = 400;

x_rRNA = 7000;

x_rRNA_unspl = 1.3E4;

x_snRNA = 1250;

x_snRNA_unspl = 2.5E3;

x_tRNA = 80;

x_vesicle = 1.4E5;

k_ATP_synthase = 3.2E7;

k_bind_p = 5E-2;

k_bind_snRNA = 1;

k_cut_p = 2E-4;

k_cut_RNA = 0.20;

k_cut_mRNA = k_cut_RNA/x_mRNA;

k_cut_rRNA = k_cut_RNA/x_rRNA;

k_cut_snRNA = k_cut_RNA/x_snRNA;

k_cut_tRNA = k_cut_RNA/x_tRNA;

k_cut_spljunk_mRNA = k_cut_RNA;

k_cut_spljunk_rRNA = k_cut_RNA;

k_cut_spljunk_snRNA = k_cut_RNA;

k_dk_AA = 2.2E-9;

k_dk_ADP = 2.2E-9;

k_dk_fat = 2.2E-9;

k_dk_NT = 2.2E-9;

k_dk_p = 3.2E-7;

k_dk_mRNA = 2.7E-4;

k_dk_rRNA = 1.2E-5;

k_dk_snRNA = 1.2E-5;

k_dk_tRNA = 3.9E-6;

k_bind_rRNA_mRNA = 5.9E-6;

k_ER_p_constant = 1.8E-07;

k_ER_RNA_constant = 5.0E-11;

k_fold_p = 1E-1;

k_grow_H = 0.1;

k_grow_Na = 0.1;

k_make_vesicle = 6.1E-2;

k_H_pump = 3.10E9;

k_Na_pump_out = 4.2E-4;

k_Na_return = 3E-5;

% k_nm_mRNA = 5.4E-4;

k_nm_mRNA = 5.3E-4;

k_nm_rRNA = 5.4E-4;

k_nm_tRNA = 5.4E-3;

k_pm_in_AA = 1.0E-2;

k_pm_in_ADP = 1.0E-4;

k_pm_in_NT = 1.0E-2;

k_pm_in_fats = 30;

k_pm_in_sugar = 1;

k_restructure = 1E-9;

k_spl = 4.0E-2;

k_tRNA_AA_binding = 1.0E-10;

k_unbind_fats = 3.0E-4;

k_bind_fats = 3.0E-3;

rRNA_spacing_on_mRNA = 100;

% RNA polymerase

k_bind_p_poly_to_DNA = 1.0E-1;

k_unbind_p_poly_from_DNA_by_APC_Cdc20 = 1.0E-4;

k_nm_in_p = 5.0E-3;

poly_loci_frac = 3.0E-11;

RNA_poly_loci_constitutive = 1.0E5;

RNA_poly_active_constitutive = 6.6E-2;

k_activate_p_poly_by_mitogen = 1.0E-4;

k_deactivate_p_poly_by_cycC_Cdk8 = 2.3E-8;

% eIF_4... z(94)--z(95)

k_activate_mRNA = 1.0E-7;

eIF_4_loci_frac = 1.0E-4;

% 1 p27...

p27_loci_frac = 2E-4;

k_u_p27_by_SCF_Skp2_fast = 3E-5;

k_u_p27_by_SCF_Skp2_slow = 3E-5;

k_u_p27_by_APC_Cdc20 = 1E-5;

%%%% p27 deactivation adjusts length of first G1 (!)

k_u_p27_by_KPC = 3E-10;

% 2 Rb...

Rb_loci_frac = 3E-5;

k_bind_Rb_to_E2F = 1E-1;

k_unbind_Rb_E2F = 1E-6;

% k_phospho_Rb_by_Cdk = 3E-5;

k_phospho_Rb_by_Cdk = 3E-6;

k_unphospho_Rb = 1E-6;

k_dephospho_Rb_by_Cdc14 = 1E-6;

% 3 cycD...

mitogen_stimulation = 0;

cycD_loci_frac = 2E-8;

cycD_constitutive = 1E4;

k_bind_cycD_to_Cdk4or6 = 1E-3;

k_unbind_cycD_Cdk4or6 = 1E-5;

k_bind_cycD_Cdk4or6_to_p27 = 5E-7;

k_unbind_cycD_Cdk4or6_p27 = 1E-7;

k_u_cycD_by_SCF_Skp2 = 3E-10;

k_u_cycD_by_APC_Cdc20 = 3E-6;

% 4 Cdk2...

Cdk2_loci_frac = 1E-9;

Cdk2_constituitive = 1E5;

% 5 cycE...

cycE_loci_frac = 6E-8;

k_bind_cycE_to_Cdk2 = 1E-3;

k_unbind_cycE_Cdk2 = 1E-6;

% k_bind_cycE_Cdk2_to_p27 = 2E-3;

% k_unbind_cycE_Cdk2_p27 = 1E-3;

k_bind_cycE_Cdk2_to_p27 = 1E-3;

k_unbind_cycE_Cdk2_p27 = 2E-3;

k_phospho_cycE_Cdk2 = 0E-6;

k_dephospho_cycE_Cdk2 = 1E-11;

k_dephospho_cycE_Cdk2_by_Cdc25A = 3E-4;

% k_dephospho_cycE_Cdk2_by_Cdc25A = 3E-6;

k_u_cycE_by_SCF_Skp2 = 1E-4;

k_u_cycE_by_SCF_Fbw7 = 1E-5;

% 6 B-Myb...

B_Myb_loci_frac = 1E-10;

k_phospho_B_Myb_by_cyc_Cdk = 1E-7;

k_unphospho_B_Myb = 1E-9;

sat_B_Myb_on_DNA = 1E3;

k_bind_B_Myb_to_DNA = 1E-4;

k_unbind_B_Myb_from_DNA = 1E-4;

k_u_B_Myb_by_SCF_Skp2 = 5E-4;

% 7 NF-Y...

NF_Y_loci_frac = 3E-11;

NF_Y_constitutive = 1E4;

k_phospho_NF_Y_by_cyc_Cdk = 5E-7;

k_unphospho_NF_Y = 0;

k_dephospho_NF_Y_by_Cdc14 = 3E-9;

sat_NF_Y_on_DNA = 3E3;

k_bind_NF_Y_to_DNA = 1E-4;

k_unbind_NF_Y_from_DNA = 1E-6;

% 8 E2F...

E2F_loci_frac = 1.5E-9;

E2F_constituitive = 1E3;

sat_E2F_on_DNA = 1.5E4;

k_bind_E2F_to_DNA = 1E-3;

%%% k_E2F_phospho_by_cyc_Cdk = 2E-3;

k_unbind_E2F_DNA = 7E-4;

k_E2F_phospho_by_cycA_Cdk2 = 0E-5;

k_E2F_phospho_by_cycA_Cdk1 = 0E-3;

k_E2F_phospho_by_cycB_Cdk1 = 1E-3;

k_E2F_phospho_by_cycD_Cdk4or6 = 0E-10;

k_u_E2F_by_SCF_Skp2 = 5E-5;

k_u_E2F_by_APC_Cdc20 = 1E-5;

% 9 cycA...

adhesion_factor = 0;

cycA_loci_frac = 2E-8;

k_bind_cycA_to_Cdk2 = 1E-5;

k_unbind_cycA_Cdk2 = 3E-8;

k_dephospho_cycA_Cdk2_by_Cdc25A = 3E-5;

k_phospho_cycA_Cdk2 = 1E-8;

k_bind_cycA_Cdk2_to_p27 = 5E-8;

% k_unbind_cycA_Cdk2_p27 = 1E-7;

k_unbind_cycA_Cdk2_p27 = 3E-8;

k_bind_cycA_to_Cdk1 = 8E-7;

k_unbind_cycA_Cdk1 = 1E-12;

k_phospho_cycA_Cdk1_by_Wee1 = 6E-6;

k_phospho_cycA_Cdk1_by_phospho_Wee1 = 2E-7;

k_unphospho_cycA_Cdk1_from_Wee1 = 0;

% k_dephospho_cycA_Cdk1_by_Cdc25A = 2E-8;

k_dephospho_cycA_Cdk1_by_Cdc25A = 1E-8;

k_dephospho_cycA_Cdk1_by_Cdc25B = 3E-6;

k_dephospho_cycA_Cdk1_by_Cdc25C = 1E-10;

k_nm_in_cycA_Cdk1_by_Plk1 = 1.5E-9;

k_nm_out_cycA_Cdk1 = 0;

k_u_cycA_by_SCF_Skp2 = 0;

k_u_cycA_by_APC_Cdc20 = 3E-13;

k_u_cycA_by_APC_Cdh1 = 1E-4;

% 10 SCF...

SCF_loci_frac = 5E-6;

k_u_SCF_in_cell_by_APC_Cdh1 = 5E-3;

k_u_SCF_active_by_APC_Cdh1 = 3E-5;

% 11 Skp2...

% Skp2_loci_frac = 7E-6;

Skp2_loci_frac = 5E-6;

k_bind_Skp2_to_SCF = 3E-4;

k_unbind_Skp2_SCF = 1E-6;

k_u_Skp2_by_APC_Cdh1 = 3E-5;

% k_u_auto_Skp2 = 3E-2;

k_u_auto_Skp2 = 2E-2;

% 12 Btrc...

Btrc_loci_frac = 1E-8;

% Btrc_loci_frac = 5E-9;

% mimic Watanabe experiment by reducing Btrc

%%% Btrc_loci_frac = 1E-12;

% k_bind_Btrc_to_SCF = 6E-7;

% k_bind_Btrc_to_SCF = 5E-7;

k_bind_Btrc_to_SCF = 3E-7;

k_unbind_Btrc_SCF = 1E-9;

k_u_Btrc_by_APC_Cdh1 = 1E-7;

k_u_auto_Btrc = 1E-4;

% 13 Fbw7...

Fbw7_loci_frac = 5E-10;

k_bind_Fbw7_to_SCF = 3E-3;

k_unbind_Fbw7_SCF = 1E-6;

k_u_auto_Fbw7 = 1E-2;

% 14 TF_grow...

TF_grow_loci_frac = 1E-6;

TF_grow_txs_by_mitogen = 1;

k_TF_grow_txs_by_Skp2 = 1E-6;

sat_TF_grow_on_DNA = 3.0E3;

k_bind_TF_grow_to_DNA = 3E-4;

k_unbind_TF_grow_DNA = 1E-4;

k_u_TF_grow_by_SCF_Fbw7 = 5E-6;

k_u_TF_grow_by_SCF_Skp2 = 5E-6;

% 15 NT...

% ATP_DNA_rep = 2 + ??????????????

% here ATP_NT_txs = old ATP_txs = 2

% x_DNA = 12E9 NT = 6E9 bp

ATP_DNA_repl = 2;

ATP_NT_txs = 2;

x_DNA = 6E9;

i_ER_DNA = 50;

k_ER_DNA_constant = 5E-11;

%%%% k_div_DNA_factor is somehow related to k_bind_cycA_to_Cdk1

k_div_DNA_factor = 1;

% 16 RC...

% x_RC = 3E9 bp / 2E5 bp = 1.5E4 RC

% x_RC_frag = 2E5 bp (Alberts et al.: segment = 3E4 to 3E5 with ave segment ~ 2E5)

% x_NT_RC_frag = 2E5 bp = 4E5 NT

% x_DNA_poly_per_RC = 2 (?)

x_RC = 1.5E4;

x_RC_frag = 2E5;

x_NT_RC_frag = 4E5;

x_DNA_poly_per_RC = 2;

RC_loci_frac = 1E-8;

k_bind_RC_to_DNA = 1E-2;

k_unbind_RC_DNA = 0;

k_phospho_RC_by_cycD_Cdk4or6 = 1E-5;

k_phospho_RC_by_cycD_Cdk4or6_on_p27 = 0E-5;

% k_phospho_RC_by_cycE_Cdk2 = 1E-3;

k_phospho_RC_by_cycE_Cdk2 = 3E-4;

% k_phospho_RC_by_cycE_Cdk2 = 2E-4;

k_phospho_RC_by_cycA_Cdk2 = 3E-4;

k_u_RC_by_SCF_Fbw7 = 0;

% k_u_RC_by_SCF_Skp2 = 6E-8;

k_u_RC_by_SCF_Skp2 = 5E-8;

k_u_RC_by_APC_Cdh1 = 1E-4;

k_clear_RC_count = 1E-1;

% 17 DNA_poly...

DNA_poly_loci_frac = 2.5E-10;

k_bind_DNA_poly_to_DNA = 5E-2;

sat_DNA_poly = 5E3;

% 18 Wee1...

Wee1_loci_frac = 3E-5;

k_phospho_Wee1_by_cycA_Cdk2 = 0;

k_phospho_Wee1_by_cycA_Cdk1 = 0;

k_phospho_Wee1_by_cycB_Cdk1 = 0;

k_phospho_Wee1_by_Plk1 = 8E-8;

k_unphospho_Wee1 = 2E-9;

% k_unphospho_Wee1 = 1E-9;

k_dephospho_Wee1_by_Cdc14 = 1E-9;

% k_u_Wee1_by_SCF_Btrc = 6E-7;

k_u_Wee1_by_SCF_Btrc = 5E-7;

% k_u_Wee1_by_SCF_Btrc = 3E-7;

k_u_Wee1_by_SCF_Skp2_test = 0;

% 19 cycB...

cycB_loci_frac = 5E-8;

k_bind_cycB_to_Cdk1 = 3E-6;

k_unbind_cycB_Cdk1 = 3E-9;

k_phospho_cycB_Cdk1_by_Wee1 = 5E-3;

k_phospho_cycB_Cdk1_by_phospho_Wee1 = 2E-4;

k_unphospho_cycB_Cdk1_from_Wee1 = 0;

k_dephospho_cycB_Cdk1_by_Cdc25A = 6E-4;

k_dephospho_cycB_Cdk1_by_Cdc25B = 1E-8;

k_dephospho_cycB_Cdk1_by_Cdc25C = 4E-5;

k_nm_in_cycB_Cdk1_by_Plk1 = 1E-9;

k_nm_out_cycB_Cdk1 = 0;

k_u_cycB_by_APC_Cdc20 = 1E-9;

k_u_cycB_by_APC_Cdh1 = 1E-4;

% 20 Cdk1...

Cdk1_loci_frac = 5E-11;

Cdk1_constitutive = 6E5;

%%%% k_activate_Cdk1_by_B_Myb_and_NF_Y also apparently adjusts length of G2

k_activate_Cdk1_by_B_Myb_and_NF_Y = 1E-8;

k_deactivate_Cdk1 = 1E-11;

k_dephospho_Cdk1_by_Cdc14 = 1E-8;

% 21 Cdc25C...

Cdc25C_loci_frac = 1E-5;

k_phospho_Cdc25C_by_cycA_Cdk1 = 1E-10;

k_phospho_Cdc25C_by_cycB_Cdk1 = 2E-8;

k_phospho_Cdc25C_by_Plk1 = 1E-10;

k_dephospho_Cdc25C = 1E-4;

k_unphospho_Cdc25C = 3E-4;

k_nm_in_Cdc25C = 1E-9;

k_nm_out_Cdc25C = 1E-11;

k_dephospho_Cdc25C_by_Cdc14 = 3E-8;

% 22 Plk1...

Plk1_loci_frac = 6E-8;

k_phospho_Plk1_by_cycA_Cdk1 = 7E-10;

k_phospho_Plk1_by_cycB_Cdk1 = 1E-9;

k_unphospho_Plk1 = 1E-8;

k_nm_in_Plk1 = 5E-5;

k_u_Plk1_by_APC_Cdh1 = 1E-6;

% 23 Emi1...

Emi1_loci_frac = 4E-8;

%%%% Cdh1 activation (k_phospho_Cdh1_by_cdks and k_bind_Emi1_to_Cdh1) adjusts length of G1

k_bind_Emi1_to_Cdh1 = 3E-7;

k_unbind_Emi1_Cdh1 = 1E-11;

k_bind_Emi1_to_Cdc20 = 1E-4;

k_unbind_Emi1_Cdc20 = 1E-9;

k_phospho_Emi1_by_cycA_Cdk1 = 1E-7;

k_phospho_Emi1_by_cycB_Cdk1 = 1E-4;

k_u_Emi1_by_SCF_Btrc = 2E-7;

k_u_Emi1_by_SCF_Skp2_test = 3E-6;

% 24 APC...

APC_loci_frac = 1E-5;

% 25 Cdh1...

Cdh1_loci_frac = 1E-5;

%%%% Cdh1 activation (k_phospho_Cdh1_by_cdks and k_bind_Emi1_to_Cdh1) adjusts length of G1

k_phospho_Cdh1_by_cycD_Cdk4or6 = 6E-8;

% k_phospho_Cdh1_by_cycD_Cdk4or6 = 9E-9;

k_phospho_Cdh1_by_cycD_Cdk4or6_on_p27 = 0E-8;

% k_phospho_Cdh1_by_cycE_Cdk2 = 6E-8;

k_phospho_Cdh1_by_cycE_Cdk2 = 2E-8;

% k_phospho_Cdh1_by_cycE_Cdk2 = 1E-8;

k_phospho_Cdh1_by_cycA_Cdk2 = 1E-4;

k_phospho_Cdh1_by_Cdk1 = 1E-6;

%%%% k_dephospho_Cdh1_by_Cdc14 adjusts length of M

k_dephospho_Cdh1_by_Cdc14 = 1E-5;

k_bind_Cdh1_to_APC = 3E-7;

k_unbind_Cdh1_APC = 1E-10;

k_u_auto_Cdh1_APC_active = 1E-6;

% 26 Cdc20...

Cdc20_loci_frac = 6E-6;

k_bind_Cdc20_to_APC = 3E-8;

k_unbind_Cdc20_APC = 3E-10;

k_phospho_Cdc20_by_Cdk1 = 1E-5;

k_unphospho_Cdc20_APC_active = 0;

k_dephospho_Cdc20_APC_by_Cdc14 = 1E-8;

k_u_Cdc20_by_APC_Cdh1 = 1E-4;

% 27 Cdc14...

Cdc14_loci_frac = 1E-5;

k_phospho_Cdc14_by_Plk1 = 1E-5;

k_unphospho_Cdc14 = 6E-5;

k_inhibit_Cdc14_by_Securin = 3E-3;

k_uninhibit_Cdc14 = 2E-4;

% 28 Cdc25A...

Cdc25A_loci_frac = 3E-10;

Cdc25A_constitutive = 3E3;

k_phospho_Cdc25A_by_Cdk2 = 3E-6;

k_phospho_Cdc25A_by_cycA_Cdk1 = 1E-8;

k_phospho_Cdc25A_by_cycB_Cdk1 = 5E-8;

k_dephospho_Cdc25A = 1E-9;

k_dephospho_Cdc25A_by_Cdc14 = 1E-8;

k_u_Cdc25A_by_APC_Cdh1 = 2E-8;

k_u_Cdc25A_by_SCF_Btrc = 1E-5;

% 29 Cdc25B...

Cdc25B_loci_frac = 1E-8;

k_phospho_Cdc25B_by_Cdk2 = 0;

k_phospho_Cdc25B_by_cycA_Cdk1 = 3E-6;

k_phospho_Cdc25B_by_cycB_Cdk1 = 1E-10;

k_phospho_Cdc25B_by_TF_grow = 0;

k_phospho_Cdc25B_by_Plk1 = 3E-7;

k_dephospho_Cdc25B = 1E-12;

k_dephospho_Cdc25B_by_Cdc14 = 1E-8;

k_u_Cdc25B_by_SCF_Btrc = 2E-10;

k_u_Cdc25B_by_APC_Cdh1 = 0;

k_u_Cdc25B_by_APC_Cdh1 = 1E-7;

% 30 Securin...

Securin_loci_frac = 3E-10;

k_bind_Securin_to_chromo = 3E-5;

k_u_Securin_by_APC_Cdc20 = 1E-7;

% 31 cycC/Cdk8...

cycC_loci_frac = 3.0E-6;

k_bind_cycC_to_Cdk8 = 1.0E-3;

k_unbind_cycC_from_Cdk8 = 1.0E-5;

k_deactivate_cycC_Cdk8_by_mitogen = 3.0E-3;

k_activate_cycC_Cdk8_by_Cdc14 = 5.0E-8;

% 32 KPC...

KPC_loci_frac = 3.0E-6;

k_activate_KPC_by_mitogen = 1.0E-4;

k_inactivate_KPC = 1.0E-6;

% !!!!!!!!!!!!!!!!!!!!!!!!!!!!!!!!!!!!!!!!!!! PACK CONSTANTS

z=[

ATP_AA; ATP_eIF_4; ATP_fat; ATP_fold; ATP_glycolysis; ATP_glycosolation;

ATP_H; ATP_mem_transfer; ATP_pm_transfer; ATP_Na; ATP_NT; ATP_NT_out_nm;

ATP_polymerization; ATP_proteasome; ATP_respiration; ATP_spl; ATP_tRNA;

ATP_u; ATP_vesicle; exon_mRNA; exon_rRNA; exon_snRNA; frac_p_cytosol;

frac_p_ER; frac_p_lysosome; frac_p_mitochondria; frac_p_nuc; frac_polyI;

frac_polyII; frac_polyII_snRNA; frac_polyIII; i_eIF_4; i_ER_p; i_ER_RNA;

i_poly_all; x_mRNA; x_mRNA_unspl; x_p; x_rRNA; x_rRNA_unspl; x_snRNA;

x_snRNA_unspl; x_tRNA; x_vesicle; k_ATP_synthase; k_bind_p;

k_bind_snRNA; k_cut_p; k_cut_RNA; k_cut_mRNA; k_cut_rRNA; k_cut_snRNA;

k_cut_tRNA; k_cut_spljunk_mRNA; k_cut_spljunk_rRNA; k_cut_spljunk_snRNA;

k_dk_AA; k_dk_ADP; k_dk_fat; k_dk_NT; k_dk_p; k_dk_mRNA; k_dk_rRNA;

k_dk_snRNA; k_dk_tRNA; k_bind_rRNA_mRNA; k_ER_p_constant;

k_ER_RNA_constant; k_fold_p; k_grow_H; k_grow_Na; k_make_vesicle;

k_H_pump; k_Na_pump_out; k_Na_return; k_nm_mRNA; k_nm_rRNA; k_nm_tRNA;

k_pm_in_AA; k_pm_in_ADP; k_pm_in_NT; k_pm_in_fats; k_pm_in_sugar;

k_restructure; k_spl; k_tRNA_AA_binding; k_unbind_fats; k_bind_fats;

rRNA_spacing_on_mRNA;

k_bind_p_poly_to_DNA; k_unbind_p_poly_from_DNA_by_APC_Cdc20; k_nm_in_p;

poly_loci_frac; RNA_poly_loci_constitutive;

RNA_poly_active_constitutive; k_activate_p_poly_by_mitogen;

k_deactivate_p_poly_by_cycC_Cdk8;

k_activate_mRNA; eIF_4_loci_frac;

p27_loci_frac; k_u_p27_by_SCF_Skp2_fast; k_u_p27_by_SCF_Skp2_slow;

k_u_p27_by_APC_Cdc20; k_u_p27_by_KPC;

Rb_loci_frac; k_bind_Rb_to_E2F; k_unbind_Rb_E2F; k_phospho_Rb_by_Cdk;

k_unphospho_Rb; k_dephospho_Rb_by_Cdc14;

mitogen_stimulation; cycD_loci_frac; cycD_constitutive;

k_bind_cycD_to_Cdk4or6; k_unbind_cycD_Cdk4or6;

k_bind_cycD_Cdk4or6_to_p27; k_unbind_cycD_Cdk4or6_p27;

k_u_cycD_by_SCF_Skp2; k_u_cycD_by_APC_Cdc20;

Cdk2_loci_frac; Cdk2_constituitive;

cycE_loci_frac; k_bind_cycE_to_Cdk2; k_unbind_cycE_Cdk2;

k_bind_cycE_Cdk2_to_p27; k_unbind_cycE_Cdk2_p27; k_phospho_cycE_Cdk2;

k_dephospho_cycE_Cdk2; k_dephospho_cycE_Cdk2_by_Cdc25A;

k_u_cycE_by_SCF_Skp2; k_u_cycE_by_SCF_Fbw7;

B_Myb_loci_frac; k_phospho_B_Myb_by_cyc_Cdk; k_unphospho_B_Myb;

sat_B_Myb_on_DNA; k_bind_B_Myb_to_DNA; k_unbind_B_Myb_from_DNA;

k_u_B_Myb_by_SCF_Skp2;

NF_Y_loci_frac; NF_Y_constitutive; k_phospho_NF_Y_by_cyc_Cdk;

k_unphospho_NF_Y; k_dephospho_NF_Y_by_Cdc14; sat_NF_Y_on_DNA;

k_bind_NF_Y_to_DNA; k_unbind_NF_Y_from_DNA;

E2F_loci_frac; E2F_constituitive; sat_E2F_on_DNA; k_bind_E2F_to_DNA;

k_unbind_E2F_DNA; k_E2F_phospho_by_cycA_Cdk2;

k_E2F_phospho_by_cycA_Cdk1; k_E2F_phospho_by_cycB_Cdk1;

k_E2F_phospho_by_cycD_Cdk4or6; k_u_E2F_by_SCF_Skp2;

k_u_E2F_by_APC_Cdc20;

adhesion_factor; cycA_loci_frac; k_bind_cycA_to_Cdk2;

k_unbind_cycA_Cdk2; k_dephospho_cycA_Cdk2_by_Cdc25A;

k_phospho_cycA_Cdk2; k_bind_cycA_Cdk2_to_p27; k_unbind_cycA_Cdk2_p27;

k_bind_cycA_to_Cdk1; k_unbind_cycA_Cdk1; k_phospho_cycA_Cdk1_by_Wee1;

k_phospho_cycA_Cdk1_by_phospho_Wee1; k_unphospho_cycA_Cdk1_from_Wee1;

k_dephospho_cycA_Cdk1_by_Cdc25A; k_dephospho_cycA_Cdk1_by_Cdc25B;

k_dephospho_cycA_Cdk1_by_Cdc25C; k_nm_in_cycA_Cdk1_by_Plk1;

k_nm_out_cycA_Cdk1; k_u_cycA_by_SCF_Skp2; k_u_cycA_by_APC_Cdc20;

k_u_cycA_by_APC_Cdh1;

SCF_loci_frac; k_u_SCF_in_cell_by_APC_Cdh1; k_u_SCF_active_by_APC_Cdh1;

Skp2_loci_frac; k_bind_Skp2_to_SCF; k_unbind_Skp2_SCF;

k_u_Skp2_by_APC_Cdh1; k_u_auto_Skp2;

Btrc_loci_frac; k_bind_Btrc_to_SCF; k_unbind_Btrc_SCF;

k_u_Btrc_by_APC_Cdh1; k_u_auto_Btrc;

Fbw7_loci_frac; k_bind_Fbw7_to_SCF; k_unbind_Fbw7_SCF; k_u_auto_Fbw7;

TF_grow_loci_frac; TF_grow_txs_by_mitogen; k_TF_grow_txs_by_Skp2;

sat_TF_grow_on_DNA; k_bind_TF_grow_to_DNA; k_unbind_TF_grow_DNA;

k_u_TF_grow_by_SCF_Fbw7; k_u_TF_grow_by_SCF_Skp2;

ATP_DNA_repl; ATP_NT_txs; x_DNA; i_ER_DNA; k_ER_DNA_constant;

k_div_DNA_factor;

x_RC ; x_RC_frag; x_NT_RC_frag; x_DNA_poly_per_RC; RC_loci_frac;

k_bind_RC_to_DNA; k_unbind_RC_DNA; k_phospho_RC_by_cycD_Cdk4or6;

k_phospho_RC_by_cycD_Cdk4or6_on_p27; k_phospho_RC_by_cycE_Cdk2;

k_phospho_RC_by_cycA_Cdk2; k_u_RC_by_SCF_Fbw7; k_u_RC_by_SCF_Skp2;

k_u_RC_by_APC_Cdh1; k_clear_RC_count;

DNA_poly_loci_frac; k_bind_DNA_poly_to_DNA; sat_DNA_poly;

Wee1_loci_frac; k_phospho_Wee1_by_cycA_Cdk2;

k_phospho_Wee1_by_cycA_Cdk1; k_phospho_Wee1_by_cycB_Cdk1;

k_phospho_Wee1_by_Plk1; k_unphospho_Wee1; k_dephospho_Wee1_by_Cdc14;

k_u_Wee1_by_SCF_Btrc; k_u_Wee1_by_SCF_Skp2_test;

cycB_loci_frac; k_bind_cycB_to_Cdk1; k_unbind_cycB_Cdk1;

k_phospho_cycB_Cdk1_by_Wee1; k_phospho_cycB_Cdk1_by_phospho_Wee1;

k_unphospho_cycB_Cdk1_from_Wee1; k_dephospho_cycB_Cdk1_by_Cdc25A;

k_dephospho_cycB_Cdk1_by_Cdc25B; k_dephospho_cycB_Cdk1_by_Cdc25C;

k_nm_in_cycB_Cdk1_by_Plk1; k_nm_out_cycB_Cdk1; k_u_cycB_by_APC_Cdc20;

k_u_cycB_by_APC_Cdh1;

Cdk1_loci_frac; Cdk1_constitutive; k_activate_Cdk1_by_B_Myb_and_NF_Y;

k_deactivate_Cdk1; k_dephospho_Cdk1_by_Cdc14;

Cdc25C_loci_frac; k_phospho_Cdc25C_by_cycA_Cdk1;

k_phospho_Cdc25C_by_cycB_Cdk1; k_phospho_Cdc25C_by_Plk1;

k_dephospho_Cdc25C; k_unphospho_Cdc25C; k_nm_in_Cdc25C; k_nm_out_Cdc25C;

k_dephospho_Cdc25C_by_Cdc14;

Plk1_loci_frac; k_phospho_Plk1_by_cycA_Cdk1;

k_phospho_Plk1_by_cycB_Cdk1; k_unphospho_Plk1; k_nm_in_Plk1;

k_u_Plk1_by_APC_Cdh1;

Emi1_loci_frac; k_bind_Emi1_to_Cdh1; k_unbind_Emi1_Cdh1;

k_bind_Emi1_to_Cdc20; k_unbind_Emi1_Cdc20; k_phospho_Emi1_by_cycA_Cdk1;

k_phospho_Emi1_by_cycB_Cdk1; k_u_Emi1_by_SCF_Btrc;

k_u_Emi1_by_SCF_Skp2_test;

APC_loci_frac;

Cdh1_loci_frac; k_phospho_Cdh1_by_cycD_Cdk4or6;

k_phospho_Cdh1_by_cycD_Cdk4or6_on_p27; k_phospho_Cdh1_by_cycE_Cdk2;

k_phospho_Cdh1_by_cycA_Cdk2; k_phospho_Cdh1_by_Cdk1;

k_dephospho_Cdh1_by_Cdc14; k_bind_Cdh1_to_APC; k_unbind_Cdh1_APC;

k_u_auto_Cdh1_APC_active;

Cdc20_loci_frac; k_bind_Cdc20_to_APC; k_unbind_Cdc20_APC;

k_phospho_Cdc20_by_Cdk1; k_unphospho_Cdc20_APC_active;

k_dephospho_Cdc20_APC_by_Cdc14; k_u_Cdc20_by_APC_Cdh1;

Cdc14_loci_frac; k_phospho_Cdc14_by_Plk1; k_unphospho_Cdc14;

k_inhibit_Cdc14_by_Securin; k_uninhibit_Cdc14;

Cdc25A_loci_frac; Cdc25A_constitutive; k_phospho_Cdc25A_by_Cdk2;

k_phospho_Cdc25A_by_cycA_Cdk1; k_phospho_Cdc25A_by_cycB_Cdk1;

k_dephospho_Cdc25A; k_dephospho_Cdc25A_by_Cdc14; k_u_Cdc25A_by_APC_Cdh1;

k_u_Cdc25A_by_SCF_Btrc;

Cdc25B_loci_frac; k_phospho_Cdc25B_by_Cdk2;

k_phospho_Cdc25B_by_cycA_Cdk1; k_phospho_Cdc25B_by_cycB_Cdk1;

k_phospho_Cdc25B_by_TF_grow; k_phospho_Cdc25B_by_Plk1;

k_dephospho_Cdc25B; k_dephospho_Cdc25B_by_Cdc14; k_u_Cdc25B_by_SCF_Btrc;

k_u_Cdc25B_by_APC_Cdh1; k_u_Cdc25B_by_APC_Cdh1;

Securin_loci_frac; k_bind_Securin_to_chromo; k_u_Securin_by_APC_Cdc20;

cycC_loci_frac; k_bind_cycC_to_Cdk8; k_unbind_cycC_from_Cdk8;

k_deactivate_cycC_Cdk8_by_mitogen; k_activate_cycC_Cdk8_by_Cdc14;

KPC_loci_frac; k_activate_KPC_by_mitogen; k_inactivate_KPC

];

# getdividing.m

function [tprev,dividing,k_div] = getdividing...

(tnow,bind_Cdh1_to_APC,unbind_Cdh1_APC,u_Cdh1_APC_active,i_NT_in_DNA,NT_in_DNA)

% if bind_Cdh1_to_APC is increasing rapidly, the cell cycle is finished,

% and the cell is dividing (actually, the cell is enterging G1)

% if cell is dividing and p_in_use is still greater than i_p_in_use, cell continues to divide

% else cell is not dividing (NOTE: I need to change this to be NT_in_DNA)

% NOTE for this algorithm to work, Cdh1 must bind to APC slower than NT_in_DNA is cut in half

dividing = 0;

tprev = 0;

k_div = 0;

if(max(100, 10*abs(unbind_Cdh1_APC + u_Cdh1_APC_active)) < bind_Cdh1_to_APC)

dividing = 1;

% k_div = 5E-3;

% k_div = 3E-3;

k_div = 1E-3;

if(1.0001*i_NT_in_DNA > NT_in_DNA)

dividing = 0;

k_div = 0;

end

end

# cellssplot.m

function cellssplot(t,y)

% cellssplot.m

% tunit=t;

tunit=t/86400;

% tunit=t/3.15576;

lt=length(t);

% close all

figure( 70)

subplot(3,2,1)

plot(tunit,y(1:lt,6),'-')

xlabel('days')

legend('6 ATP in cell')

subplot(3,2,2)

plot(tunit,y(1:lt,4),'-')

xlabel('days')

legend('4 ADP in cell')

subplot(3,2,3)

plot(tunit,y(1:lt,10),'-')

xlabel('days')

legend('10 H between mito mems')

subplot(3,2,4)

plot(tunit,y(1:lt,11),'-')

xlabel('days')

legend('11 H in inner mito mems')

subplot(3,2,5)

plot(tunit,y(1:lt,27),'-')

xlabel('days')

legend('27 p in use')

subplot(3,2,6)

plot(tunit,y(1:lt,233),'-')

xlabel('days')

legend('233 NT in DNA')

figure( 71)

subplot(3,2,1)

plot(tunit,y(1:lt,16),'-',tunit,y(1:lt,17),'--')

xlabel('days')

legend('16 mRNA cytosol','17 mRNA in use')

subplot(3,2,2)

plot(tunit,y(1:lt,30),'-',tunit,y(1:lt,31),'--')

xlabel('days')

legend('30 rRNA cytosol','31 rRNA in use')

subplot(3,2,3)

plot(tunit,y(1:lt,40),'-',tunit,y(1:lt,41),'--')

xlabel('days')

legend('40 tRNA cytosol','41 tRNA in use')

subplot(3,2,4)

plot(tunit,y(1:lt,63),'-',tunit,y(1:lt,64),'--')

xlabel('days')

legend('63 eIF 4 cytosol','64 eIF 4 in use')

subplot(3,2,5)

plot(tunit,y(1:lt,24),'-',tunit,y(1:lt,25),'--',tunit,y(1:lt,233),':')

xlabel('days')

legend('24 NT in cell','25 NT in RNA','233 NT in DNA')

subplot(3,2,6)

plot(tunit,y(1:lt,73),'-',tunit,y(1:lt,74),'--',tunit,y(1:lt,75),':')

xlabel('days')

legend('73 p27 cytosol','74 p27 nuc','75 p27 on cyclins')

figure( 72)

subplot(2,2,1)

plot(tunit,y(1:lt,85),'-',tunit,y(1:lt,86),'--',tunit,y(1:lt,87),':')

xlabel('days')

legend('85 Rb nuc','86 Rb on E2F','87 Rb phospho inactive')

subplot(2,2,2)

plot(tunit,y(1:lt,97),'-',tunit,y(1:lt,98),'--',tunit,y(1:lt,99),':')

xlabel('days')

legend('97 cycD nuc','98 cycD Cdk4or6 active','99 cycD Cdk4or6 on p27')

subplot(2,2,3)

plot(tunit,y(1:lt,109),'-',tunit,y(1:lt,110),'--',tunit,y(1:lt,111),':')

xlabel('days')

legend('109 Cdk2 nuc','110 Cdk2 on cycE','111 Cdk2 on cycA')

subplot(2,2,4)

plot(tunit,y(1:lt,288),'-',tunit,y(1:lt,289),'--',tunit,y(1:lt,290),':',tunit,y(1:lt,291),'-.')

xlabel('days')

legend('288 Cdk1 inactive','289 Cdk1 in cell','290 Cdk1 on cycB','291 Cdk1 on cycA')

figure( 73)

subplot(2,2,1)

plot(tunit,y(1:lt,121),'-',tunit,y(1:lt,122),'--',tunit,y(1:lt,123),':',tunit,y(1:lt,124),'-.')

xlabel('days')

legend('121 cycE nuc','122 cycE Cdk2 inactive','123 cycE Cdk2 active','124 cycE Cdk2 on p27')

subplot(2,2,2)

plot(tunit,y(1:lt,158),'-',tunit,y(1:lt,159),'--',tunit,y(1:lt,160),':')

xlabel('days')

legend('158 E2F nuc','159 E2F Rb inactive','160 E2F on DNA')

subplot(2,2,3)

plot(tunit,y(1:lt,170),'-',tunit,y(1:lt,171),'--',tunit,y(1:lt,173),':',tunit,y(1:lt,172),'-.')

xlabel('days')

legend('170 cycA nuc','171 cycA Cdk2 inactive','173 cycA Cdk2 active','172 cycA Cdk2 on p27')

subplot(2,2,4)

plot(tunit,y(1:lt,169),'-',tunit,y(1:lt,174),'--',tunit,y(1:lt,175),':',tunit,y(1:lt,176),'-.')

xlabel('days')

legend('169 cycA cytosol','174 cycA Cdk1 cytosol active','175 cycA Cdk1 phospho inactive','176 cycA Cdk1 nuc active')

figure( 74)

subplot(2,2,1)

plot(tunit,y(1:lt,185),'-',tunit,y(1:lt,186),'--',tunit,y(1:lt,187),':',tunit,y(1:lt,188),'-.')

xlabel('days')

legend('185 SCF in cell','186 SCF on Btrc','187 SCF on Fbw7','188 SCF on Skp2')

subplot(2,2,2)

plot(tunit,y(1:lt,198),'-',tunit,y(1:lt,199),'--')

xlabel('days')

legend('198 Skp2 nuc','199 Skp2 SCF active')

subplot(2,2,3)

plot(tunit,y(1:lt,208),'-',tunit,y(1:lt,209),'--')

xlabel('days')

legend('208 Btrc cytosol','209 Btrc SCF active')

subplot(2,2,4)

plot(tunit,y(1:lt,219),'-',tunit,y(1:lt,220),'--')

xlabel('days')

legend('219 Fbw7 nuc','220 Fbw7 SCF active')

figure( 75)

subplot(2,2,1)

plot(tunit,y(1:lt,134),'-',tunit,y(1:lt,135),'--',tunit,y(1:lt,136),':')

xlabel('days')

legend('134 B Myb nuc','135 B Myb phospho active','136 B Myb on DNA')

subplot(2,2,2)

plot(tunit,y(1:lt,146),'-',tunit,y(1:lt,147),'--',tunit,y(1:lt,148),':')

xlabel('days')

legend('146 NF Y nuc','147 NF Y phospho active','148 NF Y on DNA')

subplot(2,2,3)

plot(tunit,y(1:lt,230),'-',tunit,y(1:lt,231),'--')

xlabel('days')

legend('230 TF grow nuc','231 TF grow on DNA')

subplot(2,2,4)

plot(tunit,y(1:lt,242),'-',tunit,y(1:lt,243),'--',tunit,y(1:lt,244),':',tunit,y(1:lt,245),'-.',tunit,y(1:lt,247),'--')

xlabel('days')

legend('242 RC nuc','243 RC on DNA','244 RC licensed','245 RC traversed','247 RC count')

figure( 76)

subplot(2,2,1)

plot(tunit,y(1:lt,256),'-',tunit,y(1:lt,257),'--')

xlabel('days')

legend('256 DNA poly nuc','257 DNA poly on DNA')

subplot(2,2,2)

plot(tunit,y(1:lt,266),'-',tunit,y(1:lt,267),'--')

xlabel('days')

legend('266 Wee1 cytosol active','267 Wee1 phospho inactive')

subplot(2,2,3)

plot(tunit,y(1:lt,276),'-',tunit,y(1:lt,277),'--',tunit,y(1:lt,278),':',tunit,y(1:lt,279),'-.')

xlabel('days')

legend('276 cycB cytosol','277 cycB Cdk1 cytosol active','278 cycB Cdk1 phospho inactive','279 cycB Cdk1 nuc active')

subplot(2,2,4)

plot(tunit,y(1:lt,311),'-',tunit,y(1:lt,312),'--',tunit,y(1:lt,313),':')

xlabel('days')

legend('311 Plk1 cytosol inactive','312 Plk1 cytosol phospho active','313 Plk1 nuc phospho active')

figure( 77)

subplot(2,2,1)

plot(tunit,y(1:lt,322),'-',tunit,y(1:lt,323),'--',tunit,y(1:lt,324),':',tunit,y(1:lt,325),'-.')

xlabel('days')

legend('322 Emi1 in cell','323 Emi1 on Cdh1','324 Emi1 on Cdc20','325 Emi1 phospho inactive')

subplot(2,2,2)

plot(tunit,y(1:lt,334),'-',tunit,y(1:lt,335),'--',tunit,y(1:lt,336),':')

xlabel('days')

legend('334 APC in cell','335 APC on Cdh1','336 APC on Cdc20')

subplot(2,2,3)

plot(tunit,y(1:lt,345),'-',tunit,y(1:lt,346),'--',tunit,y(1:lt,347),':',tunit,y(1:lt,348),'-.')

xlabel('days')

legend('345 Cdh1 in cell','346 Cdh1 Emi1 inactive','347 Cdh1 phospho inactive','348 Cdh1 APC active')

subplot(2,2,4)

plot(tunit,y(1:lt,358),'-',tunit,y(1:lt,359),'--',tunit,y(1:lt,360),':',tunit,y(1:lt,361),'-.')

xlabel('days')

legend('358 Cdc20 nuc','359 Cdc20 Emi1 inactive','360 Cdc20 APC inactive','361 Cdc20 APC active')

figure( 78)

subplot(2,2,1)

plot(tunit,y(1:lt,383),'-',tunit,y(1:lt,384),'--')

xlabel('days')

legend('383 Cdc25A nuc inactive','384 Cdc25A phospho active')

subplot(2,2,2)

plot(tunit,y(1:lt,393),'-',tunit,y(1:lt,394),'--')

xlabel('days')

legend('393 Cdc25B cytosol inactive','394 Cdc25B cytosol phospho active')

subplot(2,2,3)

plot(tunit,y(1:lt,300),'-',tunit,y(1:lt,301),'--',tunit,y(1:lt,302),':')

xlabel('days')

legend('300 Cdc25C cytosol inactive','301 Cdc25C cytosol phospho active','302 Cdc25C nuc phospho active')

subplot(2,2,4)

plot(tunit,y(1:lt,371),'-',tunit,y(1:lt,372),'--',tunit,y(1:lt,373),':')

xlabel('days')

legend('371 Cdc14 nuc inactive','372 Cdc14 inhibited','373 Cdc14 phospho active')

figure( 79)

subplot(2,2,1)

plot(tunit,y(1:lt,404),'-',tunit,y(1:lt,405),'--')

xlabel('days')

legend('404 Securin nuc','405 Securin on chromo')

subplot(2,2,2)

plot(tunit,y(1:lt,52),'-',tunit,y(1:lt,53),'--',tunit,y(1:lt,54),':')

xlabel('days')

legend('52 p poly nuc inactive','53 p poly nuc active','54 p poly in use')

subplot(2,2,3)

plot(tunit,y(1:lt,426),'-',tunit,y(1:lt,427),'--')

xlabel('days')

legend('426 KPC in cell','427 KPC active')

subplot(2,2,4)

plot(tunit,y(1:lt,415),'-',tunit,y(1:lt,416),'--',tunit,y(1:lt,417),':')

xlabel('days')

legend('415 cycC nuc','416 cycC Cdk8 active','417 cycC Cdk8 inactive')

figure( 80)

subplot(3,2,1)

plot(tunit,y(1:lt,266),'-',tunit,y(1:lt,267),'--')

xlabel('days')

legend('266 Wee1 cytosol active','267 Wee1 phospho inactive')

subplot(3,2,2)

plot(tunit,y(1:lt,311),'-',tunit,y(1:lt,312),'--',tunit,y(1:lt,313),':')

xlabel('days')

legend('311 Plk1 cytosol inactive','312 Plk1 cytosol phospho active','313 Plk1 nuc phospho active')

subplot(3,2,3)

plot(tunit,y(1:lt,185),'-',tunit,y(1:lt,186),'--',tunit,y(1:lt,187),':',tunit,y(1:lt,188),'-.')

xlabel('days')

legend('185 SCF in cell','186 SCF on Btrc','187 SCF on Fbw7','188 SCF on Skp2')

subplot(3,2,4)

plot(tunit,y(1:lt,169),'-',tunit,y(1:lt,174),'--',tunit,y(1:lt,175),':',tunit,y(1:lt,176),'-.')

xlabel('days')

legend('169 cycA cytosol','174 cycA Cdk1 cytosol active','175 cycA Cdk1 phospho inactive','176 cycA Cdk1 nuc active')

subplot(3,2,5)

plot(tunit,y(1:lt,276),'-',tunit,y(1:lt,277),'--',tunit,y(1:lt,278),':',tunit,y(1:lt,279),'-.')

xlabel('days')

legend('276 cycB cytosol','277 cycB Cdk1 cytosol active','278 cycB Cdk1 phospho inactive','279 cycB Cdk1 nuc active')

subplot(3,2,6)

plot(tunit,y(1:lt,27),'-')

xlabel('days')

legend('27 p in use')

# celldynplot.m

celldynplot.m is identical to cellssplot.m except for the following…

On line 2, change…

< function cellssplot(t,y)

> function celldynplot(t,y)

On line 4, change…

< % cellssplot.m

> % celldynplot.m

On line 15, change…

< figure( 70)

> figure( 90)

On line 41, change…

< figure( 71)

> figure( 91)

On line 67, change…

< figure( 72)

> figure( 92)

On line 85, change…

< figure( 73)

> figure( 93)

On line 103, change…

< figure( 74)

> figure( 94)

On line 121, change…

< figure( 75)

> figure( 95)

On line 139, change…

< figure( 76)

> figure( 96)

On line 157, change…

< figure( 77)

> figure( 97)

On line 175, change…

< figure( 78)

> figure( 98)

On line 193, change…

< figure( 79)

> figure( 99)

On line 211, change…

< figure( 80)

> figure(100)

# cellssrates.m

function cellssrates(ylength,y)

fid=fopen('rates.dat','w');

% !!!!!!!!!!!!!!!!!!!!!!!!!!!!!!!!!!!!!!!!!! GET CONSTANTS

[z,y0]=getssconst;

% !!!!!!!!!!!!!!!!!!!!!!!!!!!!!!!!!!!!!!! UNPACK CONSTANTS

ATP_AA = z(1);

ATP_eIF_4 = z(2);

ATP_fat = z(3);

ATP_fold = z(4);

ATP_glycolysis = z(5);

ATP_glycosolation = z(6);

ATP_H = z(7);

ATP_mem_transfer = z(8);

ATP_pm_transfer = z(9);

ATP_Na = z(10);

ATP_NT = z(11);

ATP_NT_out_nm = z(12);

ATP_polymerization = z(13);

ATP_proteasome = z(14);

ATP_respiration = z(15);

ATP_spl = z(16);

ATP_tRNA = z(17);

ATP_u = z(18);

ATP_vesicle = z(19);

exon_mRNA = z(20);

exon_rRNA = z(21);

exon_snRNA = z(22);

frac_p_cytosol = z(23);

frac_p_ER = z(24);

frac_p_lysosome = z(25);

frac_p_mitochondria = z(26);

frac_p_nuc = z(27);

frac_polyI = z(28);

frac_polyII = z(29);

frac_polyII_snRNA = z(30);

frac_polyIII = z(31);

i_eIF_4 = z(32);

i_ER_p = z(33);

i_ER_RNA = z(34);

i_poly_all = z(35);

x_mRNA = z(36);

x_mRNA_unspl = z(37);

x_p = z(38);

x_rRNA = z(39);

x_rRNA_unspl = z(40);

x_snRNA = z(41);

x_snRNA_unspl = z(42);

x_tRNA = z(43);

x_vesicle = z(44);

k_ATP_synthase = z(45);

k_bind_p = z(46);

k_bind_snRNA = z(47);

k_cut_p = z(48);

k_cut_RNA = z(49);

k_cut_mRNA = z(50);

k_cut_rRNA = z(51);

k_cut_snRNA = z(52);

k_cut_tRNA = z(53);

k_cut_spljunk_mRNA = z(54);

k_cut_spljunk_rRNA = z(55);

k_cut_spljunk_snRNA = z(56);

k_dk_AA = z(57);

k_dk_ADP = z(58);

k_dk_fat = z(59);

k_dk_NT = z(60);

k_dk_p = z(61);

k_dk_mRNA = z(62);

k_dk_rRNA = z(63);

k_dk_snRNA = z(64);

k_dk_tRNA = z(65);

k_bind_rRNA_mRNA = z(66);

k_ER_p_constant = z(67);

k_ER_RNA_constant = z(68);

k_fold_p = z(69);

k_grow_H = z(70);

k_grow_Na = z(71);

k_make_vesicle = z(72);

k_H_pump = z(73);

k_Na_pump_out = z(74);

k_Na_return = z(75);

k_nm_mRNA = z(76);

k_nm_rRNA = z(77);

k_nm_tRNA = z(78);

k_pm_in_AA = z(79);

k_pm_in_ADP = z(80);

k_pm_in_NT = z(81);

k_pm_in_fats = z(82);

k_pm_in_sugar = z(83);

k_restructure = z(84);

k_spl = z(85);

k_tRNA_AA_binding = z(86);

k_unbind_fats = z(87);

k_bind_fats = z(88);

rRNA_spacing_on_mRNA = z(89);

k_bind_p_poly_to_DNA = z(90);

k_unbind_p_poly_from_DNA_by_APC_Cdc20 = z(91);

k_nm_in_p = z(92);

poly_loci_frac = z(93);

RNA_poly_loci_constitutive = z(94);

RNA_poly_active_constitutive = z(95);

k_activate_p_poly_by_mitogen = z(96);

k_deactivate_p_poly_by_cycC_Cdk8 = z(97);

k_activate_mRNA = z(98);

eIF_4_loci_frac = z(99);

p27_loci_frac = z(100);

k_u_p27_by_SCF_Skp2_fast = z(101);

k_u_p27_by_SCF_Skp2_slow = z(102);

k_u_p27_by_APC_Cdc20 = z(103);

k_u_p27_by_KPC = z(104);

Rb_loci_frac = z(105);

k_bind_Rb_to_E2F = z(106);

k_unbind_Rb_E2F = z(107);

k_phospho_Rb_by_Cdk = z(108);

k_unphospho_Rb = z(109);

k_dephospho_Rb_by_Cdc14 = z(110);

mitogen_stimulation = z(111);

cycD_loci_frac = z(112);

cycD_constitutive = z(113);

k_bind_cycD_to_Cdk4or6 = z(114);

k_unbind_cycD_Cdk4or6 = z(115);

k_bind_cycD_Cdk4or6_to_p27 = z(116);

k_unbind_cycD_Cdk4or6_p27 = z(117);

k_u_cycD_by_SCF_Skp2 = z(118);

k_u_cycD_by_APC_Cdc20 = z(119);

Cdk2_loci_frac = z(120);

Cdk2_constituitive = z(121);

cycE_loci_frac = z(122);

k_bind_cycE_to_Cdk2 = z(123);

k_unbind_cycE_Cdk2 = z(124);

k_bind_cycE_Cdk2_to_p27 = z(125);

k_unbind_cycE_Cdk2_p27 = z(126);

k_phospho_cycE_Cdk2 = z(127);

k_dephospho_cycE_Cdk2 = z(128);

k_dephospho_cycE_Cdk2_by_Cdc25A = z(129);

k_u_cycE_by_SCF_Skp2 = z(130);

k_u_cycE_by_SCF_Fbw7 = z(131);

B_Myb_loci_frac = z(132);

k_phospho_B_Myb_by_cyc_Cdk = z(133);

k_unphospho_B_Myb = z(134);

sat_B_Myb_on_DNA = z(135);

k_bind_B_Myb_to_DNA = z(136);

k_unbind_B_Myb_from_DNA = z(137);

k_u_B_Myb_by_SCF_Skp2 = z(138);

NF_Y_loci_frac = z(139);

NF_Y_constitutive = z(140);

k_phospho_NF_Y_by_cyc_Cdk = z(141);

k_unphospho_NF_Y = z(142);

k_dephospho_NF_Y_by_Cdc14 = z(143);

sat_NF_Y_on_DNA = z(144);

k_bind_NF_Y_to_DNA = z(145);

k_unbind_NF_Y_from_DNA = z(146);

E2F_loci_frac = z(147);

E2F_constituitive = z(148);

sat_E2F_on_DNA = z(149);

k_bind_E2F_to_DNA = z(150);

k_unbind_E2F_DNA = z(151);

k_E2F_phospho_by_cycA_Cdk2 = z(152);

k_E2F_phospho_by_cycA_Cdk1 = z(153);

k_E2F_phospho_by_cycB_Cdk1 = z(154);

k_E2F_phospho_by_cycD_Cdk4or6 = z(155);

k_u_E2F_by_SCF_Skp2 = z(156);

k_u_E2F_by_APC_Cdc20 = z(157);

adhesion_factor = z(158);

cycA_loci_frac = z(159);

k_bind_cycA_to_Cdk2 = z(160);

k_unbind_cycA_Cdk2 = z(161);

k_dephospho_cycA_Cdk2_by_Cdc25A = z(162);

k_phospho_cycA_Cdk2 = z(163);

k_bind_cycA_Cdk2_to_p27 = z(164);

k_unbind_cycA_Cdk2_p27 = z(165);

k_bind_cycA_to_Cdk1 = z(166);

k_unbind_cycA_Cdk1 = z(167);

k_phospho_cycA_Cdk1_by_Wee1 = z(168);

k_phospho_cycA_Cdk1_by_phospho_Wee1 = z(169);

k_unphospho_cycA_Cdk1_from_Wee1 = z(170);

k_dephospho_cycA_Cdk1_by_Cdc25A = z(171);

k_dephospho_cycA_Cdk1_by_Cdc25B = z(172);

k_dephospho_cycA_Cdk1_by_Cdc25C = z(173);

k_nm_in_cycA_Cdk1_by_Plk1 = z(174);

k_nm_out_cycA_Cdk1 = z(175);

k_u_cycA_by_SCF_Skp2 = z(176);

k_u_cycA_by_APC_Cdc20 = z(177);

k_u_cycA_by_APC_Cdh1 = z(178);

SCF_loci_frac = z(179);

k_u_SCF_in_cell_by_APC_Cdh1 = z(180);

k_u_SCF_active_by_APC_Cdh1 = z(181);

Skp2_loci_frac = z(182);

k_bind_Skp2_to_SCF = z(183);

k_unbind_Skp2_SCF = z(184);

k_u_Skp2_by_APC_Cdh1 = z(185);

k_u_auto_Skp2 = z(186);

Btrc_loci_frac = z(187);

k_bind_Btrc_to_SCF = z(188);

k_unbind_Btrc_SCF = z(189);

k_u_Btrc_by_APC_Cdh1 = z(190);

k_u_auto_Btrc = z(191);

Fbw7_loci_frac = z(192);

k_bind_Fbw7_to_SCF = z(193);

k_unbind_Fbw7_SCF = z(194);

k_u_auto_Fbw7 = z(195);

TF_grow_loci_frac = z(196);

TF_grow_txs_by_mitogen = z(197);

k_TF_grow_txs_by_Skp2 = z(198);

sat_TF_grow_on_DNA = z(199);

k_bind_TF_grow_to_DNA = z(200);

k_unbind_TF_grow_DNA = z(201);

k_u_TF_grow_by_SCF_Fbw7 = z(202);

k_u_TF_grow_by_SCF_Skp2 = z(203);

ATP_DNA_repl = z(204);

ATP_NT_txs = z(205);

x_DNA = z(206);

i_ER_DNA = z(207);

k_ER_DNA_constant = z(208);

k_div_DNA_factor = z(209);

x_RC = z(210);

x_RC_frag = z(211);

x_NT_RC_frag = z(212);

x_DNA_poly_per_RC = z(213);

RC_loci_frac = z(214);

k_bind_RC_to_DNA = z(215);

k_unbind_RC_DNA = z(216);

k_phospho_RC_by_cycD_Cdk4or6 = z(217);

k_phospho_RC_by_cycD_Cdk4or6_on_p27 = z(218);

k_phospho_RC_by_cycE_Cdk2 = z(219);

k_phospho_RC_by_cycA_Cdk2 = z(220);

k_u_RC_by_SCF_Fbw7 = z(221);

k_u_RC_by_SCF_Skp2 = z(222);

k_u_RC_by_APC_Cdh1 = z(223);

k_clear_RC_count = z(224);

DNA_poly_loci_frac = z(225);

k_bind_DNA_poly_to_DNA = z(226);

sat_DNA_poly = z(227);

Wee1_loci_frac = z(228);

k_phospho_Wee1_by_cycA_Cdk2 = z(229);

k_phospho_Wee1_by_cycA_Cdk1 = z(230);

k_phospho_Wee1_by_cycB_Cdk1 = z(231);

k_phospho_Wee1_by_Plk1 = z(232);

k_unphospho_Wee1 = z(233);

k_dephospho_Wee1_by_Cdc14 = z(234);

k_u_Wee1_by_SCF_Btrc = z(235);

k_u_Wee1_by_SCF_Skp2_test = z(236);

cycB_loci_frac = z(237);

k_bind_cycB_to_Cdk1 = z(238);

k_unbind_cycB_Cdk1 = z(239);

k_phospho_cycB_Cdk1_by_Wee1 = z(240);

k_phospho_cycB_Cdk1_by_phospho_Wee1 = z(241);

k_unphospho_cycB_Cdk1_from_Wee1 = z(242);

k_dephospho_cycB_Cdk1_by_Cdc25A = z(243);

k_dephospho_cycB_Cdk1_by_Cdc25B = z(244);

k_dephospho_cycB_Cdk1_by_Cdc25C = z(245);

k_nm_in_cycB_Cdk1_by_Plk1 = z(246);

k_nm_out_cycB_Cdk1 = z(247);

k_u_cycB_by_APC_Cdc20 = z(248);

k_u_cycB_by_APC_Cdh1 = z(249);

Cdk1_loci_frac = z(250);

Cdk1_constitutive = z(251);

k_activate_Cdk1_by_B_Myb_and_NF_Y = z(252);

k_deactivate_Cdk1 = z(253);

k_dephospho_Cdk1_by_Cdc14 = z(254);

Cdc25C_loci_frac = z(255);

k_phospho_Cdc25C_by_cycA_Cdk1 = z(256);

k_phospho_Cdc25C_by_cycB_Cdk1 = z(257);

k_phospho_Cdc25C_by_Plk1 = z(258);

k_dephospho_Cdc25C = z(259);

k_unphospho_Cdc25C = z(260);

k_nm_in_Cdc25C = z(261);

k_nm_out_Cdc25C = z(262);

k_dephospho_Cdc25C_by_Cdc14 = z(263);

Plk1_loci_frac = z(264);

k_phospho_Plk1_by_cycA_Cdk1 = z(265);

k_phospho_Plk1_by_cycB_Cdk1 = z(266);

k_unphospho_Plk1 = z(267);

k_nm_in_Plk1 = z(268);

k_u_Plk1_by_APC_Cdh1 = z(269);

Emi1_loci_frac = z(270);

k_bind_Emi1_to_Cdh1 = z(271);

k_unbind_Emi1_Cdh1 = z(272);

k_bind_Emi1_to_Cdc20 = z(273);

k_unbind_Emi1_Cdc20 = z(274);

k_phospho_Emi1_by_cycA_Cdk1 = z(275);

k_phospho_Emi1_by_cycB_Cdk1 = z(276);

k_u_Emi1_by_SCF_Btrc = z(277);

k_u_Emi1_by_SCF_Skp2_test = z(278);

APC_loci_frac = z(279);

Cdh1_loci_frac = z(280);

k_phospho_Cdh1_by_cycD_Cdk4or6 = z(281);

k_phospho_Cdh1_by_cycD_Cdk4or6_on_p27 = z(282);

k_phospho_Cdh1_by_cycE_Cdk2 = z(283);

k_phospho_Cdh1_by_cycA_Cdk2 = z(284);

k_phospho_Cdh1_by_Cdk1 = z(285);

k_dephospho_Cdh1_by_Cdc14 = z(286);

k_bind_Cdh1_to_APC = z(287);

k_unbind_Cdh1_APC = z(288);

k_u_auto_Cdh1_APC_active = z(289);

Cdc20_loci_frac = z(290);

k_bind_Cdc20_to_APC = z(291);

k_unbind_Cdc20_APC = z(292);

k_phospho_Cdc20_by_Cdk1 = z(293);

k_unphospho_Cdc20_APC_active = z(294);

k_dephospho_Cdc20_APC_by_Cdc14 = z(295);

k_u_Cdc20_by_APC_Cdh1 = z(296);

Cdc14_loci_frac = z(297);

k_phospho_Cdc14_by_Plk1 = z(298);

k_unphospho_Cdc14 = z(299);

k_inhibit_Cdc14_by_Securin = z(300);

k_uninhibit_Cdc14 = z(301);

Cdc25A_loci_frac = z(302);

Cdc25A_constitutive = z(303);

k_phospho_Cdc25A_by_Cdk2 = z(304);

k_phospho_Cdc25A_by_cycA_Cdk1 = z(305);

k_phospho_Cdc25A_by_cycB_Cdk1 = z(306);

k_dephospho_Cdc25A = z(307);

k_dephospho_Cdc25A_by_Cdc14 = z(308);

k_u_Cdc25A_by_APC_Cdh1 = z(309);

k_u_Cdc25A_by_SCF_Btrc = z(310);

Cdc25B_loci_frac = z(311);

k_phospho_Cdc25B_by_Cdk2 = z(312);

k_phospho_Cdc25B_by_cycA_Cdk1 = z(313);

k_phospho_Cdc25B_by_cycB_Cdk1 = z(314);

k_phospho_Cdc25B_by_TF_grow = z(315);

k_phospho_Cdc25B_by_Plk1 = z(316);

k_dephospho_Cdc25B = z(317);

k_dephospho_Cdc25B_by_Cdc14 = z(318);

k_u_Cdc25B_by_SCF_Btrc = z(319);

k_u_Cdc25B_by_APC_Cdh1 = z(320);

k_u_Cdc25B_by_APC_Cdh1 = z(321);

Securin_loci_frac = z(322);

k_bind_Securin_to_chromo = z(323);

k_u_Securin_by_APC_Cdc20 = z(324);

cycC_loci_frac = z(325);

k_bind_cycC_to_Cdk8 = z(326);

k_unbind_cycC_from_Cdk8 = z(327);

k_deactivate_cycC_Cdk8_by_mitogen = z(328);

k_activate_cycC_Cdk8_by_Cdc14 = z(329);

KPC_loci_frac = z(330);

k_activate_KPC_by_mitogen = z(331);

k_inactivate_KPC = z(332);

% !!!!!!!!!!!!!!!!!!!!!!!!!!!!!!! UNPACK INITIAL CONDITIONS

i_AA_in_cytosol = y0(1);

i_AA_in_p = y0(2);

i_AA_on_tRNA = y0(3);

i_ADP_in_cell = y0(4);

i_ADP_trash = y0(5);

i_ATP_in_cell = y0(6);

i_fats_cytosol = y0(7);

i_fats_in_mem = y0(8);

i_fats_in_vesicles = y0(9);

i_H_between_mito_mems = y0(10);

i_H_in_inner_mito_mems = y0(11);

i_junk_spl = y0(12);

i_junk_spl_mRNA = y0(13);

i_junk_spl_rRNA = y0(14);

i_junk_spl_snRNA = y0(15);

i_mRNA_cytosol = y0(16);

i_mRNA_in_use = y0(17);

i_mRNA_nuc = y0(18);

i_mRNA_inactive = y0(19);

i_mRNA_trash = y0(20);

i_mRNA_unspl = y0(21);

i_Na_in_cell = y0(22);

i_Na_out_cell = y0(23);

i_NT_in_cell = y0(24);

i_NT_in_RNA = y0(25);

i_p_in_cell = y0(26);

i_p_in_use = y0(27);

i_p_trash = y0(28);

i_p_unfold = y0(29);

i_rRNA_cytosol = y0(30);

i_rRNA_in_use = y0(31);

i_rRNA_nuc = y0(32);

i_rRNA_trash = y0(33);

i_rRNA_unspl = y0(34);

i_snRNA_in_use = y0(35);

i_snRNA_nuc = y0(36);

i_snRNA_trash = y0(37);

i_snRNA_unspl = y0(38);

i_sugar_in_cell = y0(39);

i_tRNA_cytosol = y0(40);

i_tRNA_in_use = y0(41);

i_tRNA_nuc = y0(42);

i_tRNA_trash = y0(43);

i_mRNA_poly_unspl = y0(44);

i_mRNA_poly_nuc = y0(45);

i_mRNA_poly_inactive = y0(46);

i_mRNA_poly_cytosol = y0(47);

i_mRNA_poly_in_use = y0(48);

i_mRNA_poly_trash = y0(49);

i_p_poly_unfold = y0(50);

i_p_poly_cytosol = y0(51);

i_p_poly_nuc_inactive = y0(52);

i_p_poly_nuc_active = y0(53);

i_p_poly_in_use = y0(54);

i_p_poly_trash = y0(55);

i_mRNA_eIF_4_unspl = y0(56);

i_mRNA_eIF_4_nuc = y0(57);

i_mRNA_eIF_4_inactive = y0(58);

i_mRNA_eIF_4_cytosol = y0(59);

i_mRNA_eIF_4_in_use = y0(60);

i_mRNA_eIF_4_trash = y0(61);

i_p_eIF_4_unfold = y0(62);

i_p_eIF_4_cytosol = y0(63);

i_p_eIF_4_in_use = y0(64);

i_p_eIF_4_trash = y0(65);

i_mRNA_p27_unspl = y0(66);

i_mRNA_p27_nuc = y0(67);

i_mRNA_p27_inactive = y0(68);

i_mRNA_p27_cytosol = y0(69);

i_mRNA_p27_in_use = y0(70);

i_mRNA_p27_trash = y0(71);

i_p27_unfold = y0(72);

i_p27_cytosol = y0(73);

i_p27_nuc = y0(74);

i_p27_on_cyclins = y0(75);

i_p27_trash = y0(76);

i_mRNA_Rb_unspl = y0(77);

i_mRNA_Rb_nuc = y0(78);

i_mRNA_Rb_inactive = y0(79);

i_mRNA_Rb_cytosol = y0(80);

i_mRNA_Rb_in_use = y0(81);

i_mRNA_Rb_trash = y0(82);

i_Rb_unfold = y0(83);

i_Rb_cytosol = y0(84);

i_Rb_nuc = y0(85);

i_Rb_on_E2F = y0(86);

i_Rb_phospho_inactive = y0(87);

i_Rb_trash = y0(88);

i_mRNA_cycD_unspl = y0(89);

i_mRNA_cycD_nuc = y0(90);

i_mRNA_cycD_inactive = y0(91);

i_mRNA_cycD_cytosol = y0(92);

i_mRNA_cycD_in_use = y0(93);

i_mRNA_cycD_trash = y0(94);

i_cycD_unfold = y0(95);

i_cycD_cytosol = y0(96);

i_cycD_nuc = y0(97);

i_cycD_Cdk4or6_active = y0(98);

i_cycD_Cdk4or6_on_p27_inactive = y0(99);

i_cycD_trash = y0(100);

i_mRNA_Cdk2_unspl = y0(101);

i_mRNA_Cdk2_nuc = y0(102);

i_mRNA_Cdk2_inactive = y0(103);

i_mRNA_Cdk2_cytosol = y0(104);

i_mRNA_Cdk2_in_use = y0(105);

i_mRNA_Cdk2_trash = y0(106);

i_Cdk2_unfold = y0(107);

i_Cdk2_cytosol = y0(108);

i_Cdk2_nuc = y0(109);

i_Cdk2_on_cycE = y0(110);

i_Cdk2_on_cycA = y0(111);

i_Cdk2_trash = y0(112);

i_mRNA_cycE_unspl = y0(113);

i_mRNA_cycE_nuc = y0(114);

i_mRNA_cycE_inactive = y0(115);

i_mRNA_cycE_cytosol = y0(116);

i_mRNA_cycE_in_use = y0(117);

i_mRNA_cycE_trash = y0(118);

i_cycE_unfold = y0(119);

i_cycE_cytosol = y0(120);

i_cycE_nuc = y0(121);

i_cycE_Cdk2_inactive = y0(122);

i_cycE_Cdk2_active = y0(123);

i_cycE_Cdk2_on_p27_inactive = y0(124);

i_cycE_trash = y0(125);

i_mRNA_B_Myb_unspl = y0(126);

i_mRNA_B_Myb_nuc = y0(127);

i_mRNA_B_Myb_inactive = y0(128);

i_mRNA_B_Myb_cytosol = y0(129);

i_mRNA_B_Myb_in_use = y0(130);

i_mRNA_B_Myb_trash = y0(131);

i_B_Myb_unfold = y0(132);

i_B_Myb_cytosol = y0(133);

i_B_Myb_nuc = y0(134);

i_B_Myb_phospho_active = y0(135);

i_B_Myb_on_DNA = y0(136);

i_B_Myb_trash = y0(137);

i_mRNA_NF_Y_unspl = y0(138);

i_mRNA_NF_Y_nuc = y0(139);

i_mRNA_NF_Y_inactive = y0(140);

i_mRNA_NF_Y_cytosol = y0(141);

i_mRNA_NF_Y_in_use = y0(142);

i_mRNA_NF_Y_trash = y0(143);

i_NF_Y_unfold = y0(144);

i_NF_Y_cytosol = y0(145);

i_NF_Y_nuc = y0(146);

i_NF_Y_phospho_active = y0(147);

i_NF_Y_on_DNA = y0(148);

i_NF_Y_trash = y0(149);

i_mRNA_E2F_unspl = y0(150);

i_mRNA_E2F_nuc = y0(151);

i_mRNA_E2F_inactive = y0(152);

i_mRNA_E2F_cytosol = y0(153);

i_mRNA_E2F_in_use = y0(154);

i_mRNA_E2F_trash = y0(155);

i_E2F_unfold = y0(156);

i_E2F_cytosol = y0(157);

i_E2F_nuc = y0(158);

i_E2F_Rb_inactive = y0(159);

i_E2F_on_DNA = y0(160);

i_E2F_trash = y0(161);

i_mRNA_cycA_unspl = y0(162);

i_mRNA_cycA_nuc = y0(163);

i_mRNA_cycA_inactive = y0(164);

i_mRNA_cycA_cytosol = y0(165);

i_mRNA_cycA_in_use = y0(166);

i_mRNA_cycA_trash = y0(167);

i_cycA_unfold = y0(168);

i_cycA_cytosol = y0(169);

i_cycA_nuc = y0(170);

i_cycA_Cdk2_inactive = y0(171);

i_cycA_Cdk2_on_p27_inactive = y0(172);

i_cycA_Cdk2_active = y0(173);

i_cycA_Cdk1_cytosol_active = y0(174);

i_cycA_Cdk1_phospho_inactive = y0(175);

i_cycA_Cdk1_nuc_active = y0(176);

i_cycA_trash = y0(177);

i_mRNA_SCF_unspl = y0(178);

i_mRNA_SCF_nuc = y0(179);

i_mRNA_SCF_inactive = y0(180);

i_mRNA_SCF_cytosol = y0(181);

i_mRNA_SCF_in_use = y0(182);

i_mRNA_SCF_trash = y0(183);

i_SCF_unfold = y0(184);

i_SCF_in_cell = y0(185);

i_SCF_on_Btrc = y0(186);

i_SCF_on_Fbw7 = y0(187);

i_SCF_on_Skp2 = y0(188);

i_SCF_trash = y0(189);

i_mRNA_Skp2_unspl = y0(190);

i_mRNA_Skp2_nuc = y0(191);

i_mRNA_Skp2_inactive = y0(192);

i_mRNA_Skp2_cytosol = y0(193);

i_mRNA_Skp2_in_use = y0(194);

i_mRNA_Skp2_trash = y0(195);

i_Skp2_unfold = y0(196);

i_Skp2_cytosol = y0(197);

i_Skp2_nuc = y0(198);

i_Skp2_SCF_active = y0(199);

i_Skp2_trash = y0(200);

i_mRNA_Btrc_unspl = y0(201);

i_mRNA_Btrc_nuc = y0(202);

i_mRNA_Btrc_inactive = y0(203);

i_mRNA_Btrc_cytosol = y0(204);

i_mRNA_Btrc_in_use = y0(205);

i_mRNA_Btrc_trash = y0(206);

i_Btrc_unfold = y0(207);

i_Btrc_cytosol = y0(208);

i_Btrc_SCF_active = y0(209);

i_Btrc_trash = y0(210);

i_mRNA_Fbw7_unspl = y0(211);

i_mRNA_Fbw7_nuc = y0(212);

i_mRNA_Fbw7_inactive = y0(213);

i_mRNA_Fbw7_cytosol = y0(214);

i_mRNA_Fbw7_in_use = y0(215);

i_mRNA_Fbw7_trash = y0(216);

i_Fbw7_unfold = y0(217);

i_Fbw7_cytosol = y0(218);

i_Fbw7_nuc = y0(219);

i_Fbw7_SCF_active = y0(220);

i_Fbw7_trash = y0(221);

i_mRNA_TF_grow_unspl = y0(222);

i_mRNA_TF_grow_inactive = y0(223);

i_mRNA_TF_grow_cytosol = y0(224);

i_mRNA_TF_grow_nuc = y0(225);

i_mRNA_TF_grow_in_use = y0(226);

i_mRNA_TF_grow_trash = y0(227);

i_TF_grow_unfold = y0(228);

i_TF_grow_cytosol = y0(229);

i_TF_grow_nuc = y0(230);

i_TF_grow_on_DNA = y0(231);

i_TF_grow_trash = y0(232);

i_NT_in_DNA = y0(233);

i_mRNA_RC_unspl = y0(234);

i_mRNA_RC_nuc = y0(235);

i_mRNA_RC_inactive = y0(236);

i_mRNA_RC_cytosol = y0(237);

i_mRNA_RC_in_use = y0(238);

i_mRNA_RC_trash = y0(239);

i_RC_unfold = y0(240);

i_RC_cytosol = y0(241);

i_RC_nuc = y0(242);

i_RC_on_DNA = y0(243);

i_RC_licensed = y0(244);

i_RC_traversed_by_DNA_poly = y0(245);

i_RC_trash = y0(246);

i_RC_count = y0(247);

i_mRNA_DNA_poly_unspl = y0(248);

i_mRNA_DNA_poly_nuc = y0(249);

i_mRNA_DNA_poly_inactive = y0(250);

i_mRNA_DNA_poly_cytosol = y0(251);

i_mRNA_DNA_poly_in_use = y0(252);

i_mRNA_DNA_poly_trash = y0(253);

i_DNA_poly_unfold = y0(254);

i_DNA_poly_cytosol = y0(255);

i_DNA_poly_nuc = y0(256);

i_DNA_poly_on_DNA = y0(257);

i_DNA_poly_trash = y0(258);

i_mRNA_Wee1_unspl = y0(259);

i_mRNA_Wee1_nuc = y0(260);

i_mRNA_Wee1_inactive = y0(261);

i_mRNA_Wee1_cytosol = y0(262);

i_mRNA_Wee1_in_use = y0(263);

i_mRNA_Wee1_trash = y0(264);

i_Wee1_unfold = y0(265);

i_Wee1_cytosol_active = y0(266);

i_Wee1_phospho_inactive = y0(267);

i_Wee1_trash = y0(268);

i_mRNA_cycB_unspl = y0(269);

i_mRNA_cycB_nuc = y0(270);

i_mRNA_cycB_inactive = y0(271);

i_mRNA_cycB_cytosol = y0(272);

i_mRNA_cycB_in_use = y0(273);

i_mRNA_cycB_trash = y0(274);

i_cycB_unfold = y0(275);

i_cycB_cytosol = y0(276);

i_cycB_Cdk1_cytosol_active = y0(277);

i_cycB_Cdk1_phospho_inactive = y0(278);

i_cycB_Cdk1_nuc_active = y0(279);

i_cycB_trash = y0(280);

i_mRNA_Cdk1_unspl = y0(281);

i_mRNA_Cdk1_nuc = y0(282);

i_mRNA_Cdk1_inactive = y0(283);

i_mRNA_Cdk1_cytosol = y0(284);

i_mRNA_Cdk1_in_use = y0(285);

i_mRNA_Cdk1_trash = y0(286);

i_Cdk1_unfold = y0(287);

i_Cdk1_inactive = y0(288);

i_Cdk1_in_cell = y0(289);

i_Cdk1_on_cycB = y0(290);

i_Cdk1_on_cycA = y0(291);

i_Cdk1_trash = y0(292);

i_mRNA_Cdc25C_unspl = y0(293);

i_mRNA_Cdc25C_nuc = y0(294);

i_mRNA_Cdc25C_inactive = y0(295);

i_mRNA_Cdc25C_cytosol = y0(296);

i_mRNA_Cdc25C_in_use = y0(297);

i_mRNA_Cdc25C_trash = y0(298);

i_Cdc25C_unfold = y0(299);

i_Cdc25C_cytosol_inactive = y0(300);

i_Cdc25C_cytosol_phospho_active = y0(301);

i_Cdc25C_nuc_phospho_active = y0(302);

i_Cdc25C_trash = y0(303);

i_mRNA_Plk1_unspl = y0(304);

i_mRNA_Plk1_nuc = y0(305);

i_mRNA_Plk1_inactive = y0(306);

i_mRNA_Plk1_cytosol = y0(307);

i_mRNA_Plk1_in_use = y0(308);

i_mRNA_Plk1_trash = y0(309);

i_Plk1_unfold = y0(310);

i_Plk1_cytosol_inactive = y0(311);

i_Plk1_cytosol_phospho_active = y0(312);

i_Plk1_nuc_phospho_active = y0(313);

i_Plk1_trash = y0(314);

i_mRNA_Emi1_unspl = y0(315);

i_mRNA_Emi1_nuc = y0(316);

i_mRNA_Emi1_inactive = y0(317);

i_mRNA_Emi1_cytosol = y0(318);

i_mRNA_Emi1_in_use = y0(319);

i_mRNA_Emi1_trash = y0(320);

i_Emi1_unfold = y0(321);

i_Emi1_in_cell = y0(322);

i_Emi1_on_Cdh1 = y0(323);

i_Emi1_on_Cdc20 = y0(324);

i_Emi1_phospho_inactive = y0(325);

i_Emi1_trash = y0(326);

i_mRNA_APC_unspl = y0(327);

i_mRNA_APC_nuc = y0(328);

i_mRNA_APC_inactive = y0(329);

i_mRNA_APC_cytosol = y0(330);

i_mRNA_APC_in_use = y0(331);

i_mRNA_APC_trash = y0(332);

i_APC_unfold = y0(333);

i_APC_in_cell = y0(334);

i_APC_on_Cdh1 = y0(335);

i_APC_on_Cdc20 = y0(336);

i_APC_trash = y0(337);

i_mRNA_Cdh1_unspl = y0(338);

i_mRNA_Cdh1_nuc = y0(339);

i_mRNA_Cdh1_inactive = y0(340);

i_mRNA_Cdh1_cytosol = y0(341);

i_mRNA_Cdh1_in_use = y0(342);

i_mRNA_Cdh1_trash = y0(343);

i_Cdh1_unfold = y0(344);

i_Cdh1_in_cell = y0(345);

i_Cdh1_Emi1_inactive = y0(346);

i_Cdh1_phospho_inactive = y0(347);

i_Cdh1_APC_active = y0(348);

i_Cdh1_trash = y0(349);

i_mRNA_Cdc20_unspl = y0(350);

i_mRNA_Cdc20_nuc = y0(351);

i_mRNA_Cdc20_inactive = y0(352);

i_mRNA_Cdc20_cytosol = y0(353);

i_mRNA_Cdc20_in_use = y0(354);

i_mRNA_Cdc20_trash = y0(355);

i_Cdc20_unfold = y0(356);

i_Cdc20_cytosol = y0(357);

i_Cdc20_nuc = y0(358);

i_Cdc20_Emi1_inactive = y0(359);

i_Cdc20_APC_inactive = y0(360);

i_Cdc20_APC_active = y0(361);

i_Cdc20_trash = y0(362);

i_mRNA_Cdc14_unspl = y0(363);

i_mRNA_Cdc14_nuc = y0(364);

i_mRNA_Cdc14_inactive = y0(365);

i_mRNA_Cdc14_cytosol = y0(366);

i_mRNA_Cdc14_in_use = y0(367);

i_mRNA_Cdc14_trash = y0(368);

i_Cdc14_unfold = y0(369);

i_Cdc14_cytosol = y0(370);

i_Cdc14_nuc_inactive = y0(371);

i_Cdc14_inhibited = y0(372);

i_Cdc14_phospho_active = y0(373);

i_Cdc14_trash = y0(374);

i_mRNA_Cdc25A_unspl = y0(375);

i_mRNA_Cdc25A_nuc = y0(376);

i_mRNA_Cdc25A_inactive = y0(377);

i_mRNA_Cdc25A_cytosol = y0(378);

i_mRNA_Cdc25A_in_use = y0(379);

i_mRNA_Cdc25A_trash = y0(380);

i_Cdc25A_unfold = y0(381);

i_Cdc25A_cytosol = y0(382);

i_Cdc25A_nuc_inactive = y0(383);

i_Cdc25A_phospho_active = y0(384);

i_Cdc25A_trash = y0(385);

i_mRNA_Cdc25B_unspl = y0(386);

i_mRNA_Cdc25B_nuc = y0(387);

i_mRNA_Cdc25B_inactive = y0(388);

i_mRNA_Cdc25B_cytosol = y0(389);

i_mRNA_Cdc25B_in_use = y0(390);

i_mRNA_Cdc25B_trash = y0(391);

i_Cdc25B_unfold = y0(392);

i_Cdc25B_cytosol_inactive = y0(393);

i_Cdc25B_cytosol_phospho_active = y0(394);

i_Cdc25B_trash = y0(395);

i_mRNA_Securin_unspl = y0(396);

i_mRNA_Securin_nuc = y0(397);

i_mRNA_Securin_inactive = y0(398);

i_mRNA_Securin_cytosol = y0(399);

i_mRNA_Securin_in_use = y0(400);

i_mRNA_Securin_trash = y0(401);

i_Securin_unfold = y0(402);

i_Securin_cytosol = y0(403);

i_Securin_nuc = y0(404);

i_Securin_on_chromo = y0(405);

i_Securin_trash = y0(406);

i_mRNA_cycC_unspl = y0(407);

i_mRNA_cycC_nuc = y0(408);

i_mRNA_cycC_inactive = y0(409);

i_mRNA_cycC_cytosol = y0(410);

i_mRNA_cycC_in_use = y0(411);

i_mRNA_cycC_trash = y0(412);

i_cycC_unfold = y0(413);

i_cycC_cytosol = y0(414);

i_cycC_nuc = y0(415);

i_cycC_Cdk8_active = y0(416);

i_cycC_Cdk8_inactive = y0(417);

i_cycC_trash = y0(418);

i_mRNA_KPC_unspl = y0(419);

i_mRNA_KPC_nuc = y0(420);

i_mRNA_KPC_inactive = y0(421);

i_mRNA_KPC_cytosol = y0(422);

i_mRNA_KPC_in_use = y0(423);

i_mRNA_KPC_trash = y0(424);

i_KPC_unfold = y0(425);

i_KPC_in_cell = y0(426);

i_KPC_active = y0(427);

i_KPC_trash = y0(428);

% !!!!!!!!!!!!!!!!!!!!!!!!!!!!!!!!!!!!!!!!!! INITIALIZATION

AA_in_cytosol = y(ylength,1);

AA_in_p = y(ylength,2);

AA_on_tRNA = y(ylength,3);

ADP_in_cell = y(ylength,4);

ADP_trash = y(ylength,5);

ATP_in_cell = y(ylength,6);

fats_cytosol = y(ylength,7);

fats_in_mem = y(ylength,8);

fats_in_vesicles = y(ylength,9);

H_between_mito_mems = y(ylength,10);

H_in_inner_mito_mems = y(ylength,11);

junk_spl = y(ylength,12);

junk_spl_mRNA = y(ylength,13);

junk_spl_rRNA = y(ylength,14);

junk_spl_snRNA = y(ylength,15);

mRNA_cytosol = y(ylength,16);

mRNA_in_use = y(ylength,17);

mRNA_nuc = y(ylength,18);

mRNA_inactive = y(ylength,19);

mRNA_trash = y(ylength,20);

mRNA_unspl = y(ylength,21);

Na_in_cell = y(ylength,22);

Na_out_cell = y(ylength,23);

NT_in_cell = y(ylength,24);

NT_in_RNA = y(ylength,25);

p_in_cell = y(ylength,26);

p_in_use = y(ylength,27);

p_trash = y(ylength,28);

p_unfold = y(ylength,29);

rRNA_cytosol = y(ylength,30);

rRNA_in_use = y(ylength,31);

rRNA_nuc = y(ylength,32);

rRNA_trash = y(ylength,33);

rRNA_unspl = y(ylength,34);

snRNA_in_use = y(ylength,35);

snRNA_nuc = y(ylength,36);

snRNA_trash = y(ylength,37);

snRNA_unspl = y(ylength,38);

sugar_in_cell = y(ylength,39);

tRNA_cytosol = y(ylength,40);

tRNA_in_use = y(ylength,41);

tRNA_nuc = y(ylength,42);

tRNA_trash = y(ylength,43);

mRNA_poly_unspl = y(ylength,44);

mRNA_poly_nuc = y(ylength,45);

mRNA_poly_inactive = y(ylength,46);

mRNA_poly_cytosol = y(ylength,47);

mRNA_poly_in_use = y(ylength,48);

mRNA_poly_trash = y(ylength,49);

p_poly_unfold = y(ylength,50);

p_poly_cytosol = y(ylength,51);

p_poly_nuc_inactive = y(ylength,52);

p_poly_nuc_active = y(ylength,53);

p_poly_in_use = y(ylength,54);

p_poly_trash = y(ylength,55);

mRNA_eIF_4_unspl = y(ylength,56);

mRNA_eIF_4_nuc = y(ylength,57);

mRNA_eIF_4_inactive = y(ylength,58);

mRNA_eIF_4_cytosol = y(ylength,59);

mRNA_eIF_4_in_use = y(ylength,60);

mRNA_eIF_4_trash = y(ylength,61);

p_eIF_4_unfold = y(ylength,62);

p_eIF_4_cytosol = y(ylength,63);

p_eIF_4_in_use = y(ylength,64);

p_eIF_4_trash = y(ylength,65);

mRNA_p27_unspl = y(ylength,66);

mRNA_p27_nuc = y(ylength,67);

mRNA_p27_inactive = y(ylength,68);

mRNA_p27_cytosol = y(ylength,69);

mRNA_p27_in_use = y(ylength,70);

mRNA_p27_trash = y(ylength,71);

p27_unfold = y(ylength,72);

p27_cytosol = y(ylength,73);

p27_nuc = y(ylength,74);

p27_on_cyclins = y(ylength,75);

p27_trash = y(ylength,76);

mRNA_Rb_unspl = y(ylength,77);

mRNA_Rb_nuc = y(ylength,78);

mRNA_Rb_inactive = y(ylength,79);

mRNA_Rb_cytosol = y(ylength,80);

mRNA_Rb_in_use = y(ylength,81);

mRNA_Rb_trash = y(ylength,82);

Rb_unfold = y(ylength,83);

Rb_cytosol = y(ylength,84);

Rb_nuc = y(ylength,85);

Rb_on_E2F = y(ylength,86);

Rb_phospho_inactive = y(ylength,87);

Rb_trash = y(ylength,88);

mRNA_cycD_unspl = y(ylength,89);

mRNA_cycD_nuc = y(ylength,90);

mRNA_cycD_inactive = y(ylength,91);

mRNA_cycD_cytosol = y(ylength,92);

mRNA_cycD_in_use = y(ylength,93);

mRNA_cycD_trash = y(ylength,94);

cycD_unfold = y(ylength,95);

cycD_cytosol = y(ylength,96);

cycD_nuc = y(ylength,97);

cycD_Cdk4or6_active = y(ylength,98);

cycD_Cdk4or6_on_p27_inactive = y(ylength,99);

cycD_trash = y(ylength,100);

mRNA_Cdk2_unspl = y(ylength,101);

mRNA_Cdk2_nuc = y(ylength,102);

mRNA_Cdk2_inactive = y(ylength,103);

mRNA_Cdk2_cytosol = y(ylength,104);

mRNA_Cdk2_in_use = y(ylength,105);

mRNA_Cdk2_trash = y(ylength,106);

Cdk2_unfold = y(ylength,107);

Cdk2_cytosol = y(ylength,108);

Cdk2_nuc = y(ylength,109);

Cdk2_on_cycE = y(ylength,110);

Cdk2_on_cycA = y(ylength,111);

Cdk2_trash = y(ylength,112);

mRNA_cycE_unspl = y(ylength,113);

mRNA_cycE_nuc = y(ylength,114);

mRNA_cycE_inactive = y(ylength,115);

mRNA_cycE_cytosol = y(ylength,116);

mRNA_cycE_in_use = y(ylength,117);

mRNA_cycE_trash = y(ylength,118);

cycE_unfold = y(ylength,119);

cycE_cytosol = y(ylength,120);

cycE_nuc = y(ylength,121);

cycE_Cdk2_inactive = y(ylength,122);

cycE_Cdk2_active = y(ylength,123);

cycE_Cdk2_on_p27_inactive = y(ylength,124);

cycE_trash = y(ylength,125);

mRNA_B_Myb_unspl = y(ylength,126);

mRNA_B_Myb_nuc = y(ylength,127);

mRNA_B_Myb_inactive = y(ylength,128);

mRNA_B_Myb_cytosol = y(ylength,129);

mRNA_B_Myb_in_use = y(ylength,130);

mRNA_B_Myb_trash = y(ylength,131);

B_Myb_unfold = y(ylength,132);

B_Myb_cytosol = y(ylength,133);

B_Myb_nuc = y(ylength,134);

B_Myb_phospho_active = y(ylength,135);

B_Myb_on_DNA = y(ylength,136);

B_Myb_trash = y(ylength,137);

mRNA_NF_Y_unspl = y(ylength,138);

mRNA_NF_Y_nuc = y(ylength,139);

mRNA_NF_Y_inactive = y(ylength,140);

mRNA_NF_Y_cytosol = y(ylength,141);

mRNA_NF_Y_in_use = y(ylength,142);

mRNA_NF_Y_trash = y(ylength,143);

NF_Y_unfold = y(ylength,144);

NF_Y_cytosol = y(ylength,145);

NF_Y_nuc = y(ylength,146);

NF_Y_phospho_active = y(ylength,147);

NF_Y_on_DNA = y(ylength,148);

NF_Y_trash = y(ylength,149);

mRNA_E2F_unspl = y(ylength,150);

mRNA_E2F_nuc = y(ylength,151);

mRNA_E2F_inactive = y(ylength,152);

mRNA_E2F_cytosol = y(ylength,153);

mRNA_E2F_in_use = y(ylength,154);

mRNA_E2F_trash = y(ylength,155);

E2F_unfold = y(ylength,156);

E2F_cytosol = y(ylength,157);

E2F_nuc = y(ylength,158);

E2F_Rb_inactive = y(ylength,159);

E2F_on_DNA = y(ylength,160);

E2F_trash = y(ylength,161);

mRNA_cycA_unspl = y(ylength,162);

mRNA_cycA_nuc = y(ylength,163);

mRNA_cycA_inactive = y(ylength,164);

mRNA_cycA_cytosol = y(ylength,165);

mRNA_cycA_in_use = y(ylength,166);

mRNA_cycA_trash = y(ylength,167);

cycA_unfold = y(ylength,168);

cycA_cytosol = y(ylength,169);

cycA_nuc = y(ylength,170);

cycA_Cdk2_inactive = y(ylength,171);

cycA_Cdk2_on_p27_inactive = y(ylength,172);

cycA_Cdk2_active = y(ylength,173);

cycA_Cdk1_cytosol_active = y(ylength,174);

cycA_Cdk1_phospho_inactive = y(ylength,175);

cycA_Cdk1_nuc_active = y(ylength,176);

cycA_trash = y(ylength,177);

mRNA_SCF_unspl = y(ylength,178);

mRNA_SCF_nuc = y(ylength,179);

mRNA_SCF_inactive = y(ylength,180);

mRNA_SCF_cytosol = y(ylength,181);

mRNA_SCF_in_use = y(ylength,182);

mRNA_SCF_trash = y(ylength,183);

SCF_unfold = y(ylength,184);

SCF_in_cell = y(ylength,185);

SCF_on_Btrc = y(ylength,186);

SCF_on_Fbw7 = y(ylength,187);

SCF_on_Skp2 = y(ylength,188);

SCF_trash = y(ylength,189);

mRNA_Skp2_unspl = y(ylength,190);

mRNA_Skp2_nuc = y(ylength,191);

mRNA_Skp2_inactive = y(ylength,192);

mRNA_Skp2_cytosol = y(ylength,193);

mRNA_Skp2_in_use = y(ylength,194);

mRNA_Skp2_trash = y(ylength,195);

Skp2_unfold = y(ylength,196);

Skp2_cytosol = y(ylength,197);

Skp2_nuc = y(ylength,198);

Skp2_SCF_active = y(ylength,199);

Skp2_trash = y(ylength,200);

mRNA_Btrc_unspl = y(ylength,201);

mRNA_Btrc_nuc = y(ylength,202);

mRNA_Btrc_inactive = y(ylength,203);

mRNA_Btrc_cytosol = y(ylength,204);

mRNA_Btrc_in_use = y(ylength,205);

mRNA_Btrc_trash = y(ylength,206);

Btrc_unfold = y(ylength,207);

Btrc_cytosol = y(ylength,208);

Btrc_SCF_active = y(ylength,209);

Btrc_trash = y(ylength,210);

mRNA_Fbw7_unspl = y(ylength,211);

mRNA_Fbw7_nuc = y(ylength,212);

mRNA_Fbw7_inactive = y(ylength,213);

mRNA_Fbw7_cytosol = y(ylength,214);

mRNA_Fbw7_in_use = y(ylength,215);

mRNA_Fbw7_trash = y(ylength,216);

Fbw7_unfold = y(ylength,217);

Fbw7_cytosol = y(ylength,218);

Fbw7_nuc = y(ylength,219);

Fbw7_SCF_active = y(ylength,220);

Fbw7_trash = y(ylength,221);

mRNA_TF_grow_unspl = y(ylength,222);

mRNA_TF_grow_inactive = y(ylength,223);

mRNA_TF_grow_cytosol = y(ylength,224);

mRNA_TF_grow_nuc = y(ylength,225);

mRNA_TF_grow_in_use = y(ylength,226);

mRNA_TF_grow_trash = y(ylength,227);

TF_grow_unfold = y(ylength,228);

TF_grow_cytosol = y(ylength,229);

TF_grow_nuc = y(ylength,230);

TF_grow_on_DNA = y(ylength,231);

TF_grow_trash = y(ylength,232);

NT_in_DNA = y(ylength,233);

mRNA_RC_unspl = y(ylength,234);

mRNA_RC_nuc = y(ylength,235);

mRNA_RC_inactive = y(ylength,236);

mRNA_RC_cytosol = y(ylength,237);

mRNA_RC_in_use = y(ylength,238);

mRNA_RC_trash = y(ylength,239);

RC_unfold = y(ylength,240);

RC_cytosol = y(ylength,241);

RC_nuc = y(ylength,242);

RC_on_DNA = y(ylength,243);

RC_licensed = y(ylength,244);

RC_traversed_by_DNA_poly = y(ylength,245);

RC_trash = y(ylength,246);

RC_count = y(ylength,247);

mRNA_DNA_poly_unspl = y(ylength,248);

mRNA_DNA_poly_nuc = y(ylength,249);

mRNA_DNA_poly_inactive = y(ylength,250);

mRNA_DNA_poly_cytosol = y(ylength,251);

mRNA_DNA_poly_in_use = y(ylength,252);

mRNA_DNA_poly_trash = y(ylength,253);

DNA_poly_unfold = y(ylength,254);

DNA_poly_cytosol = y(ylength,255);

DNA_poly_nuc = y(ylength,256);

DNA_poly_on_DNA = y(ylength,257);

DNA_poly_trash = y(ylength,258);

mRNA_Wee1_unspl = y(ylength,259);

mRNA_Wee1_nuc = y(ylength,260);

mRNA_Wee1_inactive = y(ylength,261);

mRNA_Wee1_cytosol = y(ylength,262);

mRNA_Wee1_in_use = y(ylength,263);

mRNA_Wee1_trash = y(ylength,264);

Wee1_unfold = y(ylength,265);

Wee1_cytosol_active = y(ylength,266);

Wee1_phospho_inactive = y(ylength,267);

Wee1_trash = y(ylength,268);

mRNA_cycB_unspl = y(ylength,269);

mRNA_cycB_nuc = y(ylength,270);

mRNA_cycB_inactive = y(ylength,271);

mRNA_cycB_cytosol = y(ylength,272);

mRNA_cycB_in_use = y(ylength,273);

mRNA_cycB_trash = y(ylength,274);

cycB_unfold = y(ylength,275);

cycB_cytosol = y(ylength,276);

cycB_Cdk1_cytosol_active = y(ylength,277);

cycB_Cdk1_phospho_inactive = y(ylength,278);

cycB_Cdk1_nuc_active = y(ylength,279);

cycB_trash = y(ylength,280);

mRNA_Cdk1_unspl = y(ylength,281);

mRNA_Cdk1_nuc = y(ylength,282);

mRNA_Cdk1_inactive = y(ylength,283);

mRNA_Cdk1_cytosol = y(ylength,284);

mRNA_Cdk1_in_use = y(ylength,285);

mRNA_Cdk1_trash = y(ylength,286);

Cdk1_unfold = y(ylength,287);

Cdk1_inactive = y(ylength,288);

Cdk1_in_cell = y(ylength,289);

Cdk1_on_cycB = y(ylength,290);

Cdk1_on_cycA = y(ylength,291);

Cdk1_trash = y(ylength,292);

mRNA_Cdc25C_unspl = y(ylength,293);

mRNA_Cdc25C_nuc = y(ylength,294);

mRNA_Cdc25C_inactive = y(ylength,295);

mRNA_Cdc25C_cytosol = y(ylength,296);

mRNA_Cdc25C_in_use = y(ylength,297);

mRNA_Cdc25C_trash = y(ylength,298);

Cdc25C_unfold = y(ylength,299);

Cdc25C_cytosol_inactive = y(ylength,300);

Cdc25C_cytosol_phospho_active = y(ylength,301);

Cdc25C_nuc_phospho_active = y(ylength,302);

Cdc25C_trash = y(ylength,303);

mRNA_Plk1_unspl = y(ylength,304);

mRNA_Plk1_nuc = y(ylength,305);

mRNA_Plk1_inactive = y(ylength,306);

mRNA_Plk1_cytosol = y(ylength,307);

mRNA_Plk1_in_use = y(ylength,308);

mRNA_Plk1_trash = y(ylength,309);

Plk1_unfold = y(ylength,310);

Plk1_cytosol_inactive = y(ylength,311);

Plk1_cytosol_phospho_active = y(ylength,312);

Plk1_nuc_phospho_active = y(ylength,313);

Plk1_trash = y(ylength,314);

mRNA_Emi1_unspl = y(ylength,315);

mRNA_Emi1_nuc = y(ylength,316);

mRNA_Emi1_inactive = y(ylength,317);

mRNA_Emi1_cytosol = y(ylength,318);

mRNA_Emi1_in_use = y(ylength,319);

mRNA_Emi1_trash = y(ylength,320);

Emi1_unfold = y(ylength,321);

Emi1_in_cell = y(ylength,322);

Emi1_on_Cdh1 = y(ylength,323);

Emi1_on_Cdc20 = y(ylength,324);

Emi1_phospho_inactive = y(ylength,325);

Emi1_trash = y(ylength,326);

mRNA_APC_unspl = y(ylength,327);

mRNA_APC_nuc = y(ylength,328);

mRNA_APC_inactive = y(ylength,329);

mRNA_APC_cytosol = y(ylength,330);

mRNA_APC_in_use = y(ylength,331);

mRNA_APC_trash = y(ylength,332);

APC_unfold = y(ylength,333);

APC_in_cell = y(ylength,334);

APC_on_Cdh1 = y(ylength,335);

APC_on_Cdc20 = y(ylength,336);

APC_trash = y(ylength,337);

mRNA_Cdh1_unspl = y(ylength,338);

mRNA_Cdh1_nuc = y(ylength,339);

mRNA_Cdh1_inactive = y(ylength,340);

mRNA_Cdh1_cytosol = y(ylength,341);

mRNA_Cdh1_in_use = y(ylength,342);

mRNA_Cdh1_trash = y(ylength,343);

Cdh1_unfold = y(ylength,344);

Cdh1_in_cell = y(ylength,345);

Cdh1_Emi1_inactive = y(ylength,346);

Cdh1_phospho_inactive = y(ylength,347);

Cdh1_APC_active = y(ylength,348);

Cdh1_trash = y(ylength,349);

mRNA_Cdc20_unspl = y(ylength,350);

mRNA_Cdc20_nuc = y(ylength,351);

mRNA_Cdc20_inactive = y(ylength,352);

mRNA_Cdc20_cytosol = y(ylength,353);

mRNA_Cdc20_in_use = y(ylength,354);

mRNA_Cdc20_trash = y(ylength,355);

Cdc20_unfold = y(ylength,356);

Cdc20_cytosol = y(ylength,357);

Cdc20_nuc = y(ylength,358);

Cdc20_Emi1_inactive = y(ylength,359);

Cdc20_APC_inactive = y(ylength,360);

Cdc20_APC_active = y(ylength,361);

Cdc20_trash = y(ylength,362);

mRNA_Cdc14_unspl = y(ylength,363);

mRNA_Cdc14_nuc = y(ylength,364);

mRNA_Cdc14_inactive = y(ylength,365);

mRNA_Cdc14_cytosol = y(ylength,366);

mRNA_Cdc14_in_use = y(ylength,367);

mRNA_Cdc14_trash = y(ylength,368);

Cdc14_unfold = y(ylength,369);

Cdc14_cytosol = y(ylength,370);

Cdc14_nuc_inactive = y(ylength,371);

Cdc14_inhibited = y(ylength,372);

Cdc14_phospho_active = y(ylength,373);

Cdc14_trash = y(ylength,374);

mRNA_Cdc25A_unspl = y(ylength,375);

mRNA_Cdc25A_nuc = y(ylength,376);

mRNA_Cdc25A_inactive = y(ylength,377);

mRNA_Cdc25A_cytosol = y(ylength,378);

mRNA_Cdc25A_in_use = y(ylength,379);

mRNA_Cdc25A_trash = y(ylength,380);

Cdc25A_unfold = y(ylength,381);

Cdc25A_cytosol = y(ylength,382);

Cdc25A_nuc_inactive = y(ylength,383);

Cdc25A_phospho_active = y(ylength,384);

Cdc25A_trash = y(ylength,385);

mRNA_Cdc25B_unspl = y(ylength,386);

mRNA_Cdc25B_nuc = y(ylength,387);

mRNA_Cdc25B_inactive = y(ylength,388);

mRNA_Cdc25B_cytosol = y(ylength,389);

mRNA_Cdc25B_in_use = y(ylength,390);

mRNA_Cdc25B_trash = y(ylength,391);

Cdc25B_unfold = y(ylength,392);

Cdc25B_cytosol_inactive = y(ylength,393);

Cdc25B_cytosol_phospho_active = y(ylength,394);

Cdc25B_trash = y(ylength,395);

mRNA_Securin_unspl = y(ylength,396);

mRNA_Securin_nuc = y(ylength,397);

mRNA_Securin_inactive = y(ylength,398);

mRNA_Securin_cytosol = y(ylength,399);

mRNA_Securin_in_use = y(ylength,400);

mRNA_Securin_trash = y(ylength,401);

Securin_unfold = y(ylength,402);

Securin_cytosol = y(ylength,403);

Securin_nuc = y(ylength,404);

Securin_on_chromo = y(ylength,405);

Securin_trash = y(ylength,406);

mRNA_cycC_unspl = y(ylength,407);

mRNA_cycC_nuc = y(ylength,408);

mRNA_cycC_inactive = y(ylength,409);

mRNA_cycC_cytosol = y(ylength,410);

mRNA_cycC_in_use = y(ylength,411);

mRNA_cycC_trash = y(ylength,412);

cycC_unfold = y(ylength,413);

cycC_cytosol = y(ylength,414);

cycC_nuc = y(ylength,415);

cycC_Cdk8_active = y(ylength,416);

cycC_Cdk8_inactive = y(ylength,417);

cycC_trash = y(ylength,418);

mRNA_KPC_unspl = y(ylength,419);

mRNA_KPC_nuc = y(ylength,420);

mRNA_KPC_inactive = y(ylength,421);

mRNA_KPC_cytosol = y(ylength,422);

mRNA_KPC_in_use = y(ylength,423);

mRNA_KPC_trash = y(ylength,424);

KPC_unfold = y(ylength,425);

KPC_in_cell = y(ylength,426);

KPC_active = y(ylength,427);

KPC_trash = y(ylength,428);

% !!!!!!!!!!!!!!!!!!!!!!!!!!!!!!!!! STEADY STATE VS DYNAMIC

% steady state

size_ratio = 1;

dividing = 0;

k_div = 0;

% dynamics

% size_ratio = p_in_use/i_p_in_use;

% [tprev,dividing,k_div] = getdividing...

% (t,bind_Cdh1_to_APC,unbind_Cdh1_APC,...

% u_Cdh1_APC_active,i_NT_in_DNA,NT_in_DNA);

% if(dividing == 1)

% pm_in_NT = 0;

% end

% if(t>2.0E4)

% mitogen_stimulation = 1;

% adhesion_factor = 1;

% end

% !!!!!!!!!!!!!!!!!!!!!!!!!!!!!!!!!!!!! CONCENTRATION FUDGE

AA_in_cytosol_conc = AA_in_cytosol/size_ratio;

AA_in_p_conc = AA_in_p/size_ratio;

AA_on_tRNA_conc = AA_on_tRNA/size_ratio;

ADP_in_cell_conc = ADP_in_cell/size_ratio;

ADP_trash_conc = ADP_trash/size_ratio;

ATP_in_cell_conc = ATP_in_cell/size_ratio;

fats_cytosol_conc = fats_cytosol/size_ratio;

fats_in_mem_conc = fats_in_mem/size_ratio;

fats_in_vesicles_conc = fats_in_vesicles/size_ratio;

H_between_mito_mems_conc = H_between_mito_mems/size_ratio;

H_in_inner_mito_mems_conc = H_in_inner_mito_mems/size_ratio;

junk_spl_conc = junk_spl/size_ratio;

junk_spl_mRNA_conc = junk_spl_mRNA/size_ratio;

junk_spl_rRNA_conc = junk_spl_rRNA/size_ratio;

junk_spl_snRNA_conc = junk_spl_snRNA/size_ratio;

mRNA_cytosol_conc = mRNA_cytosol/size_ratio;

mRNA_in_use_conc = mRNA_in_use/size_ratio;

mRNA_nuc_conc = mRNA_nuc/size_ratio;

mRNA_inactive_conc = mRNA_inactive/size_ratio;

mRNA_trash_conc = mRNA_trash/size_ratio;

mRNA_unspl_conc = mRNA_unspl/size_ratio;

Na_in_cell_conc = Na_in_cell/size_ratio;

Na_out_cell_conc = Na_out_cell/size_ratio;

NT_in_cell_conc = NT_in_cell/size_ratio;

NT_in_RNA_conc = NT_in_RNA/size_ratio;

p_in_cell_conc = p_in_cell/size_ratio;

p_in_use_conc = p_in_use/size_ratio;

p_trash_conc = p_trash/size_ratio;

p_unfold_conc = p_unfold/size_ratio;

rRNA_cytosol_conc = rRNA_cytosol/size_ratio;

rRNA_in_use_conc = rRNA_in_use/size_ratio;

rRNA_nuc_conc = rRNA_nuc/size_ratio;

rRNA_trash_conc = rRNA_trash/size_ratio;

rRNA_unspl_conc = rRNA_unspl/size_ratio;

snRNA_in_use_conc = snRNA_in_use/size_ratio;

snRNA_nuc_conc = snRNA_nuc/size_ratio;

snRNA_trash_conc = snRNA_trash/size_ratio;

snRNA_unspl_conc = snRNA_unspl/size_ratio;

sugar_in_cell_conc = sugar_in_cell/size_ratio;

tRNA_cytosol_conc = tRNA_cytosol/size_ratio;

tRNA_in_use_conc = tRNA_in_use/size_ratio;

tRNA_nuc_conc = tRNA_nuc/size_ratio;

tRNA_trash_conc = tRNA_trash/size_ratio;

mRNA_poly_unspl_conc = mRNA_poly_unspl/size_ratio;

mRNA_poly_nuc_conc = mRNA_poly_nuc/size_ratio;

mRNA_poly_inactive_conc = mRNA_poly_inactive/size_ratio;

mRNA_poly_cytosol_conc = mRNA_poly_cytosol/size_ratio;

mRNA_poly_in_use_conc = mRNA_poly_in_use/size_ratio;

mRNA_poly_trash_conc = mRNA_poly_trash/size_ratio;

p_poly_unfold_conc = p_poly_unfold/size_ratio;

p_poly_cytosol_conc = p_poly_cytosol/size_ratio;

p_poly_nuc_inactive_conc = p_poly_nuc_inactive/size_ratio;

p_poly_nuc_active_conc = p_poly_nuc_active/size_ratio;

p_poly_in_use_conc = p_poly_in_use/size_ratio;

p_poly_trash_conc = p_poly_trash/size_ratio;

mRNA_eIF_4_unspl_conc = mRNA_eIF_4_unspl/size_ratio;

mRNA_eIF_4_nuc_conc = mRNA_eIF_4_nuc/size_ratio;

mRNA_eIF_4_inactive_conc = mRNA_eIF_4_inactive/size_ratio;

mRNA_eIF_4_cytosol_conc = mRNA_eIF_4_cytosol/size_ratio;

mRNA_eIF_4_in_use_conc = mRNA_eIF_4_in_use/size_ratio;

mRNA_eIF_4_trash_conc = mRNA_eIF_4_trash/size_ratio;

p_eIF_4_unfold_conc = p_eIF_4_unfold/size_ratio;

p_eIF_4_cytosol_conc = p_eIF_4_cytosol/size_ratio;

p_eIF_4_in_use_conc = p_eIF_4_in_use/size_ratio;

p_eIF_4_trash_conc = p_eIF_4_trash/size_ratio;

mRNA_p27_unspl_conc = mRNA_p27_unspl/size_ratio;

mRNA_p27_nuc_conc = mRNA_p27_nuc/size_ratio;

mRNA_p27_inactive_conc = mRNA_p27_inactive/size_ratio;

mRNA_p27_cytosol_conc = mRNA_p27_cytosol/size_ratio;

mRNA_p27_in_use_conc = mRNA_p27_in_use/size_ratio;

mRNA_p27_trash_conc = mRNA_p27_trash/size_ratio;

p27_unfold_conc = p27_unfold/size_ratio;

p27_cytosol_conc = p27_cytosol/size_ratio;

p27_nuc_conc = p27_nuc/size_ratio;

p27_on_cyclins_conc = p27_on_cyclins/size_ratio;

p27_trash_conc = p27_trash/size_ratio;

mRNA_Rb_unspl_conc = mRNA_Rb_unspl/size_ratio;

mRNA_Rb_nuc_conc = mRNA_Rb_nuc/size_ratio;

mRNA_Rb_inactive_conc = mRNA_Rb_inactive/size_ratio;

mRNA_Rb_cytosol_conc = mRNA_Rb_cytosol/size_ratio;

mRNA_Rb_in_use_conc = mRNA_Rb_in_use/size_ratio;

mRNA_Rb_trash_conc = mRNA_Rb_trash/size_ratio;

Rb_unfold_conc = Rb_unfold/size_ratio;

Rb_cytosol_conc = Rb_cytosol/size_ratio;

Rb_nuc_conc = Rb_nuc/size_ratio;

Rb_on_E2F_conc = Rb_on_E2F/size_ratio;

Rb_phospho_inactive_conc = Rb_phospho_inactive/size_ratio;

Rb_trash_conc = Rb_trash/size_ratio;

mRNA_cycD_unspl_conc = mRNA_cycD_unspl/size_ratio;

mRNA_cycD_nuc_conc = mRNA_cycD_nuc/size_ratio;

mRNA_cycD_inactive_conc = mRNA_cycD_inactive/size_ratio;

mRNA_cycD_cytosol_conc = mRNA_cycD_cytosol/size_ratio;

mRNA_cycD_in_use_conc = mRNA_cycD_in_use/size_ratio;

mRNA_cycD_trash_conc = mRNA_cycD_trash/size_ratio;

cycD_unfold_conc = cycD_unfold/size_ratio;

cycD_cytosol_conc = cycD_cytosol/size_ratio;

cycD_nuc_conc = cycD_nuc/size_ratio;

cycD_Cdk4or6_active_conc = cycD_Cdk4or6_active/size_ratio;

cycD_Cdk4or6_on_p27_inactive_conc = cycD_Cdk4or6_on_p27_inactive/size_ratio;

cycD_trash_conc = cycD_trash/size_ratio;

mRNA_Cdk2_unspl_conc = mRNA_Cdk2_unspl/size_ratio;

mRNA_Cdk2_nuc_conc = mRNA_Cdk2_nuc/size_ratio;

mRNA_Cdk2_inactive_conc = mRNA_Cdk2_inactive/size_ratio;

mRNA_Cdk2_cytosol_conc = mRNA_Cdk2_cytosol/size_ratio;

mRNA_Cdk2_in_use_conc = mRNA_Cdk2_in_use/size_ratio;

mRNA_Cdk2_trash_conc = mRNA_Cdk2_trash/size_ratio;

Cdk2_unfold_conc = Cdk2_unfold/size_ratio;

Cdk2_cytosol_conc = Cdk2_cytosol/size_ratio;

Cdk2_nuc_conc = Cdk2_nuc/size_ratio;

Cdk2_on_cycE_conc = Cdk2_on_cycE/size_ratio;

Cdk2_on_cycA_conc = Cdk2_on_cycA/size_ratio;

Cdk2_trash_conc = Cdk2_trash/size_ratio;

mRNA_cycE_unspl_conc = mRNA_cycE_unspl/size_ratio;

mRNA_cycE_nuc_conc = mRNA_cycE_nuc/size_ratio;

mRNA_cycE_inactive_conc = mRNA_cycE_inactive/size_ratio;

mRNA_cycE_cytosol_conc = mRNA_cycE_cytosol/size_ratio;

mRNA_cycE_in_use_conc = mRNA_cycE_in_use/size_ratio;

mRNA_cycE_trash_conc = mRNA_cycE_trash/size_ratio;

cycE_unfold_conc = cycE_unfold/size_ratio;

cycE_cytosol_conc = cycE_cytosol/size_ratio;

cycE_nuc_conc = cycE_nuc/size_ratio;

cycE_Cdk2_inactive_conc = cycE_Cdk2_inactive/size_ratio;

cycE_Cdk2_active_conc = cycE_Cdk2_active/size_ratio;

cycE_Cdk2_on_p27_inactive_conc = cycE_Cdk2_on_p27_inactive/size_ratio;

cycE_trash_conc = cycE_trash/size_ratio;

mRNA_B_Myb_unspl_conc = mRNA_B_Myb_unspl/size_ratio;

mRNA_B_Myb_nuc_conc = mRNA_B_Myb_nuc/size_ratio;

mRNA_B_Myb_inactive_conc = mRNA_B_Myb_inactive/size_ratio;

mRNA_B_Myb_cytosol_conc = mRNA_B_Myb_cytosol/size_ratio;

mRNA_B_Myb_in_use_conc = mRNA_B_Myb_in_use/size_ratio;

mRNA_B_Myb_trash_conc = mRNA_B_Myb_trash/size_ratio;

B_Myb_unfold_conc = B_Myb_unfold/size_ratio;

B_Myb_cytosol_conc = B_Myb_cytosol/size_ratio;

B_Myb_nuc_conc = B_Myb_nuc/size_ratio;

B_Myb_phospho_active_conc = B_Myb_phospho_active/size_ratio;

B_Myb_on_DNA_conc = B_Myb_on_DNA/size_ratio;

B_Myb_trash_conc = B_Myb_trash/size_ratio;

mRNA_NF_Y_unspl_conc = mRNA_NF_Y_unspl/size_ratio;

mRNA_NF_Y_nuc_conc = mRNA_NF_Y_nuc/size_ratio;

mRNA_NF_Y_inactive_conc = mRNA_NF_Y_inactive/size_ratio;

mRNA_NF_Y_cytosol_conc = mRNA_NF_Y_cytosol/size_ratio;

mRNA_NF_Y_in_use_conc = mRNA_NF_Y_in_use/size_ratio;

mRNA_NF_Y_trash_conc = mRNA_NF_Y_trash/size_ratio;

NF_Y_unfold_conc = NF_Y_unfold/size_ratio;

NF_Y_cytosol_conc = NF_Y_cytosol/size_ratio;

NF_Y_nuc_conc = NF_Y_nuc/size_ratio;

NF_Y_phospho_active_conc = NF_Y_phospho_active/size_ratio;

NF_Y_on_DNA_conc = NF_Y_on_DNA/size_ratio;

NF_Y_trash_conc = NF_Y_trash/size_ratio;

mRNA_E2F_unspl_conc = mRNA_E2F_unspl/size_ratio;

mRNA_E2F_nuc_conc = mRNA_E2F_nuc/size_ratio;

mRNA_E2F_inactive_conc = mRNA_E2F_inactive/size_ratio;

mRNA_E2F_cytosol_conc = mRNA_E2F_cytosol/size_ratio;

mRNA_E2F_in_use_conc = mRNA_E2F_in_use/size_ratio;

mRNA_E2F_trash_conc = mRNA_E2F_trash/size_ratio;

E2F_unfold_conc = E2F_unfold/size_ratio;

E2F_cytosol_conc = E2F_cytosol/size_ratio;

E2F_nuc_conc = E2F_nuc/size_ratio;

E2F_Rb_inactive_conc = E2F_Rb_inactive/size_ratio;

E2F_on_DNA_conc = E2F_on_DNA/size_ratio;

E2F_trash_conc = E2F_trash/size_ratio;

mRNA_cycA_unspl_conc = mRNA_cycA_unspl/size_ratio;

mRNA_cycA_nuc_conc = mRNA_cycA_nuc/size_ratio;

mRNA_cycA_inactive_conc = mRNA_cycA_inactive/size_ratio;

mRNA_cycA_cytosol_conc = mRNA_cycA_cytosol/size_ratio;

mRNA_cycA_in_use_conc = mRNA_cycA_in_use/size_ratio;

mRNA_cycA_trash_conc = mRNA_cycA_trash/size_ratio;

cycA_unfold_conc = cycA_unfold/size_ratio;

cycA_cytosol_conc = cycA_cytosol/size_ratio;

cycA_nuc_conc = cycA_nuc/size_ratio;

cycA_Cdk2_inactive_conc = cycA_Cdk2_inactive/size_ratio;

cycA_Cdk2_on_p27_inactive_conc = cycA_Cdk2_on_p27_inactive/size_ratio;

cycA_Cdk2_active_conc = cycA_Cdk2_active/size_ratio;

cycA_Cdk1_cytosol_active_conc = cycA_Cdk1_cytosol_active/size_ratio;

cycA_Cdk1_phospho_inactive_conc = cycA_Cdk1_phospho_inactive/size_ratio;

cycA_Cdk1_nuc_active_conc = cycA_Cdk1_nuc_active/size_ratio;

cycA_trash_conc = cycA_trash/size_ratio;

mRNA_SCF_unspl_conc = mRNA_SCF_unspl/size_ratio;

mRNA_SCF_nuc_conc = mRNA_SCF_nuc/size_ratio;

mRNA_SCF_inactive_conc = mRNA_SCF_inactive/size_ratio;

mRNA_SCF_cytosol_conc = mRNA_SCF_cytosol/size_ratio;

mRNA_SCF_in_use_conc = mRNA_SCF_in_use/size_ratio;

mRNA_SCF_trash_conc = mRNA_SCF_trash/size_ratio;

SCF_unfold_conc = SCF_unfold/size_ratio;

SCF_in_cell_conc = SCF_in_cell/size_ratio;

SCF_on_Btrc_conc = SCF_on_Btrc/size_ratio;

SCF_on_Fbw7_conc = SCF_on_Fbw7/size_ratio;

SCF_on_Skp2_conc = SCF_on_Skp2/size_ratio;

SCF_trash_conc = SCF_trash/size_ratio;

mRNA_Skp2_unspl_conc = mRNA_Skp2_unspl/size_ratio;

mRNA_Skp2_nuc_conc = mRNA_Skp2_nuc/size_ratio;

mRNA_Skp2_inactive_conc = mRNA_Skp2_inactive/size_ratio;

mRNA_Skp2_cytosol_conc = mRNA_Skp2_cytosol/size_ratio;

mRNA_Skp2_in_use_conc = mRNA_Skp2_in_use/size_ratio;

mRNA_Skp2_trash_conc = mRNA_Skp2_trash/size_ratio;

Skp2_unfold_conc = Skp2_unfold/size_ratio;

Skp2_cytosol_conc = Skp2_cytosol/size_ratio;

Skp2_nuc_conc = Skp2_nuc/size_ratio;

Skp2_SCF_active_conc = Skp2_SCF_active/size_ratio;

Skp2_trash_conc = Skp2_trash/size_ratio;

mRNA_Btrc_unspl_conc = mRNA_Btrc_unspl/size_ratio;

mRNA_Btrc_nuc_conc = mRNA_Btrc_nuc/size_ratio;

mRNA_Btrc_inactive_conc = mRNA_Btrc_inactive/size_ratio;

mRNA_Btrc_cytosol_conc = mRNA_Btrc_cytosol/size_ratio;

mRNA_Btrc_in_use_conc = mRNA_Btrc_in_use/size_ratio;

mRNA_Btrc_trash_conc = mRNA_Btrc_trash/size_ratio;

Btrc_unfold_conc = Btrc_unfold/size_ratio;

Btrc_cytosol_conc = Btrc_cytosol/size_ratio;

Btrc_SCF_active_conc = Btrc_SCF_active/size_ratio;

Btrc_trash_conc = Btrc_trash/size_ratio;

mRNA_Fbw7_unspl_conc = mRNA_Fbw7_unspl/size_ratio;

mRNA_Fbw7_nuc_conc = mRNA_Fbw7_nuc/size_ratio;

mRNA_Fbw7_inactive_conc = mRNA_Fbw7_inactive/size_ratio;

mRNA_Fbw7_cytosol_conc = mRNA_Fbw7_cytosol/size_ratio;

mRNA_Fbw7_in_use_conc = mRNA_Fbw7_in_use/size_ratio;

mRNA_Fbw7_trash_conc = mRNA_Fbw7_trash/size_ratio;

Fbw7_unfold_conc = Fbw7_unfold/size_ratio;

Fbw7_cytosol_conc = Fbw7_cytosol/size_ratio;

Fbw7_nuc_conc = Fbw7_nuc/size_ratio;

Fbw7_SCF_active_conc = Fbw7_SCF_active/size_ratio;

Fbw7_trash_conc = Fbw7_trash/size_ratio;

mRNA_TF_grow_unspl_conc = mRNA_TF_grow_unspl/size_ratio;

mRNA_TF_grow_inactive_conc = mRNA_TF_grow_inactive/size_ratio;

mRNA_TF_grow_cytosol_conc = mRNA_TF_grow_cytosol/size_ratio;

mRNA_TF_grow_nuc_conc = mRNA_TF_grow_nuc/size_ratio;

mRNA_TF_grow_in_use_conc = mRNA_TF_grow_in_use/size_ratio;

mRNA_TF_grow_trash_conc = mRNA_TF_grow_trash/size_ratio;

TF_grow_unfold_conc = TF_grow_unfold/size_ratio;

TF_grow_cytosol_conc = TF_grow_cytosol/size_ratio;

TF_grow_nuc_conc = TF_grow_nuc/size_ratio;

TF_grow_on_DNA_conc = TF_grow_on_DNA/size_ratio;

TF_grow_trash_conc = TF_grow_trash/size_ratio;

NT_in_DNA_conc = NT_in_DNA/size_ratio;

mRNA_RC_unspl_conc = mRNA_RC_unspl/size_ratio;

mRNA_RC_nuc_conc = mRNA_RC_nuc/size_ratio;

mRNA_RC_inactive_conc = mRNA_RC_inactive/size_ratio;

mRNA_RC_cytosol_conc = mRNA_RC_cytosol/size_ratio;

mRNA_RC_in_use_conc = mRNA_RC_in_use/size_ratio;

mRNA_RC_trash_conc = mRNA_RC_trash/size_ratio;

RC_unfold_conc = RC_unfold/size_ratio;

RC_cytosol_conc = RC_cytosol/size_ratio;

RC_nuc_conc = RC_nuc/size_ratio;

RC_on_DNA_conc = RC_on_DNA/size_ratio;

RC_licensed_conc = RC_licensed/size_ratio;

RC_traversed_by_DNA_poly_conc = RC_traversed_by_DNA_poly/size_ratio;

RC_trash_conc = RC_trash/size_ratio;

RC_count_conc = RC_count/size_ratio;

mRNA_DNA_poly_unspl_conc = mRNA_DNA_poly_unspl/size_ratio;

mRNA_DNA_poly_nuc_conc = mRNA_DNA_poly_nuc/size_ratio;

mRNA_DNA_poly_inactive_conc = mRNA_DNA_poly_inactive/size_ratio;

mRNA_DNA_poly_cytosol_conc = mRNA_DNA_poly_cytosol/size_ratio;

mRNA_DNA_poly_in_use_conc = mRNA_DNA_poly_in_use/size_ratio;

mRNA_DNA_poly_trash_conc = mRNA_DNA_poly_trash/size_ratio;

DNA_poly_unfold_conc = DNA_poly_unfold/size_ratio;

DNA_poly_cytosol_conc = DNA_poly_cytosol/size_ratio;

DNA_poly_nuc_conc = DNA_poly_nuc/size_ratio;

DNA_poly_on_DNA_conc = DNA_poly_on_DNA/size_ratio;

DNA_poly_trash_conc = DNA_poly_trash/size_ratio;

mRNA_Wee1_unspl_conc = mRNA_Wee1_unspl/size_ratio;

mRNA_Wee1_nuc_conc = mRNA_Wee1_nuc/size_ratio;

mRNA_Wee1_inactive_conc = mRNA_Wee1_inactive/size_ratio;

mRNA_Wee1_cytosol_conc = mRNA_Wee1_cytosol/size_ratio;

mRNA_Wee1_in_use_conc = mRNA_Wee1_in_use/size_ratio;

mRNA_Wee1_trash_conc = mRNA_Wee1_trash/size_ratio;

Wee1_unfold_conc = Wee1_unfold/size_ratio;

Wee1_cytosol_active_conc = Wee1_cytosol_active/size_ratio;

Wee1_phospho_inactive_conc = Wee1_phospho_inactive/size_ratio;

Wee1_trash_conc = Wee1_trash/size_ratio;

mRNA_cycB_unspl_conc = mRNA_cycB_unspl/size_ratio;

mRNA_cycB_nuc_conc = mRNA_cycB_nuc/size_ratio;

mRNA_cycB_inactive_conc = mRNA_cycB_inactive/size_ratio;

mRNA_cycB_cytosol_conc = mRNA_cycB_cytosol/size_ratio;

mRNA_cycB_in_use_conc = mRNA_cycB_in_use/size_ratio;

mRNA_cycB_trash_conc = mRNA_cycB_trash/size_ratio;

cycB_unfold_conc = cycB_unfold/size_ratio;

cycB_cytosol_conc = cycB_cytosol/size_ratio;

cycB_Cdk1_cytosol_active_conc = cycB_Cdk1_cytosol_active/size_ratio;

cycB_Cdk1_phospho_inactive_conc = cycB_Cdk1_phospho_inactive/size_ratio;

cycB_Cdk1_nuc_active_conc = cycB_Cdk1_nuc_active/size_ratio;

cycB_trash_conc = cycB_trash/size_ratio;

mRNA_Cdk1_unspl_conc = mRNA_Cdk1_unspl/size_ratio;

mRNA_Cdk1_nuc_conc = mRNA_Cdk1_nuc/size_ratio;

mRNA_Cdk1_inactive_conc = mRNA_Cdk1_inactive/size_ratio;

mRNA_Cdk1_cytosol_conc = mRNA_Cdk1_cytosol/size_ratio;

mRNA_Cdk1_in_use_conc = mRNA_Cdk1_in_use/size_ratio;

mRNA_Cdk1_trash_conc = mRNA_Cdk1_trash/size_ratio;

Cdk1_unfold_conc = Cdk1_unfold/size_ratio;

Cdk1_inactive_conc = Cdk1_inactive/size_ratio;

Cdk1_in_cell_conc = Cdk1_in_cell/size_ratio;

Cdk1_on_cycB_conc = Cdk1_on_cycB/size_ratio;

Cdk1_on_cycA_conc = Cdk1_on_cycA/size_ratio;

Cdk1_trash_conc = Cdk1_trash/size_ratio;

mRNA_Cdc25C_unspl_conc = mRNA_Cdc25C_unspl/size_ratio;

mRNA_Cdc25C_nuc_conc = mRNA_Cdc25C_nuc/size_ratio;

mRNA_Cdc25C_inactive_conc = mRNA_Cdc25C_inactive/size_ratio;

mRNA_Cdc25C_cytosol_conc = mRNA_Cdc25C_cytosol/size_ratio;

mRNA_Cdc25C_in_use_conc = mRNA_Cdc25C_in_use/size_ratio;

mRNA_Cdc25C_trash_conc = mRNA_Cdc25C_trash/size_ratio;

Cdc25C_unfold_conc = Cdc25C_unfold/size_ratio;

Cdc25C_cytosol_inactive_conc = Cdc25C_cytosol_inactive/size_ratio;

Cdc25C_cytosol_phospho_active_conc = Cdc25C_cytosol_phospho_active/size_ratio;

Cdc25C_nuc_phospho_active_conc = Cdc25C_nuc_phospho_active/size_ratio;

Cdc25C_trash_conc = Cdc25C_trash/size_ratio;

mRNA_Plk1_unspl_conc = mRNA_Plk1_unspl/size_ratio;

mRNA_Plk1_nuc_conc = mRNA_Plk1_nuc/size_ratio;

mRNA_Plk1_inactive_conc = mRNA_Plk1_inactive/size_ratio;

mRNA_Plk1_cytosol_conc = mRNA_Plk1_cytosol/size_ratio;

mRNA_Plk1_in_use_conc = mRNA_Plk1_in_use/size_ratio;

mRNA_Plk1_trash_conc = mRNA_Plk1_trash/size_ratio;

Plk1_unfold_conc = Plk1_unfold/size_ratio;

Plk1_cytosol_inactive_conc = Plk1_cytosol_inactive/size_ratio;

Plk1_cytosol_phospho_active_conc = Plk1_cytosol_phospho_active/size_ratio;

Plk1_nuc_phospho_active_conc = Plk1_nuc_phospho_active/size_ratio;

Plk1_trash_conc = Plk1_trash/size_ratio;

mRNA_Emi1_unspl_conc = mRNA_Emi1_unspl/size_ratio;

mRNA_Emi1_nuc_conc = mRNA_Emi1_nuc/size_ratio;

mRNA_Emi1_inactive_conc = mRNA_Emi1_inactive/size_ratio;

mRNA_Emi1_cytosol_conc = mRNA_Emi1_cytosol/size_ratio;

mRNA_Emi1_in_use_conc = mRNA_Emi1_in_use/size_ratio;

mRNA_Emi1_trash_conc = mRNA_Emi1_trash/size_ratio;

Emi1_unfold_conc = Emi1_unfold/size_ratio;

Emi1_in_cell_conc = Emi1_in_cell/size_ratio;

Emi1_on_Cdh1_conc = Emi1_on_Cdh1/size_ratio;

Emi1_on_Cdc20_conc = Emi1_on_Cdc20/size_ratio;

Emi1_phospho_inactive_conc = Emi1_phospho_inactive/size_ratio;

Emi1_trash_conc = Emi1_trash/size_ratio;

mRNA_APC_unspl_conc = mRNA_APC_unspl/size_ratio;

mRNA_APC_nuc_conc = mRNA_APC_nuc/size_ratio;

mRNA_APC_inactive_conc = mRNA_APC_inactive/size_ratio;

mRNA_APC_cytosol_conc = mRNA_APC_cytosol/size_ratio;

mRNA_APC_in_use_conc = mRNA_APC_in_use/size_ratio;

mRNA_APC_trash_conc = mRNA_APC_trash/size_ratio;

APC_unfold_conc = APC_unfold/size_ratio;

APC_in_cell_conc = APC_in_cell/size_ratio;

APC_on_Cdh1_conc = APC_on_Cdh1/size_ratio;

APC_on_Cdc20_conc = APC_on_Cdc20/size_ratio;

APC_trash_conc = APC_trash/size_ratio;

mRNA_Cdh1_unspl_conc = mRNA_Cdh1_unspl/size_ratio;

mRNA_Cdh1_nuc_conc = mRNA_Cdh1_nuc/size_ratio;

mRNA_Cdh1_inactive_conc = mRNA_Cdh1_inactive/size_ratio;

mRNA_Cdh1_cytosol_conc = mRNA_Cdh1_cytosol/size_ratio;

mRNA_Cdh1_in_use_conc = mRNA_Cdh1_in_use/size_ratio;

mRNA_Cdh1_trash_conc = mRNA_Cdh1_trash/size_ratio;

Cdh1_unfold_conc = Cdh1_unfold/size_ratio;

Cdh1_in_cell_conc = Cdh1_in_cell/size_ratio;

Cdh1_Emi1_inactive_conc = Cdh1_Emi1_inactive/size_ratio;

Cdh1_phospho_inactive_conc = Cdh1_phospho_inactive/size_ratio;

Cdh1_APC_active_conc = Cdh1_APC_active/size_ratio;

Cdh1_trash_conc = Cdh1_trash/size_ratio;

mRNA_Cdc20_unspl_conc = mRNA_Cdc20_unspl/size_ratio;

mRNA_Cdc20_nuc_conc = mRNA_Cdc20_nuc/size_ratio;

mRNA_Cdc20_inactive_conc = mRNA_Cdc20_inactive/size_ratio;

mRNA_Cdc20_cytosol_conc = mRNA_Cdc20_cytosol/size_ratio;

mRNA_Cdc20_in_use_conc = mRNA_Cdc20_in_use/size_ratio;

mRNA_Cdc20_trash_conc = mRNA_Cdc20_trash/size_ratio;

Cdc20_unfold_conc = Cdc20_unfold/size_ratio;

Cdc20_cytosol_conc = Cdc20_cytosol/size_ratio;

Cdc20_nuc_conc = Cdc20_nuc/size_ratio;

Cdc20_Emi1_inactive_conc = Cdc20_Emi1_inactive/size_ratio;

Cdc20_APC_inactive_conc = Cdc20_APC_inactive/size_ratio;

Cdc20_APC_active_conc = Cdc20_APC_active/size_ratio;

Cdc20_trash_conc = Cdc20_trash/size_ratio;

mRNA_Cdc14_unspl_conc = mRNA_Cdc14_unspl/size_ratio;

mRNA_Cdc14_nuc_conc = mRNA_Cdc14_nuc/size_ratio;

mRNA_Cdc14_inactive_conc = mRNA_Cdc14_inactive/size_ratio;

mRNA_Cdc14_cytosol_conc = mRNA_Cdc14_cytosol/size_ratio;

mRNA_Cdc14_in_use_conc = mRNA_Cdc14_in_use/size_ratio;

mRNA_Cdc14_trash_conc = mRNA_Cdc14_trash/size_ratio;

Cdc14_unfold_conc = Cdc14_unfold/size_ratio;

Cdc14_cytosol_conc = Cdc14_cytosol/size_ratio;

Cdc14_nuc_inactive_conc = Cdc14_nuc_inactive/size_ratio;

Cdc14_inhibited_conc = Cdc14_inhibited/size_ratio;

Cdc14_phospho_active_conc = Cdc14_phospho_active/size_ratio;

Cdc14_trash_conc = Cdc14_trash/size_ratio;

mRNA_Cdc25A_unspl_conc = mRNA_Cdc25A_unspl/size_ratio;

mRNA_Cdc25A_nuc_conc = mRNA_Cdc25A_nuc/size_ratio;

mRNA_Cdc25A_inactive_conc = mRNA_Cdc25A_inactive/size_ratio;

mRNA_Cdc25A_cytosol_conc = mRNA_Cdc25A_cytosol/size_ratio;

mRNA_Cdc25A_in_use_conc = mRNA_Cdc25A_in_use/size_ratio;

mRNA_Cdc25A_trash_conc = mRNA_Cdc25A_trash/size_ratio;

Cdc25A_unfold_conc = Cdc25A_unfold/size_ratio;

Cdc25A_cytosol_conc = Cdc25A_cytosol/size_ratio;

Cdc25A_nuc_inactive_conc = Cdc25A_nuc_inactive/size_ratio;

Cdc25A_phospho_active_conc = Cdc25A_phospho_active/size_ratio;

Cdc25A_trash_conc = Cdc25A_trash/size_ratio;

mRNA_Cdc25B_unspl_conc = mRNA_Cdc25B_unspl/size_ratio;

mRNA_Cdc25B_nuc_conc = mRNA_Cdc25B_nuc/size_ratio;

mRNA_Cdc25B_inactive_conc = mRNA_Cdc25B_inactive/size_ratio;

mRNA_Cdc25B_cytosol_conc = mRNA_Cdc25B_cytosol/size_ratio;

mRNA_Cdc25B_in_use_conc = mRNA_Cdc25B_in_use/size_ratio;

mRNA_Cdc25B_trash_conc = mRNA_Cdc25B_trash/size_ratio;

Cdc25B_unfold_conc = Cdc25B_unfold/size_ratio;

Cdc25B_cytosol_inactive_conc = Cdc25B_cytosol_inactive/size_ratio;

Cdc25B_cytosol_phospho_active_conc = Cdc25B_cytosol_phospho_active/size_ratio;

Cdc25B_trash_conc = Cdc25B_trash/size_ratio;

mRNA_Securin_unspl_conc = mRNA_Securin_unspl/size_ratio;

mRNA_Securin_nuc_conc = mRNA_Securin_nuc/size_ratio;

mRNA_Securin_inactive_conc = mRNA_Securin_inactive/size_ratio;

mRNA_Securin_cytosol_conc = mRNA_Securin_cytosol/size_ratio;

mRNA_Securin_in_use_conc = mRNA_Securin_in_use/size_ratio;

mRNA_Securin_trash_conc = mRNA_Securin_trash/size_ratio;

Securin_unfold_conc = Securin_unfold/size_ratio;

Securin_cytosol_conc = Securin_cytosol/size_ratio;

Securin_nuc_conc = Securin_nuc/size_ratio;

Securin_on_chromo_conc = Securin_on_chromo/size_ratio;

Securin_trash_conc = Securin_trash/size_ratio;

mRNA_cycC_unspl_conc = mRNA_cycC_unspl/size_ratio;

mRNA_cycC_nuc_conc = mRNA_cycC_nuc/size_ratio;

mRNA_cycC_inactive_conc = mRNA_cycC_inactive/size_ratio;

mRNA_cycC_cytosol_conc = mRNA_cycC_cytosol/size_ratio;

mRNA_cycC_in_use_conc = mRNA_cycC_in_use/size_ratio;

mRNA_cycC_trash_conc = mRNA_cycC_trash/size_ratio;

cycC_unfold_conc = cycC_unfold/size_ratio;

cycC_cytosol_conc = cycC_cytosol/size_ratio;

cycC_nuc_conc = cycC_nuc/size_ratio;

cycC_Cdk8_active_conc = cycC_Cdk8_active/size_ratio;

cycC_Cdk8_inactive_conc = cycC_Cdk8_inactive/size_ratio;

cycC_trash_conc = cycC_trash/size_ratio;

mRNA_KPC_unspl_conc = mRNA_KPC_unspl/size_ratio;

mRNA_KPC_nuc_conc = mRNA_KPC_nuc/size_ratio;

mRNA_KPC_inactive_conc = mRNA_KPC_inactive/size_ratio;

mRNA_KPC_cytosol_conc = mRNA_KPC_cytosol/size_ratio;

mRNA_KPC_in_use_conc = mRNA_KPC_in_use/size_ratio;

mRNA_KPC_trash_conc = mRNA_KPC_trash/size_ratio;

KPC_unfold_conc = KPC_unfold/size_ratio;

KPC_in_cell_conc = KPC_in_cell/size_ratio;

KPC_active_conc = KPC_active/size_ratio;

KPC_trash_conc = KPC_trash/size_ratio;

% !!!!!!!!!!!!!!!!!!!!!!!!!!!!!!!!!!!!!!!!!!!!!!! RATE EQNS

% equations "ATP_" give actual rate of energy used in a process

% equations "energy_" give rate of energy anticipated in a process

% equations "ER_" give elongation rate

% equations "NT_frac_" give percent of various RNA types

% equations "poly" give number of RNA polymerases

% equations "R_diff_" give normalized ratio (times 10^6)

% of difference of input and output rates

%

% 000000000011111111112222222222333333333344444444445555555555666666666677777777778888888888

%

energy_dispose = ATP_u*k_dk_p...

*(p_in_cell_conc + p_in_use_conc + p_unfold_conc)...

+ ATP_proteasome*k_cut_p*p_trash_conc*x_p;

energy_Na_pump = ATP_Na*k_Na_pump_out*Na_in_cell_conc;

energy_out_nm = ATP_NT_out_nm*(x_mRNA*k_nm_mRNA*mRNA_nuc_conc...

+ x_rRNA*k_nm_rRNA*rRNA_nuc_conc...

+ x_tRNA*k_nm_tRNA*tRNA_nuc_conc);

energy_p_transport = ATP_mem_transfer*k_bind_p*p_in_cell_conc...

*(frac_p_nuc + frac_p_ER + frac_p_mitochondria...

+ frac_p_lysosome);

spl_pool = exon_mRNA*mRNA_unspl + exon_rRNA*rRNA_unspl...

+ exon_snRNA*snRNA_unspl;

spl_pool_conc = spl_pool/size_ratio;

spl_frac = min(1, k_spl*snRNA_in_use_conc/spl_pool_conc);

energy_spl = ATP_spl*(exon_mRNA*min(mRNA_unspl,...

spl_frac*exon_mRNA*mRNA_unspl)...

+ exon_rRNA*min(rRNA_unspl, spl_frac*exon_rRNA*rRNA_unspl)...

+ exon_snRNA*min(snRNA_unspl,...

spl_frac*exon_snRNA*snRNA_unspl));

energy_structure = ATP_polymerization*frac_p_cytosol*k_bind_p*p_in_cell_conc...

+ ATP_glycosolation*frac_p_ER*k_bind_p*p_in_cell_conc...

+ ATP_fat*k_make_vesicle*fats_cytosol_conc...

+ ATP_vesicle/x_vesicle*k_bind_fats*fats_in_vesicles_conc;

ER_p = k_ER_p_constant*i_ER_p*AA_on_tRNA_conc;

k_p_txl = ER_p/x_p;

e_txl_tRNA = ATP_tRNA*min(k_tRNA_AA_binding*tRNA_cytosol_conc...

*AA_in_cytosol_conc, AA_in_cytosol_conc);

e_txl_AA = ATP_AA*x_p*min(k_p_txl*rRNA_in_use_conc, tRNA_in_use/x_p);

e_txl_fold = ATP_fold*k_fold_p*p_unfold;

e_txl_eIF_4 = ATP_eIF_4*k_bind_rRNA_mRNA*mRNA_cytosol_conc*rRNA_cytosol_conc;

energy_txl = e_txl_tRNA + e_txl_AA + e_txl_fold + e_txl_eIF_4;

ER_RNA = k_ER_RNA_constant*i_ER_RNA*NT_in_cell_conc;

ER_DNA = k_ER_DNA_constant*i_ER_DNA*NT_in_cell_conc;

polyI = frac_polyI*p_poly_in_use;

polyII = frac_polyII*p_poly_in_use;

polyIII = frac_polyIII*p_poly_in_use;

NT_request = ER_RNA*(polyI + polyII + frac_polyII_snRNA*polyII + polyIII)...

+ DNA_poly_on_DNA*ER_DNA;

NT_frac_avail = min(1, NT_in_cell/NT_request);

k_mRNA_txs = NT_frac_avail*ER_RNA/x_mRNA_unspl;

k_rRNA_txs = NT_frac_avail*ER_RNA/x_rRNA_unspl;

k_snRNA_txs = NT_frac_avail*ER_RNA/x_snRNA_unspl;

k_tRNA_txs = NT_frac_avail*ER_RNA/x_tRNA;

energy_txs = ATP_NT_txs*NT_frac_avail...

*(x_mRNA_unspl*k_mRNA_txs*polyII...

+ x_rRNA_unspl*k_rRNA_txs*polyI...

+ x_snRNA_unspl*k_snRNA_txs*frac_polyII_snRNA*polyII...

+ x_tRNA*k_tRNA_txs*polyIII);

energy_DNA_repl = ATP_DNA_repl*NT_frac_avail*ER_DNA*DNA_poly_on_DNA;

energy_in_pm = ATP_pm_transfer*(k_pm_in_AA...

*max(0, i_AA_in_cytosol - AA_in_cytosol_conc)...

+ k_pm_in_ADP*max(0, i_ATP_in_cell + i_ADP_in_cell...

- (ATP_in_cell_conc + ADP_in_cell_conc))...

+ k_pm_in_NT*max(0, i_NT_in_cell - NT_in_cell_conc));

energy_request = energy_dispose + energy_out_nm + energy_Na_pump...

+ energy_p_transport + energy_txs + energy_txl...

+ energy_structure + energy_spl + energy_in_pm;

ATP_frac_avail = min(1, ATP_in_cell/energy_request);

txs_mRNA = ATP_frac_avail*k_mRNA_txs*polyII;

txs_rRNA = ATP_frac_avail*k_rRNA_txs*polyI;

txs_snRNA = ATP_frac_avail*k_snRNA_txs*frac_polyII_snRNA*polyII;

txs_tRNA = ATP_frac_avail*k_tRNA_txs*polyIII;

ATP_txs = ATP_NT*(x_mRNA_unspl*txs_mRNA + x_rRNA_unspl*txs_rRNA...

+ x_snRNA_unspl*txs_snRNA + x_tRNA*txs_tRNA);

bind_p = ATP_frac_avail*k_bind_p*p_in_cell_conc;

u1_p = ATP_frac_avail*k_dk_p*p_in_cell_conc;

u2_p = ATP_frac_avail*k_dk_p*p_in_use_conc;

u3_p = ATP_frac_avail*k_dk_p*p_unfold_conc;

cut_p = ATP_frac_avail*k_cut_p*p_trash_conc;

ATP_dispose = ATP_u*(u1_p + u2_p + u3_p) + ATP_proteasome*x_p*cut_p;

Na_pump = k_Na_pump_out*ATP_frac_avail*Na_in_cell_conc;

ATP_Na_pump = ATP_Na*Na_pump;

nm_out_mRNA = ATP_frac_avail*k_nm_mRNA*mRNA_nuc_conc;

nm_out_rRNA = ATP_frac_avail*k_nm_rRNA*rRNA_nuc_conc;

nm_out_tRNA = ATP_frac_avail*k_nm_tRNA*tRNA_nuc_conc;

ATP_out_nm = ATP_NT_out_nm*(x_mRNA*nm_out_mRNA + x_rRNA*nm_out_rRNA...

+ x_tRNA*nm_out_tRNA);

glycosolation = ATP_frac_avail*frac_p_ER*bind_p;

polymerization = ATP_frac_avail*frac_p_cytosol*bind_p;

p_into_ER = frac_p_ER*bind_p;

p_into_lysosomes = frac_p_lysosome*bind_p;

p_into_mitochondria = frac_p_mitochondria*bind_p;

p_into_nm = frac_p_nuc*bind_p;

ATP_p_transport = ATP_mem_transfer*(p_into_nm + p_into_ER...

+ p_into_lysosomes + p_into_mitochondria);

spl_mRNA = ATP_frac_avail*spl_frac*mRNA_unspl;

spl_rRNA = ATP_frac_avail*spl_frac*rRNA_unspl;

spl_snRNA = ATP_frac_avail*spl_frac*snRNA_unspl;

ATP_for_spl = ATP_spl*(spl_mRNA*exon_mRNA + spl_rRNA*exon_rRNA...

+ spl_snRNA*exon_snRNA);

make_vesicle = ATP_frac_avail*k_make_vesicle*fats_cytosol_conc;

bind_fats = ATP_frac_avail*k_bind_fats*fats_in_vesicles_conc;

ATP_structure = ATP_polymerization*polymerization...

+ ATP_glycosolation*glycosolation...

+ ATP_fat*make_vesicle + ATP_vesicle/x_vesicle*bind_fats;

mRNA_in_use_blocked = rRNA_in_use*rRNA_spacing_on_mRNA/x_mRNA;

tot_mRNA_avail = mRNA_cytosol + mRNA_in_use - mRNA_in_use_blocked;

tot_mRNA_avail_conc = tot_mRNA_avail/size_ratio;

bind_rRNA = ATP_frac_avail*k_bind_rRNA_mRNA*tot_mRNA_avail_conc...

*rRNA_cytosol_conc;

bind_mRNA = bind_rRNA*mRNA_cytosol_conc/tot_mRNA_avail_conc;

% bind_rRNA = ATP_frac_avail*k_bind_rRNA_mRNA*mRNA_cytosol_conc...

% *rRNA_cytosol_conc;

% bind_mRNA = bind_rRNA;

bind_snRNA = k_bind_snRNA*snRNA_nuc_conc;

bind_tRNA = ATP_frac_avail*k_tRNA_AA_binding*tRNA_cytosol_conc...

*AA_in_cytosol_conc;

bind_NT_in_RNA = x_mRNA_unspl*txs_mRNA + x_rRNA_unspl*txs_rRNA...

+ x_snRNA_unspl*txs_snRNA + x_tRNA*txs_tRNA;

bind_NT_in_DNA = ATP_frac_avail*NT_frac_avail*ER_DNA*DNA_poly_on_DNA;

unbind_rRNA = ATP_frac_avail*k_p_txl*rRNA_in_use_conc;

txl_p = unbind_rRNA;

ATP_txl = ATP_tRNA*bind_tRNA + ATP_AA*x_p*txl_p + ATP_fold*txl_p...

+ ATP_eIF_4*bind_rRNA;

pm_in_AA = ATP_frac_avail*k_pm_in_AA...

*max(0, i_AA_in_cytosol - AA_in_cytosol_conc);

pm_in_ADP = ATP_frac_avail*k_pm_in_ADP*max(0, i_ATP_in_cell...

+ i_ADP_in_cell - (ATP_in_cell_conc + ADP_in_cell_conc));

% if(dividing == 1)

% pm_in_NT = 0;

% else

pm_in_NT = ATP_frac_avail*k_pm_in_NT*max(0, i_NT_in_cell - NT_in_cell_conc);

% end

ATP_in_pm = ATP_pm_transfer*(pm_in_AA + pm_in_ADP + pm_in_NT);

ATP_demand = ATP_dispose + ATP_out_nm + ATP_p_transport + ATP_txl + ATP_txs...

+ ATP_structure + ATP_for_spl + ATP_Na_pump + ATP_in_pm;

Eq_ATP = ADP_in_cell/ATP_in_cell;

% ATP_synthase = k_ATP_synthase*ADP_in_cell_conc*H_between_mito_mems_conc...

% /H_in_inner_mito_mems_conc;

% ATP_synthase = k_ATP_synthase*ADP_in_cell_conc*(H_between_mito_mems_conc...

% - H_in_inner_mito_mems_conc);

ATP_synthase = k_ATP_synthase*Eq_ATP*H_between_mito_mems_conc...

/H_in_inner_mito_mems_conc;

% ATP_synthase = k_ATP_synthase*Eq_ATP*(H_between_mito_mems_conc...

% - H_in_inner_mito_mems_conc);

bind_AA = bind_tRNA;

bind_ADP = ATP_H*ATP_synthase;

cut_spljunk_mRNA = k_cut_spljunk_mRNA*junk_spl_mRNA;

cut_spljunk_rRNA = k_cut_spljunk_rRNA*junk_spl_rRNA;

cut_spljunk_snRNA = k_cut_spljunk_snRNA*junk_spl_snRNA;

cut_mRNA = k_cut_mRNA*mRNA_trash;

cut_rRNA = k_cut_rRNA*rRNA_trash;

cut_snRNA = k_cut_snRNA*snRNA_trash;

cut_tRNA = k_cut_tRNA*tRNA_trash;

dispose_ADP = ADP_trash;

dk1_ADP = k_dk_ADP*ADP_in_cell;

dk1_mRNA = k_dk_mRNA*mRNA_cytosol;

dk1_rRNA = k_dk_rRNA*rRNA_cytosol;

dk1_snRNA = k_dk_snRNA*snRNA_nuc;

dk1_tRNA = k_dk_tRNA*tRNA_cytosol;

dk2_ADP = k_dk_ADP*ATP_in_cell;

dk2_mRNA = k_dk_mRNA*mRNA_unspl;

dk2_rRNA = k_dk_rRNA*rRNA_unspl;

dk2_snRNA = k_dk_snRNA*snRNA_unspl;

dk2_tRNA = k_dk_tRNA*tRNA_nuc;

dk3_mRNA = k_dk_mRNA*mRNA_nuc;

dk3_rRNA = k_dk_rRNA*rRNA_nuc;

dk3_snRNA = k_dk_snRNA*snRNA_in_use;

dk3_tRNA = k_dk_tRNA*tRNA_in_use;

dk4_mRNA = k_dk_mRNA*mRNA_in_use;

dk4_rRNA = k_dk_rRNA*rRNA_in_use;

dk5_mRNA = k_dk_mRNA*mRNA_inactive;

% dk5_mRNA = 0;

dk_AA = ATP_frac_avail*k_dk_AA*(AA_in_cytosol + AA_on_tRNA + AA_in_p);

dk1_fats = k_dk_fat*fats_cytosol;

dk2_fats = k_dk_fat*fats_in_vesicles;

dk3_fats = k_dk_fat*fats_in_mem;

% NOTE: I am assuming when an NT decays in RNA, it causes the RNA to decay,

% and it is accounted for in RNA decay,

% and I ignore NT decay in DNA

dk_NT = k_dk_NT*NT_in_cell;

fold_p = ATP_frac_avail*k_fold_p*p_unfold;

H_grow = k_grow_H*max(0, i_H_between_mito_mems - H_between_mito_mems_conc);

H_shrink = 0;

% zwork25

% H_pump = min(ATP_respiration/ATP_H*sugar_in_cell,...

% k_H_pump*H_in_inner_mito_mems_conc/H_between_mito_mems_conc...

% /ATP_in_cell_conc);

% H_pump = min(ATP_respiration/ATP_H*sugar_in_cell,...

% k_H_pump*H_in_inner_mito_mems_conc*H_in_inner_mito_mems_conc...

% /H_between_mito_mems_conc/ATP_in_cell_conc);

H_pump = min(ATP_respiration/ATP_H*sugar_in_cell,...

k_H_pump*Eq_ATP*H_in_inner_mito_mems_conc/H_between_mito_mems_conc);

mRNA_synthesis_frac = x_mRNA_unspl*txs_mRNA/bind_NT_in_RNA;

% Na_grow = k_grow_Na*max(0, size_ratio*(i_Na_out_cell - Na_out_cell));

Na_grow = k_grow_Na*max(0, i_Na_out_cell - Na_out_cell_conc);

Na_shrink = 0;

Na_return = max(0, k_Na_return*(Na_out_cell_conc - Na_in_cell_conc));

NT_junk_spl_pool = junk_spl_mRNA + junk_spl_rRNA + junk_spl_snRNA + junk_spl;

NT_mRNA_pool = x_mRNA_unspl*mRNA_unspl...

+ x_mRNA*(mRNA_nuc + mRNA_cytosol + mRNA_in_use);

NT_mRNA_txs = 0.5*polyII*x_mRNA_unspl;

NT_rRNA_pool = x_rRNA_unspl*rRNA_unspl...

+ x_rRNA*(rRNA_nuc + rRNA_cytosol + rRNA_in_use);

NT_rRNA_txs = 0.5*polyI*x_rRNA_unspl;

NT_snRNA_pool = x_snRNA_unspl*snRNA_unspl...

+ x_snRNA*(snRNA_nuc + snRNA_in_use);

NT_snRNA_txs = 0.5*frac_polyII_snRNA*polyII*x_snRNA_unspl;

NT_trash_pool = x_mRNA*mRNA_trash + x_rRNA*rRNA_trash...

+ x_snRNA*snRNA_trash + x_tRNA*tRNA_trash;

NT_tRNA_pool = x_tRNA*(tRNA_nuc + tRNA_cytosol + tRNA_in_use);

NT_tRNA_txs = 0.5*polyIII*x_tRNA;

NT_txs = NT_mRNA_txs + NT_rRNA_txs + NT_snRNA_txs + NT_tRNA_txs;

temp_NT = NT_txs + NT_mRNA_pool + NT_rRNA_pool + NT_snRNA_pool...

+ NT_tRNA_pool + NT_junk_spl_pool + NT_trash_pool;

total_NT = temp_NT + NT_in_cell;

NT_frac_hnRNA = (0.5*polyII*x_mRNA_unspl + x_mRNA_unspl*mRNA_unspl...

+ x_mRNA*mRNA_nuc + (dk2_mRNA + dk3_mRNA)...

/cut_mRNA*mRNA_trash*x_mRNA + junk_spl_mRNA)/temp_NT;

NT_frac_mRNA = x_mRNA*(mRNA_cytosol + mRNA_in_use...

+ (dk1_mRNA + dk4_mRNA + dk5_mRNA)/cut_mRNA*mRNA_trash)/temp_NT;

% NT_frac_mRNA = x_mRNA*(mRNA_cytosol + mRNA_in_use...

% + (dk1_mRNA + dk4_mRNA)/cut_mRNA*mRNA_trash)/temp_NT;

NT_frac_rRNA = x_rRNA*(rRNA_cytosol + rRNA_in_use...

+ (dk1_rRNA + dk4_rRNA)/cut_rRNA*rRNA_trash)/temp_NT;

NT_frac_rRNA_precursors = (0.5*polyI*x_rRNA_unspl + x_rRNA_unspl*rRNA_unspl...

+ x_rRNA*rRNA_nuc + (dk2_rRNA + dk3_rRNA)...

/cut_rRNA*rRNA_trash*x_rRNA...

+ junk_spl_rRNA)/temp_NT;

NT_frac_snRNA = (0.5*frac_polyII_snRNA*polyII*x_snRNA...

+ x_snRNA_unspl*snRNA_unspl + x_snRNA*(snRNA_nuc...

+ snRNA_trash + snRNA_in_use) + junk_spl_snRNA)/temp_NT;

NT_frac_tRNA = (0.5*polyIII*x_tRNA + x_tRNA*(tRNA_nuc...

+ tRNA_cytosol + tRNA_trash + tRNA_in_use))/temp_NT;

NT_frac_RNA_nuc = NT_frac_rRNA_precursors + NT_frac_hnRNA + NT_frac_snRNA...

+ (x_tRNA*tRNA_nuc + 0.5*polyIII*x_tRNA)/temp_NT;

pm_in_fats = k_pm_in_fats*max(0, i_fats_cytosol - fats_cytosol_conc);

pm_out_sugar = H_pump*ATP_H/(ATP_respiration + ATP_glycolysis);

pm_in_sugar = k_pm_in_sugar*max(0, i_sugar_in_cell - sugar_in_cell_conc);

R_diff_mRNA_cyto = 1E6*(nm_out_mRNA...

- (dk1_mRNA + dk4_mRNA + dk5_mRNA))/nm_out_mRNA;

R_diff_mRNA_dk = 1E6*(txs_mRNA...

- (dk1_mRNA + dk2_mRNA + dk3_mRNA + dk4_mRNA...

+ dk5_mRNA))/txs_mRNA;

% R_diff_mRNA_cyto = 1E6*(nm_out_mRNA - (dk1_mRNA + dk4_mRNA))/nm_out_mRNA;

% R_diff_mRNA_dk = 1E6*(txs_mRNA...

% - (dk1_mRNA + dk2_mRNA + dk3_mRNA + dk4_mRNA))/txs_mRNA;

R_diff_mRNA_nm = 1E6*(spl_mRNA - (nm_out_mRNA + dk3_mRNA))/spl_mRNA;

R_diff_mRNA_spl = 1E6*(txs_mRNA - (spl_mRNA + dk2_mRNA))/txs_mRNA;

R_diff_p_dk = 1E6*(txl_p - (u1_p + u2_p + u3_p))/txl_p;

R_diff_p_fold = 1E6*(fold_p - (u1_p + u2_p))/fold_p;

R_diff_rRNA_cyto = 1E6*(nm_out_rRNA - (dk1_rRNA + dk4_rRNA))/nm_out_rRNA;

R_diff_rRNA_dk = 1E6*(txs_rRNA...

- (dk1_rRNA + dk2_rRNA + dk3_rRNA + dk4_rRNA))/txs_rRNA;

R_diff_rRNA_nm = 1E6*(spl_rRNA - (nm_out_rRNA + dk3_rRNA))/spl_rRNA;

R_diff_rRNA_spl = 1E6*(txs_rRNA - (spl_rRNA + dk2_rRNA))/txs_rRNA;

R_diff_snRNA_dk = 1E6*(txs_snRNA...

- (dk1_snRNA + dk2_snRNA + dk3_snRNA))/txs_snRNA;

R_diff_snRNA_spl = 1E6*(txs_snRNA - (spl_snRNA + dk2_snRNA))/txs_snRNA;

R_diff_tRNA_cyto = 1E6*(nm_out_tRNA - (dk1_tRNA + dk3_tRNA))/nm_out_tRNA;

R_diff_tRNA_dk = 1E6*(txs_tRNA - (dk1_tRNA + dk2_tRNA + dk3_tRNA))/txs_tRNA;

R_diff_tRNA_nm = 1E6*(txs_tRNA - (dk1_tRNA + dk2_tRNA + dk3_tRNA))/txs_tRNA;

rRNA_synthesis_frac = x_rRNA_unspl*txs_rRNA/bind_NT_in_RNA;

snRNA_synthesis_frac = x_snRNA_unspl*txs_snRNA/bind_NT_in_RNA;

spljunk = junk_spl;

spljunk_mRNA = (x_mRNA_unspl - x_mRNA)*(spl_mRNA + dk2_mRNA);

spljunk_rRNA = (x_rRNA_unspl - x_rRNA)*(spl_rRNA + dk2_rRNA);

spljunk_snRNA = (x_snRNA_unspl - x_snRNA)*(spl_snRNA + dk2_snRNA);

tRNA_synthesis_frac = x_tRNA*txs_tRNA/bind_NT_in_RNA;

unbind_AA = x_p*cut_p;

unbind_tRNA = x_p*txl_p;

unbind_AA_txl = unbind_tRNA;

unbind_AA_from_dk_tRNA = dk3_tRNA;

unbind_ADP = ATP_demand;

unbind_fats = ATP_frac_avail*k_unbind_fats*fats_in_mem_conc;

unbind_NT_RNA = x_mRNA*cut_mRNA + x_rRNA*cut_rRNA + x_snRNA*cut_snRNA...

+ x_tRNA*cut_tRNA + spljunk_mRNA + spljunk_rRNA...

+ spljunk_snRNA;

unbind_NT_DNA = 0;

% unbind_mRNA = unbind_rRNA*mRNA_in_use/(rRNA_in_use - mRNA_new_in_use);

% unbind_mRNA_new_in_use = 3*ER_p*mRNA_new_in_use/rRNA_spacing_on_mRNA;

% unbind_mRNA = 3*ER_p*mRNA_in_use/rRNA_spacing_on_mRNA;

% unbind_mRNA = 3*ER_p*mRNA_in_use/rRNA_spacing_on_mRNA + dk4_rRNA;

% unbind_mRNA_new_in_use = 0;

unbind_mRNA = unbind_rRNA*mRNA_in_use_conc/rRNA_in_use_conc;

unbind_p = ATP_frac_avail*k_restructure*p_in_use_conc;

unbind_snRNA = k_spl*snRNA_in_use_conc;

activate_mRNA = k_activate_mRNA*mRNA_inactive_conc*p_eIF_4_cytosol_conc;

% RNA polymerase

txs_mRNA_poly = ATP_frac_avail*k_mRNA_txs*polyII*poly_loci_frac...

*(RNA_poly_loci_constitutive + TF_grow_on_DNA);

% txs_mRNA_poly = ATP_frac_avail*k_mRNA_txs*polyII*poly_loci_frac...

% *RNA_poly_loci_constitutive;

spl_mRNA_poly = ATP_frac_avail*spl_frac*mRNA_poly_unspl;

nm_out_mRNA_poly = ATP_frac_avail*k_nm_mRNA*mRNA_poly_nuc_conc;

activate_mRNA_poly = activate_mRNA*mRNA_poly_inactive/mRNA_inactive;

bind_mRNA_poly = bind_mRNA*mRNA_poly_cytosol/mRNA_cytosol;

unbind_mRNA_poly = unbind_mRNA*mRNA_poly_in_use/mRNA_in_use;

% bind_mRNA_poly = eIF_4*k_bind_rRNA_mRNA*mRNA_poly_cytosol_conc*rRNA_cytosol_conc;

% unbind_mRNA_poly = 3*ER_p*mRNA_poly_in_use/rRNA_spacing_on_mRNA;

dk1_mRNA_poly = k_dk_mRNA*mRNA_poly_cytosol;

dk2_mRNA_poly = k_dk_mRNA*mRNA_poly_unspl;

dk3_mRNA_poly = k_dk_mRNA*mRNA_poly_nuc;

dk4_mRNA_poly = k_dk_mRNA*mRNA_poly_inactive;

dk5_mRNA_poly = k_dk_mRNA*mRNA_poly_in_use;

cut_mRNA_poly = k_cut_mRNA*mRNA_poly_trash;

txl_p_poly = unbind_rRNA*(mRNA_poly_cytosol + mRNA_poly_in_use)...

/(mRNA_cytosol + mRNA_in_use);

fold_p_poly = ATP_frac_avail*k_fold_p*p_poly_unfold_conc;

nm_in_p_poly = ATP_frac_avail*k_nm_in_p*p_poly_cytosol_conc;

activate_p_poly_by_mitogen = k_activate_p_poly_by_mitogen...

*p_poly_nuc_inactive_conc...

*(RNA_poly_active_constitutive...

+ mitogen_stimulation);

% activate_p_poly_by_mitogen = k_activate_p_poly_by_mitogen...

% *p_poly_nuc_inactive_conc...

% *mitogen_stimulation;

deactivate_p_poly_by_cycC_Cdk8 = k_deactivate_p_poly_by_cycC_Cdk8...

*cycC_Cdk8_active_conc...

*p_poly_nuc_active_conc;

% bind_p_poly = max(0, k_bind_p_poly...

% *min(i_poly_avail/size_ratio, p_poly_nuc_conc));

bind_p_poly_to_DNA = k_bind_p_poly_to_DNA*p_poly_nuc_active_conc;

% unbind_p_poly_from_DNA = txs_mRNA + txs_rRNA + txs_snRNA + txs_tRNA;

unbind_p_poly_from_DNA = txs_mRNA + txs_rRNA + txs_snRNA + txs_tRNA...

+ k_unbind_p_poly_from_DNA_by_APC_Cdc20...

*Cdc20_APC_active_conc*p_poly_in_use_conc;

u_p_poly_unfold = ATP_frac_avail*k_dk_p*p_poly_unfold;

u_p_poly_cytosol = ATP_frac_avail*k_dk_p*p_poly_cytosol;

u_p_poly_nuc_active = ATP_frac_avail*k_dk_p*p_poly_nuc_active;

u_p_poly_nuc_inactive = ATP_frac_avail*k_dk_p*p_poly_nuc_inactive;

u_p_poly_in_use = ATP_frac_avail*k_dk_p*p_poly_in_use;

cut_p_poly = ATP_frac_avail*k_cut_p*p_poly_trash_conc;

% eIF_4

txs_mRNA_eIF_4 = ATP_frac_avail*k_mRNA_txs*polyII*eIF_4_loci_frac;

spl_mRNA_eIF_4 = ATP_frac_avail*spl_frac*mRNA_eIF_4_unspl;

nm_out_mRNA_eIF_4 = ATP_frac_avail*k_nm_mRNA*mRNA_eIF_4_nuc_conc;

activate_mRNA_eIF_4 = activate_mRNA*mRNA_eIF_4_inactive/mRNA_inactive;

bind_mRNA_eIF_4 = bind_mRNA*mRNA_eIF_4_cytosol/mRNA_cytosol;

unbind_mRNA_eIF_4 = unbind_mRNA*mRNA_eIF_4_in_use/mRNA_in_use;

dk1_mRNA_eIF_4 = k_dk_mRNA*mRNA_eIF_4_cytosol;

dk2_mRNA_eIF_4 = k_dk_mRNA*mRNA_eIF_4_unspl;

dk3_mRNA_eIF_4 = k_dk_mRNA*mRNA_eIF_4_nuc;

dk4_mRNA_eIF_4 = k_dk_mRNA*mRNA_eIF_4_inactive;

dk5_mRNA_eIF_4 = k_dk_mRNA*mRNA_eIF_4_in_use;

cut_mRNA_eIF_4 = k_cut_mRNA*mRNA_eIF_4_trash;

txl_p_eIF_4 = unbind_rRNA*(mRNA_eIF_4_cytosol + mRNA_eIF_4_in_use)...

/(mRNA_cytosol + mRNA_in_use);

fold_p_eIF_4 = ATP_frac_avail*k_fold_p*p_eIF_4_unfold_conc;

% bind_p_eIF_4 = max(0, k_activate_mRNA*mRNA_cytosol_conc...

% *min(i_eIF_4_avail/size_ratio, p_eIF_4_cytosol_conc));

bind_p_eIF_4 = activate_mRNA;

unbind_p_eIF_4 = dk1_mRNA + dk4_mRNA;

u1_p_eIF_4 = ATP_frac_avail*k_dk_p*p_eIF_4_cytosol_conc;

u2_p_eIF_4 = ATP_frac_avail*k_dk_p*p_eIF_4_unfold_conc;

% u3_p_eIF_4 = ATP_frac_avail*k_dk_p*p_eIF_4_in_use_conc;

u3_p_eIF_4 = 0;

cut_p_eIF_4 = ATP_frac_avail*k_cut_p*p_eIF_4_trash_conc;

% ****************** calculate ratios to later correct for ubiq and decay of partner molecules

% for correction of ubiq and decay of p27 on cycD, cycE, and cycA

% (NOTE the idea here is to divide the released cyclins amongst the cyclins according to the

% fraction that is in the cyc_Cdk2_on_p27_inactive state for each cyclin)

u_p27_by_SCF_Skp2_fast = ATP_frac_avail*k_u_p27_by_SCF_Skp2_fast...

*Skp2_SCF_active_conc;

u_p27_by_APC_Cdc20 = ATP_frac_avail*k_u_p27_by_APC_Cdc20*Cdc20_APC_active_conc;

u_p27_by_KPC = ATP_frac_avail*k_u_p27_by_KPC*KPC_active_conc;

u_p27_fast = u_p27_by_SCF_Skp2_fast + u_p27_by_APC_Cdc20 + u_p27_by_KPC;

u_p27_on_cyclins = u_p27_fast*p27_on_cyclins_conc...

+ ATP_frac_avail*k_dk_p*p27_on_cyclins;

cyc_on_p27_tot = cycD_Cdk4or6_on_p27_inactive...

+ cycE_Cdk2_on_p27_inactive...

+ cycA_Cdk2_on_p27_inactive;

if(cyc_on_p27_tot <= 0E-9)

u_p27_on_cycD_Cdk4or6_frac = 0E-9;

u_p27_on_cycE_Cdk2_frac = 0E-9;

u_p27_on_cycA_Cdk2_frac = 0E-9;

else

u_p27_on_cycD_Cdk4or6_frac = u_p27_on_cyclins...

*cycD_Cdk4or6_on_p27_inactive/cyc_on_p27_tot;

u_p27_on_cycE_Cdk2_frac = u_p27_on_cyclins...

*cycE_Cdk2_on_p27_inactive/cyc_on_p27_tot;

u_p27_on_cycA_Cdk2_frac = u_p27_on_cyclins...

*cycA_Cdk2_on_p27_inactive/cyc_on_p27_tot;

end

% for correction of decay of Cdk2 on p27 (assume Cdk4or6 does not decay)

u_Cdk2_on_cycE = ATP_frac_avail*k_dk_p*Cdk2_on_cycE;

u_Cdk2_on_cycA = ATP_frac_avail*k_dk_p*Cdk2_on_cycA;

Cdk2_on_cycE_tot = cycE_Cdk2_inactive + cycE_Cdk2_active...

+ cycE_Cdk2_on_p27_inactive;

if(Cdk2_on_cycE_tot <= 0E-9)

u_Cdk2_on_cycE_p27 = 0E-9;

else

u_Cdk2_on_cycE_p27 = u_Cdk2_on_cycE*cycE_Cdk2_on_p27_inactive/Cdk2_on_cycE_tot;

end

Cdk2_on_cycA_tot = cycA_Cdk2_inactive + cycA_Cdk2_active...

+ cycA_Cdk2_on_p27_inactive;

if(Cdk2_on_cycA_tot <= 0E-9)

u_Cdk2_on_cycA_p27 = 0E-9;

else

u_Cdk2_on_cycA_p27 = u_Cdk2_on_cycA*cycA_Cdk2_on_p27_inactive/Cdk2_on_cycA_tot;

end

% for correction of decay of Cdk2 on cycE

if(Cdk2_on_cycE_tot <= 0E-9)

u_Cdk2_on_cycE_inactive_frac = 0E-9;

u_Cdk2_on_cycE_active_frac = 0E-9;

u_Cdk2_on_cycE_on_p27_inactive_frac = 0E-9;

else

u_Cdk2_on_cycE_inactive_frac = u_Cdk2_on_cycE...

*cycE_Cdk2_inactive/Cdk2_on_cycE_tot;

u_Cdk2_on_cycE_active_frac = u_Cdk2_on_cycE...

*cycE_Cdk2_active/Cdk2_on_cycE_tot;

u_Cdk2_on_cycE_on_p27_inactive_frac = u_Cdk2_on_cycE...

*cycE_Cdk2_on_p27_inactive/Cdk2_on_cycE_tot;

end

% for correction of decay of Cdk2 on cycA

if(Cdk2_on_cycA_tot <= 0E-9)

u_Cdk2_on_cycA_inactive_frac = 0E-9;

u_Cdk2_on_cycA_active_frac = 0E-9;

u_Cdk2_on_cycA_on_p27_inactive_frac = 0E-9;

else

u_Cdk2_on_cycA_inactive_frac = u_Cdk2_on_cycA...

*cycA_Cdk2_inactive/Cdk2_on_cycA_tot;

u_Cdk2_on_cycA_active_frac = u_Cdk2_on_cycA...

*cycA_Cdk2_active/Cdk2_on_cycA_tot;

u_Cdk2_on_cycA_on_p27_inactive_frac = u_Cdk2_on_cycA...

*cycA_Cdk2_on_p27_inactive/Cdk2_on_cycA_tot;

end

% for correction of decay of Cdk1 on cycA

u_Cdk1_on_cycB = ATP_frac_avail*k_dk_p*Cdk1_on_cycB;

u_Cdk1_on_cycA = ATP_frac_avail*k_dk_p*Cdk1_on_cycA;

Cdk1_on_cycA_tot = cycA_Cdk1_cytosol_active + cycA_Cdk1_phospho_inactive...

+ cycA_Cdk1_nuc_active;

if(Cdk1_on_cycA_tot <= 0E-9)

u_Cdk1_on_cycA_cytosol_active_frac = 0E-9;

u_Cdk1_on_cycA_phospho_inactive_frac = 0E-9;

u_Cdk1_on_cycA_nuc_active_frac = 0E-9;

else

u_Cdk1_on_cycA_cytosol_active_frac = u_Cdk1_on_cycA...

*cycA_Cdk1_cytosol_active...

/Cdk1_on_cycA_tot;

u_Cdk1_on_cycA_phospho_inactive_frac = u_Cdk1_on_cycA...

*cycA_Cdk1_phospho_inactive...

/Cdk1_on_cycA_tot;

u_Cdk1_on_cycA_nuc_active_frac = u_Cdk1_on_cycA...

*cycA_Cdk1_nuc_active/Cdk1_on_cycA_tot;

end

% for correction of decay of Cdk1 on cycB

Cdk1_on_cycB_tot = cycB_Cdk1_cytosol_active + cycB_Cdk1_phospho_inactive...

+ cycB_Cdk1_nuc_active;

if(Cdk1_on_cycB_tot <= 0E-9)

u_Cdk1_on_cycB_cytosol_active_frac = 0E-9;

u_Cdk1_on_cycB_phospho_inactive_frac = 0E-9;

u_Cdk1_on_cycB_nuc_active_frac = 0E-9;

else

u_Cdk1_on_cycB_cytosol_active_frac = u_Cdk1_on_cycB...

*cycB_Cdk1_cytosol_active...

/Cdk1_on_cycB_tot;

u_Cdk1_on_cycB_phospho_inactive_frac = u_Cdk1_on_cycB...

*cycB_Cdk1_phospho_inactive...

/Cdk1_on_cycB_tot;

u_Cdk1_on_cycB_nuc_active_frac = u_Cdk1_on_cycB...

*cycB_Cdk1_nuc_active...

/Cdk1_on_cycB_tot;

end

% for correction of decay of APC on Cdc20

u_APC_on_Cdc20 = ATP_frac_avail*k_dk_p*APC_on_Cdc20;

APC_on_Cdc20_tot = Cdc20_APC_inactive + Cdc20_APC_active;

if(APC_on_Cdc20_tot <= 0E-9)

u_APC_on_Cdc20_inactive_frac = 0E-9;

u_APC_on_Cdc20_active_frac = 0E-9;

else

u_APC_on_Cdc20_inactive_frac = u_APC_on_Cdc20...

*Cdc20_APC_inactive/APC_on_Cdc20_tot;

u_APC_on_Cdc20_active_frac = u_APC_on_Cdc20...

*Cdc20_APC_active/APC_on_Cdc20_tot;

end

% 2 Rb RATES

txs_mRNA_Rb = ATP_frac_avail*k_mRNA_txs*polyII*Rb_loci_frac...

/max(1, E2F_on_DNA);

spl_mRNA_Rb = ATP_frac_avail*spl_frac*mRNA_Rb_unspl;

nm_out_mRNA_Rb = ATP_frac_avail*k_nm_mRNA*mRNA_Rb_nuc_conc;

activate_mRNA_Rb = activate_mRNA*mRNA_Rb_inactive/mRNA_inactive;

bind_mRNA_Rb = bind_mRNA*mRNA_Rb_cytosol/mRNA_cytosol;

unbind_mRNA_Rb = unbind_mRNA*mRNA_Rb_in_use/mRNA_in_use;

dk_mRNA_Rb_cytosol = k_dk_mRNA*mRNA_Rb_cytosol;

dk_mRNA_Rb_unspl = k_dk_mRNA*mRNA_Rb_unspl;

dk_mRNA_Rb_nuc = k_dk_mRNA*mRNA_Rb_nuc;

dk_mRNA_Rb_inactive = k_dk_mRNA*mRNA_Rb_inactive;

dk_mRNA_Rb_in_use = k_dk_mRNA*mRNA_Rb_in_use;

cut_mRNA_Rb = k_cut_mRNA*mRNA_Rb_trash;

txl_Rb = unbind_rRNA*(mRNA_Rb_cytosol + mRNA_Rb_in_use)...

/(mRNA_cytosol + mRNA_in_use);

fold_Rb = ATP_frac_avail*k_fold_p*Rb_unfold_conc;

nm_in_Rb = ATP_frac_avail*k_nm_in_p*Rb_cytosol_conc;

bind_Rb_to_E2F = k_bind_Rb_to_E2F*Rb_nuc_conc*E2F_nuc_conc;

unbind_Rb_E2F = k_unbind_Rb_E2F*Rb_on_E2F_conc;

% cyclins phosphorylate Rb

Cdk_active_conc = cycD_Cdk4or6_active_conc...

+ cycD_Cdk4or6_on_p27_inactive...

+ cycE_Cdk2_active_conc + cycA_Cdk2_active_conc...

+ cycA_Cdk1_nuc_active_conc + cycB_Cdk1_nuc_active_conc;

phospho_Rb_nuc_by_Cdk = k_phospho_Rb_by_Cdk*Cdk_active_conc*Rb_nuc_conc;

phospho_Rb_on_E2F_by_Cdk = k_phospho_Rb_by_Cdk*Cdk_active_conc*Rb_on_E2F_conc;

% cyclins keep Rb phosphorylated

unphospho_Rb = k_unphospho_Rb*Rb_phospho_inactive_conc...

+ k_dephospho_Rb_by_Cdc14*Rb_phospho_inactive_conc...

*Cdc14_phospho_active_conc;

u_Rb_unfold = ATP_frac_avail*k_dk_p*Rb_unfold;

u_Rb_cytosol = ATP_frac_avail*k_dk_p*Rb_cytosol;

u_Rb_nuc = ATP_frac_avail*k_dk_p*Rb_nuc;

u_Rb_on_E2F = ATP_frac_avail*k_dk_p*Rb_on_E2F;

u_Rb_phospho_inactive = ATP_frac_avail*k_dk_p*Rb_phospho_inactive;

cut_Rb = ATP_frac_avail*k_cut_p*Rb_trash_conc;

% 3 cycD RATES

% mitogen_stimulation = 0 or 1; --> see define_const.in and makedynfiles.f

% txs_mRNA_cycD = ATP_frac_avail*k_mRNA_txs*polyII*cycD_loci_frac...

% *mitogen_stimulation;

% txs_mRNA_cycD = ATP_frac_avail*k_mRNA_txs*polyII*cycD_loci_frac...

% *mitogen_stimulation*B_Myb_on_DNA;

txs_mRNA_cycD = ATP_frac_avail*k_mRNA_txs*polyII*cycD_loci_frac...

*mitogen_stimulation...

*(cycD_constitutive + B_Myb_on_DNA);

spl_mRNA_cycD = ATP_frac_avail*spl_frac*mRNA_cycD_unspl;

nm_out_mRNA_cycD = ATP_frac_avail*k_nm_mRNA*mRNA_cycD_nuc_conc;

activate_mRNA_cycD = activate_mRNA*mRNA_cycD_inactive/mRNA_inactive;

bind_mRNA_cycD = bind_mRNA*mRNA_cycD_cytosol/mRNA_cytosol;

unbind_mRNA_cycD = unbind_mRNA*mRNA_cycD_in_use/mRNA_in_use;

dk_mRNA_cycD_cytosol = k_dk_mRNA*mRNA_cycD_cytosol;

dk_mRNA_cycD_unspl = k_dk_mRNA*mRNA_cycD_unspl;

dk_mRNA_cycD_nuc = k_dk_mRNA*mRNA_cycD_nuc;

dk_mRNA_cycD_inactive = k_dk_mRNA*mRNA_cycD_inactive;

dk_mRNA_cycD_in_use = k_dk_mRNA*mRNA_cycD_in_use;

cut_mRNA_cycD = k_cut_mRNA*mRNA_cycD_trash;

txl_cycD = unbind_rRNA*(mRNA_cycD_cytosol + mRNA_cycD_in_use)...

/(mRNA_cytosol + mRNA_in_use);

fold_cycD = ATP_frac_avail*k_fold_p*cycD_unfold_conc;

nm_in_cycD = ATP_frac_avail*k_nm_in_p*cycD_cytosol_conc;

bind_cycD_to_Cdk4or6 = k_bind_cycD_to_Cdk4or6*cycD_nuc_conc;

% note -- I originally had unbinding due to Cdk4or6 decay

% unbind_cycD_Cdk4or6 = ATP_frac_avail*k_dk_p*cycD_Cdk4or6_active;

unbind_cycD_Cdk4or6 = k_unbind_cycD_Cdk4or6*cycD_Cdk4or6_active;

% ASSUME p27 does not inhibit cycD/Cdk4or6, so I set these rate constants to 0

bind_cycD_Cdk4or6_to_p27 = k_bind_cycD_Cdk4or6_to_p27*cycD_Cdk4or6_active_conc...

*p27_nuc_conc;

unbind_cycD_Cdk4or6_p27 = k_unbind_cycD_Cdk4or6_p27...

*cycD_Cdk4or6_on_p27_inactive_conc;

u_cycD_by_SCF_Skp2 = ATP_frac_avail*k_u_cycD_by_SCF_Skp2*Skp2_SCF_active_conc;

% some "other" ubiquinates cycD -- ASSUME it is Cdc20

u_cycD_by_APC_Cdc20 = ATP_frac_avail*k_u_cycD_by_APC_Cdc20...

*Cdc20_APC_active_conc;

u_cycD = u_cycD_by_SCF_Skp2 + u_cycD_by_APC_Cdc20;

u_cycD_unfold = ATP_frac_avail*k_dk_p*cycD_unfold;

u_cycD_cytosol = ATP_frac_avail*k_dk_p*cycD_cytosol;

u_cycD_nuc = u_cycD*cycD_nuc_conc...

+ ATP_frac_avail*k_dk_p*cycD_nuc;

u_cycD_Cdk4or6_active = u_cycD*cycD_Cdk4or6_active_conc...

+ ATP_frac_avail*k_dk_p*cycD_Cdk4or6_active;

u_cycD_Cdk4or6_on_p27_inactive = u_cycD...

*cycD_Cdk4or6_on_p27_inactive_conc...

+ ATP_frac_avail...

*k_dk_p*cycD_Cdk4or6_on_p27_inactive;

cut_cycD = ATP_frac_avail*k_cut_p*cycD_trash_conc;

cycD_conc_all = cycD_nuc_conc + cycD_Cdk4or6_active_conc...

+ cycD_Cdk4or6_on_p27_inactive_conc;

energy_u_cycD_by_SCF_Skp2 = ATP_u*u_cycD_by_SCF_Skp2*cycD_conc_all;

% energy_u_cycD_by_APC_Cdh1 = ATP_u*u_cycD_by_APC_Cdh1*cycD_conc_all;

energy_u_cycD_by_APC_Cdc20 = ATP_u*u_cycD_by_APC_Cdc20*cycD_conc_all;

% 5 cycE RATES

txs_mRNA_cycE = ATP_frac_avail*k_mRNA_txs*polyII*cycE_loci_frac*E2F_on_DNA;

spl_mRNA_cycE = ATP_frac_avail*spl_frac*mRNA_cycE_unspl;

nm_out_mRNA_cycE = ATP_frac_avail*k_nm_mRNA*mRNA_cycE_nuc_conc;

activate_mRNA_cycE = activate_mRNA*mRNA_cycE_inactive/mRNA_inactive;

bind_mRNA_cycE = bind_mRNA*mRNA_cycE_cytosol/mRNA_cytosol;

unbind_mRNA_cycE = unbind_mRNA*mRNA_cycE_in_use/mRNA_in_use;

dk_mRNA_cycE_cytosol = k_dk_mRNA*mRNA_cycE_cytosol;

dk_mRNA_cycE_unspl = k_dk_mRNA*mRNA_cycE_unspl;

dk_mRNA_cycE_nuc = k_dk_mRNA*mRNA_cycE_nuc;

dk_mRNA_cycE_inactive = k_dk_mRNA*mRNA_cycE_inactive;

dk_mRNA_cycE_in_use = k_dk_mRNA*mRNA_cycE_in_use;

cut_mRNA_cycE = k_cut_mRNA*mRNA_cycE_trash;

txl_cycE = unbind_rRNA*(mRNA_cycE_cytosol + mRNA_cycE_in_use)...

/(mRNA_cytosol + mRNA_in_use);

fold_cycE = ATP_frac_avail*k_fold_p*cycE_unfold_conc;

nm_in_cycE = ATP_frac_avail*k_nm_in_p*cycE_cytosol_conc;

bind_cycE_to_Cdk2 = k_bind_cycE_to_Cdk2*cycE_nuc_conc*Cdk2_nuc_conc;

unbind_cycE_Cdk2 = k_unbind_cycE_Cdk2*cycE_Cdk2_inactive_conc;

% cycE/Cdk2 is activated (dephospohorylated) by Cdc25A

% (any Cdc25, but only Cdc25A is nuclear at this time)

dephospho_cycE_Cdk2 = k_dephospho_cycE_Cdk2*cycE_Cdk2_inactive_conc;

dephospho_cycE_Cdk2_by_Cdc25A = k_dephospho_cycE_Cdk2_by_Cdc25A*cycE_Cdk2_inactive_conc...

*Cdc25A_phospho_active_conc;

phospho_cycE_Cdk2 = k_phospho_cycE_Cdk2*cycE_Cdk2_active_conc;

% cycE/Cdk2 is inhibited by p27

% note that unbinding p27 entails phospho of p27, which is assumed to be automatic by Cdk2

bind_cycE_Cdk2_to_p27 = k_bind_cycE_Cdk2_to_p27*p27_nuc_conc...

*cycE_Cdk2_inactive_conc;

unbind_cycE_Cdk2_p27 = k_unbind_cycE_Cdk2_p27*cycE_Cdk2_on_p27_inactive_conc;

% cycE/Cdk2 is ubiq by SCF(Fbw7); cycE free (in nucleus) is ubiq by SCF(Skp2)

u_cycE_by_SCF_Skp2 = ATP_frac_avail*k_u_cycE_by_SCF_Skp2*Skp2_SCF_active_conc;

u_cycE_by_SCF_Fbw7 = ATP_frac_avail*k_u_cycE_by_SCF_Fbw7*Fbw7_SCF_active_conc;

u_cycE_unfold = ATP_frac_avail*k_dk_p*cycE_unfold;

u_cycE_cytosol = ATP_frac_avail*k_dk_p*cycE_cytosol;

u_cycE_nuc = u_cycE_by_SCF_Skp2*cycE_nuc_conc...

+ ATP_frac_avail*k_dk_p*cycE_nuc;

u_cycE_Cdk2_inactive = u_cycE_by_SCF_Fbw7*cycE_Cdk2_inactive_conc...

+ ATP_frac_avail*k_dk_p*cycE_Cdk2_inactive;

u_cycE_Cdk2_active = u_cycE_by_SCF_Fbw7*cycE_Cdk2_active_conc...

+ ATP_frac_avail*k_dk_p*cycE_Cdk2_active;

u_cycE_Cdk2_on_p27_inactive = u_cycE_by_SCF_Fbw7*cycE_Cdk2_on_p27_inactive_conc...

+ ATP_frac_avail*k_dk_p*cycE_Cdk2_on_p27_inactive;

cut_cycE = ATP_frac_avail*k_cut_p*cycE_trash_conc;

u_cycE_by_SCF_Skp2_all = u_cycE_by_SCF_Skp2*cycE_nuc_conc;

u_cycE_by_SCF_Fbw7_all = u_cycE_by_SCF_Fbw7...

*(cycE_Cdk2_inactive_conc + cycE_Cdk2_active_conc...

+ cycE_Cdk2_on_p27_inactive_conc);

energy_u_cycE_by_SCF_Skp2 = ATP_u*u_cycE_by_SCF_Skp2_all;

energy_u_cycE_by_SCF_Fbw7 = ATP_u*u_cycE_by_SCF_Fbw7_all;

% 6 B-Myb RATES

% log10_B_Myb_on_DNA = log10(max(10, B_Myb_on_DNA));

% txs_mRNA_B_Myb = ATP_frac_avail*k_mRNA_txs*polyII*B_Myb_loci_frac...

% *E2F_on_DNA*log10_B_Myb_on_DNA;

txs_mRNA_B_Myb = ATP_frac_avail*k_mRNA_txs*polyII*B_Myb_loci_frac...

*(E2F_on_DNA + B_Myb_on_DNA);

spl_mRNA_B_Myb = ATP_frac_avail*spl_frac*mRNA_B_Myb_unspl;

nm_out_mRNA_B_Myb = ATP_frac_avail*k_nm_mRNA*mRNA_B_Myb_nuc_conc;

activate_mRNA_B_Myb = activate_mRNA*mRNA_B_Myb_inactive/mRNA_inactive;

bind_mRNA_B_Myb = bind_mRNA*mRNA_B_Myb_cytosol/mRNA_cytosol;

unbind_mRNA_B_Myb = unbind_mRNA*mRNA_B_Myb_in_use/mRNA_in_use;

dk_mRNA_B_Myb_cytosol = k_dk_mRNA*mRNA_B_Myb_cytosol;

dk_mRNA_B_Myb_unspl = k_dk_mRNA*mRNA_B_Myb_unspl;

dk_mRNA_B_Myb_nuc = k_dk_mRNA*mRNA_B_Myb_nuc;

dk_mRNA_B_Myb_inactive = k_dk_mRNA*mRNA_B_Myb_inactive;

dk_mRNA_B_Myb_in_use = k_dk_mRNA*mRNA_B_Myb_in_use;

cut_mRNA_B_Myb = k_cut_mRNA*mRNA_B_Myb_trash;

txl_B_Myb = unbind_rRNA*(mRNA_B_Myb_cytosol + mRNA_B_Myb_in_use)...

/(mRNA_cytosol + mRNA_in_use);

fold_B_Myb = ATP_frac_avail*k_fold_p*B_Myb_unfold_conc;

nm_in_B_Myb = ATP_frac_avail*k_nm_in_p*B_Myb_cytosol_conc;

Cdk2_active_conc = cycE_Cdk2_active_conc + cycA_Cdk2_active_conc;

phospho_B_Myb_by_cyc_Cdk = k_phospho_B_Myb_by_cyc_Cdk*B_Myb_nuc_conc...

*Cdk2_active_conc;

unphospho_B_Myb = 0;

% bind_B_Myb_to_DNA = k_bind_B_Myb_to_DNA*B_Myb_phospho_active_conc;

bind_B_Myb_to_DNA = min(k_bind_B_Myb_to_DNA*B_Myb_phospho_active_conc, ...

k_bind_B_Myb_to_DNA*max(0, sat_B_Myb_on_DNA - B_Myb_on_DNA));

% unbind_B_Myb_from_DNA = k_unbind_B_Myb_from_DNA*B_Myb_on_DNA_conc;

unbind_B_Myb_from_DNA = k_unbind_B_Myb_from_DNA*B_Myb_on_DNA;

u_B_Myb_by_SCF_Skp2 = ATP_frac_avail*k_u_B_Myb_by_SCF_Skp2*Skp2_SCF_active_conc;

u_B_Myb_unfold = ATP_frac_avail*k_dk_p*B_Myb_unfold;

u_B_Myb_cytosol = ATP_frac_avail*k_dk_p*B_Myb_cytosol;

u_B_Myb_nuc = u_B_Myb_by_SCF_Skp2*B_Myb_nuc_conc...

+ ATP_frac_avail*k_dk_p*B_Myb_nuc;

u_B_Myb_phospho_active = u_B_Myb_by_SCF_Skp2*B_Myb_phospho_active_conc...

+ ATP_frac_avail*k_dk_p*B_Myb_phospho_active;

% ASSUME no ubiq of B-Myb while it is on DNA

u_B_Myb_on_DNA = ATP_frac_avail*k_dk_p*B_Myb_on_DNA;

cut_B_Myb = ATP_frac_avail*k_cut_p*B_Myb_trash_conc;

energy_u_B_Myb_by_SCF_Skp2 = ATP_u*u_B_Myb_by_SCF_Skp2...

*(B_Myb_nuc_conc + B_Myb_phospho_active_conc);

% 7 NF-Y RATES

txs_mRNA_NF_Y = ATP_frac_avail*k_mRNA_txs*polyII*NF_Y_loci_frac...

*(NF_Y_constitutive + E2F_on_DNA);

spl_mRNA_NF_Y = ATP_frac_avail*spl_frac*mRNA_NF_Y_unspl;

nm_out_mRNA_NF_Y = ATP_frac_avail*k_nm_mRNA*mRNA_NF_Y_nuc_conc;

activate_mRNA_NF_Y = activate_mRNA*mRNA_NF_Y_inactive/mRNA_inactive;

bind_mRNA_NF_Y = bind_mRNA*mRNA_NF_Y_cytosol/mRNA_cytosol;

unbind_mRNA_NF_Y = unbind_mRNA*mRNA_NF_Y_in_use/mRNA_in_use;

dk_mRNA_NF_Y_cytosol = k_dk_mRNA*mRNA_NF_Y_cytosol;

dk_mRNA_NF_Y_unspl = k_dk_mRNA*mRNA_NF_Y_unspl;

dk_mRNA_NF_Y_nuc = k_dk_mRNA*mRNA_NF_Y_nuc;

dk_mRNA_NF_Y_inactive = k_dk_mRNA*mRNA_NF_Y_inactive;

dk_mRNA_NF_Y_in_use = k_dk_mRNA*mRNA_NF_Y_in_use;

cut_mRNA_NF_Y = k_cut_mRNA*mRNA_NF_Y_trash;

txl_NF_Y = unbind_rRNA*(mRNA_NF_Y_cytosol + mRNA_NF_Y_in_use)...

/(mRNA_cytosol + mRNA_in_use);

fold_NF_Y = ATP_frac_avail*k_fold_p*NF_Y_unfold_conc;

nm_in_NF_Y = ATP_frac_avail*k_nm_in_p*NF_Y_cytosol_conc;

Cdk2_active_conc = cycE_Cdk2_active_conc + cycA_Cdk2_active_conc;

phospho_NF_Y_by_cyc_Cdk = k_phospho_NF_Y_by_cyc_Cdk*NF_Y_nuc_conc...

*Cdk2_active_conc;

unphospho_NF_Y = k_unphospho_NF_Y*NF_Y_phospho_active_conc;

dephospho_NF_Y_by_Cdc14 = k_dephospho_NF_Y_by_Cdc14*Cdc14_phospho_active_conc...

*NF_Y_phospho_active_conc;

% bind_NF_Y_to_DNA = k_bind_NF_Y_to_DNA*NF_Y_phospho_active_conc;

bind_NF_Y_to_DNA = min(k_bind_NF_Y_to_DNA*NF_Y_phospho_active_conc, ...

k_bind_NF_Y_to_DNA*max(0, sat_NF_Y_on_DNA - NF_Y_on_DNA));

unbind_NF_Y_from_DNA = k_unbind_NF_Y_from_DNA*NF_Y_on_DNA_conc;

% NOTE -- NF-Y probably should be ubiquinated by SCF(Btrc) or APC(Cdc20)

u_NF_Y_unfold = ATP_frac_avail*k_dk_p*NF_Y_unfold;

u_NF_Y_cytosol = ATP_frac_avail*k_dk_p*NF_Y_cytosol;

u_NF_Y_nuc = ATP_frac_avail*k_dk_p*NF_Y_nuc;

u_NF_Y_phospho_active = ATP_frac_avail*k_dk_p*NF_Y_phospho_active;

u_NF_Y_on_DNA = ATP_frac_avail*k_dk_p*NF_Y_on_DNA;

cut_NF_Y = ATP_frac_avail*k_cut_p*NF_Y_trash_conc;

% 8 E2F RATES

txs_mRNA_E2F = ATP_frac_avail*k_mRNA_txs*polyII*E2F_loci_frac...

*(E2F_constituitive + E2F_on_DNA);

% /max(1, B_Myb_on_DNA);

spl_mRNA_E2F = ATP_frac_avail*spl_frac*mRNA_E2F_unspl;

nm_out_mRNA_E2F = ATP_frac_avail*k_nm_mRNA*mRNA_E2F_nuc_conc;

activate_mRNA_E2F = activate_mRNA*mRNA_E2F_inactive/mRNA_inactive;

bind_mRNA_E2F = bind_mRNA*mRNA_E2F_cytosol/mRNA_cytosol;

unbind_mRNA_E2F = unbind_mRNA*mRNA_E2F_in_use/mRNA_in_use;

dk_mRNA_E2F_cytosol = k_dk_mRNA*mRNA_E2F_cytosol;

dk_mRNA_E2F_unspl = k_dk_mRNA*mRNA_E2F_unspl;

dk_mRNA_E2F_nuc = k_dk_mRNA*mRNA_E2F_nuc;

dk_mRNA_E2F_inactive = k_dk_mRNA*mRNA_E2F_inactive;

dk_mRNA_E2F_in_use = k_dk_mRNA*mRNA_E2F_in_use;

cut_mRNA_E2F = k_cut_mRNA*mRNA_E2F_trash;

txl_E2F = unbind_rRNA*(mRNA_E2F_cytosol + mRNA_E2F_in_use)...

/(mRNA_cytosol + mRNA_in_use);

fold_E2F = ATP_frac_avail*k_fold_p*E2F_unfold_conc;

nm_in_E2F = ATP_frac_avail*k_nm_in_p*E2F_cytosol_conc;

bind_E2F_to_DNA = min(k_bind_E2F_to_DNA*E2F_nuc_conc, ...

k_bind_E2F_to_DNA*max(0, sat_E2F_on_DNA - E2F_on_DNA));

% Cdk_all_conc = cycA_Cdk2_active_conc...

% + cycA_Cdk1_nuc_active_conc + cycB_Cdk1_nuc_active_conc;

% unbind_E2F_DNA = k_E2F_phospho_by_cyc_Cdk*Cdk_all_conc*E2F_on_DNA_conc;

% unbind_E2F_DNA = k_E2F_phospho_by_cyc_Cdk*Cdk_all_conc*E2F_on_DNA;

% unbind_E2F_DNA = (k_E2F_phospho_by_cyc_Cdk*Cdk_all_conc...

% + k_E2F_phospho_by_cycD_Cdk4or6*cycD_Cdk4or6_active...

% + k_unbind_E2F_DNA)...

% *E2F_on_DNA;

unbind_E2F_DNA = (k_E2F_phospho_by_cycA_Cdk2*cycA_Cdk2_active_conc...

+ k_E2F_phospho_by_cycA_Cdk1*cycA_Cdk1_nuc_active_conc...

+ k_E2F_phospho_by_cycB_Cdk1*cycB_Cdk1_nuc_active_conc...

+ k_E2F_phospho_by_cycD_Cdk4or6*cycD_Cdk4or6_active...

+ k_unbind_E2F_DNA)...

*E2F_on_DNA;

bind_E2F_to_Rb = bind_Rb_to_E2F;

unbind_E2F_Rb = unbind_Rb_E2F + phospho_Rb_on_E2F_by_Cdk;

% E2F is ubiq by SCF(Skp2) (in nucleus) and "other"

% ASSUME (although it is probably not needed) SCF(Skp2) does not ubiq E2F_on_DNA

% ASSUME "other" is APC(Cdc20)

u_E2F_by_SCF_Skp2 = ATP_frac_avail*k_u_E2F_by_SCF_Skp2*Skp2_SCF_active_conc;

u_E2F_by_APC_Cdc20 = ATP_frac_avail*k_u_E2F_by_APC_Cdc20*Cdc20_APC_active_conc;

u_E2F = u_E2F_by_SCF_Skp2 + u_E2F_by_APC_Cdc20;

u_E2F_unfold = ATP_frac_avail*k_dk_p*E2F_unfold;

u_E2F_cytosol = ATP_frac_avail*k_dk_p*E2F_cytosol;

u_E2F_nuc = u_E2F*E2F_nuc_conc...

+ ATP_frac_avail*k_dk_p*E2F_nuc;

u_E2F_on_DNA = u_E2F_by_APC_Cdc20*E2F_on_DNA_conc...

+ ATP_frac_avail*k_dk_p*E2F_on_DNA;

u_E2F_Rb_inactive = u_E2F*E2F_Rb_inactive_conc...

+ ATP_frac_avail*k_dk_p*E2F_Rb_inactive;

cut_E2F = ATP_frac_avail*k_cut_p*E2F_trash_conc;

E2F_conc_some = E2F_nuc_conc + E2F_Rb_inactive_conc;

E2F_conc_all = E2F_nuc_conc + E2F_on_DNA_conc...

+ E2F_Rb_inactive_conc;

energy_u_E2F_by_SCF_Skp2 = ATP_u*u_E2F_by_SCF_Skp2*E2F_conc_some;

energy_u_E2F_by_ACP_Cdc20 = ATP_u*u_E2F_by_APC_Cdc20*E2F_conc_all;

% 9 cycA RATES

% adhesion_factor = 0 or 1; --> see define_const.in and makedynfiles.f

txs_mRNA_cycA = ATP_frac_avail*k_mRNA_txs*polyII*cycA_loci_frac...

*(mitogen_stimulation + adhesion_factor)...

*(E2F_on_DNA + NF_Y_on_DNA);

spl_mRNA_cycA = ATP_frac_avail*spl_frac*mRNA_cycA_unspl;

nm_out_mRNA_cycA = ATP_frac_avail*k_nm_mRNA*mRNA_cycA_nuc_conc;

activate_mRNA_cycA = activate_mRNA*mRNA_cycA_inactive/mRNA_inactive;

bind_mRNA_cycA = bind_mRNA*mRNA_cycA_cytosol/mRNA_cytosol;

unbind_mRNA_cycA = unbind_mRNA*mRNA_cycA_in_use/mRNA_in_use;

dk_mRNA_cycA_cytosol = k_dk_mRNA*mRNA_cycA_cytosol;

dk_mRNA_cycA_unspl = k_dk_mRNA*mRNA_cycA_unspl;

dk_mRNA_cycA_nuc = k_dk_mRNA*mRNA_cycA_nuc;

dk_mRNA_cycA_inactive = k_dk_mRNA*mRNA_cycA_inactive;

dk_mRNA_cycA_in_use = k_dk_mRNA*mRNA_cycA_in_use;

cut_mRNA_cycA = k_cut_mRNA*mRNA_cycA_trash;

txl_cycA = unbind_rRNA*(mRNA_cycA_cytosol + mRNA_cycA_in_use)...

/(mRNA_cytosol + mRNA_in_use);

fold_cycA = ATP_frac_avail*k_fold_p*cycA_unfold_conc;

nm_in_cycA = ATP_frac_avail*k_nm_in_p*cycA_cytosol_conc;

bind_cycA_to_Cdk2 = k_bind_cycA_to_Cdk2*cycA_nuc_conc*Cdk2_nuc_conc;

unbind_cycA_Cdk2 = k_unbind_cycA_Cdk2*cycA_Cdk2_inactive_conc;

dephospho_cycA_Cdk2_by_Cdc25A = k_dephospho_cycA_Cdk2_by_Cdc25A...

*cycA_Cdk2_inactive_conc...

*Cdc25A_phospho_active_conc;

phospho_cycA_Cdk2 = k_phospho_cycA_Cdk2*cycA_Cdk2_active_conc;

bind_cycA_Cdk2_to_p27 = k_bind_cycA_Cdk2_to_p27...

*(p27_nuc_conc/max(1, Skp2_nuc_conc))...

*cycA_Cdk2_inactive_conc;

unbind_cycA_Cdk2_p27 = k_unbind_cycA_Cdk2_p27*cycA_Cdk2_on_p27_inactive_conc;

bind_cycA_to_Cdk1 = k_bind_cycA_to_Cdk1*cycA_nuc_conc...

*Cdk1_in_cell_conc;

unbind_cycA_Cdk1 = k_unbind_cycA_Cdk1*cycA_Cdk1_cytosol_active_conc;

phospho_cycA_Cdk1_by_Wee1 = k_phospho_cycA_Cdk1_by_Wee1...

*Wee1_cytosol_active_conc*cycA_Cdk1_cytosol_active_conc...

+ k_phospho_cycA_Cdk1_by_phospho_Wee1...

*Wee1_phospho_inactive_conc*cycA_Cdk1_cytosol_active_conc;

unphospho_cycA_Cdk1_from_Wee1 = k_unphospho_cycA_Cdk1_from_Wee1...

*cycA_Cdk1_phospho_inactive_conc;

% cycA/Cdk1 is activated when dephosphorylated by Cdc25B or Cdc25C in cytosol

dephospho_cycA_Cdk1_by_Cdc25 = (k_dephospho_cycA_Cdk1_by_Cdc25A...

*Cdc25A_phospho_active_conc...

+ k_dephospho_cycA_Cdk1_by_Cdc25B...

*Cdc25B_cytosol_phospho_active_conc...

+ k_dephospho_cycA_Cdk1_by_Cdc25C...

*Cdc25C_cytosol_phospho_active_conc)...

*cycA_Cdk1_phospho_inactive_conc;

nm_in_cycA_Cdk1 = ATP_frac_avail*k_nm_in_cycA_Cdk1_by_Plk1...

*Plk1_cytosol_phospho_active_conc...

*cycA_Cdk1_cytosol_active_conc;

nm_out_cycA_Cdk1 = k_nm_out_cycA_Cdk1*cycA_Cdk1_nuc_active_conc;

% ASSUME SCF(Skp2) and APC(Cdc20) are nuclear

% ASSUME APC(Cdh1) is cellular

% NOTE -- Skp2 binds with cycA to protect it from p27 (Ji et al., 2006)

u_cycA_by_SCF_Skp2 = ATP_frac_avail*k_u_cycA_by_SCF_Skp2*Skp2_SCF_active_conc;

u_cycA_by_APC_Cdh1 = ATP_frac_avail*k_u_cycA_by_APC_Cdh1*Cdh1_APC_active_conc;

u_cycA_by_APC_Cdc20 = ATP_frac_avail*k_u_cycA_by_APC_Cdc20*Cdc20_APC_active_conc;

u_cycA = u_cycA_by_SCF_Skp2 + u_cycA_by_APC_Cdh1...

+ u_cycA_by_APC_Cdc20;

u_cycA_unfold = ATP_frac_avail*k_dk_p*cycA_unfold;

u_cycA_cytosol = u_cycA_by_APC_Cdh1*cycA_cytosol...

+ ATP_frac_avail*k_dk_p*cycA_cytosol;

u_cycA_Cdk1_cytosol_active = u_cycA_by_APC_Cdh1*cycA_Cdk1_cytosol_active_conc...

+ ATP_frac_avail*k_dk_p*cycA_Cdk1_cytosol_active;

u_cycA_Cdk1_phospho_inactive = u_cycA_by_APC_Cdh1*cycA_Cdk1_phospho_inactive_conc...

+ ATP_frac_avail*k_dk_p*cycA_Cdk1_phospho_inactive;

u_cycA_Cdk1_nuc_active = u_cycA*cycA_Cdk1_nuc_active_conc...

+ ATP_frac_avail*k_dk_p*cycA_Cdk1_nuc_active;

u_cycA_nuc = u_cycA*cycA_nuc_conc...

+ ATP_frac_avail*k_dk_p*cycA_nuc;

u_cycA_Cdk2_inactive = u_cycA*cycA_Cdk2_inactive_conc...

+ ATP_frac_avail*k_dk_p*cycA_Cdk2_inactive;

u_cycA_Cdk2_active = u_cycA*cycA_Cdk2_active_conc...

+ ATP_frac_avail*k_dk_p*cycA_Cdk2_active;

u_cycA_Cdk2_on_p27_inactive = u_cycA*cycA_Cdk2_on_p27_inactive_conc...

+ ATP_frac_avail*k_dk_p*cycA_Cdk2_on_p27_inactive;

cut_cycA = ATP_frac_avail*k_cut_p*cycA_trash_conc;

cycA_conc_all = cycA_nuc_conc...

+ cycA_Cdk2_inactive_conc + cycA_Cdk2_active_conc...

+ cycA_Cdk2_on_p27_inactive_conc...

+ cycA_Cdk1_nuc_active_conc;

energy_u_cycA_by_SCF_Skp2 = ATP_u*u_cycA_by_SCF_Skp2*cycA_conc_all;

energy_u_cycA_by_APC_Cdh1 = ATP_u*u_cycA_by_APC_Cdh1...

*(cycA_conc_all + cycA_cytosol_conc...

+ cycA_Cdk1_cytosol_active_conc...

+ cycA_Cdk1_phospho_inactive_conc);

energy_u_cycA_by_APC_Cdc20 = ATP_u*u_cycA_by_APC_Cdc20*cycA_conc_all;

% 1 p27 RATES

txs_mRNA_p27 = ATP_frac_avail*k_mRNA_txs*polyII*p27_loci_frac;

spl_mRNA_p27 = ATP_frac_avail*spl_frac*mRNA_p27_unspl;

nm_out_mRNA_p27 = ATP_frac_avail*k_nm_mRNA*mRNA_p27_nuc_conc;

activate_mRNA_p27 = activate_mRNA*mRNA_p27_inactive/mRNA_inactive;

bind_mRNA_p27 = bind_mRNA*mRNA_p27_cytosol/mRNA_cytosol;

unbind_mRNA_p27 = unbind_mRNA*mRNA_p27_in_use/mRNA_in_use;

dk_mRNA_p27_cytosol = k_dk_mRNA*mRNA_p27_cytosol;

dk_mRNA_p27_unspl = k_dk_mRNA*mRNA_p27_unspl;

dk_mRNA_p27_nuc = k_dk_mRNA*mRNA_p27_nuc;

dk_mRNA_p27_inactive = k_dk_mRNA*mRNA_p27_inactive;

dk_mRNA_p27_in_use = k_dk_mRNA*mRNA_p27_in_use;

cut_mRNA_p27 = k_cut_mRNA*mRNA_p27_trash;

txl_p27 = unbind_rRNA*(mRNA_p27_cytosol + mRNA_p27_in_use)...

/(mRNA_cytosol + mRNA_in_use);

fold_p27 = ATP_frac_avail*k_fold_p*p27_unfold_conc;

nm_in_p27 = ATP_frac_avail*k_nm_in_p*p27_cytosol_conc;

bind_p27_to_cyclins = bind_cycD_Cdk4or6_to_p27 + bind_cycE_Cdk2_to_p27...

+ bind_cycA_Cdk2_to_p27;

unbind_p27_cyclins = unbind_cycD_Cdk4or6_p27 + unbind_cycE_Cdk2_p27...

+ unbind_cycA_Cdk2_p27;

% p27 is ubiq by SCF(Skp2) and APC(Cdc20) and primarily by KPC

% p27 ubiq by SCF(Skp2) is fast when p27_on_cyclins

% ASSUME APC(Cdc20) and SCF(Skp2) are nuclear, thus can only ubiq p27_nuc and p27_on_cyclins

% ASSUME KPC is cellular

u_p27_by_SCF_Skp2_fast = ATP_frac_avail*k_u_p27_by_SCF_Skp2_fast...

*Skp2_SCF_active_conc;

u_p27_by_SCF_Skp2_slow = ATP_frac_avail*k_u_p27_by_SCF_Skp2_slow...

*Skp2_SCF_active_conc;

u_p27_by_APC_Cdc20 = ATP_frac_avail*k_u_p27_by_APC_Cdc20*Cdc20_APC_active_conc;

u_p27_by_KPC = ATP_frac_avail*k_u_p27_by_KPC*KPC_active_conc;

u_p27_fast = u_p27_by_SCF_Skp2_fast + u_p27_by_APC_Cdc20 + u_p27_by_KPC;

u_p27_slow = u_p27_by_SCF_Skp2_slow + u_p27_by_APC_Cdc20 + u_p27_by_KPC;

u_p27_unfold = ATP_frac_avail*k_dk_p*p27_unfold;

u_p27_cytosol = u_p27_by_KPC*p27_cytosol_conc + ATP_frac_avail*k_dk_p*p27_cytosol;

u_p27_nuc = u_p27_slow*p27_nuc_conc + ATP_frac_avail*k_dk_p*p27_nuc;

u_p27_on_cyclins = u_p27_fast*p27_on_cyclins_conc...

+ ATP_frac_avail*k_dk_p*p27_on_cyclins;

% u_p27_nuc = (u_p27_by_KPC + u_p27_slow)*p27_nuc_conc...

% + ATP_frac_avail*k_dk_p*p27_nuc;

% u_p27_on_cyclins = (u_p27_by_KPC + u_p27_fast)*p27_on_cyclins_conc...

% + ATP_frac_avail*k_dk_p*p27_on_cyclins;

cut_p27 = ATP_frac_avail*k_cut_p*p27_trash_conc;

p27_all_conc = p27_nuc_conc + p27_on_cyclins_conc;

energy_u_p27_by_SCF_Skp2 = ATP_u*u_p27_by_SCF_Skp2_slow*p27_nuc_conc;

+ ATP_u*u_p27_by_SCF_Skp2_fast*p27_on_cyclins_conc;

energy_u_p27_by_APC_Cdc20 = ATP_u*u_p27_by_APC_Cdc20*p27_all_conc;

energy_u_p27_by_KPC = ATP_u*u_p27_by_KPC*p27_cytosol_conc;

% energy_u_p27_by_KPC = ATP_u*u_p27_by_KPC*(p27_cytosol_conc + p27_all_conc);

% 4 Cdk2 RATES

txs_mRNA_Cdk2 = ATP_frac_avail*k_mRNA_txs*polyII*Cdk2_loci_frac...

*(Cdk2_constituitive + E2F_on_DNA);

% txs_mRNA_Cdk2 = ATP_frac_avail*k_mRNA_txs*polyII*Cdk2_loci_frac;

spl_mRNA_Cdk2 = ATP_frac_avail*spl_frac*mRNA_Cdk2_unspl;

nm_out_mRNA_Cdk2 = ATP_frac_avail*k_nm_mRNA*mRNA_Cdk2_nuc_conc;

activate_mRNA_Cdk2 = activate_mRNA*mRNA_Cdk2_inactive/mRNA_inactive;

bind_mRNA_Cdk2 = bind_mRNA*mRNA_Cdk2_cytosol/mRNA_cytosol;

unbind_mRNA_Cdk2 = unbind_mRNA*mRNA_Cdk2_in_use/mRNA_in_use;

dk_mRNA_Cdk2_cytosol = k_dk_mRNA*mRNA_Cdk2_cytosol;

dk_mRNA_Cdk2_unspl = k_dk_mRNA*mRNA_Cdk2_unspl;

dk_mRNA_Cdk2_nuc = k_dk_mRNA*mRNA_Cdk2_nuc;

dk_mRNA_Cdk2_inactive = k_dk_mRNA*mRNA_Cdk2_inactive;

dk_mRNA_Cdk2_in_use = k_dk_mRNA*mRNA_Cdk2_in_use;

cut_mRNA_Cdk2 = k_cut_mRNA*mRNA_Cdk2_trash;

txl_Cdk2 = unbind_rRNA*(mRNA_Cdk2_cytosol + mRNA_Cdk2_in_use)...

/(mRNA_cytosol + mRNA_in_use);

fold_Cdk2 = ATP_frac_avail*k_fold_p*Cdk2_unfold_conc;

nm_in_Cdk2 = ATP_frac_avail*k_nm_in_p*Cdk2_cytosol_conc;

bind_Cdk2_to_cycE = bind_cycE_to_Cdk2;

unbind_Cdk2_cycE = unbind_cycE_Cdk2;

bind_Cdk2_to_cycA = bind_cycA_to_Cdk2;

unbind_Cdk2_cycA = unbind_cycA_Cdk2;

u_Cdk2_unfold = ATP_frac_avail*k_dk_p*Cdk2_unfold;

u_Cdk2_cytosol = ATP_frac_avail*k_dk_p*Cdk2_cytosol;

u_Cdk2_nuc = ATP_frac_avail*k_dk_p*Cdk2_nuc;

u_Cdk2_on_cycE = ATP_frac_avail*k_dk_p*Cdk2_on_cycE;

u_Cdk2_on_cycA = ATP_frac_avail*k_dk_p*Cdk2_on_cycA;

cut_Cdk2 = ATP_frac_avail*k_cut_p*Cdk2_trash_conc;

% 11 Skp2 RATES

txs_mRNA_Skp2 = ATP_frac_avail*k_mRNA_txs*polyII*Skp2_loci_frac;

spl_mRNA_Skp2 = ATP_frac_avail*spl_frac*mRNA_Skp2_unspl;

nm_out_mRNA_Skp2 = ATP_frac_avail*k_nm_mRNA*mRNA_Skp2_nuc_conc;

activate_mRNA_Skp2 = activate_mRNA*mRNA_Skp2_inactive/mRNA_inactive;

bind_mRNA_Skp2 = bind_mRNA*mRNA_Skp2_cytosol/mRNA_cytosol;

unbind_mRNA_Skp2 = unbind_mRNA*mRNA_Skp2_in_use/mRNA_in_use;

dk_mRNA_Skp2_cytosol = k_dk_mRNA*mRNA_Skp2_cytosol;

dk_mRNA_Skp2_unspl = k_dk_mRNA*mRNA_Skp2_unspl;

dk_mRNA_Skp2_nuc = k_dk_mRNA*mRNA_Skp2_nuc;

dk_mRNA_Skp2_inactive = k_dk_mRNA*mRNA_Skp2_inactive;

dk_mRNA_Skp2_in_use = k_dk_mRNA*mRNA_Skp2_in_use;

cut_mRNA_Skp2 = k_cut_mRNA*mRNA_Skp2_trash;

txl_Skp2 = unbind_rRNA*(mRNA_Skp2_cytosol + mRNA_Skp2_in_use)...

/(mRNA_cytosol + mRNA_in_use);

fold_Skp2 = ATP_frac_avail*k_fold_p*Skp2_unfold_conc;

nm_in_Skp2 = ATP_frac_avail*k_nm_in_p*Skp2_cytosol_conc;

% bind_Skp2_to_SCF = k_bind_Skp2_to_SCF*SCF_nuc_conc*Skp2_nuc_conc;

bind_Skp2_to_SCF = k_bind_Skp2_to_SCF*SCF_in_cell_conc*Skp2_nuc_conc;

unbind_Skp2_SCF = k_unbind_Skp2_SCF*Skp2_SCF_active_conc;

u_Skp2_by_APC_Cdh1 = ATP_frac_avail*k_u_Skp2_by_APC_Cdh1*Cdh1_APC_active_conc;

u_Skp2_unfold = ATP_frac_avail*k_dk_p*Skp2_unfold;

u_Skp2_cytosol = u_Skp2_by_APC_Cdh1*Skp2_cytosol_conc...

+ ATP_frac_avail*k_dk_p*Skp2_cytosol;

u_Skp2_nuc = u_Skp2_by_APC_Cdh1*Skp2_nuc_conc + ATP_frac_avail*k_dk_p*Skp2_nuc;

% Skp2 autoubiquinates when bound to SCF and there are no substrates

% substrates are p27, cycD, cycE (free), and RC

% (autoubiquination is dependent on number of molecules, not concentration)

% ASSUME Skp2 is nuclear and APC(Cdh1) is cellular

% IGNORE cycD

% NOTE -- Skp2 binds with cycA to protect it from p27 (Ji et al., 2006)

% substrates_Skp2_all = p27_cytosol + p27_nuc + p27_on_cyclins...

% + cycD_cytosol + cycD_nuc...

% + cycD_Cdk4or6_active + cycD_Cdk4or6_on_p27_inactive...

% + cycE_cytosol + cycE_nuc...

% + E2F_cytosol + E2F_nuc + E2F_Rb_inactive + E2F_on_DNA...

% + RC_cytosol + RC_nuc;

% I want Skp2 to hang around longer (to delay SCF(Btrc)) so I think any RC delays autoubiq

substrates_Skp2_all = p27_nuc + p27_on_cyclins...

+ cycE_nuc...

+ E2F_nuc...

+ RC_nuc + RC_on_DNA + RC_licensed + RC_traversed_by_DNA_poly;

% I want Skp2 to be able to ubiq Wee1 when Btrc is blocked 5-11-07

% substrates_Skp2_all = p27_nuc + p27_on_cyclins...

% + cycE_nuc...

% + E2F_nuc...

% + RC_nuc + RC_on_DNA + RC_licensed + RC_traversed_by_DNA_poly...

% + Wee1_phospho_inactive;

u_auto_Skp2 = ATP_frac_avail*k_u_auto_Skp2*Skp2_SCF_active...

/max(1, substrates_Skp2_all);

u_Skp2_SCF_active = u_Skp2_by_APC_Cdh1*Skp2_SCF_active_conc + u_auto_Skp2...

+ ATP_frac_avail*k_dk_p*Skp2_SCF_active;

cut_Skp2 = ATP_frac_avail*k_cut_p*Skp2_trash_conc;

energy_u_Skp2_by_APC_Cdh1 = ATP_u*u_Skp2_by_APC_Cdh1...

*(Skp2_cytosol_conc + Skp2_nuc_conc...

+ Skp2_SCF_active_conc);

energy_u_auto_Skp2 = ATP_u*u_auto_Skp2;

% 12 Btrc RATES

txs_mRNA_Btrc = ATP_frac_avail*k_mRNA_txs*polyII*Btrc_loci_frac*E2F_on_DNA;

spl_mRNA_Btrc = ATP_frac_avail*spl_frac*mRNA_Btrc_unspl;

nm_out_mRNA_Btrc = ATP_frac_avail*k_nm_mRNA*mRNA_Btrc_nuc_conc;

activate_mRNA_Btrc = activate_mRNA*mRNA_Btrc_inactive/mRNA_inactive;

bind_mRNA_Btrc = bind_mRNA*mRNA_Btrc_cytosol/mRNA_cytosol;

unbind_mRNA_Btrc = unbind_mRNA*mRNA_Btrc_in_use/mRNA_in_use;

dk_mRNA_Btrc_cytosol = k_dk_mRNA*mRNA_Btrc_cytosol;

dk_mRNA_Btrc_unspl = k_dk_mRNA*mRNA_Btrc_unspl;

dk_mRNA_Btrc_nuc = k_dk_mRNA*mRNA_Btrc_nuc;

dk_mRNA_Btrc_inactive = k_dk_mRNA*mRNA_Btrc_inactive;

dk_mRNA_Btrc_in_use = k_dk_mRNA*mRNA_Btrc_in_use;

cut_mRNA_Btrc = k_cut_mRNA*mRNA_Btrc_trash;

txl_Btrc = unbind_rRNA*(mRNA_Btrc_cytosol + mRNA_Btrc_in_use)...

/(mRNA_cytosol + mRNA_in_use);

fold_Btrc = ATP_frac_avail*k_fold_p*Btrc_unfold_conc;

bind_Btrc_to_SCF = k_bind_Btrc_to_SCF*SCF_in_cell_conc*Btrc_cytosol_conc;

unbind_Btrc_SCF = k_unbind_Btrc_SCF*Btrc_SCF_active_conc;

% ASSUME Btrc ubiq by cellular APC(Cdh1)

u_Btrc_by_APC_Cdh1 = ATP_frac_avail*k_u_Btrc_by_APC_Cdh1*Cdh1_APC_active_conc;

u_Btrc_unfold = ATP_frac_avail*k_dk_p*Btrc_unfold;

u_Btrc_cytosol = u_Btrc_by_APC_Cdh1*Btrc_cytosol_conc...

+ ATP_frac_avail*k_dk_p*Btrc_cytosol;

% Btrc autoubiqs (because it is cytoplasmic)

% Btrc autoubiquinates when bound to SCF and there are no substrates

% substrates are Emi1 and Wee1_phospho_inactive (i.e., marked by cycB/Cdk1 and Plk1)

% (autoubiquination is dependent on number of molecules, not concentration)

% substrates_Btrc_all = Emi1_in_cell + Emi1_on_Cdh1 + Emi1_on_Cdc20...

% + Emi1_phospho_inactive...

% + Wee1_cytosol_active + Wee1_phospho_inactive...

% + Cdc25A_cytosol + Cdc25A_nuc_inactive + Cdc25A_phospho_active;

% ASSUME Cdc25A is a substrate only as a checkpoint

substrates_Btrc_all = Emi1_in_cell + Emi1_on_Cdh1 + Emi1_on_Cdc20...

+ Emi1_phospho_inactive...

+ Wee1_phospho_inactive;

u_auto_Btrc = ATP_frac_avail*k_u_auto_Btrc*Btrc_SCF_active...

/max(1, substrates_Btrc_all);

u_Btrc_SCF_active = u_Btrc_by_APC_Cdh1*Btrc_SCF_active_conc...

+ ATP_frac_avail*k_dk_p*Btrc_SCF_active;

cut_Btrc = ATP_frac_avail*k_cut_p*Btrc_trash_conc;

energy_u_auto_Btrc = ATP_u*u_Btrc_by_APC_Cdh1...

*(Btrc_cytosol_conc + Btrc_SCF_active_conc)...

+ ATP_u*u_auto_Btrc;

% 13 Fbw7 RATES

txs_mRNA_Fbw7 = ATP_frac_avail*k_mRNA_txs*polyII*Fbw7_loci_frac*E2F_on_DNA;

spl_mRNA_Fbw7 = ATP_frac_avail*spl_frac*mRNA_Fbw7_unspl;

nm_out_mRNA_Fbw7 = ATP_frac_avail*k_nm_mRNA*mRNA_Fbw7_nuc_conc;

activate_mRNA_Fbw7 = activate_mRNA*mRNA_Fbw7_inactive/mRNA_inactive;

bind_mRNA_Fbw7 = bind_mRNA*mRNA_Fbw7_cytosol/mRNA_cytosol;

unbind_mRNA_Fbw7 = unbind_mRNA*mRNA_Fbw7_in_use/mRNA_in_use;

dk_mRNA_Fbw7_cytosol = k_dk_mRNA*mRNA_Fbw7_cytosol;

dk_mRNA_Fbw7_unspl = k_dk_mRNA*mRNA_Fbw7_unspl;

dk_mRNA_Fbw7_nuc = k_dk_mRNA*mRNA_Fbw7_nuc;

dk_mRNA_Fbw7_inactive = k_dk_mRNA*mRNA_Fbw7_inactive;

dk_mRNA_Fbw7_in_use = k_dk_mRNA*mRNA_Fbw7_in_use;

cut_mRNA_Fbw7 = k_cut_mRNA*mRNA_Fbw7_trash;

txl_Fbw7 = unbind_rRNA*(mRNA_Fbw7_cytosol + mRNA_Fbw7_in_use)...

/(mRNA_cytosol + mRNA_in_use);

fold_Fbw7 = ATP_frac_avail*k_fold_p*Fbw7_unfold_conc;

nm_in_Fbw7 = ATP_frac_avail*k_nm_in_p*Fbw7_cytosol_conc;

bind_Fbw7_to_SCF = k_bind_Fbw7_to_SCF*SCF_in_cell_conc*Fbw7_nuc_conc;

unbind_Fbw7_SCF = k_unbind_Fbw7_SCF*Fbw7_SCF_active_conc;

u_Fbw7_unfold = ATP_frac_avail*k_dk_p*Fbw7_unfold;

u_Fbw7_cytosol = ATP_frac_avail*k_dk_p*Fbw7_cytosol;

u_Fbw7_nuc = ATP_frac_avail*k_dk_p*Fbw7_nuc;

% Fbw7 autoubiquinates when bound to SCF and there are no substrates

% substrates are TF_grow (not on DNA), cycE (bound to Cdk2), and RC (all in nucleus)

% (autoubiquination is dependent on number of molecules, not concentration)

% Assume Fbw7 nuclear

% substrates_Fbw7_all = TF_grow_nuc_conc...

% + cycE_Cdk2_active_conc + cycE_Cdk2_inactive_conc...

% + cycE_Cdk2_on_p27_inactive_conc...

% + RC_nuc + RC_on_DNA + RC_licensed + RC_traversed_by_DNA_poly;

substrates_Fbw7_all = cycE_Cdk2_active_conc + cycE_Cdk2_inactive_conc...

+ cycE_Cdk2_on_p27_inactive_conc;

u_auto_Fbw7 = ATP_frac_avail*k_u_auto_Fbw7*Fbw7_SCF_active_conc...

/max(1, substrates_Fbw7_all);

% u_auto_Fbw7 = ATP_frac_avail*k_u_auto_Fbw7*Fbw7_SCF_active_conc...

% /log10(max(10, substrates_Fbw7_all));

u_Fbw7_SCF_active = u_auto_Fbw7 + ATP_frac_avail*k_dk_p*Fbw7_SCF_active;

cut_Fbw7 = ATP_frac_avail*k_cut_p*Fbw7_trash_conc;
[truncated: 193,573 more chars]
